# Legume Cytosolic and Plastid Acetyl-Coenzyme—A Carboxylase Genes Differ by Evolutionary Patterns and Selection Pressure Schemes Acting before and after Whole-Genome Duplications

**DOI:** 10.3390/genes9110563

**Published:** 2018-11-21

**Authors:** Anna Szczepaniak, Michał Książkiewicz, Jan Podkowiński, Katarzyna B. Czyż, Marek Figlerowicz, Barbara Naganowska

**Affiliations:** 1 Department of Genomics, Institute of Plant Genetics, Polish Academy of Sciences, 60-479 Poznań, Poland; aszc@igr.poznan.pl (A.S.); bnag@igr.poznan.pl (B.N.); 2 Department of Genomics, Institute of Bioorganic Chemistry, Polish Academy of Sciences, 61-704 Poznań, Poland; jpodkow@man.poznan.pl (J.P.); m.figlerowicz@ibch.poznan.pl (M.F.); 3 Department of Biometry and Bioinformatics, Institute of Plant Genetics, Polish Academy of Sciences, 60-479 Poznań, Poland; kwyr@igr.poznan.pl

**Keywords:** legumes, acetyl-coenzyme A carboxylase, evolution, fatty acid synthesis, duplication, synteny

## Abstract

Acetyl-coenzyme A carboxylase (ACCase, E.C.6.4.1.2) catalyzes acetyl-coenzyme A carboxylation to malonyl coenzyme A. Plants possess two distinct ACCases differing by cellular compartment and function. Plastid ACCase contributes to de novo fatty acid synthesis, whereas cytosolic enzyme to the synthesis of very long chain fatty acids, phytoalexins, flavonoids, and anthocyanins. The narrow leafed lupin (*Lupinus angustifolius* L.) represents legumes, a plant family which evolved by whole-genome duplications (WGDs). The study aimed on the contribution of these WGDs to the multiplication of ACCase genes and their further evolutionary patterns. The molecular approach involved bacterial artificial chromosome (BAC) library screening, fluorescent in situ hybridization, linkage mapping, and BAC sequencing. In silico analysis encompassed sequence annotation, comparative mapping, selection pressure calculation, phylogenetic inference, and gene expression profiling. Among sequenced legumes, the highest number of ACCase genes was identified in lupin and soybean. The most abundant plastid ACCase subunit genes were *accB*. ACCase genes in legumes evolved by WGDs, evidenced by shared synteny and Bayesian phylogenetic inference. Transcriptional activity of almost all copies was confirmed. Gene duplicates were conserved by strong purifying selection, however, positive selection occurred in *Arachis* (*accB2*) and *Lupinus* (*accC*) lineages, putatively predating the WGD event(s). Early duplicated *accA* and *accB* genes underwent transcriptional sub-functionalization.

## 1. Introduction

Legumes are the third largest family of higher plants and are second to cereals in agricultural importance. Besides being a well-known source of dietary protein, grain legumes provide about one-third of vegetable oil for human consumption [1]. They are also appreciated as organic fertilizer plants, due to nitrogen fixation in a symbiotic system with bacteria [2]. The origin and diversification of this ecologically and economically important family has been recognized by time-calibrated phylogenies, based on multiple DNA sequences from nuclear and plastid genomes [3,4,5,6]. The last decade has witnessed spectacular progress in the development of next-generation sequencing resources. Numerous legume genomes have been sequenced, such as *Arachis duranensis*, *A. ipaensis* [7], *Cajanus cajan* [8], *Cicer arietinum* [9], *Glycine max* [10], *Lotus japonicus* [11], *Medicago truncatula* [12], *Phaseolus vulgaris* [13], *Trifolium pratense* [14], *Vigna radiata* [15], and *Vigna angularis* [16]. Transcriptome assemblies of more than 1000 plant species, representing all major lineages across the Viridiplantae (green plants) and including 33 legume species, have been released [17,18]. Some of these resources were exploited to improve the evolutionary tree of legumes and shed light on the early divergence of genistoids in the Papilionoideae lineage [19].

Herein, the narrow-leafed lupin (*Lupinus angustifolius* L.), paleopolyploid [20,21] with relatively low chromosome number 2n = 40, small genome size 2C = 1.89 pg [22] and genome sequence length 924 Mbp (according to flow cytometry) or 1037–1153 Mbp (based on K-number/peak depth model) [23,24], was considered as a reference species for the genus and (so far) the whole genistoid clade. Genetic maps of *L. angustifolius*, based on diverse marker systems [25,26,27,28], highlighted ancient whole-genome duplication/triplication events which might have occurred during early evolution of lupins. Bacterial artificial chromosome (BAC) libraries of the nuclear genomes of two *L. angustifolius* cultivars, Sonet [29] and Tanjil [30], were developed. BAC clones were analyzed by fluorescent in situ hybridization (FISH) to assign all linkage groups to chromosomes and identify gene-rich regions [31,32,33,34,35]. Comparative mapping of these regions to the genome assemblies of other legumes revealed the preserved remnants of the hypothetical ancestral genome. These ancient regions remained intact, despite the whole-genome duplication(s) and subsequent chromosomal rearrangements that have hypothetically occurred during the evolution of Papilionoideae [32]. Putatively due to these whole-genome duplication(s), numerous lupin genes are present in several copies, regardless of their relatively low copy number in other legumes. Such observations were done for genes encoding phosphatidyl ethanolamine-binding proteins, chalcone isomerase-fold proteins, isoflavone synthase, as well as for some genes encoding enzymes required for symbiosome activity during nitrogen fixation process [33,35,36,37]. Due to such considerable uniqueness of lupins, accompanied by a well-developed molecular tool-box, we have selected *L. angustifolius* as a reference genome to study the evolutionary conserved group of genes encoding acetyl-coenzyme A carboxylases.

Acetyl-coenzyme A carboxylase (ACCase, E.C.6.4.1.2) is an enzyme from the group of biotin dependent carboxylases, catalyzing acetyl-coenzyme A carboxylation to malonyl coenzyme A [38] and providing the only entry point for all carbon atoms in the fatty acid synthesis pathway [39,40]. Due to the importance of fatty acids, ACCase is an essential enzyme as each cell depends on its own pool of malonyl coenzyme A [41]. Plants are exceptional among Eukaryota because they possess two distinct ACCases: one in plastids and the other in the cytoplasm [38,42]. In the majority of plants, the plastid and cytosolic enzymes are of distinct origin; the first one descended from Cyanobateria whereas the second one originated from the common ancestor of Eukaryota [43,44]. Plastid and cytosolic enzymes are associated with distinct cellular processes as their product cannot be transported across plastid membrane and both pools of malonyl coenzyme A are separated [45]. Plastid ACCase contributes to de novo fatty acid synthesis, whereas the cytosolic enzyme to the synthesis of very long chain fatty acids, phytoalexins, flavonoids and anthocyanins. These compounds are traditionally classified as secondary metabolites, but some of them are indispensable for plant life by forming cuticle or enabling pollen tube development on stigma [39,46,47,48,49,50]. The Papilionoideae plastid ACCase consists of four subunits, each coded by a separate gene: biotin carboxylase (BC, gene *accC*), biotin carboxyl carrier protein (BCCP, gene *accB*), alpha-carboxyltransferase (a-CT, gene *accA*) and beta-carboxyltransferase (b-CT, gene *accD*). The genes coding three of these subunits: *accC*, *accB*, and *accA*, are localized in the nuclear genome whereas *accD* gene is localized in the plastid genome [51,52]. This type of enzyme forms active complex inside plastids and is named heteromeric (ht) ACCase or bacterial type ACCase. The structure of cytosolic ACCase is different because this enzyme is coded by one nuclear gene yielding a very long peptide with domains of BC, BCCP, and CT activity [38,42,53,54,55,56]. This enzyme is named homomeric (hm) ACCase or eukaryotic type ACCase and, despite catalyzing the same reaction as ht ACCase, is phylogenetically more closely related to fungi and animal homologs. The gene structure, protein domains organization, and amino acid sequence suggest that ht ACCases and hm ACCases differentiated before the arising of Eucarya [38].

Gene duplication is a major power driving evolution. Therefore, the knowledge of the mechanisms underlying gene duplications and subsequent processes is fundamental to understand the forces shaping genome contents, evolution, and phylogenetic relationships [57]. In this study, we analyzed the evolution patterns of ht and hm ACCases: two proteins of the same enzymatic activity but quite different structures, genetics, and roles in cellular physiology. *L. angustifolius* BAC clones carrying cytosolic acetyl-coenzyme A carboxylase and plastid subunit (biotin carboxylase, biotin carboxyl carrier protein and α-carboxyltransferase) coding sequences were subjected to comprehensive molecular and in silico analysis to determine the structure, organization, and copy number of those genes. Both genetic and physical mapping were performed to localize these sequences in the *L. angustifolius* genome. Sequences of nuclear genes encoding cytosolic ACCase and subunits of plastid ACCase were revealed from genome and transcriptome assemblies representing all major legume lineages. Phylogeny and selection pressure of these genes as well as patterns of collinearity covering surrounding genome regions were investigated.

## 2. Materials and Methods

### 2.1. Biological Material

Seeds of *Lupinus angustifolius* cv. Sonet were obtained from the Polish Lupin Gene Bank (Poznan Plant Breeding Ltd., Wiatrowo, Poland). Seeds of the mapping population comprising 112 F_8_ recombinant inbred lines (RILs), developed from the cross combination 83A:476 (domestic) × P27255 (wild-type) of *L. angustifolius* [58], were kindly provided by Dr. Hua’an Yang, Department of Agriculture and Food, Western Australia and Dr. Matthew Nelson, Kew Gardens, United Kingdom. BAC clones originated from the nuclear genome library of *L. angustifolius* cv. Sonet (NCBI GenBank LIBGSS_038728), developed using plndigoBAC5 HindIII-Cloning Ready vector [29].

### 2.2. DNA Isolation

Plant genomic DNA was extracted from 3–4 young leaves, using a DNeasy Plant Mini Kit (Qiagen, Hilden, Germany). BAC clone DNA was isolated from single *Escherichia coli* colonies using PhasePrep BAC DNA Kit (Sigma Aldrich, St. Louis, MO, USA). Assessment of DNA quality and concentration was done by absorbance measurements (spectrophotometer NanoDrop 2000; ThermoScientific, Waltham, MA, USA) and agarose gel electrophoresis.

### 2.3. Bacterial Artificial Chromosome Library Screening

Genes of cytosolic acetyl-CoA carboxylase and plastid acetyl CoA carboxylase subunits were assayed in the study (Table 1). Primer sequences were designed using *L. angustifolius* or *M. truncatula* cDNA sequences as a template (Table S1). PCR products were purified (QIAquick PCR Purification Kit; Qiagen), sequenced to confirm the amplification of target sequences, and radiolabeled by random priming (HexaLabel DNA Labeling Kit; Fermentas, Waltham, MA, USA) in the presence of 50 μCi [α-32P]-dCTP. High-density DNA macroarrays containing clones from the *L. angustifolius* nuclear genome BAC library were prepared using GeneTAC G3 robotic station (Genomic Solutions, Ann Arbor, MI, USA) on Hybond N^+^ 22.2 × 22.2 cm nylon filters (AP Biotech, Little Chalfont, UK). Hybridization was carried out as previously described [31,33,36].

### 2.4. Contig Assembly and Bacterial Artificial Chromosome Clone Sequencing

BAC clones showing positive hybridization signals were verified by PCR amplification using BAC DNA as a template and the same primer pairs as those used for probe amplification. The PCR-verified clones were subjected to BAC-end sequencing on the ABI PRISM 3130 XL Genetic Analyzer (Applied Biosystems, Foster City, CA, USA) using pIndigoBAC5 sequencing primers (Table S1).

Sequences obtained with BAC3 and BAC5 primers were named with suffixes “3” and “5”, respectively, and deposited under GenBank NCBI accession numbers MF346806-MF346859. BAC-end sequences (BESs) were analyzed and manually edited with Chromas Lite software (Technylesium, South Brisbane, Australia). BESs were aligned using BLASTn algorithm [59] in Geneious 8.1 [60] to the draft assembly of the *L. angustifolius* genome [23] and overlapping BAC clones were identified. The contigs were verified by PCR amplification using BAC DNA and primers designed on the templates of appropriate BESs. The whole insert sequencing was performed using Miseq platform (Illumina, San Diego, CA, USA) in paired-end 2 × 250 bp approach (Genomed, Warsaw, Poland). Nucleic acid fragmentation was performed in E210× (Covaris, Woburn, MA, USA). Sequencing libraries were constructed using NEBNext^®^ DNA Library Prep Master Mix Set for Illumina (E6040, NEB, Ipswich, MA, USA). Ten BAC clones per lane were sequenced with expected output min. 7.5 Gb. Sequence trimming was done in Cutadapt (https://cutadapt.readthedocs.io/en/stable/) whereas the assembly was in CLC Genomics Workbench.

### 2.5. Functional Annotation of Bacterial Artificial Chromosome Sequences

The process of in silico annotation included de novo detection of specific marks located on the genomic sequences as well as a comparative analysis. Narrow-leafed lupin genome contains more than 57% of repetitive content [23]. Prior to gene prediction, it is recommended to mask repetitive sequences including low-complexity regions and transposable elements [61,62]. Repetitive elements were identified using RepeatMasker Web Server version 4.0.6 with implemented repeat libraries RMLib 20160829 and Dfam 2.0 (A.F.A. Smit, R. Hubley & P. Green, Institute for Systems Biology, Seattle, WA, USA, http://www.repeatmasker.org). The following options were chosen: search engine, cross_match; speed/sensitivity, slow; DNA source, *Arabidopsis thaliana*. DNA sequences were also submitted to Censor [63]. The following settings were applied: Viridiplantae sequence source, report simple repeats, force translated search. Repetitive content was masked according to Repeatmasker/Censor results. To find reference protein sequences BLAST to RefSeq database (NCBI, https://www.ncbi.nlm.nih.gov/refseq/) was performed. The following parameters were applied: *e*-value cut-off, 1E-10; word size, 8; gap existence cost, 5; gap elongation cost, 2; nucleotide match/mismatch scores, 1/−2; max matches in a query range, 1. For each putative gene, protein-based hidden Markov model gene prediction in FGENESH+ [64] was executed, using matching reference RefSeq proteins. Annotated BAC sequences were deposited in NCBI GenBank under accession numbers (MK045264-MK045274).

### 2.6. Fluorescent In Situ Hybridization

Localization of BAC clones in metaphase mitotic chromosomes was performed with the use of fluorescent in situ hybridization (BAC-FISH). BAC probe labelling, chromosome squash preparation, and the FISH procedure was done according to the protocol [34]. The quality of chromosome slides was checked under a phase-contrast microscope BX41 (Olympus, Tokyo, Japan). After FISH, slides were examined with an epifluorescence microscope BX60 (Olympus) using the Cell_F software (Olympus). Images were captured using a CCD monochromatic camera and superimposed using Micrografx Picture Publisher 8 (Corel, Ottawa, ON, Canada).

### 2.7. Droplet Digital PCR

The number of gene copies was determined by the PCR multiplex reaction performed using QX200 Droplet Digital PCR System (Bio-Rad, Hercules, CA, USA). Briefly, each of the 20 μL reactions contained 1× QX200 ddPCR EvaGreen Supermix (Bio-Rad), 200 nM gene-specific primers, 50–80 nM reference (aspartate aminotransferase, *AAT*) gene-specific primers, and *L. angustifolius* cv. Sonet DNA template (in dilutions ranging from 0.125 to 2.0 ng/μL). The PCRs were performed in a C1000 Touch Thermal Cycler (Bio-Rad) with the following cycling conditions: 1× (95 °C for 5 min), 40× (95 °C for 30 s, 57–60.1 °C for 30 s, 72 °C for 45 s), 1× (4 °C for 5 min, 90 °C for 5 min) with 2 °C/s ramp rate. Primer annealing for *ACC*, *accA*, and *accB*/*accC* genes was performed in 60 °C, 60.1 °C, and 57 °C, respectively. The number of droplets and their fluorescence intensity were measured with the QX200 Droplet Reader (Bio-Rad). Data analysis was performed in QuantaSoft (Bio-Rad). To determine the number of gene copies in 1 µL of the analyzed samples, the fraction of positive droplets was background-corrected using template-free control samples and evaluated using Poisson statistics. Primers used for Droplet Digital PCR are provided in Table S1.

### 2.8. Genetic Mapping

Annotated BES and BAC sequences were used to design PCR primers for genetic marker development (Table S1). Amplification was performed using DNA isolated from the parental lines of the *L. angustifolius* mapping population (83A:476 and P27255). Amplicons were recovered directly from the post-reaction mixtures (QIAquick PCR Purification Kit; Qiagen) and sequenced. Allele-specific PCR (AS-PCR) polymorphisms were visualized by 1% agarose gel electrophoresis, whereas nucleotide substitution polymorphisms were solved by the Cleaved Amplified Polymorphic Sequence (CAPS) approach. Restriction sites were identified using dCAPS Finder 2.0 [65]. Digestion products were separated by 2% agarose gel electrophoresis. The segregation data of skeleton markers from the most recent genetic map of the narrow-leafed lupin [23] was used for linkage mapping (Map Manager QTXb20) [66]. Graphical illustration of linkage groups was performed using MapChart [67].

### 2.9. Identification of Homolog Sequences in Legumes

Based on the literature data, reference sequences were selected for *ACC* (AT1G36160 and AT1G36180), *accA* (AT2G38040), *accB* (AT5G16390 and AT5G15530), and *accC* (AT5G35360) genes [68]. These sequences were used to screen the following genome assemblies: *A. duranensis* (accession V14167) and *A. ipaensis* (accession K30076) [7], *C. cajan* [8] (project PRJNA72815, v1.0), *C. arietinum* [9] (v1.0), *G. max* [10] (v. 9.0), *L. japonicus* [11] (v3.0), *L. angustifolius* [23] (Tanjil), *M. truncatula* [12] (strain A17, JCVI v4.0), *P. vulgaris* [13] (v0.9), and *V. radiata* [15] (PRJNA243847). Moreover, the following transcriptome assemblies were mined: lupins—*L. luteus* [69], *L. albus* [70], *L. angustifolius* [25] as well as representatives of other clades, namely: Cercideae (*Bauhinia tomentosa*, *Cercis canadensis*), Detarieae (*Copaifera officinalis*), Mimosoid/MCC (*Acacia argyrophylla*, *Desmanthus illinoensis*, *Gleditsia sinensis*, *Gleditsia triacanthos*, *Gymnocladus dioicus*, *Senna hebecarpa*), Mirbelioids (*Gompholobium polymorphum*), and early diverging Papilionoideae (*Cladrastis lutea*, *Xanthocercis zambesiaca*) [17,18], http://dx.doi.org/10.5061/dryad.ff1tq. The following tblastn parameters were set in Geneious v8.1: matrix, blosum62; maximum *e*-value, 1E-06, word size, 3; gap cost open/extend, 11/1. For each alignment, the sequence of genome region extended by 20,000 nt (or the full transcript sequence) was extracted and submitted to FGENESH+ gene prediction using appropriate *A. thaliana* reference protein. Coding sequences (CDS) shorter than 60% of the length of corresponding *A. thaliana* homolog were discarded from further analysis. Gene structures were visualized using Gene Structure Display Server (GSDS) [71]. 

### 2.10. Microsynteny Analysis

Regions (~200,000 nt) carrying the genes encoding cytosolic ACCase and subunits of plastid ACCase were extracted from the *L. angustifolius* genome, repeat-masked, and blasted against the same genome assemblies as those used in gene prediction (Section 2.9). An identical scenario was applied for BAC clone sequences carrying analyzed genes. Sequence similarity search was performed using CoGe BLAST with parameters: *e*-value, 1E-10; word size, 8; gap existence/extension cost, 5/2; low complexity filter, yes; match/mismatch scores: 1/2. Sequences producing excessive numbers of alignments to loci dispersed over numerous chromosomes were considered as repetitive and removed from total score calculation and collinearity visualization. Genome Synteny Viewer (GSV) [72] and Circos [73] were used for drawing synteny graphs.

### 2.11. Phylogenetic Survey

CDSs with trimmed stop codons were aligned in Geneious using MAFFT 7.017 [74] with the following parameters: alignment, translation; genetic code, standard; translation frame, 1; algorithm, E-INS-I; scoring matrix, blosum 80; gap open penalty, 1.25; offset value, 0. The same parameters were applied for protein sequence alignment. Selection of best-fit model for nucleotide substitution was performed in jModelTest 2.1.10 [75]. The mRNA-based phylogenetic inference was conducted in Mr Bayes 3.2.2 [76] with the following settings: chain length (number of iterations), 1,000,000; subsampling frequency, 200; burn-in length (number of initial iterations excluded), 200,000; nchains (number of chains used for Metropolis-coupled Markov chain Monte Carlo analysis), 6; heated chain temperature 0.30; rate variation, gamma; gamma categories, 4; unconstrained branch lengths, exponential 10; shape parameter, exponential 10. Two substitution models were tested, GTR+I+G and codon M1 (site-specific neutral). For protein-based phylogeny, LG [77] and JTT substitution model were selected [78]; all other parameters were identical. Majority consensus trees were drawn in Geneious. The conservation of selected protein regions was calculated as a percent of identical amino acids at all positions within given region. The analysis was performed using conserved domain database (CDD, https://www.ncbi.nlm.nih.gov/Structure/cdd/cdd.shtml).

### 2.12. Selection Pressure Analysis

Following the topology of the tree, pairs of sequences were selected for selection pressure test. Pairwise translation sequence alignments were done in MAFFT v7.017, using E-INS, BLOSUM 80 and gap open penalty 1.25. Selection pressure parameters, Ka (the number of nonsynonymous substitutions per nonsynonymous site), Ks (the number of synonymous substitutions per synonymous site), and Ka/Ks ratios were calculated in DnaSP 5 [79]. To address the selection pressure in a wider phylogenetic context, branch-site test of positive selection was performed in PAML4 [80] for *Lupinus* and *Arachis* clades. The following foreground branches were analyzed: *Arachis* spp. *ACC*, *G. max ACC*, *Lupinus* spp. *ACC*, *Arachis* spp. *accA1* and *accA2*, *G. max accA*, *Lupinus* spp. *accA*, *B. tomentosa accA2* (with descending branches), *Arachis* spp. *accB1*, *G. max accB1*, *Lupinus* spp. *accB1*, *A. duranensis accB2a* + *A. ipaensis accB2b*, *A. duranensis accB2b* + *A. ipaensis accB2a*, *G. max accB2*, *Lupinus* spp. *accB2*, *Arachis* spp. *accC1*, *Arachis* spp. *accC2*, *G. max accC*, and *Lupinus* spp. *accC*. The numbers of sequences in the alignments were as follows: *ACC*, 24; *accA*, 32; *accB1*, 18; *accB2*, 38; *accC*, 26. Two models were considered: a null model (model = 2, NSites = 2, fix_omega = 0), in which the foreground branch might have different proportions of sites under neutral selection to the background (i.e., relaxed purifying selection), and an alternative model (model = 2, NSites = 2, fix_omega = 1), in which the foreground branch might have a proportion of sites under positive selection. Verification of hypothesis was based on the likelihood ratio test (alternative vs. null model) and *p*-value under Chi-square distribution and 1 degree of freedom. False discovery rate (FDR) correction (0.05) was applied in *Q*-value, https://github.com/StoreyLab/qvalue.

### 2.13. In Silico Gene Expression Assay

To provide insight into gene expression pattern across selected representatives of the legume family, data mining of sequence repositories was performed. Depending on the availability, two types of resources were used: RNA-seq gene expression atlases and microarray archives. The following repositories were used: *A. hypogaea* [81], https://peanutbase.org/gene_expression/atlas; *C. cajan* [82]; *C. arietinum* [83], http://plantgrn.noble.org/LegumeIP/SearchExpArray_All.jsp; *G. max* [84], https://soybase.org/soyseq/; *L. japonicus* [85], https://ljgea.noble.org/v2/index.php; *M. truncatula* [86,87], https://mtgea.noble.org/v3/; *P. vulgaris* [88], http://plantgrn.noble.org/PvGEA/; *V. unguiculata* [89], https://vugea.noble.org/. Normalized values of gene expression were retrieved from these archives. According to the published papers, data were normalized as follows: fragments per kilobase million, FPKM (*A. hypogaea*, *C. cajan*, *V. unguiculata*) [81,82,89], reads per kilobase million, RPKM (*G. max*, *P. vulgaris*) [84,88], rank-based method (*C. arietinum*) [83], quantile method with Robust Multichip Average (*M. truncatula*) [86,87], Z-score (*L. japonicus*) [85]. As the type of data (microarray chip hybridization vs. RNAseq) and normalization procedures (FPKM, RPKM, rank-based method, Z-score) differed between species, we decided to perform two analyses—one using the data as it is (normalized) and the other implementing recalculation to the expression level of a reference gene (standardized). The reference genes were selected based on literature data and the copy number (single copy genes were preferred to avoid ambiguous sequence alignments). The following reference genes were evaluated: *UBC1* (Medtr3g110110.1 and Medtr2g096690.3); *HEL*, Medtr4g134790.1; *TUB*, Medtr7g089120.3; *ATPaseV0*, Medtr7g009590.1; tumorprotein, Medtr2g436620.1. Coefficient of variation (CV = SD/mean) for gene expression of all reference genes among available tissues was calculated. The gene with the lowest average CV value was selected (*ATPaseV0*) to standardize gene expression values. Two datasets were constructed—normalized (unchanged) and standardized (recalculated to the expression level of *ATPaseV0* = 100).

## 3. Results

### 3.1. Both Homologous and Heterologous Probe(s) Were Applicable to Select BAC Clones Carrying Genes Encoding Cytosolic ACCase and Subunits of Plastid ACCase

Screening of the *L. angustifolius* genomic BAC library with probes carrying fragments of ACCase gene sequences (Table 1 and Table S1) resulted in selection of seven clones for cytosolic ACCase gene (*ACC*) and 32 for plastid ACCase subunit genes (*accA*, *accB* and *accC*). PCR verification using DNA isolated from BACs as a template, followed by amplicon sequencing, confirmed the presence of *ACC* gene sequence in four, *accA* in six, and *accB* in five clones. As the *accC* probe originated from *M. truncatula* cDNA sequence, no PCR verification with *L. angustifolius* primers was possible and all 12 clones tagged by hybridization were used in the subsequent analyses.

BESs were obtained for all 27 clones analyzed (accessions MF346806-MF346859). Mapping of BESs to the *L. angustifolius* genome sequence anchored all clones to this assembly and revealed that clones carry two *ACC* (*Lan_ACC1* and *Lan_ACC2*), two *accA* (*Lan_accA1* and *Lan_accA2*), one *accB* (*Lan_accB1*), and one *accC* (*Lan_accC1*) genes. BAC clones that were revealed to carry no target genes were named as “random”. BES mapping confirmed the results of PCR-based verification and yielded in construction of contigs carrying 2 *Lan_accC1* clones, 7 *Lan_accA2* clones, 3 *Lan_ACC1* clones, and 5 *Lan_accB1* clones, as well as two contigs carrying “random” clones (Table S2). Eleven singletons were identified, including those carrying *Lan_ACC2* and *Lan_accA1* genes.

### 3.2. Genes Encoding Cytosolic ACCase and Subunits of Plastid ACCase are Located in Different L. angustifolius Chromosomes

**Figure 1:**
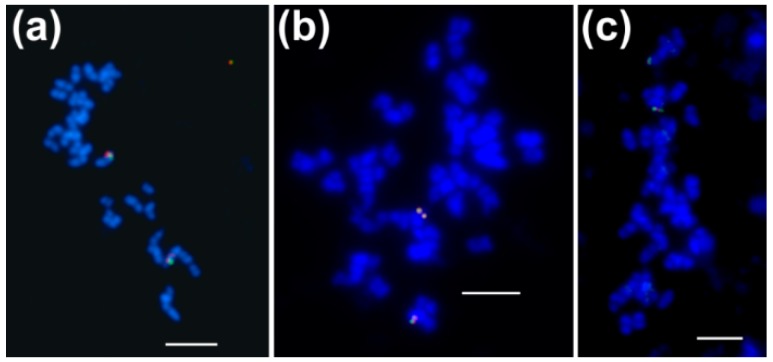
Localization of BAC-based chromosome-specific markers in the *L. angustifolius* genome. Chromosomes were counterstained with DAPI (blue). BACs were labeled with digoxigenin-11-dUTP (green signals) and tetramethylrhodamine-5-dUTP (red signals); (**a**) 009a01 (green) and 126d14 (red) (*accC* sub-library, NLL-06); (**b**) 046i04 (green) and 060f02 (red) (*accA* sub-library, NLL-11); (**c**) 080b11 (green) and 009a01 (red) (NLL-06). Scale bar = 5 μm.

BAC-FISH was performed to localize BAC clones in the mitotic chromosomes of *L. angustifolius* as well as to determine if particular pairs of clones are located in the same regions of the genome. Out of 27 clones tested, 15 yielded single locus signals, including one clone from the each of *Lan_ACC1*, *Lan_ACC2*, *Lan_accA1*, and *Lan_accB1* sub-libraries, two clones from the *Lan_accC1* and four clones from the *Lan_accA2* sub-libraries (Table 2). BAC clones originating from the same contig gave overlapping single locus signals on one pair of homologous chromosomes, whereas those from different contigs showed single locus signals on two different chromosome pairs (Table 2, Figure 1). Singletons were confirmed to be unlinked to any other clone from the analyzed set.

To anchor contigs and singletons to the linkage groups of the narrow-leafed lupin genetic map, molecular markers were developed. Fifty-four sequences derived from 27 clones were used for primer design (Table S1). Expected PCR amplicons were obtained for 63 of 71 primer pairs. The presence of sequence polymorphism between parental lines of the mapping population (83A:476 and P27255) was found for 14 primer pairs, allowing design of twelve CAPS and two AS-PCR molecular markers (accessions: MF398274-MF398299). Segregation in RIL population revealed statistically significant deviation from Mendelian under Chi-square distribution (*p* = 0.05) for two markers, 016J11_5 and 060F02_3 (Table 3). All markers were localized in linkage groups with LOD (logarithm of odds) values from 9.2 to 22.6. Taking into consideration the RIL number in the population (N = 112) and several lines with missing marker data between datasets (resulting from different RIL sets used by research teams during 12 years of the narrow-leafed lupin studies), the statistical significance is conferred by LOD values of about 3–4. LOD values of about 10 can be considered as very high, whereas LOD of about 23 is the maximum possible value. Overlapping clones showed close linkage, as follows: markers 009K06_5 and 040M06_3 (linkage group NLL-14, distance between markers 0.0 cM), 016J11_5 and 060F02_3 (NLL-11, 0.7 cM), 011G20_5 and 069L17_3 (NLL-13, 0.7 cM). All BAC-derived ACCase gene sequences were mapped to linkage groups, as follows: *Lan_ACC1* to NLL-14, *Lan_ACC2* to NLL-15, *Lan_accB1* to NLL-13, and *Lan_accC1* to NLL-06. New markers anchored 12 clones showing single locus BAC-FISH signals, making a considerable improvement of the integrated map of the species, carrying hitherto 73 chromosome-specific landmarks [33]. Segregation data for developed molecular markers was provided in the Table S3.

### 3.3. Lupins and Soybean Have Duplicates of All Nuclear ACCase Genes (*ACC*, *accA*, *accB* and *accC*)

Twelve BAC clones were selected for sequencing including those carrying genes in question (*Lan_ACC1*, *Lan_ACC2*, *Lan_accA1*, *Lan_accA2*, and *Lan_accB1*) and a few “random” ones. The sequencing provided 2.6 mln reads per clone (ranging from 0.4 to 5.1 mln), resulting in an average sequence coverage of 9700×. Full-length insert sequences were recovered for all BACs except two clones from “random” sub-library (089L06 and 096G16). Excluding these two truncated inserts, the length of BAC sequences varied from 31,722 to 162,642 bp, with an average value of 65,220.1 bp (Table 4). This is about 35% less than the average value calculated for the genome BAC library as well as 32.5% less than the average value for clones selected by hybridization from this library in previous studies [31,32,33,90]. BAC clone 011G20 was shown to be fully nested within the 075C05 clone and therefore was discarded from further analyses.

**Figure 2:**
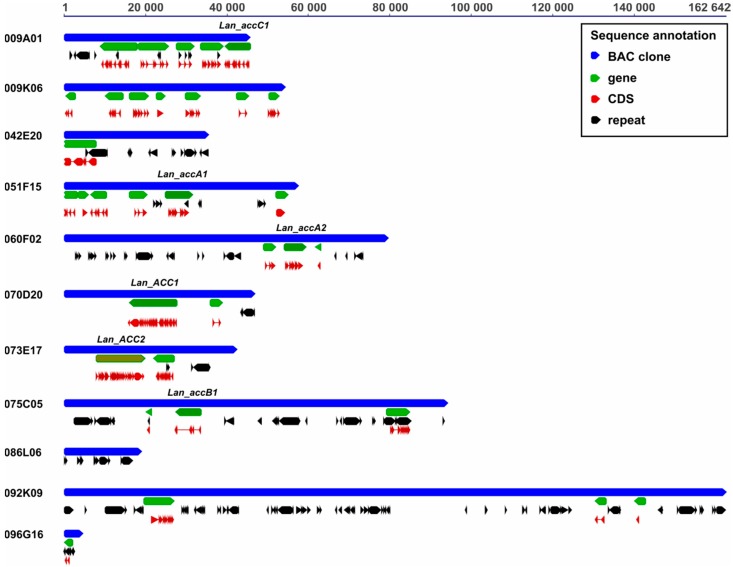
Annotation of *L. angustifolius* BAC sequences. Particular elements are marked by color: blue, BAC clone sequences (009A01, 009K06, 042E20, 051F15, 060F02, 070D20, 073E17, 075C05, 086L06, 092K09, 096G16); black, repetitive elements; green, genes; red, coding sequences. All sequences are drawn to scale provided at the top of the figure (bp).

Repetitive content varied from 2.2% (009K06) to 36.2% (092K09). No correlation between the abundance of repeats and the type of BAC-FISH signal was found, i.e., the low-repeat clone 070D20 (7.1% of repetitive content) yielded dispersed signals whereas the high-repeat clone (37.3% of repetitive content) gave single locus signals. There was no correlation with the type of repeats as well; there were clones carrying LTR/Copia, LTR/Gypsy, and DNA/hAT (051F15) or LTR/Gypsy, LTR/Copia, and DNA/Helitron (060F02) and still yielding BAC-FISH signals single locus type. Gene annotation revealed that BAC clone sequences differed by the number of genes predicted, from 0 in the clone 089L06 to seven in 009K06 (Table 4, Figure 2). The presence of gene *Lan_ACC1* in 070D20, *Lan_ACC1* in 073E17, *Lan_accA1* in 051F15, *Lan_accA2* in 060F02, *Lan_accB1* in 075C05 and *Lan_accC1* in 009A01 clones was confirmed. Two clones carrying *Lan_accA1*/*Lan_accA2* homologs were found to have partially similar gene content, whereas gene content of two clones carrying *Lan_ACC1*/*Lan_ACC2* duplicates was different. Annotated BAC clones were deposited under accession numbers (MK045264-MK045274). Droplet Digital PCR profiling with primers anchored in conserved regions of multiple sequence alignment confirmed the presence of two *ACC*, three *accA*, one *accB1*, and two *accC* genes in the *L. angustifolius* genome (Table S4).

Ten genome and thirteen transcriptome assemblies of species representing all major legume clades were analyzed by BLAST and protein-based gene prediction to reveal the presence of coding sequences (CDS) for cytosolic ACCase and subunits of plastid ACCase. Homologous sequences were identified in all datasets, however, due to low quality of the RNA-seq assembly, only truncated sequences were retrieved for eight species, namely *Cladrastis lutea*, *Desmanthus illinoensis*, *Gleditsia sinensis*, *Gleditsia triacanthos*, *Gymnocladus dioicus*, *Lathyrus sativus*, *Senna hebecarpa*, and *Xanthocercis zambesiaca*. Due to the unpublished stage of the one KP (1000 Plants consortium) initiative hosting these sequences, we were not allowed to access raw data and to re-assemble these transcriptomes using *L. angustifolius* genes as references. To keep the reasonable number of gapped positions in the multiple sequence alignment, species with truncated homolog sequences were not included in the phylogenetic analysis. The lowest numbers of genes encoding both types of ACCase proteins were identified for *Cercis canadensis* and *Copaifera officinalis*, whereas the highest for *L. albus*, *G. max*, and *L. angustifolius*. The latter three species revealed to retain duplicates of all four nuclear genes (*ACC*, *accA*, *accB*, and *accC*). Particular genes differed by copy number: *ACC* and *accC* were the rarest (an average of 1.4–1.5 copies per the assembly), whereas *accB* the most abundant (an average of 3.2 copies) (Table 5, Tables S5 and S6).

### 3.4. The Structure of Nuclear Genes Encoding Cytosolic ACCase and Subunits of Plastid ACCase Is Highly Conserved among the Legume Family

The structure of ACCase genes was evaluated in ten legume species. The genes for cytosolic ACCase have an average length of 12,697 bp (min. 10,145 bp, max. 14,071 bp). Thirty-one exons were predicted for all cyto-ACCase homologs except both *M. truncatula* copies which had 29 exons (Table S5 and Figure S1). An average gene length of *accA* homologs, except *Cca_accA1*, was estimated as 4980 bp (min. 3666 bp, max 7368 bp). *Cca_accA1* revealed a very long first intron, putatively artificially introduced by gene prediction algorithm due to the lack of expected transcription start site. The number of exons varied from 10 in thirteen homologs through 11 in four sequences up to 12 in *Mtr_accA* (Table S5 and Figure S2). The average length of *accB* genes was estimated to be 3830 bp (min. 1653 bp, max. 7463 bp). Exon number was also highly conserved, six in five homologs and seven in thirty (Table S5 and Figure S3). Gene length of *accC* homologs was relatively even, with an average value of 7242 bp (min. 5114 bp, max 9656 bp). The number of predicted exons was 15 for three *accC* genes and 16 for eleven (Table S5 and Figure S4).

**Figure 3:**
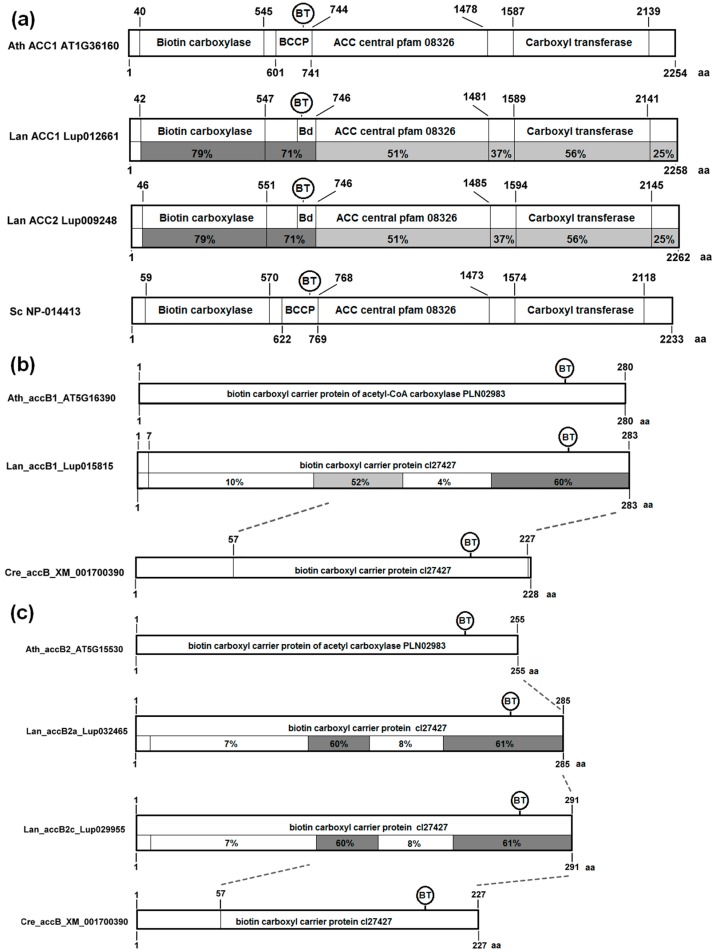
The structure of cytosolic acetyl-coenzyme A carboxylase (ACCase) and biotin carboxyl carrier protein (BCCP) subunits of plastid ACCase. (**a**) cytosolic ACCase gene; (**b**) plastid subunit *accB1* gene; (**c**) plastid subunit *accB2* gene. Amino acid positions and sequence conservation values are provided. Blocks are colored according to sequence conservation level, namely: white, 4–19%; light grey, 20–59%; dark grey, 60–79%; black, 80–100%. Legume sequences used for calculation are provided in the Tables S11, S13 and S14.

The analysis of conserved domain revealed that cytosolic ACCase is composed of three parts of near equal length (Figure 3a). The N-terminal part contains domains of BC and BCCP activity, and the C-terminal region contains CT activity. Between these two parts there is the ACC central domain, pfam08326, specific for Eukaryotes, which does not possess any bacterial homologs and whose function is unknown. The conservation degree of this domain, only slightly lower than this of CT domain, suggests some stabilizing function during evolution.

Subunits of plastid ACCase revealed different degrees of conservation resulting from different selective pressures. The BCCP (*accB*) homologs possess two conserved regions: one shorter in the center of the protein and another longer located on the C-terminus (Figure 3b,c). The biotinylation site is located in the C-terminal conserved region. The C-terminal region of plant BCCP carrying both conserved regions is homologous with cyanobacterial protein, whereas the N-terminal region does not show any significant similarity to bacterial ancestor. The *L. angustifolius* BCCP1 is more conserved than BCCP2, but the difference is very small (60% vs. 52% in the middle domain) and might result from lower number of analyzed proteins in the BCCP1 clade.

**Figure 4:**
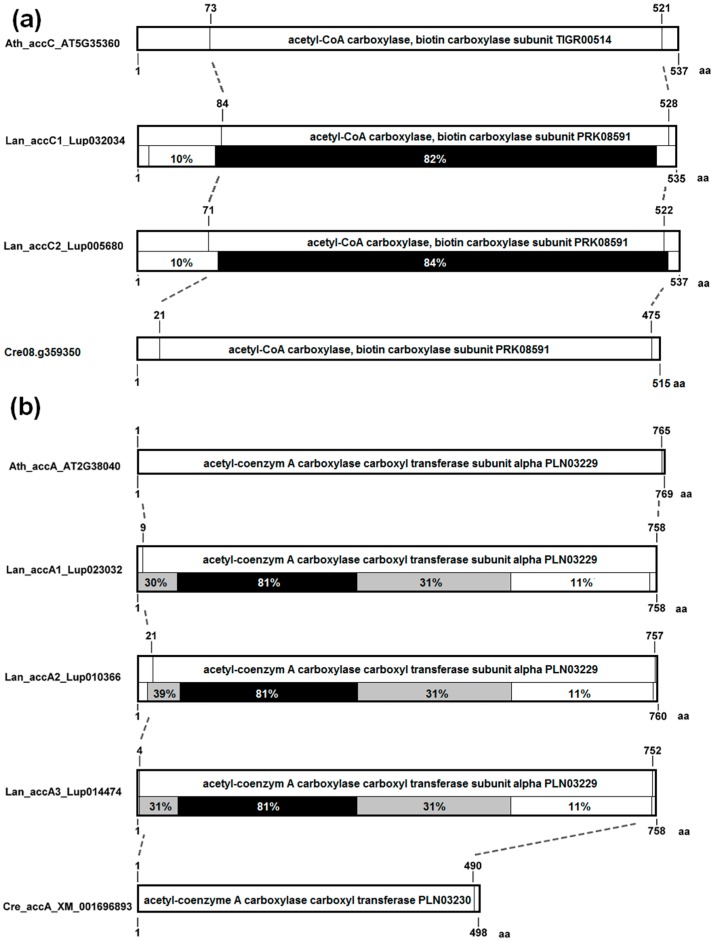
The structure of BC and CTα subunits of plastid ACCase. (**a**) alpha carboxyltransferase, α-CT (gene *accA*); (**b**) biotin carboxyl carrier protein, BCCP (gene *accB*); (**c**) biotin carboxylase, BC, (gene *accC*). Amino acid positions and sequence conservation values are provided. Blocks are colored according to sequence conservation level, namely: white, 4–19%; light grey, 20–59%; dark grey, 60–79%; black, 80–100%. Legume sequences used for calculation are provided in Tables S12 and S15.

The BC subunit (*accC*) is very conserved (large block with ~80% sequence identity) and does not reveal any major remodeling between cyanobacterial and plant proteins (Figure 4a). In CT-alpha (*accA*), the conservation degree decreases gradually from N- to C-terminus, starting from ca. 80% at the N-end part (Figure 4b). The transition from cyanobacterial to eukaryotic protein is associated with multiple inserts scattered along the N-terminal part of the protein.

### 3.5. Whole-Genome Duplication Event(s) Shaped the Evolution of *L. angustifolius* Nuclear Genes for Cytosolic ACCase and Plastid ACCase Subunits

**Figure 5:**
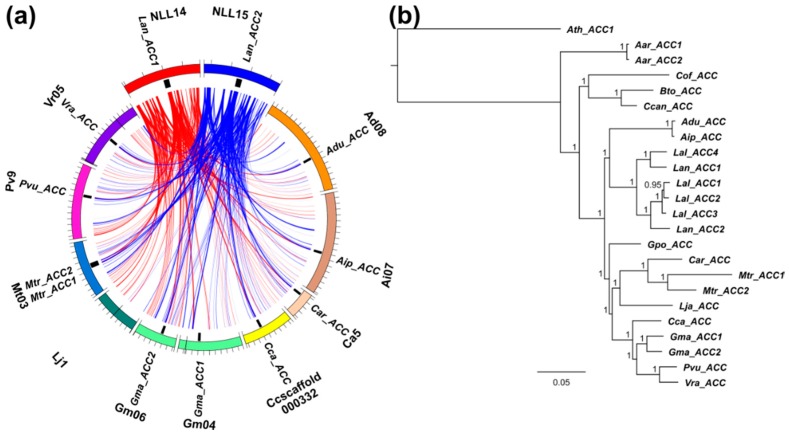
Comparative mapping and phylogenetic inference of legume cytosolic ACCase genes. (**a**) syntenic patterns revealed for narrow-leafed lupin genome regions and corresponding regions of legume chromosomes. Abbreviated species names are provided as follows: Aar, *Acacia argyrophylla*; Adu, *Arachis duranensis*; Aip, *A. ipaensis*; Ath, *Arabidopsis thaliana*; Bto, *Bauhinia tomentosa*; Car, *Cicer arietinum*; Cca, *Cajanus cajan*; Ccan, *Cercis canadensis*; Cof, *Copaifera officinalis*; Gma, *Glycine max*; Gpo, *Gompholobium polymorphum*; Lal, *Lupinus albus*; Lan, *L. angustifolius*; Lja, *Lotus japonicus*; Mtr, *Medicago truncatula*; Pvu, *Phaseolus vulgaris*; Vra, *Vigna radiata*. ACCase gene loci are indicated by black bars. Ribbons visualize homologous links identified by DNA sequence alignments. *L. angustifolius* genomic sequences and homology links are marked by colors: red and blue. (**b**) majority rule consensus tree found in a Bayesian analysis of legume cytosolic ACCase genes. Numbers are posterior probabilities. The length of MAFFT alignment was 6825 nt.

**Figure 6:**
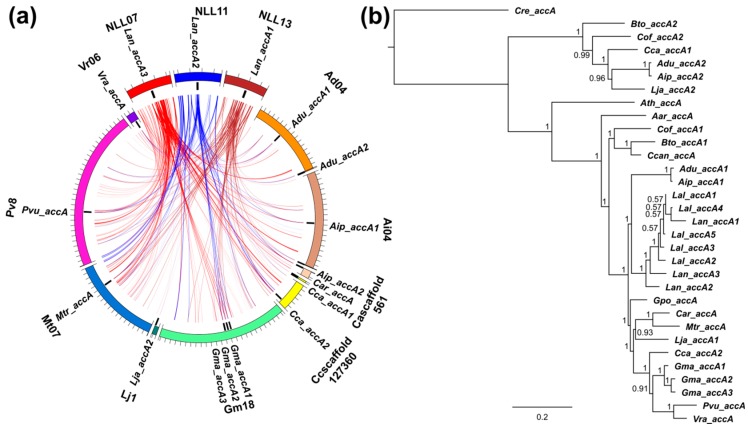
Comparative mapping and phylogenetic inference of legume alpha carboxyltransferase (*accA*) subunit genes of plastid ACCase. (**a**) syntenic patterns revealed for narrow-leafed lupin genome regions and corresponding regions of legume chromosomes. Abbreviated species names are provided as follows: Aar, *Acacia argyrophylla*; Adu, *Arachis duranensis*; Aip, *A. ipaensis*; Ath, *Arabidopsis thaliana*; Bto, *Bauhinia tomentosa*; Car, *Cicer arietinum*; Cca, *Cajanus cajan*; Ccan, *Cercis canadensis*; Cof, *Copaifera officinalis*; Cre, *Chlamydomonas reinhardtii*; Gma, *Glycine max*; Gpo, *Gompholobium polymorphum*; Lal, *Lupinus albus*; Lan, *L. angustifolius*; Lja, *Lotus japonicus*; Mtr, *Medicago truncatula*; Pvu, *Phaseolus vulgaris*; Vra, *Vigna radiata*. ACCase gene loci are indicated by black bars. Ribbons visualize homologous links identified by DNA sequence alignments. *L. angustifolius* genomic sequences and homology links are marked by colors: red, blue and brown. (**b**) majority rule consensus tree found in a Bayesian analysis of legume *accA* genes. Numbers are posterior probabilities. The length of MAFFT alignment was 2445 nt.

**Figure 7:**
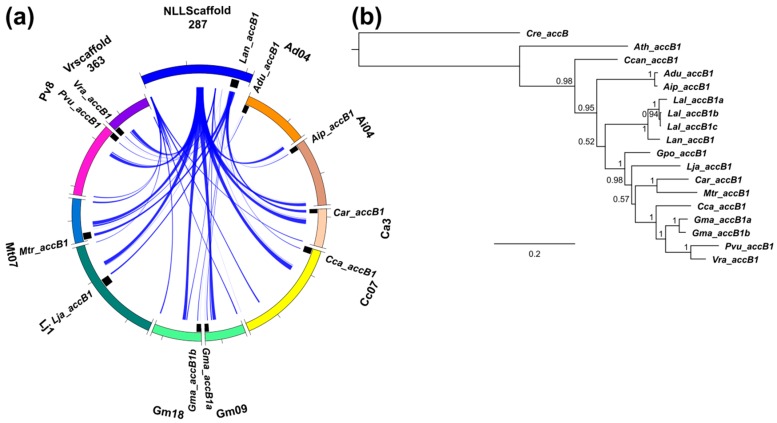

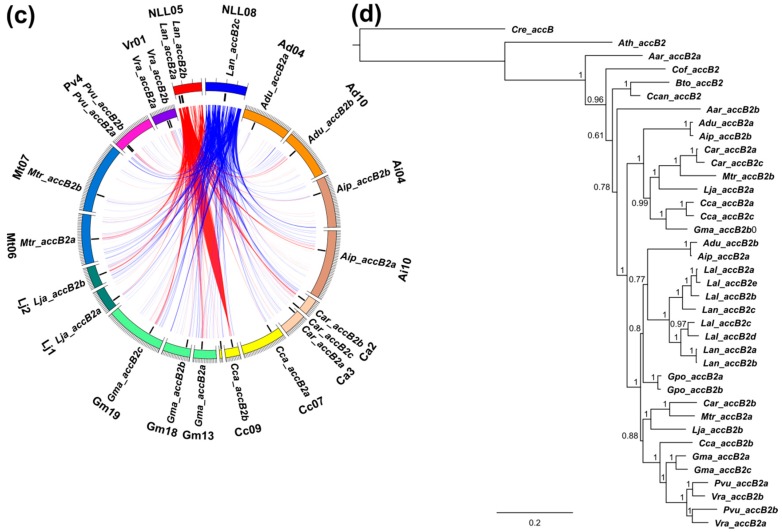
Comparative mapping and phylogenetic inference of legume biotin carboxyl carrier protein (*accB*) subunit genes of plastid ACCase. (**a**,**c**) syntenic patterns revealed for narrow-leafed lupin genome regions and corresponding regions of legume chromosomes. Abbreviated species names are provided as follows: Aar, *Acacia argyrophylla*; Adu, *Arachis duranensis*; Aip, *A. ipaensis*; Ath, *Arabidopsis thaliana*; Bto, *Bauhinia tomentosa*; Car, *Cicer arietinum*; Cca, *Cajanus cajan*; Ccan, *Cercis canadensis*; Cof, *Copaifera officinalis*; Cre, *Chlamydomonas reinhardtii*; Gma, *Glycine max*; Gpo, *Gompholobium polymorphum*; Lal, *Lupinus albus*; Lan, *L. angustifolius*; Lja, *Lotus japonicus*; Mtr, *Medicago truncatula*; Pvu, *Phaseolus vulgaris*; Vra, *Vigna radiata*. ACCase gene loci are indicated by black bars. Ribbons visualize homologous links identified by DNA sequence alignments. *L. angustifolius* genomic sequences and homology links are marked by colors: red and blue. (**b**,**d**) majority rule consensus trees found in a Bayesian analysis of legume *accB1* and *accB2* genes. Numbers are posterior probabilities. The lengths of MAFFT alignments were 933 nt and 1014 nt.

**Figure 8:**
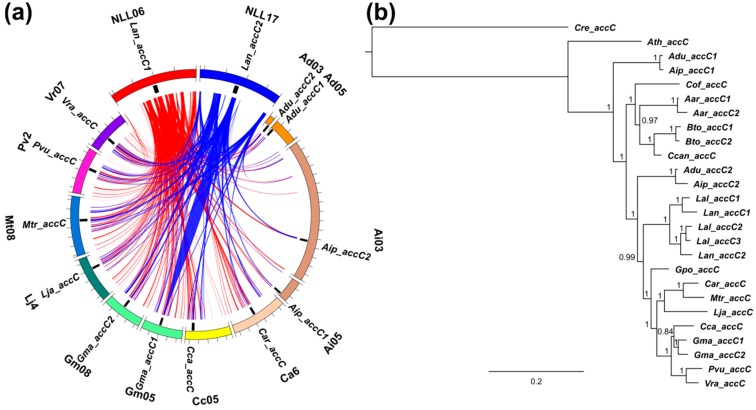
Comparative mapping and phylogenetic inference of legume biotin carboxylase (*accC*) subunit genes of plastid ACCase. (**a**) syntenic patterns revealed for narrow-leafed lupin genome regions and corresponding regions of legume chromosomes. Abbreviated species names are provided as follows: Aar, *Acacia argyrophylla*; Adu, *Arachis duranensis*; Aip, *A. ipaensis*; Ath, *Arabidopsis thaliana*; Bto, *Bauhinia tomentosa*; Car, *Cicer arietinum*; Cca, *Cajanus cajan*; Ccan, *Cercis canadensis*; Cof, *Copaifera officinalis*; Cre, *Chlamydomonas reinhardtii*; Gma, *Glycine max*; Gpo, *Gompholobium polymorphum*; Lal, *Lupinus albus*; Lan, *L. angustifolius*; Lja, *Lotus japonicus*; Mtr, *Medicago truncatula*; Pvu, *Phaseolus vulgaris*; Vra, *Vigna radiata*. ACCase gene loci are indicated by black bars. Ribbons visualize homologous links identified by DNA sequence alignments. *L. angustifolius* genomic sequences and homology links are marked by colors: red and blue. (**b**) majority rule consensus trees found in a Bayesian analysis of legume *accC* genes. Numbers are posterior probabilities. The length of MAFFT alignment used for phylogenetic inference was 1716 nt.

Sequence collinearity of genome regions carrying ACCase genes was analyzed in ten legume species. Highly conserved synteny, evidenced by high total score values of sequence alignments, was observed for *Lan_accB2c*, *Lan_ACC2*, and *Lan_ACC1* regions. The least conserved collinearity was revealed for *Lan_accB1*. Regions surrounding *ACC* and *accA* genes revealed synteny to other genome regions carrying these genes as well as to other regions lacking any appropriate homolog. Genome regions carrying *accB* and *accC* genes were syntenic only to those encoding corresponding homologs. The pattern of synteny indicated lineage-specific duplications of *ACC* (*L. angustifolius*, *M. truncatula* and *G. max*), *accA* (*L. angustifolius* and *G. max*), *accB1* (*G. max*), and *accC* (*L. angustifolius* and *G. max*) genes. *accB2* genes revealed a complex pattern, indicating both lineage-specific and ancestral duplications. No synteny between *accB1* and *accB2* genome regions was observed. The representatives of *accB1* and *accB2* genes were found in all legume taxa having sequenced genomes. The presence of *accB1* and *accB2* genes in the genome of a model plant, *A. thaliana*, highlights early separation of these genes in plant evolution (Tables S7–S10). Patterns of synteny observed for sequences: *Lan_ACC1* vs. *Lan_ACC2*, *Lan_accA1* vs. *Lan_accA2* vs. *Lan_accA3*, *Lan_accB2a*/*Lan_accB2b* vs. *Lan_accB2c*, and *Lan_accC1* vs. *Lan_accC2* were similar to each other, evidencing ancient whole-genome duplication event (Figure 5a, Figure 6a, Figure 7a,c and Figure 8a). In other legume species, the synteny provided evidence for WGD-based origin of *Gma_ACC1*, *Gma_ACC2*; *Gma_accB1a*, *Gma_accB1b*; *Adu_accB2a*, *Adu_accB2b*; *Aip_accB2a*, *Aip_accB2b*; *Cca_accB2a*, *Cca_accB2b*; *Gma_accB2a*, *Gma_accB2b*, *Gma_aacB2c*; *Lja_accB2a*, *Lja_accB2b*; *Mtr_accB2* and *Mtr_accB2b* genes. Tandem duplications contributed to the evolution of *Mt_ACC1*, *Mt_ACC2*; *Gma_accA1*, *Gma_accA2*, *Gma_accA3*; *Lan_accB2a*, *Lan_accB2b*; *Pvu_accB2a*, *Pvu_accB2b*; *Vra_accB2a* and *Vra_accB2b*. Genes *Adu_accA2*, *Aip_accA2*, *Adu_accC1 and Aip_accC1* were revealed to arise by single gene duplications.

### 3.6. Legume ACC Genes Evolved by Lineage-Specific Duplications, Whereas *accA*, *accB* and *accC* Genes Both by Early and Lineage-Specific Duplications

The topologies of majority rule consensus trees of *ACC*, *accA*, *accB1*, *accB2*, and *accC* genes highlighted the recently resolved phylogeny of legumes, with the early diverging *Cercideae*, *Detarieae*, and mimosoid/MCC clades localized at top nodes (Tables S11–S15, Figure 5b, Figure 6b, Figure 7b,d and Figure 8b). *Arachis* and *Lupinus* clades localized at expected positions, ancestral (*accB1* and *accC*) or parallel (*ACC*, *accA* and *accB2*) to the mirbelioid clade represented by *G. polymorphum*. *Lupinus* lineage was found to be descendant (*accB1*, *accC1*) or parallel (*ACC*, *accA* and *accB2*) to *Arachis*.

All lupin *ACC*, *accA*, *accB1*, *accB2*, and *accC* copies arose after the divergence of the *Lupinus* lineage. However, lupin *ACC*, *accA*, *accB2*, and *accC* sequences do not form monophyletic clades which indicates the occurrence of one duplication event before the divergence between *L. angustifolius* and *L. albus.* No such duplication was revealed for the *accB1* clade. The split between *accB1* and *accB2* clades must have occurred very early, before the divergence of *Arabidopsis*. Therefore, we used the *C. reinhardtii* sequence to anchor all trees except *ACC* as the *C. reinhardtii* cytosolic ACCase possesses five long insertions scattered along the peptide. Tree topology indicated that one duplication event of *accB2* might have occurred at the early evolution of Papillionoideae, putatively after the divergence of the mimosoid/MCC clade but before the appearance of dalbergioids (Figure 7b). One duplicate was well preserved in all descendant lineages analyzed, whereas the second was revealed to be currently absent in few of them, including *Lupinus*, *Phaseolus*, and *Vigna*. The issue whether this duplication encompassed genistoid lineage requires high-resolution phylogenetic inference involving extensive sampling of Old- and New World lupins. Such studies are currently hampered by the lack of high quality sequence data.

Despite the general consistence of gene consensus trees with the recently resolved legume phylogeny [19], some exceptions involving one or more branches were revealed in *accA* and *accC* genes. A pair of *Adu_accC1*/*Aip_accC1* was revealed to evolve independently of all other legume homologs (Figure 8b). The possible explanation of such observation is ancient *accC* duplication before the origin of legumes and subsequent elimination of new duplicates in all descendant lineages except *Arachis*. This hypothesis requires further studies. A similar phenomenon was revealed for *accA* genes, where *Bto_accA2*, *Cof_accA2*, *Cca_accA1*, *Adu_accA2*, *Aip_accA2*, and *Lja_accA2* were found to form a clade diverging before the *A. thaliana* lineage (Figure 6b).

### 3.7. Purifying Selection Shaped Evolution of Nuclear Genes Encoding Cytosolic ACCase and Subunits of Plastid ACCase

According to the topology of the majority consensus tree, 53 pairs of duplicated sequences were analyzed, including those located at sister branches and those originating from different clades. The nonsynonymous to synonymous substitution rate (Ka/Ks) ratio analysis revealed that sequence pairs *Lal_accB1a*/*Lal_accB1b* and *Lal_accB1a*/*Lal_accB1c* were subjected to positive selection. These sequences were derived from the transcriptome assemblies, therefore they were confirmed to be expressed. The lack of premature stop codons and the presence of nonsynonymous mutations close to the 5′ end indicate that these sequences are not pseudogenes [91]. For three pairs (*Aip_accA1*/*Aip_accA2*, *Cca_accA1*/*Cca_accA2*, *Lja_accA1*/*Lja_accA2*) the proportion of differences was too high to compute Jukes and Cantor correction. Overlapping parts of *Lan_accB2a*/*Lan_accB2b* sequences were identical. All the remaining pairs were revealed to be under purifying selection. Average Ka/Ks value was the lowest for *ACC* genes (0.16) and the highest for *accB* genes (0.50) (Table S16). The clade encompassing *Bto_accA2*, *Cof_accA2*, *Cca_accA1*, *Adu_accA2*, *Aip_accA2*, and *Lja_accA2* sequences revealed considerably different rates of gene evolution than the clade carrying *Ath_accA* and descendant branches. This was manifested by 34.7-fold higher average Ks value calculated for alignments encompassing sequences from both clades than those carrying only sequences located below *Ath_accA* node.

To evaluate the selection pressure in a wider phylogenetic context, branch-site test of positive selection was performed for *Arachis*, *Lupinus*, and *Glycine* clades (Table S17). Statistically significant *p*-values (<0.05) under Chi-square distribution were revealed for *accB2 Arachis* spp. clade (FDR *q*-value 0.02) and *accC Lupinus* spp. clade (FDR *q*-value 0.07). Positively selected sites were F46 to H and A68 to N in *Adu_accB2a*/*Aip_accB2b* branch, S257 to G in *Adu_accB2b*/*Aip_accB2a* branch, and G445 to N in *Lupinus* spp. *accC* clade. The sites S257 and G445 are located in conserved regions, while the regions carrying F46 and A68 sites are relatively variable.

Branch-site model surveys revealed a very strong positive selection signal (*p* = 2.1E-07) in the clade composed of *Bto_accA2*, *Cof_accA2*, *Cca_accA1*, *Adu_accA2*, *Aip_accA2*, and *Lja_accA2* genes (FDR *q*-value 3E-06). Affected positions were as follows: P75 (substitution to M or I), F182 (to W), K411 (to S), and P723 (to K). First three positions are located in conserved regions. This observation highlights the difference in evolutionary patterns of these homologs and those from the main clade, as visualized by distinct positions on the phylogenetic tree.

### 3.8. Transcription Profiles of *accA*, *accB*, and *accC* Duplicates are Different, Indicating the Possibility of Gene Sub-Functionalization

**Figure 9:**
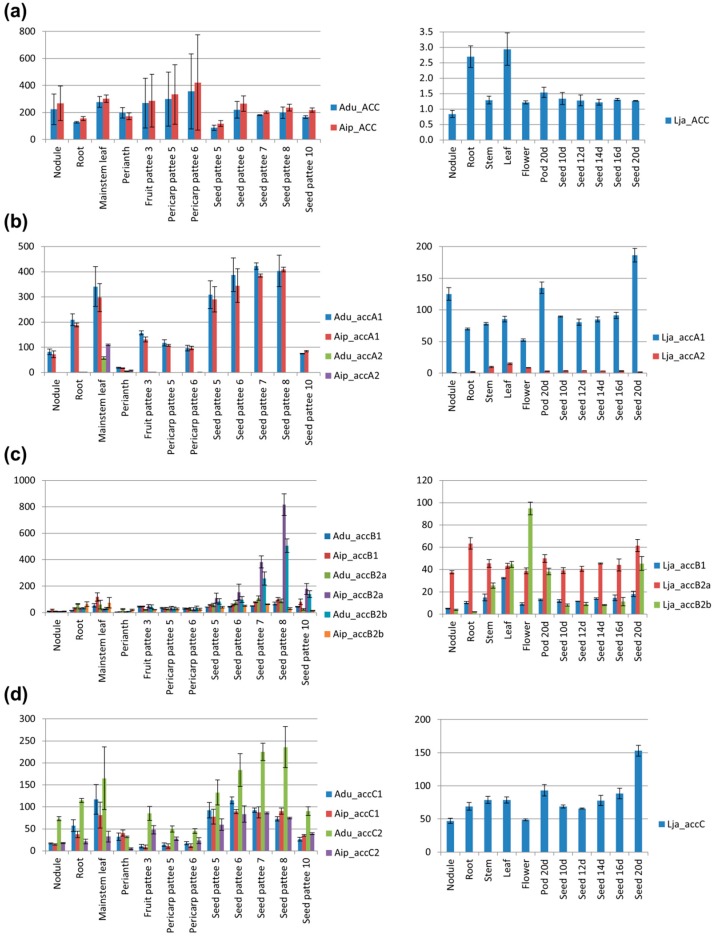
Organ-specific expression profiles of cytosolic ACCase genes and subunits of plastid ACCase genes. (**a**) *ACC* genes; (**b**) *accA* genes; (**c**) *accB* genes; (**d**) *accC* genes. Values were obtained from gene expression atlases of *Arachis hypogaea* [81], https://peanutbase.org/gene_expression/atlas and *Lotus japonicus* [85], https://ljgea.noble.org/v2/index.php, and recalculated to the level of *ATPaseV0* gene. “Pattee” refers to developmental stage (original term used in the peanut database), 10d, 12d etc. are days after pod development.

To address the issue of possible transcriptional sub-functionalization of ACCase duplicates, transcriptome-derived datasets were screened and gene expression across species representing all main descendant lineages of Papilionoideae was evaluated. Some duplicates were not found in gene expression atlases, mostly due to lack of corresponding probe set or annotated gene. However, the number of identified homologs was sufficient to provide insight into differential gene expression patterns among several tissues—nodules, roots, leaves, stems, flowers, seeds and pods (Figure 9, Tables S18 and S19).

High expression of cytosolic ACCase was observed in roots, young leaves, and seeds or pods during maturation in the majority of analyzed species (Figure 9a). The two *G. max* genes (*Gma_ACC1* and *Gma_ACC2*) revealed very similar expression patterns which suggests that their divergence is very recent or that there is no pressure for functional differentiation.

*L. japonicus*, *C. cajan*, *A. duranensis*, and *A. ipaensis* possess two *accA* genes assigned into two clades: the well-separated minor one, grouping also *B. tomentosa* and *C. officinalis* homologs, and the major one grouped together with the remaining Papilionoideae *accA* genes (Figure 6b). Interestingly, *accA* genes from the distinct, minor clade (*Adu_accA2*, *Aip_accA2*, *Lja_accA2*, *Cca_accA1*) showed a similar expression profile, remarkably different from the main clade (Figure 9b). They were expressed on a much lower level with the highest expression in leaves whereas *accA* genes from the main clade were expressed in all analyzed organs with the highest level observed in leaves and during seed or pod development. *L. japonicus* and *M. truncatula accA* genes from the main clade were highly expressed also in nodules (on similar level as in leaves). High expression in roots, on a similar level as that in leaves, was revealed for *Cca_accA2*, *Mtr_accA* and *V. unguiculata accA*. In *G. max*, the expression pattern of three closely related *accA* genes was similar, however for *Gma_accA3* it was almost stable, while *Gm_accA2* considerably differed between organs, especially nodules and leaves.

A high level of *accB* transcripts was found in leaves, seeds and pods, in particular cases showing seed/pod developmental stage specificity (Figure 9c). *L. japonicus*, *M. truncatula*, and *V. unguiculata* also revealed high expression of *accB* genes in flowers, which might be associated with complex regulation during pollen–stigma interactions and on early stages of embryo development. Comparative analysis of the expression profile of *accB* duplicates highlighted hypothetical sub-functionalization as the *accB1* genes had usually the highest level of expression in leaves, whereas for *accB2* genes it was in seeds, pods, stems, or flowers. Such an observation was made for *L. japonicus*, *C. cajan*, *C. arietinum*, *P. vulgaris*, *G. max*, and *V. unguiculata* homologs. Organ specialization was visualized in *L. japonicus* by diverse expression profiles of three *accB* duplicates. On the other hand, expression of *M. truncatula* and *P. vulgaris accB* duplicates differed rather in level than in pattern.

Transcriptional activity of *accC* genes was associated with leaf tissue and with maturation of seeds or pods (Figure 9d). In *L. japonicus*, *C. cajan* and *M. truncatula* high expression was observed in roots, nodules, or flowers. Summarizing, *accC* revealed expression patterns similar to those of *accA*. Moreover, transcription profiles of duplicated *accC* genes (from *G. max*, *A. ipaensis*, and *A. duranensis*) were generally similar to each other. However, the statistically significant difference was observed between *Adu_accC1* and *Adu_accC2* transcription level during seed development, which might be associated with their functional differentiation. 

## 4. Discussion

### 4.1. Bacterial Artificial Chromosome-Based Approach Is Still Efficient in Current Genomic Analyses

Next generation sequencing (NGS) methods started the new era in genomics. Nevertheless, traditional, more direct approaches are still incorporated in many studies concerning gene identification and characterization, especially for species without the sequenced genome or in a draft phase of the assembly. Current narrow-leafed lupin pseudochromosome assembly has a length of 470 Mbp, whereas the estimated genome size based on cytogenetics or k-mer analysis is about 924–1153 Mbp [22,23,24]. Therefore, this assembly provides information on 41–51% of the expected narrow-leafed lupin genome sequence. It should be noted that the first *G. max* pseudochromosome assembly covered ~85% of the expected genome sequence, so twice more than the *L. angustifolius* one [10]. Moreover, the current narrow-leafed assembly was obtained only by linkage mapping and synteny-based approach with no verification by physical mapping methods. The assembly is composed of many small contigs (average length 758 bp), which are organized in larger scaffolds with numerous gaps and tracks of undefined nucleotides. Taking into consideration that ACCase genes are large structures carrying numerous exons, there was a non-negligible risk of a gap or misassembly inside a gene. Thus, in our study the genome sequence was used to localize analyzed genes. However, we decided also to use a BAC-based approach as a reliable method providing direct visualization of particular sequence probes on a chromosome.

Genome BAC libraries, including *L. angustifolius* libraries, served as very useful tool to select genome regions carrying genes of interest as well as to support high throughput data analysis and genome assembly [23,30,31,32,33]. Moreover, BAC-based assembly is still considered as efficient approach for studying complex genomes, where polyploidy or high repeat content hampers whole-genome shotgun sequencing [92,93]. BAC libraries have also been recently used for gene family studies, i.e., fructokinase gene family in *Saccharum* [94] as well as phosphoethanolamine binding proteins in *Lupinus* [36,90]. The use of gene and species specific probes to screen BAC libraries ensures accurate selection of research material. In our studies the number of verified BAC clones selected based on *ACC, accA, accB* homogenic hybridization varied from four to six, whereas the use of heterogenic *M. truncatula accC* gene probe resulted in a higher number of hybridization signals, including several false positives. BAC library approach was beneficial, although did not allow us to find all *accB* genes due to high sequence differentiation between *accB1* and *accB2* clades. In this case, sequence comparative mapping among legumes was indispensable to reveal all lupin homologs. Sequence mining based on closely related reference genes is a commonly used approach in genome-wide studies [95,96].

### 4.2. Nuclear Genes Encoding Cytosolic ACCase and Plastid ACCase Subunits Evolved by Whole-Genome Duplication

Whole-genome duplications (WGDs) are hypothesized to have occurred frequently during plant evolution [97]. It is anticipated that a WGD event predated the differentiation of all extant seed plants (dated ~319 million years ago, mya) and another such event occurred in the common ancestor of all extant angiosperms (~192 mya) [98]. Early diversification of the core eudicots could be associated with old genome triplication (~117 mya), as revealed by phylogenomic approach [99]. These duplications have putatively driven the evolution of regulatory genes controlling seed and flower development which resulted in further dominance of seed plants on Earth [100]. Legume expansion could be also anchored in an ancient WGD, which hypothetically occurred in the progenitor line of Papilionoideae. This WGD was dated to about 44–65 mya and putatively launched the divergence of ancient lineages of Papilionoideae [9,19,101,102,103]. The remnants of such an event have been found in numerous clades including Xanthocercis, Cladrastis, dalbergioids (*Arachis* spp.), genistoids (e.g., *L. angustifolius*), millettioids (*P. vulgaris*, *G. max*, *C. cajan*, *V. radiata*), galegoids (*M. truncatula*, *L. japonicus*, *C. arietinum*), [19,101,104,105,106]. Besides this major ancestral WGD, additional events occurred in early evolution stages of descendant lineages, including Mimosoideae-Cassiinae-Caesalpinieae, Detarieae, Cercideae and *Lupinus* clades, dated roughly ~30–55 mya [19]. Sequence-based comparative mapping provided compelling evidence for large-scale duplication and/or triplication in the *L. angustifolius* genome, highlighting one or more ancestral polyploidy events [26]. *Lupinus* spp. WGD is considered to have happened before the divergence of New World and Old World clades, however no genus-wide phylogenetic support has been provided hitherto [19,35]. It should be emphasized that some legume lineage-specific WGDs occurred relatively recently, i.e., 13 mya in *G. max* and several mya in *Arachis* [10,19]. Here, we demonstrated that *L. angustifolius* cytosolic ACCase duplicates *Lan_ACC1* and *Lan_ACC2* as well as plastid ACCase subunit duplicates of *accA* (*Lan_accA1*, *Lan_accA2* and *Lan_accA3*), *accB* (*Lan_accB2a/b* and *Lan_accB2c*) and *accC* (*Lan*_accC1 and *Lan_accC2*) originated from lineage-specific WGD, whereas the pair of genes *Lan_accB2a* and *Lan_accB2b* evolved by local tandem duplication. It was well-evidenced by cytogenetic and comparative mapping that WGD shaped the evolution of numerous *L. angustifolius* genes, including genes from phenylpropanoid pathway (chalcone isomerase, chalcone isomerase like, fatty acid binding protein and isoflavone synthase) [35,37], phosphatidylethanolamine binding protein family [36] as well as genes involved in symbiotic interactions and nitrogen fixation (early nodulin, nodulin 26-like, phosphoenolpyruvate carboxylase, and glutamine synthetase genes) [33].

Direct counting of all WGD events which are assumed to have occurred during plant evolution would lead to the conclusion that even the relatively small genome of *Arabidopsis* should carry the remnants of at least five ancestral duplications but obviously it does not have so many duplicates [100]. There are evolutionary mechanisms influencing newly arisen gene homologs which result in their elimination, pseudogenization or survival, usually with some sub- or neo-functionalization [107]. We observed that *L. angustifolius* nuclear genome retained relatively high number of ACCase gene duplicates, involving both cytosolic enzyme and plastid subunits. The same phenomenon was observed for other legume species, particularly *G. max* and *L. albus*.

### 4.3. Functional Differentiation of Duplicated ACCase Genes

Homomeric ACCase provides malonyl-CoA for fatty acid elongation as well as polyketide biosynthesis, malonylation reactions, and other specialized metabolic processes [47]. *G. max ACC* genes, *Gma_ACC1* and *Gma_ACC2*, are paralogs which arose after *G. max* speciation and did not reveal any functional specialization. The short time period from their divergence might be one of the factors responsible for high similarity of their expression profiles. *M. truncatula* also possesses two paralogous ACCases, which reveal stronger sequence diversification than *G. max* proteins, but expression pattern was accessible only for *Mtr_ACC2*, due to the lack of *Mtr_ACC1*-specific probe set. The high level of the cytosolic ACCase mRNA level observed in analyzed legume transcriptomes reflects a high demand for fatty acid elongation, needed for the synthesis of cuticular wax, which is deposited on the surface of developing leaves to protect them against water loss and environmental stresses [108,109]. Moreover, malonyl-CoA pool produced by the cytosolic ACCase is also used for biosynthesis of flavonoids including pigments, signaling, and defense compounds [48,49]. High expression of ACC in leaves might be associated with wax synthesis and cuticle formation whereas expression in nodules (as observed for *Adu_ACC* and *Aip_ACC*) might be part of plant mechanisms controlling symbiotic bacteria development within plant cells [110,111]. In seeds and/or pods, during their development, expression of cytosolic ACCase is associated with formation of embryo and protection of seeds against water (both loss and penetration) [112].

The *accA* gene encodes carboxyl transferase alpha subunit of plastid ACCase, which is associated with fatty acid formation. The activity of this gene is important for phospholipid synthesis and cellular and plastid membranes formation as well as oil deposition [38,113,114,115]. Papilionoideae *accA* genes are divided into two sub-clades: the main clade carrying representatives from all studied species and the early diverging clade. The genes from early diverging clade (*C. cajan*, *L. japonicus, A. duranensis* and *A. ipaensis*) revealed much lower expression in all analyzed organs than the genes from the main clade. The strong difference in expression levels indicates evolutionary conserved functional specialization between these clades. The situation in *G. max* is quite different which has three *accA* homologs, all from the main *accA* clade, showing similar expression patterns. *Gma_accA2* high activity in leaves and *Gma_accA1* high activity at the end of seed maturation may suggest that these paralogous genes are at the early stage of functional diversification.

Genes encoding BCCP are divided into two clades, *accB1* and *accB2*, which evolved before the origin of Papilionoideae. Whereas in *A. thaliana* BCCP1, At5g16390, is the major gene and the second homolog BCCP2, At5g15530, is not indispensable, in legumes genes from the clade BCCP1 appeared to be lacking in some lineages, whereas genes from BCCP2 were found in every species analyzed. Also the frequency of gene duplications in Papilionoideae BCCP2 clade was much higher than in BCCP1. A considerably higher number of *accB* genes found in all analyzed plants, in comparison with genes for other pt-ACCase subunits, indicates that the genes for pt-ACCase subunits are under different selection forces. These differences are probably associated with the BCCP role in regulation of the complex enzyme activity. The BCCP-dependent regulatory mechanisms involve posttranslational modification, BCCP dimerization, BCCP-related BADC negative regulatory proteins competition during enzyme assembly, BCCP interactions with signal integrator PII protein, as well as genetic control by the WR1 transcription factor [47,116,117,118,119]. The other forces driving BCCP evolution might be associated with nuclear–cytoplasmic conflict between BCCP and CT-beta, as it was identified in *Pisum sativum* [120]. Indeed, the pair of nuclear *Bccp3* and plastid *accD* genes seems to act like speciation genes in pea, hampering successful crossing of wild representatives with cultivated forms [120]. The diversity of regulatory mechanisms depending on BCCP might be responsible for frequent conservation of newly aroused homologs and their subsequent specialization. Nevertheless, in all analyzed plants, the organs of the highest expression of *accB* genes were leaves and seeds, probably associated with high demand for fatty acids either for phospholipid membranes in plastids of mesophyll cells or as a storage material in seeds [38,39,121,122,123].

The majority of legume species with accessible transcriptomic data possess only one *accC* gene, except *A. duranensis*, *A. ipaensis* and *G. max* which carry two homologs. However, given the very low apparent expression of some loci (Figure 9), it is possible that a single transcriptome sequence for some taxa did not reveal some of the paralogues. *Gm_ accC1* and *Gm_accC2* are paralogs with similar expression patterns indicating that there was no pressure for their functional diversification or simply there was not enough time for such a process. In *Arachis* spp., *accC* duplication occurred before *A. duranensis* and *A. ipaensis* differentiation and these genomes contain two copies of *accC1* and *accC2*. The similar expression patterns of *Adu_accC1* and *Aip_accC1* orthologs suggest that their functions were preserved during evolution. Contrarily, *Adu_accC2* and *Aip_accC2* genes revealed different expression patterns, visualized by high expression of *Adu_accC2* in nodules and roots as well as different expression profile during seed development. These differences suggest that *A. duranensis* and *A. ipaensis accC2* orthologues are under different selective pressures.

### 4.4. Selection Constraints of Genes Encoding Cytosolic ACCase and Subunits of Plastid ACCase

Calculation of the nonsynonymous to synonymous substitution rate (Ka/Ks) ratio performed for 53 pairs of duplicated sequences revealed that nuclear genes encoding cytosolic ACCase and subunits of plastid ACCase have been subjected to strong purifying selection. Only a few examples of positive (*Lal_accB1a* vs. *Lal_accB1b*/*Lal_accB1c*) or neutral (*Aar_accC1* vs. *Aar_accC2*) selection in this pairwise comparison were identified. Cytosolic ACC-ase has lower average of Ka/Ks ratio (0.16) than plastid subunits (0.48). The observation of negative selection pressure is consistent with the enzyme position in the biosynthesis pathways as well as with general importance of ACCase as a key metabolic enzyme [124]. Plastid ACCase is the first enzyme in lipid biosynthesis pathway whereas cytosolic enzyme is pivotal for variety secondary metabolism reactions [38,39,49,108]. In general, genes encoding upstream enzymes in molecular pathways evolve more slowly and are subjected to stronger purifying selection than the downstream ones [125]. The relation between enzyme position in the pathway and selective pressure acting on its gene was identified in numerous studies from different metabolic pathways [126,127]. It has been hypothesized that natural selection may act discriminatively on enzymes with high flux control, usually located at the beginning of the pathway [128]. Moreover, nonsynonymous nucleotide substitutions in genes of upstream enzymes may not be preserved because they may have more deleterious impact on final products than similar changes in genes of downstream enzymes [129]. Both cytosolic and plastid ACCases are enzymes affecting numerous downstream products and as such should be under purifying selection. Hence, other factors might have contributed to observed difference of selection constraints between genes encoding cytosolic enzyme and plastid subunits of ACCase.

Branch-site model analysis evidenced that positive selection might have occurred during evolution of *accB2* gene in *Arachis* spp. and *accC* gene in *Lupinus* spp. Positive selection had putatively acted in these lineages at their early evolution stage, before the divergence into particular species. Following gene duplication, further amino acid changes were preserved by purifying selection. Similar marks of transient evolutionary episode of positive selection in *Lupinus* spp. were revealed for isoflavone synthase genes and were suggested to occur before the whole-genome duplication episode and divergence of *L. angustifolius*, *L. albus*, and *L. luteus* [37].

## 5. Conclusions

Nuclear genes encoding cytosolic ACCase and plastid ACCase subunits in legumes evolved by whole-genome duplication. Further evolution of these genes was shaped by strong purifying selection. Legume plastid ACCase subunit genes differ by copy number: the most abundant are accB genes, what reflects the regulatory role of BCCP subunit in pt-ACCase activity. During evolution of particular legume lineages duplicated plastid ACCase subunit genes underwent transcriptional sub-functionalization.

## Figures and Tables

**Table 1:** Characterization of sequence-defined probes used for *Lupinus angustifolius* nuclear genome library screening.

Probe	Accession Number of Sequence Used for Primer Design	Accession Number of Probe Sequence	Tm ^1^	Probe Size (bp)	Primer Pairs
La ACC	XM_003638746	MF378602	58 °C	404	Pri-129 + Pri-131
La BCCP	XM_003534455	MF378603	56 °C	251	Pri-139 + Pri-140
La CT-α	NM_001249264	MF378604	58 °C	437	Pri-142 + Pri-145
Mt BC	XM_003630560	MF378605	60 °C	1141	J08-35 + J08-36

^1^ Tm, annealing temperature. ACC: cytosolic acetyl-coenzyme A carboxylase; BCCP: biotin carboxyl carrier protein; CT: alpha-carboxyltransferase; BC: biotin carboxylase.

**Table 2:** Direct visualization of bacterial artificial chromosome (BAC) clones distribution in *Lupinus angustifolius* chromosomes.

	**009k09**	**073e17**	**002f03**	**005g02**	**009a01**	**049f04**	**089l06**	**096g16**	**126d16**	**011g20**	**016j11**	**046i04**	**048n08**	**051f15**	**060f02**
**009k06**	-														
**073e17**	N *	-													
**002f03**	N	N	-												
**005g02**	N/A	N/A	N	-											
**009a01**	N	N	N	N	-										
**049f04**	N	N/A	N/A	N/A	N/A	-									
**089l06**	N/A	N	N	N	N	N	-								
**096g16**	N/A	N	N	N/A	N	N/A	N	-							
**126d14**	N	N	N	N	Y	N	N	N	-						
**011g20**	N	N	N	N/A	N	N/A	N/A	N/A	N	-					
**016j11**	N	N	N	N/A	N	N/A	N	N	N	N	-				
**046i04**	N	N	N	N/A	N	N/A	N	N	N	N	Y	-			
**048n08**	N	N	N	N/A	N	N/A	N	N	N	N	Y	Y	-		
**051f15**	N	N	N	N/A	N	N/A	N/A	N	N	N/A	N	N	N	-	
**060f02**	N	N	N	N/A	N	N/A	N	N	N	N	Y	Y	Y	N	-

Y—pairs of BAC clones hybridized to the same chromosome pair; N—pairs of BAC clones hybridized to different chromosome pairs; N/A, not analyzed.

**Table 3:** Genetic markers developed and their localization on the *L. angustifolius* linkage map.

Marker	Marker Type	Enzyme	Products 83A:476 (bp)	Products P27255 (bp)	Chi-Square *p*-Value	NLL	Distance to Other Markers (cM)	LOD Values
002F03_5	CAPS	MnlI	315, 168, 59, 58	209, 168, 106, 59, 58	0.526	NLL-10	1.3, 3.0	20.3, 14.7
005G02_3	CAPS	TaqI	352, 268, 109	292, 267, 109, 60	0.332	NLL-03	0.6, 1.8	22.3, 14.0
009K06_5	CAPS	MjaIV	289, 196, 84	485, 84	0.272	NLL-14	0.0, 0.8	16.9, 17.9
011G20_5	PCR	-	624	-	0.546	NLL-13	0.7, 2.8	18.8, 16.1
016J11_5	CAPS	MaeIII	353	254, 99	0.019	NLL-11	1.4, 0.7	17.4, 20.9
040M06_3	CAPS	DdeI	350, 303	350, 207, 96	0.174	NLL-14	0.8, 0.0	16.7, 16.9
042C13_3	CAPS	HphI	280, 223, 162	385, 280	0.669	NLL-08	0.0, 1.4	22.6, 18.6
051F15_5	CAPS	MfeI	342, 273	615	0.652	NLL-13	3.6, 8.5	14.6, 9.2
060F02_3	CAPS	AciI	158, 67	225	0.003	NLL-11	0.7, 1.3	20.9, 19.7
069L17_3	CAPS	Hpy188I	467	353, 112	0.583	NLL-13	0.7, 0.7	19.7, 18.8
073E17_5	CAPS	MnlI	186, 131, 84, 82, 82, 52, 3	219, 211, 84, 82, 3	0.113	NLL-15	0.5, 1.6	16.0, 17.6
077J19_5	PCR	-	352	-	0.599	NLL-07	0.6, 1.9	22.0, 18.8
112B24_5	CAPS	MnlI	255, 135, 70, 48	255, 183, 70	0.669	NLL-05	1.4, 4.0	17.7, 12.7
126D14_5	CAPS	BclI	628	354, 275	0.654	NLL-06	1.3, 1.3	20.0, 19.2

LOD: logarithm of odds.

**Table 4:** Characterization of BAC clone sequences.

BAC	Length (bp)	GC (%)	Repetitive Elements (%)	Major Fractions of Repetitive Elements	BAC-FISH Signals	No. of Predicted Genes
009A01	45,596	34.3	16.4	LTR/Gypsy, NonLTR/RTE, Simple repeats	S	5
009K06	54,264	32.4	2.2	Simple repeats	S	7
042E20	35,419	33.7	25.6	LTR/Copia, DNA/Helitron, DNA/EnSpm/CACT	R	1
051F15	57,486	32.0	9.3	LTR/Copia, LTR/Gypsy, DNA/hAT	S	6
060F02	79,573	31.8	23.8	LTR/Gypsy, LTR/Copia, DNA/Helitron	S	3
070D20	46,840	33.4	7.1	LTR/Copia, Simple repeats	R	2
073E17	42,375	33.8	11.1	DNA/MuDR, LTR/Copia	S	2
075C05	94,179	35.7	33.2	LTR/Copia, LTR/Gypsy, Simple repeats	R	3
089L06 ^1^	23,289	32.3	37.3	LTR/Copia, NonLTR/L1, Simple repeats	S	0
092K09	162,642	33.4	36.2	LTR/Copia, LTR/Gypsy, DNA/EnSpm/CACTA	R	3
096G16 ^1^	4599	31.2	83.2	NonLTR/L1	S	1

^1^ partial BAC clone sequence. S, single locus BAC-FISH signal; R, repetitive (dispersed) BAC-FISH signal.

**Table 5:** Number of predicted ACCase sequences.

Species	Sequence Type	*ACC*	*accA*	*accB*	*accC*
*Acacia argyrophylla*	transcriptome	2	1	2	2
*Arachis duranensis*	genome	1	2	3	2
*Arachis ipaensis*	genome	1	2	3	2
*Arabidopsis thaliana* ^1^	genome	2	1	2	1
*Bauhinia tomentosa*	transcriptome	1	2	1	2
*Cicer arietinum*	genome	1	1	4	1
*Cajanus cajan*	genome	1	2	4	1
*Cercis canadensis*	transcriptome	1	1	2	1
*Copaifera officinalis*	transcriptome	1	2	1	1
*Chlamydomonas reinhardtii* ^2^	genome	0	1	1	1
*Glycine max*	genome	2	3	5	2
*Gompholobium polymorphum*	transcriptome	1	1	3	1
*Lupinus albus*	transcriptome	4	5	8	3
*Lupinus angustifolius*	genome	2	3	4	2
*Lotus japonicus*	genome	1	2	3	1
*Medicago truncatula*	genome	2	1	3	1
*Phaseolus vulgaris*	genome	1	1	3	1
*Vigna radiata*	genome	1	1	3	1

Non-legume species: ^1^ model plant, ^2^ model green alga.

## Supplementary Materials

The following are available online at http://www.mdpi.com/2073-4425/9/11/563/s1, Table S1: List of primers used in the study, Table S2: BAC end mapping to the *L. angustifolius* genome, Table S3: Segregation data for molecular markers developed for 83:476 × P27255 *Lupinus angustifolius* recombinant inbred line mapping population, Table S4: Copy number evaluation of *L. angustifolius* cytosolic ACCase and subunits of plastid ACCase genes by Droplet Digital PCR, Table S5: Sequence coordinates for cytosolic ACCase and subunits of plastid ACCase genes derived from legume genome assemblies, Table S6: Sequence coordinates for cytosolic ACCase and subunits of plastid ACCase genes derived from legume transcriptome assemblies, Table S7: Total score values calculated for legume *ACC* syntenic regions, Table S8: Total score values calculated for legume *accA* syntenic regions, Table S9: Total score values calculated for legume *accB* syntenic regions, Table S10: Total score values calculated for legume *accC* syntenic regions, Table S11: FASTA alignment of *ACC* sequences used for phylogenetic inference, Table S12: FASTA alignment of *accA* sequences used for phylogenetic inference, Table S13: FASTA alignment of *accB1* sequences used for phylogenetic inference, Table S14: FASTA alignment of *accB2* sequences used for phylogenetic inference, Table S15: FASTA alignment of *accC* sequences used for phylogenetic inference, Table S16: Substitutions in ACCase paralogous comparisons, Table S17: Results of branch-site model analysis of positive selection, Table S18: Gene expression values of legume cytosolic ACCase genes and subunits of plastid ACCase genes (normalized), Table S19: Gene expression values of legume cytosolic ACCase genes and subunits of plastid ACCase genes standardized to the expression of *ATPaseV0* gene, Figure S1: Structure of *ACC* genes, Figure S2: Structure of *accA* genes, Figure S3: Structure of *accB* genes, Figure S4: Structure of *accC* genes.

## References

[1] Graham P.H., Vance C.P. (2003). Legumes: importance and constraints to greater use. Plant Physiol..

[2] Anglade J., Billen G., Garnier J. (2015). Relationships for estimating N_2_ fixation in legumes: incidence for N balance of legume-based cropping systems in Europe. Ecosphere.

[3] Hughes C., Eastwood R. (2006). Island radiation on a continental scale: exceptional rates of plant diversification after uplift of the Andes. Proc. Natl. Acad. Sci. USA.

[4] Drummond C.S., Eastwood R.J., Miotto S.T.S., Hughes C.E. (2012). Multiple continental radiations and correlates of diversification in *Lupinus* (Leguminosae): Testing for key innovation with incomplete taxon sampling. Syst. Biol..

[5] Mahe F., Markova D., Pasquet R., Misset M.T., Ainouche A. (2011). Isolation, phylogeny and evolution of the *SymRK* gene in the legume genus *Lupinus* L.. Mol. Phylogen. Evol..

[6] Drummond C.S. (2008). Diversification of *Lupinus* (Leguminosae) in the western New World: Derived evolution of perennial life history and colonization of montane habitats. Mol. Phylogen. Evol..

[7] Bertioli D.J., Cannon S.B., Froenicke L., Huang G., Farmer A.D., Cannon E.K.S., Liu X., Gao D., Clevenger J., Dash S. (2016). The genome sequences of *Arachis duranensis* and *Arachis ipaensis*, the diploid ancestors of cultivated peanut. Nat. Genet..

[8] Varshney R.K., Chen W., Li Y., Bharti A.K., Saxena R.K., Schlueter J.A., Donoghue M.T.A., Azam S., Fan G., Whaley A.M. (2012). Draft genome sequence of pigeonpea (*Cajanus cajan*), an orphan legume crop of resource-poor farmers. Nat. Biotechnol..

[9] Varshney R.K., Song C., Saxena R.K., Azam S., Yu S., Sharpe A.G., Cannon S., Baek J., Rosen B.D., Tar’an B. (2013). Draft genome sequence of chickpea (*Cicer arietinum*) provides a resource for trait improvement. Nat. Biotechnol..

[10] Schmutz J., Cannon S.B., Schlueter J., Ma J., Mitros T., Nelson W., Hyten D.L., Song Q., Thelen J.J., Cheng J. (2010). Genome sequence of the palaeopolyploid soybean. Nature.

[11] Sato S., Nakamura Y., Kaneko T., Asamizu E., Kato T., Nakao M., Sasamoto S., Watanabe A., Ono A., Kawashima K. (2008). Genome structure of the legume, *Lotus japonicus*. DNA Res..

[12] Young N.D., Debellé F., Oldroyd G.E.D., Geurts R., Cannon S.B., Udvardi M.K., Benedito V.A., Mayer K.F.X., Gouzy J., Schoof H. (2011). The *Medicago* genome provides insight into the evolution of rhizobial symbioses. Nature.

[13] Schmutz J., McClean P.E., Mamidi S., Wu G.A., Cannon S.B., Grimwood J., Jenkins J., Shu S., Song Q., Chavarro C. (2014). A reference genome for common bean and genome-wide analysis of dual domestications. Nat. Genet..

[14] De Vega J.J., Ayling S., Hegarty M., Kudrna D., Goicoechea J.L., Ergon Å., Rognli O.A., Jones C., Swain M., Geurts R. (2015). Red clover (*Trifolium pratense* L.) draft genome provides a platform for trait improvement. Sci. Rep..

[15] Kang Y.J., Kim S.K., Kim M.Y., Lestari P., Kim K.H., Ha B.-K., Jun T.H., Hwang W.J., Lee T., Lee J. (2014). Genome sequence of mungbean and insights into evolution within *Vigna* species. Nat. Commun..

[16] Yang K., Tian Z., Chen C., Luo L., Zhao B., Wang Z., Yu L., Li Y., Sun Y., Li W. (2015). Genome sequencing of adzuki bean (*Vigna angularis*) provides insight into high starch and low fat accumulation and domestication. Proc. Natl. Acad. Sci. USA.

[17] Matasci N., Hung L.H., Yan Z., Carpenter E.J., Wickett N.J., Mirarab S., Nguyen N., Warnow T., Ayyampalayam S., Barker M. (2014). Data access for the 1,000 Plants (1KP) project. Gigascience.

[18] Wickett N.J., Mirarab S., Nguyen N., Warnow T., Carpenter E., Matasci N., Ayyampalayam S., Barker M.S., Burleigh J.G., Gitzendanner M.A. (2014). Phylotranscriptomic analysis of the origin and early diversification of land plants. Proc. Natl. Acad. Sci. USA.

[19] Cannon S.B., McKain M.R., Harkess A., Nelson M.N., Dash S., Deyholos M.K., Peng Y., Joyce B., Stewart C.N., Rolf M. (2015). Multiple polyploidy events in the early radiation of nodulating and nonnodulating legumes. Mol. Biol. Evol..

[20] Atkins C.A., Smith P.M.C., Gupta S., Jones M.G.K., Caligari P.D.S., Gladstones J.S., Atkins C.A., Hamblin J. (1998). Genetics, Cytology and Biotechnology. Lupins as Crop Plants: Biology, Production, and Utilization.

[21] Wendel J.F. (2000). Genome evolution in polyploids. Plant Mol. Biol..

[22] Naganowska B., Wolko B., Sliwińska E., Kaczmarek Z. (2003). Nuclear DNA content variation and species relationships in the genus *Lupinus* (Fabaceae). Ann. Bot..

[23] Hane J.K., Ming Y., Kamphuis L.G., Nelson M.N., Garg G., Atkins C.A., Bayer P.E., Bravo A., Bringans S., Cannon S. (2017). A comprehensive draft genome sequence for lupin (*Lupinus angustifolius*), an emerging health food: insights into plant-microbe interactions and legume evolution. Plant Biotechnol. J..

[24] Yang H., Tao Y., Zheng Z., Zhang Q., Zhou G., Sweetingham M.W., Howieson J.G., Li C. (2013). Draft genome sequence, and a sequence-defined genetic linkage map of the legume crop species *Lupinus angustifolius* L.. PLoS ONE.

[25] Kamphuis L.G., Hane J.K., Nelson M.N., Gao L., Atkins C.A., Singh K.B. (2015). Transcriptome sequencing of different narrow-leafed lupin tissue types provides a comprehensive uni-gene assembly and extensive gene-based molecular markers. Plant Biotechnol. J..

[26] Kroc M., Koczyk G., Święcicki W., Kilian A., Nelson M.N. (2014). New evidence of ancestral polyploidy in the Genistoid legume *Lupinus angustifolius* L. (narrow-leafed lupin). Theor. Appl. Genet..

[27] Nelson M.N., Moolhuijzen P.M., Boersma J.G., Chudy M., Lesniewska K., Bellgard M., Oliver R.P., Swiecicki W., Wolko B., Cowling W.A. (2010). Aligning a new reference genetic map of *Lupinus angustifolius* with the genome sequence of the model legume, *Lotus japonicus*. DNA Res..

[28] Nelson M.N., Phan H.T.T., Ellwood S.R., Moolhuijzen P.M., Hane J., Williams A., O’Lone C.E., Fosu-Nyarko J., Scobie M., Cakir M. (2006). The first gene-based map of *Lupinus angustifolius* L.-location of domestication genes and conserved synteny with *Medicago truncatula*. Theor. Appl. Genet..

[29] Kasprzak A., Safár J., Janda J., Dolezel J., Wolko B., Naganowska B. (2006). The bacterial artificial chromosome (BAC) library of the narrow-leafed lupin (*Lupinus angustifolius* L.). Cell. Mol. Biol. Lett..

[30] Gao L.-L., Hane J.K., Kamphuis L.G., Foley R., Shi B.-J., Atkins C.A., Singh K.B. (2011). Development of genomic resources for the narrow-leafed lupin (*Lupinus angustifolius*): Construction of a bacterial artificial chromosome (BAC) library and BAC-end sequencing. BMC Genom..

[31] Książkiewicz M., Wyrwa K., Szczepaniak A., Rychel S., Majcherkiewicz K., Przysiecka Ł., Karlowski W., Wolko B., Naganowska B. (2013). Comparative genomics of *Lupinus angustifolius* gene-rich regions: BAC library exploration, genetic mapping and cytogenetics. BMC Genom..

[32] Książkiewicz M., Zielezinski A., Wyrwa K., Szczepaniak A., Rychel S., Karlowski W., Wolko B., Naganowska B. (2015). Remnants of the legume ancestral genome preserved in gene-rich regions: Insights from *Lupinus angustifolius* physical, genetic, and comparative mapping. Plant Mol. Biol. Rep..

[33] Wyrwa K., Książkiewicz M., Szczepaniak A., Susek K., Podkowiński J., Naganowska B. (2016). Integration of *Lupinus angustifolius* L. (narrow-leafed lupin) genome maps and comparative mapping within legumes. Chromosome Res..

[34] Leśniewska K., Książkiewicz M., Nelson M.N., Mahé F., Aïnouche A., Wolko B., Naganowska B. (2011). Assignment of 3 genetic linkage groups to 3 chromosomes of narrow-leafed lupin. J. Hered..

[35] Przysiecka Ł., Książkiewicz M., Wolko B., Naganowska B. (2015). Structure, expression profile and phylogenetic inference of chalcone isomerase-like genes from the narrow-leafed lupin (*Lupinus angustifolius* L.) genome. Front. Plant Sci..

[36] Książkiewicz M., Rychel S., Nelson M.N., Wyrwa K., Naganowska B., Wolko B. (2016). Expansion of the phosphatidylethanolamine binding protein family in legumes: A case study of *Lupinus angustifolius* L. *FLOWERING LOCUS T* homologs, *LanFTc1* and *LanFTc2*. BMC Genom..

[37] Narożna D., Książkiewicz M., Przysiecka Ł., Króliczak J., Wolko B., Naganowska B., Mądrzak C.J. (2017). Legume isoflavone synthase genes have evolved by whole-genome and local duplications yielding transcriptionally active paralogs. Plant Sci..

[38] Nikolau B.J., Ohlrogge J.B., Wurtele E.S. (2003). Plant biotin-containing carboxylases. Arch. Biochem. Biophys..

[39] Tong L. (2013). Structure and function of biotin-dependent carboxylases. Cell. Mol. Life Sci..

[40] Huerlimann R., Heimann K. (2013). Comprehensive guide to acetyl-carboxylases in algae. Crit. Rev. Biotechnol..

[41] Schwender J., Ohlrogge J.B. (2002). Probing *in vivo* metabolism by stable isotope labeling of storage lipids and proteins in developing *Brassica napus* embryos. Plant Physiol..

[42] Sasaki Y., Konishi T., Nagano Y. (1995). The compartmentation of acetyl-coenzyme A carboxylase in plants. Plant Physiol..

[43] Deusch O., Landan G., Roettger M., Gruenheit N., Kowallik K.V., Allen J.F., Martin W., Dagan T. (2008). Genes of cyanobacterial origin in plant nuclear genomes point to a heterocyst-forming plastid ancestor. Mol. Biol. Evol..

[44] Lombard J., Moreira D. (2011). Early evolution of the biotin-dependent carboxylase family. BMC Evol. Biol..

[45] Konishi T., Sasaki Y. (1994). Compartmentalization of two forms of acetyl-CoA carboxylase in plants and the origin of their tolerance toward herbicides. Proc. Natl. Acad. Sci. USA.

[46] Sasaki Y., Nagano Y. (2004). Plant acetyl-CoA carboxylase: structure, biosynthesis, regulation, and gene manipulation for plant breeding. Biosci. Biotechnol. Biochem..

[47] Salie M.J., Thelen J.J. (2016). Regulation and structure of the heteromeric acetyl-CoA carboxylase. Biochim. Biophys. Acta.

[48] Podkowinski J., Jelenska J., Sirikhachornkit A., Zuther E., Haselkorn R., Gornicki P. (2003). Expression of cytosolic and plastid acetyl-coenzyme A carboxylase genes in young wheat plants. Plant Physiol..

[49] Shirley B.W. (1996). Flavonoid biosynthesis: ‘New’ functions for an ‘old’ pathway. Trends Plant Sci..

[50] Cronan J.E., Waldrop G.L. (2002). Multi-subunit acetyl-CoA carboxylases. Prog. Lipid Res..

[51] Reverdatto S., Beilinson V., Nielsen N.C. (1999). A multisubunit acetyl coenzyme A carboxylase from soybean. Plant Physiol..

[52] Chen Y., Elizondo-Noriega A., Cantu D.C., Reilly P.J. (2012). Structural classification of biotin carboxyl carrier proteins. Biotechnol. Lett..

[53] Gornicki P., Podkowinski J., Scappino L.A., DiMaio J., Ward E., Haselkorn R. (1994). Wheat acetyl-coenzyme A carboxylase: cDNA and protein structure. Proc. Natl. Acad. Sci. USA.

[54] Podkowinski J., Sroga G.E., Haselkorn R., Gornicki P. (1996). Structure of a gene encoding a cytosolic acetyl-CoA carboxylase of hexaploid wheat. Proc. Natl. Acad. Sci. USA.

[55] Roesler K.R., Savage L.J., Shintani D.K., Shorrosh B.S., Ohlrogge J.B. (1996). Co-purification, co-immunoprecipitation, and coordinate expression of acetyl-coenzyme A carboxylase activity, biotin carboxylase, and biotin carboxyl carrier protein of higher plants. Planta.

[56] Shorrosh B.S., Dixon R.A., Ohlrogge J.B. (1994). Molecular cloning, characterization, and elicitation of acetyl-CoA carboxylase from alfalfa. Proc. Natl. Acad. Sci. USA.

[57] Magadum S., Banerjee U., Murugan P., Gangapur D., Ravikesavan R. (2013). Gene duplication as a major force in evolution. J. Genet..

[58] Boersma J.G., Pallotta M., Li C., Buirchell B.J., Sivasithamparam K., Yang H. (2005). Construction of a genetic linkage map using MFLP and identification of molecular markers linked to domestication genes in narrow-leafed lupin (*Lupinus angustifolius* L.). Cell. Mol. Biol. Lett..

[59] Altschul S.F., Gish W., Miller W., Myers E.W., Lipman D.J. (1990). Basic local alignment search tool. J. Mol. Biol..

[60] Kearse M., Moir R., Wilson A., Stones-Havas S., Cheung M., Sturrock S., Buxton S., Cooper A., Markowitz S., Duran C. (2012). Geneious Basic: an integrated and extendable desktop software platform for the organization and analysis of sequence data. Bioinformatics.

[61] Yandell M., Ence D. (2012). A beginner’s guide to eukaryotic genome annotation. Nat. Rev. Genet..

[62] Ekblom R., Wolf J.B.W. (2014). A field guide to whole-genome sequencing, assembly and annotation. Evol. Appl..

[63] Kohany O., Gentles A.J., Hankus L., Jurka J. (2006). Annotation, submission and screening of repetitive elements in Repbase: RepbaseSubmitter and Censor. BMC Bioinform..

[64] Solovyev V. (2004). Statistical approaches in eukaryotic gene prediction. Handbook of Statistical Genetics.

[65] Neff M.M., Turk E., Kalishman M. (2002). Web-based primer design for single nucleotide polymorphism analysis. Trends Genet..

[66] Manly K.F., Robert H., Cudmore J., Meer J.M. (2001). Map Manager QTX, cross-platform software for genetic mapping. Mamm. Genome.

[67] Voorrips R.E. (2002). MapChart: software for the graphical presentation of linkage maps and QTLs. J. Hered..

[68] Arabidopsis Genome Initiative (2000). Analysis of the genome sequence of the flowering plant *Arabidopsis thaliana*. Nature.

[69] Parra-González L.B., Aravena-Abarzúa G.A., Navarro-Navarro C.S., Udall J., Maughan J., Peterson L.M., Salvo-Garrido H.E., Maureira-Butler I.J. (2012). Yellow lupin (*Lupinus luteus* L.) transcriptome sequencing: molecular marker development and comparative studies. BMC Genom..

[70] O’Rourke J.A., Yang S.S., Miller S.S., Bucciarelli B., Liu J., Rydeen A., Bozsoki Z., Uhde-Stone C., Tu Z.J., Allan D. (2013). An RNA-Seq transcriptome analysis of orthophosphate-deficient white lupin reveals novel insights into phosphorus acclimation in plants. Plant Physiol..

[71] Hu B., Jin J., Guo A.-Y., Zhang H., Luo J., Gao G. (2015). GSDS 2.0: An upgraded gene feature visualization server. Bioinformatics.

[72] Revanna K.V., Chiu C.-C., Bierschank E., Dong Q. (2011). GSV: A web-based genome synteny viewer for customized data. BMC Bioinform..

[73] Krzywinski M., Schein J., Birol I., Connors J., Gascoyne R., Horsman D., Jones S.J., Marra M.A. (2009). Circos: An information aesthetic for comparative genomics. Genome Res..

[74] Katoh K., Misawa K., Kuma K.-i., Miyata T. (2002). MAFFT: A novel method for rapid multiple sequence alignment based on fast Fourier transform. Nucleic Acids Res..

[75] Darriba D., Taboada G.L., Doallo R., Posada D. (2012). jModelTest 2: More models, new heuristics and parallel computing. Nat. Methods.

[76] Huelsenbeck J.P., Ronquist F. (2001). MRBAYES: Bayesian inference of phylogenetic trees. Bioinformatics.

[77] Le S.Q., Gascuel O. (2008). An improved general amino acid replacement matrix. Mol. Biol. Evol..

[78] Jones D.T., Taylor W.R., Thornton J.M. (1992). The rapid generation of mutation data matrices from protein sequences. Comput. Appl. Biosci..

[79] Librado P., Rozas J. (2009). DnaSP v5: A software for comprehensive analysis of DNA polymorphism data. Bioinformatics.

[80] Yang Z. (2007). PAML 4: Phylogenetic analysis by maximum likelihood. Mol. Biol. Evol..

[81] Clevenger J., Chu Y., Scheffler B., Ozias-Akins P. (2016). A developmental transcriptome map for allotetraploid *Arachis hypogaea*. Front. Plant Sci..

[82] Pazhamala L.T., Purohit S., Saxena R.K., Garg V., Krishnamurthy L., Verdier J., Varshney R.K. (2017). Gene expression atlas of pigeonpea and its application to gain insights into genes associated with pollen fertility implicated in seed formation. J. Exp. Bot..

[83] Li J., Dai X., Liu T., Zhao P.X. (2012). LegumeIP: An integrative database for comparative genomics and transcriptomics of model legumes. Nucleic Acids Res..

[84] Severin A.J., Woody J.L., Bolon Y.T., Joseph B., Diers B.W., Farmer A.D., Muehlbauer G.J., Nelson R.T., Grant D., Specht J.E. (2010). RNA-Seq Atlas of *Glycine max*: A guide to the soybean transcriptome. BMC Plant Biol..

[85] Verdier J., Torres-Jerez I., Wang M., Andriankaja A., Allen S.N., He J., Tang Y., Murray J.D., Udvardi M.K. (2013). Establishment of the *Lotus japonicus* Gene Expression Atlas (LjGEA) and its use to explore legume seed maturation. Plant J..

[86] Benedito V.A., Torres-Jerez I., Murray J.D., Andriankaja A., Allen S., Kakar K., Wandrey M., Verdier J., Zuber H., Ott T. (2008). A gene expression atlas of the model legume *Medicago truncatula*. Plant J..

[87] He J., Benedito V.A., Wang M., Murray J.D., Zhao P.X., Tang Y., Udvardi M.K. (2009). The *Medicago truncatula* gene expression atlas web server. BMC Bioinform..

[88] O’Rourke J.A., Iniguez L.P., Fu F., Bucciarelli B., Miller S.S., Jackson S.A., McClean P.E., Li J., Dai X., Zhao P.X. (2014). An RNA-Seq based gene expression atlas of the common bean. BMC Genom..

[89] Yao S., Jiang C., Huang Z., Torres-Jerez I., Chang J., Zhang H., Udvardi M., Liu R., Verdier J. (2016). The *Vigna unguiculata* Gene Expression Atlas (VuGEA) from de novo assembly and quantification of RNA-seq data provides insights into seed maturation mechanisms. Plant J..

[90] Nelson M.N., Książkiewicz M., Rychel S., Besharat N., Taylor C.M., Wyrwa K., Jost R., Erskine W., Cowling W.A., Berger J.D. (2017). The loss of vernalization requirement in narrow-leafed lupin is associated with a deletion in the promoter and de-repressed expression of a *Flowering Locus T* (*FT*) homologue. New Phytol..

[91] Zou C., Lehti-Shiu M.D., Thibaud-Nissen F., Prakash T., Buell C.R., Shiu S.-H. (2009). Evolutionary and expression signatures of pseudogenes in *Arabidopsis* and rice. Plant Physiol..

[92] Visendi P., Berkman P.J., Hayashi S., Golicz A.A., Bayer P.E., Ruperao P., Hurgobin B., Montenegro J., Chan C.-K.K., Staňková H. (2016). An efficient approach to BAC based assembly of complex genomes. Plant Methods.

[93] Varshney R.K., Shi C., Thudi M., Mariac C., Wallace J., Qi P., Zhang H., Zhao Y., Wang X., Rathore A. (2017). Pearl millet genome sequence provides a resource to improve agronomic traits in arid environments. Nat. Biotechnol..

[94] Chen Y., Zhang Q., Hu W., Zhang X., Wang L., Hua X., Yu Q., Ming R., Zhang J. (2017). Evolution and expression of the fructokinase gene family in Saccharum. BMC Genom..

[95] Rispail N., Rubiales D. (2016). Genome-wide identification and comparison of legume MLO gene family. Sci. Rep..

[96] Zheng F., Wu H., Zhang R., Li S., He W., Wong F.-L., Li G., Zhao S., Lam H.-M. (2016). Molecular phylogeny and dynamic evolution of disease resistance genes in the legume family. BMC Genom..

[97] Cui L., Wall P.K., Leebens-Mack J.H., Lindsay B.G., Soltis D.E., Doyle J.J., Soltis P.S., Carlson J.E., Arumuganathan K., Barakat A. (2006). Widespread genome duplications throughout the history of flowering plants. Genome Res..

[98] Jiao Y., Wickett N.J., Ayyampalayam S., Chanderbali A.S., Landherr L., Ralph P.E., Tomsho L.P., Hu Y., Liang H., Soltis P.S. (2011). Ancestral polyploidy in seed plants and angiosperms. Nature.

[99] Jiao Y., Leebens-Mack J., Ayyampalayam S., Bowers J.E., McKain M.R., McNeal J., Rolf M., Ruzicka D.R., Wafula E., Wickett N.J. (2012). A genome triplication associated with early diversification of the core eudicots. Genome Biol..

[100] Van de Peer Y. (2011). A mystery unveiled. Genome Biol..

[101] Schlueter J.A., Dixon P., Granger C., Grant D., Clark L., Doyle J.J., Shoemaker R.C. (2004). Mining EST databases to resolve evolutionary events in major crop species. Genome.

[102] Cannon S.B., Ilut D., Farmer A.D., Maki S.L., May G.D., Singer S.R., Doyle J.J. (2010). Polyploidy did not predate the evolution of nodulation in all legumes. PLoS ONE.

[103] Lavin M., Herendeen P.S., Wojciechowski M.F. (2005). Evolutionary rates analysis of Leguminosae implicates a rapid diversification of lineages during the tertiary. Syst. Biol..

[104] Pfeil B.E., Schlueter J.A., Shoemaker R.C., Doyle J.J. (2005). Placing paleopolyploidy in relation to taxon divergence: a phylogenetic analysis in legumes using 39 gene families. Syst. Biol..

[105] Bertioli D.J., Moretzsohn M.C., Madsen L.H., Sandal N., Leal-Bertioli S.C.M., Guimarães P.M., Hougaard B.K., Fredslund J., Schauser L., Nielsen A.M. (2009). An analysis of synteny of *Arachis* with *Lotus* and *Medicago* sheds new light on the structure, stability and evolution of legume genomes. BMC Genom..

[106] Cannon S.B., Sterck L., Rombauts S., Sato S., Cheung F., Gouzy J., Wang X., Mudge J., Vasdewani J., Schiex T. (2006). Legume genome evolution viewed through the *Medicago truncatula* and *Lotus japonicus* genomes. Proc. Natl. Acad. Sci. USA.

[107] Wang Z., Zhou Z., Liu Y., Liu T., Li Q., Ji Y., Li C., Fang C., Wang M., Wu M. (2015). Functional evolution of phosphatidylethanolamine binding proteins in soybean and *Arabidopsis*. Plant Cell.

[108] Lu S., Zhao H., Parsons E.P., Xu C., Kosma D.K., Xu X., Chao D., Lohrey G., Bangarusamy D.K., Wang G. (2011). The *glossyhead1* allele of *ACC1* reveals a principal role for multidomain acetyl-coenzyme A carboxylase in the biosynthesis of cuticular waxes by *Arabidopsis*. Plant Physiol..

[109] Post-Beittenmiller D. (1996). Biochemistry and molecular biology of wax production in plants. Annu. Rev. Plant Physiol. Plant Mol. Biol..

[110] Caffrey J.J., Choi J.-K., Wurtele E.S., Nikolau B.J. (1998). Tissue distribution of acetyl-CoA carboxylase in leaves of leek (*Allium porrum* L.). J. Plant Physiol..

[111] Garcia-Ponce B., Rocha-Sosa M. (2000). The octadecanoid pathway is required for pathogen-induced multi-functional acetyl-CoA carboxylase accumulation in common bean (*Phaseolus vulgaris* L.). Plant Sci..

[112] Baud S., Guyon V., Kronenberger J., Wuilleme S., Miquel M., Caboche M., Lepiniec L., Rochat C. (2003). Multifunctional acetyl-CoA carboxylase 1 is essential for very long chain fatty acid elongation and embryo development in *Arabidopsis*. Plant J..

[113] Ke J., Wen T.N., Nikolau B.J., Wurtele E.S. (2000). Coordinate regulation of the nuclear and plastidic genes coding for the subunits of the heteromeric acetyl-coenzyme A carboxylase. Plant Physiol..

[114] Wan H., Cui Y., Ding Y., Mei J., Dong H., Zhang W., Wu S., Liang Y., Zhang C., Li J. (2016). Time-series analyses of transcriptomes and proteomes reveal molecular networks underlying oil accumulation in canola. Front. Plant Sci..

[115] Wang J., Singh S.K., Du C., Li C., Fan J., Pattanaik S., Yuan L. (2016). Comparative transcriptomic analysis of two *Brassica napus* near-isogenic lines reveals a network of genes that influences seed oil accumulation. Front. Plant Sci..

[116] Chen M., Mooney B.P., Hajduch M., Joshi T., Zhou M., Xu D., Thelen J.J. (2009). System analysis of an *Arabidopsis* mutant altered in *de novo* fatty acid synthesis reveals diverse changes in seed composition and metabolism. Plant Physiol..

[117] Salie M.J., Zhang N., Lancikova V., Xu D., Thelen J.J. (2016). A family of negative regulators targets the committed step of *de novo* fatty acid biosynthesis. Plant Cell.

[118] Feria Bourrellier A.B., Valot B., Guillot A., Ambard-Bretteville F., Vidal J., Hodges M. (2010). Chloroplast acetyl-CoA carboxylase activity is 2-oxoglutarate-regulated by interaction of PII with the biotin carboxyl carrier subunit. Proc. Natl. Acad. Sci. USA.

[119] Focks N., Benning C. (1998). wrinkled1: A novel, low-seed-oil mutant of *Arabidopsis* with a deficiency in the seed-specific regulation of carbohydrate metabolism. Plant Physiol..

[120] Bogdanova V.S., Zaytseva O.O., Mglinets A.V., Shatskaya N.V., Kosterin O.E., Vasiliev G.V. (2015). Nuclear-cytoplasmic conflict in pea (*Pisum sativum* L.) is associated with nuclear and plastidic candidate genes encoding acetyl-CoA carboxylase subunits. PLoS ONE.

[121] Li X., Ilarslan H., Brachova L., Qian H.R., Li L., Che P., Wurtele E.S., Nikolau B.J. (2011). Reverse-genetic analysis of the two biotin-containing subunit genes of the heteromeric acetyl-coenzyme A carboxylase in *Arabidopsis* indicates a unidirectional functional redundancy. Plant Physiol..

[122] Roesler K., Shintani D., Savage L., Boddupalli S., Ohlrogge J. (1997). Targeting of the *Arabidopsis* homomeric acetyl-coenzyme A carboxylase to plastids of rapeseeds. Plant Physiol..

[123] Gu K., Chiam H., Tian D., Yin Z. (2011). Molecular cloning and expression of heteromeric ACCase subunit genes from *Jatropha curcas*. Plant Sci..

[124] Harwood J.L. (1988). Fatty acid metabolism. Annu. Rev. Plant Physiol. Plant Mol. Biol..

[125] Cork J.M., Purugganan M.D. (2004). The evolution of molecular genetic pathways and networks. Bioessays.

[126] Ramsay H., Rieseberg L.H., Ritland K. (2009). The correlation of evolutionary rate with pathway position in plant terpenoid biosynthesis. Mol. Biol. Evol..

[127] Clotault J., Peltier D., Soufflet-Freslon V., Briard M., Geoffriau E. (2012). Differential selection on carotenoid biosynthesis genes as a function of gene position in the metabolic pathway: A study on the carrot and dicots. PLoS ONE.

[128] Olson-Manning C.F., Lee C.R., Rausher M.D., Mitchell-Olds T. (2013). Evolution of flux control in the glucosinolate pathway in *Arabidopsis thaliana*. Mol. Biol. Evol..

[129] Rausher M.D., Miller R.E., Tiffin P. (1999). Patterns of evolutionary rate variation among genes of the anthocyanin biosynthetic pathway. Mol. Biol. Evol..

