# Supplementary material for: Legume Cytosolic and Plastid Acetyl-Coenzyme—A Carboxylase Genes Differ by Evolutionary Patterns and Selection Pressure Schemes Acting before and after Whole-Genome Duplications
Source: Genes (Basel). 2018 Nov 21;9(11):563. doi: 10.3390/genes9110563 (PMC6265850; doi:10.3390/genes9110563)
Supplement: Supplementary file 1 [file genes-09-00563-s001.zip › Supplementary-fig-final.docx]

Supplementary Figures

Legume cytosolic and plastid acetyl-coenzyme A carboxylase genes differ by evolutionary patterns and selection pressure schemes acting before and after whole-genome duplications

Anna Szczepaniak, Michał Książkiewicz, Jan Podkowiński, Katarzyna B. Czyż, Marek Figlerowicz, Barbara Naganowska


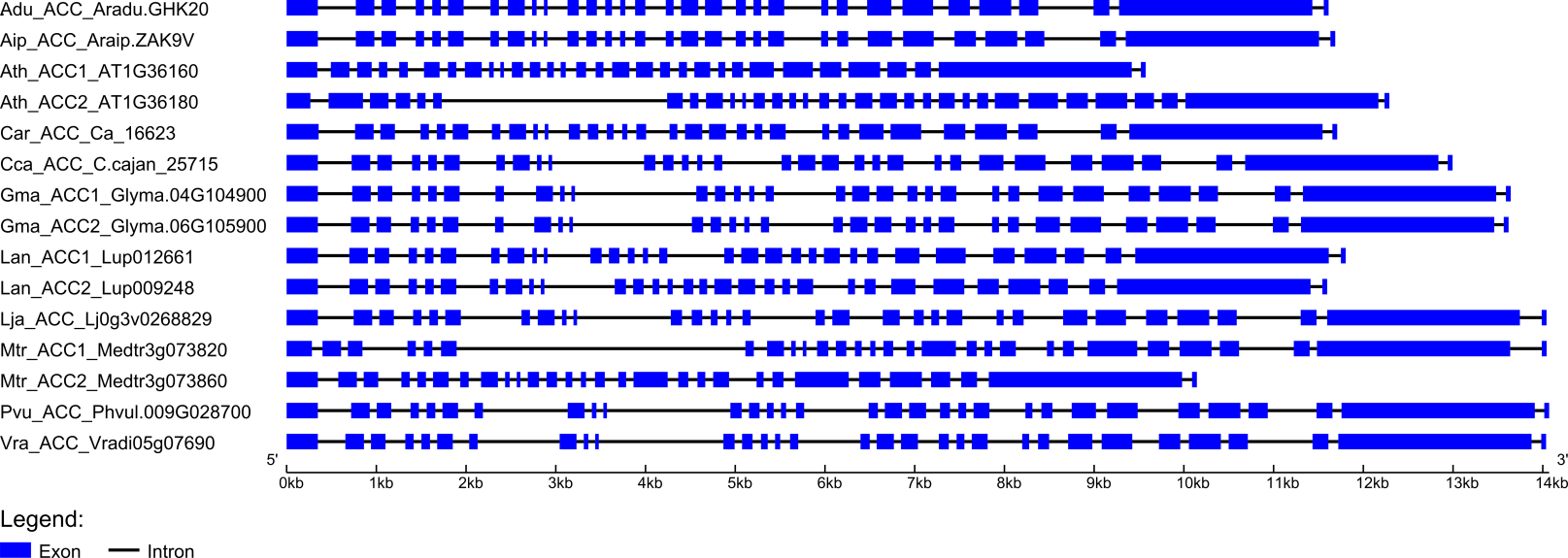


**Figure S1.** Structure of *ACC* genes


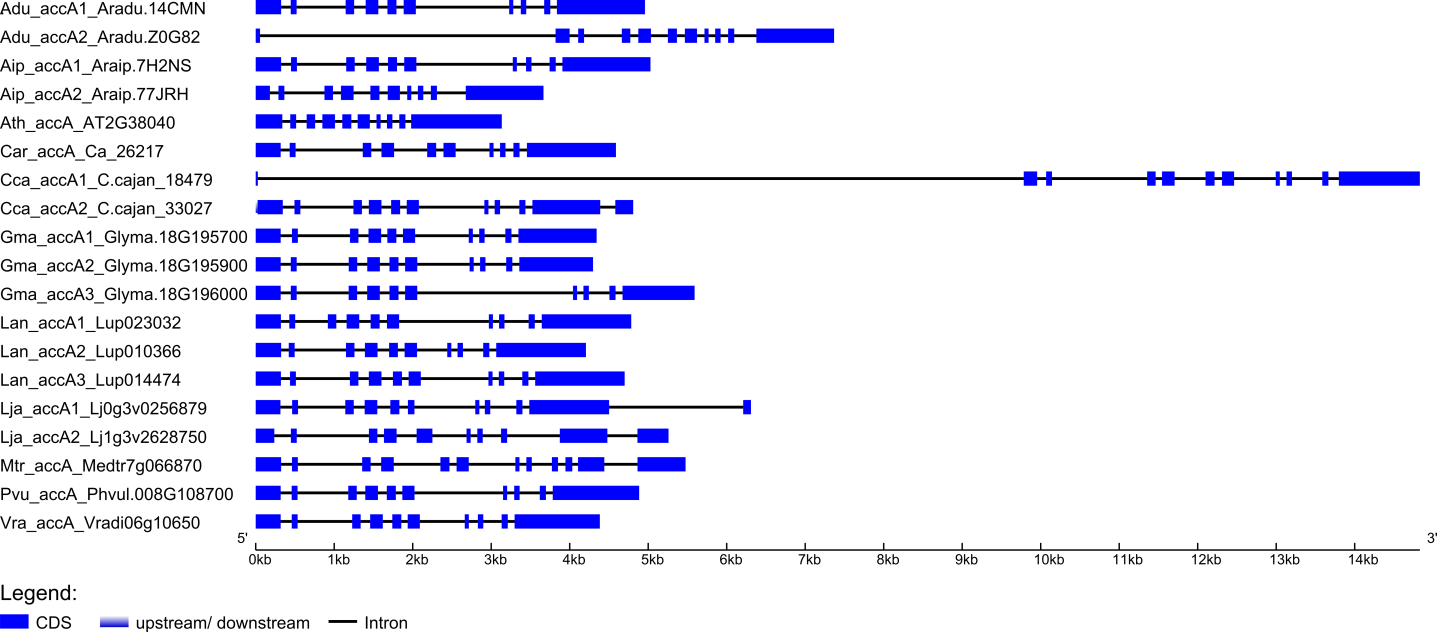


**Figure S2.** Structure of *accA* genes


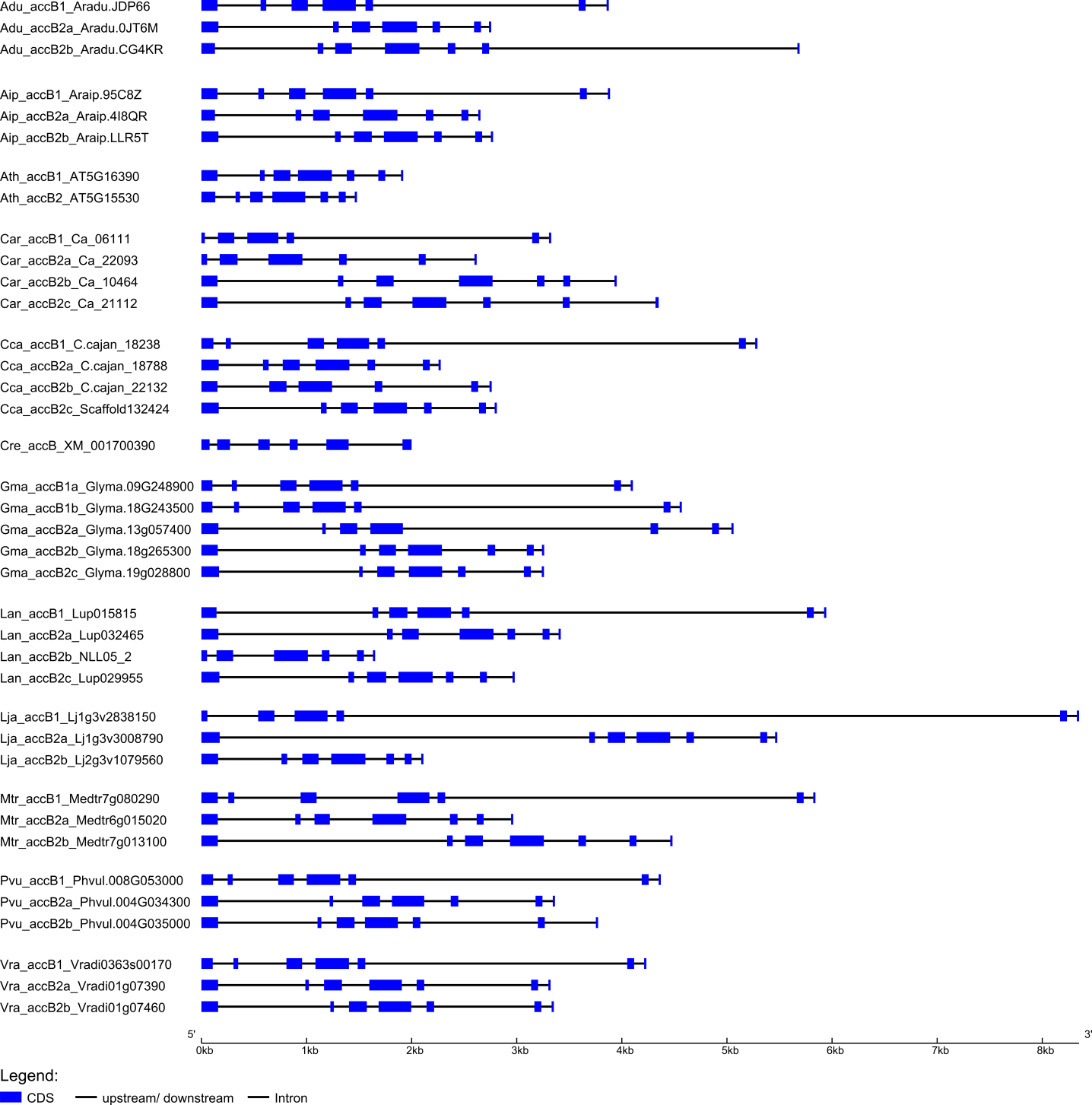


**Figure S3.** Structure of *accB* genes


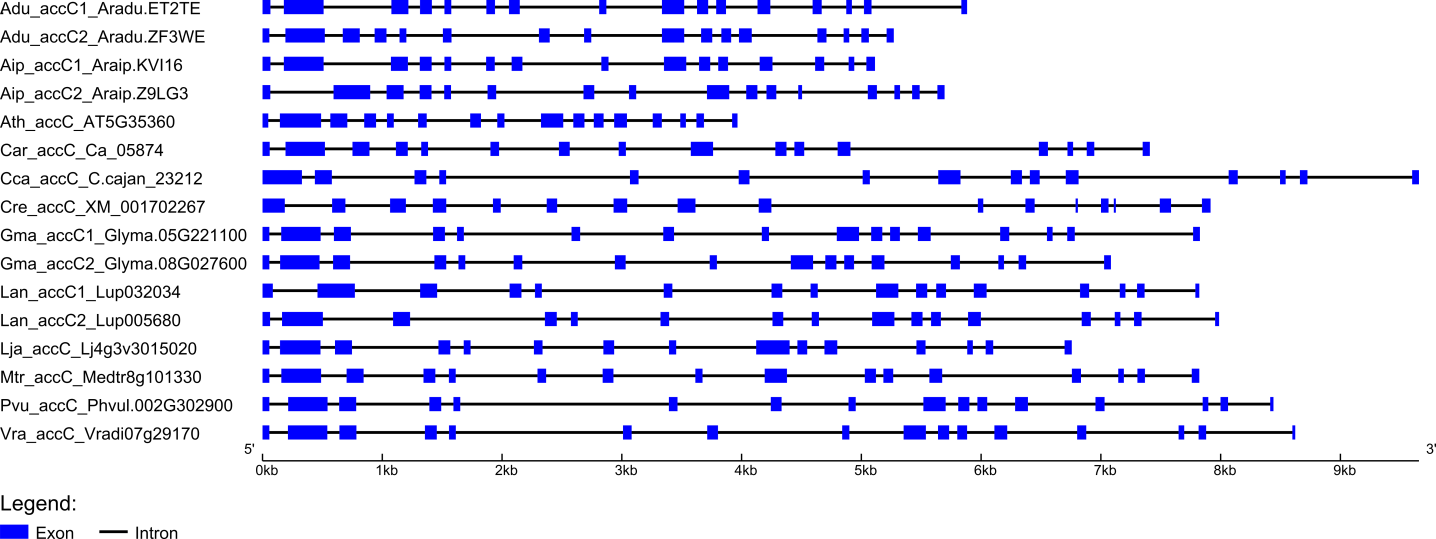


**Figure S4.** Structure of *accC* genes
