# Supplementary material for: Legume Cytosolic and Plastid Acetyl-Coenzyme—A Carboxylase Genes Differ by Evolutionary Patterns and Selection Pressure Schemes Acting before and after Whole-Genome Duplications
Source: Genes (Basel). 2018 Nov 21;9(11):563. doi: 10.3390/genes9110563 (PMC6265850; doi:10.3390/genes9110563)
Supplement: Supplementary file 1 [file genes-09-00563-s001.zip › Supplementary-tab-final.docx]

Supplementary Tables

Legume cytosolic and plastid acetyl-coenzyme A carboxylase genes differ by evolutionary patterns and selection pressure schemes acting before and after whole-genome duplications

Anna Szczepaniak, Michał Książkiewicz, Jan Podkowiński, Katarzyna B. Czyż, Marek Figlerowicz, Barbara Naganowska

# Table S1. List of primers used in the study

| **Primer name** | **Forward primer** | **Reverse primer** | **Task** |
| --- | --- | --- | --- |
| Pri-129, Pri-131 | TGACAARGACAGCTTTGTGGAGACA | CWCGGAGTTCRCCCATCATTGG | Probe amplification |
| Pri-139, Pri-140 | CATTGTRGARTTGMAGHTGAAGCAG | CACCMGGWSCAGGACTKCGGTA | Probe amplification |
| Pri-142, Pri-145 | GAGGTTGATCACGAATWBTYTRAGGC | AAGCCAACCTCNTHWBTAAACTCAATGAT | Probe amplification |
| J08-35, J08-36 | TACAATGTTGCATCCTGGATATGGT | ATGATAATCAATTGTTGTAGGAACTC | Probe amplification |
| BAC3, BAC5 | GGATGTGCTGCAAGGCGATTAAGTTGG | CTCGTATGTTGTGTGGAATTGTGAGC | BAC-end sequencing |
| ddAAT-P2 | CCCAGCTCTCTTCGATGAAT | CAATCCTTTCCACTCTTGTCTTTAG | ddPCR |
| ddACC | ACCTTGTGAGTGCTCCTTTG | AGAGCCTGCGTACATAAGTTTC | ddPCR |
| ddBC | AGAGTTGCGGAAGGCAAT | CCCTTTCATCCAATAGAAACTCAAC | ddPCR |
| ddBCCP | GTAGAAACACTGGGTACAAAGGA | ACAAGACTTGCGACTTGACTAA | ddPCR |
| ddCT-α | ACTCCATGGTGATCGGGAA | GGAGTTGGCATCCCAAAGTTA | ddPCR |
| 002e06_3 | CAATCACCTTTCACAATCCTC | ATCTCTATTTCCCCTAAAATGATG | Linkage mapping |
| 002e06_3_2 | CCATATTGGCTTAGCATTAGTATTC | ATTGAAGAGCAGGGTTGATGAC | Linkage mapping |
| 002e06_5 | ATGGAACAACATCTCAATAATAGGA | CGATTAAATGACAGCAGATAGCC | Linkage mapping |
| 002f03_3 | AGCTTCTTCTCCTAAATACATTTC | TTCTTTTATGTTGGAGATGATGGA | Linkage mapping |
| 002f03_5 | ATTCGTCTACTAATTTTATCCTTCG | TCTGATGTAAACTTGATATCTCTGC | Linkage mapping |
| 005g02_3 | AGCTTGAGGAGATTTGATGAATG | GAGGTGTAATTTGGTGGTTAGACA | Linkage mapping |
| 005g02_3_2 | TGGGGCTATTCCCTTTTATATTC | TATTGAGGGCAACCTAGTCAAA | Linkage mapping |
| 005g02_5 | GCTTAACTGTGACTGTGGAGTTG | TATCACTTTCGACCAAATAGGG | Linkage mapping |
| 009a01_3 | GCATATTGCTGGTAATCAGACTCA | TTTGCAGCATGTAAAGCAATTT | Linkage mapping |
| 009a01_3_2 | TTCCTCTACAGTTCTGATCTTTCTGTT |  | Linkage mapping |
| 009a01_5 | AAGCTTGTCATGCCTAAAATAACA | CAATTAGGCACAAGTTACAGATACA | Linkage mapping |
| 009k06_3 | AAAAAGAAGCTGCCTTTGGAGT | GGGGGAAATTAAGGTAGAGAGG | Linkage mapping |
| 009k06_5 | TGAATCTTGCCATTTTGTGA | TATGCATTCCATGCCTATTT | Linkage mapping |
| 011g20_3 | CCTTGAGATGAATGGATTTGCT | CAACCTCTAATACCTGGTTGGTT | Linkage mapping |
| 011g20_5 | GTCATACACAAGCTTGGCACAT | CAAAGCCGCTATAATGGTTCA | Linkage mapping |
| 016j11_3 | ATATTAATCAGGTTTGTAGCTTG | TTGGAGTAATTTTTGGGGCT | Linkage mapping |
| 016j11_3_2 | ACGTGTTCTTTTGTTTCTTTTTCACAC |  | Linkage mapping |
| 016j11_5 | GCTTGAAAGCATACATAAATCCTC | ACCACCATCACCTTCCTCCAATA | Linkage mapping |
| 034g21_3 | CTTCCAATTTTTCTTTGCCTTT | GACTGTAATGGCTGTAACCAACC | Linkage mapping |
| 034g21_5 | ATGCCCAAGCTACTGAACAAG | ATATTTCTTTTGGGCCGCTGA | Linkage mapping |
| 034g21_5_2 | AAGGAGGTGACAAAGCTACTTGA |  | Linkage mapping |
| 040m06_3 | AAGCTTTGAAATGAGGAACTTC | GTGGATAAAAGCATGAAAAACAA | Linkage mapping |
| 040m06_5 | ATGTGTTGATGGAGCCAACT | CCTCTTATGCCATATCTAAGTGT | Linkage mapping |
| 042c13_3 | CATCCACTAATATGGCCACAC | TTGTTTCATACAAATTCCTAAC | Linkage mapping |
| 042c13_5 | AACACCAACATGAATTTCTTTC | TGGATTTCCATTTATCAGATT | Linkage mapping |
| 042c13_5_2 | ACAGAAATTCCAATCAAACAACA |  | Linkage mapping |
| 042e20_3 | CTTGGATTCACCAGTTCCA | GTTGTTCAATTTGCAAGAAGCA | Linkage mapping |
| 042e20_5 | CATTTGAAGCATGCTATGGC | TTTGGTATATGATCCAACCCA | Linkage mapping |
| 042e20_5_2 | CAAACATTTGGGGGTAAATTTCTATG | GCACCTGCATACCAAGAAAGAA | Linkage mapping |
| 046i04_3 | CAAAGACAGATTGGTCGAAGA | AGATTCATGGGCTTTTTCTT | Linkage mapping |
| 046i04_3_2 | ATCACATAACAAACCCTGGAAATAAA | GTTCAGTGTGGTGGTCAGGTT | Linkage mapping |
| 046i04_5 | ATATAACAAAATAAACTCCCACCAC | TAAATAAGTGCACTCTAAAGAGGA | Linkage mapping |
| 048n08_3 | ATCCTCTCAAACAATGGTGGAC | GTTCCACCCTTTAACCCCAT | Linkage mapping |
| 048n08_5 | AAGCTAAGGAATAAATCAATGGTG | GCTCAACTTGGTATTCTTTGTT | Linkage mapping |
| 049f04_3 | TTTCTTTCAAATCACTAATGTG | TTTTCAGGTCTCTTTTCTTCT | Linkage mapping |
| 049f04_3_2 | CTGGCTATTTTGGTCAACTAATTTT | TCTACTGACAAAGCAAGCAGAAA | Linkage mapping |
| 049f04_5 | AAGCCAAGTTTAGTCCCAGTCA | CAAACATTCCTCCCATTCAC | Linkage mapping |
| 051f15_3 | TGTATGAAAAATGAATAGACCGACA | AAGTCAACATAATTTGAAGATCG | Linkage mapping |
| 051f15_5 | GCATATCAGGTGCATCAAAGT | AAAGGACACTAAACCCGTGGAT | Linkage mapping |
| 060f02_3 | TTAAACCTCCTAACCAAATCACG | TGGACTGAGATAACATGTCTCTTG | Linkage mapping |
| 060f02_5 | AGCTTTAAAGTGTAGTGAAAATTCC | TCCAAAACTCATGGGTAGAGAAA | Linkage mapping |
| 066e06_3 | AGCAAACATTCCTGCTCAATTA | GAGATGCAAAGAGAAGTGATGG | Linkage mapping |
| 066e06_5 | GTAGTCTCGCTTAGCTGGTTGG | TGCATCTGCTCATCAAATACATC | Linkage mapping |
| 066e06_5_2 | TCGCTTATGTGAAAAGTTCTGTTTGT |  | Linkage mapping |
| 069l17_3 | AAGCTTTCTTTAGGCTTCTTGGA | TGGAACATAACATAGACCAACAA | Linkage mapping |
| 069l17_5 | TGAGTCCTGGTTTCATTTGTTG | CGCCATTTGACAATACTGTTCC | Linkage mapping |
| 069l17_5_2 | TGCCACCAGTTAAAATTGTCTGT |  | Linkage mapping |
| 070d20_3 | AAGCTTGAATCTGCATTTGG | GGGTGTTGAATGTTTCAATTTT | Linkage mapping |
| 070d20_3_2 | CATTCAGCACTCTTATGCTTCTCT | CATATACAATGCGGATTAAGAGTT | Linkage mapping |
| 070d20_5 | CAATGTGTTGATGGAGCCAACT | GTGCTTTTGTGGGTGGCAAT | Linkage mapping |
| 070d20_5_2 | CAGTGACCAAAAGTATCTTCAATC | TATTTGCCCTTTCTTTTGTTCC | Linkage mapping |
| 073e17_3 | TTTCTCAAAAACACTATTGGTCCAT | TCATATTTTCGTGTTTAGTCACTCT | Linkage mapping |
| 073e17_5 | AGCTTAATCAACAAAGACTTCCGTA | AGTTCCCGATGTACAACAAACA | Linkage mapping |
| 075c05_3 | AAGCTTTCTTTCTTCAAGCCTAA | AACAACACATGATTAAAGGAGCA | Linkage mapping |
| 075c05_3_2 | TTAAATGTCGTATCCAAGCAACG |  | Linkage mapping |
| 075c05_5 | AGCTTGAGTCCTGGTTTCATTT | CGCCATTTGACAATACTGTTCC | Linkage mapping |
| 075c05_5_2 | TTTGCCACCAGTTAAAATTGTCT |  | Linkage mapping |
| 077j19_3 | CTTTGATTCTCTCAACGGTGCAA | AACAGTGGGATATTCTTTCAAATGG | Linkage mapping |
| 077j19_5 | GAGAACCCTATATGTAAATGCATCA | AGAAGATGTATTCCCTAACAAGCA | Linkage mapping |
| 089l06_3 | TTCTGAGTTACATCGTGCTTCC | CATCCAAGTCCATGTAATCCAA | Linkage mapping |
| 089l06_3_2 | GCTACATCACTATAAGGACGAATTG | TCGAAAAGAAGAAAACTCAGGTG | Linkage mapping |
| 089l06_5 | TTTGCAACCATATAACAATGCAC | AAAAATTAACTTGCAAACAGACC | Linkage mapping |
| 092k09_3 | AGAGGGTGAGAATCCCGTCTT | CGACGAGAGGCTATAACACACC | Linkage mapping |
| 092k09_5 | AAGCTTGGAGTTGTAGTCCATTG | TCAGGGTTCTATTATTTCTTGTTGC | Linkage mapping |
| 096g16_3 | AAGCTTTATGGCATATGATTTCT | TGTGCTCATATTACACCCTGGA | Linkage mapping |
| 096g16_3_2 | CTTTCATTTCCCTCAAAATTCAAC | GTTTTATGTATTACTGCTTGTGATG | Linkage mapping |
| 096g16_5 | AAGCTTGAGAGCCATTAGAGG | GTCTTGGATTCCTGAGGTTATTTC | Linkage mapping |
| 096g16_5_2 | GCTCCAGTATTCCTGAGACAAACC | GCTCAAAACTCATCCTCAAACTG | Linkage mapping |
| 112b24_3 | TTGACTCAAGCTATCAATTTGC | TCTTCACCGTCAAATATTTTCAGA | Linkage mapping |
| 112b24_5 | TCAAGATTCAAGCAATACATCATTC | GATGCCTTAAAAGGAAGAAATGG | Linkage mapping |
| 126d14_3 | CGGGGAAGGATTTAAAACAAAT | ATGATTTGCAATTGATTGTCTG | Linkage mapping |
| 126d14_5 | AAGCTTAACCCCGTAAAGGTAAT | GTTATGAAAAGCATGTTGTTTTG | Linkage mapping |

# Table S2. BAC-end mapping to the *L. angustifolius* genome

|  | **Query** | **Query coverage** | **Sequence Length** | **Name** | **% Pairwise Identity** | **Bit-Score** | **E Value** | **Hit start** | **Hit end** | **Query start** | **Query end** | **ACCase genes** |
| --- | --- | --- | --- | --- | --- | --- | --- | --- | --- | --- | --- | --- |
| s1 | 049O09_3 | 100.0% | 30153019 | NLL-03 | 100.0% | 1026.0 | 0 | 10143612 | 10144166 | 1 | 555 | - |
|  | 049O09_5 | 100.0% | 30153019 | NLL-03 | 99.4% | 1164.5 | 0 | 10170381 | 10169742 | 1 | 644 | - |
| s2 | 002B02_3 | 100.0% | 27333975 | NLL-04 | 100.0% | 1059.3 | 0 | 11471 | 12043 | 1 | 573 | - |
|  | 002B02_5 | 100.0% | 27333975 | NLL-04 | 99.8% | 1131.3 | 0 | 43833 | 43219 | 1 | 615 | - |
| c1 | 126D14_5 | 100.0% | 33111450 | NLL-06 | 99.8% | 1155.3 | 0 | 11936426 | 11937053 | 1 | 628 | accC1 |
|  | 009A01_5 | 100.0% | 33111450 | NLL-06 | 99.7% | 1144.2 | 0 | 11944094 | 11944719 | 1 | 625 | accC1 |
|  | 126D14_3 | 100.0% | 33111450 | NLL-06 | 99.8% | 1195.9 | 0 | 11949426 | 11948776 | 1 | 650 | accC1 |
|  | 009A01_3 | 99.9% | 33111450 | NLL-06 | 99.6% | 1249.5 | 0 | 11995250 | 11994565 | 1 | 684 | accC1 |
| c2 | 077J19_3 | 93.4% | 19782170 | NLL-07 | 99.9% | 1297.5 | 0 | 3026000 | 3026704 | 51 | 756 | - |
|  | 077J19_5 | 85.8% | 19782170 | NLL-07 | 99.7% | 1168.2 | 0 | 3086249 | 3085612 | 107 | 744 | - |
|  | 089L06_5 | 100.0% | 19782170 | NLL-07 | 95.5% | 983.5 | 0 | 7343488 | 7344102 | 1 | 617 | - |
|  | 089L06_3 | 100.0% | 19782170 | NLL-07 | 99.8% | 1109.1 | 0 | 7366906 | 7366304 | 1 | 603 | - |
| s3 | 112B24_5 | 100.0% | 21753712 | NLL-09 | 98.7% | 1112.8 | 0 | 12273756 | 12274380 | 1 | 627 | - |
|  | 112B21_3 | 100.0% | 21753712 | NLL-09 | 99.8% | 1103.6 | 0 | 12306757 | 12306158 | 1 | 600 | - |
| s4 | 002F03_5 | 100.0% | 16341955 | NLL-10 | 99.9% | 1240.2 | 0 | 12597565 | 12598238 | 1 | 675 | - |
|  | 002F03_3 | 100.0% | 16341955 | NLL-10 | 99.8% | 1151.6 | 0 | 12624754 | 12624129 | 1 | 626 | - |
| c3 | 049F04_3 | 100.0% | 35963958 | NLL-11 | 99.1% | 1057.4 | 0 | 3346101 | 3346688 | 1 | 586 | - |
|  | 096G16_3 | 100.0% | 35963958 | NLL-11 | 99.5% | 1094.3 | 0 | 3383246 | 3382645 | 1 | 601 | - |
|  | 096G16_5 | 100.0% | 35963958 | NLL-11 | 97.3% | 935.5 | 0 | 3386746 | 3387295 | 1 | 552 | - |
| s5 | 042E20_3 | 100.0% | 35963958 | NLL-11 | 100.0% | 1153.4 | 0 | 9662171 | 9662794 | 1 | 624 | - |
| c4 | 056N03_5 | 98.8% | 35963958 | NLL-11 | 96.5% | 1123.9 | 0 | 33412418 | 33413098 | 1 | 679 | accA2 |
|  | 020A06_3 | 100.0% | 35963958 | NLL-11 | 99.6% | 1122.0 | 0 | 33434643 | 33435255 | 1 | 613 | accA2 |
|  | 030F02_5 | 100.0% | 35963958 | NLL-11 | 96.8% | 968.8 | 0 | 33445214 | 33445786 | 1 | 589 | accA2 |
|  | 046I04_3 | 100.0% | 35963958 | NLL-11 | 95.3% | 713.9 | 0 | 33451802 | 33452251 | 1 | 449 | accA2 |
|  | 060F02_3 | 99.6% | 35963958 | NLL-11 | 96.1% | 911.5 | 0 | 33453271 | 33453832 | 3 | 558 | accA2 |
|  | 016J11_5 | 100.0% | 35963958 | NLL-11 | 94.9% | 1026.0 | 0 | 33471683 | 33472336 | 1 | 661 | accA2 |
|  | 048N08_3 | 100.0% | 35963958 | NLL-11 | 93.6% | 778.6 | 0 | 33471683 | 33472197 | 1 | 531 | accA2 |
|  | 020A06_5 | 100.0% | 35963958 | NLL-11 | 98.1% | 1173.7 | 0 | 33513216 | 33512543 | 1 | 675 | accA2 |
|  | 056N03_3 | 98.9% | 35963958 | NLL-11 | 95.4% | 448.0 | 2.13E-124 | 33516643 | 33516361 | 1 | 280 | accA2 |
|  | 046I04_5 | 100.0% | 35963958 | NLL-11 | 96.8% | 1077.7 | 0 | 33530364 | 33529718 | 1 | 645 | accA2 |
|  | 016J11_3 | 100.0% | 35963958 | NLL-11 | 98.8% | 1033.4 | 0 | 33531753 | 33531174 | 1 | 581 | accA2 |
|  | 060F02_5 | 100.0% | 35963958 | NLL-11 | 97.4% | 1044.5 | 0 | 33536705 | 33536096 | 1 | 616 | accA2 |
|  | 030F02_3 | 100.0% | 35963958 | NLL-11 | 99.4% | 874.6 | 0 | 33544447 | 33543966 | 1 | 483 | accA2 |
|  | 048N08_5 | 100.0% | 35963958 | NLL-11 | 97.6% | 710.2 | 0 | 33582031 | 33581619 | 1 | 416 | accA2 |
| s6 | 002E06_5 | 100.0% | 17820680 | NLL-13 | 99.4% | 1291.9 | 0 | 8569097 | 8569808 | 1 | 711 | - |
|  | 002E06_3 | 100.0% | 17820680 | NLL-13 | 100.0% | 1159.0 | 0 | 8622463 | 8621837 | 1 | 627 | - |
| s7 | 051F15_5 | 100.0% | 17820680 | NLL-13 | 99.7% | 1170.1 | 0 | 16669801 | 16670438 | 1 | 640 | accA1 |
|  | 051F15_3 | 100.0% | 17820680 | NLL-13 | 99.5% | 1210.7 | 0 | 16728302 | 16727639 | 1 | 664 | accA1 |
| c5 | 009K06_3 | 100.0% | 16251777 | NLL-14 | 99.8% | 1164.5 | 0 | 13277657 | 13278289 | 1 | 634 | ACC1 |
|  | 040M06_5 | 100.0% | 16251777 | NLL-14 | 99.1% | 985.4 | 0 | 13323180 | 13323727 | 1 | 549 | ACC1 |
|  | 070D20_5 | 100.0% | 16251777 | NLL-14 | 98.1% | 1085.1 | 0 | 13323180 | 13323801 | 1 | 624 | ACC1 |
|  | 070D20_3 | 100.0% | 16251777 | NLL-14 | 97.9% | 915.2 | 0 | 13375275 | 13374751 | 1 | 531 | ACC1 |
|  | 009K06_5 | 100.0% | 16251777 | NLL-14 | 99.8% | 1170.1 | 0 | 13375466 | 13374831 | 1 | 637 | ACC1 |
|  | 040M06_3 | 100.0% | 16251777 | NLL-14 | 99.5% | 1190.4 | 0 | 13400417 | 13399764 | 1 | 653 | ACC1 |
| s8 | 073E17_5 | 100.0% | 20964703 | NLL-15 | 96.9% | 1072.2 | 0 | 17546663 | 17547304 | 1 | 637 | ACC2 |
|  | 073E17_3 | 100.0% | 20964703 | NLL-15 | 93.9% | 898.6 | 0 | 17590399 | 17589795 | 1 | 589 | ACC2 |
| s9 | 092K09_5 | 100.0% | 23035 | S_21_146 | 99.1% | 990.9 | 0 | 4331 | 3781 | 1 | 551 | - |
| c6 | 034G21_3 | 45.7% | 665528 | S_287 | 97.1% | 291.0 | 5.18E-77 | 445740 | 445911 | 1 | 172 | accB1 |
|  | 066E06_5 | 99.8% | 665528 | S_287 | 98.6% | 1092.5 | 0 | 449063 | 449685 | 1 | 614 | accB1 |
|  | 069L17_5 | 100.0% | 665528 | S_287 | 99.1% | 1181.1 | 0 | 508772 | 509431 | 1 | 654 | accB1 |
|  | 075C05_5 | 99.8% | 665528 | S_287 | 99.5% | 1144.2 | 0 | 508772 | 509400 | 1 | 627 | accB1 |
|  | 011G20_3 | 100.0% | 665528 | S_287 | 99.9% | 1415.7 | 0 | 527984 | 528753 | 1 | 769 | accB1 |
|  | 066E06_3 | 100.0% | 665528 | S_287 | 98.8% | 1225.5 | 0 | 543979 | 543290 | 1 | 684 | accB1 |
|  | 011G20_5 | 100.0% | 665528 | S_287 | 99.4% | 1127.6 | 0 | 559750 | 559127 | 1 | 620 | accB1 |
|  | 034G21_5 | 100.0% | 665528 | S_287 | 98.4% | 752.7 | 0 | 559750 | 559326 | 1 | 431 | accB1 |
|  | 069L17_3 | 100.0% | 665528 | S_287 | 99.7% | 1256.8 | 0 | 585819 | 585134 | 1 | 686 | accB1 |
|  | 075C05_3 | 100.0% | 665528 | S_287 | 99.4% | 1227.3 | 0 | 600440 | 599764 | 1 | 675 | accB1 |
| s10 | 042C13_5 | 100.0% | 15678 | S_35_61 | 99.9% | 1266.1 | 0 | 13896 | 14583 | 1 | 688 | - |
|  | 042C13_3 | 100.0% | 35290 | S_35_66 | 99.7% | 1243.9 | 0 | 4017 | 3339 | 1 | 679 | - |
| s11 | 005G02_5 | 100.0% | 82758 | S_5_199 | 100.0% | 1136.8 | 0 | 20426 | 21040 | 1 | 615 | - |
|  | 005G02_3 | 100.0% | 82758 | S_5_199 | 99.7% | 1194.1 | 0 | 56569 | 55919 | 1 | 653 | - |
|  | 042E20_5 | 99.7% | 83510 | S_566 | 99.3% | 1256.8 | 0 | 30839 | 30147 | 1 | 697 | - |
|  | 049F04_5 | 100.0% | 18672 | S_74_2 | 99.5% | 1072.2 | 0 | 11310 | 10723 | 1 | 590 | - |
|  | 092K09_3 | - | - | - | - | - | - | - | - | - | - | - |
|  | - | - | - | - | - | - | - | - | - | - | - | accA3 |
|  | - | - | - | - | - | - | - | - | - | - | - | accB2a |
|  | - | - | - | - | - | - | - | - | - | - | - | accB2b |
|  | - | - | - | - | - | - | - | - | - | - | - | accB2c |
|  | - | - | - | - | - | - | - | - | - | - | - | accC2 |

s1, s2, …, s11 - singletons

c1, c2, …, c6 - contigs

# Table S3. Segregation data for molecular markers developed for 83:476 × P27255 *Lupinus angustifolius* recombinant inbred line mapping population.

| **Marker name** | 97L380-008 | 97L380-009 | 97L380-010 | 97L380-011 | 97L380-012 | 97L380-013 | 97L380-014 | 97L380-015 | 97L380-017 | 97L380-018 | 97L380-021 | 97L380-023 | 97L380-024 | 97L380-025 | 97L380-026 | 97L380-027 | 97L380-028 | 97L380-032 | 97L380-033 | 97L380-034 | 97L380-035 | 97L380-036 | 97L380-037 | 97L380-038 | 97L380-039 | 97L380-040 |
| --- | --- | --- | --- | --- | --- | --- | --- | --- | --- | --- | --- | --- | --- | --- | --- | --- | --- | --- | --- | --- | --- | --- | --- | --- | --- | --- |
| 002F03_5 | 1 | 0 | 2 | 0 | 2 | 0 | 1 | 0 | 1 | 2 | 2 | 1 | 1 | 2 | 1 | 1 | 2 | 0 | 0 | 1 | 1 | 2 | 0 | 2 | 2 | 2 |
| 005G02_3 | 0 | 1 | 0 | 0 | 0 | 2 | 1 | 2 | 2 | 0 | 1 | 1 | 1 | 1 | 0 | 0 | 1 | 0 | 0 | 0 | 0 | 0 | 0 | 1 | 2 | 2 |
| 009K06_5 | 1 | 0 | 1 | 0 | 1 | 0 | 1 | 0 | 2 | 2 | 2 | 2 | 0 | 1 | 2 | 2 | 1 | 0 | 0 | 1 | 2 | 2 | 0 | 2 | 0 | 2 |
| 011G20_5 | 1 | 0 | 1 | 0 | 1 | 2 | 1 | 1 | 2 | 2 | 1 | 2 | 1 | 1 | 2 | 2 | 2 | 0 | 0 | 2 | 2 | 1 | 0 | 2 | 2 | 2 |
| 016J11_5 | 2 | 1 | 1 | 0 | 2 | 0 | 1 | 2 | 2 | 2 | 1 | 2 | 2 | 2 | 1 | 1 | 2 | 0 | 0 | 2 | 1 | 2 | 0 | 1 | 2 | 2 |
| 040M06_3 | 1 | 0 | 1 | 0 | 1 | 0 | 1 | 1 | 2 | 2 | 2 | 2 | 0 | 1 | 2 | 2 | 1 | 0 | 0 | 1 | 2 | 2 | 0 | 2 | 0 | 0 |
| 042C13_3 | 0 | 2 | 1 | 0 | 0 | 1 | 0 | 2 | 1 | 2 | 2 | 1 | 1 | 2 | 2 | 2 | 2 | 0 | 0 | 2 | 1 | 0 | 0 | 1 | 1 | 2 |
| 051F15_5 | 0 | 2 | 1 | 0 | 2 | 1 | 1 | 1 | 0 | 2 | 1 | 2 | 1 | 1 | 2 | 2 | 2 | 0 | 0 | 2 | 1 | 0 | 0 | 1 | 0 | 1 |
| 060F02_3 | 0 | 0 | 1 | 0 | 2 | 2 | 1 | 2 | 2 | 2 | 1 | 2 | 2 | 2 | 1 | 1 | 2 | 0 | 0 | 2 | 1 | 2 | 0 | 1 | 2 | 2 |
| 069L17_3 | 1 | 2 | 1 | 0 | 1 | 0 | 1 | 1 | 2 | 2 | 1 | 0 | 1 | 1 | 2 | 2 | 2 | 0 | 0 | 2 | 0 | 1 | 0 | 2 | 2 | 0 |
| 073E17_5 | 2 | 1 | 2 | 0 | 2 | 0 | 2 | 1 | 1 | 1 | 1 | 1 | 2 | 1 | 2 | 2 | 2 | 0 | 0 | 1 | 0 | 0 | 0 | 1 | 2 | 0 |
| 077J19_5 | 1 | 2 | 2 | 0 | 1 | 2 | 2 | 1 | 2 | 2 | 2 | 2 | 2 | 2 | 1 | 1 | 2 | 0 | 0 | 2 | 1 | 2 | 0 | 2 | 1 | 2 |
| 112B24_5 | 1 | 1 | 1 | 0 | 1 | 0 | 2 | 2 | 0 | 1 | 2 | 0 | 1 | 2 | 2 | 0 | 1 | 0 | 0 | 1 | 2 | 2 | 0 | 1 | 1 | 1 |
| 126D14_5 | 0 | 2 | 2 | 0 | 1 | 2 | 1 | 2 | 1 | 1 | 1 | 2 | 1 | 2 | 1 | 2 | 1 | 0 | 0 | 2 | 2 | 2 | 0 | 2 | 1 | 1 |

| **Marker name** | 97L380-041 | 97L380-042 | 97L380-044 | 97L380-045 | 97L380-046 | 97L380-047 | 97L380-048 | 97L380-049 | 97L380-050 | 97L380-051 | 97L380-052 | 97L380-053 | 97L380-054 | 97L380-055 | 97L380-057 | 97L380-058 | 97L380-059 | 97L380-061 | 97L380-062 | 97L380-063 | 97L380-064 | 97L380-065 | 97L380-066 | 97L380-067 | 97L380-069 | 97L380-070 |
| --- | --- | --- | --- | --- | --- | --- | --- | --- | --- | --- | --- | --- | --- | --- | --- | --- | --- | --- | --- | --- | --- | --- | --- | --- | --- | --- |
| 002F03_5 | 2 | 1 | 2 | 0 | 2 | 0 | 2 | 1 | 1 | 2 | 1 | 1 | 2 | 0 | 2 | 2 | 1 | 0 | 0 | 0 | 1 | 1 | 0 | 0 | 0 | 1 |
| 005G02_3 | 1 | 2 | 0 | 2 | 1 | 0 | 1 | 0 | 0 | 2 | 2 | 1 | 2 | 0 | 0 | 1 | 0 | 0 | 0 | 0 | 1 | 0 | 2 | 0 | 0 | 2 |
| 009K06_5 | 1 | 1 | 2 | 0 | 2 | 0 | 2 | 1 | 1 | 1 | 1 | 1 | 1 | 0 | 1 | 2 | 2 | 0 | 0 | 0 | 1 | 1 | 1 | 0 | 2 | 1 |
| 011G20_5 | 1 | 1 | 2 | 2 | 1 | 0 | 1 | 1 | 0 | 1 | 2 | 2 | 1 | 0 | 1 | 1 | 1 | 0 | 0 | 0 | 2 | 2 | 2 | 0 | 1 | 1 |
| 016J11_5 | 2 | 1 | 1 | 0 | 2 | 0 | 1 | 1 | 2 | 2 | 1 | 1 | 2 | 0 | 2 | 2 | 1 | 0 | 0 | 0 | 1 | 2 | 2 | 0 | 1 | 1 |
| 040M06_3 | 1 | 1 | 0 | 1 | 2 | 0 | 2 | 1 | 1 | 0 | 1 | 1 | 1 | 0 | 0 | 2 | 2 | 0 | 0 | 0 | 1 | 0 | 1 | 0 | 0 | 1 |
| 042C13_3 | 2 | 1 | 2 | 1 | 2 | 0 | 1 | 0 | 1 | 1 | 2 | 1 | 1 | 0 | 1 | 2 | 2 | 0 | 0 | 0 | 1 | 2 | 2 | 0 | 2 | 2 |
| 051F15_5 | 1 | 1 | 1 | 1 | 2 | 0 | 1 | 0 | 2 | 1 | 2 | 1 | 2 | 0 | 1 | 1 | 1 | 0 | 0 | 0 | 2 | 2 | 2 | 0 | 1 | 1 |
| 060F02_3 | 2 | 1 | 1 | 2 | 2 | 0 | 1 | 1 | 2 | 2 | 1 | 1 | 2 | 0 | 2 | 2 | 1 | 0 | 0 | 0 | 1 | 2 | 2 | 0 | 1 | 1 |
| 069L17_3 | 0 | 1 | 2 | 0 | 2 | 0 | 1 | 1 | 2 | 1 | 2 | 2 | 0 | 0 | 1 | 1 | 1 | 0 | 0 | 0 | 2 | 2 | 2 | 0 | 1 | 1 |
| 073E17_5 | 1 | 0 | 1 | 2 | 2 | 0 | 2 | 0 | 2 | 2 | 1 | 1 | 1 | 0 | 1 | 2 | 2 | 0 | 0 | 0 | 1 | 0 | 2 | 0 | 1 | 1 |
| 077J19_5 | 1 | 1 | 1 | 1 | 2 | 0 | 1 | 2 | 2 | 2 | 1 | 1 | 1 | 0 | 1 | 2 | 1 | 0 | 0 | 0 | 2 | 2 | 1 | 0 | 2 | 2 |
| 112B24_5 | 0 | 2 | 2 | 0 | 2 | 0 | 1 | 1 | 2 | 2 | 1 | 1 | 1 | 0 | 1 | 1 | 2 | 0 | 0 | 0 | 2 | 1 | 2 | 0 | 2 | 2 |
| 126D14_5 | 2 | 2 | 1 | 1 | 2 | 0 | 2 | 1 | 2 | 2 | 1 | 1 | 2 | 0 | 2 | 2 | 2 | 0 | 0 | 0 | 2 | 1 | 1 | 0 | 1 | 1 |

| **Marker name** | 97L380-071 | 97L380-073 | 97L380-074 | 97L380-075 | 97L380-076 | 97L380-077 | 97L380-078 | 97L380-079 | 97L380-080 | 97L380-081 | 97L380-082 | 97L380-083 | 97L380-084 | 97L380-085 | 97L380-086 | 97L380-089 | 97L380-092 | 97L380-093 | 97L380-094 | 97L380-095 | 97L380-097 | 97L380-098 | 97L380-099 | 97L380-100 | 97L380-101 | 97L380-102 |
| --- | --- | --- | --- | --- | --- | --- | --- | --- | --- | --- | --- | --- | --- | --- | --- | --- | --- | --- | --- | --- | --- | --- | --- | --- | --- | --- |
| 002F03_5 | 0 | 0 | 1 | 0 | 1 | 1 | 1 | 0 | 2 | 0 | 2 | 0 | 0 | 2 | 1 | 1 | 2 | 1 | 2 | 2 | 1 | 1 | 2 | 1 | 0 | 0 |
| 005G02_3 | 0 | 0 | 2 | 0 | 1 | 2 | 1 | 0 | 2 | 0 | 2 | 2 | 2 | 1 | 0 | 2 | 0 | 2 | 0 | 2 | 2 | 1 | 2 | 2 | 0 | 1 |
| 009K06_5 | 0 | 2 | 2 | 0 | 2 | 1 | 1 | 0 | 2 | 0 | 1 | 1 | 1 | 1 | 1 | 1 | 1 | 2 | 1 | 2 | 2 | 1 | 1 | 2 | 0 | 0 |
| 011G20_5 | 0 | 1 | 2 | 0 | 2 | 2 | 1 | 0 | 1 | 0 | 1 | 2 | 2 | 1 | 1 | 2 | 2 | 2 | 2 | 2 | 0 | 2 | 1 | 2 | 0 | 1 |
| 016J11_5 | 0 | 1 | 2 | 0 | 1 | 2 | 2 | 0 | 2 | 0 | 2 | 2 | 2 | 2 | 2 | 2 | 2 | 2 | 1 | 1 | 2 | 2 | 2 | 1 | 0 | 1 |
| 040M06_3 | 0 | 2 | 0 | 0 | 2 | 1 | 1 | 0 | 2 | 0 | 1 | 1 | 0 | 1 | 1 | 1 | 1 | 2 | 1 | 2 | 0 | 1 | 1 | 2 | 0 | 2 |
| 042C13_3 | 0 | 2 | 2 | 0 | 1 | 2 | 1 | 0 | 2 | 0 | 1 | 2 | 2 | 2 | 0 | 1 | 2 | 1 | 1 | 2 | 2 | 1 | 1 | 1 | 0 | 1 |
| 051F15_5 | 0 | 2 | 2 | 0 | 1 | 2 | 1 | 0 | 1 | 0 | 1 | 2 | 2 | 1 | 1 | 2 | 2 | 2 | 2 | 2 | 0 | 2 | 1 | 1 | 0 | 1 |
| 060F02_3 | 0 | 1 | 2 | 0 | 1 | 2 | 2 | 0 | 2 | 0 | 2 | 2 | 2 | 2 | 2 | 2 | 2 | 2 | 1 | 1 | 2 | 2 | 2 | 1 | 0 | 1 |
| 069L17_3 | 0 | 1 | 2 | 0 | 2 | 2 | 1 | 0 | 1 | 0 | 1 | 2 | 2 | 1 | 1 | 2 | 2 | 2 | 2 | 2 | 1 | 2 | 1 | 2 | 0 | 1 |
| 073E17_5 | 0 | 0 | 2 | 0 | 2 | 1 | 2 | 0 | 2 | 0 | 1 | 2 | 2 | 1 | 0 | 2 | 1 | 2 | 2 | 1 | 1 | 2 | 2 | 2 | 0 | 2 |
| 077J19_5 | 0 | 2 | 2 | 0 | 1 | 2 | 2 | 0 | 1 | 0 | 2 | 1 | 1 | 1 | 2 | 1 | 2 | 2 | 1 | 1 | 2 | 2 | 1 | 1 | 0 | 1 |
| 112B24_5 | 0 | 1 | 1 | 0 | 2 | 2 | 1 | 0 | 1 | 0 | 1 | 2 | 2 | 2 | 2 | 2 | 1 | 1 | 1 | 2 | 2 | 2 | 1 | 1 | 0 | 2 |
| 126D14_5 | 0 | 2 | 2 | 0 | 2 | 1 | 2 | 0 | 2 | 0 | 2 | 1 | 0 | 1 | 1 | 2 | 2 | 1 | 1 | 1 | 2 | 1 | 1 | 2 | 0 | 2 |

| **Marker name** | 97L380-105 | 97L380-106 | 97L380-109 | 97L380-111 | 97L380-112 | 97L380-113 | 97L380-114 | 97L380-115 | 97L380-116 | 97L380-117 | 97L380-118 | 97L380-119 | 97L380-120 | 97L380-121 | 97L380-123 | 97L380-124 | 97L380-125 | 97L380-127 | 97L380-128 | 97L380-130 | 97L380-132 | 97L380-135 | 97L380-136 | 83A:476 | P27255 |
| --- | --- | --- | --- | --- | --- | --- | --- | --- | --- | --- | --- | --- | --- | --- | --- | --- | --- | --- | --- | --- | --- | --- | --- | --- | --- |
| 002F03_5 | 0 | 2 | 2 | 1 | 2 | 2 | 1 | 0 | 0 | 0 | 2 | 1 | 1 | 0 | 2 | 2 | 1 | 1 | 1 | 1 | 1 | 2 | 1 | 1 | 2 |
| 005G02_3 | 0 | 1 | 1 | 2 | 2 | 0 | 2 | 0 | 1 | 0 | 1 | 2 | 1 | 0 | 2 | 2 | 0 | 2 | 1 | 2 | 0 | 2 | 1 | 1 | 2 |
| 009K06_5 | 0 | 1 | 1 | 0 | 0 | 1 | 2 | 0 | 2 | 0 | 0 | 2 | 2 | 0 | 1 | 1 | 0 | 0 | 0 | 0 | 0 | 2 | 1 | 1 | 2 |
| 011G20_5 | 0 | 2 | 0 | 2 | 2 | 2 | 1 | 0 | 1 | 0 | 2 | 1 | 1 | 0 | 1 | 2 | 2 | 2 | 1 | 2 | 2 | 1 | 0 | 1 | 2 |
| 016J11_5 | 0 | 2 | 2 | 2 | 2 | 2 | 2 | 0 | 1 | 0 | 1 | 1 | 1 | 0 | 2 | 2 | 2 | 2 | 2 | 2 | 1 | 0 | 2 | 1 | 2 |
| 040M06_3 | 0 | 1 | 0 | 1 | 1 | 0 | 2 | 0 | 2 | 0 | 1 | 2 | 2 | 0 | 1 | 1 | 2 | 2 | 1 | 1 | 2 | 2 | 1 | 1 | 2 |
| 042C13_3 | 0 | 1 | 2 | 1 | 2 | 2 | 1 | 0 | 1 | 0 | 2 | 1 | 1 | 0 | 1 | 2 | 2 | 2 | 2 | 1 | 1 | 0 | 1 | 1 | 2 |
| 051F15_5 | 0 | 2 | 1 | 2 | 1 | 2 | 1 | 0 | 2 | 0 | 2 | 1 | 2 | 0 | 2 | 2 | 2 | 2 | 1 | 1 | 1 | 1 | 2 | 1 | 2 |
| 060F02_3 | 0 | 2 | 2 | 2 | 2 | 2 | 2 | 0 | 1 | 0 | 1 | 1 | 2 | 0 | 2 | 2 | 2 | 2 | 2 | 2 | 1 | 2 | 2 | 1 | 2 |
| 069L17_3 | 0 | 2 | 1 | 2 | 2 | 2 | 1 | 0 | 1 | 0 | 2 | 1 | 1 | 0 | 1 | 2 | 2 | 2 | 1 | 2 | 2 | 1 | 0 | 1 | 2 |
| 073E17_5 | 0 | 0 | 2 | 1 | 2 | 2 | 2 | 0 | 1 | 0 | 1 | 0 | 2 | 0 | 0 | 0 | 2 | 0 | 1 | 2 | 2 | 0 | 0 | 1 | 2 |
| 077J19_5 | 0 | 1 | 1 | 1 | 2 | 2 | 2 | 0 | 2 | 0 | 2 | 2 | 2 | 0 | 1 | 1 | 2 | 1 | 1 | 1 | 1 | 1 | 2 | 1 | 2 |
| 112B24_5 | 0 | 1 | 0 | 2 | 1 | 1 | 2 | 0 | 2 | 0 | 1 | 2 | 1 | 0 | 2 | 1 | 2 | 2 | 2 | 1 | 2 | 0 | 0 | 1 | 2 |
| 126D14_5 | 0 | 1 | 2 | 2 | 1 | 2 | 2 | 0 | 1 | 0 | 1 | 2 | 1 | 0 | 1 | 1 | 2 | 1 | 1 | 1 | 1 | 1 | 1 | 1 | 2 |

# Table S4. Copy number evaluation of *L. angustifolius* cytosolic ACCase and subunits of plastid ACCase genes by Droplet Digital PCR

| **Product (gene)** | **Sample concentration (ng/μl)** | **Accepted droplets** | **Positives** | **Negatives** | **Conc (copies/µl)** | **Poisson Confidence Max68** | **Poisson Confidence Min68** |
| --- | --- | --- | --- | --- | --- | --- | --- |
| *ACC** | 1 | 12526 | 3587 | 8939 | 397 | 404 | 390 |
| *ACC* | 1 | 14790 | 4284 | 10506 | 402 | 409 | 396 |
| *ACC* | 0.5 | 17503 | 2353 | 15150 | 170 | 173.4 | 166.3 |
| *accA* | 0.125 | 16313 | 1387 | 14926 | 105 | 107.3 | 101.7 |
| *accA* | 0.125 | 17152 | 1093 | 16059 | 77.5 | 79.8 | 75.1 |
| *accA* | 0.25 | 16366 | 3454 | 12912 | 279 | 283.6 | 274.1 |
| *accB* | 0.25 | 14065 | 1152 | 12913 | 101 | 103.5 | 97.6 |
| *accB* | 0.25 | 14030 | 1237 | 12793 | 109 | 111.7 | 105.5 |
| *accB* | 0.5 | 17457 | 2717 | 14740 | 199 | 202.9 | 195.2 |
| *accC* | 0.25 | 13197 | 2296 | 10901 | 225 | 229.6 | 220.2 |
| *accC* | 0.5 | 17355 | 3848 | 13507 | 275 | 284 | 267 |
| *accC* | 0.5 | 16943 | 3234 | 13709 | 249 | 253.6 | 244.8 |

# Table S5. Sequence coordinates for cytosolic ACCase and subunits of plastid ACCase genes derived from legume genome assemblies

| **CDS** | **CDS start** | **CDS end** | **Chromosome**  **name** | **Gene start** | **Gene end** | **Gene length** | **Exon number** |
| --- | --- | --- | --- | --- | --- | --- | --- |
| Adu_ACC_Aradu.GHK20 | 1 | 6789 | Ad08 | 12461246 | 12449634 | 11613 | 31 |
| Aip_ACC_Araip.ZAK9V | 1 | 6789 | Ai07 | 120612450 | 120600764 | 11687 | 31 |
| Ath_ACC1_AT1G36160 | 1 | 6762 | At01 | 13534196 | 13543770 | 9575 | 31 |
| Ath_ACC2_AT1G36180 | 1 | 7065 | At01 | 13546047 | 13558336 | 12290 | 32 |
| Car_ACC_Ca_16623 | 1 | 6795 | Ca05 | 29285702 | 29273994 | 11709 | 31 |
| Cca_ACC_C.cajan_25715 | 1 | 6780 | Cc_Scaffold000332 | 379492 | 392486 | 12995 | 31 |
| Gma_ACC1_Glyma.04G104900 | 1 | 6780 | Gm04 | 9984664 | 9998306 | 13643 | 31 |
| Gma_ACC2_Glyma.06G105900 | 1 | 6780 | Gm06 | 8469468 | 8483086 | 13619 | 31 |
| Lan_ACC1_Lup012661 | 1 | 6774 | NLL-14 | 13357159 | 13345356 | 11804 | 31 |
| Lan_ACC2_Lup009248 | 1 | 6786 | NLL-15 | 17582721 | 17571125 | 11597 | 31 |
| Lja_ACC_Lj0g3v0268829 | 1 | 6771 | Lj0 | 139228799 | 139242840 | 14042 | 31 |
| Mtr_ACC1_Medtr3g073820 | 1 | 6657 | Mt03 | 33332916 | 33318874 | 14043 | 29 |
| Mtr_ACC2_Medtr3g073860 | 1 | 6768 | Mt03 | 33354381 | 33344237 | 10145 | 29 |
| Pvu_ACC_Phvul.009G028700 | 1 | 6780 | Pv09 | 6373338 | 6359268 | 14071 | 31 |
| Vra_ACC_Vradi05g07690 | 1 | 6780 | Vr05 | 15266129 | 15280165 | 14037 | 31 |
| Adu_accA1_Aradu.14CMN | 1 | 2259 | Ad04 | 87675728 | 87680686 | 4959 | 10 |
| Adu_accA2_Aradu.Z0G82 | 1 | 2037 | Ad04 | 109440671 | 109433304 | 7368 | 11 |
| Aip_accA1_Araip.7H2NS | 1 | 2259 | Ai04 | 96083512 | 96088540 | 5029 | 10 |
| Aip_accA2_Araip.77JRH | 1 | 2007 | Ai04 | 118074209 | 118070544 | 3666 | 10 |
| Ath_accA_AT2G38040 | 1 | 2307 | At02 | 15917612 | 15920746 | 3135 | 10 |
| Car_accA_Ca_26217 | 1 | 2265 | Ca_scaffold561 | 45199 | 49787 | 4589 | 10 |
| Cca_accA1_C.cajan_18479 | 1 | 2043 | Cc07 | 12723108 | 12708279 | 14830 | 11 |
| Cca_accA2_C.cajan_33027 | 1 | 2223 | Cc_Scaffold127360 | 358587 | 353779 | 4809 | 11 |
| Gma_accA1_Glyma.18G195700 | 1 | 2127 | Gm18 | 51290598 | 51294940 | 4343 | 10 |
| Gma_accA2_Glyma.18G195900 | 1 | 2070 | Gm18 | 51322895 | 51327191 | 4297 | 10 |
| Gma_accA3_Glyma.18G196000 | 1 | 2049 | Gm18 | 51354672 | 51360262 | 5591 | 10 |
| Lan_accA1_Lup023032 | 1 | 2274 | NLL-13 | 16696622 | 16701405 | 4784 | 10 |
| Lan_accA2_Lup010366 | 1 | 2280 | NLL-11 | 33482743 | 33478537 | 4207 | 10 |
| Lan_accA3_Lup014474 | 1 | 2274 | NLL-07 | 18818156 | 18822855 | 4700 | 10 |
| Lja_accA1_Lj0g3v0256879 | 1 | 2172 | Lj0 | 133350740 | 133344432 | 6309 | 11 |
| Lja_accA2_Lj1g3v2628750 | 1 | 1983 | Lj1 | 30761544 | 30756286 | 5259 | 10 |
| Mtr_accA_Medtr7g066870 | 1 | 2172 | Mt07 | 24404819 | 24399344 | 5476 | 12 |
| Pvu_accA_Phvul.008G108700 | 1 | 2232 | Pv08 | 12640127 | 12635243 | 4885 | 10 |
| Vra_accA_Vradi06g10650 | 1 | 2217 | Vr06 | 24908570 | 24912953 | 4384 | 10 |
| Adu_accB1_Aradu.JDP66 | 1 | 837 | Ad04 | 113608924 | 113605049 | 3876 | 7 |
| Adu_accB2a_Aradu.0JT6M | 1 | 879 | Ad04 | 118543543 | 118546299 | 2757 | 7 |
| Adu_accB2b_Aradu.CG4KR | 1 | 828 | Ad10 | 102159434 | 102165124 | 5691 | 7 |
| Aip_accB1_Araip.95C8Z | 1 | 837 | Ai04 | 123618248 | 123614361 | 3888 | 7 |
| Aip_accB2a_Araip.4I8QR | 1 | 828 | Ai10 | 128334014 | 128336668 | 2655 | 7 |
| Aip_accB2b_Araip.LLR5T | 1 | 867 | Ai04 | 128502086 | 128504860 | 2775 | 7 |
| Ath_accB1_AT5G16390 | 1 | 840 | At05 | 5363020 | 5361101 | 1920 | 7 |
| Ath_accB2_AT5G15530 | 1 | 765 | At05 | 5038955 | 5040434 | 1480 | 7 |
| Car_accB1_Ca_06111 | 1 | 642 | Ca03 | 24095637 | 24092310 | 3328 | 6 |
| Car_accB2a_Ca_22093 | 1 | 702 | Ca03 | 4876905 | 4874287 | 2619 | 6 |
| Car_accB2b_Ca_10464 | 1 | 846 | Ca02 | 3913812 | 3917762 | 3951 | 7 |
| Car_accB2c_Ca_21112 | 1 | 864 | Ca02 | 4249719 | 4254068 | 4350 | 7 |
| Cca_accB1_C.cajan_18238 | 1 | 783 | Cc07 | 9730402 | 9725113 | 5290 | 7 |
| Cca_accB2a_C.cajan_18788 | 1 | 861 | Cc07 | 15796216 | 15798492 | 2277 | 7 |
| Cca_accB2b_C.cajan_22132 | 1 | 795 | Cc09 | 315347 | 318108 | 2762 | 6 |
| Cca_accB2c_Scaffold132424 | 1 | 855 | Cc_Scaffold132424 | 25200 | 28010 | 2811 | 7 |
| Gma_accB1a_Glyma.09G248900 | 1 | 783 | Gm09 | 43806260 | 43810364 | 4105 | 7 |
| Gma_accB1b_Glyma.18G243500 | 1 | 786 | Gm18 | 57436721 | 57432151 | 4571 | 7 |
| Gma_accB2a_Glyma.13g057400 | 1 | 828 | Gm13 | 6372642 | 6377704 | 5063 | 7 |
| Gma_accB2b_Glyma.18g265300 | 1 | 852 | Gm18 | 59282825 | 59286085 | 3261 | 7 |
| Gma_accB2c_Glyma.19g028800 | 1 | 840 | Gm19 | 3547928 | 3551186 | 3259 | 7 |
| Lan_accB1_Lup015815 | 1 | 849 | NLL_Scaffold_287 | 545983 | 540039 | 5945 | 7 |
| Lan_accB2a_Lup032465 | 1 | 855 | NLL-05 | 1636861 | 1640279 | 3419 | 7 |
| Lan_accB2b_NLL05_2 | 1 | 693 | NLL-05 | 1638627 | 1640279 | 1653 | 6 |
| Lan_accB2c_Lup029955 | 1 | 873 | NLL-08 | 10948275 | 10951255 | 2981 | 7 |
| Lja_accB1_Lj1g3v2838150 | 1 | 690 | Lj1 | 32295983 | 32288521 | 7463 | 6 |
| Lja_accB2a_Lj1g3v3008790 | 1 | 867 | Lj1 | 34197127 | 34202605 | 5479 | 7 |
| Lja_accB2b_Lj2g3v1079560 | 1 | 852 | Lj2 | 17554992 | 17557103 | 2112 | 7 |
| Mtr_accB1_Medtr7g080290 | 1 | 828 | Mt07 | 30536157 | 30530316 | 5842 | 7 |
| Mtr_accB2a_Medtr6g015020 | 1 | 834 | Mt06 | 4863721 | 4866687 | 2967 | 7 |
| Mtr_accB2b_Medtr7g013100 | 1 | 861 | Mt07 | 3720674 | 3725155 | 4482 | 7 |
| Pvu_accB1_Phvul.008G053000 | 1 | 786 | Pv08 | 4711586 | 4715956 | 4371 | 7 |
| Pvu_accB2a_Phvul.004G034300 | 1 | 828 | Pv04 | 3798834 | 3795471 | 3364 | 7 |
| Pvu_accB2b_Phvul.004G035000 | 1 | 837 | Pv04 | 3851753 | 3855526 | 3774 | 7 |
| Vra_accB1_Vradi0363s00170 | 1 | 780 | Vr_scaffold_363 | 40535 | 36303 | 4233 | 7 |
| Vra_accB2a_Vradi01g07390 | 1 | 831 | Vr01 | 11817945 | 11814624 | 3322 | 7 |
| Vra_accB2b_Vradi01g07460 | 1 | 831 | Vr01 | 11889689 | 11893041 | 3353 | 7 |
| Adu_accC1_Aradu.ET2TE | 1 | 1620 | Ad05 | 88084520 | 88078638 | 5883 | 16 |
| Adu_accC2_Aradu.ZF3WE | 1 | 1620 | Ad03 | 99628915 | 99623645 | 5271 | 16 |
| Aip_accC1_Araip.KVI16 | 1 | 1581 | Ai05 | 140669721 | 140674834 | 5114 | 15 |
| Aip_accC2_Araip.Z9LG3 | 1 | 1530 | Ai03 | 85423627 | 85417933 | 5695 | 16 |
| Ath_accC_AT5G35360 | 1 | 1611 | At05 | 13584300 | 13588265 | 3966 | 16 |
| Car_accC_Ca_05874 | 1 | 1623 | Ca06 | 4279312 | 4286720 | 7409 | 16 |
| Cca_accC_C.cajan_23212 | 1 | 1560 | Cc05 | 1425351 | 1435006 | 9656 | 15 |
| Gma_accC1_Glyma.05G221100 | 1 | 1617 | Gm05 | 40282075 | 40289901 | 7827 | 16 |
| Gma_accC2_Glyma.08G027600 | 1 | 1617 | Gm08 | 2203747 | 2196664 | 7084 | 16 |
| Lan_accC1_Lup032034 | 1 | 1605 | NLL-06 | 11942343 | 11950164 | 7822 | 16 |
| Lan_accC2_Lup005680 | 1 | 1611 | NLL-17 | 19429254 | 19437240 | 7987 | 16 |
| Lja_accC_Lj4g3v3015020 | 1 | 1629 | Lj4 | 40303717 | 40296961 | 6757 | 15 |
| Mtr_accC_Medtr8g101330 | 1 | 1626 | Mt08 | 42540665 | 42532844 | 7822 | 16 |
| Pvu_accC_Phvul.002G302900 | 1 | 1587 | Pv02 | 46528564 | 46520124 | 8441 | 16 |
| Vra_accC_Vradi07g29170 | 1 | 1584 | Vr07 | 52899265 | 52907887 | 8623 | 16 |

# Table S6. Sequence coordinates for cytosolic ACCase and subunits of plastid ACCase genes derived from legume transcriptome assemblies

| **CDS** | **CDS start** | **CDS end** | **Transcript name** | **Transcript start** | **Transcript end** |
| --- | --- | --- | --- | --- | --- |
| Aar_ACC1_ZCDJ-2008558 | 1 | 6777 | ZCDJ-2008558 | 13 | 6789 |
| Aar_ACC2_ZCDJ-2008559 | 1 | 5277 | ZCDJ-2008559 | 36 | 5312 |
| Bto_ACC_JETM-2025864 | 1 | 6771 | JETM-2025864 | 7205 | 435 |
| Ccan_ACC_RKFX-2008745 | 1 | 6279 | RKFX-2008745 | 6849 | 571 |
| Cof_ACC_RKLL-2000979 | 1 | 6771 | RKLL-2000979 | 847 | 7712 |
| Gpo_ACC_VLNB-2076277 | 1 | 6426 | VLNB-2076277 | 6766 | 341 |
| Lal_ACC1_LAGI01_185 | 1 | 6729 | LAGI01_185 | 302 | 7030 |
| Lal_ACC2_LAGI01_188 | 1 | 6783 | LAGI01_188 | 7144 | 362 |
| Lal_ACC3_LAGI01_739 | 1 | 5628 | LAGI01_739 | 5633 | 6 |
| Lal_ACC4_LAGI01_948 | 1 | 5136 | LAGI01_948 | 387 | 5522 |
| Aar_accA_ZCDJ-2005513 | 1 | 2277 | ZCDJ-2005513 | 198 | 2474 |
| Bto_accA1_JETM-2027321 | 1 | 2187 | JETM-2027321 | 351 | 2537 |
| Bto_accA2_JETM-2010170 | 1 | 2097 | JETM-2010170 | 564 | 2716 |
| Ccan_accA_RKFX-2008617 | 1 | 2196 | RKFX-2008617 | 2 | 2256 |
| Cof_accA1_RKLL-2017992 | 1 | 2307 | RKLL-2017992 | 340 | 2646 |
| Cof_accA2_RKLL-2019542 | 1 | 2076 | RKLL-2019542 | 415 | 2543 |
| Gpo_accA_VLNB-2004340 | 1 | 2313 | VLNB-2004340 | 2667 | 355 |
| Lal_accA1_LAGI01_9432 | 1 | 2277 | LAGI01_9432 | 2740 | 464 |
| Lal_accA2_LAGI01_9477 | 1 | 2277 | LAGI01_9477 | 2737 | 461 |
| Lal_accA3_LAGI01_9523 | 1 | 2244 | LAGI01_9523 | 2732 | 464 |
| Lal_accA4_LAGI01_9668 | 1 | 2277 | LAGI01_9668 | 280 | 2556 |
| Lal_accA5_LAGI01_11229 | 1 | 2277 | LAGI01_11229 | 2568 | 292 |
| Aar_accB2a_ZCDJ-2015355 | 1 | 846 | ZCDJ-2015355 | 1109 | 264 |
| Aar_accB2b_ZCDJ-2015788 | 1 | 714 | ZCDJ-2015788 | 1174 | 461 |
| Bto_accB2_JETM-2006359 | 1 | 804 | JETM-2006359 | 1058 | 255 |
| Ccan_accB1_RKFX-2045617 | 1 | 840 | RKFX-2045617 | 208 | 1047 |
| Ccan_accB2_RKFX-2046821 | 1 | 864 | RKFX-2046821 | 300 | 1163 |
| Cof_accB2_RKLL-2007824 | 1 | 846 | RKLL-2007824 | 1105 | 260 |
| Gpo_accB1_VLNB-2074938 | 1 | 822 | VLNB-2074938 | 1016 | 195 |
| Gpo_accB2a_VLNB-2013644 | 1 | 774 | VLNB-2013644 | 339 | 1180 |
| Gpo_accB2b_VLNB-2013645 | 1 | 846 | VLNB-2013645 | 339 | 1184 |
| Gsi_accB1a_VHZV-2005994 | 1 | 771 | VHZV-2005994 | 186 | 1005 |
| Gsi_accB1b_VHZV-2005995 | 1 | 822 | VHZV-2005994 | 186 | 1005 |
| Gsi_accB2_VHZV-2015670 | 1 | 882 | VHZV-2015670 | 338 | 1219 |
| Lal_accB1a_LAGI01_36216 | 1 | 846 | LAGI01_36216 | 1426 | 476 |
| Lal_accB1b_LAGI01_41055 | 1 | 849 | LAGI01_41055 | 149 | 997 |
| Lal_accB1c_LAGI01_41651 | 1 | 849 | LAGI01_41651 | 86 | 934 |
| Lal_accB2a_LAGI01_35736 | 1 | 858 | LAGI01_35736 | 1253 | 396 |
| Lal_accB2b_LAGI01_37973 | 1 | 843 | LAGI01_37973 | 295 | 1137 |
| Lal_accB2c_LAGI01_40148 | 1 | 846 | LAGI01_40148 | 1099 | 254 |
| Lal_accB2d_LAGI01_40382 | 1 | 861 | LAGI01_40382 | 202 | 1062 |
| Lal_accB2e_LAGI01_44867 | 1 | 858 | LAGI01_44867 | 202 | 1059 |
| Aar_accC1_ZCDJ-2014388 | 1 | 1590 | ZCDJ-2014388 | 1890 | 301 |
| Aar_accC2_ZCDJ-2014389 | 1 | 1587 | ZCDJ-2014389 | 2543 | 957 |
| Bto_accC1_JETM-2008872 | 1 | 1590 | JETM-2008872 | 1914 | 325 |
| Bto_accC2_JETM-2008874 | 1 | 1590 | JETM-2008874 | 1914 | 325 |
| Ccan_accC_RKFX-2047844 | 1 | 1605 | RKFX-2047844 | 255 | 1859 |
| Cof_accC_RKLL-2067350 | 1 | 1605 | RKLL-2067350 | 382 | 1986 |
| Gpo_accC_VLNB-2076119 | 1 | 1617 | VLNB-2076119 | 180 | 1796 |
| Gsi_accC_VHZV-2021326 | 1 | 1605 | VHZV-2021326 | 169 | 1773 |
| Lal_accC1_LAGI01_20167 | 1 | 1602 | LAGI01_20167 | 1986 | 385 |
| Lal_accC2_LAGI01_21333 | 1 | 1629 | LAGI01_21333 | 2023 | 395 |
| Lal_accC3_LAGI01_21523 | 1 | 1629 | LAGI01_21523 | 2013 | 385 |

# Table S7. Total score values calculated for legume *ACC* syntenic regions

| **Syntenic region** | **Homolog presence** | **Homolog accessions** | **Total score for *ACC1* region (NLL-14)** | **Total score for *ACC2* region (NLL-15)** |
| --- | --- | --- | --- | --- |
| Ad08|12.45 | yes | Aradu.GHK20 | 22 807 | 24 236 |
| Ai07|120.60 | yes | Araip.ZAK9V | 23 244 | 25 504 |
| Ca05|29.28 | yes | Ca_16623 | 15 333 | 19 406 |
| Cascaffold1301_1 | no | - | 10 020 | 8 575 |
| Cc02|05.53 | no | - | 4 267 | 6 605 |
| Cc03|01.54/9 | no | - | 10 343 | 8 563 |
| Ccscaff332|00.38 | yes | C.cajan_25715 | 17 160 | 21 809 |
| Ccscaff126149 | no | - | 4 706 | 2 089 |
| Gm13|19.57 | no | - | 7 944 | 7 759 |
| Gm14|42.00 | no | - | 6 926 | 6 777 |
| Gm04|10.06 | yes | Glyma.04G104900 | 17 664 | 25 647 |
| Gm04|52.10 | no | - | 6 684 | 0 |
| Gm06|08.48 | yes | Glyma.06G105900 | 28 433 | 30 198 |
| Gm06|45.12 | fragment | Glyma.06G263800, truncated | 503 | 499 |
| Gm07|16.30 | fragment | Glyma.07G137400, truncated | 944 | 1 004 |
| Gm10|25.43 | fragment | Glyma.10G109600, truncated | 906 | 1 072 |
| Lj0|139.24 | yes | Lj0g3v0268829.1 | n/a | n/a |
| Lj01|08.27 | no | - | 8 919 | 2 975 |
| Lj01|55.87 | yes | Lj1g3v4694390, Lj1g3v4699650 | 2 316 | 2 344 |
| Mt03|33.32 | yes | Medtr3g073820, Medtr3g073860 | 31 795 | 33 820 |
| Mt05|25.49 | no | - | 5 648 | 9 474 |
| Pv08|43.95 | no | - | 8 253 | 7 640 |
| Pv09|06.36 | yes | Phvul.009G028700 | 25 649 | 27 681 |
| Vr05|15.27/19.58 | yes | Vradi05g07690 | 25 737 | 26 838 |
| Vr06|12.00 | no | - | 3 196 | 5 196 |

# Table S8. Total score values calculated for legume *accA* syntenic regions

| **Syntenic region** | **Homolog presence** | **Homolog accessions** | **Total score for *accA1* region (NLL-13)** | **Total score for *accA2* region (NLL-11)** | **Total score for *accA3* region (NLL-07)** |
| --- | --- | --- | --- | --- | --- |
| Ad02|78.09 | no | - | 5 010 | 3 684 | 6 101 |
| Ad04|75.81/87.67 | -/yes | Aradu.14CMN | 8 326 | 7 205 | 13 626 |
| Ad04|109.435 | yes | Aradu.Z0G82 | 246 | 360 | 339 |
| Ai02|89.44 | no | - | 3 725 | 3 144 | 7 817 |
| Ai04|81.67/96.00 | -/yes | Araip.7H2NS | 9 676 | 8 479 | 13 727 |
| Ai04|118.072 | yes | Araip.77JRH | 252 | 355 | 345 |
| Ca03|18.55 | no | - | 5 865 | 6 787 | 8 058 |
| Cascaff545 | no | - | 5 454 | 2 161 | 5 567 |
| Cascaff561 | yes | Ca_26217 | 1 562 | 3 069 | 4 207 |
| Ccscaff127360 | yes | C.cajan_33027 | 4 671 | 5 108 | 9 984 |
| ccLG07|12.710 | yes | C.cajan_18479 | 220 | 310 | 235 |
| Gm01|36.00 | no | - | 5 104 | 3 904 | 7 198 |
| Gm18|47.02 | yes | Glyma.18G195700, Glyma.18G195900, Glyma.18G196000 | 16 771 | 15 107 | 18 093 |
| Gm07|17.20 | no | - | 12 294 | 9 907 | 18 823 |
| Lj0|133.34 | yes | Lj0g3v0256879.1 | n/a | n/a | n/a |
| Lj01|27.0/39.7 | yes | Lj1g3v2628750 | 9 131 | 4 510 | 4 498 |
| Mt04|06.21 | no | - | 4 276 | 3 718 | 6 212 |
| Mt07|24.40 | yes | Medtr7g066870 | 10 119 | 10 705 | 15 720 |
| Pv08|12.63 | yes | Phvul.008G108700 | 11 841 | 9 906 | 15 281 |
| Vr02|07.76 | no | - | 0 | 3 014 | 0 |
| Vr06|24.90 | yes | Vradi06g10650 | 2 635 | 3 078 | 3 266 |
| Vr07|0.01 | fragment | truncated | 1 088 | 976 | 1 013 |
| Vr08|8.08 | no | - | 2 498 | 4 246 | 6 898 |
| Vr09|12.04 | no | - | 3 002 | 2 018 | 3 181 |
| Vrscaff251 | no | - | 2 910 | 1 698 | 1 316 |
| Vrscaff392 | no | - | 1 275 | 260 | 0 |

# Table S9. Total score values calculated for legume *accB* syntenic regions

| **Syntenic region** | **Homolog presence** | **Homolog accessions** | **Total score for *accB1* region (Scaff287)** | **Total score for *accB2a/b* region (NLL05)** | **Total score for *accB2c* region (NLL08)** |
| --- | --- | --- | --- | --- | --- |
| Ad04|113.60 | yes | Aradu.JDP66 | 2 592 | 0 | 0 |
| Ad09|72.31 | yes | Aradu.CP1HR truncated | 99 | 0 | 0 |
| Ai04|123.61 | yes | Araip.95C8Z | 2 310 | 0 | 0 |
| Ai09|88.11 | yes | Araip.I6HDR truncated | 99 | 0 | 0 |
| Ca3|24.09 | yes | Ca_06111 | 2 896 | 0 | 0 |
| Cc07|09.73 | yes | C.cajan_18238 | 2 937 | 0 | 0 |
| Gm18|53.16 | yes | Glyma.18G243500 | 2 811 | 0 | 0 |
| Gm9|47.00 | yes | Glyma.09G248900 | 3 077 | 0 | 0 |
| Lj01|32.29 | yes | Lj1g3v2838150 | 2 695 | 0 | 0 |
| Mt07|30.53 | yes | Medtr7g080290 | 2 895 | 0 | 0 |
| Pv08|04.69 | yes | Phvul.008G053000 | 2 612 | 0 | 0 |
| Vrscaff363 | yes | Vradi0363s00170 | 2 715 | 0 | 0 |
| Ad04|118.54 | yes | Aradu.0JT6M | 0 | 4 440 | 24 788 |
| Ad10|102.16 | yes | Aradu.CG4KR | 0 | 15 112 | 18 958 |
| Ai04|128.50 | yes | Araip.LLR5T | 0 | 4 815 | 25 187 |
| Ai10|128.33 | yes | Araip.4I8QR | 0 | 16 187 | 19 077 |
| Ca2|3.91/4.25 | yes | Ca_10464, Ca_21112 | 0 | 17 782 | 18 639 |
| Ca3|04.87 | yes | Ca_22093 | 0 | 2 751 | 22 978 |
| Cc07|15.79 | yes | C.cajan_18788 | 0 | 4 085 | 8 882 |
| Cc09|00.31 | yes | C.cajan_22132 | 0 | 13 692 | 11 955 |
| Ccscaffold113408 | yes | C.cajan_48468 fragment | 0 | 0 | 0 |
| Ccscaffold132424 | yes | new gene truncated | 182 | 2 894 | 1 378 |
| Gm13|15.50 | yes | Glyma.13G057400 | 0 | 19 957 | 20 657 |
| Gm18|55.01 | yes | Glyma.18G265300 | 0 | 4 781 | 27 160 |
| Gm19|03.58 | yes | Glyma.19G028800 | 206 | 18 672 | 22 385 |
| Gm8|21.00 | no |  | 0 | 4 545 | 26 267 |
| Lj01|34.20 | yes | Lj1g3v3008790 | 0 | 4 860 | 8 333 |
| Lj02|17.55 | yes | Lj2g3v1079560 | 0 | 18 442 | 24 668 |
| Mt06|04.82 | yes | Medtr6g014880 truncated, Medtr6g015020 | 0 | 14 256 | 22 477 |
| Mt07|03.72 | yes | Medtr7g013100 | 0 | 3 391 | 21 772 |
| Pv04|03.79 | yes | Phvul.004G034300, Phvul.004G035000 | 0 | 17 241 | 23 348 |
| Vr01|11.82 | yes | Vradi01g07390, Vradi01g07460 | 87 | 16 202 | 18 131 |
| Vr04|18.20 | no |  | 0 | 3 149 | 24 778 |

# Table S10. Total score values calculated for legume *accC* syntenic regions

| **Syntenic region** | **Homolog presence** | **Homolog accessions** | **Total score for *accC1* region (NLL-06)** | **Total score for *accC2* region (NLL-17)** |
| --- | --- | --- | --- | --- |
| Ad03|60.31/86.71/99.62 | yes | Aradu.ZF3WE | 19 542 | 9 242 |
| Ad05|88.08 | yes | Aradu.ET2TE | 5 313 | 3 880 |
| Ai03|85.41 | yes | Araip.Z9LG3 | 19 633 | 9 016 |
| Ai05|132.70 | fragment | Araip.W3HRA truncated | 150 | 150 |
| Ai05|140.67 | yes | Araip.KVI16 | 5 491 | 3 845 |
| Ca06|04.28 | yes | Ca_05874 | 22 324 | 10 513 |
| Cc05|01.43 | yes | C.cajan_23212 | 20 196 | 11 518 |
| Ccscaff131698 | no |  | 6 630 | 0 |
| Gm05|40.04 | yes | Glyma.05G221100 | 24 816 | 12 215 |
| Gm08|02.18 | yes | Glyma.08G027600 | 23 611 | 12 247 |
| Lj04|40.29 | yes | Lj4g3v3015020 | 23 615 | 10 662 |
| Mt08|42.52 | yes | Medtr8g101330 | 23 579 | 12 675 |
| Pv02|46.52 | yes | Phvul.002G302900 | 22 509 | 11 074 |
| Vr07|52.91 | yes | Vradi07g29170 | 21 813 | 10 432 |

# Table S11. FASTA alignment of *ACC* sequences used for phylogenetic inference

>Aar_ACC1_ZCDJ‑2008558

ATGGCGGGCACTGGGCACGGGAAT‑‑‑‑‑‑‑‑‑GGATTCATAAATGGTATACCACCAAATAGGCATTCTGCTACAATCTCTGTAGTAGACGACTTCTGCCACGCCCTTGGGGGAAAGAGGCCAATTCACAGCATACTAATTGCAAACAATGGAATGGCAGCAGTCAAGTTTATACGTAGTGTTAGGACCTGGGCCTATGAGACATTTGGCACGGAGAAGGCCATCTTGTTGGTTGCCATGGCTACTCCTGAGGATATGAGAATCAACGCAGAACACATCAGAATGGCCGATCAATTTGTTGAAGTTCCTGGTGGAACCAATAACAATAACTATGCCAACGTACAACTTATTGTTGAG‑‑‑‑‑‑‑‑‑GTGGCTGAGATAACCCATGTTGAAGCAGTGTGGCCTGGTTGGGGTCATGCATCAGAGAATCCTGAACTGCCAGATGCTTTAAATGCAAAAGGAATTGTCTTTCTTGGACCCCCTTCTGTATCTATGGCAGCATTAGGAGATAAAATTGGTTCGTCATTGATTGCTCAAGCAGCAGATGTGCCAACCCTTCCATGGAGTGGTTCTCATGTGAAAATTCCCCCTGCGAATTGTTTAATTACCATTCCTGATGATATTTACCGACAAGCATGTGTTTATACAACAGAGGAAGCGATTGCTAGCTGTCAAGTTGTAGGTTACCCTGCAATGATTAAGGCCTCCTGGGGTGGTGGTGGTAAAGGCATAAGAAAGGTTCATAATGATGATGAGGTCAGGGCATTGTTCAAGCAAGTTCAAGGGGAAGTTCCAGGTTCTCCTATATTCATAATGAAGTTAGCATCCCAGAGCCGACATTTGGAAGTCCAGTTACTTTGTGATCAGTATGGAAATGTTGCTGCTTTGCATAGTCGTGATTGCAGTATTCAAAGGAGGCACCAAAAAATCATTGAAGAGGGACCGATTACTGTAGCTCCTCTGGATACAGTGAAAAAACTGGAACAGGCAGCTAGAAGATTGGCTAAAAGCGTAAACTACATTGGTGCAGCTACAGTTGAGTATCTATTCAGCATGGAGACTGGCGAGTACTACTTTTTGGAGCTGAACCCTCGATTACAGGTTGAACATCCTGTTACTGAGTGGATAGCAGAGATAAATCTGCCGGCAGCACAAGTTGTTGTTGGGATGGGCATTCCTCTCTGGCAAATTCCTGAGATAAGGCGTTTCTATGGGGTGGAACATGGTGGGGGTTATGATGCTTGGAGGAAAACAGCAGCTTTAGCCACTCCTTTTGGTTTTGACGAAGCTGAATCTACAAGGCCAAAAGGTCATTGTGTGGCTGTACGGGTGACCAGTGAGGACCCTGATGATGGGTTTAAACCTACGAGTGGGAAAGTGCTGGAGCTGAGCTTTAAAAGTAAGCCAAATGTGTGGGCATACTTCTCTGTTAAGTCCGGAGGAGGAATTCATGAATTCTCAGATTCTCAGTTTGGGCATGTTTTTGCATTTGGAGAATCTAGGGCTTTAGCAATTGCAAATATGGTTCTTGGGCTGAAGGAGGTTCAGATTCGGGGAGAAATTCGTACCAATGTTGACTATACTATAGATCTTTTAAATGCCTCAGACTACAGAGACAACAAAATCCACACGGGATGGTTGGACAGTAGAATTGCAATGAGGGTTAGAGCAGAAAGGCCACCTTGGCATCTTTCTGTTGTTGGAGGGGCACTCTATAAAGCCTCTGCTAGCAGTGCAGCTTTGGTTTCAGACTATGTTGGCTATCTTGAAAAGGGACAAATCCCTCCAAAGCACATATCTCTTGTCCACTCTCAAGTGTCTTTGAACATTGAAGGAAGCAAATATACGATTGACATGGTAGGAGGAGGACCAGGAAGATATAGATTGAGGATGAATGAATCAGAAATAGAAGCAGAGATACATACTTTACGGGATGGAGGTTTGCTAATGCAG‑‑‑‑‑‑‑‑‑TTGGATGGAAACAGTCATGTGATATATGCAGAGGAAGAGGCAGCTGGAACTCGTCTTCTAATAGATGGAAGAACTTGCTTGCTTCAGAATGATCATGATCCATCGAAACTAGTTGCAGAGACACCGTGCAAGCTTCTGAGATATTTGATTACAGATGATAGTCATATTGATGCAGACTCACCATATGCAGAGGTTGAGGTCATGAAGATGTGTATGCCTCTTCTCTCACCTGCTTCTGGCGTTATTCATTTTAAAATGACTGAAGGTCAAGCAATGCAGACTGGTGAACTTATAGCAAGGCTTGATTTAGACGATCCTTCAGCTGTAAGAAAAGCAGAACCTTTTCATGGGAGCCTCCCAGTTTTGGGTCCTCCAACTGCAATTTCTGGCAAAGTTCATCAGAAATGTGCTGCAAGTTTAAATGCAGCACGCATGATTCTGGCTGGCTATGAGCATAATATTGAGGAAGTTATGCAAAGTTTACTCTATTGCCTTGATAGTCCTGAATTGCCTTTCCTTCAATGGCAAGAGTGCCTGGCTGTTTTGGTGACTCGTCTTCCCAAAGATCTCAAAAATGAGTTGGAATCAAAATTCAAAGAGTTTGAGAGGATTTCA‑‑‑AACTCCCAAAATGTTGATTTCCCCGCCAAGTTATTGAAGGGAATCCTTGATGCTCATCTTTCCTCCTGTCCTGACAAAGAAAAAGGAGCCCAAGAAAGGCTTGTTGAACCTCTGATGAGTCTGGTGAAGTCTTATGAGGGGGGACGAGAGAGCCATGCTCGTATTATTGTTCAATCCCTTTTTGAGGAGTATCTTTCTGTTGAAGAACTCTTTAGTGATAACATACAGGCTGATGTAATTGAACGCCTCCGTCTCCAATACAAGAAAGATCTACTTAAGATTGTGGATATTGTGCTCTCTCACCAGGGTGTCAAAAGTAAAAATAAACTCATACTGCGATTAATGGAACAACTGGTTTACCCAAACCCTGCGGCCTACAGGGATCAACTGATCCGTTTCTCTGCACTCAACCATACAAATTATTCTCAGTTGGCTCTTAAGGCTAGTCAATTACTGGAGCAAACTAAATTGAGTGAACTTCGTTCTAGCATTGCTAGAAGTCTTTCGGAACTAGAGATGTTCACTGAGGATGGTGAAAATATTGACACTCCCAGGAGGAAGAGTGCCATTAATGATCGAATGGAGGACCTTGTGAGTGCTCCCTTGGCAGTTGAAGATGCCCTTGTAGGTTTGTTTGATCACAGTGATCATACTCTTCAAAGGAGGGTTGTGGAAACTTATATTCGCAGGCTGTACCAGCCATATCTTGTCAAAGGGAGTGTTAGGATGCAGTGGCACAGATCTGGTCTTATTGCTTCATGGGAGTTCTTAGAAGAGTATGTCGAAAGCAAGAGT‑‑‑GGAACTGAAGAC‑‑‑CAAATGTCTGATAAAGCATCGTTGGAGAAACATAGCAAGAGAAAATGGGGAGCGATGGCTATAATTAAATCTCTTCAATTTTTGCCTGCGATAATCAGTGCTGCAATAAGGGAAGCAACCCATAACTTTCATGGAGCA‑‑‑CTTCCAAATAGTTCTGCTGAACTGATTGACTATGGCAATATGATGCATATTGCATTGGCAGGCATCAACAATCAGATGAGTTTACTGCAAGACAGTGGTGATGAGGATCAGGCTCAAGAAAGGATCAATAAGTTAGCCAAAATACTTAAGGAAAAGGACGTAGGCTCCACTATACATGCTGCAGGTGTGGGAGTAATTAATTGTATCATACAGAGGGATGAAGGTCGAGCCCCCATGAGGCACTCCTTTTACTGGTCAGCAGAAAATCTCTATTATGAGGAGGAGCCCTTGTTGCGTCATCTAGAACCTCCCCTATCCATTTATCTTGAATTGGACAAACTTAAAGGATATGATAATATACGGTATACTCCATCCCGAGATCGTCAGTGGCACTTGTACACAGTTCTAGATCATAAGCCTCAACCGATTCAAAGAATGTTTCTCCGAACACTTGTAAGGCAGCCAGTTACAAGTGATGGATTCTCTTCATATCAAGGCCTAGATGCAGAAATATCCCGTGCCCAATTGACTATGCCTTTCACTTCAAGGAGCATTTTGAGGTCCTTGCTGACTGCAATGGAGGAGTTGGAACTTAATGCACACAATGCCACTGTCAGACCTGAACATGCTCATATGTACCTCTATATCATACGCGAGCAACAAATTAGTGATCTTTTGCCCTACACAAAGAGAATTGACATAAATGCTGACCAAGAAGAAACAGCAGTTGAGGCAATTTTGGAAGAACTGCCACGTGAAATCCATTCTTTTGTTGGTGTAAGAATGCATAGATTAGGTGTTGCTGAGTGGGAAGTCAAACTCTGGATGGCATCTTCTGGACCAGCAAATGGTGCTTGGAGGATTATTGTAACTAATGTGACTGGTCATACATGCACTGTACATATATACCGAGAACTGGAGGATACCAGCACCCACAAAGTGATATACAGTTCAGTCACTGTAAAGGGTCCTCTGCATGGTGTACCAGTGAATGAAAACTATCGACCTTTGGGAGTCATTGACCGAAAACGTCTTGTAGCAAGGAAGAACAGCACCACTTACTGCTATGATTTTCCACTGGCATTTGAAACAGCATTGGAGCAATCATGGACAAACCAATACCCAGGATTTCAAAAGGCTAAAGATAAA‑‑‑GTTCTAAAAGTAACAGAGCTAAAGTTTGCTGACAAGGAGGGCAATTGGGGTACTCCTCTTGTTCCTGTGGAGCATCCCCTTGGACTCAATGACGTAGGCATGGTAGCCTGGTTAATGGAAATGTGTACTCCTGAGTTCCCAAATGGAAGGAAAATCTTAGTTGTAGCAAATGACGTGACTTTTAAGGCAGGTTCTTTTGGTCCAAGGGAGGATGCCTTCTTTCATGCAGTAACTGACCTTGCGTGTAAGAAAAGATTGCCATTGATTTACCTAGCAGCAAATTCTGGTGCCCGTTTAGGCGTAGCTGAGGAAGTCAAAGCTTGTTTCAGAGTTGGTTGGTCTGATGAATCTAACCCTGAGCATGGTTTGCAGTATGTATACTTAACACCTGAGGATTATGGTAGGATCAAATCATCTGTAATAGCGCACGAGTTGAAGCTGGAAAGTGGAGAAACCAGATGGATAATTGATAGCATTGTTGGGAAAGAGGATGGTCTAGGGGTTGAAAACCTAAGTGGCAGTGGAGCTATTGCTAGTGCCTATTCAAGGGCATATAAGGAAACTTTCACCTTGACATACGTGACCGGAAGAACAGTGGGAATAGGGGCTTATCTTGCTAGGCTGGGAATGAGGTGCATACAGAGGCTTGATCAGCCCATTATTCTTACTGGTTTTTCTGCATTGAACAAACTCCTTGGTCGGGAGGTATACAGTTCTCACATGCAACTTGGTGGGCCTAAGATCATGGCAACAAATGGAGTTGTTCATCTTACTGTTTCAGATGATCTTGAAGGTGTTTCTGCTATTTTGAGGTGGCTCAGCTACATTCCTTCTCATGTAGGTGGTACACTTCCAATTGTACAACCCCATGATCCTCCAGAAAGACCAGTGGAGTATTTCCCAGAGAACTCGTGTGACCCTCGTGCTGCCATATGTGGTTCCGTGGATAGTAATGGAAAATGGCTGGGTGGAATTTTTGACAAGGAAAGCTTTGTGGAGACGCTAGATGGATGGGCCAGGACAGTTGTTACAGGAAGGGCAAAGCTTGGAGGAATCCCTGTGGGAATTGTTGCTGTTGAGACGCAGACTGTGATGCAAGTTATACCAGCTGATCCAGGTCAGCTTGATTCTCATGAAAGGGTGGTTCCTCAGGCAGGGCAGGTCTGGTTTCCTGATTCTGCAACTAAAACAGCCCAGTCAATACTAGATTTCAACAGAGAAGAGCTCCCACTTTTCATTCTTGCAAATTGGAGAGGTTTTTCAGGTGGGCAGAGGGACCTTTTTGAAGGAATTCTTCAAGCTGGATCAACCATTGTGGAGAACCTTCGAACATACAAGCAGCCTGTATTTGTATATATCCCAATGATGGGTGAACTCCGTGGTGGGGCATGGGTTGTTGTAGACAGCCGAATTAATCCAGACCACATTGAAATGTATGCTGATCGCACAGCTAAAGGGAATGTCCTTGAGCCAGAAGGGATGATAGAGATCAAATTTAGGACAAAGGAATTATTGGAGTGCATGGGTAGACTTGACCAGCAGCTGATAAACCTCAAAGCAAAATTACAGGAAGCCAAGAGTAATAGGGCTATCGGGACCATTGATTCCCTGCAGCAGCAGATTAAATCCCGGGAGAAACAGCTTTTGCCTCTGTATACGCAGATAGCTATCAAATTTGCAGAATTACATGATACCTCCCTAAGGATGGCCGCAAAGGGGGTAATAAGAGAAGTTCTGGACTGGCGTAACTCTCGCTCTTTCCTGTACCGCAGACTACGCAGGAGAATTGGTGAACATTCACTGATAAACAGTGTCAGAGATGCTGCT‑‑‑GGTGACCATATGTCTAATATATCTGCAATAGAGTTGATAAAAAATTGGTATTTGAATTCTGATATTGCCAAAGGGAGAGATGATGCTTGGTTGGATGATGAGGCTTTCTTCACATGGAAGAAAGAGCAGCAGAATTACGAGGATAAATTAAAGGAATTGCGTGCCCAAAAGGTGCTGCTTCAACTGACAAATATTGGCGATTCAGTTTTGGACTTGGAAGCTTTACCTCAAGGTCTTGCTGCTCTTTTAAGCAAGTTGGAACCATCAAACCGAGCGAAATTGATTGATGAGCTCCGGAAGGTACTTGGT‑‑‑

>Aar_ACC2_ZCDJ‑2008559

‑‑‑‑‑‑‑‑‑‑‑‑‑‑‑‑‑‑‑‑‑‑‑‑‑‑‑‑‑‑‑‑‑‑‑‑‑‑‑‑‑‑‑‑‑‑‑‑‑‑‑‑‑‑‑‑‑‑‑‑‑‑‑‑‑‑‑‑‑‑‑‑‑‑‑‑‑‑‑‑‑‑‑‑‑‑‑‑‑‑‑‑‑‑‑‑‑‑‑‑‑‑‑‑‑‑‑‑‑‑‑‑‑‑‑‑‑‑‑‑‑‑‑‑‑‑‑‑‑‑‑‑‑‑‑‑‑‑‑‑‑‑‑‑‑‑‑‑‑‑‑‑‑‑‑‑‑‑‑‑‑‑‑‑‑‑‑‑‑‑‑‑‑‑‑‑‑‑‑‑‑‑‑‑‑‑‑‑‑‑‑‑‑‑‑‑‑‑‑‑‑‑‑‑‑‑‑‑‑‑‑‑‑‑‑‑‑‑‑‑‑‑‑‑‑‑‑‑‑‑‑‑‑‑‑‑‑‑‑‑‑‑‑‑‑‑‑‑‑‑‑‑‑‑‑‑‑‑‑‑‑‑‑‑‑‑‑‑‑‑‑‑‑‑‑‑‑‑‑‑‑‑‑‑‑‑‑‑‑‑‑‑‑‑‑‑‑‑‑‑‑‑‑‑‑‑‑‑‑‑‑‑‑‑‑‑‑‑‑‑‑‑‑‑‑‑‑‑‑‑‑‑‑‑‑‑‑‑‑‑‑‑‑‑‑‑‑‑‑‑‑‑‑‑‑‑‑‑‑‑‑‑‑‑‑‑‑‑‑‑‑‑‑‑‑‑‑‑‑‑‑‑‑‑‑‑‑‑‑‑‑‑‑‑‑‑‑‑‑‑‑‑‑‑‑‑‑‑‑‑‑‑‑‑‑‑‑‑‑‑‑‑‑‑‑‑‑‑‑‑‑‑‑‑‑‑‑‑‑‑‑‑‑‑‑‑‑‑‑‑‑‑‑‑‑‑‑‑‑‑‑‑‑‑‑‑‑‑‑‑‑‑‑‑‑‑‑‑‑‑‑‑‑‑‑‑‑‑‑‑‑‑‑‑‑‑‑‑‑‑‑‑‑‑‑‑‑‑‑‑‑‑‑‑‑‑‑‑‑‑‑‑‑‑‑‑‑‑‑‑‑‑‑‑‑‑‑‑‑‑‑‑‑‑‑‑‑‑‑‑‑‑‑‑‑‑‑‑‑‑‑‑‑‑‑‑‑‑‑‑‑‑‑‑‑‑‑‑‑‑‑‑‑‑‑‑‑‑‑‑‑‑‑‑‑‑‑‑‑‑‑‑‑‑‑‑‑‑‑‑‑‑‑‑‑‑‑‑‑‑‑‑‑‑‑‑‑‑‑‑‑‑‑‑‑‑‑‑‑‑‑‑‑‑‑‑‑‑‑‑‑‑‑‑‑‑‑‑‑‑‑‑‑‑‑‑‑‑‑‑‑‑‑‑‑‑‑‑‑‑‑‑‑‑‑‑‑‑‑‑‑‑‑‑‑‑‑‑‑‑‑‑‑‑‑‑‑‑‑‑‑‑‑‑‑‑‑‑‑‑‑‑‑‑‑‑‑‑‑‑‑‑‑‑‑‑‑‑‑‑‑‑‑‑‑‑‑‑‑‑‑‑‑‑‑‑‑‑‑‑‑‑‑‑‑‑‑‑‑‑‑‑‑‑‑‑‑‑‑‑‑‑‑‑‑‑‑‑‑‑‑‑‑‑‑‑‑‑‑‑‑‑‑‑‑‑‑‑‑‑‑‑‑‑‑‑‑‑‑‑‑‑‑‑‑‑‑‑‑‑‑‑‑‑‑‑‑‑‑‑‑‑‑‑‑‑‑‑‑‑‑‑‑‑‑‑‑‑‑‑‑‑‑‑‑‑‑‑‑‑‑‑‑‑‑‑‑‑‑‑‑‑‑‑‑‑‑‑‑‑‑‑‑‑‑‑‑‑‑‑‑‑‑‑‑‑‑‑‑‑‑‑‑‑‑‑‑‑‑‑‑‑‑‑‑‑‑‑‑‑‑‑‑‑‑‑‑‑‑‑‑‑‑‑‑‑‑‑‑‑‑‑‑‑‑‑‑‑‑‑‑‑‑‑‑‑‑‑‑‑‑‑‑‑‑‑‑‑‑‑‑‑‑‑‑‑‑‑‑‑‑‑‑‑‑‑‑‑‑‑‑‑‑‑‑‑‑‑‑‑‑‑‑‑‑‑‑‑‑‑‑‑‑‑‑‑‑‑‑‑‑‑‑‑‑‑‑‑‑‑‑‑‑‑‑‑‑‑‑‑‑‑‑‑‑‑‑‑‑‑‑‑‑‑‑‑‑‑‑‑‑‑‑‑‑‑‑‑‑‑‑‑‑‑‑‑‑‑‑‑‑‑‑‑‑‑‑‑‑‑‑‑‑‑‑‑‑‑‑‑‑‑‑‑‑‑‑‑‑‑‑‑‑‑‑‑‑‑‑‑‑‑‑‑‑‑‑‑‑‑‑‑‑‑‑‑‑‑‑‑‑‑‑‑‑‑‑‑‑‑‑‑‑‑‑‑‑‑‑‑‑‑‑‑‑‑‑‑‑‑‑‑‑‑‑‑‑‑‑‑‑‑‑‑‑‑‑‑‑‑‑‑‑‑‑‑‑‑‑‑‑‑‑‑‑‑‑‑‑‑‑‑‑‑‑‑‑‑‑‑‑‑‑‑‑‑‑‑‑‑‑‑‑‑‑‑‑‑‑‑‑‑‑‑‑‑‑‑‑‑‑‑‑‑‑‑‑‑‑‑‑‑‑‑‑‑‑‑‑‑‑‑‑‑‑‑‑‑‑‑‑‑‑‑‑‑‑‑‑‑‑‑‑‑‑‑‑‑‑‑‑‑‑‑‑‑‑‑‑‑‑‑‑‑‑‑‑‑‑‑‑‑‑‑‑‑‑‑‑‑‑‑‑‑‑‑‑‑‑‑‑‑‑‑‑‑‑‑‑‑‑‑‑‑‑‑‑‑‑‑‑‑‑‑‑‑‑‑‑‑‑‑‑‑‑‑‑‑‑‑‑‑‑‑‑‑‑‑‑‑‑‑‑‑‑‑‑‑‑‑‑‑‑‑‑‑‑‑‑‑‑‑‑‑‑‑‑‑‑‑‑‑‑‑‑‑‑‑‑‑‑‑‑‑‑‑‑‑‑‑‑‑‑‑‑‑‑‑‑‑‑‑‑‑‑‑‑‑‑‑‑‑‑‑‑‑‑‑‑‑‑‑‑‑‑‑‑‑‑‑‑‑‑‑‑‑‑‑‑‑‑‑‑‑‑‑‑‑‑‑‑‑‑‑‑‑‑‑ATGGTTCTAGGATTGAAGGAAATTCAAATTCGAGGAGAAATTCGCACGAATGTTGACTATACCATTGATCTTCTAAATGCTTCAGACTACAGAGAAAATAAAATTCATACAGGATGGTTGGACAGTAGAATTGCAATGAGGGTTAGAGCAGAAAGGCCACCTTGGCATCTTTCTGTTGTTGGAGGGGCACTCTATAAAGCCTCTGCTAGCAGTGCAGCTTTGGTTTCAGACTATGTTGGCTATCTTGAAAAGGGACAAATCCCTCCAAAGCACATATCTCTTGTCCACTCTCAAGTGTCTTTGAACATTGAAGGAAGCAAATATACGATTGACATGGTAGGAGGAGGACCAGGAAGATATAGATTGAGGATGAATGAATCAGAAATAGAAGCAGAGATACATACTTTACGGGATGGAGGTTTGCTAATGCAG‑‑‑‑‑‑‑‑‑TTGGATGGAAACAGTCATGTGATATATGCAGAGGAAGAGGCAGCTGGAACTCGTCTTCTAATAGATGGAAGAACTTGCTTGCTTCAGAATGATCATGATCCATCGAAACTAGTTGCAGAGACACCGTGCAAGCTTCTGAGATATTTGATTACAGATGATAGTCATATTGATGCAGACTCACCATATGCAGAGGTTGAGGTCATGAAGATGTGTATGCCTCTTCTCTCACCTGCTTCTGGCGTTATTCATTTTAAAATGACTGAAGGTCAAGCAATGCAGACTGGTGAACTTATAGCAAGGCTTGATTTAGACGATCCTTCAGCTGTAAGAAAAGCAGAACCTTTTCATGGGAGCCTCCCAGTTTTGGGTCCTCCAACTGCAATTTCTGGCAAAGTTCATCAGAAATGTGCTGCAAGTTTAAATGCAGCACGCATGATTCTGGCTGGCTATGAGCATAATATTGAGGAAGTTATGCAAAGTTTACTCTATTGCCTTGATAGTCCTGAATTGCCTTTCCTTCAATGGCAAGAGTGCCTGGCTGTTTTGGTGACTCGTCTTCCCAAAGATCTCAAAAATGAGTTGGAATCAAAATTCAAAGAGTTTGAGAGGATTTCA‑‑‑AACTCCCAAAATGTTGATTTCCCCGCCAAGTTATTGAAGGGAATCCTTGATGCTCATCTTTCCTCCTGTCCTGACAAAGAAAAAGGAGCCCAAGAAAGGCTTGTTGAACCTCTGATGAGTCTGGTGAAGTCTTATGAGGGGGGACGAGAGAGCCATGCTCGTATTATTGTTCAATCCCTTTTTGAGGAGTATCTTTCTGTTGAAGAACTCTTTAGTGATAACATACAGGCTGATGTAATTGAACGCCTCCGTCTCCAATACAAGAAAGATCTACTTAAGATTGTGGATATTGTGCTCTCTCACCAGGGTGTCAAAAGTAAAAATAAACTCATACTGCGATTAATGGAACAACTGGTTTACCCAAACCCTGCGGCCTACAGGGATCAACTGATCCGTTTCTCTGCACTCAACCATACAAATTATTCTCAGTTGGCTCTTAAGGCTAGTCAATTACTGGAGCAAACTAAATTGAGTGAACTTCGTTCTAGCATTGCTAGAAGTCTTTCGGAACTAGAGATGTTCACTGAGGATGGTGAAAATATTGACACTCCCAGGAGGAAGAGTGCCATTAATGATCGAATGGAGGACCTTGTGAGTGCTCCCTTGGCAGTTGAAGATGCCCTTGTAGGTTTGTTTGATCACAGTGATCATACTCTTCAAAGGAGGGTTGTGGAAACTTATATTCGCAGGCTGTACCAGCCATATCTTGTCAAAGGGAGTGTTAGGATGCAGTGGCACAGATCTGGTCTTATTGCTTCATGGGAGTTCTTAGAAGAGTATGTCGAAAGCAAGAGT‑‑‑GGAACTGAAGAC‑‑‑CAAATGTCTGATAAAGCATCGTTGGAGAAACATAGCAAGAGAAAATGGGGAGCGATGGCTATAATTAAATCTCTTCAATTTTTGCCTGCGATAATCAGTGCTGCAATAAGGGAAGCAACCCATAACTTTCATGGAGCA‑‑‑CTTCCAAATAGTTCTGCTGAACTGATTGACTATGGCAATATGATGCATATTGCATTGGCAGGCATCAACAATCAGATGAGTTTACTGCAAGACAGTGGTGATGAGGATCAGGCTCAAGAAAGGATCAATAAGTTAGCCAAAATACTTAAGGAAAAGGACGTAGGCTCCACTATACATGCTGCAGGTGTGGGAGTAATTAATTGTATCATACAGAGGGATGAAGGTCGAGCCCCCATGAGGCACTCCTTTTACTGGTCAGCAGAAAATCTCTATTATGAGGAGGAGCCCTTGTTGCGTCATCTAGAACCTCCCCTATCCATTTATCTTGAATTGGACAAACTTAAAGGATATGATAATATACGGTATACTCCATCCCGAGATCGTCAGTGGCACTTGTACACAGTTCTAGATCATAAGCCTCAACCGATTCAAAGAATGTTTCTCCGAACACTTGTAAGGCAGCCAGTTACAAGTGATGGATTCTCTTCATATCAAGGCCTAGATGCAGAAATATCCCGTGCCCAATTGACTATGCCTTTCACTTCAAGGAGCATTTTGAGGTCCTTGCTGACTGCAATGGAGGAGTTGGAACTTAATGCACACAATGCCACTGTCAGACCTGAACATGCTCATATGTACCTCTATATCATACGCGAGCAACAAATTAGTGATCTTTTGCCCTACACAAAGAGAATTGACATAAATGCTGACCAAGAAGAAACAGCAGTTGAGGCAATTTTGGAAGAACTGCCACGTGAAATCCATTCTTTTGTTGGTGTAAGAATGCATAGATTAGGTGTTGCTGAGTGGGAAGTCAAACTCTGGATGGCATCTTCTGGACCAGCAAATGGTGCTTGGAGGATTATTGTAACTAATGTGACTGGTCATACATGCACTGTACATATATACCGAGAACTGGAGGATACCAGCACCCACAAAGTGATATACAGTTCAGTCACTGTAAAGGGTCCTCTGCATGGTGTACCAGTGAATGAAAACTATCGACCTTTGGGAGTCATTGACCGAAAACGTCTTGTAGCAAGGAAGAACAGCACCACTTACTGCTATGATTTTCCACTGGCATTTGAAACAGCATTGGAGCAATCATGGACAAACCAATACCCAGGATTTCAAAAGGCTAAAGATAAA‑‑‑GTTCTAAAAGTAACAGAGCTAAAGTTTGCTGACAAGGAGGGCAATTGGGGTACTCCTCTTGTTCCTGTGGAGCATCCCCTTGGACTCAATGACGTAGGCATGGTAGCCTGGTTAATGGAAATGTGTACTCCTGAGTTCCCAAATGGAAGGAAAATCTTAGTTGTAGCAAATGACGTGACTTTTAAGGCAGGTTCTTTTGGTCCAAGGGAGGATGCCTTCTTTCATGCAGTAACTGACCTTGCGTGTAAGAAAAGATTGCCATTGATTTACCTAGCAGCAAATTCTGGTGCCCGTTTAGGCGTAGCTGAGGAAGTCAAAGCTTGTTTCAGAGTTGGTTGGTCTGATGAATCTAACCCTGAGCATGGTTTGCAGTATGTATACTTAACACCTGAGGATTATGGTAGGATCAAATCATCTGTAATAGCGCACGAGTTGAAGCTGGAAAGTGGAGAAACCAGATGGATAATTGATAGCATTGTTGGGAAAGAGGATGGTCTAGGGGTTGAAAACCTAAGTGGCAGTGGAGCTATTGCTAGTGCCTATTCAAGGGCATATAAGGAAACTTTCACCTTGACATACGTGACCGGAAGAACAGTGGGAATAGGGGCTTATCTTGCTAGGCTGGGAATGAGGTGCATACAGAGGCTTGATCAGCCCATTATTCTTACTGGTTTTTCTGCATTGAACAAACTCCTTGGTCGGGAGGTATACAGTTCTCACATGCAACTTGGTGGGCCTAAGATCATGGCAACAAATGGAGTTGTTCATCTTACTGTTTCAGATGATCTTGAAGGTGTTTCTGCTATTTTGAGGTGGCTCAGCTACATTCCTTCTCATGTAGGTGGTACACTTCCAATTGTACAACCCCATGATCCTCCAGAAAGACCAGTGGAGTATTTCCCAGAGAACTCGTGTGACCCTCGTGCTGCCATATGTGGTTCCGTGGATAGTAATGGAAAATGGCTGGGTGGAATTTTTGACAAGGAAAGCTTTGTGGAGACGCTAGATGGATGGGCCAGGACAGTTGTTACAGGAAGGGCAAAGCTTGGAGGAATCCCTGTGGGAATTGTTGCTGTTGAGACGCAGACTGTGATGCAAGTTATACCAGCTGATCCAGGTCAGCTTGATTCTCATGAAAGGGTGGTTCCTCAGGCAGGGCAGGTCTGGTTTCCTGATTCTGCAACTAAAACAGCCCAGTCAATACTAGATTTCAACAGAGAAGAGCTCCCACTTTTCATTCTTGCAAATTGGAGAGGTTTTTCAGGTGGGCAGAGGGACCTTTTTGAAGGAATTCTTCAAGCTGGATCAACCATTGTGGAGAACCTTCGAACATACAAGCAGCCTGTATTTGTATATATCCCAATGATGGGTGAACTCCGTGGTGGGGCATGGGTTGTTGTAGACAGCCGAATTAATCCAGACCACATTGAAATGTATGCTGATCGCACAGCTAAAGGGAATGTCCTTGAGCCAGAAGGGATGATAGAGATCAAATTTAGGACAAAGGAATTATTGGAGTGCATGGGTAGACTTGACCAGCAGCTGATAAACCTCAAAGCAAAATTACAGGAAGCCAAGAGTAATAGGGCTATCGGGACCATTGATTCCCTGCAGCAGCAGATTAAATCCCGGGAGAAACAGCTTTTGCCTCTGTATACGCAGATAGCTATCAAATTTGCAGAATTACATGATACCTCCCTAAGGATGGCCGCAAAGGGGGTAATAAGAGAAGTTCTGGACTGGCGTAACTCTCGCTCTTTCCTGTACCGCAGACTACGCAGGAGAATTGGTGAACATTCACTGATAAACAGTGTCAGAGATGCTGCT‑‑‑GGTGACCATATGTCTAATATATCTGCAATAGAGTTGATAAAAAATTGGTATTTGAATTCTGATATTGCCAAAGGGAGAGATGATGCTTGGTTGGATGATGAGGCTTTCTTCACATGGAAGAAAGAGCAGCAGAATTACGAGGATAAATTAAAGGAATTGCGTGCCCAAAAGGTGCTGCTTCAACTGACAAATATTGGCGATTCAGTTTTGGACTTGGAAGCTTTACCTCAAGGTCTTGCTGCTCTTTTAAGCAAGTTGGAACCATCAAACCGAGCGAAATTGATTGATGAGCTCCGGAAGGTACTTGGT‑‑‑

>Adu_ACC_Aradu.GHK20

ATGGCTGGTGTTGGGCGTGGAAAT‑‑‑‑‑‑‑‑‑GGATACACAAATGGTGTGGTACCTAACAGGCACCCTGCTACAATATCTGAAGTAGATGAATACTGCAATGCACTTGGGGGAACAAGGCCAATTCATAGTATATTAATTGCAAACAATGGAATGGCCGCAGTCAAGTTTATACGCAGTGTGAGGAGCTGGGCTTATGAGACGTTTGGTACGGAGAAGGCTATCTTGTTGGTTGCCATGGCTACTCCAGAGGACATGAGAATCAATGCAGAACATATCAGAATAGCCGATCAATTTGTCGAAGTACCTGGTGGGACCAATAACAATAACTATGCCAATGTGCAGCTTATTGTAGAG‑‑‑‑‑‑‑‑‑ATGGCTGAGATAACTCGGGTTGATGCTGTGTGGCCAGGTTGGGGTCATGCATCAGAAAATCCTGAGCTTCCAGATGCGTTAAAAGCAAAAGGAATTGTATTTCTTGGACCTCCAGCTGTATCTATGGCAGCACTGGGAGACAAAATTGGTTCATCATTGATTGCTCAAGCAGCAGAAGTGCCAACCCTTCCATGGAGTGGTTCTCATGTGAAAATTCCTCCCGATAGTTGCTTGGTTACTATTCCTGATGAAATTTACCGGGAAGCATGTGTTTATACAACAGAAGAAGCAATTGCCAGTTGTCAAGTTGTCGGTTACCCTGCAATGATCAAAGCATCTTGGGGTGGTGGTGGTAAAGGCATAAGAAAGGTTCATAATGATGATGAGGTAAGGGCATTGTTCAAGCAAGTCCAAGGTGAAGTTCCGGGCTCACCTATATTTATAATGAAGGTTGCCTCCCAGAGCCGACATCTAGAAGTCCAGTTACTTTGTGATCAGTATGGAAATGTTGCAGCTTTGCATAGCCGTGATTGCAGTGTTCAAAGGAGGCACCAAAAGATTATTGAAGAGGGTCCCATTACTGTAGCTCCTCCACAAACGGTGAAACAACTAGAACAGGCAGCTAGAAGGTTGGCTAAATCTGTAAATTATGTTGGGGCTGCTACTGTTGAGTATCTTTTCAGTATGGAAACTGGCGAGTACTACTTTTTGGAATTGAACCCTCGACTACAGGTTGAGCACCCTGTTACTGAGTGGATAGCGGAGATAAATCTGCCAGCAGCACAAGTTGCAATTGGGATGGGTATCCCTCTTTGGCAACTTCCTGAAATAAGACGTTTCTATGGGGTGGAACATGGTGGGGGGAATGATGCTTGGAGGAAAACATCAGCTTTGGCTACCCCTTTTGATTTTGACAAAGCACAATCCACAAAGCCAAAAGGTCACTGTGTGGCTGTGCGAGTGACAAGTGAGGACCCTGATGATGGTTTCAAGCCTACAAGTGGGAAAGTGCAGGAGCTTAGCTTTAAAAGCAAGCCAAATGTGTGGGCATACTTCTCTGTTAAGTCTGGAGGAGGAATACACGAGTTTTCAGATTCTCAGTTTGGGCATGTTTTTGCTTTTGGAGAATCTAGGGCTTTAGCTATAGCAAATATGGTTCTAGGGCTGAAGGAGATTCAAATTCGTGGAGAGATTCGTACCAATGTTGATTATACCATTGATCTTCTGAATGCTTCAGACTACAGAGACAACAAAATTCACACGGGATGGCTGGACAGTAGAATTGCGATGAGGGTTAGAGCAGAGAGGCCTCCCTGGTATCTTTCTGTTGTTGGAGGGGCCCTCTATAAAGCTTCTGCAAGCAGTGCAGCTCTGGTATCAGACTATGTTGGCTATCTGGAAAAGGGACAAATCCCTCCCAAGCATATATCTCTTGTGCATTCTCAAGTGTCCTTGAACATTGAAGGAAGCAAATACACGATTGACATGGTACGAGGAGGGTCTGGAAGTTATAGATTGAGAATGAATCAATCCGAAGTAGAAGCTGAGATACATACTTTACGTGATGGAGGTTTGCTGATGCAGGCAAGTATTTTGGATGGAAACAGTCATGTTATATATGCAGAGGAAGAAGCTGCTGGAACGCGCCTTCTAATTGATGGAAGGACTTGCTTGCTTCAGAATGATCATGATCCATCGAAGTTAGTTGCAGAGACACCATGCAAGCTTATGAGGTATTTGGTTGTAGATGACAGCCATATTGATGCTGACACACCTTATGCTGAAGTTGAAGTCATGAAGATGTGCATGCCACTTCTTTCACCTGCTTCTGGGGTTATTCATTTCAAAATGTCTGAAGGTCAACCGATGCAGGCTGGTGAACTAATAGCAAGGCTTGATCTAGATGATCCTTCAGCAGTAAGAAAGGCAGAACCCTTCAATGGAAAATTCCCAGTCCTGGGCCCACCCACTGCAACTTCTGATAAAGTTCATCAGAAATGTGCTGCAAGCTTAAGTGCTGCACAGATGATTCTTGCTGGTTATGAGCACAATATTGATGAGGTTGTGCAAAGTTTGCTCAATTGCCTTGATAGTCCTGAATTACCTTTCCTTCAATGGCAAGAGTGCTTTGCAGTTCTAGCAAACCGTCTTCCCAAAGATCTGAAAAATGAGTTGGAATCGAAATATAAGGAGTACGAGAGGATTTCA‑‑‑AGCTTCCAAGTTGTTGATTTCCCTGCCAAACTTTTGAAGGGAATTCTTGAAGCTCATTTGTCCTCATGTCCCAACAAAGAAAAAGGGGCTCAAGAAAGGCTGATTGAACCTCTGTTGAGTCTTGTGAAGTCCTATGAGGGTGGAAGAGAGAGCCATGCTCGTAAAATTGTCCAATCCCTTTTTGAAGAGTATCTTTTTGTTGAAGAACTATTTAGTGATAATATACAGGCTGATGTAATTGAACGTCTCCGTCTTCAATATAAGAAAGATCTGTTGAAGATTGTGGATATAGTTCTCTCACATCAGGGTATCAAGAGCAAAAATAAGCTGATACTACGACTAATGGATAAACTGGTTTACCCAAATCCTGCTGCCTACAGGGATCAATTAATCCGCTTCTCTCAACTCAACCATACTAACTATTCTCAGTTGGCCCTAAAGGCAAGTCAATTGCTGGAACAAACTAAATTGAGTGAACTTCGATCCAACATTGCTAGAAGTCTTTCTGAGTTAGAGATGTTCACTGAGGATGGTGAAAATATTGATACTCCCAAGAGGAAAAGCGCTATTAATGACCGAATGGAGGACCTTGTTAGTGCTCCTTTGGCAGTTGAAGATGCTTTGGTGGGTTTATTTGATCACAGTGATCACACCCTTCAAAGAAGGGTTGTGGAAACTTATATACGCAGACTCTACCAGCCGTATCTTGTCAAAGGGAGTGTCAGAATGCAGTGGCACAGATCTGGTCTTATTGCTTCATGGGAGTTCTTAGAAGAGTACATTGAAAGGAAGAGT‑‑‑GGGGTTGAAGAC‑‑‑CAAATGTCAGATAAAACGCTGGTGGAGAAACACACTGAGAAAAAATGGGGCGTAATGGTTGTAATTAAATCTCTTCACTTTTTGCCTGCAATTATCACTGCTGCGTTAAAGGAAGCAACCAACAATCTTCATGAAGCA‑‑‑GTTTCAAGTGCTGCTGGTGAACCAGTTAAGCATGGTAATATGATGCATGTTGCATTAGTGGGCATCAACAACCAGATGAGTTTACTGCAAGACAGTGGTGACGAGGATCAGGCTCAAGAAAGAATCAATAAGTTGGCGAAAATACTAAAAGAAGAGGAAGTAGGCTCCACTATACGAGGTACTGGTGTTGGAGTAATTAGCTGTATCATACAGAGGGATGAAGGGCGTACCCCAATGAGGCACTCCTTTCACTGGTCAGCAGAAAAGCTCTATTATCAGGAGGAACCTCTGTTGCGTCATTTGGAACCACCGCTATCCATTTATCTTGAATTGGACAAACTTAAAGGCTATGAGAACATACGGTATACTCCTTCTCGAGATCGTCAATGGCATCTGTACACTGTTATGGACCAAAAGCCTCAACCTGTTCAAAGAATGTTTCTTCGAACACTTTTAAGACAGCCAACCACAAATGAAGGATTCTCTTCGTATCAAAGGACGGATGCAGAAACACCTAGTACCGAATTGGCTATGTCCTTCACTTCAAGGAGCATTTTTAGGTCCTTGATGGCTGCAATGGAGGAGTTGGAACTTAATTCACACAATGCCACTATCAGACCTGAACACGCTCATATGTACCTCTATATTATACGCGAGCAGGAAATAAATGATCTTGTGCCTTATCCCAAGAGAGTTGACATAGATGCGGGCCAAGAAGAAACAACAGTTGAGGCAACCTTGGAAGAACTAGCACATGAAATCCATTCATCGGTTGGTGTAAGAATGCATAGATTAGGAGTTGTAGTTTGGGAAGTCAAGCTCTGGATGGCAGCCTGTGCACAGGCAAATGGCGCATGGAGGATTGTTGTAAACAATGTGACAGGTCATACATGCACTGTACATATATACCGAGAAATGGAAGATACCAATACCCATAGAGTGGTATACAGTTCAATCACCGTAAAGGGTCCACTGCATGGTGTACCTGTGAATGAAACTTATCAACCTTTGGGAGTTATTGATCGAAAACGTCTATCAGCAAGAAAGAACAGTACCACTTTTTGCTATGATTTCCCCCTGGCATTTGAAACAGCCTTGGAACAGTCGTGGGCAATCCAACAGCCGGGATTTCGAAGACCAAAAGATAAAAATCTGTTAAAGGTAACAGAGCTTAGATTTGCTGACAAAGAGGGTAGTTGGGGTACTCCTCTTGTTCCTGTGGAGCATTCTGCTGGACTCAATGATGTTGGCATGGTAGCTTGGTTTATGGACATGTGTACCCCTGAATTCCCATCCGGAAGGACAATATTAGTTGTAGCAAATGATGTGACATTCAAGGCTGGTTCTTTTGGCCCTAGAGAGGATGCATTCTTCCGTGCAGTTACTGATCTTGCATGTGCAAAAAAACTGCCTTTAATTTATTTAGCAGCAAATTCTGGTGCCCGTTTAGGTGTTGCCGAGGAAGTCAAAGCCTGTTTTAAAGTTGGTTGGTCTGAGGAGTCCAACCCTGAGCATGGTTTTCAGTATGTATATTTAACACCTGAGGATTTTGCTCGGATTGGATCATCTGTGATTGCACACGAGCTAAAGCTTGAAAGCGGTGAAACCAGATGGATAATAGATACCATTGTTGGGAAAGAGGATGGCCTGGGGGTTGAAAACTTGAGTGGTAGTGGAGCCATTGCTGGTTCCTACTCAAGGGCATACAAGGAAACTTTCACATTAACATATGTGACTGGTAGGACTGTTGGAATAGGGGCTTATCTTGCTAGGCTTGGAATGAGATGCATACAGAGGCTTGATCAGCCTATAATTCTTACTGGTTTCTCAGCACTAAACAAACTTCTTGGTCGGGAGGTATACAGCTCTCACATGCAACTTGGTGGACCTAAAATCATGGCTACTAATGGAGTTGTACATCTTACAGTTTCAGATGACCTTGAAGGTGTTTCTGCTATTTTGAAGTGGCTTAGCTACATTCCTTCTCATGTAGGTGGCTCACTTCCCATCGTAAAGCCCCTTGACCCTCCTGAAAGACCAGTGGAGTACTTACCAGAAAATTCTTGTGATCCTCGTGCTGCTATTTCTGGAACTTTGGATGGTAATGGAAGATGGCTCGGTGGAATTTTCGACAAGGACAGCTTCGTGGAGACACTAGAAGGATGGGCAAGGACAGTCGTTACAGGAAGGGCAAAGCTTGGAGGAATCCCTGTGGGGATTGTTGCTGTAGAAACACAGACAGTGATGCAAATAATACCTGCTGATCCGGGCCAGCTTGATTCCCATGAGAGAGTTGTTCCTCAGGCTGGCCAAGTGTGGTTCCCTGATTCTGCTACTAAAACAGCCCAAGCAATAATGGATTTCAACAGAGAAGAGCTCCCACTTTTCATTCTAGCAAACTGGAGAGGCTTCTCTGGTGGTCAGAGGGACCTTTTCGAAGGAATTCTCCAGGCTGGTTCGACAATTGTGGAGAACCTTAGAACATACAAGCAGCCCATTTTTGTCTACATCCCAATGATGGGTGAACTCCGTGGTGGAGCATGGGTTGTCGTTGACAGTCGGATCAATTCCGACCACATTGAAATGTATGCCGACAGAACAGCTAAAGGAAATGTCCTTGAACCAGAAGGGATGATTGAGATTAAGTTTAGAACAAGGGAATTGTTGGAGTGCATGGGTAGACTTGATCAGAAGTTGATAACTCTGAAGGCAAAACTTCAGGAGGCTAAGGACAAGAGGGACACCGAGTCCTTTGAATCTCTACAGCAGCAGATTAAATCTCGCGAAAAACAGCTTTTGCCTCTGTATACCCAGATAGCTACCAAATTTGCTGAACTTCATGATACTTCCTTAAGAATGGCTGCTAAGGGGGTAATAAGACAAGTTCTGGACTGGGGTAACTCGCGCGCCGTCTTCTACCGGAGACTGTACAGGAGAATTGGTGAGCAGTCACTTATCAACAATGTGAGAGAAGCTGCT‑‑‑GGTGACCATTTGTCACATGTTTCCGCCATGGACTTAGTCAAAAACTGGTATTTGAGTTCCAACATTGCCAAAGGTAGAAAAGATGCTTGGCTGGACGATGAAGCCTTCTTCAGTTGGAAGGAAAATCCATCGAATTATGAGGATAAACTGAAGGAATTGCGTGCACAGAAAGTGTTGCTTCAATTGACAAACATTGGTGACTCGGTTCTAGATTTGCAAGCTCTGCCTCAAGGACTTGCTGCTCTTTTAAGCAAGTTGGAGCCATCGAGTCGGGTGAAGTTGGCGGAAGAACTTCGAAAAGTACTTGGT‑‑‑

>Aip_ACC_Araip.ZAK9V

ATGGCTGGTGTTGGGCGTGGAAAT‑‑‑‑‑‑‑‑‑GGATACACAAATGGTGTGGTACCTAACAGGCACCCTGCTACAATATCTGAAGTAGATGAATACTGCAATGCACTTGGGGGAACAAGGCCAATTCATAGTATATTAATTGCAAACAATGGAATGGCCGCAGTCAAGTTTATACGTAGTGTGAGGAGCTGGGCTTATGAGACGTTTGGTACGGAGAAGGCTATCTTGTTGGTTGCCATGGCTACTCCAGAGGACATGAGAATCAATGCAGAACATATCAGAATAGCCGATCAATTTGTCGAAGTACCTGGTGGGACCAATAACAATAACTATGCCAATGTGCAGCTTATTGTAGAG‑‑‑‑‑‑‑‑‑ATGGCTGAGATAACTCGGGTTGATGCTGTGTGGCCAGGTTGGGGTCATGCATCAGAAAATCCTGAGCTTCCAGATGCATTAAAAGCAAAAGGAATTGTATTTCTTGGACCTCCAGCTGTATCTATGGCAGCACTGGGAGACAAAATTGGTTCATCATTGATTGCTCAAGCAGCAGAAGTGCCAACCCTTCCATGGAGTGGTTCTCATGTGAAAATTCCTCCCGATAGTTGCTTGGTTACTATTCCTGATGAAATTTACCGGGAAGCATGTGTTTATACAACAGAAGAAGCAATTGCCAGTTGTCAAGTTGTCGGTTACCCTGCAATGATCAAAGCATCTTGGGGTGGTGGTGGTAAAGGCATAAGAAAGGTTCATAATGATGATGAGGTAAGGGCATTGTTCAAGCAAGTCCAAGGTGAAGTTCCGGGCTCACCTATATTTATAATGAAGGTTGCCTCCCAGAGCCGACATCTAGAAGTCCAGTTACTTTGTGATCAGTATGGAAATGTTGCAGCTTTGCATAGCCGTGATTGCAGTGTTCAAAGGAGGCACCAAAAGATTATTGAAGAGGGTCCCATTACTGTAGCTCCTCCACAAACGGTGAAACTACTAGAACAGGCAGCTAGAAGGTTGGCTAAATCTGTAAATTATGTTGGGGCTGCTACTGTTGAGTATCTTTTCAGTATGGAAACTGGCGAGTACTACTTTTTGGAATTGAACCCTCGACTACAGGTTGAGCACCCTGTTACTGAGTGGATAGCGGAGATAAATCTGCCAGCAGCACAAGTTGCAATTGGGATGGGTATCCCTCTTTGGCAACTTCCTGAGATAAGACGTTTCTATGGGGTGGAACATGGTGGGGGGAATGATGCTTGGAGGAAAACATCAGCTTTGGCTACCCCTTTTGATTTTGACAAAGCACAATCCACAAAGCCAAAAGGTCACTGTGTGGCTGTGCGAGTGACAAGTGAGGACCCTGATGATGGTTTTAAGCCTACAAGTGGGAAAGTGCAGGAGCTTAGCTTTAAAAGCAAGCCAAATGTGTGGGCATACTTCTCTGTTAAGTCTGGAGGAGGAATACACGAGTTTTCAGATTCTCAGTTTGGGCATGTTTTTGCTTTTGGAGAATCTAGGGCTTTAGCTATAGCAAATATGGTTCTAGGGCTGAAGGAGATTCAAATTCGTGGAGAGATTCGTACCAATGTTGATTATACCATTGATCTTCTGAATGCTTCAGACTACAGAGACAACAAAATTCACACGGGATGGCTGGACAGTAGAATTGCGATGAGGGTTAGAGCAGAGAGGCCTCCCTGGTATCTTTCTGTTGTTGGAGGGGCCCTCTATAAAGCTTCTGCAAGCAGTGCAGCTCTGGTATCAGACTATGTTGGCTATCTGGAAAAGGGACAAATCCCTCCCAAGCATATATCTCTTGTGCATTCTCAAGTGTCCTTGAACATTGAAGGAAGCAAATACACAATTGACATGGTACGAGGAGGGTCTGGAAGTTATAGATTGAGAATGAATCAATCCGAAGTAGAAGCTGAGATACATACTTTACGTGATGGAGGTTTGCTGATGCAGGCAAGTATTTTGGATGGAAACAGTCATGTTATATATGCAGAGGAAGAAGCTGCTGGAACGCGCCTTCTAATTGATGGAAGGACTTGCTTGCTTCAGAATGATCATGATCCATCGAAGTTAGTTGCAGAGACACCATGCAAGCTTATGAGGTATTTGGTTGTAGATGACAGCCATATTGATGCTGACACACCTTATGCTGAAGTTGAAGTCATGAAGATGTGCATGCCACTTCTTTCACCTGCTTCTGGGGTTATTCATTTCAAAATGTCTGAAGGTCAACCGATGCAGGCTGGTGAACTAATAGCAAGGCTTGATCTAGATGATCCTTCAGCAGTAAGAAAGGCAGAACCCTTCAATGGAAAATTCCCAGTCCTGGGCCCACCCACTGCAACTTCTGATAAAGTTCATCAGAAATGTGCTGCAAGCTTAAATGCTGCACAGATGATTCTTGCTGGTTATGAGCACAATATTGATGAGGTTGTGCAAAGTTTGCTCAATTGCCTTGATAGTCCTGAATTACCTTTCCTTCAATGGCAAGAGTGCTTTGCAGTTCTAGCAAACCGTCTTCCCAAAGATCTGAAAAATGAGTTGGAATCGAAATATAAGGAGTACGAGAGGATTTCA‑‑‑AGCTTCCAAGTGGTTGATTTCCCTGCCAAACTTTTGAAGGGAATTCTTGAAGCTCATTTGTCCTCATGTCCCAACAAGGAAAAAGGGGCTCAAGAAAGGCTGATTGAACCTCTGTTGAGTCTTGTGAAGTCCTATGAGGGTGGAAGAGAGAGCCATGCTCGTAAAATTGTCCAATCCCTTTTTGAAGAGTATCTTTTTGTTGAAGAACTATTTAGTGATAATATACAGGCTGATGTAATTGAACGTCTCCGTCTTCAATATAAGAAAGATCTGTTGAAGATTGTGGATATAGTTCTCTCACATCAGGGTATCAAGAGTAAAAATAAGCTGATACTACGACTAATGGATAAACTGGTTTACCCCAATCCTGCTGCCTACAGGGATCAATTAATCCGCTTCTCTCAACTCAACCATACTAACTATTCTCAGTTGGCCCTTAAGGCAAGTCAATTGCTGGAACAAACTAAATTGAGTGAACTTCGATCCAACATTGCTAGAAGTCTTTCTGAGTTAGAGATGTTCACTGAGGATGGTGAAAATATTGATACTCCCAAGAGGAAAAGCGCTATTAATGACCGAATGGAGGACCTTGTTAGTGCTCCTTTGGCAGTTGAAGATGCTTTGGTGGGTTTATTTGATCACAGTGATCACACCCTTCAAAGAAGGGTTGTGGAAACTTATATACGCAGACTCTACCAGCCATATCTTGTCAAAGGGAGTGTCAGAATGCAGTGGCACAGATCTGGTCTTATTGCTTCATGGGAGTTCTTAGAAGAGTACATTGAAAGAAAGAGT‑‑‑GGGGTTGAAGAC‑‑‑CAAATGTCAGATAAAACGCTGGTGGAGAAACACACTGAGAAAAAATGGGGCGTAATGGTTGTAATTAAATCTCTTCACTTTTTGCCTGCAATTATCACTGCTGCGTTAAAGGAAGCAACCAATAATCTTCATGAAGCA‑‑‑GTTTCAAGTGCTGCTGGTGAACCAGTTAAGCATGGTAATATGATGCATGTTGCATTAGTGGGCATCAACAACCAGATGAGTTTACTGCAAGACAGTGGTGACGAGGATCAGGCTCAAGAAAGAATCAATAAGTTGGCGAAAATACTAAAAGAAGAGGAAGTAGGCTCCACTATACGAGGTACTGGTGTTGGAGTAATTAGCTGTATCATACAGAGGGATGAAGGGCGTACCCCGATGAGGCACTCATTTCACTGGTCAGCAGAAAAGCTCTATTATCAGGAGGAACCTCTGTTGCGTCATTTGGAACCACCGCTATCCATTTACCTTGAATTGGACAAACTTAAAGGCTATGAGAACATACGGTATACTCCTTCTCGAGATCGTCAATGGCATCTGTACACTGTTATGGACCAAAAGCCTCAACCTGTTCAAAGAATGTTTCTTCGAACACTTTTAAGACAGCCAACCACAAATGAAGGATTCTCTTCGTATCAAAGGACGGATGCAGAAACACCTAGTACCGAATTGGCTATGTCCTTCACTTCAAGGAGCATTTTTAGGTCCTTGATGGCTGCAATGGAGGAGTTGGAACTTAATTCACACAATGCCACTATCAGACCTGAACACGCTCATATGTACCTCTATATTATACGCGAGCAGGAAATAAATGATCTTGTGCCTTATCCCAAGAGAGTTGACATAGATGCGGGCCAAGAAGAAACAACAGTTGAGGCAACCTTGGAAGAACTAGCACATGAAATCCATTCATCGGTTGGTGTAAGAATGCATAGATTAGGAGTTGTAGTTTGGGAAGTCAAGCTCTGGATGGCAGCCTGTGGACAGGCAAATGGCGCATGGAGGATTGTTGTAAACAATGTGACAGGTCATACATGCACTGTACATATATACCGAGAAATGGAAGATACCAATACTCATAGAGTGGTATACAGTTCAATCACTGTAAAGGGTCCACTGCATGGTGTACCTGTGAATGAAACTTATCAACCTTTGGGAGTTATTGATCGAAAACGTCTATCAGCAAGAAAGAACAGTACCACTTTTTGCTATGATTTCCCCCTGGCATTTGAAACAGCCTTGGAACAGTCGTGGGCAATCCAACAGCCGGGATTTCGAAGACCAAAAGATAAAAATCTGTTAAAGGTAACAGAGCTTAGATTTGCTGACAAAGAGGGTAGTTGGGGTACTCCTCTTGTTCCTGTGGAGCATTCTGCTGGACTCAATGATGTTGGGATGGTAGCTTGGTTTATGGACATGTGTACCCCCGAATTCCCATCCGGAAGGACAATATTGGTTGTAGCAAATGATGTGACATTCAAGGCTGGTTCTTTTGGCCCTAGAGAGGATGCATTCTTCCGTGCAGTTACTGATCTTGCATGTGCAAAAAAACTGCCTTTAATTTATTTAGCAGCAAATTCTGGTGCCCGTTTAGGTGTTGCCGAGGAAGTCAAAGCCTGTTTTAAAGTTGGTTGGTCTGAGGAGTCCAACCCTGAGCATGGTTTTCAGTATGTATATTTAACACCTGAGGATTTTGCTCGGATTGGATCATCTGTGATTGCACACGAGCTAAAGCTTGAAAGCGGTGAAACCAGATGGATAATAGATACCATTGTTGGGAAAGAGGATGGCCTGGGGGTTGAAAACTTGAGTGGTAGTGGAGCCATTGCTGGTTCCTACTCAAGGGCATACAAGGAAACTTTCACATTAACATATGTGACTGGTAGGACTGTTGGAATAGGGGCTTATCTTGCTAGGCTTGGAATGAGATGCATACAGAGGCTTGATCAGCCTATAATTCTTACTGGTTTCTCAGCACTAAACAAACTTCTTGGTCGGGAGGTATACAGCTCTCACATGCAACTTGGTGGACCTAAAATCATGGCTACTAATGGAGTTGTACATCTTACAGTTTCAGATGACCTTGAAGGTGTTTCTGCTATTTTGAAGTGGCTTAGCTACATTCCTTCTCATGTAGGTGGCTCACTTCCCATCGTAAAGCCCCTTGACCCTCCTGAAAGACCAGTGGAGTACTTACCAGAAAATTCTTGTGATCCTCGTGCTGCTATTTCTGGAACTTTGGATGGTAATGGAAGATGGCTCGGTGGAATTTTCGACAAGGACAGCTTCGTGGAGACACTAGAAGGATGGGCAAGGACAGTCGTTACAGGAAGGGCAAAGCTTGGAGGAATCCCTGTGGGGATTGTTGCTGTAGAAACACAGACAGTGATGCAAATAATACCTGCTGATCCGGGCCAGCTTGATTCCCATGAGAGAGTTGTTCCTCAGGCTGGCCAAGTGTGGTTCCCTGATTCTGCTACTAAAACAGCCCAAGCAATAATGGATTTCAACAGAGAAGAGCTCCCACTTTTCATTCTAGCAAACTGGAGAGGCTTCTCTGGTGGTCAGAGGGACCTTTTCGAAGGAATTCTCCAGGCTGGTTCGACAATTGTGGAGAACCTTAGAACATACAAGCAGCCCATTTTTGTATACATCCCAATGATGGGTGAACTCCGTGGTGGAGCATGGGTTGTCGTTGACAGTCGGATCAATTCCGACCATATTGAAATGTATGCCGACAGAACAGCTAAAGGAAATGTCCTTGAACCAGAAGGGATGATTGAGATTAAGTTTAGAACAAGGGAATTGTTGGAGTGCATGGGTAGACTTGATCAGAAGTTGATAACTCTGAAGGCAAAACTTCAGGAGGCTAAGGACAAGAGGGACACCGAGTCCTTTGAATCTCTACAGCAGCAGATTAAATCTCGCGAAAAACAGCTTTTGCCTCTGTATACCCAGATAGCTACCAAATTTGCTGAACTTCATGATACTTCCTTAAGAATGGCTGCTAAGGGGGTAATAAGACAAGTTCTGGACTGGGGTAACTCGCGCGCCGTCTTCTACCGGAGACTGTACAGGAGAATTGGTGAGCAGTCACTTATCAACAATGTGAGAGAAGCTGCT‑‑‑GGTGACCATTTGTCACATGTTTCCGCCATGGACTTGGTCAAAAACTGGTATTTGAGTTCCAACATTGCCAAAGGTAGAAAAGATGCTTGGCTGGACGATGAAGCCTTCTTCAGTTGGAAGGAAAATCCATTGAATTATGAGGATAAACTAAAGGAATTGCGTGCACAGAAAGTGTTGCTTCAATTGACAAACATTGGTGACTCGGTTCTAGATTTGCAAGCTCTGCCTCAAGGACTTGCTGCTCTTTTAAGCAAGTTGGAGCCATCGAGTCGGGTGAAGTTGACGGAAGAACTTCGAAAAGTACTTGGT‑‑‑

>Ath_ACC1_AT1G36160

ATGGCT‑‑‑‑‑‑GGCTCGGTTAAC‑‑‑‑‑‑‑‑‑GGGAATCATAGTGCTGTAGGACCTGGTATAAATTATGAGACGGTGTCTCAAGTGGATGAGTTCTGTAAAGCACTTAGAGGGAAAAGGCCGATCCATAGTATTTTGATAGCTAACAATGGAATGGCGGCTGTGAAGTTTATACGTAGTGTCAGAACATGGGCTTATGAAACATTTGGTACGGAAAAAGCCATATTGTTGGTGGGGATGGCAACCCCTGAAGACATGCGGATCAATGCGGAGCATATCAGAATCGCTGATCAGTTTGTTGAGGTTCCCGGAGGAACCAACAATAACAATTATGCTAACGTTCAGCTGATTGTGGAG‑‑‑‑‑‑‑‑‑ATGGCTGAAGTAACACGCGTGGATGCAGTTTGGCCTGGTTGGGGTCATGCATCTGAAAACCCCGAATTACCTGATGCCCTAGATGCAAAAGGAATCATATTTCTTGGTCCTCCAGCATCTTCAATGGCAGCACTGGGAGATAAGATTGGTTCTTCGTTGATTGCACAAGCTGCTGATGTACCCACTCTGCCATGGAGTGGTTCCCATGTTAAAATACCTCCTAATAGCAACTTGGTAACCATCCCAGAGGAGATCTACCGGCAAGCATGTGTCTACACAACTGAAGAAGCGATTGCTAGCTGTCAAGTTGTCGGTTACCCAGCAATGATCAAAGCATCGTGGGGTGGTGGTGGTAAAGGAATCAGGAAGGTTCATAATGATGATGAGGTTAGGGCTCTATTCAAGCAAGTTCAGGGTGAGGTCCCAGGCTCACCAATATTCATAATGAAGGTTGCGTCACAGAGTCGGCATCTAGAGGTCCAGCTGCTCTGTGACAAGCATGGAAATGTTTCAGCTCTGCATAGCCGTGATTGTAGCGTCCAGAGAAGACATCAAAAGATCATAGAGGAGGGTCCAATTACTGTGGCTCCGCCAGAAACTGTCAAGAAACTTGAACAAGCAGCTAGAAGGTTGGCTAAGAGTGTTAACTATGTTGGAGCTGCTACTGTTGAGTATCTCTACAGTATGGACACTGGGGAGTACTACTTCTTAGAGCTTAACCCTCGCTTACAGGTTGAGCATCCTGTCACTGAGTGGATTGCCGAGATAAATCTTCCTGCTGCCCAAGTTGCTGTGGGGATGGGAATTCCTCTCTGGCAAATCCCTGAGATAAGACGGTTCTATGGAATAGAACATGGTGGAGGTTATGATTCTTGGCGAAAAACATCTGTTGTAGCCTTCCCTTTTGATTTTGATAAAGCTCAATCTATAAGGCCAAAAGGTCATTGTGTGGCTGTACGTGTGACAAGTGAGGATCCTGATGACGGGTTCAAACCAACCAGCGGTAGAGTTCAGGAGTTGAGTTTTAAGAGCAAGCCAAATGTGTGGGCGTACTTCTCTGTCAAGTCTGGTGGAGGCATCCACGAGTTCTCGGATTCCCAGTTTGGACATGTTTTTGCATTTGGGGAATCCAGAGCCCTGGCGATAGCGAATATGGTTCTTGGGCTAAAAGAAATTCAGATCCGTGGAGAAATTAGGACTAACGTTGACTACACGATCGACCTTTTACATGCTTCTGATTACCGTGATAACAAAATTCACACTGGTTGGTTGGATAGTAGGATTGCTATGCGGGTCAGAGCTGAGAGGCCTCCATGGTATCTCTCTGTTGTCGGCGGAGCTCTCTATAAAGCATCAGCGACCAGTGCTGCTGTGGTTTCAGATTACGTTGGTTATCTGGAGAAGGGGCAAATCCCTCCAAAGCATATATCTCTTGTACATTCTCAAGTGTCTCTGAATATTGAAGGAAGTAAATATACGATTGATGTAGTCCGGGGTGGATCAGGAACCTACAGGCTAAGAATGAACAAGTCAGAAGTGGTAGCAGAAATACACACTCTACGTGATGGAGGTCTGTTGATGCAG‑‑‑‑‑‑‑‑‑TTGGATGGCAAAAGCCATGTGATATATGCAGAGGAAGAAGCTGCAGGAACTCGTCTTCTCATTGATGGAAGAACTTGTTTGCTACAGAATGACCACGATCCATCAAAGTTAATGGCTGAGACACCGTGCAAGTTGATGAGGTATTTGATTTCCGACAACAGCAATATTGACGCTGATACGCCTTATGCCGAAGTTGAGGTCATGAAGATGTGCATGCCACTTCTTTCACCTGCTTCAGGAGTTATCCATTTTAAAATGTCTGAAGGACAAGCCATGCAGGCTGGTGAACTTATAGCCAATCTTGATCTTGATGATCCTTCTGCTGTAAGAAAGGCCGAACCCTTCCATGGAAGTTTCCCAAGATTAGGGCTTCCAACTGCAATATCCGGTAGAGTTCATCAGAGATGTGCCGCAACATTAAATGCTGCACGCATGATTCTTGCTGGCTATGAGCATAAAGTAGATGAGGTTGTTCAAGACTTACTTAATTGCCTTGATAGCCCTGAACTCCCATTTCTTCAGTGGCAAGAGTGCTTTGCAGTTCTGGCGACACGACTACCTAAAAATCTCAGGAACATGCTAGAATCAAAGTATAGGGAATTTGAGAGTATTTCCAGAAACTCTTTGACCACCGATTTCCCTGCCAAACTTTTAAAAGGCATTCTTGAGGCACATTTATCTTCTTGTGATGAGAAAGAGAGAGGTGCCCTTGAAAGGCTCATTGAACCATTGATGAGCCTTGCAAAATCTTATGAAGGTGGTAGAGAAAGTCATGCCCGTGTTATTGTTCATTCTCTCTTTGAAGAATATCTATCAGTAGAAGAATTATTCAATGATAACATGCTGGCTGATGTTATAGAACGCATGCGTCAGCTATACAAGAAAGATCTGTTGAAAATTGTGGATATAGTGCTCTCACACCAGGGCATAAAAAACAAAAACAAACTCGTTCTCCGGCTCATGGAGCAGCTTGTTTACCCTAATCCTGCTGCTTACAGAGATAAACTTATTCGATTCTCAACACTTAACCATACTAACTACTCTGAGTTGGCGCTCAAGGCGAGTCAATTACTTGAACAGACCAAACTAAGTGAGCTTCGTTCAAACATTGCTAGAAGCCTTTCAGAGTTAGAAATGTTTACAGAGGACGGAGAAAATATGGATACTCCCAAGAGGAAAAGTGCCATTAATGAAAGAATAGAAGATCTTGTAAGCGCATCTTTAGCTGTTGAAGACGCTCTCGTGGGACTATTTGACCATAGCGATCACACACTTCAAAGACGGGTTGTTGAGACTTATATTCGCAGATTATACCAGCCCTACGTCGTTAAAGATAGCGTGAGGATGCAGTGGCACCGTTCTGGTCTTCTTGCTTCCTGGGAGTTCCTAGAGGAGCATATGGAAAGAAAAAACATTGGCTTAGACGATCCCGACACATCTGAAAAAGGATTGGTTGAGAAGCGTAGTAAGAGAAAATGGGGGGCTATGGTTATAATCAAATCTTTGCAGTTTCTTCCAAGTATAATAAGTGCAGCATTGAGAGAAACAAAGCACAACGACTATGAAACT‑‑‑‑‑‑‑‑‑‑‑‑‑‑‑‑‑‑GCCGGAGCTCCTTTATCTGGCAATATGATGCACATTGCTATTGTGGGCATCAACAACCAGATGAGTCTGCTTCAGGACAGTGGGGATGAAGACCAAGCTCAGGAAAGAGTAAACAAGTTGGCCAAAATTCTTAAAGAGGAAGAAGTGAGTTCAAGCCTCTGTTCTGCCGGTGTTGGTGTAATCAGCTGTATAATTCAGCGAGATGAAGGACGAACACCCATGAGACATTCTTTCCATTGGTCGTTGGAGAAACAGTATTATGTAGAAGAGCCGTTGCTGCGTCATCTTGAACCTCCTCTGTCCATTTACCTTGAGTTGGATAAGCTGAAAGGATACTCAAATATACAATATACGCCTTCTCGAGATCGTCAATGGCATCTGTATACTGTTACAGAC‑‑‑AAGCCAGTGCCAATCAAGAGGATGTTCCTGAGATCTCTTGTTCGACAGGCTACAATGAACGATGGATTTATATTGCAGCAAGGGCAGGATAAGCAGCTTAGCCAAACACTGATCTCCATGGCGTTTACGTCGAAATGTGTTCTGAGGTCTTTGATGGATGCCATGGAGGAACTGGAACTGAATGCCCATAATGCTGCAATGAAACCAGATCACGCACATATGTTTCTTTGCATATTGCGTGAGCAGCAGATAGATGATCTTGTGCCTTTCCCCAGGAGAGTTGAAGTGAATGCGGAGGATGAAGAAACTACAGTTGAAATGATCTTAGAAGAAGCAGCACGAGAGATACATAGATCTGTTGGAGTGAGAATGCATAGGTTGGGCGTGTGCGAGTGGGAAGTGCGGCTGTGGTTGGTGTCCTCTGGACTGGCATGTGGTGCTTGGAGGGTTGTGGTTGCAAACGTGACAGGCCGTACATGCACTGTCCACATATACCGAGAAGTTGAAACTCCTGGAAGAAACAGTTTAATCTACCACTCAATAACCAAGAAGGGACCTTTGCATGAAACACCAATCAGTGATCAATATAAGCCCCTGGGATATCTCGACAGGCAACGTTTAGCAGCAAGGAGGAGTAACACTACTTATTGCTATGACTTCCCGTTGGCATTTGGGACAGCCTTGGAACTGTTGTGGGCATCACAACACCCAGGAGTTAAGAAACCATATAAGGATACTCTGATCAATGTTAAAGAGCTTGTATTCTCAAAACCAGAAGGTTCTTCGGGTACATCTCTAGATCTGGTTGAAAGACCACCCGGTCTCAACGACTTTGGAATGGTTGCCTGGTGCCTAGATATGTCGACCCCAGAGTTTCCTATGGGGCGGAAACTTCTCGTGATTGCGAATGATGTCACCTTCAAAGCTGGTTCTTTTGGTCCTAGAGAGGACGCGTTTTTCCTTGCTGTTACTGAACTCGCTTGTGCCAAGAAGCTTCCCTTGATTTACTTGGCAGCAAATTCTGGTGCCCGACTTGGGGTTGCTGAAGAAGTCAAAGCCTGCTTCAAAGTTGGATGGTCGGATGAAATTTCCCCTGAGAATGGTTTTCAGTATATATACCTAAGCCCTGAAGACCACGAAAGGATTGGATCATCTGTCATTGCCCATGAAGTAAAGCTCTCTAGTGGGGAAACTAGGTGGGTGATTGATACGATCGTTGGCAAAGAAGATGGTATTGGTGTAGAGAACTTAACAGGAAGTGGGGCCATAGCGGGTGCTTACTCAAAGGCATACAATGAAACTTTTACTTTAACCTTTGTTAGTGGAAGAACGGTTGGAATTGGTGCTTATCTTGCCCGCCTAGGTATGCGGTGCATACAGAGACTTGATCAGCCGATCATCTTGACTGGCTTCTCTACACTCAACAAGTTACTTGGGCGTGAGGTCTATAGCTCTCACATGCAACTGGGTGGCCCGAAAATCATGGGCACAAATGGTGTTGTTCATCTTACAGTCTCAGATGATCTTGAAGGCGTATCAGCAATTCTCAACTGGCTCAGCTACATTCCTGCTTACGTGGGTGGTCCTCTTCCTGTTCTTGCCCCTTTAGATCCACCGGAGAGAATTGTGGAGTATGTCCCAGAGAACTCTTGCGACCCACGAGCGGCTATAGCTGGGGTCAAAGACAATACCGGTAAATGGCTTGGAGGTATCTTTGATAAAAATAGTTTCATTGAGACTCTTGAAGGCTGGGCAAGGACGGTAGTGACTGGTAGAGCCAAGCTCGGGGGAATACCCGTTGGAGTTGTTGCAGTTGAGACACAGACTGTCATGCAGATCATCCCAGCCGATCCTGGACAGCTTGACTCTCATGAAAGAGTGGTTCCGCAAGCAGGGCAAGTCTGGTTTCCTGATTCAGCGGCCAAGACTGCTCAAGCGCTTATGGATTTCAACCGGGAAGAGCTTCCATTGTTTATCCTAGCGAACTGGAGAGGGTTTTCAGGTGGGCAGAGAGATCTTTTCGAAGGAATACTTCAGGCAGGTTCAACTATAGTAGAAAATCTGAGAACCTATCGTCAGCCAGTGTTTGTGTACATCCCAATGATGGGAGAGCTGCGCGGTGGAGCGTGGGTTGTTGTTGACAGCCAGATAAATTCGGATTATGTTGAAATGTATGCTGATGAAACAGCTCGTGGAAATGTGCTTGAGCCAGAAGGGACAATAGAGATAAAATTTAGAACAAAAGAGCTATTAGAGTGCATGGGAAGGTTGGACCAGAAGCTAATCAGTCTGAAAGCAAAACTGCAAGATGCCAAGCAAAGCGAGGCCTATGCAAACATCGAGCTTCTCCAGCAACAGATTAAAGCCCGAGAGAAACAGCTTTTACCAGTTTATATCCAAATCGCCACCAAATTTGCAGAACTTCATGACACTTCCATGAGAATGGCTGCAAAGGGAGTGATCAAAAGTGTTGTGGAATGGAGCGGCTCGCGGTCCTTCTTCTACAAAAAGCTCAATAGGAGAATCGCTGAGAGCTCTCTTGTGAAAAACGTAAGAGAAGCATCT‑‑‑GGAGACAACTTAGCATATAAATCTTCAATGCGTCTGATTCAGGATTGGTTCTGCAACTCTGATATTGCAAAGGGGAAAGAAGAAGCTTGGACAGACGACCAAGTGTTCTTTACATGGAAGGACAATGTTAGTAACTACGAGTTGAAGCTGAGCGAGTTGAGAGCGCAGAAACTACTGAACCAACTTGCAGAGATTGGGAATTCCTCA‑‑‑GATTTGCAAGCTCTGCCACAAGGACTTGCTAATCTTCTAAACAAGGTGGAGCCGTCGAAAAGAGAAGAGCTGGTGGCTGCTATTCGAAAGGTCTTGGGT‑‑‑

>Bto_ACC_JETM‑2025864

ATGGCCGGCATCGGGCATGGGAAT‑‑‑‑‑‑‑‑‑GGATACATAAATGGCGTACTACCAAATAGGCATCCTGCTACAATATCTGAAGTAGATGAGTTCTGCCACGCACTTGGGGGAAAGAAGCCAATTCACAGCATATTAATAGCAAACAATGGAATGGCGGCTGTCAAATTTATTCGTAGCGTCAGGAGCTGGGCTTATGAGACATTTGGCACAGAGAAGGCTATCTTGTTGGTAGCCATGGCTACTCCAGAGGACATGAGAATCAATGCAGAACATATTAGAATAGCTGATCAATTTGTAGAAGTACCTGGTGGGACCAATAATAATAACTATGCCAATGTGCAGCTTATTGTAGAG‑‑‑‑‑‑‑‑‑ATGGCCGAGATAACTCATGTTGATGCAGTATGGCCTGGTTGGGGTCACGCATCTGAGAATCCTGAACTGCCAGATGCATTAAATGCAAAAGGAATTGTTTTTCTTGGCCCTCCTTCCATATCTATGGCAGCATTGGGAGATAAAATTGGTTCATCATTAATTGCTCAAGCAGCAGATGTGCCCACTCTACCATGGAGTGGTTCTCATGTGAAAATTCCTGCCGAAAGTTGCTTGGTAACGATACCTGATGAAATTTACCGGGAAGCATGTGTCTATACAACTGAGGAAGCAATTGCCAGTTGTCAAGTTGTGGGTTACCCTGCAATGATCAAGGCATCTTGGGGAGGTGGTGGCAAAGGCATAAGAAAGGTTCATAATGATGATGAGGTTAGGGCATTGTTCAAGCAAGTTCAAGGTGAAGTTCCAGGCTCACCTATATTTATAATGAAGGTTGCATCTCAGAGCCGGCATTTAGAAGTTCAATTACTTTGCGATCAGTATGGAAATGTTGCAGCTTTGCATAGCCGTGATTGCAGTGTTCAAAGGAGGCACCAAAAGATTATTGAGGAGGGACCAATCACTGTAGCTCCACTGGAGACAGTGAAAAAACTGGAACAGGCAGCTAGAAGATTAGCTAAATCTGTAAATTATGTTGGAGCAGCTACTGTTGAGTATCTATATAGTATGGAAACTGGCGAGTACTACTTTCTAGAGCTGAATCCCCGACTACAGGTTGAGCATCCTGTTACTGAGTGGATAGCAGAGATAAATCTCCCAGCTGCACAAGTTGCTGTTGGGATGGGCATCCCACTCTGGCAAATTCCTGAGATAAGGCGTTTCTATGGGGTAGAACATGGTGGAGGATATGATGCTTGGAGGAAAACATCCACTTTAGCTACTCCTTTTGATTTTGATAGAGCAGAATCTACAAGGCCGAAAGGTCATTGTGTGGCTGTGCGAGTGACAAGTGAGGATCCCGATGATGGTTTTAAGCCAACAAGTGGGAAAGTGCAGGAATTGAGCTTTAAAAGCAAGCCGAATGTGTGGGCATACTTCTCTGTTAAGTCTGGAGGAGGAATTCATGAATTTTCAGATTCTCAGTTTGGACATGTTTTCGCATTTGGAGAGTCCAGAGCTTTAGCTATTGCAAATATGGTTCTAGGGCTGAAGGAGGTTCAAATACGGGGAGAAATTCGTACCAATGTTGATTATACCATTGATCTTTTAAACGCTTCGGACTATAGAGAGAACAAAATTCACACTGGATGGTTGGACAGTAGAATAGCAATGCGGGTTAGGGCAGAAAGGCCCCCTTGGTATCTTTCTGTTGTTGGAGGAGCACTCTTTAAAGCCTCCACTAGCAGTGCAGCAATAGTTTCAGATTATGTTGGATATCTTGAAAAGGGACAAATCCCTCCCAAGCATATATCTCTTGTCCACTCTCAAGTGTCCTTGAACATTGAAGGAAGTAAATACACGATTGACATGGTAAGAGGAGGACCGGGAAGTTATAGATTGAGAATGAATGGTTCAGAAATAGAAGCAGAGATACATACATTACGTGATGGGGGTTTGCTGATGCAG‑‑‑‑‑‑‑‑‑TTGGATGGAAACAGTCATGTGATATATGCAGAGGAAGAAGCAGCAGGAACTCGCCTTCTAATAGATGGAAGGACTTGTTTGCTTCAGAATGATCATGATCCATCAAAGTTAGTTGCAGAGACACCATGCAAGCTTCTGAGATATTTGGTTATGGATGATAGTCATATTGATGCTGACACTCCGTATGCAGAAGTTGAGGTCATGAAGATGTGTATGCCTCTTCTCTCGCCTGCTTCTGGGTTTATTCATTTCAAGATGTCTGAAGGTCAAGCAATGCAGGCTGGTGAACTTATTGCGAGGCTTGATCTAGATGACCCATCAGCAGTAAGAAAAGCAGAACCTTTCCACGGGAGCTTCCCAGTCCTTGGTCCTCCTACTGCAATTTCTGGTAAAGTTCATCAGAAATGCGCTGCAAGTTTAAATGCAGCTAGAATGATCCTGGCTGGTTATGAGCACAATATTGATGAAGTTATGCAAAATTTGCTCAATTGCCTTGATAGTCCTGAACTGCCTTTCCTTCAATGGCAAGAGTGCTTGTCTGTTCTGGCAACCCGTCTTCCCAAAGATCTTAAAAATGAGTTGGAATCGAAATGTAAGGAATTTGAGAGGACTTCA‑‑‑AGCACCCAAAATGTTGATTTTCCTGCCAAATTGTTGAAGGGAATCCTTGAAGCTCATCTTTCCTCCTGTCCTGACAAAGAAAAAGGAGCCCAAGAAAGGCTTGTTGAGCCTCTTATGAGTCTGGTGAAGTCTTATGAGGGTGGAAGAGAAAGCCATGCCCGTGGAATTGTTCAATCCCTTTTTGAAGAATATCTTTCTGTTGAGGAACTTTTTAGTGATAACATTCAGGCTGATGTAATTGAACGGCTCCGACTTCAGTATAAGAAAGATTTATTGAAGGTTGTAGATATAGTACTCTCTCATCAGGGTGTCAGGAGTAAAAATAAGCTGATATTGCGACTGATGGAACAACTGGTGTATCCAAACCCTGCTGCCTACAGAGATCAATTGATCCGTTTCTCTGTACTCAACCATACAAATTATTCTGAGTTGGCACTTAAAGCGAGTCAACTATTGGAACAAACTAAATTGAGTGAACTTCGTTCCAGCATTGCTAGAAGTCTTTCTGAGCTAGAGATGTTCACTGAGGATGGTGAAAATATTGATACTCCGAAAAGGAAGAGTGCTATTAATGAAAGAATGGAGGACCTTGTCAGTGCTCCTTTGGCAGTTGAAGATGCTCTTGTAGGTTTGTTTGATCACGGTGATCACACCCTTCAAAGGCGTGTTATGGAGACCTACATCCGCAGGCTGTACCAGCCATATCTAGTAAGAGGGAGTGTCAGGATGCAGTGGCACAGATCTGGTCTTATTGCTACATGGGAGTTCTTGGAAGAGTACGTTGAGAAGAAGAAT‑‑‑GGAACTGAG‑‑‑‑‑‑‑‑‑‑‑‑TCTGATAAAACGCTAATGGAGAAACATAGTGAGAGGAAATGGGGAGTGATGGTTATAATTAAATCTCTTCAATTTTTGCCTGCAATTATCAGTGCCGCTTTAAGGGAAGCAACTCATAACCTTAACGAAGCA‑‑‑CTTCAAAATGGTCCTATGGAACCACTTAACCATGGCAATATGATGCACATTGCATTGGTGGGCATCAACAATCAGATGAGTTTACTGCAAGACAGTGGTGATGAGGATCAGGCTCAAGAAAGGATTAATAAGTTAGCCAAAATACTCAAAGACCAGGAAGTTGGTTCAACCATTCATACTGCAGGTGTTGGAGTAATTAGCTGTATTATACAGAGGGATGAAGGGCGAACCCCAATGAGGCATTCCTTTCACTGGTCAGCAGAAAAGTGCTATTATGAGGAGGAGCCTCTCTTGCGTCATCTGGAACCTCCCCTATCAATTTATCTTGAATTGGACAAACTTAAAGGCTATGAGAATATACGCTATACTCCATCCCGTGATCGTCAGTGGCACTTGTACACAGTTGTAGATCCCAAGCCACCATCAATTCAGAGAATGTTTCTTCGAACACTTGTAAGACAGCCAACCACAAATGAAGGATTCTCATCTTATCAAAGGCTGGATGTTGAAACATCTCGTACCCAGTTGGCTATGTCTTTTACTTCAAGGAGCATTTTGAGGCCCTTGATGGCTGCAATGGAGGAGTTAGAACTGAATGCACACAATGCCACTGTCAGATCTGAACATGCTCATATGTACCTTTATATCATACAGGAACAACGAATAGATGATCTTGTGCATTACTCCAAGAGAGTCAACATAGATGCCGGTCAAGAAGTAACAGCAGTTGAGGCAATCTTGGAAGAAATGGCGCATGAAATCCATTCATCTGTTGGTGTGAGAATGCATAGATTAGGTGTTGTTGAGTGGGAAGTCAAGCTTTGGATGGCATCCTCTGGAATGGCAGATGGTGCTTGGAGATTTGTTGTTACGAATGTGACTGGCCATACATGCACTGTACATATATACAGAGAAATGGAGGATTCTAGCAGTCGTAAAGTGATATATGGTTCAGTCACTGCAAAGGGTCCTTTACAAAGTTTACCAGTGAATGCACACTATCAACCTTTGGGAGTTCTTGACCGAAAACGTCTTTCAGCAAGGAAGAATAACACTACATACTGCTATGATTTTCCACTGGCATTTGAAAAGGCCTTGGAACAGTCATGGGTGGCCCAATACCCAGGATTCCAAAAGTCCAAGGATAAAGTTCTTCTAAAAGTAACAGAGATTAAATTTGCTGACAAAGAAGGCAGATGGGGTACTCCTCTGGTTCCTGTGGAGCGTGCACCTGGACTTAATGATGTTGGCATGGTAGCCTGGTTTATGGAAATGCGAACCCCTGAATTTCCATCTGGAAGGACCATATTGGTTGTAGCAAACGATGTAACCTTCAAGGCTGGTTCATTTGGCCCAAGAGAGGATGCATTCTATCGTGCAGTTACTGATCTTGCTATTCTGAAGAAACTACCTTTGATTTATCTGGCAGCAAATTCTGGTGCCCGTTTAGGTGTTGCTGAGGAAGTCAAAGCCTGTTTCAGAGTTGGGTGGTCTGATGAATCTAACCCTGAGAATGGTTTTCAGTATGTGTATTTAACTCCTGAGGATTATGCTAGGATTGGGTCATCAGTTATAGCACACGAGTTGAAGCTGGAAAGTGGAGAAACCAGATGGGTCATTGATACTATCGTTGGGAAAGAGGACGGCTTGGGGGTTGAAAACTTGAGTGGCAGTGGAGCCATTGCTGGTGCCTATTCAAGGGCATACAAGGAAACTTTTACATTGACATATGTGACTGGCAGGACTGTTGGAATAGGTGCTTATCTTGCCAGGCTTGGGATGAGATGTATACAAAAACTTGATCAGCCCATAATTCTCACTGGTTTTTCCGCATTAAATAAACTTCTTGGTCGGGAGGTTTACAGCTCTCACATGCAACTTGGTGGACCTAAAATCATGGCAACAAATGGAGTTGTTCATCTTACAGTTTCTGATGACCTTGAAGGTGTTTCTGCTATTTTGAAGTGGCTTAGTTATATCCCTTCTCATATAGGTGGTGCACTTCCCATTCTACGGTCCCTTGATCCTCCAGAAAGGCCTGTGGAGTATTTCCCTGAGAACTCATGCGATCCTCGTGCTGCTATTTCTGGTGCTCTGGATGGTAGTGGAAGATGGCTGGGGGGTATTTTTGACAAGGACAGCTTTGTTGAGACGCTAGAAGGATGGGCAAGAACAGTTGTTACAGGAAGGGCAAAGCTTGGAGGAATCCCTGTGGGAATTATTGCCGTTGAAACACAAACAGTGATGCAAGTAATACCTGCGGATCCGGGCCAGCTTGATTCTCATGAGAGGGTTGTTCCTCAGGCCGGGCAAGTTTGGTTTCCTGATTCTGCAACTAAGACAGCCCAGTCAATATTAGATTTCAACAAAGAGGAGCTCCCACTTTTCATTCTTGCAAATTGGAGAGGCTTTTCAGGTGGGCAGAGGGACCTTTTTGAAGGAATCCTTCAAGCTGGATCGACCATTGTAGAGAACCTCAGAACATACAAGCAGCCAATATTTGTATATATCCCAATGATGGGTGAACTCCGAGGTGGGGCATGGGTTGTTGTAGACAGTAGAATCAATTCAGACCACATTGAAATGTATGCCGAGCGAACAGCTAAAGGCAATGTCCTTGAGCCAGAAGGAATGATCGAGATCAAATTTAGGACAAAAGAATTGTTGGAGACCATGGGTAGACTTGATCAACAATTGATAAATCTCAAGGAACAACTTCAGGAGGCCAAGAATAATAGAATCTATGAGACCATTGAATCCCTACAGCAGCAGATCAAGTTCCGTGAGAAACAACTTCTGCCGTTGTACACCCAGATAGCTACTAGGTTTGCTGAATTGCATGATACTTCCCTTAGAATGGCAGCAAAGGGTGTAGTAAGAGAAGTTTTGGACTGGAGTAACTCCCGTTCCTTCTTCTACAAGAGACTGCACAGGAGAATTGGTGAGCAGTCAATGATCAACAGTGTGAAAAATGCTGCC‑‑‑GGTGACCATTTATCACATATAGCTGCAGCGGACTTGATCAAAAACTGGTATTTGGATTCTGAAATTGCCAAAGGTAAAGATGATGCTTGGTTAGATGATGAGGCTTTCTTCAGATGGAAGGATGATCCTAAGTACTACGAGGATAAACTCAAGGAATTGCATGTCCAGAAGGTATTGCTTCAATTGACAAATATTGGTGACTCAGTTTCTGATTTGCAAGCTTTACCTCAAGGTCTTGCTGCCCTTTTAAGCAAGTTGGAGACATCAAGCCGTGTGAAATTAACTGATGAACTTCGCAAGGTACTTGGC‑‑‑

>Car_ACC_Ca_16623

ATGGCTGGTGTCGGGCGTGGGAACAGTGGGAGTGGATATATAAACGGCGTGAACCCAAATAGGCACCCTGCTACAATTTCTGAAGTAGACGAGTTCTGCAATGCTCTTGGGGGAAACAGGCCGATTCATAGTATATTGATTGCAAACAATGGAATGGCAGCGGTCAAGTTTATACGTAGTGTTAGGAGTTGGGCATATGAGACATTTGGCACAGAAAAGGCTATCTTGTTGGTTGCCATGGCTACTCCAGAGGACATGAGAATCAATGCAGAACATATCAGAATAGCTGATCAGTTTGTCGAAGTACCTGGTGGTACCAATAACAATAATTATGCCAATGTGCAGCTTATTCTTGAG‑‑‑‑‑‑‑‑‑ATTGCCGAGATAACTCATGTTGATGCGGTGTGGCCTGGTTGGGGTCATGCATCTGAGAATCCTGAGCTTCCAGATGCATTAAAAGCAAAAGGAATTGTATTTCTTGGACCTCCTGCAGTATCTATGGCAGCATTGGGAGATAAAATTGGTTCTTCGTTGATTGCTCAAGCAGCAGAAGTGCCAACCCTTCCATGGAGTGGTTCTCATGTGAAAATTCCTCCTGAAAGCTCCTTGATTACTATTCCTGATGAAATTTACCGGGCAGCATGTGTTTATACAACAGAAGAAGCAGTTGCAAGTTGTCAAGTTGTAGGGTACCCTGCAATGATCAAGGCGTCTTGGGGTGGTGGTGGTAAAGGCATAAGAAAGGTTCATAATGATGATGAGGTAAGGGCATTGTTCAAGCAAGTTCAAGGTGAAGTTCCGGGCTCACCTATATTTATAATGAAAGTTGCTTCCCAGAGCCGGCATCTTGAAGTCCAGTTAATTTGTGATCAGTATGGAAACGTTGCGGCTTTGCACAGCCGTGATTGCAGTGTTCAAAGAAGGCACCAAAAGATTATTGAGGAGGGTCCCATTACTGTAGCACCTCCAGAAACGGTGAAAGAACTTGAACAGGCAGCTAGAAGATTGGCTAAATCTGTAAATTATGTTGGTGCAGCTACTGTTGAGTATCTTTATTGCATGGAAACTGGCGAGTACTACTTTTTAGAGTTGAACCCCAGACTACAGGTTGAGCATCCTGTTACTGAATGGATAGCCGAGATAAATCTGCCAGCAGCACAAGTTGCCATTGGGATGGGTATCCCTCTATGGCAAATTCCTGAGATTAGGCGTTTCTATGGGGTGGAACATGGTGGTGGGAATGATGCTTGGAGGAAAACATCAGTTTTAGCTACCCCTTTTGATTTTGACAAAGCAGAATCTACAAGGCCAAAAGGTCATGTTGTGGCTGTGCGAGTGACTAGCGAGGACCCCGATGACGGTTTTAAGCCTACAGGCGGGAAAGTGCAGGAGCTCAGCTTTAAAAGCAAACCAAATGTGTGGGCATATTTCTCTGTTAAGTCTGGAGGAGGAATCCATGAATACTCAGATTCTCAGTTTGGACATGTTTTTGCTTTTGGAGAATCTAGGGCTTTAGCAATTGCAAATATGGTTTTGGGGCTGAAGGAAATCCAAATTCGTGGAGAAATCCGGACCAATGTTGATTACACAATTGATCTTCTGAATGCTGCAGACTACAGAGAAAACAAATTTCACACTGGTTGGTTGGACAGTAGAATTGCAATGCGTGTTAGAGCAGAGAGGCCTCCTTGGTATCTGTCTGTTGTTGGAGGGGCACTCTATAAAGCTTCTGCCAGCAGTGCAGCTTTGGTTTCAGACTATGTTGGCTATCTTGAAAAGGGACAAATCCCTCCTAAGCGCATATCTCTTGTCCATTCTCAAGTGTCCTTGAGCATTGAAGGAAGCAAATATACGATTGACATGATACGAGGAGGACCTGGAAGTTATAAATTGAAATTGAATCAATCTGAGATAGAAGCAGAGATACATACTTTACGTGATGGAGGTTTGCTGATGCAGGCAAGT‑‑‑TTGGATGGAAACAGTCATATATTATACGCAGAAGAAGAAGCAGCAGGAACTCGTCTTCTAATAGATGGAAGGACTTGCTTACTTCAGAATGATCACGATCCATCAAAGTTAATTGCTGAGACACCATGCAAGCTTATGAGATATTTGGTTGCGGATGACAGTCACATTGATGCGGACACACCGTACGCTGAGGTTGAGGTCATGAAGATGTGCATGCCTCTTCTTTCCCCTGCTTCTGGGATTATTCATTTTAGAATGGCCGAAGGTCAAGCCATGCAGGCTGGTGAACTTATAGCAAGGCTTGATCTAGATGATCCTTCTGCTGTAAGAAAGGCAGAACCCTTCAATGGGAGCTTCCCAATCCTAGGCCCTCCAGCTGCAATTTCAGGTAAAGTTCATCAGAAATGTGCAGCAAGCTTAAATGCTGCACGGATGATTCTTGCTGGCTATGAGCACAATATCGATGAAGTTGTTCAAAATTTGCTGAATTGCCTTGATAGCCCTGAATTGCCTTTCTTTCAATGGCAAGAGGTATTGGCAGTTCTGGCAACCCGTCTTCCCAAAGATCTAAGAAATGAGTTGGAAGCAAAATATAAGGAGTTCGAAAGTATTTCA‑‑‑AGCTCACAAAATATTGATTTCCCTGCCAAATTATTGAAGGGAATTCTTGAAGCTCATCTTTCCTCCTCTCCTGAAAGAGAAAAAGGAGCCTTAGAAAGACTAGTTGAACCTCTGATGAGTCTTGTAAAGTCTTATGAGGGTGGAAGAGAGAGCCATGCCCATAAAATTGTTCAATCTCTTTTTGAAGAGTATCTTTCAGTCGAAGAACTATTTAGTGACAATATACAGGCTGATGTAATTGAACGACTCCGTCTTCAATACAAGAAGGATTTGTTGAAGATTGTAGATATTGTGCTCTCTCATCAGGGTGTCAAGAGCAAAAATAAGCTGATACTGCGACTAATGGATAAACTGGTTTACCCTAACCCTGCTGCCTACAGAGATCAATTGATCCGATTCTCCACACTAAACCATACAATTTATTCTGAGTTGGCTCTCAAGGCAAGTCAGCTGCTAGAACAAACTAAATTGAGTGAACTTCGATCCAGCATTGCTAGAAGTCTTTCTGAACTTGAAATGTTTACTGAGGACGGTGAAAATATTGATACTCCTAAGAGGAAGAGTGCCATTAATGACCGAATGGAGGACCTTGTGAGTACTCCTTTGGCAGTTGAAGATGCCCTTGTGGGTTTATTTGATCACAGTGATCACACCCTTCAAAGGAGGGTTGTGGAAACTTATATTCGTAGGCTCTACCAGCCATATCTTGTCAAAGAGAGCGTCAGGATGCAGTGGCATCGATCTGGCCTTATTGCTACGTGGGAGTTCTTAGAAGAGCACGTTGAACGGAAGAAT‑‑‑GACGTTGACGAC‑‑‑CAGACATCGGAAAAAACAGTGGTCGAGAAACGTAGTGAGAAGAAATGGGGAGTGATGGTTGTAATTAAATCTCTTCAATTTTTGCCAGCAATTATCAGCGCTGCATTAAGGGAAGCAACCAATAACTTTAACAAAGCA‑‑‑CTTAGAAGTGGTTCTGGTGACTCAAGTACACATGGTAATATGATGCATATTGGATTAGTGGGAATCAACAACCAAATGAGTTTACTTCAAGACAGTGGTGATGAGGATCAGGCTCAAGAAAGAATCAATAAGTTGGCCAAAATACTCAAAGATCCGGAAGTAGGCTCCACAATACATGCTGCAGGTGTTGGAAATATTAGCTGTATCATACAAAGGGATGAAGGGCGAGCCCCGATGAGGCATTCATTTCACTGGTCTGCTGAAAAGCTATATTATGAAGAGGAACCTCTGCTGCGTCATCTGGAACCTCCCCTATCCATTTATCTTGAATTGGACAAACTTAAAGGCTATGAGAATATACGGTATACACCATCCCGAGATCGTCAATGGCACCTCTACACGGTTTTGGATAGCAAGCCACAACCAATTCAAAGAATGTTTCTTAGAACACTTTTAAGACAGCCAACCACAAATGAAGGATTCTCTTCTTATCAAAGGCTGGATGCAGAAACTTCTCGTGCACAATTGGCTATGTCCTTTACTTCAAGGAGCATTTTTAGGTCCCTGATGGGTGCAATGGAGGAGTTGGAACTTAATTCACACAATGCCACCATCAAATCCGAACATGCCCATATGTACCTCTATGTCATCCGTGAGCAGCAAGTAGATGATCTTGTGCCTTTTCCCAAGAAAATTAACATAGATGCTGGTCAAGAAGAAACAACAGTTGAGGCAATCTTGGAAGAACTGGCACAGGAAATCCATTCGTCTGTCGGTGTAAGAATGCATAGATTAGGGGTTTTTGTATGGGAAGTCAAGCTCTGGATTGCAGCATGTGGACAGGCAAATGGTGCTTGGAGGATCATTGTAAACAATGTGACTGGTCATACATGCACTGTACATATATATCGAGAAGTGGAGGACGCTACCACTCATAAAGTGGTCTACAGTTCAGTCACTGCAAAGGGTCCACTGCATGGTGTACCAGTGAACGAAAACTATCAACCTTTGGGAGTTATTGACCGAAAACGTCTTGCAGCAAGAAAGAGCAGCACCACATACTGCTATGATTTTCCCCTTGCATTTAAAACATCCTTGGAACAGTCATGGTCAATTCAACAGACAGGAATTCAAAGAGCCAAAAATAAGGATCTCATAAAAATAACAGAGCTTAAATTTTTAGAGAAAGAAGGTAGTTGGGGTACTCCTCTTGTTCCTGCGGAGCGTCCTCCTGGACTCAACGATGTTGGCATGGTAGCCTGGTCCATGGAAATGTGTACCCCTGAATTTCCATCTGGAAGGACCATATTGGTTGTTTCAAATGATGTGACCTTCAAGGCTGGTTCTTTTGGCCAAAGAGAGGATGCATTCTTTAAAGCAGTAACTGATCTTGCCTGTGCAAAAAAATTACCTTTAATTTACTTAGCGGCAAATTCTGGTGCTCGTTTAGGTGTAGCCGAGGAAGTCAAAGCTTGTTTCAGAGTTGGTTGGTCCGAGGAATCTAACCCCGAACATGGTTTTCAGTATGTATATTTAACACCTGAGGATTATGCTCGAATTGGATCGTCCGTGATAGCACATGAATTAAAGCTTGAAAGTGGAGAAACCAGATGGGTTATAGATACCATTGTTGGTAACGAGGATGGACTGGGAGTTGAAAACTTGAGTGGAAGTGGGGCCATTGCCGGTGCCTATTCAAGAGCATACAAGGAAACATTTACATTGACATATGTGACCGGTAGGACTGTTGGAATTGGTGCTTATCTTGCTAGGCTTGGAATGAGATGCATACAGAGGCTTGATCAGCCTATAATTCTTACCGGATTTTCAGCATTAAACAAACTTCTCGGTCGGGAGGTTTACAGCTCTCACATGCAACTTGGTGGACCTAAAATCATGGCAACTAATGGAGTTGTTCATCTTACAGTTTCGGACGACCTTGAAGGTGTTTCTTCTATTTTGAAGTGGCTTAGCTACATTCCTTCTCATATAGGTGGTGCACTTCCCATTCTAAAGCCCCTCGATCCCCCAGAGAGGGAAGTGGAGTATTTACCGGAAAATTCATGCGATCCTCGTGCTGCCATTTCTGGAACTCTGGATGTTAGTGGAAAATGGCTTGGAGGCATTTTTGACAAGGACAGCTTTGTGGAGACACTAGAAGGATGGGCAAGGACCGTTGTTACAGGAAGGGCAAAGCTTGGAGGAATCCCTGTCGGAATCGTTGCTGTAGAAACACAAACAGTTACGCAAATAATACCTGCTGATCCAGGTCAGCTTGATTCTCATGAGAGGGTTGTTCCTCAGGCTGGGCAAGTGTGGTTTCCCGATTCTGCGACCAAGACAGCCCAAGCAATATTGGATTTCAACAGAGAAGAGCTTCCGCTTTTCATTCTCGCAAATTGGAGAGGCTTTTCGGGTGGGCAAAGGGACCTTTTTGAAGGAATTCTTCAGGCCGGTTCAACTATTGTGGAAAACCTTAGAACATACAAGCAGCCCATATTTGTATACATACCAATGATGGGTGAACTCCGTGGCGGGGCTTGGGTTGTTGTTGACAGCCGAATCAACTCAGACCACATTGAAATGTATGCCGAGCGAACAGCTAAAGGTAACGTCCTTGAGCCAGAAGGAATGATTGAGATCAAATTTAGAACAAGAGAATTGTTGGAGTGTATGGGAAGACTTGATCAACCATTGATTACTTTGAAGGCAAAACTTCAGGAAGCCAAGAGTAACAAGGACTATGGTACAATTGATTCTCTGCAGCAGCAAATTAGATTCCGCGAAAAACAAATTTTACCTTTGTATACCCAGATAGCTACCAAATTTGCTGAACTGCATGACACTTCCTTAAGAATGGCTGCAAAGGGTGTAATCAGAGAAGTTCTGGACTGGCGTAACTCCCGTGCTGTCTTCTACCGGAGACTGCACAGGAGAATTGGCGAGCACTCGCTGATCAACAGTGTGAGAGATGCTGCT‑‑‑GGTGACGAATTGTCATATGTATCTGCCATGAACTTGCTCAAAAACTGGTATCTGAATTCTGATATCGCGAAAGGTAGAGAAGATGCTTGGTTGGATGATGAAGCCTTCTTCAGATGGAGGAATGATCCCGCAAATTACGAGGATAAACTAAAGGAGTTGCGCGTCCAGAAACTGTTGCTGCAGTTGACAAACATTGGCGACTCAGATCTAGATTTACAAGCTCTACCTCAAGGTCTTGCCGCCCTTTTAAGCAAGTTGGAAGGATCAAGTCGCAATAAGTTGACCAATGAGCTTCGCAAGGTACTTGGT‑‑‑

>Cca_ACC_C.cajan_25715

ATGGCTGACATTGGGCGTCGGAAT‑‑‑‑‑‑‑‑‑GGATACGCAAACAGTCTACTACCAAATAGGCACCCTGCTGCAATATCTGAAGTAGACGAGTTCTGCAAGGCCCTTGGCGGAAACAGGCCAATTCACAGCATATTAATTGCCAACAATGGAATGGCAGCAGTCAAGTTTATACGTAGTGTTAGGGGCTGGGCCTATGAGACATTTGGCACAGAGAAAGCCATCTTGTTGGTTGCCATGGCTACTCCAGAGGACATGAGAATCAATGCAGAACATATCAGAATGGCTGATCAATTTGTAGAAGTACCTGGTGGGACCAATAACAATAACTATGCCAATGTGCAGCTTATTCTTGAG‑‑‑‑‑‑‑‑‑ATGGCCGAGATAACACATGTGGATGCAGTGTGGCCTGGTTGGGGTCATGCATCAGAAAATCCTGAGCTTCCAGATGCGTTAAAAGCAAAAGGAATTGTATTTCTCGGACCTCCTGCCATATCTATGGCTGCATTGGGAGATAAAATTGGTTCATCATTGATTGCTCAAGCAGCGGATGTGCCAACCCTTCCATGGAGTGGTTCTCATGTGAAAATTCCGCCTGAAAGTTCCTTGATTACTATTCCTGATGAAATTTATCGGGAAGCATGTGTTTATACAACAGAGGAAGCAATTGCGAGTTGTCAAGTTGTAGGATACCCGGCAATGATCAAGGCATCTTGGGGTGGTGGTGGTAAAGGCATAAGAAAGGTTCATAATGATGATGAGGTAAGGGCATTGTTCAAGCAAGTTCAAGGTGAAGTTCCGGGTTCACCTATATTTATCATGAAAGTTGCATCCCAGAGCCGACATCTAGAAGTCCAGTTACTTTGTGATCAGTATGGAAATGTTGTGGCTTTGCATAGCCGTGATTGCAGTGTTCAAAGGAGGCACCAAAAGATTATTGAGGAGGGTCCTATTACTGTAGCACCAATAGAAACGGTGAAAAAACTGGAACAGGCAGCTAGAAGGTTGGCTAAATCTGTAAATTATGTAGGGGCGGCTACTGTTGAGTATCTTTTTAGTATGGAAACTGGCGAGTACTACTTTTTAGAGTTGAACCCCCGGCTACAGGTTGAACATCCTGTTACTGAGTGGATAGCAGAGGTAAATCTGCCAGCAGCACAGGTTGCCATTGGGATGGGTATCCCTCTCTGGCAAATACCGGAGATAAGGCGTTTCTATGGGGTGGAACATGGTGGGGGTAATGATGCTTGGAGAAAAACATCAGTTTTAGCTACCCCTTTTGATTTTGACAAAGCAGAATCTACAAGGCCAAAAGGTCATTGTGTAGCTGTGCGAGTGACTAGTGAAGACCCTGATGATGGTTTTAAGCCTACAAGTGGGAAAGTGCAGGAGCTTAGCTTTAAAAGCAAACCAAATGTTTGGGCATACTTCTCTGTCAAGTCTGGAGGAGGAATTCATGAATTCTCAGATTCACAGTTTGGGCATGTTTTCGCTTTTGGAGAATCTAGGGCTTTAGCAATTGCAAATATGGTTCTGGGGCTGAAGGAGATTCAAATTCGTGGGGAAATCCGAACCAATGTTGATTACACCATTGATCTTCTGAATGCTTCAGACTACAGAGAAAACAAAATTCACACAGGTTGGCTAGACAGTAGAATTGCAATGCGGGTTAGAGCAGAGAGGCCTCCCTGGTATCTTTCTGTTGTTGGAGGGGCACTCTATAAAGCTTCTGCTAGCAGTGCAGCTTTGGTTTCAGACTATGTCGGCTATCTTGAAAAGGGACAAATCCCTCCCAAGCACATATCTCTTGTCCATTCTCAAGTTTCCTTGAACATTGAAGGAAGCAAATATACGATTGACATGATACGAGGTGGGCCTGGAAGTTATAGATTGAAAATGAATCAATCAGAGATAGAAGCAGAAATACATACTTTACGTGATGGAGGTTTGCTAATGCAG‑‑‑‑‑‑‑‑‑TTGAATGGAAACAGTCATGTATTATATGCAGAGGAAGAAGCAGCTGGAACTCGCCTTCTTATAGATGGAAGGACTTGCTTGCTTCAGAATGATCATGATCCATCAAAGTTGGTTGCGGAGACACCATGCAAGCTTCTGAGATATTTGGTTACAGATGACAGTCATGTTGATGCTGACACACCATATGCTGAAGTTGAGGTCATGAAAATGTGCATGCCTCTTCTTTCCCCTGCTTCTGGGATTATTCATTTCAAAATGTCTGAAGGTCAAGCAATGCAGGCTGGTGAACTTATAGCAAGACTTGATCTAGATGATCCTTCAGCTGTACGGAAGGCAGAACCCTTCACTGGGAGCTTCCCAGTCCTGGGACCTCCTACTGCAATTTCTGGTAAAGTTCATCAGAAATGTGCTGCAAGCTTAAATGCTGCACGGATGATTCTTGCTGGGTATGAGCACAATATTGATGAAGTTGTGCAAAGTTTGCTTAATTGCCTTGATAGTCCAGAATTGCCTTTTCTTCAATGGCAAGAGTGCTTTGCAGTTTTAGCAACCCGTCTTCCCAAAGATCTTAAAAATGAGTTGGAATCAAAATACAAGGAATTTGAGAAGATTTCA‑‑‑AGCTCTCAAGTTGTTGATTTCCCTGCCAAATTATTGAAGGGAATTCTTGAAGCTCATCTTTCCTCCTGTCCTGACAAAGAAAAAGGAGCCCTAGAAAGACTAGTTGAACCTCTAATGAGTGTTGTTAAGTCTTATGAGGGTGGAAGAGAGAGCCATGCCCATATAATTGTTCAATCCCTTTTTGAAGAGTATCTTTTTGTTGAAGAATTGTTTAGTGATAATATACAGGCTGATGTGATTGAACGTCTTCGTCTTCAATACAAGAAAGATCTATTGAAGATTGTAGATATAGTGCTCTCTCATCAGGGCATCAAGAGTAAAAATAAGCTGATATTGCGACTAATGGATAAATTGGTTTACCCAAACCCTGCTGCTTACAGGGATCAGTTAATCCGTTTCTCAGTACTCAACCATACAAATTATTCTGAGTTGGCTCTAAAAGCAAGTCAACTGCTGGAACAAACTAAATTAAGTGAACTTCGATCCAACATTGCTAGAAGTCTTTCTGAACTAGAGATGTTCACAGAGGATGGTGAAAATATTGATACTCCCAAGAGGAAGAGTGCCATTAATGACCGAATGGAGGACATTGTAAGTGCTCCTTTGGCGGTTGAAGATGCCCTTGTGGGTTTATTTGATCACAGTGATCATACCCTTCAGAGGAGGGTTGTGGAAACTTATGTTCGCAGGCTTTACCAGCCATATCTTGTCAAAGGGAGTGTCAGGATGCAATGGCACAGATCTGGTCTTATTGCTACATGGGAGTTTTATGATGAGTACATTGAAAGGAAGAAT‑‑‑GGAGTTGAAGAC‑‑‑CAAACTTCAAATAAAACAGTGGTGGAGAAACATAGTGAGAAAAAGTGGGGAGTGATGGTTATAATTAAATCTCTTCAATTTTTGCCAGCAATTATCAGTGCTGCTTTAAGGGAGGCAACCAATAACCTTCATGAAACA‑‑‑CTTACAAGTGGTTCTGTGGAACCAGTTAACTATGGTAATATGATGCATATTGGATTAGTGGGCATCAACAACCAGATGAGTTTACTGCAAGACAGTGGTGATGAGGATCAGGCTCAAGAAAGAATCAATAAGTTAGCTAAAATACTGAAAGAGCAGGAAGTAGGCTCCACCATACGAGCTGCAGGTGTTGGAGTAATCAGCTGTATCATACAAAGGGATGAAGGACGTGCCCCAATGAGGCACTCGTTTCACTGGTCAGCAGAAAAGCTGTACTATGAGGAGGAACCTCTGTTGCGTCATCTGGAACCTCCACTATCCATTTATCTTGAATTGGACAAACTTAAAGGCTATGAGAATATACGGTATACTCCATCTCGAGATCGTCAATGGCACCTGTACACAGTTGTGGATCACAAGCCACTACCAATTCAAAGAATGTTTCTTCGTACACTTCTAAGACAGCCAACCACAAATGAAGGATTCTCTTCGTATCAAAGGCTGGATGCTGAAACATCTCGAACCCAATTGGCTATGTCTTTTACTTCAAGGAGCATTTTTAGGTCTTTGATGGCTGCAATGGAGGAGTTGGAACTTAATGCACACAATGCCAATATCAAATCTGAACATGCTCATATGTACCTCTATATCATACGTGAGCAACAAATAGATGATCTTGTGCCGTATCCCAAGAAAATTAACATAGATGCTGGGAAAGAAGAAACTACAGTTGAGGCAGTCTTGGAAGAACTGGCCCGGGAAATCCATTCATCTGTTGGTGTAAGAATGCATAGATTAGGGGTTGTTGTGTGGGAAGTCAAGCTCTGGATGGCAGCCTGTGGACAAGCAAATGGTGCTTGGAGGGTCATTGTAAACAATGTGACTGGTCATACATGCACTGTACATATATACCGAGAAAACGAGGATGCTGACACTCATAAAGTGGTATACAGTTCAGTCTCAGTTAAGGGTCCACTGCATGGTGTGCCAGTGAATGAAAATTATCAACCTTTGGGAGTTATTGACCGAAAACGTCTTTCAGCAAGAAAGAACAGCACCACTTATTGCTACGATTTTCCTCTTGCATTTAAAACAGCCTTGGAACAGTCATGGGCAATCCAACAACCAGGATTTCAAAGACCCAAAGATAAAAATCTTCTAAAGGTAACAGAGCTTAAGTTTGCTGACAAAGAAGGTAGCTGGGGTACTCCTCTTGTTCCTGTAGAGCGTTACCCTGGATTCAATGATGTTGGCATGGTAGCCTGGTTTATGGAAATGTGTACTCCTGAGTTCCCAACTGGAAGGACAATATTGGTTGTTTCAAACGATGTGACCTTCAAGGCTGGTTCTTTTGGCCCAAGAGAGGATGCATTCTTCCGAGCAGTAACTGATCTTGCGTGTACAAAAAAACTACCTTTAATTTATTTAGCAGCAAATTCTGGTGCTCGTTTAGGTGTAGCTGAGGAAGTCAAATCCTGTTTCAGAGTTGGTTGGTCTGAGGAATCTAATCCTGAGCATGGCTTTCAGTATGTATATTTAACGCCTGAGGATTATTCTCGGATTGGATCATCAGTGATCGCACATGAATTAAAGCTTGAGAGTGGAGAAACCAGATGGGTTATAGACACCATTGTTGGCAAGGAGGATGGCCTTGGGGTTGAGAACTTGAGTGGAAGTGGGGCTATTGCCGGTGCTTATTCAAGAGCATACAAGGAAACTTTTACATTGACATATGTGACTGGTAGAACTGTTGGAATAGGTGCTTATCTTGCTAGGCTTGGGATGAGATGCATACAGAGGCTTGATCAGCCCATAATTCTTACTGGTTTTTCAGCATTAAACAAGCTTCTTGGTCGGGAGGTATACAGCTCTCACATGCAACTTGGTGGACCTAAAATCATGGCAACTAATGGAGTAGTTCATCTTACAGTTTCTGATGATCTCGAAGGTGTTTCTTCTATTTTGAAGTGGCTTAGCTACATTCCTTCCCATGTAGGCGGTGTGCTTCCCATTGTAAAGCCCCTTGATCCTCCAGAAAGACCGGTAGAGTATTTCCCAGAAAACTCGTGTGATCCTCGTGCTGCCATTTCTGGAACCCTGGATGGTAATGGAAGATGGCTGGGAGGCATTTTTGACAAGGACAGCTTTGTGGAGACACTAGAGGGATGGGCAAGAACAGTTGTTACAGGAAGGGCAAAGCTTGGAGGCATCCCTGTTGGAATTGTTGCTGTAGAAACGCAGACAGTGATGCAAATAATACCTGCTGATCCAGGCCAGCTCGATTCTCACGAGCGCGTTGTTCCTCAAGCTGGGCAAGTTTGGTTTCCTGATTCTGCAACCAAGACAGCCCAAGCAATATTGGATTTCAACAGAGAAGAGCTCCCGCTTTTCATTATGGCAAACTGGAGAGGCTTTTCAGGAGGGCAAAGGGACCTTTTTGAAGGAATTCTTCAGGCCGGATCAACTATTGTGGAGAACCTTAGAACATACAAGCAGCCCATATTTGTATACATTCCAATGATGGGTGAGCTTCGTGGCGGGGCATGGGTTGTCGTTGACAGTCGAATCAATTCAGACCACATTGAAATGTATGCCGAGCGAACGGCTAAAGGTAATGTCCTTGAGCCAGAAGGAATGATCGAGATCAAGTTTAGAACAAGAGAATTGTTGGAGTGCATGGGTAGACTTGATCAGCAGTTGATAACTCTGAAGGCAAATCTTCAGGAAGTCAAGAGTAACTGGGACACTACCACCTTTGAATCCCTACAGCAGCAGATTAAATCGCGTGAGAAACAGCTTTTGCCTGTGTATACCCAGATAGCTACCAAGTTTGCTGAACTGCATGATACTTCCTTAAGAATGGCTGCCAAGGGTGTCATTAGAGAAGTTCTGGACTGGCGTAACTCCCGCACCGTCTTCTACCGGAGACTGTACAGGAGAATTGGTGAGCAGTCACTGATCAACAGTTTGAGAGATGCTGCT‑‑‑GGTGACCATTTGTCACATGCATCTGCACTGAACATGCTCAAAGAATGGTATTTGAATTCTGATATTGCCAACGGTAAAGAAGGTGCTTGGTTGGATGATGAAGCCTTCTTCAGATGGAAGAATAATCCTGCAAATTTCGAGAATAGGCTAAAGGAGTTGCGAGTGCAGAAAGTGTTGCTTCAATTGACAAATATTGGTGACTCGGCTCTAGATTTGCAAGCTCTACCACAGGGTCTTGCTGCCCTTTTAAGCAAGTTGGAGCCATCAGGTCGTGTGAAGTTGACTGATGAACTTCGCAAGGTACTTGGT‑‑‑

>Ccan_ACC_RKFX‑2008745

‑‑‑‑‑‑‑‑‑‑‑‑‑‑‑‑‑‑‑‑‑‑‑‑‑‑‑‑‑‑‑‑‑‑‑‑‑‑‑‑‑‑‑‑‑‑‑‑‑‑‑‑‑‑‑‑‑‑‑‑‑‑‑‑‑‑‑‑‑‑‑‑‑‑‑‑‑‑‑‑‑‑‑‑‑‑‑‑‑‑‑‑‑‑‑‑‑‑‑‑‑‑‑‑‑‑‑‑‑‑‑‑‑‑‑‑‑‑‑‑‑‑‑‑‑‑‑‑‑‑‑‑‑‑‑‑‑‑‑‑‑‑‑‑‑‑‑‑‑‑‑‑‑‑‑‑‑‑‑‑‑‑‑‑‑‑‑‑‑‑‑‑‑‑‑‑‑‑‑‑‑‑‑‑‑‑‑‑‑‑‑‑‑‑‑‑‑‑‑‑‑‑‑‑‑‑‑‑‑‑‑‑‑‑‑‑‑‑‑‑‑‑‑‑‑‑‑‑‑‑‑‑‑‑‑‑‑‑‑‑‑‑‑‑‑‑‑‑‑‑‑‑‑‑‑‑‑‑‑‑‑‑‑‑‑‑‑‑‑‑‑‑‑‑‑‑‑‑‑‑‑‑‑‑‑‑‑‑‑‑‑‑‑‑‑‑‑‑‑‑‑‑‑‑‑‑‑‑‑‑‑‑‑‑‑‑‑‑‑‑‑‑‑‑‑‑‑‑‑‑‑‑‑‑‑‑‑‑‑‑‑‑‑‑‑‑‑‑‑‑‑‑‑‑‑‑‑‑‑‑‑‑‑‑‑‑‑‑‑‑‑‑‑‑‑‑‑‑‑‑‑‑‑‑‑‑‑‑‑‑‑‑‑‑‑‑‑‑‑‑‑‑‑‑‑‑‑‑‑‑‑‑‑‑‑‑‑‑‑‑‑‑‑‑‑‑‑‑‑‑‑‑‑‑‑‑‑‑‑‑‑‑‑‑‑‑‑‑‑‑‑‑‑‑‑‑‑‑‑‑‑‑‑‑‑‑‑‑‑‑‑‑‑‑‑‑‑‑‑‑‑‑‑‑‑‑‑‑‑‑‑‑‑‑‑‑‑‑‑‑‑‑‑‑GATAAAATTGGTTCGTCATTAATTGCTCAAGCAGCAGAAGTGCCAACTCTACCATGGAGTGGTTCTCATGTGAAAATTCCTGCTGATAGTTGCTTGGTTACGATACCTGATGAAATTTACCGGGAAGCATGTGTCTATACAACAGAGGAAGCAATTGCAAGTTGTCAAGTTGTAGGCTACCCCGCAATGATAAAGGCATCTTGGGGAGGTGGTGGTAAAGGAATAAGAAAGGTTCATAATGGTGATGAGGTTAGGGCATTGTTCAAGCAAGTTCAAGGTGAAGTTCCAGGCTCACCTATATTTATAATGAAGGTTGCATCCCAGAGCCGGCATTTAGAAGTCCAGTTACTTTGTGATCAGTACGGAAATGTTGCAGCTTTGCATAGCCGTGATTGCAGTGTTCAAAGGAGGCACCAAAAGATCATTGAGGAGGGACCAATTACTGTAGCTCCGATGGAGACAGTGAAAAAACTGGAACAGGCAGCTAGAAGATTGGCTAAATGTGTAAATTATGTTGGAGCAGCTACTGTTGAGTATCTATATAGTATGGACACTGGCGAGTACTACTTTCTAGAGCTGAACCCCCGACTACAGGTTGAGCACCCTGTTACTGAGTGGATAGCAGAGATAAATCTCCCAGCTGCACAAGTTGCTGTTGGGATGGGTATCCCTCTCTGGCATATTCCTGAGATAAGGCGTTTCTATGGGGTAGAACATGGTGCAGGATATGATGCTTGGAGGAAAACATTCACTTTAGCGACTCCTTTTGATTTTGATAGAGTAGAATCTATAAGGCCAAAAGGTCATTGTGTGGCCGTGCGAGTGACAAGTGAGGATCCTGATGACGGTTTTAAGCCTACAAGTGGGAAAGTACAGGAATTGAGCTTTAAAAGCAAGCCGAATGTGTGGGCATACTTCTCTGTTAAGTCTGGAGGAGGAATTCATGAATTTTCAGATTCTCAGTTTGGACACGTTTTCGCATTTGGAGAGTCTAGAGCTTTAGCTATTGCAAATATGGTTCTAGGGCTGAAGGAAGTTCAAATTCGGGGAGAAATTCGTACTAATGTTGATTATACCATTGATCTTTTAAATGCTTCCGACTACAGAGAAAACAAAATTCACACTGGATGGTTGGACAGTAGAATTGCAATGCGGGTTAGAGCAGAAAGGCCCCCTTGGTATCTTTCTGTTGTTGGAGGGGCACTCTTTAAAGCCTCTACTAGCAGTGCAGCAATAGTTTCAGATTATGTTGGCTATCTTGAAAAGGGACAAATCCCTCCCAAGCATATATCTCTTGTCCACTCTCAAGTGTCCTTGAACATTGAAGGAAGTAAGTACACGATTGACATGGTAAGAGGAGGACCAGGAAGTTATAGATTGAGAATGAATGAATCAGAAATGGAAGCAGAGATACATACATTACGTGATGGGGGTTTGCTGATGCAG‑‑‑‑‑‑‑‑‑TTGGATGGAAACAGTCATGTGATATATGCAGAGGAAGAAGCAGCAGGAACTCGCCTTCTAATAGATGGAAGGACTTGTTTGCTTCAGAATGATCATGATCCATCAAAGTTAGTTGCAGAGACACCATGCAAGCTTCTGAGATATTTGGTTATGGATGATAGTCATATTGATGCTGACGCTCCATATGCAGAAGTTGAGGTCATGAAGATGTGTATGCCTCTTCTTTCGCCTGCTTCTGGGTTTATTCATTTCAAAATGTCTGAAGGTCAAGCAATGCAGGCTGGTGAACTTATAGCAAGGCTTGATCTAGATGATCCATCAGCAGTAAGAAAAGCTGAACCTTTCCATGGGAGCTTCCCAGTCCTTGGTCCTCCTACCGCAATTTCTGGTAAAGTTCATCAGAAATGCGCTGCAAGTTTAAATGCAGCAAGAATGATCCTGGCTGGCTATGAGCACAATATAGATGAAGTTATGCAAAATTTGCTCAATTGCCTTGATAGTCCTGAACTGCCTTACCTTCAATGGCAAGAGTGCTTGGCTGTTCTGGCAAACCGTCTTCCCAAAGATCTTAAAAATGAGTTGGAATCAAAATATAAGGAATTTGAGAGGATTTCA‑‑‑AGCTCCCAAAATGTTGATTTTCCTGCCAAATTCTTGAAGGGAATCCTTGAAGCCCATCTTTCCTCCTGTCCTGAGAAAGAAAAAGGGGCCCAAGAAAGGCTTGTTGAACCTCTTATGAGTCTGGTGAAGTCTTACGAGGGTGGAAGAGAAAGCCATGCCCGTAGAATTGTTCAATCCCTTTTTGAAGAGTATCTTTCCGTTGAAGAACTTTTTAGTGATAACATACAGGCTGATGTAATTGAACGTCTCCGACTTCAGTATAAGAAAGATCTATTGAAGGTTGTAGACATAGTGGTCTCTCATCAGGGTGTTAGGAGTAAAAATAAGCTGATACTGCGACTGATGGAACAACTGGTGTATCCTAACCCTGCTGCCTACAGAGATCAATTGATCCGTTTCTCTGTACTCAACCATACAAATTATTCTGAGTTGGCACTGAAAGCAAGTTATCTGCTGGAACAAACTAAATTGAGTGAACTTCGTTCCAGCATTGCTAGAAGTCTTTCTGAGCTAGAGATGTTCACTGAGGATGGTGAAAATATTGATACTCCCAAGAGGAAGAGTGCCATTAATGACAGAATGGAGGACCTTGTGAGTGCTCCTTTGGCAGTTGAAGATGCCCTTGTAGGTTTGTTTGATCACAGTGATCACACCCTTCAAAGGCGAGTTGTGGAGACTTACATCCGCAGGCTGTACCAGCCATACCTTGTCAAAGAGAGTGTCAGGATGCAGTGGCACAGATCTGGTCTTATTGCTACATGGGAGTTCTTGGAAGAGTACATTGAGAGGAAGAAT‑‑‑GGGACTGAG‑‑‑‑‑‑‑‑‑‑‑‑TCTGATAAAACGCTGGTGGAGAAACATAGTGAGAGGAAATGGGGAGTGATGGTTATAATTAAATCTCTTCAATTTTTACCTGCAATTATTAGTGCTGCTTTAATTGTAGCAACTCATAACCTTCACGAAGCA‑‑‑CTTCCAAATGTTTCTATGGAACCAGCTAACCATGGTAATATGATGCATATTGCATTGGTGGGCATCAACAATCAGATGAGTTTACTGCAAGACAGTGGTGATGAGGATCAGGCTCAAGAAAGGATCAATAAGTTAGCCAAAATACTCAAAGACCAGGAAGTAGGCTCAACCATACATACTGCAGGTGTTGGAGTAATTAGCTGTATCATACAGAGGGATGAAGGGCGAGCCCCTATGAGGCACTCCTTTCACTGGTCAGCAGAAAAGCACTATTATGAGGAGGAGCCTCTCTTGCGTCATCTGGAACCTCCCCTATCAATTTACCTTGAATTGGACAAACTTAAAGGCTATGAGAATATATGCTATACTCCATCCCGAGATCGTCAGTGGCACTTGTATACAGTTGTAGATCCCAAGCCACCATCAATTCAGAGAATGTTTCTTCGAACACTTGTAAGACAGCCAACCACAAATGAAGGATTCTCATCTTATCAAAGACTGGATGTGGAAACATCTCGTACCCAATTGGCTATGTCTTTTACTTCAAGGAGCATTTTGAGGTCCTTGACGGCTGCAATGGAAGAGTTGGAACTTAATACACACAATGCCACTGTCAGATCTGAACATGCTCATATGTACCTCTATATCATACGAGAGCAACAAGTAGATGATCTTGTGCCTTACTCCAAGAGAGTCAACATAGATGCTGGTCAAGAAGTAACAGCAGTTGAAGCAATCTTGGAAGAACTGGCGCATGAAATCCATTCATCTGTTGGTGTGAGAATGCATAGATTAGGTGTTGTTAAGTGGGAAGTCAAGCTTTGGATGGCATCCTCTGGACTGGCAGATGGTGCTTGGAGATTTGTTGTTACAAATGTGACTGGCCATACATGCACTGTACATATATACCGAGAATTGGAGGATTCTAGCAGTCATAAAGTGATATACAGTCCAGTCACTGCAAAGGGTCCTCTGCAAGGTGTACCAGTGAATGCACACTATCAACCTTTGGGAGTTATTGACCGAAAACGTCTTTCAGCAAGGAAGAACAACACCACATACTGCTATGATTTTCCACTGGCATTTGAAACAGCCTTGGAACAGTCATGGGCTATCCAATACCCAGGATTCCAAAGGGCCAAGGATAAAGTTCTTCTAAAAGTAACAGAGATTAAATTTGCTGACAAAGAAGGTAGTTGGGGTACTCCTCTTGTTCCTGTGGAGCGTTTACCTGGACTCAATGATGTTGGCATGGTAGCCTGGTTTATGGAAATGCATACCCCTGAGTTCCCATCTGGAAGGACAATATTGGTTGTAGCAAACGATGTGACCTTCAAGGCTGGTTCTTTTGGCCCAAGAGAGGATGCATTCTATCGTGCAGTTACTGATCTTGCTATTCTGAAGAAAATACCTTTAATTTATCTGGCAGCAAATTCTGGTGCCCGTTTAGGTGTTGCTGAGGAAGTCAAAGCCTGTTTCAGAGTTGGGTGGTCTGATGAATCTAACCCTGAGAATGGTTTTCAGTATGTGTATTTAACACCTGAGGATTATGCTCAGATTGGGTCATCAGTGATAGCACACGAGTTAAAGCTGGAAAGTGGAGAAACCAGATGGGTCATTGATACTATTGTTGGGAAAGAGGATGGTTTGGGGGTTGAAAACTTGAGTGGCAGTGGGGCCATTGCTGGTGCCTATTCAAGGGCATACAAGGAAACTTTTACATTGACATATGTGACTGGCAGGACTGTGGGAATAGGTGCTTATCTTGCCAGGCTTGGGATGAGATGTATACAGAAGCTTGATCAGCCCATCATTCTTACTGGTTTTTCCGCATTAAATAAACTTCTTGGTCGGGAGGTTTACAGCTCTCACATGCAACTTGGTGGACCTAAAATCATGGCAACTAATGGAGTTGTTCATCTTACAGTTTCAGATGACCTTGAAGGTGTTTCTGCTATTTTGAAGTGGCTTAGCTATATCCCTTCTCATGTAGGTGGTGCACTTCCCATTCTACGGTCCCTTGATCCTCCAGAAAGGCCTGTGGAGTATTTCCCAGAAAACTCATGCGATCCTCGTGCTGCTATTTGTGGTGCTCTGGATGGTAGTGGAAGATGGCTGGGGGGTATTTTCGACAAGGACAGCTTTGTTGAGACACTAGACGGATGGGCAAGAACAGTTGTTACAGGAAGGGCAAAACTTGGAGGAATCCCTGTGGGAATTGTTGCTGTTGAAACACAGACAGTGATGCAAGTAATACCTGCGGATCCAGGCCAGCTTGATTCTCATGAGAGGGTTGTTCCTCAGGCCGGGCAAGTTTGGTTTCCCGATTCTGCAACTAAGACAGCCCAATCAATATTGGATTTCAACAAAGAGGAGCTCCCACTTTTCATTCTTGCAAATTGGAGGGGCTTTTCAGGTGGGCAGAGGGACCTTTTTGAAGGAATCCTTCAGGCTGGATCAACCATTGTAGAGAACCTTAGAACATACAAGCAGCCAATATTTGTATATATCCCAATGATGGGTGAACTCCGAGGTGGGGCGTGGGTTGTTGTAGACAGTAGAATCAATTCAGACCACATTGAAATGTATGCTGAGCGAACAGCTAAAGGCAATGTCCTTGAGCCAGAAGGGATGATTGAGATCAAATTTAGGACAAAAGAATTGTTGGAGACCATGGGTAGACTTGATCAACAATTGATAAATCTCAAGGAAAAACTTCAGGATGCCAAGAATAATAGGATCCATGAGACCATTGAATCCCTACAGCAGCAGATCAAGTCCCGTGAGAAACAGCTTCTGCCGCTGTACACCCAGATAGCTACCAGGTTTGCTGAATTGCATGATACTTCCCTCAGAATGGCAGCAAAGGGTGTAGTAAGAGAAGTTTTGGACTGGGGTAACTCCCGTTCCTTCTTCTACAAGAGACTGCGCAGGAGAATTGGTGAGCAGTCAATGATCAACAGTGTGAAAGAAGCTGCT‑‑‑GGTGACCATTTATCACATATAGCTGCAACGGACTTGATCAAAAACTGGTATTTGAATTCTGATATTGCCAAAGGTAGAGAAGATGCTTGGTTGGATGATGAGGCTTTCTTCAGATGGAAGGATGATCCTAAGTACTACGTGGATAAACTAAAGGAATTGCGTATCCAGAAGGTATTACTTCAATTGACAAATATTGGTGAGTCAGTTTCAGATTTGCAAGCTTTACCTCAAGGTCTTGCTGCCCTTTTAAGCAAGTTGGAGCCATCAAGCCGTGTGAAATTAACTGATGAACTTCGCAAGTTAAGAGAGCAT

>Cof_ACC_RKLL‑2000979

ATGTCTGGCATTGGACGTGGGAAT‑‑‑‑‑‑‑‑‑GGATACAATAATGTTGTAATGCCAAATAGGCATCCTGCTACAATTTCTGAAGTTGATGAATTTTGCCATGCCCTCGGGGGAAAGAAGCCAATCCATAGCATACTAATCGCAAACAATGGAATGGCTGCTGTCAAGTTTATTCGTAGCGTTAGGAGCTGGGCCTACGAGACATTTGGCACAGAGAAGGCTATCTTGTTGATGGCCATGGCTACTCCAGAGGACATGAGAATCAACGCAGAACATATCAGAATAGCTGATCAATTTGAAAAAGTACCTGGTGGGACCAATAACAATAACTATGCTAATGTGCAACTTATCGTAGAG‑‑‑‑‑‑‑‑‑TTGGCAGAGAGAACTCATGTAGATGCAGTCTGGCCGGGCTGGGGTCATGCATCAGAGAATCCTGAGCTGCCAGATGCATTAAATGCAAAAGGAATTGTATTTCTTGGACCTCCTGCCATTTCTATGGCAGCTTTGGGAGATAAAATTGGTTCATCCTTAATTGCACAAGCAGCTGAAGTGCCAACGCTTCCATGGAGTGGTTCTCATGTTAAAATTCCTGCTGATAGTCGCTTGGTCACCATTCCTGATGAAATCTACTGTGAAGCATGTATCTATACAACAGAGGAAGCAATTACAAGTTGTCAAGTTGTAGGTTACCCTGCAATGATCAAGGCATCTTGGGGCGGTGGTGGCAAAGGGATAAGAAAGGTTCATAATGATGATGAAGTCAGGGCATTATTCAAGCAAGTTCAAGGTGAAGTTCCCGGTTCACCTATATTTATAATGAAGGTTGCATCCCAGAGCCGACATTTAGAAGTGCAGTTACTGTGTGATCAATATGGGAATGTTGCAGCTTTGCATAGCCGTGATTGCAGTGTTCAGAGGAGGCACCAAAAGATTATTGAGGAGGGACCAATTACTGTAGCTCCTTTGGAGACTGTTAAAAAGCTGGAACAGGCAGCTAGAAGATTGGCCAAAAGCGTAAATTATGTTGGAGCAGCTACTGTTGAGTATCTATACAGCATGGAGACTGGCGAGTACTACTTTTTAGAGCTGAACCCCCGGCTACAGGTTGAACACCCTGTTACTGAGTGGATAGCAGAGGTTAATCTGCCAGCAGCACAAGTTGTTGTTGGGATGGGTATCCCTCTCTGGCAAATTCCTGAGATAAGGCGTTTCTATGGGATCGAACATGGTGCGGGATATGATGCTTGGAGGAAAACATCAGTTTTAGCTACCCCTTTTGACTTTGACAAAGTAGAATCTACAAAGCCAAAAGGTCACTGTGTGGCAGTGCGAGTGACAAGTGAGGACCCTGATGATGGTTTTAAGCCTACAAGTGGCAAAGTACAGGAGCTGAGCTTTAAAAGCAAACCAAATGTGTGGGCATACTTCTCTGTTAAGTCTGGAGGAGGAATTCATGAATTCTCAGATTCTCAGTTCGGGCATGTTTTTGCATTTGGAGAGTCCAGAGCTTTAGCTATTGCAAATATGGTTCTAGGCCTGAAGGAGATTCAAATTCGGGGAGAAATTCACACCAACGTTGATTATACTGTCGATCTCTTAAATGCATCAGACTACAGAGAAAACAAAATCCACACAGGATGGTTGGACAGTCGAATTGCAATGCGGGTCAGAGCAGAGAGGCCCCCTTGGTATATTTCTGTTGTTGGAGGGGCACTCTACAAAGCTTCTACTAGCAGTGCAGCTTTAGTTTCAGATTACGTTGGCTATCTTGAAAAAGGGCAAATTCCTCCCAAGCACATATCTCTTGTCCACTCTCAAGTGTCCTTGAACATTGAAGGAAGCAAATACACTATTGACATGGTAAGAGGAGGACCGGGAAGTTACAGATTGCAATTGAATGGATCAAAAATCGAAGCAGAGATACATACATTACGTGATGGTGGTTTGCTGATGCAG‑‑‑‑‑‑‑‑‑TTGGATGGAAACAGTCATGTAATATATGCAGAGGAAGAAGCAGCTGGCACTCGCCTTCTAATTGATGGAAGGACTTGCTTGCTTCAGAATGATCATGATCCATCAAAATTAGTTGCAGAGACCCCATGCAAACTCCTGAGATATTTGGTTATGGATGATAGTCATATTAATTCTGATACACCATATGCTGAAGTTGAGGTCATGAAGATGTGTATGCCTCTTCTTTCACCTGCTTCTGGGGTTATTCATTTCAAAATGTCTGAAGGGCAAGCAATGCAGGCTGGTGAGCTTATTGCAAGGCTTGATCTAGATGACCCTTCAGCTGTGAGAAAAGCAGAACCTTTCACAGGGAGCTTGCCAGTCCTGGGTCCTCCTACTGCAGTTTCTGGTAAAGTACATCAGAAATGTGCTGCAAGTTTAAATTCAGCATGGATGATTCTTGCTGGCTATGAGCACAATATCGATGAAGTTGTTCAAAATTTATTGAATTGCCTTGATAGTCCAGAATTGCCCTTCCTTCAGTGGCAAGAGTGCTTGGCTGTTCTGGCAACACGTCTTCCTAAAGATCTGAAAAATGAGTTGGAATCAAAATATAAGGAGTTTGAGAGCATTTCT‑‑‑AGCTCCCAAAATGTTGATTTTCCTGCTAAATTATTGAGGCAAATCATTGAAGTTCATCTCTCATCCTGCCCTGAAAAGGAAAAAGGAGCTCAAGAAAGGCTTGTGGAACCTCTCGTGAGTCTAGTGAAGTCTTATGAAGGTGGAAGAGAGAGCCATGCCCGTATAATGGTTCAGTCCCTTTTTGAAGGGTATCTTTCTGTTGAAGAATTATTTAGTGATAACATACAGGCTGATGTAATTGAACACCTCCGTCTTCAATATAAGAAAGATCTGTTGAAGGTTGTAGACATAGTGCTCTCTCATCAGGGTGTCACGAGTAAAAATAAGCTGATATTGCGACTTATGGAACAACTGGTTTATCCTAATCCTGCAGCCTACAGGGATCAACTGATTCGTTTCTCTGCACTGAACCATACAAGTTATTCTGAGTTGGCACTTAAGGCAAGTCAGTTACTGGAACAGACTAAATTAAGTGAACTTCGTTCCAGCATTGCTAGAAGCCTTTCTGAATTGGAGATGTTCACTGAAGATGGTGAAAATATTGATACTCCAAAGAGGAAGAGTGCCATTAATGACCGAATGGAGGACCTTGTGAGTGCTCCCTTGGCAGTTGAAGATGCCCTTGTTGGTTTGTTTGATCACAGTGATCATACCCTTCAAAGGCGGGTTGTGGAAACTTATGTCCACAGGCTTTACCAGCCATATCTTGTCAAGGAAAGTGTCAGGATGCAGTGGCACAGATCTGGTCTTATTGCTTCATGGGAGTTTTTGGAGGAGTACGTGGAAAGGAAGAGT‑‑‑GAAACTGAAGTC‑‑‑CAAATGGCTGATAAA‑‑‑‑‑‑‑‑‑AACAAACCAAATAATAGGAAATGGGGAGTGATGGTTATAATTAAATCTCTCCAATTTTTACCTGCAACTATCACTTCTGCACTGAGAGAAGCAACTCATAACCTTCATGAAGCA‑‑‑CTTCCAAATGATTATGCAGATCCAGCTAATCATGGTAATATGATGCATGTTGCATTGGTGGGCATCAACAACCAGATGAGCTTACTGCAAGACAGTGGTGATGAGGATCAGGCTCAAGAAAGGATCAATAAGTTAGCCAAAATACTTAAAGAGAAGGAAGTAAGCACAAGTATACGTTCTGCAGGAGTTGAAGTAATTAGCTGTATTATACAGAGAGATGAAGGGCGAACTCCCATGAGGCACTCTTTTCACTGGTCTGCAGAAAAGCTCTGTTATGAGGAGGAGCCTCTCTTGCGTCATTTGGAACCTCCCCTGTCCATCTATCTTGAATTGGATAAACTTAAAGGCTATGAGAATATTCATTATACCCCATCCCGGGATCGTCAGTGGCACTTGTACACAGTTGTTGATCCCAAGCCATTATCAATTCAGAGAATGTTCCTTAGAACACTTGTTAGGCAGCCAAGTGTAAATGAAGGATTCTCATTATATCAAGGGCTGAATGTGGAATTATCTCGTACCCAATTGGCTATGTCTTTTACTTCAAGGAGCATTTTGAGGTCTTTGATGGCTGCGATGGAGGAACTGGAACTCAATGCACACAATGCAACCGTTAAGTCTGAGCATACTCATATGTATTTATATATCATACAGGAGCAGCAAATAGATAATCTTTTGCCTTATCCGAAGAGAATTAATGTGGATGCTGGCAAAGAAGCAGCAGCTGTTGAGGCAATCCTGCAAGAAGTGGCACATGAAATCCATTCATCTGTTGGTGTAAGAATGCATAGATTTGGTGTTGTTGAATGGGAATTTAAGCTTTGGATGGCATCCTCTGGACAGGCAAATGGTGCTTGGAGGGTTGTTGTAAGCAACGTGACAGGCCATACATGCACTGTACATATATATCGAGAAAGGGAGGATACCGGCTCTCACAAAGTGGTATACAGTTCAGTCACTGTAAAGGGTCCTCTTCATGGTGTACAAGTGAATGCACAATATCAACCTTTGGGAGTTATTGACCGAAAACGTCTTTCAGCAAGGAAAAACAACACTACATACTGCTATGATTTCCCATTGGCCTTTGAAACAGCCTTGGAACAGTCGTGGGCAATTCAATGCCCAGGATTCCAAAAAGCCAAGGAAAAAGCCCTTCTTAAAGTAACAGAGCTTAAATTGGCTGACAAAGAAGGTAGCTGGGGTACTCCTCTTGTACCTGTGGAATGTTCGCCCGGACTCAATGATGTTGGCATGGTAGCCTGGACTATGGAAATGTGTACCCCTGAGTTCTCATCCGGAAGGACAATATTGGTTGTTGCAAATGATGTGACCTTCAAAGCTGGCTCTTTTGGGCCAAAAGAGGATGCATTCTTCCACGCAGTAACTGATCTTGCTTGTGTGAAGAAACTACCTTTAATTTATCTAGCAGCAAATTCTGGTGCTCGTTTAGGTGTTGCAGAGGAAGTCAAATCCTGTTTCAGAGTTGGTTGGTCTGATAAATCCGATCCTGAGCATGGTTTTCAGTATGTATATTTGACAGCTGATGATTATGCTCGGATTGGATCTTCAGTGATAGCACACAAGTTGAAGCTGGAAAGTGGAGAAACCAGATGGGTTATAGATACTATTGTCGGGAAGGAGGATGGTCTGGGGGTTGAAAACTTGAGTGGCAGTGGAGCTATTGCTGGTGTCTATTCAAGGGCATACAGGGAAACCTTTACACTGACATATGTGACTGGCAGGACTGTTGGAATAGGAGCTTATCTTGCTAGGCTTGGGATGAGATGCATCCAGAGACTTGATCAGCCCATAATTCTGACCGGTTTTTCTGCATTAAACAAACTTCTTGGTCGAGAGGTATACAGCTCTCACATGCAACTTGGTGGACCTAAAATTATGGCAACTAATGGAGTCGTTCATCTTACAGTCTCTGATGACCTTGAGGGTGTTTCTGCTATTTTGAAGTGGCTTAGCCATATTCCTTCTCATGTAGGCGGTTCACTTCCCATTGTGCATCCACTTGATCCACCAGAGAGGCTTGTGGAGTATTTCCCAGAAAACTCATGTGATCCCCGTGCTGCTATTTGCGGTAGTCAGGATGGTAATGGAAGATGGCTGGGAGGAATTTTTGACAAGGACAGCTTTGTGGAGACTTTAGAGGGATGGGCCAGAACTGTGGTTACCGGAAGGGCAAGGCTGGGAGGAATTCCTGTGGGAATTGTGGCTGTTGAGACACAGACAGTAATGCAAGTTATACCTGCTGATCCAGGCCAGCTTGATTCTCATGAAAGGGTTGTTCCTCAGGCTGGACAAGTCTGGTTTCCTGACTCTGCAACTAAGACGGCCCAAGCAATATTGGACTTCAACAGAGAAGAGCTCCCACTTTTCATTCTTGCAAATTGGAGAGGCTTTTCAGGTGGGCAGAGGGACCTCTTCGAAGGAATTCTTCAGGCTGGATCAACTATTGTGGAGAACCTTAGAACATTCAAGCAGCCTATATTTGTATATATCCCAGTGATGGGAGAACTCCGTGGTGGGGCATGGGTTGTTGTGGATAGTCGAATCAATTCAGATCACATTGAAATGTACGCAGAACGAACGGCTAAAGGTAATGTCCTTGAGCCAGAAGGGATGATTGAGATCAAATTTAGGACAAGGGAATTACTGGAGTGTATGGGTCGGCTTGATCAACAGTTGATAAATCTCAAGGTAAAACTGCAAGATGCCAAGAGTAACAGTGATTATCAGGCCATTGAATCCCTACAGCAGCAGATGAGATCTCGTGAGAAACAGCTCCTGCCCGTGTACACCCAGATAGCTACCAAGTTTGCTGAACTGCATGATACTTCCCTAAGGATGGCTGCAAAGGGTGTAATAAGAGAAGTTCTGGACTGGGGTAACTCCCGATCCTTCTTCTACCAAAGACTGCACCGAAGAATAGGTGAGCACTCACTGATAAACATTGTGAGAGGTGCTGCG‑‑‑GGGGACCATTTGTCACATACATCTGCAATGGAATTAGTCAGAAGCTGGTATTTGAATTCTGATATTGCCAAGGATAGGGCAGATCCTTGGTTGGATAATGAAGCATTCTTCAGATGGAAAGATGATCCAAAGAATTACGAGAATAAATTAAAGGAATTAAGTGCCCAGAAGGTGTTGCTTCAGTTGACAAACATTGGAGATTCAGTTTTAGATTTGCAAGCTTTACCTCAAGGTCTTGCTGCCCTTTTAAGCAAGTTGGAGCCACCACGCCGTGAAAAATTGACAGACGAACTCCGAAAGGTGATTGGT‑‑‑

>Gma_ACC1_Glyma.04G104900

ATGGCTGACATTGGGCATAGGAAT‑‑‑‑‑‑‑‑‑GGATATGTAAACAGTGTACTGCCAAATAGGCCCCCTGCTGCAATATCTGAAGTAGATGATTTCTGCAATGCCCTTTGCGGAAACAGGCCAATTCACAGTATATTAATTGCAAATAATGGAATGGCAGCAGTCAAGTTTATACGTAGTGTTAGGAGCTGGGCCTATGAGACATTTGGCTCAGAGAAAGCCATCTTGTTGGTTGCCATGGCTACTCCAGAGGACATGAGAATCAATGCAGAACATATCAGAATAGCTGATCAATTTGTGGAAGTACCTGGTGGGACCAATAACAATAACTATGCCAACGTGCAGCTTATTCTTGAG‑‑‑‑‑‑‑‑‑ATGGCTGAGATAACTCATGTTGATGCGGTGTGGCCTGGTTGGGGTCATGCATCAGAAAATCCTGAGCTCCCAGATGCATTAAAAGCAAAAGGAATTGTATTTCTTGGACCTCCTGCTATATCTATGGCTGCATTGGGAGATAAAATTGGTTCATCATTGATTGCTCAAGCAGCAGAAGTGCCAACCCTTCCATGGAGTGGTTCTCATGTGAAAATTCCGCCTGAAAGTTCCTTGATTACTATTCCTGACGAAATTTATCGGGAAGCCTGTGTTTATACCACAGAGGAAGCGGTGGCAAGTTGTCAAGTTGTAGGATACCCTGCAATGATCAAGGCATCTTGGGGTGGTGGTGGTAAAGGAATAAGAAAGGTTCATAATGATGATGAGGTAAGGGCATTGTTCAAGCAAGTTCAAGGTGAAGTTCCAGGTTCTCCCATATTTATAATGAAGGTTGCATCCCAGAGCCGACATCTAGAAGTCCAGTTACTTTGTGATCAGTACGGTAATGTCGCAGCTTTGCATAGCCGTGATTGCAGTATTCAAAGGAGGCACCAAAAGATTATTGAGGAGGGTCCCATTACTGTAGCACCCATAGAAACAGTGAAACAATTGGAACAGGCAGCTAGAAGGTTGGCTAAATCTGTAAATTATGTTGGGGCGGCTACTGTTGAGTATCTTTTTAGTATGGAAACTGGCGAGTACTACTTTTTAGAGTTGAACCCTCGGCTACAGGTTGAACATCCTGTTACTGAGTGGATAGCAGAGATAAATCTGCCAGCAGCACAAGTTGCCATTGGGATGGGTATCCCTCTCTGGCAAATTCCTGAGATAAGGCGTTTCTATGGGGTGGAACATGGTGGAGGGTATGATGCCTGGAGAAAAACATCTGTTTTAGCTACCCCTTTCGATTTTGACAAAGCACAATCTACGAGGCCAAAAGGTCATTGTGTGGCAGTGCGAGTAACTAGTGAGGACCCTGATGATGGTTTTAAGCCTACAAGTGGCAAAGTGCAGGAGCTTAGCTTTAAAAGCAAGCCAAATGTGTGGGCATACTTCTCTGTCAAGTCTGGAGGAGGAATTCATGAATTCTCAGATTCACAGTTTGGGCATGTTTTTGCTTTTGGAGAATCTAGGGCCTTAGCAATTGCAAATATGGTTCTGGGGCTGAAGGAGATTCAGATTCGGGGAGAAATCCGTACCAATGTTGATTACACCATTGATCTTCTGAATGCTTCAGACTACAGAGAAAACAAAATTCACACAGGTTGGCTGGACAGTAGAATTGCAATGCGGGTTAGAGCAGAGAGGCCTCCCTGGTATCTTTCTGTCGTTGGAGGGGCACTCTATAAAGCTTCCACTAGCAGTGCAGCTTTGGTTTCAGATTATGTTGGCTATCTTGAAAAGGGACAAATCCCTCCCAAGCACATATCTCTTGTCCATTCTCAAGTGTCCTTGAACATTGAAGGAAGCAAATATACGATTGACATGATACGAGGTGGGTCTGGAAGTTATAGATTGAGAATGAATCAATCAGAGATAGAAGCAGAAATACATACTTTACGTGATGGAGGTTTGCTAATGCAG‑‑‑‑‑‑‑‑‑TTGGATGGAAACAGTCATGTAATATATGCAGAGGAAGAAGCAGCTGGAACTCGCCTTCTAATAGATGGAAGGACTTGCTTGCTTCAGAATGATCATGATCCGTCGAAGTTGGTTGCCGAGACACCATGCAAGCTTCTGAGATATTTGGTTGCAGATGACAGTCATGTTGATGCTGACACACCGTATGCTGAAGTTGAGGTCATGAAAATGTGCATGCCTCTTCTTTCGCCTGCTTCTGGGATTATTCATTTCAAAATGTCTGAAGGTCAAGCAATGCAGGCTGGTGAACTTATAGCAAGGCTTGATCTGGATGATCCTTCAGCTGTAAGAAAGGCAGAACCCTTCACTGGGAGCTTCCCAGTCCTGGGACCTCCTACCGCAATTTCTGGTAAAGTTCATCAGAAATGCGCTGCAAGCTTAAATGCTGCACGGATGATTCTTGCTGGGTATGAGCACAATATTGATGAAGTTGTGCAAAGTTTGCTTAATTGCCTTGATAGTCCTGAATTACCTTTCCTTCAATGGCAAGAGTGCTTGGCAGTTCTGGCAACCCGTCTTCCCAAAGATCTAAAAAATGAGTTAGAATCAAAATATAAGGAGTTTGAGGGGATTTCC‑‑‑AGCTCCCAAATTGTTGATTTCCCTGCCAAATTATTGAAGGGAATTCTTGAAGCTCATCTTTCCTCGTGTCCCGACAAAGAAAAAGGAGCTCAAGAAAGACTAGTTGAACCTCTATTGAGTCTTGTTAAGTCTTATGAGGGTGGAAGAGAGAGCCATGCCCATATAATTGTTCAATCCCTTTTTGAAGAATATCTTTCTGTTGAAGAATTATTTAGTGATAATATTCAGGCTGATGTGATTGAACGTCTGCGTCTACAATACCAGAAAGATCTATTGAAGATTGTTGATATAGTGCTTTCTCATCAGGGAATCAAGAGTAAAAATAAGCTGATATTGCTACTAATGGATAAATTGGTTTACCCTAACCCTGCTGCCTATAGGGATCAGTTAATCCGTTTCTCTTTACTCAACCATACAAATTATTCTGAGTTGGCTCTAAAGGCAAGTCAACTGCTGGAACAAACTAAATTAAGTGAACTTCGATCCAACATTGCTAGAAGTCTTTCTGAGTTAGAGATGTTTACAGAGGATGGTGAAAATATTGATACTCCCAAGAGGAAGAGTGCCATTAATGACCGAATGGAGGACCTTGTGAGTGCTCCTTTGGCGGTTGAAGATGCCCTCGTGGGTTTATTTGATCACAGTGATCACACCCTTCAGAGGAGGGTTGTGGAAACTTATATACGCAGGCTCTACCAGCCATATCTTGTCAAAGGGAGTGTCAGGATGCAATGGCACAGATCTGGTCTTATTGCTACTTGGGAGTTCTATGATGAGTACATTGAACGGAAGAAT‑‑‑GGGGTTGAAGAC‑‑‑CAAACTTTGAATAAAATGGTGGAGGAGAAACATGGTGAGAAAAAGTGGGGAGTGATGGTTATAATCAAATCTCTTCAATTTTTGCCTGCAATTATCAGTGCTGCATTGAGGGAGGCAACCAACAACCTTCACGAAGCA‑‑‑CTTACAAGTGGTTCTGTGGAACCAGTTAACTATGGCAATATGATGCATATTGGATTAGTGGGCATCAACAACCAGATGAGTTTACTGCAAGATAGTGGTGATGAGGATCAGGCTCAAGAAAGAATCAATAAGTTAGCTAAAATACTCAAAGAGCATGAAGTAGGCTCCACCATACGAGCTGCAGGTGTCAGAGTAATCAGCTGTATTATTCAAAGGGATGAAGGACGTGCCCCAATGAGGCACTCCTTTCACTGGTCAGAAGAAAAGCTTTACTATGCAGAGGAACCTCTGTTGCGTCATCTGGAACCTCCGCTATCCATTTATCTTGAATTGGACAAACTTAAAGCCTATGAGAATATACGGTATACTCCATCTCGAGATCGGCAATGGCATCTATATACGGTTGTGGATCACAAGCCACAACCAATTCAAAGAATGTTTCTTCGAACACTTCTAAGACAGCCGACAACAAATGAAGGATTCTCTTCATATCAAAGGCTGGATGCAGAAACATCTCGCACCCAATTGGCCATGTCCTTTACTACAAGGAGCATTTTTAGGTCCTTGATGGCTGCAATGGAGGAGTTGGAACTTAATGCACACAATGCCAATATCAAATCTGAACATGCTCATATGTACCTCTATATCATACGCGAGCAACAAATAGATGATCTTGTGCCCTATCCCAAGAGAATTAACATAGATGCTGGGAAAGAAGAAACAACAGTTGAGGCAATCTTGGAAGAACTGGCACGGGAAATCCATTCATCTGTTGGTGTTAGAATGCATAGATTAGGGGTTGTTGTGTGGGAAGTCAAGCTCTGGATGGCAGCTTGTGGACAGGCAAATGGTGCTTGGAGGGTCATTGTAAACAATGTGACTGGTCATACATGCACCGTACATATATACCGAGAAAAGGAGGATACTGTCACTCATAAAGTGGTATACAGATCAGTCTCGATTAAGGGTCCACTGCATGGTGTGCCAGTGAATGAGAATTATCAACCTTTGGGAGTTATTGACCGAAAGCGTCTTTCTGCAAGAAAGAACAGTACCACATACTGCTACGATTTTCCTCTTGCATTTGAAACGGCCTTGGAACAGTCATGGGCAATCCAACAGCCAGGATTTCAAAGGGCCAAAGATAAAAATCTTTTAAAGGTAACAGAGCTCAAATTTGCGGATAAAGAAGGTAGCTGGGGTGCTCCTCTTGTTCCTGTGGAGCGTTACCCTGGACTCAACGATGTTGGCATGGTAGCCTGGTTTATGGAAATGTGTACCCCTGAGTTCCCATCTGGAAGGACAATATTGGTTGTTGCAAATGATGTGACCTTCAAGGCTGGTTCTTTTGGTCCAAGAGAGGATGCATTCTTCCGTGCAGTAACTGATCTTGCATGTACAAAAAAACTACCTTTAATTTATTTAGCTGCAAATTCTGGTGCCCGTTTAGGTGTAGCTGAAGAAGTCAAATCCTGTTTCAGAGTTGGTTGGTCTGAGGAATCCAATCCTGAGCATGGCTTTCAGTATGTATATTTAACACCTGAGGATTATGCTCGGATTGGATCTTCAGTGATAGCACATGAGTTAAAGCTGGAGAGTGGAGAAACCAGATGGGTTATTGATACCATTGTTGGCAAGGAGGATGGCCTTGGGGTTGAAAACTTGAGTGGAAGTGGGGCTATTGCTGGTGCTTATTCAAGGGCATACAAGGAAACCTTTACACTGACATATGTAACTGGCAGAACTGTTGGAATTGGTGCTTATCTTGCTAGGCTTGGGATGAGATGCATACAAAGGCTTGATCAGCCCATAATTCTTACTGGTTTTTCAGCATTAAACAAACTTCTTGGTCGGGAGGTATACAGCTCTCACATGCAACTTGGTGGACCTAAAATCATGGCAACTAACGGAGTCGTTCATCTTACAGTTTCCGATGATCTTGAAGGTATTTCTTCTATTTTGAAGTGGCTTAGCTACATTCCTTCTCATGTAGGTGGTGCACTTCCCATTGTAAAGCCGCTTGATCCTCCAGAAAGACCAGTGGAGTATTTCCCAGAAAACTCGTGTGATCCTCGTGCTGCCATTTCTGGAACCTTGGATGGTAATGGAAGATGGCTGGGAGGAATTTTTGACAAAGACAGCTTTGTGGAGACACTAGAGGGATGGGCAAGAACAGTTGTTACAGGAAGGGCAAAGCTTGGAGGAATCCCTGTGGGAATAGTTGCTGTAGAAACACAGACAGTAATGCAAATAATACCTGCTGATCCTGGTCAGCTTGATTCTCACGAGAGGGTTGTTCCTCAAGCCGGGCAAGTGTGGTTCCCTGATTCCGCAACCAAGACAGCCCAAGCAATATTGGATTTCAACAGAGAAGAGCTCCCGCTTTTCATTCTTGCAAACTGGAGAGGCTTTTCAGGAGGGCAAAGGGACCTTTTTGAAGGAATTCTTCAGGCTGGTTCCACTATTGTGGAGAACCTTAGAACATACAAGCAGCCCATATTTGTATACATCCCAATGATGGGCGAACTCCGTGGCGGGGCATGGGTTGTTGTTGACAGTCGAATCAATTCAGACCACATCGAAATGTACGCGGATAGAACAGCTAAAGGTAATGTCCTTGAGCCAGAAGGAATGATCGAGATCAAATTTAGAACAAGAGAATTGTTGGAGTCTATGGGTAGACTTGATCAGCAATTGATAACTTTGAAGGTAAAACTTCAGGAAGCCAAGAGTAACAGGGACATCGCGGCCTTTGAATCCCTACAGCAGCAGATTAAATCACGCGAGAGACAGCTTTTGCCTGTGTATACCCAGATAGCTACCAAGTTTGCTGAACTGCACGATACTTCCTTAAGAATGGCTGCCAAGGGTGTCGTTAGAGAAGTTCTGGACTGGTGTAACTCCCGTGCTGTCTTCTATCAGAGACTGCACAGGAGAATTGGTGAGCAGTCACTGATCAACAGTGTGAGAGATGCTGCT‑‑‑GGTGACCAATTGTCACATGCATCTGCACTGAACTTGCTCAAAGAATGGTATTTACATTCTGATATTGCCAAAGGTAGAGCAGATGCTTGGTTGGATGACAAAGCCTTCTTCAGATGGAAGGATAATCCTGCAAATTACGAGAATAAACTAAAGGAATTGCGTGCGCAGAAAGTGTTGCTTCAATTGACAAATATTGGTGACTCAGCTCTAGATTTGCAAGCTCTACCTCAAGGTCTTGCTGCCCTTTTAAGCAAGTTGGAGCCATCGGGTCGCGTGAAGTTGACCGATGAACTTCGCAAGGTACTTGGT‑‑‑

>Gma_ACC2_Glyma.06G105900

ATGGCTGACATTGGGCGTAGGAAT‑‑‑‑‑‑‑‑‑GGATATGCAAACAGTGTACTACCAAATAGGCCACCTGCTGCAATATCTGAAGTAGATGAATTCTGCAATGCCCTTGGTGGAAACAGGCCAATTCATAGTATATTAATTGCAAATAATGGAATGGCAGCAGTCAAGTTTATACGTAGTGTTAGGAGCTGGGCCTATGAGACATTTGGCTCAGAGAAAGCCATCTTGTTGGTTGCCATGGCTACTCCAGAGGACATGAGAATCAATGCAGAACATATCAGAATAGCTGATCAATTTGTGGAAGTACCTGGTGGGACCAATAACAATAACTATGCCAACGTGCAGCTTATTCTTGAG‑‑‑‑‑‑‑‑‑ATGGCTGAGATAACTCATGTTGATGCGGTGTGGCCTGGTTGGGGTCATGCATCAGAAAATCCTGAGCTTCCAGATGCATTAAAAGCAAAAGGAATTGTATTTCTTGGACCTCCTGCTATATCTATGGCTGCATTGGGGGATAAAATTGGTTCATCATTGATTGCTCAAGCAGCGGAAGTGCCTACCCTTCCATGGAGTGGTTCTCATGTGAAAATTCCGCCTGAAAGTTCCTTGATTACTATTCCTGATGAAATTTATCGGGAAGCATGTGTTTATACAACAGAGGAAGCAGTGGCAAGTTGTCAAGTTGTAGGATACCCTGCAATGATCAAGGCATCTTGGGGTGGTGGTGGTAAAGGAATAAGAAAGGTTCATAATGATGATGAGGTAAGGGCATTGTTCAAGCAAGTTCAAGGTGAAGTTCCGGGTTCACCCATATTTATAATGAAGGTTGCATCCCAGAGCCGACATCTAGAAGTCCAGTTACTTTGTGATCAGTACGGAAATGTCGCAGCTTTGCATAGCCGTGATTGCAGTGTTCAAAGGAGGCACCAAAAGATTATTGAGGAGGGGCCCATTACTGTAGCACCCATAGAAACAGTGAAAAAACTGGAACAGGCCGCTAGAAGGTTGGCTATATCTGTAAATTATGTTGGGGCGGCTACTGTTGAGTATCTTTATAGTATGGAAACTGGCGAGTACTACTTTTTAGAGTTGAACCCTCGGCTACAGGTTGAACATCCTGTTACTGAGTGGATAGCAGAGATAAATCTGCCAGCAGCACAAGTTGCCATTGGGATGGGTGTTCCTCTCTGGCAAATTCCTGAGATAAGGCGTTTCTATGGGGTGGAACATGGTGGAGGGTATGATGCTTGGAGAAAAACATCCGTTTTAGCTACCCCTTTTGATTTTGACAAAGCACAATCTACAAGGCCAAAAGGTCATTGTGTGGCTGTGCGAGTAACTAGTGAGGACCCTGATGATGGTTTTAAGCCTACAAGTGGCAAAGTGCAGGAGCTTAACTTTAAAAGCAAGCCAAATGTGTGGGCGTACTTCTCTGTCAAGTCTGGAGGAGGAATTCATGAATTCTCAGATTCACAGTTTGGGCATGTTTTTGCTTTTGGAGAATCTAGGGCTTTAGCAATTGCAAATATGGTTCTGGGGCTGAAGGAGATTCAGATTCGGGGAGAAATCCGTACCAATGTTGATTACACCATTGATCTTCTGAATGCTTCAGACTACAGAGAAAACAAAATTCACACAGGTTGGCTGGACAGTAGAATTGCAATGCGGGTTAGAGCAGAAAGGCCTGCCTGGTATCTTTCGGTCGTTGGAGGGGCACTCTATAAAGCTTCTGCTAGCAGTGCAGCTTTGGTTTCAGATTATGTTGGTTATCTTGAAAAGGGACAAATCCCTCCCAAGCACATATCTCTTGTCCATTCTCAAGTGTCCTTGAACATTGAAGGAAGCAAATATACGATTGACATGATACGAGGTGGGTCTGGAAGTTATAGATTGAGAATGAATCAATCAGAGATAGAAGCAGAAATACATACTTTACGTGATGGAGGTTTGCTAATGCAG‑‑‑‑‑‑‑‑‑TTGGATGGAAACAGTCATGTAATATATGCAGAGGAAGAAGCAGCTGGAACTCGCCTTCTAATAGATGGAAGGACTTGCTTGCTTCAGAATGATCATGATCCATCAAAGTTGGTTGCGGAGACACCATGCAAGCTTCTGAGATATTTGGTTGCAGATGACAGTCATGTTGATGCTGACACACCATATGCTGAAGTTGAGGTCATGAAAATGTGCATGCCTCTTCTTTCGCCTGCTTCTGGGATTATTCATTTCAAAATGTCTGAAGGTCAAGCAATGCAGGCTGGTGAACTTATAGCAAGGCTTGATCTGGATGATCCTTCAGCTGTAAGAAAGGCAGAACCCTTCACTGGGAGCTTCCCAGTTCTGGGACCTCCTACTGCAATTTCTGGTAAAGTTCATCAGAAATGCGCTGCAAGCTTAAATGCCGCACGGATGATTCTCTCTGGGTATGAGCACAATATTGATGAAGTTGTGCAAAGTTTGCTTAATTGCCTTGATAGTCCTGAATTGCCTTTCCTTCAATGGCAAGAGTGCTTGGCAGTTCTTGCAACCCGTCTTCCCAAAGAGCTAAAAAATGAGTTGGAATCAAAATATAAGGAGTTTGAGGGGATTTCA‑‑‑AGCTCCCAAATTGTTGATTTCCCTGCCAAATTATTGAAGGGAATTATTGAAGCTCATCTTTCCTCCTGTCCCGACAAAGAAAAAGGAGCTCAAGAAAGACTAGTTGAACCTCTATTGAGTCTTGTTAAGTCTTATGAGGGTGGAAGAGAGAGCCATGCCCATATAATTGTTCAATCCCTATTTGATGAGTATCTTTCTGTTGAAGAATTATTTAGCGATAATATTCAGGCTGATGTGATTGAACGTCTGCGTCTACAATACAAGAAAGATCTATTGAAGATTGTTGATATAGTGCTCTCTCATCAGGGAATCAAGAGTAAAAATAAGCTGATCTTGCAACTGATGGATAAATTGGTTTACCCTAACCCTGTTGCCTATAGGGATCAGTTAATCCGTTTCTCTTTACTCAACCATACAAATTATTCTGAGTTGGCTCTAAAGGCAAGTCAACTGCTGGAACAAACTAAATTAAGTGAACTTCGATCCAACATTGCTAGAAGTCTTTCCGAATTAGAGATGTTCACAGAGGATGGTGAAAATATTGATACACCCAAGAGGAAGAGTGCCATTAATGACCGAATGGAGGACCTTGTGAGTGCTCCTTTTGCAGTTGAAGATGCTCTCGTGGGTTTATTTGATCACAGTGATCACACCCTTCAGAGGAGGGTTGTGGAAAGTTATATACGTAGGCTCTATCAGCCATATCTTGTCAAAGGGAGTGCCAGGATGCAATGGCACAGATCTGGTCTTATTGCTACATGGGAGTTCTATGATGAGTACATTGAAAGGAAGAAT‑‑‑GGGGTTGAAGAC‑‑‑CAAAGTTTGAGTAAAACAGTGGAGGAGAAACATAGTGAGAAAAAGTGGGGAGTGATGGTTATAATTAAATCTCTTCAATTTTTGCCTGCAATTATCACTGCTGCATTGAGGGAGGCAACCAACAACCCTCACGAAGCA‑‑‑CTTACAAGTGGTTCTGTGGAACCAGTTAACTATGGTAATATGATGCATATTGGATTAGTGGGCATCAACAACCAGATGAGTTTACTGCAAGACAGTGGTGATGAGGATCAAGCTCAAGAAAGAATCAATAAGTTAGCTAAAATACTCAAAGAGCAGGAAGTAGGCTCCACCATACGAGCTGCAGGTGTCGGTGTAATCAGCTGTATCATACAAAGGGATGAAGGGCGTGCCCCAATGAGGCACTCCTTTCACTGGTCAGAAGAAAAGCTTTACTATGCGGAGGAACCTCTGTTGCGTCATCTGGAACCTCCGCTATCCATTTATCTTGAATTGGACAAACTTAAAGCCTATGAGAATATACGGTATACTCCATCTCGAGATCGTCAATGGCACCTCTACACAGTTGTGGATCACAAGCCACAACCAATTCAAAGAATGTTTCTTCGAACACTTGTAAGACAGCCGACAACAAATGAAGGATTTTCTTCATATCAAAGGCTGGATGCAGAAACATCTCGTACCCAATTGGCCATGTCCTTTACTTCAAGGAGCATTTTTAGGTCTTTGATGGCTGCAATGGAGGAGTTGGAACTTAATGCACACAATGTCAATATCAAATCTGAACATGCTCATATGTACCTATATATCATACGCGAACAACAAATAGATGATCTTGTGCCCTATCCCAAGAGAATTAACATAGAAGCTGGGAAAGAAGAAATAACAGTTGAGGCAGTCTTGGAAGAACTGGCACGGGAAATCCATTCATCTGTTGGTGTAAGAATGCATAGATTAGGGGTTGTTGTGTGGGAAATCAAGCTCTGGATGGCAGCCTGTGGACAGGCAAATGGTGCTTGGAGGGTCATTGTAAACAATGTGACTGGTCATACATGCACTGTACATCTATACCGAGAAAAGGAGGATACCATCACTCATAAAGTGGTATACAGTTCAGTATCGGTTAAGGGTCCACTGCATGGTGTGGCAGTGAATGAAAATTATCAACCTTTAGGAGTTATTGACCGAAAGCGTCTTTCAGCAAGAAAGAACAGCACCACATACTGCTATGATTTTCCTCTTGCATTTGAAACTGCCTTGGAACAGTCATGGGCAATCCAACAGCCAGGATTTCAAAGAGCCAAAGATAAAAATCTTTTAAAGGTAACAGAGCTTAAATTTGCAGATAAAGAAGGTAGCTGGGGTACTCCTCTTGTTCCTGTGGAGAATTACCCTGGGCTCAATGATGTTGGCATGGTAGCCTGGTTTATGGAAATGTGTACCCCTGAGTTCCCATCTGGAAGGACAATATTGGTTGTTGCAAATGATGTGACCTTCAAGGCTGGTTCTTTTGGCCCAAGAGAGGATGCATTCTTCCGTGCAGTAACTGATCTTGCATGTACAAAAAAACTACCTTTGATTTATTTAGCTGCAAATTCTGGTGCCCGTTTAGGTGTAGCTGAAGAAGTCAAATCCTGTTTCAGAGTTGGTTGGTCTGAGGAATCTAATCCTGAGAATGGCTTTCAGTATGTATATTTAACACCTGAGGATAATGCTCGGATTGGATCATCGGTGATAGCACATGAGTTAAAGCTTGAGAGTGGAGAAACCAGATGGGTTATAGATACCATTGTTGGCAAGGAGGATGGCCTTGGCGTTGAAAACTTGAGTGGAAGTGGGGCTATTGCTGGTGCTTATTCAAGGGCATACAAGGAAACTTTTACACTGACATATGTGACTGGTAGAACTGTTGGAATTGGAGCTTATCTTGCTAGGCTTGGGATGAGATGCATACAAAGGCTTGATCAGCCCATAATTCTTACTGGTTTTTCAGCATTAAACAAACTTCTTGGTCGGGAGGTTTACAGCTCTCACATGCAACTTGGTGGACCTAAAATCATGGCAACTAATGGAGTCGTTCATCTTACAGTTTCCGATGATCTTGAAGGTGTTTCTTCTATTTTGAAGTGGCTTAGCTACATTCCTTCTCATGTTGGCGGTGCACTTCCCATTGTAAAGCCACTTGATCCTCCAGAAAGACCAGTGGAGTATTTCCCAGAAAACTCGTGTGATCCTCGTGCTGCCATTTCTGGAACCTTGGATGGTAATGGAAGATGGCTGGGAGGAATTTTTGACAAAGACAGCTTTGTGGAGACACTAGAGGGATGGGCAAGAACAGTTGTTACAGGAAGGGCAAAGCTTGGAGGAATCCCTGTGGGAGTAGTTGCTGTAGAAACACAGACGGTAATGCAAATAATACCTGCTGATCCTGGCCAGCTTGATTCTCACGAGAGGGTTGTTCCTCAAGCCGGGCAAGTGTGGTTCCCTGATTCCGCAACCAAGACAGCCCAAGCAATATTGGATTTCAACAGAGAAGAGCTCCCGCTTTTCATTCTTGCAAACTGGAGAGGCTTTTCAGGAGGGCAAAGGGACCTTTTCGAAGGAATTCTTCAGGCTGGTTCCACTATTGTGGAGAACCTTAGAACATACAAGCAGCCCATATTTGTATATATCCCAATGATGGGCGAACTCCGTGGTGGGGCATGGGTGGTTGTTGACAGTAGAATCAATTCAGACCACATCGAAATGTATGCCGATCGAACAGCCAAAGGTAATGTCCTAGAGCCAGAAGGAATGATTGAGATCAAATTTAGAACAAGAGAATTGTTGGAGAGTATGGGTAGACTTGATCAGCAATTGATAACTCTGAAGGCAAAACTTCAGGAAGCCAAGAGTAGCAGGAACATTGTGGCCTTTGAATCCCTACAGCAGCAGATTAAATCACGCGAGAGACAACTTTTGCCTGTGTATACCCAGATAGCTACCAAGTTTGCTGAACTGCATGATACTTCCTTAAGAATGGCTGCCAAGGGTGTCATTAGAGAAGTTCTGGACTGGCGTAACTCCCGCTCTGTCTTTTACCAGAGACTGCACAGGAGAATTGGTGAGCAATCACTGATCAACAGTGTGAGAGATGCTGCT‑‑‑GGTGACCAATTGTCACATGCATCTGCAATGAACTTGCTCAAAGAATGGTATTTGAATTCCGATATTGCCAAAGGTAGAGAAGATGCTTGGTTGGATGATGAAGCCTTCTTCAGGTGGAAGGATATTCCTTCAAATTACGAGAATAAACTAAAGGAATTGCGTGTGCAGAAAGTGTTACTTCAATTGACAAATATTGGTGACTCAGCTCTAGATTTGCAAGCTCTACCTCAAGGTCTTGCTGCCCTTTTAAGCAAGTTGGAGCCATTAGGTCGCGTGAAGTTGACGGATGAACTTCGCAAGGTACTTGGT‑‑‑

>Gpo_ACC_VLNB‑2076277

‑‑‑‑‑‑‑‑‑‑‑‑‑‑‑‑‑‑‑‑‑‑‑‑‑‑‑‑‑‑‑‑‑‑‑‑‑‑‑‑‑‑‑‑‑‑‑‑‑‑‑‑‑‑‑‑‑‑‑‑‑‑‑‑‑‑‑‑‑‑‑‑‑‑‑‑‑‑‑‑‑‑‑‑‑‑‑‑‑‑‑‑‑‑‑‑‑‑‑‑‑‑‑‑‑‑‑‑‑‑‑‑‑‑‑‑‑‑‑‑‑‑‑‑‑‑‑‑‑‑‑‑‑‑‑‑‑‑‑‑‑‑‑‑‑‑‑‑‑‑‑‑‑‑‑‑‑‑‑‑‑‑‑‑‑‑‑‑‑‑‑‑‑‑‑‑‑‑‑‑‑‑‑‑‑‑‑‑‑‑‑‑‑‑‑‑‑‑‑‑‑‑‑‑‑‑‑‑‑‑‑‑‑‑‑‑‑‑‑‑‑‑‑‑‑‑‑‑‑‑‑‑‑‑‑‑‑‑‑‑‑‑‑‑‑‑‑‑‑‑‑‑‑‑‑‑‑‑‑‑‑‑‑‑‑‑‑‑‑‑‑‑‑‑‑‑‑‑‑‑‑‑‑‑‑‑‑‑‑‑‑‑‑‑‑‑‑‑‑‑‑‑‑‑‑‑‑‑‑‑‑‑‑‑‑‑‑‑‑‑‑‑‑‑‑‑‑‑‑‑‑‑‑‑‑‑‑‑‑‑‑‑‑‑‑‑‑‑‑‑‑‑‑‑‑‑‑‑‑‑‑‑‑‑‑‑ATGGCTGAAATAACTCATGTTGATGCGGTGTGGCCTGGTTGGGGTCATGCATCAGAGAATCCTGAGCTTCCAGATGCATTAAAAGCAAAAGGAATTGTATTTCTTGGACCTCCTGCTATATCCATGGCGGCATTGGGAGATAAAATTGGTTCGTCGTTGATTGCTCAAGCAGCAAAAGTGCCAACCCTTCCATGGAGTGGTTCTCATGTGAAAATTCCTCCCGACAGTAGCTTGATTACTATTCCTGATGAAGTTTATCGGGAAGCATGTGTTTATACAACAGAAGAAGCAATTGCTAGTTGTCAGATTGTAGGATACCCTGCAATGATCAAGGCATCTTGGGGTGGTGGTGGTAAAGGCATACGAAAGGTTCATAATGATGACGAGGTAAGGGCATTGTTCAAGCAAGTTCAAGGTGAAGTCCCTGGTTCACCTATATTTATAATGAAGGTTGCATCCCAGAGCCGACATCTAGAAGTCCAGTTACTCTGTGATCAGTATGGAAATGTTGCAGCTTTGCATAGTCGTGATTGCAGTGTTCAAAGAAGGCACCAAAAGATTATCGAGGAGGGTCCCATTACTGTGGCTCCTCTGGAAACGGTGAAACTACTAGAACAGGCAGCTAGAAGATTGGCTAAATCTGTAAATTATGTTGGGGCGGCTACTGTTGAGTATCTTTATAGTATGGAAACTGGCGAGTACTACTTTTTAGAATTAAACCCTCGACTACAGGTTGAGCACCCTGTTACTGAGTGGATAGCAGAGATAAATCTGCCAGCTGCACAAGTTGCCGTTGGGATGGGTATCCCTCTCTGGCAAATTCCTGAGATAAGGCGTTTCTATGGGGTGGAACATGGTGGAGGGAATGATGCTTGGAGAAAAACATCAGTTTTAGCTACCCCTTTTGATTTTGACAAAGCAAAATCTACAAGGCCAAAAGGTCATTGTGTGGCTGTGCGAGTGACTAGCGAGGACCCTGACGATGGTTTTAAGCCAACAAGCGGGAAAGTGCAGGAGCTCAGCTTTAAAAGCAAGCCAAATGTGTGGGCATACTTCTCTGTCAAGTCTGGAGGAGGAATCCATGAGTTCTCAGATTCTCAGTTTGGGCATGTTTTTGCTTTTGGAGAATCTAGGGCTTTAGCAATTGCAAATATGGTTCTGGGGCTGAAGGAGATTCAAATTCGGGGAGAAATTCGTACCAATGTTGATTACACCGTTGATCTTCTGAATGTTTCAGACTACAGAGAAAACAAAATTCACACAGGATGGCTGGACAGTAGAATTGCAATGCGGGTTAGAGCAGAGAGGCCTCCCTGGTATCTTTCTGTTGTTGGAGGGGCACTCTATAAAGCATCTGCTAGTAGTGCAGCTTTGGTTTCAGATTATGTTGGCTATCTTGAAAAGGGACAAATCCCTCCCAAGCACATATCTCTTGTCCATTCTCAAGTGTCCTTGAACATTGAAGGAAGCAAATACACGATTGACATGATACGAGGAGGACCTGGAAGTTATAAATTGATAATGAATCAATCAGAGATAGAAGCAGAGATACATACGTTACGTGATGGAGGTTTGCTGATGCAG‑‑‑‑‑‑‑‑‑TTGGATGGAAACAGTCATGTGATATATGCAGAGGAAGAAGCAGCTGGAACTCGCCTTCTAATAGATGGAAGGACTTGCTTGCTCCAGAATGATCATGATCCATCAAAGTTAATTGCGGAGACACCATGCAAGCTTCTGAGATATTTGGTCATGGATGAGAGTCATATTGATGCAGACACACCATATGCTGAAGTTGAGGTCATGAAGATGTGCATGCCTCTTCTTTCACCTGCTTCTGGGATTATTCACTTCAAAATGTCTGAAGGTCAAGCAATGCAGGCTGGTGAACTTATAGCAAGGCTCGATCTAGATGATCCTTCAGCTGTACGAAAGGCAGAACCCTTCAATGGGAGCTTTCCAATCCTGGGCCCTCCCACCGCAATTTCTGGTAAAGTTCATCAGAAATGCACCGCAAGCTTAAATGCTGCGCGGATGATTCTTGCTGGCTATGAGCACAATATTGATGAAGTTGTGCAAAGTTTGCTCAATTGCCTTGATAGTCCTGAATTGCCTTTCCTTCAATGGCAAGAGTGCATGGCAGTTTTGGCCACCCGTCTTCCCAAAGATCTAAAAAATGAGTTGGAATCAAAATATAAGGAGTTCGAAAGGATTTCA‑‑‑AGCTCCCAAATTGTTGATTTCCCTGCCAAATTATTGAAGGGAATTCTTGAAGCTCATCTTTCCTCCTGTCCTGACAAAGAAAAAGGAGCTCAAGAAAGACTAGTTGAACCTCTGATGACTCTTGTGAAGTCTTATGAGGGTGGAAGGGAGAGTCATGCCCATATAATTGTTCAATCCCTTTTTGAAGAGTATCTTTTTGTTGAAGAGCTATTTAGTGATAATATACGGGCTGATGTAATTGAACGTCTCCGTCTTCAATATAAGAAAGATCTATTGAAGATTGTAGATATTGTGCTCTCTCATCAGGGTGTCAAGAGTAAAAACAAGCTGATTCTGCGACTAATGGATAAGCTGGTTTACCCTAACCCTGCTGCCTATAGGGATCAATTGATCCGTTTTTCTGCACTCAACCATACAAATTATTCTGAGTTGGCTCTTAAGGCAAGTCAACTGCTGGAACAAACTAAATTGAGTGAACTTCGATCCAGCATTGCTAGAAGTCTTTCTGAGCTTGAAATGTTCACTGAGGATGGTGAAAGCATTGATACTCCCAAGAGGAAGAGTGCCATTAATGATCGAATGGAGGACCTTGTGAGTGCTCCTTTGGCGGTTGAAGATGCCCTTGTGGGTTTATTTGATCACAGTGATCACACCCTTCAAAGGAGGGTTGTGGAAACGTATGTACGCAGGCTCTACCAGCCATATCTTGTCAAAGGGAGTGTCAGGATGCAGTGGCACAGATCTGGTTTAATTGCTACATGGGAGTTCTTAGAAGAGTACATTGAAAGGAAGAGT‑‑‑GGGGTTGAAGAC‑‑‑CAAATGTCAAACAAAATGCTGGTTGAGAAACATAGTGAGAAGAAATGGGGAGTAATGGTTATAATTAAATCTCTTCAATTTTTACCTGCAATTATCAGTGCTGCATTAAGGGAAGCAACCAATAACCTTCACGAAGCA‑‑‑CTTACAAGTGGTTCTGGTGAACCAGTAAACTATGGTAATATGATGCATATTGGATTAGTGGGAATCAACAACCAGATGAGCTTACTGCAAGACAGTGGTGACGAGGATCAGGCTCAAGAAAGAATCAATAAGTTAGCCAAAATACTCAAGGAGCAGGAAGTAGGCTCTGCTATACGTGCTGCGGGTGTCGGAGTAATTAGCTGTATTATACAGAGGGATGAAGGGCGTGCCCCAATGAGGCACTCCTTTCACTGGTCAGTAGAAAAGCTCTATTATGAGGAGGAACCTCTGTTGCGTCATCTGGAACCTCCCCTATCCATTTATCTTGAATTGGACAAACTTAAAGGCTATGAGAATATACGATATACCCCATCCCGAGATCGTCAATGGCACCTGTACACAGTTGTGGATCACAAGCCCCAACCTATTCAAAGAATGTTTCTTCGAACACTTCTAAGACAACCAACTACTAATGAAGGATTCTCTACATATCAAAGGATGGATGCAGAAACATCTCGTACCCAATTGGCTATGTCCTTTACCTCAAGGAGCATTTTTAGGTCCTTGATGGCTGCAATGGAGGAGTTAGAACTTAATGCACACAATGCCACTATCAAATCTGAACATGCCCATATGTACCTCTATATCATACGAGAGCAACACATAAATGATCTTGTGCCATATCCCAAGAGAATTGACATAGATGCTGGCCAAGAAGAAACAACAGTTGAGTCAATCTTGGAAGAACTTGCACTGGAAATCCATTCATCTGTTGGTGTAAGAATGCATAGATTAGGGGTGGTTGTGTGGGAAGTCAAGCTCTGGATGGCAGCCTGTGGACAGGCAAATGGTGCTTGGAGGGTCATTGTAAACAATGTGACTGGTCATACATGCACTGTACATATATACCGAGAAATGGAGGATACCAGCACTCATAAAGTGGTATACAGTTCAGTCACTAAAAATGGTCCACTGCATGGTATACCAGTGAATGAAAACTATCAACCTTTGGGAGTTATTGACCGAAAACGTCTTTCAGCAAGAAAGAACAGCACCACATACTGCTATGATTTTCCCCTGGCATTTGAAACGGCCTTGGAACAGTCTTGGGCAATG‑‑‑‑‑‑CCAGGATTTGAAAGAGCCAAAGATAAAGACCTTATAAAAGTAACAGAGCTTAAGTTTGCTGATAAAGAAGGTAGTTGGGGCACTCCTCTTGTTCCTGTGGAGCGTTCTCCTGGACTCAATGATGTTGGCATGGTAGCCTGGTTTATGGAAATGTGTACCCCTGAGTTCCCATCTGGAAGGACAATATTGGTTGTTTCAAATGATGTGACCTTCAAGGCCGGTTCTTTTGGCCCAAGAGAGGATGCATTCTTTCGTGCAGTAACTGATCTTGCATGTGCAAAAAAGCTACCTTTGATTTATTTAGCTGCAAATTCTGGTGCCCGTTTAGGTGTAGCTGAAGAAGTCAAAGCCTGTTTTAGAGTTGGTTGGTCTGAGGAATCTAACCCTGAGCATGGTTTTCAGTATGTATACTTAACACCCGAGGATTATGCTCTGATGGGATCATCAGTGATAGCACATGAATTAAAGCTTGAAAGTGGAGAAACCAGATGGATTATAGATACCATTGTTGGCAAGGATGATGGCCTGGGGGTTGAAAACTTGAGTGGCAGTGGGGCCATTGCTGGTGCCTATTCAAGGGCATACAAGGAAACTTTTACATTGACGTATGTGACTGGTAGGACTGTTGGAATAGGTGCTTATCTTGCTAGGCTTGGGATGAGATGCATACAGAGGCTTGATCAGCCCATAATTCTTACTGGTTTTTCAGCATTAAATAAACTTCTTGGTCGGGAGGTATACAGCTCTCACATGCAACTTGGTGGACCTAAAATCATGGCAACTAATGGAGTTGTTCATCTTACAGTTTCAGATGACCTTGAAGGTGTTTCTTCTATTTTGAAGTGGCTTAGCTACATTCCTGCTCATGTAGGGGGTGCGCTTCCTATTGTAAAGCCACTTGATCCTCCAGAAAGACCAGTGGAGTATTTCCCAGAAAACTCGTGTGATCCTCGTGCTGCCATTTGTGGAACTCTGGATGGTAATGGAAGATGGATGGGGGGCATTTTTGACAAGGACAGCTTTGTGGAGACACTAGATGGATGGGCAAGGACAGTTGTTACAGGAAGGGCAAAGCTGGGAGGAATCCCTGTGGGAATTGTTGCTGTAGAAACACAGACAGTAATGCAAATAATACCTGCTGATCCAGGCCAGCTTGATTCTCATGAAAGGGTTGTTCCTCAAGCTGGGCAAGTATGGTTTCCTGATTCTGCGACCAAGACAGCCCAAGCAATATTGGATTTCAACAGAGAAGAGCTCCCACTTTTCATTCTTGCAAACTGGAGAGGCTTTTCAGGTGGGCAGAGGGACCTTTTTGAAGGAATTCTTCAGGCTGGTTCAACTATTGTGGAGAACCTTAGAACATACAAGCAGCCCATATTTGTATACATCCCAATGATGGGAGAACTCCGTGGTGGGGCATGGGTTGTTGTTGACAGTCGAATCAATTCAGATCACATTGAAATGTATGCTGATCGAACAGCTAAAGGTAATGTCCTTGAGCCTGAAGGAATGATTGAGATCAAATTTAGAACAAGAGAGTTGTTGGAGTGTATGGGTAGACTTGATCAACAGTTGATAACTCTGAAGGCAAAACTTCAGGATGCCAAGTGTAATAGGGACCTTGGGACCATTGAATCCCTACAGCAGCAGATTAAATCCCGTGAGAAACAACTCTTGCCTGTGTATACTCAGATAGCTACCAAATTTGCTGAACTGCATGATACTTCCTTAAGAATGGCTGCAAAGGGTGTAATTAGAGAAGTTCTGGACTGGGGAAATTCCCGTGCTGTCTTCTACCGGAGACTGCACAGGAGAATTGTGGAGCAGTCACTGATCAACAATCTGAGAGATGCTGCT‑‑‑GGTGACCATTTGTCACATACATCTGCAATGAACATGCTAAAAAACTGGTACTTGAATTCTGATAGTGTCAGAGGTAGAGAAGATGCATGGTTGGATGATGAAAACTTCTTCAGATGGAAGGATAATCCTGCAAATTACGAGGATAAACTAAAGGAATTGCGTGTCCAGAAAGTGTTGCTTCAATTGACAAGTATTGGTGATTCAATTCTAGATTTGCAAGCTCTACCTCAAGGTCTTGCTGCCCTTTTAAGCAAGTTGGAGCCATCAGGTCGTGTGAAGTTGAGTGATGAACTTCGCAAGGTACTTGGT‑‑‑

>Lal_ACC1_LAGI01_185

ATGGCTGGCATTGGCCGTGGGAAT‑‑‑‑‑‑‑‑‑GGGTACATAAATGGTATACAACCAAATAAGCACCCGACTACAATATCTAATATAGCCGAGTTCTGCAGCGCCCTTGGGGGAAATAAACCAATTCATAGCATATTGATTGCAAACAATGGAATGGCAGCAGTCAAGTTTATACGTAGTGTTAGGAGCTGGGCCTATGAGACCTTTGGCACAGAGAAGGCTATCCTGTTGGTTGCCATGGCTACACCAGAGGACATGAGGATCAATGCAGAACATATTAGAATAGCTGATCAATTTGTCGAAGTACCTGGTGGGACCAATAACAATAACTATGCCAATGTACAGCTTATTGTAGAG‑‑‑‑‑‑‑‑‑ATGGCTGAGATAACTCATGTTGATGCCGTATGGCCCGGTTGGGGTCATGCATCAGAGAATCCTGAGCTTCCTGATGCTTTGAAAGAAAAAGGAATTGTATTTCTTGGTCCTCCTGCTGTATCTATGGCAGCACTGGGAGATAAAATTGGTTCATCGTTGATTGCTCAAGCAGCGGAAGTGCCAACCCTTCCATGGAGTGGTTCTCATGTGAAAATACCTTTTGACAGTTGCTTGGTTACCATTCCTGATGAAATTTATCGGGAAGCATGTGTTTATACTACAGAAGAAGCAATTGCGAGTTGTCAAGTTGTAGGATACCCTGCAATGATCAAGGCATCTTGGGGTGGTGGTGGTAAAGGCATAAGAAAGGTTCATAATGATGATGAAGTAAGGGCATTGTTCAAGCAAGTTCAAGGCGAAGTTCCAGGCTCACCTATATTTATAATGAAGGTTGCATCGCAGAGCCGGCATCTAGAAGTCCAGTTACTTTGTGATCAATATGGAAATGTTGCAGCTTTGCATAGCCGTGATTGCAGTATTCAAAGGAGGCATCAAAAGATTATCGAGGAGGGCCCCATTACTGTAGCTCCTCCGGCAACGGTGAAACAACTAGAACAGGCAGCTAGAAGATTAGCAAAATCTGTAAATTATGTTGGTGCAGCTACTGTTGAGTATCTTTATAGCATGGAAACCGGCGAGTACTACTTTCTAGAGTTGAACCCCCGACTACAGGTTGAGCACCCGGTTACAGAGTGGATAGCAGAGATAAACCTACCAGCAGCACAAGTTGCCGTTGGGATGGGTATCCCTCTCTGGCAAATTCCTGAGATAAGGCGTTTCTATGGGGTGGAACATGGTGGGGGTAATGATGCTTGGAGGAAAACATCAGGTTTAGCTACTCCTTTTGATTTTGACAAGGTAGAATCAACAAGGCCAAAAGGTCATTGTGTGGCTGTGCGAGTCACAAGTGAGGACCCAGATGATGGTTTTAAGCCTACAAGCGGGAAAGTGCAGGAGCTAAGCTTTAAAAGCAAGCCAAATGTGTGGGCATACTTCTCTGTTAAGTCTGGAGGAGGAATCCACGAATTCTCAGATTCTCAGTTTGGGCATGTTTTTGCTTTTGGAGAATCTAGGGGTTTAGCTATTGCAAATATGGTTTTGGGTCTAAAGGAGATTCAAATTCGAGGAGAAATTCGTACCAATGTTGATTACACCATTGATCTTCTGAATGCTTCAGACTACAGAGATAACAAGATTCACACAGGGTGGCTGGACAGTAGAATTGCAATGCGGGTTAGAGCAGAGAGGCCTCCCTGGTATCTTTCTGTTGTTGGAGGGGCACTCTATAAAGCTTCTGCTTGTAGTGCAGCTTTGGTTTCAGACTATGTTGGTTATCTTGAAAAGGGACAAATCCCTCCCAAGCACTTGTCTCTTGTCCATTCTCAAGTGTCCTTGAACATAGAAGGAAGCAAATACACGATCGACATGATACGAGGAGGGTCTGGAAGTTATAGATTGAGAATGAATGAATCAGAGATAGAAGCAGAGATACATACTTTACGTGATGGAGGTTTGCTGATGCAG‑‑‑‑‑‑‑‑‑CTGAATGGAAACAGTCATATAATATATGCAGAGGAAGAAGCAGCTGGAACTCGCCTTCTAATAGATGGAAGGACTTGCTTACTTCAGAATGATCATGATCCATCAAAGTTAATTGCGGAGACTCCTTGCAAGCTTATGAGATATTTGGTTGTGGATGACAGTCATGTTGATGCTGACACACCATATGCTGAAGTTGAGGTCATGAAGATGTGCATGCCTCTTCTTTCGCCTGCTTCTGGAATTATTCATTTCAAAATGTCTGAAGGTCAAGCAATGCAGGCTGGTGAACTTATAGCAAGGCTTGATCTCGATGATCCTTCAGCTGTAAGAAAGGCAGAACCTTTCAATGGGAGCTTCCCTGTTCTGGGCCCTCCCACTGCAATTTCTGGTAGAGTTCATCAGAAATGTGCTGCAAGCTTAAATTCTGCACGCATGATTCTTGCTGGGTACGAGCACAATATTGATGATGTTGTACAAAGTTTGCTCCATTGCCTTGATAGTCCTGAATTGCCTTTTCTTCAATGGCAAGAGTGCTTCGCAGTTCTGGCAAATCGTCTTCCCAAAGATCTGAGAAGCGAGTTGGAATCAAAATATAAGGAGTTCGAGAGGATTTCA‑‑‑AGCACACAAATTGTTGATTTCCCTGCCAAATTATTGAAGGGATTTCTTGAAGCTCATCTTTCCTCTTGTCCCGACAAAGAAAAAGGAGCTCAAGAAAGATTAGTCGAACCTCTGCTGAGTCTTGTGAAGTCTTATGATGGTGGAAGAGAGAGTCATGCCCATATAATCGTTCAGTCCCTTTTTGAAGAGTATCTTTTTGTTGAAGAACTATTCAGTGATAATATACTGGCTGATGTTATTGAACGCCTCCGCCTTCAATATAAGAAAGATTTATTGAAGATTGTAGACATAGTGCTCTCTCATCAGGGTGTCAAGAGTAAAAATAAGCTGATACTGCAACTCATGGATAAACTGGTTTACCCTAACCCTGCTGCCTACAGAGATCAACTAATCCGTTTCTCTCAACTCAACCATACAAATTATTCTCAGTTGGCTCTTAAGGCAAGTCAACTGCTGGAACAAACTAAATTGAGTGAACTGCGGTCCAGCATTGCTAGAAGTCTTTCTGAACTAGAGATGTTCACCGAGGATGGTGAAACTATCGATACTCCCAAGAGGAAGAGTGCCATCAATGACCGAATGGAGGACCTTGTGAGTGCTCCTTTGGCAGTTGAAGATGCTCTTGTGGGTTTATTTGATCACAGCGATCACACCCTTCAAAGGAGGGTTGTGGAAACTTATGTACGCAGGCTCTACCAGCCATATCTTGTCAAAGGGAGTGTCAGGATGCAGTGGAACAGATCTGGTCTTATTGCTTCATGGGAGTTTTTAGAGGAGTATATTGAAGTGAAGAGT‑‑‑GGGGTCGAAAAC‑‑‑CAAATGTTAGAAAAAGCACCGGTGGTGAAACACAGTGAGAAGAAATGGGGAGTGATGGTTATAATTAAATCTCTTCAATTTTTACCTGCTATGATAAGTGATGCGCTAAGGGAAGCAAGCAGTAACCTACACGAAGCA‑‑‑CTTACAGGTGGTTCTGGCGAACCAGTTAAGTATGGTAATATGATGCATATTGCACTAGTGGGTATCAACAACCAGATGAGTTTACTGCAAGACAGTGGTGATGAGGATCAGGCTCAAGAAAGAATAAAAAAGTTAGCCAAAATTCTCAAAGATCAGGAAGTGGGTTCCACTATACGAGTTGCAGGTGTTGGAGTTATTAGCTGTATCATACAGAGGGATGAAGGGCGTGCACCGGTGAGGCACTCTTTTCACTGGTCAGCAGAAAAACACTATTATGAGGAGGAGCCTCTGTTGCGGCATTTGGAACCTCCCCTATCCATTTATCTTGAATTGGACAAACTTAAAGCCTATGAAAATATACGGTATACCCCATCTCGAGATCGTCAATGGCATCTGTACACAGTTACGGATAACAAACCACAATCAATTCAAAGAATGTTTCTTCGTACACTTCTAAGACAGCCAACCACAAATGAAGGGTTCTCTTTGTATCAAAGGCTGGATGCAGAAACATCTCGTACTCAATTGGCTATGTCCTTCACTTCCAGGAGCATTTTTAGGTCCTTGATGGCTGCAATGGAGGAGTTGGAACTTAATGCACACAATGCCAATACCAAATCTGAACATGCTCATATGTACCTATATATCATACGCCAGCAACAAATAGATGATCTTGTGCCTTACCCAAAGAGAATTGACATAGTTGATGGCCAAGAAGAAACAACAGTTGAGGCAATCTTAGAAGGACTGGCTCATGAAGTCCATTCTTCTGTTGGTGTAAGAATGCATAGATTGGGGGTTGTTGTGTGGGAAATCAAGCTCTGGATGGCAGCCTTTGGTCAGGCAAGTGGTGCTTGGAGGGTTATTGTAAACAACGTGACGGGTCATACATGCACTGTACATATATACCGAGAAATGGAGGATACCAGCACTCATAAAGTGGTATACAGTTCAGTAGGTGTAATGGGCCCGCTGCATGGTGTACCAGTGAGTGAAAATTATCAATCTTTGGGAGTTATTGACCGAAAACGTCTTTCAGCGAGAAAGAACAGCACCACATACTGCTATGATTTTCCCCTGGCATTTGAAACAGCCTTGGAACAGTCATGGGCAATCCAGCAGTCTGCATTTCAAAAAACCAAAGATACAGATCTTCTAAAAGCAACAGAGCTTAAATTTGCTGACAAAGAGGGTGGTTGGGGTACACCTCTTATTACTGTGGAGAATTCTCCTGGACTGAATGATGTTGGCATGGTAGCCTGGTTAATGGAAATGCGTACCCCTGAGTTTCCATCTGGAAGGACAATATTGGTTGTAGCAAACGATGTAACCTTCAAGGCTGGTTCTTTTGGCCCAAGAGAGGATGCATTCTTCCGTGCAGTAACTGATCTTGCATGTGAGAAAAAACTACCTTTAATTTATTTAGCAGCAAACTCTGGTGCCCGCTTAGGTGTAGCCGAGGAAGTCAAATCCTGTTTCAAAGTTGGTTGGTCTGAGGAATCTAGACCTGAGCATGGTTTTCAGTATGTATATTTAACACCTGAGGATTACGCTCGAATTGAATCATCAGTGATAGCACATGAATTAAAGCTTGAAAGTGGAGAAACCAGATGGGTCATAGATACCATTGTTGGGAAAGAGGATGGTCTGGGGGTTGAAAACTTGAGTGGTAGTGGGGCCATTGCCGGTGCCTATTCAAGGGCATACCAGGAAATTTTTACATTGACATATGTGACTGGCAGGACTGTTGGAATAGGGGCTTATCTTGCCAGGCTTGGGATGAGATGCATACAGAGGCTTGATCAACCCATAATTCTTACTGGTTTTTCAGCGCTAAATAAACTTCTCGGTCGGGAGGTATACAGCTCTCACATGCAACTTGGTGGACCTAAAATCATGGCAACTAATGGAGTTGTTCACCTTACAGTTTCGGATGACCTTGAAGGTGTTTCCGCTATTTTGAAGTGGCTTAGCTACGTTCCTTCTCATGTAGGTGGTAAGCTTCCCATTTTGAAGTCCCTTGATTCTCCAGAAAGACGAGTGGAGTACTTGCCTGAAAACTCATGTGATCCTCGTGCTGCCATTGCTGGAACTATGGATGGTAATGGAAGATGGCTAGGAGGCATTTTTGACAAGGACAGCTTTGTGGAGACATTAGAAGGATGGGCAAGGACAATTCCTACAGGAAGGGCAAAGCTTGGAGGAATTCCTGTGGGAATTGTTGCCGTGGAAACACAAACGGTAATGCAAATAATACCTGCTGATCCAGGTCAGCTTGATTCTCATGAGAGGGTTGTTCCTCAAGCTGGGCAAGTGTGGTTTCCTGATTCTGCTACCAAGACAGCCCAAGCAATATTGGATTTCAACAGAGAAGAGCTCCCACTTTTCATTCTTGCAAATTGGAGAGGCTTTTCAGGTGGGCAGAGGGACCTTTTTGAAGGAATTCTTCAGGCTGGTTCAACCATTGTAGAGAACCTTAGGACATACAAGCAGCCCATATTTGTGTACATCCCAATGATGGGCGAACTCCGTGGTGGGGCGTGGGTTGTTGTTGACAGTCGAATCAATTCAGACCACATTGAAATGTATGCCGATCGAACAGCTAAAGGCAATGTCCTGGAGCCAGAAGGAATGATTGAGATCAAATTCAGAACAAGAGAATTGTTGGAGCGCATGGGCAGACTGGATCAACAGTTGATAACTCTGAAAGCAAAACTTCAGGAGGCCAAAAGTAACAGGGAAATTGAGACCATTGAATCCCTACAGCAGCAGATTAAGTCCCGTGAGAAACAGCTTTTGCCCATGTATACCCAGATAGCTACCAAATTTGCTGAACTGCATGATACTTCCTTAAGAATGGCTGCAAAGGGGGTGGTAAGAGAAGTTTTGGACTGGGGTAACTCCCGTGCTGTCTTCTACCGAAGATTGCACAGAAGAATTGGTGAGCAGACACTGATCAACAGTTTTAGAGATGCTGCAGGTGGCGACCATTTGTCACATGTATCCGCGTTGGAATTGCTCAAAAACTTGTATCTGAACTCTGATATCGCCAAAGGTAGAGAAGATGCCTGGTTAGACGATGAAGCCTTCTTCAGATGGAAGGATAATCCTACAAACTATGAGGATAAACTAAAGGAATTGCGTGCCCAGAAAGTATTGCTTCAACTGACAAATATCGGTGACTCAGTTCTAGATATGCAAGCTTTACCCCAAGGTCTTGCTGCCCTTTTAAGCAAGTTGGAGCCATCAAGCCGTGCGAAGTTGACGGATGAACTTCGTAAGGTACTTGGT‑‑‑

>Lal_ACC2_LAGI01_188

ATGGCTGGCATTGGCCGTGGGAAT‑‑‑‑‑‑‑‑‑GGGTACATAAATGGTATACAACCAAATAAGCACCCGACTACAATATCTAATATAGCCGAGTTCTGCAGCGCCCTTGGGGGAAATAAACCAATTCATAGCATATTGATTGCAAACAATGGAATGGCAGCAGTCAAGTTTATACGTAGTGTTAGGAGCTGGGCCTATGAGACCTTTGGCACAGAGAAGGCTATCCTGTTGGTTGCCATGGCTACACCAGAGGACATGAGGATCAATGCAGAACATATTAGAATAGCTGATCAATTTGTCGAAGTACCTGGTGGGACCAATAACAATAACTATGCCAATGTACAGCTTATTGTAGAG‑‑‑‑‑‑‑‑‑ATGGCTGAGATAACTCATGTTGATGCCGTATGGCCCGGTTGGGGTCATGCATCAGAGAATCCTGAGCTTCCTGATGCTTTGAAAGAAAAAGGAATTGTATTTCTTGGTCCTCCTGCTGTATCTATGGCAGCACTGGGAGATAAAATTGGTTCATCGTTGATTGCTCAAGCAGCGGAAGTGCCAACCCTTCCATGGAGTGGTTCTCATGTGAAAATACCTTTTGACAGTTGCTTGGTTACCATTCCTGATGAAATTTATCGGGAAGCATGTGTTTATACTACAGAAGAAGCAATTGCGAGTTGTCAAGTTGTAGGATACCCTGCAATGATCAAGGCATCTTGGGGTGGTGGTGGTAAAGGCATAAGAAAGGTTCATAATGATGATGAAGTAAGGGCATTGTTCAAGCAAGTTCAAGGCGAAGTTCCAGGCTCACCTATATTTATAATGAAGGTTGCATCGCAGAGCCGGCATCTAGAAGTCCAGTTACTTTGTGATCAATATGGAAATGTTGCAGCTTTGCATAGCCGTGATTGCAGTATTCAAAGGAGGCATCAAAAGATTATCGAGGAGGGCCCCATTACTGTAGCTCCTCCGGCAACGGTGAAACAACTAGAACAGGCAGCTAGAAGATTAGCAAAATCTGTAAATTATGTTGGTGCAGCTACTGTTGAGTATCTTTATAGCATGGAAACCGGCGAGTACTACTTTCTAGAGTTGAACCCCCGACTACAGGTTGAGCACCCGGTTACAGAGTGGATAGCAGAGATAAACCTACCAGCAGCACAAGTTGCCGTTGGGATGGGTATCCCTCTCTGGCAAATTCCTGAGATAAGGCGTTTCTATGGGGTGGAACATGGTGGGGGTAATGATGCTTGGAGGAAAACATCAGGTTTAGCTACTCCTTTTGATTTTGACAAGGTAGAATCAACAAGGCCAAAAGGTCATTGTGTGGCTGTGCGAGTCACAAGTGAGGACCCAGATGATGGTTTTAAGCCTACAAGCGGGAAAGTGCAGGAGCTAAGCTTTAAAAGCAAGCCAAATGTGTGGGCATACTTCTCTGTTAAGTCTGGAGGAGGAATCCACGAATTCTCAGATTCTCAGTTTGGGCATGTTTTTGCTTTTGGAGAATCTAGGGGTTTAGCTATTGCAAATATGGTTTTGGGTCTAAAGGAGATTCAAATTCGAGGAGAAATTCGTACCAATGTTGATTACACCATTGATCTTCTGAATGCTTCAGACTACAGAGATAACAAGATTCACACAGGGTGGCTGGACAGTAGAATTGCAATGCGGGTTAGAGCAGAGAGGCCTCCCTGGTATCTTTCTGTTGTTGGAGGGGCACTCTATAAAGCTTCTGCTTGTAGTGCAGCTTTGGTTTCAGACTATGTTGGTTATCTTGAAAAGGGACAAATCCCTCCCAAGCACTTGTCTCTTGTCCATTCTCAAGTGTCCTTGAACATAGAAGGAAGCAAATACACGATCGACATGATACGAGGAGGGTCTGGAAGTTATAGATTGAGAATGAATGAATCAGAGATAGAAGCAGAGATACATACTTTACGTGATGGAGGTTTGCTGATGCAG‑‑‑‑‑‑‑‑‑CTGAATGGAAACAGTCATATAATATATGCAGAGGAAGAAGCAGCTGGAACTCGCCTTCTAATAGATGGAAGGACTTGCTTACTTCAGAATGATCATGATCCATCAAAGTTAATTGCGGAGACTCCTTGCAAGCTTATGAGATATTTGGTTGTGGATGACAGTCATGTTGATGCTGACACACCATATGCTGAAGTTGAGGTCATGAAGATGTGCATGCCTCTTCTTTCGCCTGCTTCTGGAATTATTCATTTCAAAATGTCTGAAGGTCAAGCAATGCAGGCTGGTGAACTTATAGCAAGGCTTGATCTCGATGATCCTTCAGCTGTAAGAAAGGCAGAACCTTTCAATGGGAGCTTCCCTGTTCTGGGCCCTCCCACTGCAATTTCTGGTAGAGTTCATCAGAAATGTGCTGCAAGCTTAAATTCTGCACGCATGATTCTTGCTGGGTACGAGCACAATATTGATGATGTTGTACAAAGTTTGCTCCATTGCCTTGATAGTCCTGAATTGCCTTTTCTTCAATGGCAAGAGTGCTTCGCAGTTCTGGCAAATCGTCTTCCCAAAGATCTGAGAAGCGAGTTGGAATCAAAATATAAGGAGTTCGAGAGGATTTCA‑‑‑AGCACACAAATTGTTGATTTCCCTGCCAAATTATTGAAGGGATTTCTTGAAGCTCATCTTTCCTCTTGTCCCGACAAAGAAAAAGGAGCTCAAGAAAGATTAGTCGAACCTCTGCTGAGTCTTGTGAAGTCTTATGATGGTGGAAGAGAGAGTCATGCCCATATAATCGTTCAGTCCCTTTTTGAAGAGTATCTTTTTGTTGAAGAACTATTCAGTGATAATATACTGGCTGATGTTATTGAACGCCTCCGCCTTCAATATAAGAAAGATTTATTGAAGATTGTAGACATAGTGCTCTCTCATCAGGGTGTCAAGAGTAAAAATAAGCTGATACTGCAACTCATGGATAAACTGGTTTACCCTAACCCTGCTGCCTACAGAGATCAACTAATCCGTTTCTCTCAACTCAACCATACAAATTATTCTCAGTTGGCTCTTAAGGCAAGTCAACTGCTGGAACAAACTAAATTGAGTGAACTGCGGTCCAGCATTGCTAGAAGTCTTTCTGAACTAGAGATGTTCACCGAGGATGGTGAAACTATCGATACTCCCAAGAGGAAGAGTGCCATCAATGACCGAATGGAGGACCTTGTGAGTGCTCCTTTGGCAGTTGAAGATGCTCTTGTGGGTTTATTTGATCACAGCGATCACACCCTTCAAAGGAGGGTTGTGGAAACTTATGTACGCAGGCTCTACCAGCCATATCTTGTCAAAGGGAGTGTCAGGATGCAGTGGAACAGATCTGGTCTTATTGCTTCATGGGAGTTTTTAGAGGAGTATATTGAAGTGAAGAGT‑‑‑GGGGTCGAAAAC‑‑‑CAAATGTTAGAAAAAGCACCGGTGGTGAAACACAGTGAGAAGAAATGGGGAGTGATGGTTATAATTAAATCTCTTCAATTTTTACCTGCTATGATAAGTGATGCGCTAAGGGAAGCAAGCAGTAACCTACACGAAGCA‑‑‑CTTACAGGTGGTTCTGGCGAACCAGTTAAGTATGGTAATATGATGCATATTGCACTAGTGGGTATCAACAACCAGATGAGTTTACTGCAAGACAGTGGTGATGAGGATCAGGCTCAAGAAAGAATAAAAAAGTTAGCCAAAATTCTCAAAGATCAGGAAGTGGGTTCCACTATACGAGTTGCAGGTGTTGGAGTTATTAGCTGTATCATACAGAGGGATGAAGGGCGTGCACCGGTGAGGCACTCTTTTCACTGGTCAGCAGAAAAACACTATTATGAGGAGGAGCCTCTGTTGCGGCATTTGGAACCTCCCCTATCCATTTATCTTGAATTGGACAAACTTAAAGCCTATGAAAATATACGGTATACCCCATCTCGAGATCGTCAATGGCATCTGTACACAGTTACGGATAACAAACCACAATCAATTCAAAGAATGTTTCTTCGTACACTTCTAAGACAGCCAACCACAAATGAAGGGTTCTCTTTGTATCAAAGGCTGGATGCAGAAACATCTCGTACTCAATTGGCTATGTCCTTCACTTCCAGGAGCATTTTTAGGTCCTTGATGGCTGCAATGGAGGAGTTGGAACTTAATGCACACAATGCCAATATCAAATCTGAACATGCTCATATGTACCTCTATATCATACGCGAGCAGCAAATAGATGATCTTGTGCCTTATACAAAGAAAATTGACGTAGATGCTGCCCAAGAAGAAATAACAGTTAAAACAATCTTAGAAGGACTGGCACATGAAGTCCATTCATCTGTTGGTGTAAGAATGCATAGATTAGGGGTTGTTGTATGGGAAATCAAGCTCTGGATGGCAGCCTGTGGTCAAGCAAATGGTGCTTGGAGGGTTATTGTAAACAACGTGACGGGTCATACATGCACTGTACATATATACCGAGAAATGGAGGATACCAGCACTCATAAAGTGGTATACAGTTCAGTAGGTGTAATGGGCCCGCTGCATGGTGTACCAGTGAGTGAAAATTATCAATCTTTGGGAGTTATTGACCGAAAACGTCTTTCAGCGAGAAAGAACAGCACCACATACTGCTATGATTTTCCCCTGGCATTTGAAACAGCCTTGGAACAGTCATGGGCAATCCAGCAGTCTGCATTTCAAAAAACCAAAGATACAGATCTTCTAAAAGCAACAGAGCTTAAATTTGCTGACAAAGAAGGTGGTTGGGGTACACCTCTTATTACTGTGGAGAATTCTCCTGGACTGAATGATGTTGGCATGGTAGCCTGGTTAATGGAAATGCGTACCCCTGAGTTTCCATCTGGAAGGACAATATTGGTTGTAGCAAACGATGTAACCTTCAAGGCTGGTTCTTTTGGCCCAAGAGAGGATGCATTCTTCCGTGCAGTAACTGATCTTGCATGTGAGAAAAAACTACCTTTAATTTATTTAGCAGCAAACTCTGGTGCCCGCTTAGGTGTAGCCGAGGAAGTCAAATCCTGTTTCAAAGTTGGTTGGTCTGAGGAATCTAGACCTGAGCATGGTTTTCAGTATGTATATTTAACACCTGAGGATTACGCTCGAATTGAATCATCAGTGATAGCACATGAATTAAAGCTTGAAAGTGGAGAAACCAGATGGGTCATAGATACCATTGTTGGGAAAGAGGATGGCCTAGGGGTTGAAAACTTAAGTGGTAGTGGTGCCATTGCTGGTGCCTATTCAAGGGCATACAAGGAAACTTTTACATTGACATATGTGACTGGCAGGACTGTCGGAATAGGGGCTTATCTTGCCAGGCTCGGGATGAGATGCATACAGAGGCTTGATCAGCCCATAATTCTTACCGGGTTTTCAGCGTTAAATAAACTTCTTGGTCGCGAGGTATACAGCTCCCATATGCAACTTGGTGGACCTAAAATCATGGCAACTAATGGAGTTGTTCACCTTACAGTTTCGGATGACCTTGAAGGTGTTTCCGCTATTTTGAAGTGGCTTAGCTACATTCCTTCTCATGTAGGTGGTAAGCTTCCCATTGTAAAGTCCCTTGATCCTCCGGAAAGAGAAGTGGAGTACTTGCCAGAAAACTCATGTGATCCTCGTGCTGCCATTGCTGGAACTGTGGATGGTAATGGAAGATGGCTAGGAGGCATTTTTGACAAGGACAGTTTTGTGGAGACATTAGAAGGATGGGCAAGGACAGTTGTTACAGGAAGGGCAAAGCTTGGAGGAATTCCTGTGGGAATTGTTGCTGTGGAAACACAAACGGTAATGCAAATAATACCTGCTGATCCAGGTCAGCTTGATTCTCATGAGAGGGTTGTTCCTCAAGCTGGGCAGGTGTGGTTTCCTGATTCTGCTGCCAAGACAGCCCAATCAATATTGGATTTCAACAGAGAAGAGCTCCCACTTTTTATTCTTGCAAATTGGAGAGGCTTTTCAGGTGGGCAGAGGGACCTTTTTGAAGGAATTCTTCAGGCTGGTTCAACCATTGTAGAGAACCTTAGGACATACAAGCAGCCCATATTTGTGTACATCCCAATGATGGGCGAACTCCGTGGTGGGGCGTGGGTTGTTGTTGACAGTCGAATCAATTCAGACCCCATTGAAATGTATGCTGATCGAACAGCTAAAGGCAATGTCCTGGAGCCAGAAGGGATGATTGAGATCAAATTCAGAACAAGAGAATTGTTGGAGTGCATGGGTAGACTGGATCAACAGTTGATAACTCTGAAAGCAAAACTTCAGGAGGCCAAAAGTAACAGGGAAATTGTGACCATTGAATCCCTACAGCAGCAGATTAAGTCCCGTGAGAAACAGCTTTTGCCCATGTATACCCAGATAGCTACCAAATTTGCTGAACTGCATGATACTTCCTTAAGAATGGCTGCAAAGGGGGTGGTAAGAGAAGTTTTGGACTGGGGTAACTCCCGTGCTGTCTTCTACCGAAGATTGCACAGAAGAATTGGTGAGCAGACACTGATCAACAGTTTTAGAGATGCTGCAGGTGGCGACCATTTGTCACATGTATCCGCGTTGGAATTGCTCAAAAACTTGTATCTGAACTCTGATATCGCCAAAGGTAGAGAAGATGCCTGGTTAGACGATGAAGCCTTCTTCAGATGGAAGGATAATCCTACAAACTATGAGGATAAACTAAAGGAATTGCGTGCTCAGAAAGTGTTGCTTCAACTGACAACTATTGGTGACTCAGTTCTAGATATGCAAGCTTTACCTCAAGGTCTTGCTGCCTTTTTAAGCAAGTTGGAGCCATCAAGCCGTGCGAAGTTGACGGATGAACTTCGTAAGGTACTTGGT‑‑‑

>Lal_ACC3_LAGI01_739

ATGGCTGGCATTGGCCGTGGGAAT‑‑‑‑‑‑‑‑‑GGGTACATAAATGGTATACAACCAAATAAGCACCCGACTACAATATCTAATATAGCCGAGTTCTGCAGCGCCCTTGGGGGAAATAAACCAATTCATAGCATATTGATTGCAAACAATGGAATGGCAGCAGTCAAGTTTATACGTAGTGTTAGGAGCTGGGCCTATGAGACCTTTGGCACAGAGAAGGCTATCCTGTTGGTTGCCATGGCTACACCAGAGGACATGAGGATCAATGCAGAACATATTAGAATAGCTGATCAATTTGTCGAAGTACCTGGTGGGACCAATAACAATAACTATGCCAATGTACAGCTTATTGTAGAG‑‑‑‑‑‑‑‑‑ATGGCTGAGATAACTCATGTTGATGCCGTATGGCCCGGTTGGGGTCATGCATCAGAGAATCCTGAGCTTCCTGATGCTTTGAAAGAAAAAGGAATTGTATTTCTTGGTCCTCCTGCTGTATCTATGGCAGCACTGGGAGATAAAATTGGTTCATCGTTGATTGCTCAAGCAGCGGAAGTGCCAACCCTTCCATGGAGTGGTTCTCATGTGAAAATACCTTTTGACAGTTGCTTGGTTACCATTCCTGATGAAATTTATCGGGAAGCATGTGTTTATACTACAGAAGAAGCAATTGCGAGTTGTCAAGTTGTAGGATACCCTGCAATGATCAAGGCATCTTGGGGTGGTGGTGGTAAAGGCATAAGAAAGGTTCATAATGATGATGAAGTAAGGGCATTGTTCAAGCAAGTTCAAGGCGAAGTTCCAGGCTCACCTATATTTATAATGAAGGTTGCATCGCAGAGCCGGCATCTAGAAGTCCAGTTACTTTGTGATCAATATGGAAATGTTGCAGCTTTGCATAGCCGTGATTGCAGTATTCAAAGGAGGCATCAAAAGATTATCGAGGAGGGCCCCATTACTGTAGCTCCTCCGGCAACGGTGAAACAACTAGAACAGGCAGCTAGAAGATTAGCAAAATCTGTAAATTATGTTGGTGCAGCTACTGTTGAGTATCTTTATAGCATGGAAACCGGCGAGTACTACTTTCTAGAGTTGAACCCCCGACTACAGGTTGAGCACCCGGTTACAGAGTGGATAGCAGAGATAAACCTACCAGCAGCACAAGTTGCCGTTGGGATGGGTATCCCTCTCTGGCAAATTCCTGAGATAAGGCGTTTCTATGGGGTGGAACATGGTGGGGGTAATGATGCTTGGAGGAAAACATCAGGTTTAGCTACTCCTTTTGATTTTGACAAGGTAGAATCAACAAGGCCAAAAGGTCATTGTGTGGCTGTGCGAGTCACAAGTGAGGACCCAGATGATGGTTTTAAGCCTACAAGCGGGAAAGTGCAGGAGCTAAGCTTTAAAAGCAAGCCAAATGTGTGGGCATACTTCTCTGTTAAGTCTGGAGGAGGAATCCACGAATTCTCAGATTCTCAGTTTGGGCATGTTTTTGCTTTTGGAGAATCTAGGGGTTTAGCTATTGCAAATATGGTTTTGGGTCTAAAGGAGATTCAAATTCGAGGAGAAATTCGTACCAATGTTGATTACACCATTGATCTTCTGAATGCTTCAGACTACAGAGATAACAAGATTCACACAGGGTGGCTGGACAGTAGAATTGCAATGCGGGTTAGAGCAGAGAGGCCTCCCTGGTATCTTTCTGTTGTTGGAGGGGCACTCTATAAAGCTTCTGCTTGTAGTGCAGCTTTGGTTTCAGACTATGTTGGTTATCTTGAAAAGGGACAAATCCCTCCCAAGCACTTGTCTCTTGTCCATTCTCAAGTGTCCTTGAACATAGAAGGAAGCAAATACACGATCGACATGATACGAGGAGGGTCTGGAAGTTATAGATTGAGAATGAATGAATCAGAGATAGAAGCAGAGATACATACTTTACGTGATGGAGGTTTGCTGATGCAG‑‑‑‑‑‑‑‑‑CTGAATGGAAACAGTCATATAATATATGCAGAGGAAGAAGCAGCTGGAACTCGCCTTCTAATAGATGGAAGGACTTGCTTACTTCAGAATGATCATGATCCATCAAAGTTAATTGCGGAGACTCCTTGCAAGCTTATGAGATATTTGGTTGTGGATGACAGTCATGTTGATGCTGACACACCATATGCTGAAGTTGAGGTCATGAAGATGTGCATGCCTCTTCTTTCGCCTGCTTCTGGAATTATTCATTTCAAAATGTCTGAAGGTCAAGCAATGCAGGCTGGTGAACTTATAGCAAGGCTTGATCTCGATGATCCTTCAGCTGTAAGAAAGGCAGAACCTTTCAATGGGAGCTTCCCTGTTCTGGGCCCTCCCACTGCAATTTCTGGTAGAGTTCATCAGAAATGTGCTGCAAGCTTAAATTCTGCACGCATGATTCTTGCTGGGTACGAGCACAATATTGATGATGTTGTACAAAGTTTGCTCCATTGCCTTGATAGTCCTGAATTGCCTTTTCTTCAATGGCAAGAGTGCTTCGCAGTTCTGGCAAATCGTCTTCCCAAAGATCTGAGAAGCGAGTTGGAATCAAAATATAAGGAGTTCGAGAGGATTTCA‑‑‑AGCACACAAATTGTTGATTTCCCTGCCAAATTATTGAAGGGATTTCTTGAAGCTCATCTTTCCTCTTGTCCCGACAAAGAAAAAGGAGCTCAAGAAAGATTAGTCGAACCTCTGCTGAGTCTTGTGAAGTCTTATGATGGTGGAAGAGAGAGTCATGCCCATATAATCGTTCAGTCCCTTTTTGAAGAGTATCTTTTTGTTGAAGAACTATTCAGTGATAATATACTGGCTGATGTTATTGAACGCCTCCGCCTTCAATATAAGAAAGATTTATTGAAGATTGTAGACATAGTGCTCTCTCATCAGGGTGTCAAGAGTAAAAATAAGCTGATACTGCAACTCATGGATAAACTGGTTTACCCTAACCCTGCTGCCTACAGAGATCAACTAATCCGTTTCTCTCAACTCAACCATACAAATTATTCTCAGTTGGCTCTTAAGGCAAGTCAACTGCTGGAACAAACTAAATTGAGTGAACTGCGGTCCAGCATTGCTAGAAGTCTTTCTGAACTAGAGATGTTCACTGAGGATGGTGAAACTATTGATACTCCCAAGAGGAAGAGTGCTATTAATGACCGAATGGAGGACCTTGTGAGTGCTCCTTTGGCAGTTGAAGATGCTCTTGTGGGTTTATTTGATCACAGCGATCACACCCTTCAAAGGAGGGTTGTGGAAACTTATGTACGCAGGCTCTACCAGCCATATCTTGTCAAAGGGAGTGTCAGGATGCAGTGGAACAGATCTGGTCTTATTGCTTCATGGGAGTTCTTAGAGGAGTACATTGAAAGGAAGAGT‑‑‑GGGGTCGAAGAC‑‑‑CAAATGTCAGAAAAGACACCAGTGGGGAAACACGATAAGAAGAAATGGGGAGTGATGGTTATACTTAAATCTCTTCAATTTTTACCTGCAATGATAAGTGATGCCCTAAGGGAAGCAAGCAGTAACTTAAACGAAGCA‑‑‑CTTACA‑‑‑‑‑‑‑‑‑AGTGAACCAATTAAGTACGGTAATATGATGCATATTGCACTGGTGGGTATCAACAACCAGATGAGTTTACTGCAAGACAGTGGTGATGAGGATCAGGCTCAAGAAAGAATAAAAAAGTTAGCCAAAATTCTCAAAGATCAGGAAGTGGGTTCCACTATACGAGTTGCAGGTGTTGGAGTTATTAGCTGTATCATACAGAGGGATGAAGGGCGTGCACCGGTGAGGCACTCTTTTCACTGGTCAGCAGAAAAACACTATTATGAGGAGGAGCCTCTGTTGCGGCATTTGGAACCTCCCCTATCCATTTATCTTGAATTGGACAAACTTAAAGCCTATGAAAATATACGGTATACCCCATCTCGAGATCGTCAATGGCATCTGTACACAGTTACGGATAACAAACCACAATCAATTCAAAGAATGTTTCTTCGTACACTTCTAAGACAGCCAACCACAAATGAAGGGTTCTCTTTGTATCAAAGGCTGGATGCAGAAACATCTCGTACTCAATTGGCTATGTCCTTCACTTCCAGGAGCATTTTTAGGTCCTTGATGGCTGCAATGGAGGAGTTGGAACTTAATGCACACAATGCCAATACCAAATCTGAACATGCTCATATGTACCTATATATCATACGCCAGCAACAAATAGATGATCTTGTGCCTTACCCAAAGAGAATTGACATAGTTGATGGCCAAGAAGAAACAACAGTTGAGGCAATCTTAGAAGGACTGGCTCATGAAGTCCATTCTTCTGTTGGTGTAAGAATGCATAGATTGGGGGTTGTTGTGTGGGAAATCAAGCTCTGGATGGCAGCCTTTGGTCAGGCAAGTGGTGCTTGGAGGGTTATTGTAAACAACGTGACGGGTCATACATGCACTGTACATATATACCGAGAAATGGAGGATACCAGCACTCATAAAGTGGTATACAGTTCAGTAGGTGTAATGGGCCCGCTGCATGGTGTACCAGTGAGTGAAAATTATCAATCTTTGGGAGTTATTGACCGAAAACGTCTTTCAGCGAGAAAGAACAGCACCACATACTGCTATGATTTTCCCCTGGCATTTGAAACAGCCTTGGAACAGTCATGGGCAATCCAGCAGTCTGCATTTCAAAAAACCAAAGATACAGATCTTCTAAAAGCAACAGAGCTTAAATTTGCTGACAAAGAAGGTGGTTGGGGTACACCTCTTATTACTGTGGAGAATTCTCCTGGACTGAATGATGTTGGCATGGTAGCCTGGTTAATGGAAATGCGTACCCCTGAGTTTCCATCTGGAAGGACAATATTGGTTGTAGCAAACGATGTAACCTTCAAGGCTGGTTCTTTTGGCCCAAGAGAGGATGCATTCTTCCGTGCAGTAACTGATCTTGCATGTGAGAAAAAACTACCTTTAATTTATTTAGCAGCAAACTCTGGTGCCCGCTTAGGTGTAGCCGAGGAAGTCAAATCCTGTTTCAAAGTTGGTTGGTCTGAGGAATCTAGACCTGAGCATGGTTTTCAGTATGTATATTTAACACCTGAGGATTACGCTCGAATTGAATCATCAGTGATAGCACATGAATTAAAGCTTGAAAGTGGAGAAACCAGATGGGTCATAGATACCATTGTTGGGAAAGAGGATGGCCTAGGGGTTGAAAACTTAAGTGGTAGTGGTGCCATTGCTGGTGCCTATTCAAGGGCATACAAGGAAACTTTTACATTGACATATGTGACTGGCAGGACTGTCGGAATAGGGGCTTATCTTGCCAGGCTCGGGATGAGATGCATACAGAGGCTTGATCAGCCCATAATTCTTACCGGGTTTTCAGCGTTAAATAAACTTCTTGGTCGCGAGGTATACAGCTCCCATATGCAACTTGGTGGACCTAAAATCATGGCAACTAATGGAGTTGTTCACCTTACAGTTTCGGATGACCTTGAAGGTGTTTCCGCTATTTTGAAGTGGCTTAGCTACATTCCTTCTCATGTAGGTGGTAAGCTTCCCATTGTAAAGTCCCTTGATCCTCCGGAAAGAGAAGTGGAGTACTTGCCAGAAAACTCATGTGATCCTCGTGCTGCCATTGCTGGAACTGTGGATGGTAATGGAAGATGGCTAGGAGGCATTTTTGACAAGGACAGTTTT‑‑‑‑‑‑‑‑‑‑‑‑‑‑‑‑‑‑‑‑‑‑‑‑‑‑‑‑‑‑‑‑‑‑‑‑‑‑‑‑‑‑‑‑‑‑‑‑‑‑‑‑‑‑‑‑‑‑‑‑‑‑‑‑‑‑‑‑‑‑‑‑‑‑‑‑‑‑‑‑‑‑‑‑‑‑‑‑‑‑‑‑‑‑‑‑‑‑‑‑‑‑‑‑‑‑‑‑‑‑‑‑‑‑‑‑‑‑‑‑‑‑‑‑‑‑‑‑‑‑‑‑‑‑‑‑‑‑‑‑‑‑‑‑‑‑‑‑‑‑‑‑‑‑‑‑‑‑‑‑‑‑‑‑‑‑‑‑‑‑‑‑‑‑‑‑‑‑‑‑‑‑‑‑‑‑‑‑‑‑‑‑‑‑‑‑‑‑‑‑‑‑‑‑‑‑‑‑‑‑‑‑‑‑‑‑‑‑‑‑‑‑‑‑‑‑‑‑‑‑‑‑‑‑‑‑‑‑‑‑‑‑‑‑‑‑‑‑‑‑‑‑‑‑‑‑‑‑‑‑‑‑‑‑‑‑‑‑‑‑‑‑‑‑‑‑‑‑‑‑‑‑‑‑‑‑‑‑‑‑‑‑‑‑‑‑‑‑‑‑‑‑‑‑‑‑‑‑‑‑‑‑‑‑‑‑‑‑‑‑‑‑‑‑‑‑‑‑‑‑‑‑‑‑‑‑‑‑‑‑‑‑‑‑‑‑‑‑‑‑‑‑‑‑‑‑‑‑‑‑‑‑‑‑‑‑‑‑‑‑‑‑‑‑‑‑‑‑‑‑‑‑‑‑‑‑‑‑‑‑‑‑‑‑‑‑‑‑‑‑‑‑‑‑‑‑‑‑‑‑‑‑‑‑‑‑‑‑‑‑‑‑‑‑‑‑‑‑‑‑‑‑‑‑‑‑‑‑‑‑‑‑‑‑‑‑‑‑‑‑‑‑‑‑‑‑‑‑‑‑‑‑‑‑‑‑‑‑‑‑‑‑‑‑‑‑‑‑‑‑‑‑‑‑‑‑‑‑‑‑‑‑‑‑‑‑‑‑‑‑‑‑‑‑‑‑‑‑‑‑‑‑‑‑‑‑‑‑‑‑‑‑‑‑‑‑‑‑‑‑‑‑‑‑‑‑‑‑‑‑‑‑‑‑‑‑‑‑‑‑‑‑‑‑‑‑‑‑‑‑‑‑‑‑‑‑‑‑‑‑‑‑‑‑‑‑‑‑‑‑‑‑‑‑‑‑‑‑‑‑‑‑‑‑‑‑‑‑‑‑‑‑‑‑‑‑‑‑‑‑‑‑‑‑‑‑‑‑‑‑‑‑‑‑‑‑‑‑‑‑‑‑‑‑‑‑‑‑‑‑‑‑‑‑‑‑‑‑‑‑‑‑‑‑‑‑‑‑‑‑‑‑‑‑‑‑‑‑‑‑‑‑‑‑‑‑‑‑‑‑‑‑‑‑‑‑‑‑‑‑‑‑‑‑‑‑‑‑‑‑‑‑‑‑‑‑‑‑‑‑‑‑‑‑‑‑‑‑‑‑‑‑‑‑‑‑‑‑‑‑‑‑‑‑‑‑‑‑‑‑‑‑‑‑‑‑‑‑‑‑‑‑‑‑‑‑‑‑‑‑‑‑‑‑‑‑‑‑‑‑‑‑‑‑‑‑‑‑‑‑‑‑‑‑‑‑‑‑‑‑‑‑‑‑‑‑‑‑‑‑‑‑‑‑‑‑‑‑‑‑‑‑‑‑‑‑‑‑‑‑‑‑‑‑‑‑‑‑‑‑‑‑‑‑‑‑‑‑‑‑‑‑‑‑‑‑‑‑‑‑‑‑‑‑‑‑‑‑‑‑‑‑‑‑‑‑‑‑‑‑‑‑‑‑‑‑‑‑‑‑‑‑‑‑‑‑‑‑‑‑‑‑‑‑‑‑‑‑‑‑‑‑‑‑‑‑‑‑‑‑‑‑‑‑‑‑‑‑‑‑‑‑‑‑‑‑‑‑‑‑‑‑‑‑‑‑‑‑‑‑‑‑‑‑‑‑‑‑‑‑‑‑‑‑‑‑‑‑‑‑‑‑‑‑‑‑‑‑‑‑‑‑‑‑‑‑‑‑‑‑‑‑‑‑‑‑‑‑‑‑‑‑‑‑‑‑‑‑‑‑‑‑‑‑‑‑‑‑‑‑‑‑‑‑‑‑‑‑‑‑‑‑‑‑‑‑‑‑‑‑‑‑‑‑‑‑‑‑‑‑‑‑‑‑‑‑‑‑‑‑‑‑‑‑‑‑‑‑‑‑‑‑‑‑‑‑‑‑‑‑‑‑‑‑‑‑‑‑‑‑‑‑‑‑‑‑‑‑‑‑‑‑‑‑‑‑‑‑‑‑‑‑‑‑‑‑‑‑‑‑‑‑‑‑‑‑‑‑‑‑‑‑‑‑‑‑‑‑‑‑‑‑‑‑‑‑‑‑‑‑‑‑‑‑‑‑‑‑‑

>Lal_ACC4_LAGI01_948

ATGGCTGGCATTGGCCGTGGGAAT‑‑‑‑‑‑‑‑‑GGGTACATAAATGGTGTACAGTCAAATAGACATCCCGCTACAATATCTGAAGTAGCTGAATTCTGCAGTGCCCTTGGTGGAAATAAGCCGATTCATAGCATATTAATTGCAAACAATGGAATGGCAGCAGTCAAGTTTATACGTAGTGTTAGGAGCTGGGCCTATGAGACATTTGGCACAGAGAAGGCTATCTTGTTGGTTGCCATGGCGACTCCAGAGGATATGAGGATCAATGCAGAACATATCAGAATAGCCGATCAATTTGTAGAAGTACCTGGTGGGACCAATAACAATAACTATGCCAATGTACAGCTTATTGTAGAG‑‑‑‑‑‑‑‑‑ATGGCTGAGATAACTCACGTTGATGCCGTGTGGCCTGGTTGGGGTCATGCATCAGAGAATCCCGAGCTTCCTGATGCATTGAAAGAAAGAGGAATTGTATTTCTTGGTCCTCCTGCTGTATCTATGGCAGCATTGGGAGATAAAATTGGTTCGTCGTTGATTGCTCAAGCAGCGGAAGTGCCAACCCTTCCATGGAGTGGTTCTCATGTGAAAATACCTCCTGAGAATTGCATGGTTACCATTCCTGATGAAATTTATCGGGAAGCATGTGTTTATACTACAGAAGAAGCAATTGCAAGTTGTCAAGTTGTAGGATACCCTGCAATGATCAAGGCATCTTGGGGTGGTGGTGGTAAAGGCATAAGAAAGGTTCATAATGATGATGAAGTAAGGGCATTGTTCAAGCAAGTTCAAGGTGAAGTCCCAGGCTCACCTATATTTATAATGAAGGTTGCATCCCAGAGCCGACATCTAGAAGTCCAGTTACTTTGTGATCAATATGGAAATGTTGCAGCTTTGCATAGCCGTGATTGCAGTATCCAAAGGAGGCATCAAAAGATCATTGAGGAGGGTCCCATTACTGTAGCTCCTCAGACGACGGTGAAACAACTAGAACAGGCAGCTAGAAGATTAGCGAAATCTGTAAATTATGTTGGGGCAGCTACTGTTGAGTATCTTTATAGCATGGAAACTGGTGCATACTACTTTTTAGAGTTGAACCCCCGACTACAGGTTGAGCATCCTGTTACTGAGTGGATAGCAGAGGTAAACCTGCCAGCAGCACAAGTTGCTGTTGGGATGGGTATCCCTCTCTGGCAAATTCCTGAGATAAGGCGTTTCTATGGCATGGAACATGGTGGGGGTAATGATGCTTGGAGGAAAACATCAGGTTTAGCTACTGCTTTTGATTTTGACAAGGCAGAGTCTACAAGGCCAAAAGGTCATTGTGTGGCTGTGCGAGTCACAAGTGAGGACCCTGATGATGGTTTTAAGCCTACAAGTGGGAAAGTGCAGGAGCTAAGCTTTAAAAGCAAGCCAAATGTGTGGGCATACTTCTCTGTTAAGTCTGGAGGAGGAATCCATGAATTTTCAGATTCTCAGTTTGGGCATGTTTTTGCTTTTGGAGAATCTAGGGGTTTAGCTATTGCAAATATGGTTTTGGGTCTAAAGGAGATTCAAATTCGAGGAGAAATTCGTACCAATGTTGATTACACCATTGATCTTCTGAATGCTTCAGACTACAGAGATAACAAGATTCACACAGGTTGGTTGGACAGTAGAATTGCAATGCGAGTTAGAGCAGAGAGGCCTCCCTGGTATCTTTCTGTTGTTGGAGGGGCACTCTACAAAGCTTCGGCTAGCAGTGCAGCTTTGGTTTCAGACTATGTTGGGTATCTTGAAAAGGGACAAATCCCGCCCAAGCACATATCCCTTGTCCATTCTCAAGTGTCCTTGAACATTGAAGGAAGCAAATACACGATTGACATGATACGAGGAGGATCTGGAAGTTATAGATTGAGAATGAATGAATCAGAGATAGAGGCAGAGATACATACTTTACGTGATGGAGGTTTGCTGATGCAG‑‑‑‑‑‑‑‑‑TTGGACGGAAATAGTCATATAATATATGCAGAGGAAGAAGCAGCTGGAACACGCCTTCTAATAGATGGAAGGACATGCTTGCTTCAGAATGATCATGATCCATCAAAGTTAATTGCGGAGACACCATGCAAGCTTATGAGATATTTGGTTGTGGATGACAGTCATGTTGATGCTGACACACCATATGCTGAAGTTGAGGTCATGAAGATGTGCATGCCTCTTCTTTCGCCTGCTTCTGGGGTTATTCATTTCAAACTGTCTGAAGGTCAAGCAATGCAGGCTGGTGAACTTATAGCAAGGCTTGATCTTGATGATCCTTCAGCTGTAAGAAAGGCAGAACTTTTCAACGGGATCTTCCCAGTCCTGGGCCTTCCTACTGCAATTTCTGGTAAAATTCATCAGAAATGTGCGGCAAGCTTAAATGCTGCACGCATGATTCTTGCTGGCTATGAGCACAATATTGATGATGTCGTACAAAGTTTGCTCCATTGCCTTGATAGTCCTGAATTGCCTTTCCTTCAATGGCAAGAGTGCTTCGCTGTTCTGGCAAACCGTCTTCCCAAAGACCTGAGAAGTGAGCTGGAATCAAAATATAAGGAGTTTGAGAGGATTTCA‑‑‑AGCACTCAAGTTGTTGAGTTCCCTACCAAATTCTTGAAGAGAATTCTTGAAGCTCATCTTTCCTCCTGCCCTGACAAAGAAAAAGGATCTCAAGAAAGACTAGTTGAACCTCTGCTGAGTCTTGTGAAGTCTTATGATGGTGGAAGAGAGAGCCATGCCCATATAATCGTTCAATCCCTTTTTGAAGAGTATCTTTTTGTTGAAGAACTATTCAGTGATAATATACTGGCTGATGTTATTGAACGCCTCCGCCTTCAATATAAGAAGGATCTATTGAATATTGTAGATATAGTGCTTTCTCATCAGGGTGTCAAGAGTAAAAATAAGCTGATACTGCAACTCATGGATAAACTGGTTTACCCTAACCCTGCTGCCTACAGAGATCAACTAATCCGTTTCTCTCAACTCAACCATACAAATTATTCTCAGTTGGCTCTTAAGGCAAGTCAACTTCTGGAACAAACTAAATTGAGTGAACTTCGATCCAGCATTGCCAGAAGTCTTTCTGAACTAGAGATGTTCACCGAGGATGGTGAAACTATTGATACTCCCAAGAGGAAGAGTGCTATTAATGACCGAATGGAGGACCTTGTAAGTGCTACTTTGGCAGTTGAAGATGCCCTCGTGGGTTTATTTGATCACAGCGATCACACCCTTCAAAGGAGGGTTGTGGAAACTTATATCCGCAGACTCTACCAGCCATATCTTGTCAAAGGGAGTGTCAGAATGCAGTGGAACAGATCTGGTCTTATTGCTTCATGGGAGTTCTTAGAGGAGTACATTGAAAGGAAGAGT‑‑‑GGGGTCGAAGAC‑‑‑CAAATGTCAGAAAAGACACCAGTGGGGAAACACGATAAGAAGAAATGGGGAGTGATGGTTATACTTAAATCTCTTCAATTTTTACCTGCAATGATAAGTGATGCCCTAAGGGAAGCAAGCAGTAACTTAAACGAAGCA‑‑‑CTTACA‑‑‑‑‑‑‑‑‑AGTGAACCAATTAAGTACGGTAATATGATGCATATTGCACTGGTGGGTATCAACAACCAGATGAGTTTACTGCAAGACAGTGGTGATGAGGATCAGGCTCAAGAAAGAATAAAAAAGTTAGCCAAAATTCTCAAAGATCAGGAAGTGGGTTCCACTATACGAGTTGCAGGTGTTGGAGTTATTAGCTGTATCATACAGAGGGATGAAGGGCGTGCACCGATGAGGCACTCCTTCCACTGGTCAGCAGAAAAGAACTATTATGAGGAGGAGCCTCTGTTGCGTCATTTGGAACCTCCCCTATCTATTTATCTGGAATTGGAAAAACTTAAAGCCTATGAGAATATACGGTATACACCATCTCGAGATCGTCAATGGCACCTGTACACAGTTATGGATAACAAACCACAACCAATTCAAAGAATGTTTCTTCGAACACTTCTAAGACAGCCAACCATAAATGAAGGGTTCTCTTTGTATCAAAGGCTGGATGCAGAAAAATCTCCTATCCAATTGGCTATGTCCTTCACTTCCAGGAGCATTTTTAGGTCCTTGATGGCTGCAATGGAGGAGTTGGAACTTAATGCACACAATGCCAATATCAAATCTGAACATGCTCATATGTACCTCTATATCATACGCGAGCAGCAAATAGATGATCTTGTGCCTTATACAAAGAAAATTGACGTAGATGCTGCCCAAGAAGAAATAACAGTTAAAACAATCTTAGAAGGACTGGCACATGAAGTCCATTCATCTGTTGGTGTAAGAATGCATAGATTAGGGGTTGTTGTATGGGAAATCAAGCTCTGGATGGCAGCCTGTGGTCAAGCAAATGGTGCTTGGAGGGTTATTGTAAACAACGTGACAGGTCATACATGCACTGTACATATATACCGAGAAATGGAGGATACCAACACTCATCAAGTGGTATACAGTTCAGTAGGTGTAAAGGGCCCATTGCATGGTGTACCAGTGAATGAAGATTATCAATCTTTGGGAGTTATTGACCGAAAACGGCTTTCAGCGAGAAAGAACAGCACCACATACTGCTACGATTTCCCCCTGGCATTTGAAACAGCCTTGGAACAGTCATGGGCAACCCAACAGTCTGGATCTCGAAGAGCCAAAGATACAGATCTTCTAAAAGCAACAGAGCTTAAATTTGCTGACAAAGAGGGTGGTTGGGGTACTCCCCTTGTTGCTGTGGAGCGTTCTCCTGGACTGAATGACGTTGGAATGGTAGCCTGGTTAATGGAAATGTGTACCTCCGAGTTTCCATCTGGAAGAACAATATTGGTTGTAACAAATGATGTAACCTTCAAGGCTGGTTCTTTTGGACCAAGAGAGGATGCATTCTTCCGTGCAGTAACTGATCTTGCATGTGCGAAAAAACTACCTTTAATTTATTTAGCAGCAAATTCTGGTGCTCGTTTAGGTGTAGCCGAGGAAGTGAAAGCCTGTTTCAGAGTTGGTTGGTCTGAAGAATCTAGCCCTGAGCACGGTTTCCAGTATGTATATTTAACACCTGTGGATTATGCTCGGATTGAATCATCAGTGATAGCACATGAATTAAAGCTTGAAAGTGGAGAAACCAGATGGGTCATAGATACCATTGTTGGGAAAGAGGATGGTCTGGGG‑‑‑‑‑‑‑‑‑‑‑‑‑‑‑‑‑‑‑‑‑‑‑‑‑‑‑‑‑‑‑‑‑‑‑‑‑‑‑‑‑‑‑‑‑‑‑‑‑‑‑‑‑‑‑‑‑‑‑‑‑‑‑‑‑‑‑‑‑‑‑‑‑‑‑‑‑‑‑‑‑‑‑‑‑‑‑‑‑‑‑‑‑‑‑‑‑‑‑‑‑‑‑‑‑‑‑‑‑‑‑‑‑‑‑‑‑‑‑‑‑‑‑‑‑‑‑‑‑‑‑‑‑‑‑‑‑‑‑‑‑‑‑‑‑‑‑‑‑‑‑‑‑‑‑‑‑‑‑‑‑‑‑‑‑‑‑‑‑‑‑‑‑‑‑‑‑‑‑‑‑‑‑‑‑‑‑‑‑‑‑‑‑‑‑‑‑‑‑‑‑‑‑‑‑‑‑‑‑‑‑‑‑‑‑‑‑‑‑‑‑‑‑‑‑‑‑‑‑‑‑‑‑‑‑‑‑‑‑‑‑‑‑‑‑‑‑‑‑‑‑‑‑‑‑‑‑‑‑‑‑‑‑‑‑‑‑‑‑‑‑‑‑‑‑‑‑‑‑‑‑‑‑‑‑‑‑‑‑‑‑‑‑‑‑‑‑‑‑‑‑‑‑‑‑‑‑‑‑‑‑‑‑‑‑‑‑‑‑‑‑‑‑‑‑‑‑‑‑‑‑‑‑‑‑‑‑‑‑‑‑‑‑‑‑‑‑‑‑‑‑‑‑‑‑‑‑‑‑‑‑‑‑‑‑‑‑‑‑‑‑‑‑‑‑‑‑‑‑‑‑‑‑‑‑‑‑‑‑‑‑‑‑‑‑‑‑‑‑‑‑‑‑‑‑‑‑‑‑‑‑‑‑‑‑‑‑‑‑‑‑‑‑‑‑‑‑‑‑‑‑‑‑‑‑‑‑‑‑‑‑‑‑‑‑‑‑‑‑‑‑‑‑‑‑‑‑‑‑‑‑‑‑‑‑‑‑‑‑‑‑‑‑‑‑‑‑‑‑‑‑‑‑‑‑‑‑‑‑‑‑‑‑‑‑‑‑‑‑‑‑‑‑‑‑‑‑‑‑‑‑‑‑‑‑‑‑‑‑‑‑‑‑‑‑‑‑‑‑‑‑‑‑‑‑‑‑‑‑‑‑‑‑‑‑‑‑‑‑‑‑‑‑‑‑‑‑‑‑‑‑‑‑‑‑‑‑‑‑‑‑‑‑‑‑‑‑‑‑‑‑‑‑‑‑‑‑‑‑‑‑‑‑‑‑‑‑‑‑‑‑‑‑‑‑‑‑‑‑‑‑‑‑‑‑‑‑‑‑‑‑‑‑‑‑‑‑‑‑‑‑‑‑‑‑‑‑‑‑‑‑‑‑‑‑‑‑‑‑‑‑‑‑‑‑‑‑‑‑‑‑‑‑‑‑‑‑‑‑‑‑‑‑‑‑‑‑‑‑‑‑‑‑‑‑‑‑‑‑‑‑‑‑‑‑‑‑‑‑‑‑‑‑‑‑‑‑‑‑‑‑‑‑‑‑‑‑‑‑‑‑‑‑‑‑‑‑‑‑‑‑‑‑‑‑‑‑‑‑‑‑‑‑‑‑‑‑‑‑‑‑‑‑‑‑‑‑‑‑‑‑‑‑‑‑‑‑‑‑‑‑‑‑‑‑‑‑‑‑‑‑‑‑‑‑‑‑‑‑‑‑‑‑‑‑‑‑‑‑‑‑‑‑‑‑‑‑‑‑‑‑‑‑‑‑‑‑‑‑‑‑‑‑‑‑‑‑‑‑‑‑‑‑‑‑‑‑‑‑‑‑‑‑‑‑‑‑‑‑‑‑‑‑‑‑‑‑‑‑‑‑‑‑‑‑‑‑‑‑‑‑‑‑‑‑‑‑‑‑‑‑‑‑‑‑‑‑‑‑‑‑‑‑‑‑‑‑‑‑‑‑‑‑‑‑‑‑‑‑‑‑‑‑‑‑‑‑‑‑‑‑‑‑‑‑‑‑‑‑‑‑‑‑‑‑‑‑‑‑‑‑‑‑‑‑‑‑‑‑‑‑‑‑‑‑‑‑‑‑‑‑‑‑‑‑‑‑‑‑‑‑‑‑‑‑‑‑‑‑‑‑‑‑‑‑‑‑‑‑‑‑‑‑‑‑‑‑‑‑‑‑‑‑‑‑‑‑‑‑‑‑‑‑‑‑‑‑‑‑‑‑‑‑‑‑‑‑‑‑‑‑‑‑‑‑‑‑‑‑‑‑‑‑‑‑‑‑‑‑‑‑‑‑‑‑‑‑‑‑‑‑‑‑‑‑‑‑‑‑‑‑‑‑‑‑‑‑‑‑‑‑‑‑‑‑‑‑‑‑‑‑‑‑‑‑‑‑‑‑‑‑‑‑‑‑‑‑‑‑‑‑‑‑‑‑‑‑‑‑‑‑‑‑‑‑‑‑‑‑‑‑‑‑‑‑‑‑‑‑‑‑‑‑‑‑‑‑‑‑‑‑‑‑‑‑‑‑‑‑‑‑‑‑‑‑‑‑‑‑‑‑‑‑‑‑‑‑‑‑‑‑‑‑‑‑‑‑‑‑‑‑‑‑‑‑‑‑‑‑‑‑‑‑‑‑‑‑‑‑‑‑‑‑‑‑‑‑‑‑‑‑‑‑‑‑‑‑‑‑‑‑‑‑‑‑‑‑‑‑‑‑‑‑‑‑‑‑‑‑‑‑‑‑‑‑‑‑‑‑‑‑‑‑‑‑‑‑‑‑‑‑‑‑‑‑‑‑‑‑‑‑‑‑‑‑‑‑‑‑‑‑‑‑‑‑‑‑‑‑‑‑‑‑‑‑‑‑‑‑‑‑‑‑‑‑‑‑‑‑‑‑‑‑‑‑‑‑‑‑‑‑‑‑‑‑‑‑‑‑‑‑‑‑‑‑‑‑‑‑‑‑‑‑‑‑‑‑‑‑‑‑‑‑‑‑‑‑‑‑‑‑‑‑‑‑‑‑‑‑‑‑‑‑‑‑‑‑‑‑‑‑‑‑‑‑‑‑‑‑‑‑‑‑‑‑‑‑‑‑‑‑‑‑‑‑‑‑‑‑‑‑‑‑‑‑‑‑‑‑‑‑‑‑‑‑‑‑‑‑‑‑‑‑‑‑‑‑‑‑‑‑‑‑‑‑‑‑‑‑‑‑‑‑‑‑‑‑‑‑‑‑‑‑‑‑‑‑‑‑‑‑‑‑‑‑‑‑‑‑‑‑‑‑‑‑‑‑‑‑‑‑‑‑‑‑‑‑‑‑‑‑‑‑‑‑‑‑‑‑‑‑‑‑‑‑‑‑‑‑‑‑‑‑‑‑‑‑‑‑‑‑‑‑‑‑‑‑‑‑‑‑‑‑‑‑‑‑‑‑‑‑‑‑‑‑‑‑‑‑‑‑‑‑‑‑‑‑‑‑‑‑‑‑‑‑‑‑‑‑‑‑‑‑‑‑‑‑‑‑‑‑‑‑‑‑‑‑‑‑‑‑‑‑‑‑‑‑‑‑‑‑‑‑‑‑‑‑‑‑‑‑‑‑‑‑‑‑‑‑‑‑

>Lan_ACC1_Lup012661

ATGGCTGGCATTGGCCGTGGGAAT‑‑‑‑‑‑‑‑‑GGGTACATAAATGGTGTACAGTCAAATAGACATCCCGCTACAGTATCTGAAGTAGCTGAATTCTGCAGTGCCCTCGGTGGAAATAAGCCGATTCATAGCATATTAATTGCAAACAATGGAATGGCAGCAGTCAAGTTTATACGTAGTGTTAGGAGCTGGGCCTATGAGACATTCGGCACAGAGAAGGCTATCTTGTTGGTTGCCATGGCTACTCCAGAGGATATGAGGATCAATGCAGAACATATCAGAATAGCCGATCAATTTGTAGAAGTACCTGGTGGGACCAATAACAATAACTATGCCAATGTACAGCTTATTGTAGAG‑‑‑‑‑‑‑‑‑ATGGCTGAGATAACTCACGTTGATGCCGTGTGGCCTGGTTGGGGTCATGCATCAGAGAATCCCGAGCTTCCTGATGCATTGAAAGAAAGAGGAATTGTATTTCTTGGTCCTCCTGCTGTATCTATGGCAGCATTGGGAGATAAAATTGGTTCGTCGTTGATTGCTCAAGCAGCGGAAGTGCCAACCCTTCCATGGAGTGGTTCTCATGTGAAAATACCTCCTGACAGTTGCTTGGTTACCATTCCTGATGAAATTTATCGGGAAGCATGTGTTTATACTACAGAAGAAGCAATTGCAAGTTGTCAAGTTGTAGGATACCCTGCTATGATCAAGGCATCTTGGGGTGGTGGTGGTAAAGGCATAAGAAAGGTTCATAATGATGATGAAGTAAGGGCATTGTTCAAGCAAGTTCAAGGTGAAGTCCCAGGCTCACCTATATTTATAATGAAGGTTGCATCCCAGAGCCGACATCTAGAAGTCCAGTTACTTTGTGATCAACATGGAAATGTTGCAGCTTTGCATAGCCGTGATTGCAGTATCCAAAGGAGGCATCAAAAGATCATTGAAGAGGGTCCCATTACTGTAGCTCCTCATGCAACGGTGAAACAACTAGAACAGGCAGCTAGAAGATTAGCGAAATCTGTAAATTATGTTGGTGCAGCTACTGTTGAGTATCTTTATAGCATGGAGACTGGCGCATACTACTTTTTAGAGTTGAACCCCCGACTACAGGTTGAGCATCCTGTTACTGAGTGGATAGCAGAGATAAACCTTCCGGCGGCACAAGTTGCTGTTGGGATGGGTATCCCTCTCTGGCAAATCCCTGAGATACGGCGTTTCTATGGTGTGGAACATGGTGGTGGTAATGATGCTTGGAGGAAAACATCAGGTTTAGCTACTGCTTTTGATTTTGACAAGGTAGAATCTACGAGGCCAAAAGGTCATTGTGTGGCTGTGCGAGTCACAAGTGAGGACCCCGATGATGGTTTTAAGCCTACAAGTGGGAAAGTGCAGGAGCTAAGCTTTAAAAGCAAGCCAAATGTGTGGGCATACTTCTCTGTTAAGTCTGGAGGAGGAATCCATGAATTCTCAGATTCTCAGTTTGGGCATGTTTTTGCTTTTGGAGAGTCTAGAGCTTTAGCTATTGCAAATATGGTTCTGGGGCTAAAGGAGATTCAAATTCGTGGAGAAATTCATACCAATGTTGATTACACCATTGATCTTCTGAATGCTGCAGACTACAGAGATAACAAGATTCACACAGGTTGGTTGGACAGTAGAATTGCAATGCGAGTTAGAGCAGAGAGGCCTCCCTGGTATCTTTCTGTTGTTGGAGGGGCACTCTATAAAGCTTCTGCTAGCAGTGCAGCATTGGTTTCAGACTATGTTGGGTATCTTGAAAAGGGACAAATCCCTCCCAAGCACATATCCCTTGTCCATTCTCAAGTGTCCTTGAACATTGAAGGAAGCAAATACACGATTGACATGATACGAGGAGGATCTGGAAGTTATAGATTGAGAATGAATGAATCAGAGATAGAGGCAGAGATACATACTTTACGTGATGGAGGTTTGCTGATGCAG‑‑‑‑‑‑‑‑‑TTGGATGGAAATAGTCATATAATATATGCCGAGGAAGAAGCAGCTGGAACTCGCCTTCTAATAGATGGAAGGACATGCTTGCTTCAGAATGACCATGATCCATCAAAGTTAATTGCGGAGACACCATGCAAGCTTATGAGATATTTGGTTGTGGATGACAGTCATGTTGATGCTGACACACCATATGCTGAAGTTGAGGTCATGAAGATGTGCATGCCTCTTCTTTCGCCTGCTTCCGGGATTATTCATTTCAAACTGTCTGAAGGTCAAGCAATGCAGGCTGGTGAACTAATAGCAAGGCTTGATCTTGATGATCCTTCAGCTGTAAGAAAGGCAGAACCTTTCAATGGGACCTTCCCAGTCTTGGGCCTTCCTACTGCAATTTCTGGTAAAGTTCATCAGAAGTGTGCGGCAAGCTTAAATGCGGCACGAATGATTCTTGCTGGCTATGAGCACAATATTGATGATGTTGTAAAAAGTTTGCTCCATTGCCTTGATAGTCCCGAATTGCCTTTCCTTCAATGGCAAGAGTGTTTCGCTGTTCTGGCAAACCGTCTTCCCAAAGACCTGAGAAATGAGCTGGAATCAAAATATAAGGAGTTCGAGAGGATTTCG‑‑‑AGCACTCAAGTTGTTGATTTCCCTGCTAAATTATTGAAGGGAATTCTTGAAGCTCATCTTTCCTCCTGCCCCGACAAAGAAAAAGGAGCTCAAGAAAGATTAGTTGAACCTCTGCTGAGTCTTGTGAAGTCTTATGATGGTGGAAGAGAAAGCCATGCCCATAAAATCGTTCAATCCCTTTTTGAAGAGTATCTTTTTGTTGAAGAACTATTTAGTGATAATATACTGGCTGATGTAATTGAGCGCCTCCGCCTTCAATATAAGAAGGATCTATTGAAGATTGTAGATATAGTGCTCTCTCATCAGGGTGTCAAGAGTAAAAATAAGCTGATACTGCAACTCATGGATAAACTGGTTTACCCTAACCCCGCTGCTTACAGGGATCAACTAATCCGTTTCTCTCAACTCAACCATACCAATTATTCTCAGTTGGCTCTTAAGGCAAGTCAACTGCTGGAACAAACTAAGTTGAGTGAACTTCGATCCAGCATTGCCAGAAGTCTTTCTGAACTAGAGATGTTCACTGAGGATGGTGAAACTATTGATACTCCCAAGAGGAAGAGCGCCATTAATGACCGAATGGAGGACCTTGTGAGTGCTACTTTGGCAGTTGAAGATGCCCTTGTGGGTTTATTTGATCACAGCGACCACACCCTTCAAAGGAGGGTTGTGGAAACTTATATACGCAGGCTCTACCAGCCATATCTTGTCAAAGGGAGTGTCCGGATGCAGTGGAACAGATCTGGTCTTATTGCTTCATGGGAGTTCTTAGAGGAGTACATTGAAAGGAAGAGT‑‑‑GGGGTCGAAGAC‑‑‑CAAATATCAGAACAAACACTGGTGGGGAAACACAATAAGAAGAAATGGGGAGTGATGGTTGTACTTAAATCTCTTCAATTTTTACCTGCAATGATAAGTGATGCCCTAAGGGAAGCAAGCGGTAACCTACACGAAGCA‑‑‑CTTCCT‑‑‑‑‑‑‑‑‑AGTGAACCAGTTAAGTATGGTAATATGATGCATATTGCACTGGTCGGTATCAACAACCAGATGAGTTTACTGCAAGACAGTGGTGATGAGGATCAGGCTCAAGAAAGAATAAAAAAATTAGCCAAAATACTCAAAGATCAGGAAGTGGGCCCCACTATACGGGCAGCGGGTGTTGGAGTAATTAGCTGTATCATACAGAGGGATGAAGGGCGTGCACCAATGAGGCACTCCTTCCACTGGCCAGCAGAAAAGAACTATTATGAGGAGGAGCCTCTGTTGCGTCATTTGGAACCTCCCCTATCCATTTATCTGGAATTGGAAAAACTTAAAGCCTATGAGAATATACGGTATACCCCATCTCGAGATCGTCAATGGCACCTGTACACAGTTACGGATAACAAACCACAACCAATTCAAAGAATGTTTCTTCGAACACTTCTAAGACAGCCAACCACAAATGAAGGGTTCTCTTTGTATCAAAGGCTGGATGCAGAAAAATCTCTTACCCAATTGGCTATGTCCTTCACTTCCAGGAGCATTTTTAGGTCATTGATGGCTGCAATGGAGGAGTTGGAACTTAATGCACACAATGCCAATATCAAATCTGAACATGCTCATATGTACCTCTATATCATACGCGAGCAGCAAATAGATGATCTTGTGCCTTATACAAAGAGAATCGACATAGATGCTGCCCGAGAAGAAGTAACAGTTGCGGAAATCTTAGAAGGACTGGCACATGAAGTCCATTCATCTGTTGGTGTAAGAATGCATAGATTAGGGGTTGTTGTATGGGAAATCAAGCTCTGGATGGCAGCCTGTGGTCAGGCAAATGGTGCTTGGAGGGTTATTGTAAACAACGTGACAGGTCATACATGCACTGTACATATATACCGAGAAATGGAGGATACCGACACTCATAAAGTGGTATACAGTTCAGTAGGTGTGAAGGGTCCGTTGCATGGTGTACCAGTGAATGAAAATTATCAATCTTTAGAAGTTATTGACCGAAAACGCCTTTCAGCGAGAAAGAACAGCACCACATACTGCTACGATTTCCCCCTGGCATTTGAAACAGCCTTGGAACAGTCATGGGCAACCCAACAGTCTGGATCTCAAAGAGCCAAAGATACAAATCTTCTAAAAGCAACAGAGCTTAAATTTGCTGACAAAGAAGGTAGTTGGGGTACTCCTCTTGTTGCTGTGGAGCGTTCTCCTGGACTGAATGACGTTGGAATGGTAGCCTGGTTAATGGAAATGTGTACCCCCGAGTTTCCATCTGGAAGAACAATATTGGTTGTAACAAATGATGTAACCTTCAAGGCTGGTTCTTTTGGCCCAAGAGAGGATGCATTCTTCCGTGCAGTAACTGATCTTGCATGTGCGAAAAAACTACCTTTAATTTATTTAGCAGCAAATTCTGGTGCCCGTTTAGGTGTAGCCGAGGAAGTGAAAGCCTGTTTTAGAGTTGGTTGGTCTGAGGAATGTAGCCCTGAGCATGGGTTCCAGTACGTATATTTAACACCTGAGGATTATGCTCGGATTGAATCATCAGTGATAGCACATGAATTAAAGCTTGAAAGTGGAGAAACCAGATGGGTCATAGACACCATTGTTGGGAAAGAGGATGGCCTGGGGGTCGAAAACTTGAGTGGTAGTGGTGCCATTGCTGGTGCCTATTCAAGGGCATACAAGGAAACTTTTACATTGACATATGTGACTGGCAGGACTGTTGGAATAGGGGCTTATCTTGCCAGGCTTGGAATGAGATGCATACAGAGGCTTGATCAGCCCATAATTCTTACTGGTTTTTCAGCGTTAAATAAACTTCTTGGTCGGGAGGTATACAGCTCTCACATGCAACTTGGTGGACCTAAAATCATGGCAACTAATGGAGTTGTTCACCTTACAGTTTCGGATGACCTTGAAGGTGTTTCTGCTATTTTGAAGTGGCTTAGCTACGTTCCTTCACATGTAGGTGGTAAGCTCCCCATTGTAAAGTCCCTTGATTCTCCAGAAAGACGAGTGGAGTACTTGCCTGAAAACTCATGTGATCCTCGTGCTGCCATCGCTGGAACTACTGATGGTAACGGAAGATGGCTAGGAGGCATTTTTGACAAGGACAGCTTTGTGGAGACATTAGAAGGATGGGCAAGGACAGTTGTTACAGGAAGGGCAAAGCTCGGAGGAATTCCTGTCGGAATTGTTGCTGTAGAAACACAAACAGTAATGCAAATAATACCTGCCGATCCAGGCCAGCTTGATTCTCATGAGAGGGTTGTTCCTCAAGCAGGACAGGTGTGGTTTCCTGATTCTGCTGCCAAGACAGCCCAATCAATATTGGATTTCAACAGAGAAGAGCTCCCACTTTTCATTCTTGCAAATTGGAGAGGCTTTTCAGGTGGGCAGAGGGACCTTTTTGAAGGAATTCTTCAGGCTGGATCAACCATTGTAGAGAACCTTAGGACATACAAACAGCCCATATTTGTGTACATCCCAATGATGGGTGAACTCCGTGGTGGGGCGTGGGTTGTTGTTGACAGTCGAATCAATTCAGACCACATTGAAATGTATGCCGATCGAACAGCTAAAGGCAATGTCCTGGAGCCAGAAGGAATGATTGAGATCAAATTCAGAACAAGAGAATTGTTAGAGTGCATGGGCAGATTGGATCAACAGTTGATAACTCTGAAGGCAAAACTTCAGGAGGCCAAGAGTAACAGAGAAATTGTGACCATTGAATCCTTACAGCAGCAGATTAAGACCCGCGAGAAACAGCTTTTGCCCATGTATACCCAGATAGCTACCAAATTTGCTGAACTGCATGATACTTCCTTAAGAATGGCTGCAAAGGGGGTGGTAAGAGAAGTTTTGGACTGGGGTAACTCCCGTGCTGTCTTCTACCGAAGATTGAACAGAAGAATTGGTGAGCAGACACTGATCAACAGTTTTAGAGATGCTGCAGGTGGTGACCATTTGTCACATGTATCCGCGTTGGAATTGCTCAAGAACGTGTATTTGAATTCTGATATCGCCAAAGGTAGAGAAGATGCCTGGTCGGATGATGAAGCCTTCTTCAGATGGAAGGATAATCCTACAAACTATGAGGATAAACTAAAGGAATTGCGTGCTCAGAAAGTGTTGCTTCAACTGACAAATATCGGTGACTCAGTTCTAGATATGCAAGCTTTACCTCAAGGCCTTGCTGCCCTTTTAAGCAAGTTGGAGCCATCAAGTCGTGTGAAGTTGACGGATGAACTTCGCAAGGTACTTGGT‑‑‑

>Lan_ACC2_Lup009248

ATGGCTGGCATTGGCCGTGGGAAT‑‑‑‑‑‑‑‑‑GGGTACATAAATGGTATACAACCAAATAGGCACCCGGCTACAATATCCGAAGTAGACGAGTTCTGCAGCGCCCTTGGGGGAAATAAACCAATCCATAGCATATTGATTGCAAACAATGGAATGGCAGCAGTCAAGTTTATACGTAGTGTTAGGAGCTGGGCCTATGAGACCTTTGGCACAGAGAAGGCTATCCTGTTGGTTGCCATGGCTACACCAGAGGACATGAGGATCAATGCAGAACATATTAGAATAGCTGATCAATTTGTCGAAGTACCTGGTGGGACCAATAACAATAACTATGCCAATGTACAGCTTATTGTAGAG‑‑‑‑‑‑‑‑‑ATGGCTGAGATAACTCATGTTGATGCTGTGTGGCCTGGTTGGGGTCATGCATCAGAGAATCCTGAGCTTCCTGATGCATTGAAAGAAAAAGGAATCGAATTTCTTGGTCCTCCTGCTGTATCTATGGCAGCATTGGGAGATAAAATTGGTTCGTCGTTGATTGCTCAAGCAGCGGAAGTGCCAACCCTTCCATGGAGTGGTTCTCATGTGAAAATACCTCCTGAAAGTTGCTTGGTTACCATCCCCGATGAAATTTATCGGGAAGCATGTGTTTATACTACAGAAGAAGCAATTGCGAGTTGTCAAGTTGTAGGATACCCTGCAATGATCAAGGCATCTTGGGGTGGTGGTGGTAAAGGCATAAGAAAGGTTCATAATGATGATGAAATAAGGGCATTGTTCAAGCAAGTTCAAGGTGAAGTTCCAGGCTCACCTATATTTATAATGAAGGTTGCATCCCAGAGCCGGCATCTAGAAGTCCAGTTACTTTGTGACCAATATGGAAATGTTGCAGCTTTGCATAGCCGTGATTGCAGTATTCAAAGGAGGCATCAAAAGATTATCGAGGAGGGCCCCATTACTGTAGCTCCTCCGGCAACGGTGAAACAACTAGAACAGGCAGCTCGAAGATTAGCAAAATCTGTAAATTATGTTGGTGCAGCTACTGTTGAGTATCTTTATAGCATGGAAACTGGCGAGTACTACTTTTTAGAGTTGAACCCTCGACTACAGGTCGAGCACCCGGTTACAGAGTGGATAGCAGAGATAAATCTGCCAGCAGCACAAGTTGCCGTTGGGATGGGTATCCCTCTCTGGCAAATTCCAGAGGTAAGGCGTTTCTATGGGGTGGAACATGGTGGGGGTAATGATGCTTGGAGGAAAACATCAGGTTTAGCTACTCCCTTTGATTTTGACAAGGTAGAATCTACAAGGCCAAAAGGTCATTGCGTGGCTGTGCGAGTCACAAGTGAGGACCCCGATGATGGTTTTAAGCCTACAAGCGGGAAAGTGCAGGAGCTAAGCTTTAAAAGCAAGCCAAATGCGTGGGCATACTTCTCTGTTAAGTCTGGAGGAGGAATCCATGAATTCTCAGATTCTCAGTTTGGGCATGTTTTTGCTTTTGGAGAATCTAGGGGTTTAGCTATTGCAAATATGGTTCTGGGTCTAAAGGAGATTCAAATTCGAGGAGAAATTCGTACCAATGTTGATTACACCATTGATCTTCTGAATGCTTCAGACTACAGAGATAACAAGATTCACACGGGGTGGCTGGACAGTAGAATTGCAATGCGGGTTAGAGCAGAGAGGCCTCCCTGGTATCTTTCTGTTGTTGGAGGGGCACTCTATAAAGCTTCTGCTTGCAGTGCAGCTTTGGTTTCTGACTATGTTGGTTATCTTGAAAAGGGACAAATCCCTCCCAAGCACTTGTCTCTTGTCCATGCTCAAGTGTCCCTGAACATAGAAGGAAGCAAATACACGATTGACATGATACGAGGAGGGTCTGGAAGTTATAGATTGAGAATGAATGAATCAGAGATAGAAGCAGAGATACATACTTTACGTGATGGAGGTTTGCTGATGCAG‑‑‑‑‑‑‑‑‑CTGAATGGAAACAGTCATATAATATATGCAGAGGAAGAAGCAGCTGGGACCCGCCTTCTAATAGATGGAAGGACTTGCTTGCTTCAGAATGATCATGATCCATCAAAGTTAATTGCGGAGACTCCTTGCAAGCTTATGAGATATTTGGTTGCGGATGACAGTCATGTTGATGCTGACACACCATATGCTGAAGTTGAGGTCATGAAGATGTGCATGCCTCTTCTTTCGCCTGCTTCTGGAATTATTCATTTCAAAATGTCTGAAGGTCAAGCTATGCAGGCTGGTGAACTTATAGCAAGGCTTGATCTTGATGATCCTTCAGCTGTAAGAAAGGCAGAACCTTTCAATGGGAGCTTCCCTGTTCTGGGCCCTCCTACTGCAATTTCTGGTAGAGTTCATCAGAAATGTGCTGCAAGCTTAAATTCTGCACGCATGATTCTTGCTGGCTACGAGCACAATATTGATGATGTTGTACAAAGTTTGCTCAATTGCCTCGATAGTCCTGAATTGCCTTTTCTTCAATGGCAAGAGTGCTTCGCAGTTCTGGCAAATCGTCTTCCCAAAGATCTGAGAAGCGAGTTGGAATCAAAATATAAGGAGTTCGAGAGGATTTCA‑‑‑AGCACACAAATTGTTGATTTCCCTGCCAAATTATTGAAGGGAATTCTTGAAGCTCATCTTTCCTCTTGTCCCGACAAAGAAAAAGGAGCTCAAGAAAGATTAGTCGAACCTTTGCTGAGTCTTGTGAAGTCTTATGATGGTGGAAGAGAGAGCCATGCCCATATAATTGTTCAGTCCCTTTTTGAAGAGTATCTTTTTGTTGAAGAAATATTTAGTGATAATATACTGGCTGATGTAATTGAACGCCTCCGGCTTCAATATAAGAAAGATTTATTGAAGATTGTAGACATAGTGCTCTCTCATCAGGGTGTGAAGAGTAAAAATAAGCTGATACTGCAGCTCATGGATAAACTGGTTTACCCTAACCCTGCTGCCTACAGGGATCAACTAATCCGTTTCTCTCAACTCAACCATACAAATTATTCTCAGTTGGCTCTTAAGGCAAGTCAACTTCTGGAACAAACTAAATTGAGTGAACTGCGGTCCAGCATTGCTAGAAGTCTTTCTGAACTAGAGATGTTCACCGAGGATGGTGAAACTATCGATACTCCCAAGAGGAAGAGTGCCATTAATGACCGAATGGAGGACCTTGTGAGTGCTCCTTTGGCAGTTGAAGATGCCCTTGTGGGTTTATTTGATCACAGCGATCACACCCTTCAAAGGAGGGTTGTGGAAACTTATGTACGCAGGCTCTACCAGCCATATCTTGTCAAAGGGAGTGTCAGGATGCAGTGGAACAGATCTGGTCTTATTGCTTCATGGGAGTTCTTAGAGGAGTACATTGAAGGGAACAGT‑‑‑GGGGTCGAAAAC‑‑‑CAAATATTAGAAAAAACACTGGTAGAGAAACACAGTGGGAAGAAATGGGGAGTGATGGTTATAATTAAATCTCTTCAATTTTTACCTGCTATGATAAGTGATGCGCTAAGAGAAGCAAGCAGTAACCTACACGAACCAAATATTACAAATGGTTCTGGTGAAGCAGTTAAGTATGGTAATATGATGCATATTGCACTAGTGGGTATCAACAACCAGATGAGTTTGCTGCAAGACAGTGGTGATGAGGATCAGGCTCAAGAAAGAATAAAAAAGTTAGCCAAATTTCTCAAAGATGAAGAAGTGGGTTCCACCATACGAGCTGCAGGTGTTGGAGTTATTAGCTGTATCATACAGAGGGATGAAGGGCGTGCACCCGTGAGGCACTCCTTTCACTGGTCAGCAGAAAAACACTATTATGAGGAGGAGCCTCTGTTGCGGCATTTGGAACCTCCACTGTCCATTTATCTTGAATTGGACAAACTTAAAGCCTATGAAAATATACGGTATACCCCATCTCGAGATCGTCAATGGCACCTGTACACAGTTGTGGATAACAAACCACAACCAATTCAAAGAATGTTTCTTCGTACACTTCTAAGACAACCAACCACAAATGAAGGGTTCTCTTTGTATCAAAGGCTGGATGCAGAAACATCTCGTACCCAATTGGCTATGTCCTTCACTTCCAGGAGCATTTTTAGGTCCTTGATGGCTGCGATGGAGGAGTTGGAACTTAATGCACACAATGCCAATACCAAATCTGAACATGCTCATATGTACCTATATATCATACGTCAGCAACAAATAGATGATCTTGTGCCTTATCCAAAGAGAATTGACTTAGTTGCTGGCCAAGAAGAAACAACAGTCGAGGCAATCTTAGAAGGACTGGCACATGAAGTCCATTCTTCTGTTGGTGTAAGGATGCATAGATTGGGAGTTGTTGTGTGGGAAATCAAGCTCTGGATGGCAGCCTTTGGTCAGGCAAATGGTGCTTGGAGGGTTATTGTAAATAATGTGACGGGTCATACTTGCACTGTACATATATACCGAGAATTGGAGGATACCAGCACTCATAAAGTGGTATACAGTTCAGTAGGTGTAAAGGGCCCGCTGCATGGTGTACCAGTAAATGAAAATTATCAATCTTTGGGAGTTATTGACCGAAAACGTCTTTCAGCAAGAAAGAACAGCACCACATACTGCTACGATTTCCCCCTGGCATTTGAAACAGCCTTGGAACAGTCATGGGCAATCCAGCAGTCTGCATTTCAAAGAGCCAAAGATACAGATCTTCTAAAAGCAACAGAGCTTAAATTTGCTGACAAAGAAGGTGGTTGGGGTACACCTCTTATTACTGTGGAGCGTTCTCCTGGACTGAATGATGTTGGCATGGTAGCCTGGTTAATGGAAATGCGTACCTCCGAGTTTCCATCTGGAAGGACAATATTTGTTGTAGCAAATGATGTAACCTTCAAGGCTGGTTCTTTTGGCCCAAGAGAAGATGCATTCTTCCGTGCAGTAACTGATCTTGCATGTGCGAAAAAACTACCTTTAATTTATTTAGCAGCAAACTCTGGTGCCCGTTTAGGTGTAGCCGAGGAAGTCAAATCCTGTTTCAAAGTTGGTTGGTCTGAGGAATCTAGACCTGAGCATGGTTTTCAGTATGTATATTTAACACCTGAGGATTACGCTCGGATTGAATCATCAGTGATAGCACATGAATTAAAGCTTGAAAGTGGAGAAACCAGATGGGTCATAGACACCATTGTTGGGAAAGAGGATGGCCTGGGGGTCGAAAACTTGAGTGGTAGTGGTGCCATTGCTGGTGCCTATTCAAGGGCATACAAGGAAACTTTTACATTGACATATGTGACTGGCAGGACTGTCGGAATAGGGGCTTATCTTGCCAGGCTCGGGATGAGATGCATACAGAGGCTTGATCAGCCCATAATTCTTACCGGGTTTTCAGCGTTAAATAAACTTCTTGGTCGTGAGGTATACAGCTCCCATATGCAACTTGGTGGACCTAAGATCATGGCAACTAATGGAGTTGTTCACCTTACAGTTTCGGATGACCTTGAAGGTGTTTCCGCTATTTTAAAGTGGCTTAGCTACGTTCCTTCTCATGTAGGTGGTAAGCTTCCCATTGTAAAGTCCCTTGATCCTCCAGAAAGACAAGTGGAGTACTTGCCAGAAAACTCATGTGATCCTCGTGCTGCCATCGCCGGAACTGTGGATGGTAATGGAAGATGGCTAGGAGGCATTTTTGACAAGGACAGTTTTGTGGAGACATTAGAAGGATGGGCAAGGACAGTTGTTACAGGAAGGGCAAAACTTGGAGGAATTCCTGTGGGAATCGTTGCTGTAGAAACACAAACGGTAATGCAAATAATACCTGCTGATCCAGGCCAGCTTGATTCTCATGAGAGGGTTGTTCCTCAAGCTGGACAAGTGTGGTTTCCTGATTCTGCTACCAAGACAGCCCAAGCAATATTGGATTTCAACAGAGAAGAGCTCCCACTTTTCATTCTTGCAAATTGGAGAGGCTTTTCAGGTGGGCAGAGGGATCTTTTTGAAGGAATTCTTCAGGCTGGTTCAACCATTGTAGAGAACCTTAGGACATACAAGCAACCCATATTTGTATACATCCCAATGATGGGCGAACTCCGTGGTGGGGCGTGGGTTGTTGTTGACAGTCAAATCAATTCAGACCACATTGAAATGTATGCCGACCGAACAGCTAAAGGCAATGTCCTGGAGCCAGAAGGGATGATTGAGATCAAATTCAGAACAAGAGAATTGTTGGAGTGCATGGGTAGACTGGATCAACAGCTGATAACTCTGAAGGCAAAACTTCAGGAGGCCAAGAGTAACAGGGACATTGTGAACATCGAATCCCTACAACAGCAAATTAAGTCACGCGAGAAACAGCTTTTGCCTGTGTATACCCAGATAGCTACCAAATTCGCTGAACTGCATGATACTTCCTTCAGAATGGCTGCAAAGGGGGTAGTAAGGGAAGTTCTGGACTGGGGTAACTCCCGTGCTGTCTTCTACCGCAGACTTAACAGGAGAATTGGTGAGCAGACATTGATCAACAGTGTCAGAGATGCTGCCGGTGGTGACCATTTGTCACATGTATCCGCATTGGAATTGCTCAAGAACTTGTATCTGAGTTCTGATATTGCTAAAGATAGCGAAAATGCATGGTTGGATGATGAAGCCTTCTTCAGATGGAAGGATAATCCTGCAAACTATGAGGATAAACTAAAGGAATTGCATGCCCAGAAAGTGTTGCTTCAACTAACAAATATTGGTGACTCGGTTCTAGATTTGCAAGCTCTACCCCAATGCCTTGCTGCCCTTTTAAGCAAGTTGGAGCCATCAAGCCGGGTGAAGTTAACCGATGAACTTCGTAAAGTGCTTGGT‑‑‑

>Lja_ACC_Lj0g3v0268829

‑‑‑‑‑‑‑‑‑ATGGGGGGTGGGAAT‑‑‑‑‑‑‑‑‑GGATATATAAATGGTGTACTCCCCAACAAGCACCTTGCTACAATATCTGAAGTAACTGAATTCTGCAACGCCCTTGGGGGGAACAGGCCAATTCATAGCATATTGATTGCAAACAATGGAATGGCAGCGGTCAAGTTCATGCGCAGTGTTAGGAGCTGGGCCTATGAGGCATTTGGCACGGAGAAGGCTATCTTGTTGGTTGCCATGGCTACACCAGAGGATATGAGAATCAATGCAGAACATATCAGAATAGCTGATCAATTTGTGGAAGTACCTGGTGGGACCAATAACAATAACTATGCCAATGTGCAGCTTATTGTCGAGGGTTGTGATATGGCTGAGATAACTCATGTTGATGCGGTGTGGCCTGGTTGGGGTCATGCATCCGAGAATCCTGAGCTTCCTGATGCACTAAAAGAAAAAGGAATTGTATTTCTTGGACCTCCTGCTGTATCTATGGCAGCATTGGGAGATAAAATTGGTTCGTCTTTGATAGCTCAAGCAGCTTCTGTGCCAACCCTTCCATGGAGTGGTTCTCATGTGAAAATTCCTCCTGACAGTTCATTGATTACTATTCCTGATGAAATTTATCGGGAAGCGTGTGTTTATACCACGGAAGAAGCAATTGCCAGTTGTCAAGTTGTAGGATACCCTGCAATGATCAAAGCATCTTGGGGTGGTGGTGGTAAAGGAATAAGAAAGGTTCATAATGATGATGAGGTAAGGGCATTGTTCAAGCAAGTTCAAGGTGAAGTTCCAGGGTCACCTATATTTATAATGAAGGTCGCATCCCAGAGCCGACATCTAGAAGTCCAGTTAGTTTGTGACCAGTATGGAAATGTTGCAGCTTTACATAGTCGTGATTGCAGTGTTCAAAGGAGGCATCAAAAGATTATCGAGGAGGGTCCTATTACTGTAGCACCTCAGGCAACGGTGAAACAACTAGAGCAGGCAGCTAGAAGATTGGCTAAATCTGTAAATTATGTTGGGGCAGCTACTGTTGAGTATCTATATAGTATGGAAACTGGCGAGTACTACTTTTTAGAGTTGAACCCCCGACTACAGGTTGAGCATCCTGTTACTGAATGGATAGCAGAGATAAATCTGCCAGCAGCACAAGTTGCCATTGGGATGGGAATCCCTCTCTGGCAAATTCCTGAGATAAGGCGTTTCTATGGGGTGGAACACGGTGGAGGGAATGATGCTTGGAGGAAAACATCAGTTTCAGCTACTCCTTTTGATTTTGACAAAGCAGAATCTACTAGGCCAAAAGGTCATTGTGTGGCTGTTCGAGTCACTAGTGAGGACCCTGATGATGGTTTTAAGCCTACTGGTGGGAAAGTGCAGGAGCTCGGCTTTAAAAGTAAGCCAAATGTGTGGGCATACTTCTCTGTTAAGTCTGGAGGAGGGATCCATGAATTCTCAGATTCTCAGTTTGGGCATGTTTTTGCTTTTGGAGAATCAAGGGCTTTAGCAATTGCAAATATGGTTTTGGGGCTGAAGGAGATTCAAATTCGGGGAGAAATTCGTACAAATGTTGATTACACCATTGATCTTCTGAATGTTGCAGACTACAGAGATAACAAAATCCACACAGGATGGCTGGACAGTAGAATTGCTATGCGGGTTAGAGCAGAGAGGCCTCCCTGGTATCTTTCTGTTGTTGGAGGGGCACTCTATAAAGCTTCTGCTAGCAGTGCAGCTTTGGTTTCTGACTATATTGGCTATCTTGAAAAGGGGCAAATCCCTCCCAAGCATATATCTCTTGTCCATTCTCAAGTGTCTTTGAACATTGAAGGAAGCAAATATACGATTGACGTGATGCGAGGAGGACCTGGAAGTTATAAATTGAGATTGAATGAATCAGAAATAGAAGCTGAGATACATACTTTACGTGATGGAGGTTTGCTGATGCAG‑‑‑‑‑‑‑‑‑TTGGATGGTAACAGTCATGTAATATACGTAGAGGAAGAAGCAGCTGGAACTCGCCTTCTAATTGATGGAAGGACTTGCTTGCTTCAGAATGATCATGATCCGTCAAAGTTAATTGCAGAGACACCATGCAAGCTTCTGAGGTATTTGGTTGTGGATGACAGTCATGTTGATGCTGACACACCATATGCTGAAGTTGAGGTTATGAAGATGTGCATGCCTCTTCTTTCACCTGCTTCTGGGAAAATTCATTTCAAAATGTCTGAAGGTCAAGCAATGCAGGCTGGTGAACTTATAGCAAGGCTTGATCTAGATGATCCTTCAGCTGTAAGAAAGGCGGAACCCTTCACTGGGAACTTCCCAGTCTTGGGTTTTCCTACTGCAATTTCAGGTAAAGTTCATCAGAAATGTGCCGCAAGCTTAAATGCTGCACGGATGATTCTTGCTGGCTATGAGCACAATATTGATGAGGTTGTGCAAAGTTTGCTCAATTGCCTTGACAGTCCTGAATTACCTTTCCTTCAATGGCAAGAGTGCTTCGCAGTTCTGGCAACTCGTCTTCCTAAAGAACTAAAAAATGAGTTGGAATCAAAATATAAGGAGTTCGAGAGGATTTCA‑‑‑AGCTCCCAAATTGTTGATTTCCCTGCCAAATTATTGAAGGGAATTCTTGAAGCTCACCTTTCCTCCTGTCCCGAAAACGAAAAAGGAGCCCAAGAAAGACTAGTTGAACCTCTGTTGAGTCTTGTGAAGTCTTATGAGGGTGGAAGAGAGAGTCATGCCCATATAATTGTTCAATCCCTTTTTGAAGAGTATCTTTTTGTTGAAGAACTATTTAGTGATAACATACAGGCTGATGTAATTGAACGTCTCCGTCTTCAGTACAAGAAAGATTTGTTAAAGATTGTAGATATAGTGCTCTCTCACCAGGGTATCAAGAGTAAAAATAAGCTCATACTGCGACTGATGGATAAACTGGTTTACCCTAACCCTGCTGCCTATAGGGATCAATTGATCCGTTTCTCTGCTCTCAACCATACAAACTATTCTCAGTTGGCTCTTAAGGCAAGTCAACTGCTGGAACAAACTAAGTTGAGTGAACTACGATCCAGTATTGCTAGAAGTCTTTCTGAACTTGAGATGTTCACTGAGGATGGTGAAACTATTGATACTCCAAGGAGGAAGAGTGCCATTAATGACCGAATGGAGGACCTTGTCAGTGCTCCTTTGGCAGTTGAAGATGCCCTTGTGGGTTTATTTGATCACAGTGATCACACCCTTCAAAGGAGGGTTGTGGAAACTTATATACGAAGGCTCTACCAGCCATATCTCGTCAAAGGGAGTGTAAGGATGCAGTGGCACAGATCTGGTCTTATTGCTACATGGGAGTTCTTTGAAGGGAATATTGAACGGAAGAAT‑‑‑GGGGTTGAAGAC‑‑‑CAG‑‑‑ACGGATAAAGCACTGGTGGAGGGACATAGTGAGAAAAAATGGGGAGTGATGGTTATTATTAAATCTCTACAGTTTTTGCCTGCAATTATCAGTGCTGCATTAAGGGAAGCAACTGGTAACCTTCCCAAAGAA‑‑‑CTTACAAGTGGTTCTGGTGACACCAATATCTATGGTAATATGATGCATATTGGATTAGCGGGCATCAATAACCAAATGAGTTTACTGCAAGACAGTGGTGACGAGGATCAGGCTCAAGAAAGAATCAATAAGTTAGCCAAAATACTCAAAGATCAGGAAGTAGGCTCCACAATACGCGCTGCAGGTGTTGGAGTAATCAGCTGTATCATACAGAGGGATGAAGGGCGTGCCCCAATGAGACACTCCTTTCACTGGTCATCTGAAAAGCTCTACTATGCTGAGGAACCTCTGTTGCGTCACCTTGAACCTCCCCTATCCATTTATCTTGAATTGGACAAACTTAAACACTATGAGAATATACGGTACACCCCATCTCGAGATCGTCAATGGCATCTATACACGGTTGTAGATAAAAAGCCACAACCAATTCAAAGAATGTTTCTCCGAACACTTCTAAGACAGCCAACCACAAATGAAGGATTCTCTTCTTATCAAAGGCTGAATGCAGAAACATCTCGTACCCAATTAAGTATGTCCTATACTTCAAGGAGCATCTTTAGGTCCTTGATGACTGCAATGGAGGAGCTGGAACTTAATGCACATAATGCCGCCATCAAACCTGAACATGCTCATATGTATCTCTATATCATACGCGAGCAACACATAGAAGACCTTGTGCCTTATCCCAAGAGAATTAACATCGATGCTGGTCGAGAAGAAGCAATAGTTGAGGCAATCTTGGAAGAACTGGCTCACGAAATCCATTCATCTGTTGGAGTAAGAATGCATAGATTAGGGGTGGTTGTGTGGGAAGTCAAGCTCTGGATGGCTGCTTGTGGACAGGCAAATGGTAATTGGAGGGTCATTGTAAACAATGTGACTGGTCATACATGCACTGTACATATATATAGAGAAGTAGAGGATGCCACCACTCATAAAGTGGTATACAGTTCAATCAACGTAAAGGGTCCTCTGCATGGTGTGCCAGTGAATGAGAACTATCAATCTTTGGGAGTTCTTGACCGGAAACGTCTTTCAGCAAGAAAGAATAGCACCACATACTGCTACGATTTTCCCCTGGCATTTAAAAGAGCCTTGGAGCATTCATGGGAAATCCAACAGCCAGGAATTGAAAGAGCCAAAGAT‑‑‑‑‑‑CTTCTAAAAGTAACAGAGCTTACGTTTGCTGACAAAGAAGGTAGCTGGGGTACTCCGCTTGTTCCTGTGGAGCGTCCTCCTGGTCTCAATGATGTCGGCATGGTAGCCTGGCTTCTGGAAATGTGTACCCCTGAATTCCCATCTGGAAGGACTATATTGGTTGTTTCAAATGATGTGACCTTCAAGGCTGGTTCTTTTGGCCCAAAAGAGGATGCATTCTTTCGTGCAGTAACTGATCTTGCATGCGCAAGAAAACTGCCTTTAATTTATTTAGCAGCAAACTCTGGTGCCCGATTAGGTGTAGCTGAGGAAGTCAAAGCCTGTTTCAGAGTTGGTTGGTCTGAAGAATCTAAACCTGAGCAAGGTTTTCAGTATGTATATTTAACACCTGAGGATTATGCTCAGATTGGATCATCAGTGATAGCACATGAATTAAAGCTTGAGAGTGGAGAAACCAGATGGGTTATAGATACCATTGTTGGCAAGGAGGATGGCCTCGGGGTTGAAAACTTGAGTGGAAGCGGGGCCATTGCTGGTGCCTATTCTAAGGCATACAAGGAAACTTTTACATTGACCTACGTGACTGGTAGGACTGTTGGAATCGGTGCTTATCTTGCAAGGCTCGGGATGAGGTGCATACAGAGGCTTGATCAGCCCATAATTCTCACTGGTTTCTCAGCATTAAACAAACTTCTTGGTCGGGAGGTATACAGCTCTCACATGCAGCTTGGTGGACCTAAAATCATGGCAACTAATGGAGTTGTTCATCTTACAGTTTCAGATGACCTTGAAGGTGTCTCTTCTATTTTAAAGTGGCTTAGCTACATTCCTTCCCATGTAGGTGGTGTGCTTCCCATTGTAAAGCCCCTTGATCCTCCAGAGAGACTGGTGGAGTATTTCCCAGAGAATTCGTGCGATCCTCGTGCCGCCATTTCGGGAACTCTGGATAGTAACGGAAAGTGGCTGGGAGGCATTTTTGACAAGGATAGCTTTGTGGAGACGCTAGATGGGTGGGCGAGGACGGTTGTTACAGGAAGAGCAAAGCTTGGAGGAATCCCTGTGGGAATTGTTGCTGTAGAAACACAGACAGTTATGCAAATAATACCTGCTGATCCGGGCCAGCTTGATTCTCATGAGCGGGTTGTTCCTCAAGCCGGGCAAGTGTGGTTTCCTGATTCCGCAACTAAGACGGCTCAAGCAATACTGGATTTCAACAAAGAAGAGCTCCCGCTTTTCATTATGGCAAACTGGAGAGGCTTTTCAGGTGGGCAAAGGGACCTTTTTGAAGGAATCCTTCAGGCCGGTTCGACTATTGTGGAGAACCTGAGAACATACAAGCAGCCAATCTTCGTATACATCCCTATGATGGGTGAACTTCGTGGAGGGGCGTGGGTTGTTGTTGACAGTCGAATCAATTCAGACCACATTGAAATGTATGCCGAACGAACAGCTAAAGGTAATGTCCTTGAGCCAGAAGGAATGATTGAGATCAAATTCAGAACAAGAGAATTGTTGGAGTGTATGGGTAGACTTGATCAACAGTTGATAACCCTGAAGGCGAAACTTCAGGAAGCCAAGACTAACAGAGACCCTGGTACCATTGAGTCCCTGCAGCAGCAGATTAAATCCCGTGAGAAACAGCTTTTGCCTATGTATACCCAGATAGCTACCAAGTTTGCTGAACTTCATGATACTTCCCTGAGAATGGCTGCAAAGGGTGTAATCAGAGAAGTTCTGGACTGGGCTAACTCTCGTGCTGTCTTCTACCGGCGACTGCACCGAAGAGTTGGCGAGCATTCACTGATCAACAGTGTGAGAGATGCTGCT‑‑‑GGGGACCAATTGTCTCACACGTCTGCGATGAACATGATCAAAAGCTGGTATTTGAGTTCTGATATTGCCAAAGGAAGAGAAGAGGCTTGGTTGGATGATGAAGCCTTCTTCAGATGGAAGGCTGATCCTGCAAATTATGAGGATAAACTGAAGGAATTGCGCGTCCAGAAACTGTTGCTTCAATTGACAAATATTGGGGACTCAGCTCTAGATTTGCAAGCTCTACCTCAAGGTCTTGCTGCCCTTTTAAGCAAGTTGGAGGCATCAAGTCGTGACAAATTGACTGATGAACTTCGCAAGGTACTTGGA‑‑‑

>Mtr_ACC1_Medtr3g073820

‑‑‑‑‑‑‑‑‑‑‑‑‑‑‑‑‑‑‑‑‑‑‑‑‑‑‑‑‑‑‑‑‑‑‑‑‑‑‑‑‑‑‑‑‑‑‑‑‑‑‑‑‑‑‑‑‑‑‑‑‑‑‑‑‑‑‑‑‑‑‑‑‑‑‑ATGGCTAGCGTGGGTGAATACTGCAATGCCCTTGGGGGAAACATGCCAATTCATAGTATTTTGATTGCAAACAATGGATTGGCTGCAGTCAAGTTTATTCGCAGCGTTAGGAGTTGGGCTTATGAGACGTTCGGAACGGAAAATGCTATTTTCTTGGTTGCCATGGCTACTCCAGAGGACATGAGAATCAATGCAGAACATATAAGAATTGCTAATCAGTTTGTGGAAGTACCTGGTGGGACCAATAACAATAACTATGCCAATGTGCAACTTATTCTAGAG‑‑‑‑‑‑‑‑‑ATTGCTGAGATAACTCACGTTGATGCGGTGTGGCCTGGTTGGGGTCATGCATCAGAGGATCTTGAGCTTCCAAATGCATTGAAAGAAAAAGGAATTGTATTTCTTGGACCTCCTGCTATATCTATGGCAGCTTTGGGAGATAAAATTGCTTCATTGTTGATTGCTCAAGCAGCAGAAGTGCCAACCCTTCCATGGAGTGGTTCTCATGTGAAATTTCCTCCAGAAAGCGACTTGATTACTATTCCTGATGAAATTTACCGTGCATCATGTGTTTATACAACAGAAGAAGCAATTGCAAGTTGTCAAGTTGTAGGTTACCCTGCAATGATCAAGGCATCATGGGGGGGCGGCGGTAAAGGCATAAGAAAGGTTCATAATGATGACGAGGTAAGGGCATTGTTCAAGCAAGTTCAAGGTGAAGTTCCAGGCTCACCTATATTTATAATGAAAGTTGCTTCCCAGAGCCGACATCTTGAAGTCCAATTGATTTGTGATCAGTATGGAAATGTTGCAGCTTTGCACAGCCGTGATTGCAGTGTTCAAAGAAGGCATCAAAAGATTATTGAGGAAGGTCCCGTTACTGTAGCACCTCCAGAAACAGTGAAAGAACTTGAACATGCGGCTAGAAGATTAGCTAAATCTGTAAATTATGTTGGGGCAGCTACCGTTGAGTATCTTTATAGCATGGAAACTGGCGAGTACTACTTTTTAGAGTTGAACCCACGACTACAGGTTGAGCATCCTGTTACTGAATGGATAGCTGAGATAAATCTGCCAGCAGCACAAGTTGCAGTTGGGATGGGTATCCCTCTCTGGAAAATTCCTGAGATTAGGCGTTTCTATGGGATGGAACATGGTGGGGGAAATGATGCTTGGAGGAAAACATCAGTTTTAGGTACCCCTTTTGATTTTGACAAAGCACAATCTACAAAGCCAAAAGGTCATTGTGTGGCTGTGCGAGTGACTAGCGAGGACTCCGATGATGGTTTTAAGCCTACAGGAGGAAAAGTGCAGGAGCTCAGCTTTAAAAGCAAGCCAAATGTGTGGGCATACTTCTCTGTTAAGTCCGGAGGAGGAATTCATGAATTCTCAGATTCTCAATTTGGACATGTTTTTGCTTTTGGAGAATCTAGAGCTTTAGCAATTTCAAATATGGTACTAGGGTTGAAGGAAATTCAAATTCGAGGAGAAATCCGTACCAATGTTGATTACACAATTGATCTTCTGAATGCTTCAGACTACAGAGACAACAAAATTCACACAGGTTGGCTAGACAGTAGAATTGCAATGCATGTTAGAGCAGAGAGGCCTATGTGGTATTTGTCTGTTGTTGGAGCGGCACTCTATAACGCTTCTGCTAGCAGTGCAGCTTTGATTTCAGACTATGTTGGCTATCTTGAAAAGGGGCAAATTCCTCCTAAGCACATTTCTCTTGTCCGTTCTCAAGTTTCTTTGAACATTGAAGGAAGCAAATACATGATTGACATGATACGAGGAGGACCTGGATGTTACAAATTGAAATTGAATCAATCAGAGATAGAAGCGGAGATACATACTTTATGTGATGGAGGTTTACTGATGCAG‑‑‑‑‑‑‑‑‑TTGGACGGGAACAGTCATGTAATATATGCAGAGGAAGAAGCAGCTGGAACTCGCCTTCTAATAGATGGAAGAACTTGTTTTCTTCAGAATGATCACGATCCATCAAAGTTAATTGCAGAGACACCCTGCAAGCTTCTGAGATATTTGGTTGTAGATGACAGTCACATTGATGCAGACACACCATATGCTGAAATTGAGGTCATGAAGATGTGCATGCCTCTTCTTTCCCCTGCTTCTGGGATTATTCATTTCAAAAAGGCTGAAGGTCAAGCCATGCAGGCCGGTGAACTTATAGCAAAGCTTGATCTAGGTGACCCTTCTGCAGTAAGAAAGGCTGAACCCTTCAACGGGAGCTTCCCAATCCTGGGTCCTCCTACTGCAATTTCAGGTAAGGTTCACCAGAAATGTGCAGCAAGCTTAAACGCTGCACGAATGATTCTTGCTGGCTATGAGCAAAATATTGATGAAGTTGTGCAAAGTTTGCTCAATTGCCTTGAAAACCCCGAATTGCCTTTCCTTCAATGGCAAAAGATTTTGGCTGTTATGGCAACCCGACTTCCTAAAGACCTAAGAAATGAGTTGGAAGCAAAATATAAGGAGTTCGAGAGTATTTCA‑‑‑AGCTCCCAAATTATTGATTTTCCTGCGAAATTATTAATGGGGATTCTTGAAGCCCATCTTTCCTCCTGTCTTGAAAAAGAAAAAGGAGCCTTAGAAAGACTAATTGAACCTCTGATGAGTCTTGTAAAATCTTATGAGGGTGGAAGAGAGAGCCATGCTCATAAAATTGTTCAGTCTCTATTTGAAGAGTATCTTTCAGTTGAAGAACTATTCGGTGATAATATTCAGGGTGATGTAATTGAACGACTCCGTCTTCAATCCAAGAAAGATTTGTTCAAGATTGTAGATGTTGTGCTCTCTCATCAGGGTGTCAAGAGAAAAAACAAACTGATACTGCGACTAATGGATAAACTGGTTTACCTTGATCCTGCTGCCTATAGTGACCAATTAATTCGATTCTCCAAACTCAACCATATAGTTTATTCTGAGTTGGTTCTTAAGGCAAGTCAAATGTTAGAGCAAACTAAACTGAGTGAACTTGGATCCAGCATTGCTAGAAGTCTTTCTGAACTAGAATTTTTTACCGAGGATGGTGAAAATACTGATACTCCGAAGAGGAAGAGTTACATTAATGATCGAATGGAAGACCTTGTGAGCTCTCCTTTGGCTGTTGAAGATGCTCTTGTTGGTTTATTTGATCACAGTGATCATGCCCTTAAACGGAGGGCTGTGGAAACTTATATCCGTAGGCTCTACCAGCAATATCTTGTCAAAGGGAGCTTAAGGATGCAGTGGCACAAATCTGGCCTTATTACTACATGGGAATTCATAGAAGAAAATTTTGAA‑‑‑‑‑‑‑‑‑‑‑‑‑‑‑‑‑‑‑‑‑‑‑‑‑‑‑‑‑‑‑‑‑‑‑‑‑‑‑‑‑‑‑‑‑‑‑‑‑‑‑‑‑‑‑‑‑‑‑‑‑‑‑CAGAAGAAATTGGGAGTGATGGTTGTAATTAAATCTCTTCAATTTTTGCCAGCAATAACCAGTGTTGCATTTAGGGAAGCAACCAAGAACTTTCACGATGCA‑‑‑TTTAAAAGTGGTTCTGGCGACTCAAGTAACCATGGGAATGTGATGCATATTGGATTAGTGGGCATCAACAACCAAACGAGTTTACTTCAAGATAGTGGTGATGAGGATCAGGATAAAAAAAGAATCGATATGTTGATCAAAGTACTCAGAGAGCAGGAAGTAGGGACCATAATACATGCTGCAGGTGTTGGAGATATTAGTTGTATCATACATAAGGATGAAGGGTTTGCTCCAATGAGGTATTCCTTTCACTGGTCAGCTGAAAAGCTATATTATGAAGAGGAACCTCTGTTGCGCCATCTGGAACCTCCCCTATCCATTTATCTTGAATTGGACAAGCTTAAAGGCTATGAAAATATCAGGTATACACCATCCCAAGATCATCAATGGCACCTCTACACTGTTGTGGATACTAAGCCACTACCAATTCAAAGAATGTTTCTTCGAACACTTCTCAAACAGCCAATCCGAAATGAAGGACACTCTTCTTATCAAAGACTGGATGGAGAAACGTCTCGTACCCAATTGGCTATGTCCTTTACTTCAAGGAGCATTCTTAGGTCCCTAATGGGTGCAATGGAGGAGTTGGAACTTAACTCACACAATACCACCATCAAATCTGAACATGCTCATATGTACCTCTATATCATCCGCAAGCAACAAGTAGATGATCTTGTGCCTAATTCCAAGAAAATTAACATCGAAGTTGGTCAAGAAGAAACAACTGTTGAGGCAATATTGGAAGATCTGGCACGGGAAATCCATTCCTCTGTCGGTGTAAGAATGCACAGATTAGGTGTTTTTGTGTGGGAAGTCAAGCTTTGGATTACAGCATATGGACAAGCAAATGGTGCTTGGAGGGTCATTGTAAACAACGTGACTGGTCATACTTGCACCGTACATATATATCGAGAAATGGAGGATGCCACCGCTCGTAAAGTGGTTTACAGTTCAGTCAATGTAAAGGGTCCACTGCATGGTGCATCAGTGAACAAAAACTATCAACCTTTGAGAGCTATTGACCGAAAACGTCTTGCAGCTAGAAAGAACAGCACCACATACTGCTATGATTTTCCACTTGCATTTCAAACATCCTTGGAACAGTCATGGTCAATTCAACAAACAAGAATTCAAATATCCAAAGGTAAGGACCTCCTAAAAGTAACAGAGCTCAAATTTTCAGAGAAAGAAGGTATCTGCGGTACTCCTCTTGTACCTGTCGAACGTCCTCCTGGACTCAATGATGTTGGCATGGTAGCCTGGTTGATGGATATGTATACGCCTGAATTCCCATCTGGAAGGACAATATTGGTTGTTTCGAATGATGTGACCTTCAAGGCCGGTTCTTTTGGCCCAGGAGAAGATGCGTACTTTAGAGCAGTAACTGATCTTGCCTGTGCGAAGAAAATACCTTTAATTTACTTAGCAGCAAGTTCTGGTGCCCGGTTAGGTGTGGCAGAGGAAGTCAAAGCTTGTTTCAGAGTCGGTTGGTTTGAGGAATCTAATCCTGAGCATGGTTTTCGCTATGTATATTTAACACCTGAGGATTATGTTCGAATTAGATCATCCGTGATGGCACATGAATTAAAGATTGAAAGTGGAGAAACCAGATGGGTTATAGATACCATTCTCGGCAAAGACGTTGGACTAGGAGTTGAAAACTTGAGTGGCAGTGGGGCCATTGCCGGTGCCTATTCAAGGGCATACAAGGAAACCTTTACACTAACATATGTGACCGGTACAACTGTTGGAATTGGTGCTTATCTTGCAAGGCTGGGGATGAGATGCATACAGAGGTTTGATCAGCCTATGATTCTTACCGGTTTTTCAGCATTAAACAAACTCCTTGGTAGGGAAGTGTACAGCTCTCACATGCAACTTGGTGGATCTAAAATCATGGCAACAAATGGAGTTGTTCATCTTACAGTTTCGGATGACCTTGAAGGTGTTTCTTCTATTTTGAAGTGGCTTAGCTACATCCCTTCCCATGTAGGTGGTGCACTTCCCGTTGTAAATCCCCTCGATTCCCCAGAGAGGGAAGTGGAATATTTACCGGAAAATTCATGTGATCCCCGTGCTGCCATTTCCGGAATTCTAGATGTTAATGGAAAGTGGCTGGGAGGCATTTTTGACAAGGACAGCTTTGTGGAGACACTAGAAGGATGGGCTAGGACAGTTGTTACAGGAAGGGCAAAGCTTGGTGGAATCCCTGTGGGAATCGTTGCGGTAGAAACACAGACAGTAATGCAAATAATACCTGCTGATCCATGTCAGCTTGATTCTCATGAGAGGGTTGTTCCTCAAGCCGGCCAAGTGTGGTTTCCTGATTCTGCGACCAAAACTGCCCAAGCGATATTGGATTTCAATAGAGAAGAGCTCCCGCTTTTCATTCTCGCAAATTGGAGAGGCTTTTCGGGTGGGCAAAGGGACCTTTTCGAAGGAATTCTTCAGGCTGGTTCAACTATTGTGGAGAACCTTAGAACATACAAGCAACCCATTTTTGTATACATCCCAATGATGGGTGAACTCCGAGGTGGGGCATGGGTTGTTGCTGACAGTCGAATAAATTCAGGCCATATTGAAACATATGCTGAGCGAACGGCTAAAGGTAACGCCCTTGAACCGGAAGGAATGATTGAGATCAAATTTAGAACAGGAGAAATGTTGGAGTGTATGAGAAGACTTGATCAGCAACTGATTACTCTGAAGGAAAAACTTTCGGAAGCCAAGAGCAACAAGGACTTTGGTACATATGATTCTCTGCAACAGCAGATTAGATTCCGTGAGAAACAGCTTTTGCCTTTGTATACTCAGATAGCTATCAAGTTTGTTGAACTGCATGATACTTCCTTAAAAATGGCTGCAATAGGCGCAATGAAAGAAGTTATTGATTGGCGTGACTCACGTTCTTTCTTCTATCGGAGACTGCACAGGAGAATCGGTGAGCACTCACTGATCAACATTGTGAGAGATGCTGCT‑‑‑GGTGACCAATTGACACATGTATCTGCCATGAACTTGCTCAAAGAATGGTATATGAATTCTGATATCTCCAAAGGTAGTGAAGATGCTTGGTTGGACGATGAAGCCTTCTTCAGATGGAGGAATGATCCCTCAAATTACGAGGATAAACTAAAGGAATTGCGCATCCAAAGATTGTTGCTTCAGTTGAAAAATATTGGTGACTCGGATCTAGATTTACAAGCTCTATCTCAAGGTCTTGCCACCCTTTTAAGTAAGTTTGAGGATCATAACAAGCATCACCATGTTTGGTCTATTTATTTGCTGGTCGAT‑‑‑

>Mtr_ACC2_Medtr3g073860

ATGGCTAGCGCAGGGCGTGGAAAT‑‑‑‑‑‑‑‑‑GGATACTTAAATGGTGTGCTACCGAGTAGGCACCCTGCTACTACAACCGAAATAGATGAATACTGCAATGCCCTTGGAGGAAACAAGCCGATTCATAGCATATTGATTGCAAACAATGGAATGGCAGCAGTCAAGTTTATACGCAGTGTTAGGAGTTGGGCTTACGAGACGTTTGGCACAGAAAAAGCTATCTTGTTGGTTGCCATGGCTACTCCAGAGGATATGAGAATCAATGCAGAACATATCAGAATAGCTGATCAATTTGTGGAAGTACCTGGTGGGACAAATAACAATAACTATGCCAATGTGCAGCTTATTCTAGAG‑‑‑‑‑‑‑‑‑ATTGCTGAGATAACTCACGTTGATGCGGTGTGGCCTGGTTGGGGTCATGCATCAGAAAATCCTGAGCTTCCAGATGCATTAAAAGCAAAAGGAATTGTATTCCTTGGACCTCCTGCTATATCTATGGCAGCATTGGGAGACAAAATTGGTTCATCATTGATTGCTCAAGCAGCAGAAGTGCCAACCCTTCCATGGAGTGGTTCTCATGTGAAAATTCCTCCAGAAAGCGACTTGATTACTATTCCTGATGAAATTTACCGTGCAGCATGTGTTTATACAACAGAAGAAGCAATTGCAAGTTGTCAAGTAGTAGGTTACCCTGCAATGATCAAGGCATCTTGGGGTGGTGGTGGTAAAGGCATAAGAAAGGTTCATAATGATGATGAGGTAAGGGCATTGTTCAAGCAAGTTCAAGGTGAAGTACCAGGCTCACCTATATTTATAATGAAAGTTGCTTCCCAGAGCCGACATCTTGAAGTCCAATTGATTTGTGATCAGCACGGAAATGTTGCAGCTTTGCACAGCCGTGATTGCAGTGTTCAAAGAAGGCATCAAAAGATTATTGAAGAGGGTCCCATTACTGTAGCACCTCCAGAAACGGTGAAAGAACTTGAACAGGCGGCTAGAAGATTAGCTAAATCTGTCAATTATGTTGGGGCAGCTACCGTTGAGTATCTTTATAGCATGGAAACTGGCGAGTACTACTTTTTAGAGTTGAACCCCCGACTACAGGTTGAGCATCCTGTTACTGAATGGATAGCTGAGATAAATCTGCCAGCAGCACAAGTTGCAGTTGGGATGGGTATCCCGCTCTGGCAAATTCCTGAGATTAGGCGTTTCTATGGGATGGAACATGGCGGGGGAAATGATGGTTGGAGGAAAACATCACTGTTAGCTACCCCTTTTGATTTTGACAAAGCACAATCTACAAAGCCAAAAGGTCATTGTGTGGCTGTGCGAGTGACCAGCGAGGACCCTGATGATGGTTTTAAGCCTACAGGAGGAAAAGTGCAGGAGCTAAGCTTTAAAAGCAAGCCAAATGTGTGGGCTTACTTCTCTGTTAAGTCCGGAGGAGGAATTCATGAATTCTCAGATTCTCAATTTGGACATGTGTTCGCTTTTGGAGAATCTAGAGCTTTAGCCATTGCAAATATGGTACTGGGGTTGAAGGAAATTCAAATTCGAGGAGAAATTCGTACCAATGTTGATTACACAATTGATCTTCTGAATGCTTCAGACTACAGAGACAACAAAATTCACACAGGATGGCTAGACAGTAGAATTGCAATGCGGGTTAGAGCAGAGAGGCCTCCCTGGTATCTGTCTGTTGTTGGAGGGGCACTCTATAAAGCTTCTGCTAGCAGTGCAGCTTTGGTTTCAGACTATGTTGGCTATCTTGAAAAGGGGCAAATCCCTCCTAAGCACATTTCTCTTGTCCATTCTCAAGTTTCTTTGAGCATTGAAGGAAGCAAATACACGATTGACATGATACGAGGAGGACCTGGAAGTTACAAACTGAAATTAAATCAATCAGAGATAGAAGCGGAGATACACACTTTACGTGATGGGGGTTTGCTTATGCAG‑‑‑‑‑‑‑‑‑TTGGATGGAAACAGTCATGTAATATATGCAGAGGAAGAAGCAGCTGGAACTCGGCTTTTAATCGATGGAAGGACTTGCTTGCTTCAGAATGATCACGATCCATCAAAGTTAATTGGAGAGACACCGTGCAAGCTTCTGAGATATTTGGTTGCGGATGATAGTCACATTGATGCAGACACACCATATGCTGAAGTTGAGGTCATGAAAATGTGCATGCCTCTTCTTTCCCCTGCTTCTGGGATTATTCATTTCAGAATGGCTGAAGGTCAAGCCATGCAGGCTGGTGAACTTATAGCAAAGCTTGACCTAGATGATCCTTCTGCAGTAAGAAAGGCAGAACCCTTCACAGGGAGCTTTCCTATCCTGGGCCCTCCTACTGCAATTTCAGGTAAAGTTCATCAGAAATGTGCAGCAAGCTTAAACGCTGCACGGATGATTCTTGCTGGCTATGAGCACAATATTGATGAAGTTGTGCAAAGTTTGCTCAATTGCCTTGACAGCCCTGAACTGCCTTTCCTTCAATGGCAAGAGTGCTTTGCAGTTTTGGCGACCCGTCTTCCCAAAGATCTAAGAAATGAGTTGGAAGCTAAATATAAGGAGTTCGAAATTATTTCA‑‑‑AGCTCCCAAACTATTGATTTCCCTGCCAAACTATTGAAGGCAATCTTTGAAGCTCATCTTTCCTCCTGTCCTGAAAACGAAAAAGGAGCTTTAGAAAGACTAGTTGAACCGCTGACAAGTCTTGTTAAGTCTTATGAGGGTGGAAGAGAGAGCCATGCTCATAAAATCGTTCAATCTCTATTTGAAGAGTATCTTTCAGTTGAAGAACTATTCAGCGATAATATACAGGCTGATGTAATTGAACGGCTCCGTCTTCAATACAAGAAAGATTTGTTGAAGATTGTAGATATCGTGCTCTCTCATCAGGGTGTCAAGAGCAAAAACAAGCTGATACTGCGACTAATGGATAAACTGGTTTACCCTAATCCTGCTGCCTATAGGGATCAGTTAATCCGATTCTCCCAACTCAACCATATAGTTTATTCTGAGTTGGCTCTTAAGGCAAGTCAACTGTTGGAGCAAACTAAACTCAGTGAACTTCGATCCAGCATTGCTAGAAGTCTTTCTGAACTAGAAATGTTTACGGAGGATGGTGAAAATATTGATACTCCGAAGAGGAAGAGTGCCATTAATGACAGAATGGAGGACCTTGTGAGCGCTCCTTTGGCTGTTGAAGATGCCCTTGTTGGTTTATTTGATCACAGTGATCACACCCTTCAAAGGAGAGTTGTGGAAACTTATATCCGTAGGCTCTATCAGCCATATCTTGTCAAAGATAGCATCAGGATGCAGTGGCACAGATCTGGCCTTATTGCTACATGGGAATTCTTAGAAGAACACGTTGAACGGAAGAAT‑‑‑GGGGTTGAAGAC‑‑‑‑‑‑‑‑‑‑‑‑‑‑‑AAAACACTGGTGGAGAAACATAGTGAGAAGAAATGGGGAGTGATGGTTGTAATTAAATCTCTTCAATTTTTGCCAGCAATTATCAGTGCTGCATTAAGAGAAGCAACTAATAACTTTCACGATCCA‑‑‑CTTAAAAGTGGTTCTGGTGACTCAAGTAACCATGGTAATATGATGCATATTGGATTAGTGGGCATCAACAACCAAATGAGTTTACTACAAGACAGTGGTGATGAGGATCAGGCTCAAGAAAGAATTGATAAGTTGGCCAAAATACTCAGAGAGCAGGAAGTAGGGTCCATAATACATGCTGCAGGTGTTGGAGATATTAGCTGTATCATACAGAGGGATGAAGGGCGTGCTCCAATGAGGCATTCCTTTCATTGGTCATCTGAAAAGCTACATTATGTAGAGGAACCTCTGTTGCGCCATCTTGAACCTCCCCTATCCATTTATCTTGAACTGGACAAGCTTAAGTGCTATGAGAATATTCGCTATACACCATCCCGAGATCGTCAATGGCACCTCTACACGGTTGTGGATACCAAGCCACAACCAATTCAAAGAATGTTTCTTCGAACACTTATCAGACAGCCAACCACAAATGAAGGATACTCTTCTTATCAAAGACTGGATGCAGACACGTCCCGTACCCAATTGGCTATGTCTTATACTTCAAGGAGCATTTTTAGGTCCCTAATGGGCGCAATGGAGGAGTTGGAACTTAACTCACACAATACCACCATCAAATCTGAACATGCTCATATGTACCTCTATATCATACGCGAGCAGCAAATAGATGATCTTGTGCCTTATTCCAAGAAAATTAACATAGAAACTGGCCAAGAAGAAACAACAGTTGAGGCAATCTTGGAAGAACTGGCACAGGAAATCCATTCCTCTGTTGGTGTAAGAATGCACAGATTAGGCGTTTTTGTGTGGGAAATCAAGCTCTGGATTACAGCATGTGGACAGGCAAATGGTGCTTGGAGGGTCATTGTAAACAATGTGACTGGTCATACATGCACTGTACATATATATCGAGAGATGGAGGATGCCATCACTCATAAAGTGGTCTACAGTTCAGTCACTCTAAAGGGACCACTGCATGGTGTACCGGTGAATGAAAACTATCAACCTTTGGGAGTTATTGACCGAAAACGTCTTGCAGCAAGAAAGAACAGCACCACATACTGCTATGATTTTCCCCTTGCATTTCAAACATCCTTGGAACAGTCCTGGTCAATTCAGCAGACAGGAATTCAAAAAGCCAACGATAAGGATCTCCTAAAAGTAACAGAGCTTAAATTTTCCGAGAAAGATGGTAGTTGGGGTACTTCTCTTGTTCCTGCAGAGCGTGTTGCTGGACTCAATGATGTTGGCATGGTAGCCTGGTTGATGGAAATGTGTACGCCTGAATTCCCATCTGGAAGGACAATATTGGTTGTTTCAAATGACGTTACCTTCAAGGCCGGTTCTTTTGGCCCAAGAGAGGATGCATTCTTTAGAGCAGTAACCGATCTTGCCTGTGCAAAGAAAATACCTTTAATTTACTTGGCAGCAAATTCTGGTGCCCGTTTAGGTGTTGCCGAGGAAGTCAAAGCTTGTTTCAAAGTTGGTTGGTCTGAGGAATCTAAACCCGAGCATGGTTTTCAGTATGTATATTTAACACCTGAGGATTATGCTCGAATCGGATCATCAGTGATGGCACATGAATTAAAACTTGAAAGTGGAGAAACCAGATGGGTTATAGATACCATTGTTGGCAAAGAAGATGGACTGGGAGTTGAAAACTTGAGTGGTAGTGGGGCCATTGCTGGTGCCTATTCAAGGGCATACAAGGAAACCTTTACATTGACATATGTTACCGGTAGGACTGTTGGAATTGGTGCTTATCTTGCTAGGCTTGGGATGAGGTGCATACAGAGGCTTGATCAGCCTATAATTCTTACCGGGTTTTCAGCATTAAACAAACTTCTTGGTAGGGAGGTGTACAGCTCTCACATGCAACTTGGTGGACCGAAAATCATGGCAACAAATGGAGTCGTTCATCTCACTGTTTCGGACGACCTTGAAGGAGTTTCTTCTATTTTGAAGTGGCTTAGCTACGTTCCTTCTCATGTAGGTGGTGCACTTCCCATTGTAAAGCCCCTTGATCCCCCAGAGAGGGAAGTGGAGTATTTACCGGAAAATTCATGCGATCCTCGTGCTGCCATTTCTGGAACTCTGGATGTTAATGGAAAGTGGCTGGGAGGTATTTTTGACAAGGACAGCTTTGTGGAGACACTAGAAGGATGGGCTAGGACGGTTGTTACAGGAAGGGCAAAGCTTGGAGGAATTCCAGTGGGAATTGTTGCGGTGGAAACGCAAACAGTTATGCAAATAATACCTGCTGATCCAGGCCAACTTGATTCTCACGAGAGGGTTGTTCCTCAAGCTGGGCAAGTGTGGTTTCCTGATTCTGCGACCAAGACGGCCCAAGCGATATTGGATTTCAACAGAGAAGAGCTCCCACTTTTCATTATCGCAAACTGGAGAGGCTTTTCAGGTGGACAAAGGGACCTTTTTGAAGGAATTCTTCAGGCTGGTTCGACTATTGTGGAGAACCTTAGGACATACAAGCAGCCCATATTTGTATACATCCCAATGATGGGTGAACTCCGAGGCGGGGCTTGGGTTGTTGTCGACAGCCGAATCAACTCAGACCACATTGAAATGTATGCCGAACGAACGGCTAAAGGTAACGTCCTTGAGCCGGAAGGAATGATTGAGATCAAATTTAGAACAAGAGAATTGTTGGAGTGTATGAGAAGACTTGATCAACAATTGATTACTTTGAAGGAAAAACTTTCTGAAGCCAAGAGTAACAAAGACTTTGGTGCATATGATTCTCTGCAGCAGCAGATTAGATTCCGTGAGAAACAGCTTTTGCCTTTGTATACTCAGATAGCTACCAAATTTGCTGAACTCCATGATACTTCATTAAGAATGAAAGCAAAGGGTGTAATCAGGGAAGTTCTTGATTGGCGTAACTCGCGTTCTGTCTTCTATCGGCGACTGCACAGGAGAATCGGTGAGCACTCACTGATCAACAGTGTGAGAGATGCTGCT‑‑‑GGTGACCAATTGTCATATGTTTCTGCCATGAACTTGCTCAAAGAATGGTATCTGAATTCTGATATCGTCAAAGGCAGTGAAGATGCTTGGTTGGACGATGAAGCCTTCTTCAGATGGAGGGATGATACATCATATTATGAGGATAAACTAAAGGAATTGCGCGTCCAGAGACTGTTGCTTCAGTTGACAAATATTGGCGACTCGGCTCTAGATTTACAAGCTCTACCTCAAGGTCTTGCCGCCCTTTTAAGCAAGTTGGAAGCATCGAGTCGCGATAAGTTGACCAATGAACTTCGCAAGGTACTTGGT‑‑‑

>Pvu_ACC_Phvul.009G028700

ATGGCTGACATTGGGCGTCGGAAT‑‑‑‑‑‑‑‑‑GGATATCCAAATAGTGTACAGCCAAATAGGCACCCTGCTGCAATATCTGAAGTAGATGAATTCTGCAGTGCCCTTGGCGGAAACAGGCCAATTCATAGCATTTTAATTGCAAACAATGGAATGGCAGCAGTCAAGTTCATACGTAGTGTTAGAAGCTGGGCCAATGAGACATTTGGCACAGAGAAAGCCATCTTGTTGGTTGCCATGGCTACTCCAGAGGACATGAGAATCAATGCGGAACATATAAGAATAGCCGATCAATTTGTCGAAGTACCTGGTGGGACCAATAACAATAACTATGCCAATGTGCAGCTTATTCTTGAG‑‑‑‑‑‑‑‑‑ATGGCTGAGATAACTCATGTTGATGCGGTGTGGCCTGGTTGGGGTCATGCTTCAGAAAATCCTGAGCTTCCAGATGCGTTGAAAGCAAGAGGAATTGTATTTCTTGGACCTCCTGCCGTATCTATGGCTGCTTTGGGAGATAAAATTGGTTCATCCTTGATTGCTCAAGCAGCAGAAGTGCCAACCCTTCGATGGAGTGGTTCTCATGTGAAAATTCCTCCTGAAAGTTCCTTGATTACTATTCCTGATGAAATTTATCGGGAAGCATGTGTGTACACAACAGAGGAAGCAGTAGCGAGTTGTCAAGTTGTAGGATACCCTGCCATGATCAAAGCATCTTGGGGTGGTGGTGGTAAAGGAATAAGAAAGGTTCATAATGATGATGAGGTAAGGGCATTGTTCAAGCAAGTTCAAGGTGAAGTTCCCGGTTCACCTATATTTATAATGAAGGTTGCATCCCAGAGTCGACATCTAGAAGTCCAGTTACTTTGTGATCAGTACGGAAATGTAGCTGCTTTGCATAGTCGTGATTGCAGTATTCAAAGGAGGCACCAAAAGATTATTGAGGAGGGCCCCATTACCGTAGCACCCATAGAAACAGTGAAACAACTGGAACAAGCAGCTAGAAGGTTGGCTAAATCTGTAAATTATGTCGGGGCAGCTACTGTTGAGTATCTTTTTAGTATGGAAACTGGAGAGTACTACTTTTTAGAGTTGAACCCTCGGCTACAGGTTGAACATCCTGTTACTGAGTGGATAGCAGAGATAAATCTGCCAGCAGCCCAAGTTGCCGTTGGGATGGGTGTCCCTCTCTGGCAAATTCCTGAAATAAGGCGTTTCTATGGGGTGGAGCATGGTGGAGGGTATGATGCTTGGAGAAAAACTTCAGTTTTAGCTACCCCTTTTGATTTTGACAAAGCACAATCCACAAGGCCAAAAGGTCATTGTGTGGCTGTGCGAGTGACTAGTGAGGACCCTGATGATGGCTTTAAGCCCACAAGTGGCAAAGTGCAGGAGCTTAGTTTTAAAAGCAAGCCAAATGTGTGGGCATACTTCTCGGTCAAGTCTGGAGGAGGAATTCATGAATTCTCAGATTCACAGTTTGGGCATGTGTTCGCTTTTGGAGAATCTAGGGCTTTAGCAATTGCAAATATGGTTCTAGGGCTGAAGGAGATTCAAATTCGGGGAGAAATCCGTACCAATGTTGATTACACGATTGATCTTCTGAATGCTTCTGACTACAGAGACAACAAAATTCACACAGGTTGGCTGGACAGTAGAATTGCAATGCGGGTTAGAGCAGAGAGGCCTCCGTGGTATCTTTCTGTTGTTGGAGGGGCACTCTATAAAGCTTCTGCTAGCAGTGCAGCTTTGGTTTCAGACTATGTTGGCTATCTTGAAAAGGGACAAATCCCTCCCAAGCACATATCTCTTGTCCATTCTCAAGTGTCCTTGAACATTGAGGGAAGCAAATATACGATTGACATGATTCGAGGTGGATCTGGAAGTTATAGATTGAGAATGAATCAATCAGAGATGGAAGCAGAAATACATACTTTACGTGATGGAGGTTTGCTAATGCAG‑‑‑‑‑‑‑‑‑CTGGATGGAAACAGTCATGTAATATATGCAGAGGAAGAAGCAGCTGGAACTCGCCTTCTAATTGATGGAAGGACTTGCTTGCTTCAGAATGATCATGATCCATCAAAGTTGGTGGCGGAGACACCATGCAAGCTTTTGAGATATTTGGTTGAAGATGACAGTCATGTTGATGCTGACACACCATATGCTGAAGTTGAGGTCATGAAAATGTGCATGCCTCTTCTTTCACCTGCTTCTGGGATTATTCATTTCAAAATGTCTGAAGGTCAAGCAATGCAGGCTGGTGAACTTATAGCAAGTCTTGACCTAGATGATCCTTCAGCTGTAAGAAAGGCAGAACCCTTCACGGGGAGCTTCCCAGTCCTAGGACCTCCTACTGCAATTTCTGGTAAAGTTCATCAAAAATGCGCTGCAAGCTTAAATGCTGCACGGATGATTCTTGCTGGGTATGAGCACAATATTGATGAAGTTGTGCAAAGTTTGCTTAATTGTCTTGATAGTCCTGAGTTGCCTTTCCTTCAATGGCAAGAGTGTCTGGCAGTTTTGGCAACCCGTCTTCCCAAAGATCTAAAAAATGAGTTGGAATCAAGATATAAGGAGTTCGAGAGGATTTCA‑‑‑AGCTCCCAAATTGTTGATTTCCCTGCCAAATTATTGAAGGGAATTCTTGAAGCTCATCTTTCTTCCTGTCCTGACAAAGAAAAAGGAGCCCAAGAAAGACTAGTTGAACCTCTGCTGAGTCTTGTTAAGTCTTATGAGGGTGGAAGAGAGAGCCATGCCCATATAATTGTTCAATCCCTTTTTGAAGAATATCTTTCTGTTGAGGAATTATTTAGCGACAATATTCAGGCTGATGTAATTGAACGTCTGCGTCTTCAATACAAGAAAGATCTATTGAAGATTGTTGATATAGTGCTCTCTCATCAGGGTGTCAAGAGTAAAAATAAGCTGATATTGCGTCTGATGGATAAATTGGTTTACCCTAACCCTGCTGCCTATAGGGATCAGTTAATCCGTTTTTCCTTACTCAACCATACAAATTATTCAGAGTTGGCACTAAAGGCAAGTCAACTGCTGGAACAAACTAAATTAAGTGAACTTCGATCCAACATTGCTAGAAGTCTTTCTGAACTAGAGATGTTCACGGAGGATGGTGAAAATATTGATACTCCCAAAAGGAAGAGTGCCATCAATGACCGAATGGAGGACCTCGTTAGTGCTCCTTTGGCTGTTGAAGATGCCCTTGTGGGTTTATTCGATCACAGTGATCACACCCTTCAGAGGAGGGTCGTGGAAACTTATATACGCAGACTCTATCAGCCGTATCTTGTCAAAGGGAGTGTCAGGATGCAATGGCACAGGTCTGGTCTTATTGCCACATGGGAGTTCTATGATGAGTACATTGAAAGGAAGAAT‑‑‑GGGGTTGAAGAC‑‑‑CTAACTTTGAAGAAATCAATCGAGGAGAAAGACAGTGAGAAAAAGTGGGGAGTGATGGTTGTAATTAAATCTCTTCAATTTTTGTCTGCAATTATCAGTGCTGCATTGAGGGAGGCAACCAATAACCTTCATGAAGCC‑‑‑CTGACAAGTGGTTCTGCGGAACCAGTTAACCATGGTAATATGATGCATATTGGATTAGTGGGCATAAACAACCAGATGAGTTTGTTGCAAGACAGTGGTGATGAGGATCAGGCTCAAGAAAGAATCAATAAATTAGCTAAAATACTAAAAGAGCAGGAAGTAGGCTCCACCATTAGAGCTGCAGGTGTTGGTGTAATCAGCTGTATCATCCAAAGGGATGAAGGACGTGCTCCAATGAGGCACTCTTTTCACTGGTCAGAAGAAAAGCTGTACTATGCAGAGGAACCTCTGTTGCGTCATTTGGAACCTCCACTATCCATTTATCTTGAATTGGACAAACTTAAGGGCTATGAGAATATTCGGTATACTCCATCTCGAGATCGTCAATGGCACCTCTACACGGTTATGGATAACAAGCCACAACCAATTCAAAGAATGTTTCTTCGCACACTTCTAAGACAGCCAACCACAAATGAAGGATTTTCTTCGTATCAAAGGCTGGATGCAGAAACATCTCGAATCCAATTGGCTATGTCCTTTACATCAAGGAGCATTTTTAGGTCCTTGATGGCTGCAATGGAGGAGTTGGAACTTAATGCACACAATGCCAATATAAAATCTGAGCATGCTCATATGTACCTCTATATCATACGGGAGCAACAAATAGACGATCTTGTTCCCTATCCGAAGAGAATTAATGTAGATGCTGGGAAAGAAGAAACAACAGTCGAGGCAATCTTGGAAGAATTGGCACAGGAAATCCATTCATCTGTTGGTGTAAGAATGCATAGATTAGGGGTTGTTGTGTGGGAAGTCAAGCTTTGGATGGCAGCCGGTGGACAGGCAAATGGTGCTTGGAGGGTCATTGTAAACAATGTGACTGGTCATACATGCACTGTACACATATACCGAGAAAAGGAGGATACCAACACTCATAAAGTGGTATATAATTCAGTCTCGGTTAAGGGTCCACTGCATGGTGTGCCAGTGAACGAAAATTATCAACCTTTGGGAGTTATTGACCGAAAACGTCTTTCAGCAAGAAAGAACGGCACCACATACTGCTATGATTTTCCTCTTGCATTTGAAACTGCTTTGGAACAGTCATGGGCAGTCCAACATCCAGGATTTCAAAGAGCCAAAGATAAAAATCTTCTAAAGGTAACAGAGCTTAAATTTGCAGAGAGAGAAGGTAGCTGGGGTACTCCTCTTGTTCCTGTGGAGCATTACCCTGGACTCAATGATGTGGGTATGGTAGCCTGGTTTATGGATATGCGTACCCCTGAGTTCCCATCTGGAAGAACAATATTGGTTGTTTCAAATGATGTGACCTTCAAGGCTGGTTCTTTTGGCCCGAGAGAGGATGCTTTCTTCCGTGCTGTAACTGATCTTGCGTGTAAAAGAAAACTACCTTTAATTTATTTAGCAGCAAATTCTGGTGCCCGTTTAGGTGTAGCAGAGGAAGTCAAATCCTGTTTCAGAGTTGGTTGGTCTGAGGAATCTAGTCCTGAGCATGGCTTTCAGTATGTATATTTAACACCTGAGGACTATGCTCGGATAGAATCTTCAGTCATGGCACATGAATTAAAGCTTGAAAGTGGAGAAACTAGATGGGTTATAGATACCATTGTTGGCAAGGAGGATGGCCTTGGGGTTGAAAACTTGAGTGGCAGTGGGGCTATTGCTGGTGCTTATTCAAGGGCATACAAGGAAACTTTTACATTGACATATGTGACTGGCAGAACTGTTGGAATAGGTGCTTATCTTGCTAGGCTTGGGATGAGATGCATACAGAGGCTTGATCAGCCCATAATTCTTACTGGTTTTTCAGCACTAAACAAACTTCTTGGTCGGGAGGTATACAGCTCTCACATGCAACTTGGTGGACCCAAAATCATGGCTACAAATGGAGTAGTTCATCTTACAGTTTCCGACGATCTTGAAGGTGTTTATTCTATTTTGAAGTGGCTTAGCTACATTCCTTCTCATATAGGTGGACCGCTTCCCATTGTTAAGCCTCTTGATCCTCCAGAAAGACCAGTGGAGTATTTACCAGAAAACTCATGTGATCCTCGTGCTGCCATTTCTGGAACCCTGGATGGTAATGGAAGATGGCTGGGAGGCATCTTTGACAAGGACAGCTTTGTGGAGACACTGGAGGGATGGGCTAGAACAGTTGTTACAGGAAGGGCAAAGCTTGGAGGAATCCCTGTGGGAATTGTTGCTGTAGAAACACAGACAGTAATGCAAATAATACCTGCTGATCCTGGTCAGCTCGATTCTCATGAGAGGGTTGTTCCTCAAGCTGGGCAAGTCTGGTTTCCTGATTCTGCAACCAAGACAGCCCAAGCAATATTGGATTTCAATAGAGAAGAACTCCCGCTTTTCATTCTCGCAAATTGGAGAGGCTTTTCAGGAGGGCAAAGGGACCTTTTTGAAGGAATTCTTCAGGCTGGTTCAACTATTGTGGAAAACCTTAGAACATACAAGCAGCCCATATTTGTGTACATCCCAATGATGGGTGAACTCCGAGGTGGTGCATGGGTGGTTGTTGACAGTCAAATCAATTCAGACCACATTGAAATGTATGCTGATCAAACAGCTAAAGGTAATGTCCTTGAGCCAGAAGGAATGATTGAGATCAAATTCAGATCAAGGGAATTGTTGGAGTGTATGGGTAGACTTGATCAACAGTTGATAACTCAGAAGGCAAAACTTCAGGAAGCCAAGAGTAACAGGGACCTTGCCGCCTTTGAATCCCTACAGCAGCAGATTAAATCCCGTGAGAAACAGCTTTTGCCTGTGTATACCCAGATAGCTACAAAGTTTGCTGAACTGCATGATACTTCCTTAAGAATGGCTGCCAAGGGTGTAATTAGGGAAGTTCTGGACTGGCGTAACTCCCGGGCTGTCTTCTACCAGAGACTTCACCGAAGAATTGGAGAGCAGTCACTGATCAACAGTGTGAGAGATGCTGCG‑‑‑GGTGATCATTTGTCACATGCATCTGCAATGAACTTGCTCAAAGAATGGTATTTGAATTCTGATATTGCCAATGGTAGAGAAGATGCATGGTTGGATGATGAAGCCTTCTTCAAATGGAAAAATAATCCTGCACATTACGAGAATAAACTAAAGGAACTGCGTGTGCAGAAAGTGTTACTTCAATTGACAAACATTGGTGACTCAGCTCTAGATTTGCAAGCTCTACCTCAGGGTCTTGCTGCCCTTTTAAGCAACTTGGAGCCTTCGGGTCGTGGGAAGTTGACTGATGAACTTCGAAAGGTACTTGGT‑‑‑

>Vra_ACC_Vradi05g07690

ATGGCTGACATTGGGCGTCGGAAT‑‑‑‑‑‑‑‑‑GGATATGCAAATAGTGTACAGCCAAATAGGCACCCTGCTGCAATATCCGAAGTAGATGAATTCTGCAATGCCCTTGGCGGAAACAGGCCAATTCATAGCATTTTAATTGCAAACAATGGAATGGCTGCAGTCAAATTTATACGTAGTGTTAGGAGCTGGGCCAATGAGACGTTTGGAACAGAAAAAGCCATCTTGTTGGTTGCCATGGCTACTCCAGAGGACATGAGAATCAATGCCGAACATATCAGAATAGCAGATCAATTTGTGGAAGTACCTGGTGGGACCAATAACAATAACTATGCCAATGTGCAGCTTATTCTTGAG‑‑‑‑‑‑‑‑‑ATGGCTGAGATAACTCATGTTGATGCCGTGTGGCCTGGTTGGGGTCATGCTTCAGAAAATCCCGAGCTTCCAGATGCATTAAAAGCAAAAGGAATTGTATTTCTCGGACCTCCTGCCATATCTATGGCTGCATTGGGAGATAAAATTGGTTCATCCTTGATTGCTCAAGCAGCAGAAGTGCCAACCCTTCGATGGAGTGGCTCTCATGTGAAAATTCCTCCTGAAAGTTCCTTGGTTACTATTCCTGATGAAATTTATCGGGAAGCGTGTGTGTATACAACAGAGGAAGCAGTAGCTAGTTGTCAAGTTGTAGGATACCCTGCAATGATCAAAGCATCTTGGGGTGGTGGTGGTAAAGGCATAAGAAAGGTTCATAATGATGATGAGGTAAGGGCATTGTTCAAGCAAGTCCAAGGTGAAGTTCCGGGTTCACCTATATTTATAATGAAGGTTGCATCCCAGAGTCGACATCTAGAAGTCCAGTTACTTTGTGATCAGCATGGAAATGTTGCAGCTTTGCATAGTCGTGATTGCAGTGTTCAAAGGAGGCACCAAAAGATTATTGAGGAGGGTCCCATTACGGTAGCACCCATAGAAACAGTGAAAAAACTGGAGCAAGCAGCTAGAAGGTTGGCTAAATCAGTAAATTATGTCGGGGCGGCTACTGTTGAGTATCTTTACAGTATGGAAACTGGAGAGTACTACTTTTTAGAGTTGAACCCTCGGCTACAGGTTGAACATCCTGTTACTGAGTGGATAGCAGAGATAAATCTGCCTGCAGCCCAAGTTGCCGTTGGAATGGGTGTCCCCCTCTGGCAAATTCCTGAGATAAGGCGTTTCTATGGGGTGGAGCATGGTGGGGGGTATGATGCTTGGAGAAAAACTTCAGCTTTAGCTACCCCTTTTGATTTTGATAAAGCACAATCCACAAAGCCAAAAGGTCATTGTGTGGCTGTTCGAGTGACTAGTGAGGACCCCGATGATGGCTTTAAGCCTACGAGTGGCAAAGTGCAGGAGCTTAGCTTTAAAAGCAAGCCAAATGTGTGGGCATACTTCTCTGTCAAGTCTGGAGGAGGAATTCATGAATTCTCAGATTCACAGTTTGGGCATGTGTTTGCTTTTGGAGAATCTAGGGCTTTAGCAATTGCAAATATGGTTCTAGGGCTGAAGGAGATTCAAATTCGGGGAGAAATCCGTACCAATGTTGATTACACCATTGATCTTCTGAATGCTTCTGACTACAGAGACAACAAAATTCACACAGGTTGGCTAGACAGTAGAATTGCAATGCGTGTTAGAGCAGAGAGGCCTCCCTGGTATCTTTCTGTTGTTGCAGGGGCACTCTATAAAGCTTCTGCTAGCAGTGCAGCTTTGGTTTCAGACTATGTTGGCTATCTTGAAAAGGGACAAATCCCTCCCAAGCACATATCTCTTGTCCATTCTCAAGTGGCCTTGAACATTGAGGGAAGCAAATATACAATTGACATGATACGAGGTGGATCTGGAAGTTATAGATTGAGAATGAATCAATCAGAGATAGAAGCAGAAATACACACTTTACGTGATGGAGGTTTGCTAATGCAG‑‑‑‑‑‑‑‑‑CTGGATGGAAACAGTCATGTAATATATGCAGAGGAAGAAGCAGCTGGAACTCGCCTTCTAATAGATGGAAGAACTTGCTTGCTTCAGAATGATCATGATCCATCAAAGTTGGTGGCGGAGACACCATGCAAGCTTTTGAGATATTTGGTCGGGGATGACAGTCATGTTGATGCTGACACACCATATGCTGAAGTTGAGGTCATGAAAATGTGCATGCCTCTTCTTTCACCTGCTTCTGGAATTATTCATTTCAAAATGTCTGAAGGTCAAGCAATGCAGGCTGGTGAACTTATAGCAAGTCTTGACCTAGATGATCCTTCAGCTGTAAGAAAGGCAGAGCCCTTCACAGGGAGCTTCCCACTCCTGGGACCTCCTACTGCAATTTCTGGTAAAGTTCATCAGAAATGTGCAGCAAACTTAAATGCTGCAAGGATGATTCTTGCTGGGTATGAGCACAATATTGATGAAGTTGTGCAAAGTTTGCTTAATTGTCTTGATAGTCCTGAGTTGCCTTTCCTTCAATGGCAAGAGTGCTTGGCTGTTCTGGCAACGCGTCTTCCCAAAGATCTAAAAAATGAGCTGGAGTCAAAATATAAGGAGTTTGAGAGTATTTCA‑‑‑AGCTCCCAAATTGTTGATTTCCCTGCCAAATTATTGAAGGGAATTCTTGAAGCTCATCTTTCTTCCTGTCCCGAGAAAGAAAAAGGAGCCCAAGAAAGACTAGTTGAACCTCTTCTGAGTCTTGTTAAGTCTTATGAGGGTGGACGAGAGAGCCATGCCCATATAATTGTTCAATCCCTTTTTGAAGAGTATCTTTCTGTTGAGGAATTATTTAGCGATAATATTCAGGCTGATGTAATTGAACGTCTGCGTCTACAATACAAGAAGGATTTATTGAAGATTGTTGATATTGTGCTCTCTCATCAGGGCATCAAAAGTAAAAATAAGCTGATATTGCGTCTAATGGATAAATTGGTTTACCCTAATCCTGCTGCTTATAGGGATCAGTTAATCCGTTTTTCTTTACTCAACCATACAAGTTATTCTGAGTTGGCACTAAAGGCAAGTCAACTGCTGGAACAAACTAAATTAAGTGAACTTCGGTCCCACATTGCTAGAAGTCTCTCTGAACTAGAGATGTTTACAGAGGATGGTGAAAATATTGATACTCCCAAGAGGAAGAGTGCCATCAACGACCGAATGGAGGACCTTGTCAGTGCTCCTTTGGCTGTTGAAGATGCCCTTGTGGGTTTATTTGATCACAGTGATCACACCCTTCAGAGGAGGGTCGTTGAAACTTATATTCGCAGACTCTACCAGCCTTATCTTGTCAAAGGGAGTGTTAGGATACAATGGCACAGATCTGGTCTTATTGCCACGTGGGAGTTCTATGATGAGTACATTGAAAGGAAGAAT‑‑‑GGGGTTGAAGAC‑‑‑CAAACTTCGAAGAAATCAGTGGAGGAGAAAGACAGTGAGAAAAAGTGGGGAGTGATGGTTGTAATTAAATCTCTTCAATTTTTGTCTGCAATTATCAGTGCTGCATTGAGGGAGGCGACCAATAACCTTCATGAAGCA‑‑‑CTGACCAGTGGTTCTGCGGAACCAGTTAACCATGGTAATATGATGCATATTGGATTAGTGGGCATCAACAACCAGATGAGTTTATTGCAAGACAGCGGTGATGAGGATCAGGCTCAAGAAAGAATCAATAAATTAGCTAAAATACTCAAAGAGCAGGAAGTAGGCTCCACCATTAGAGCTGCAGGTGTCGGTGTAATCAGTTGTATCATCCAAAGGGATGAAGGACGTACCCCAATGAGGCACTCTTTTCACTGGTCAGAAGAAAAGCTGTACTATGCAGAGGAACCTCTATTGCGTCATCTGGAACCTCCTCTATCCATTTATCTTGAATTGGACAAACTTAAGGGCTATGAGAATATACGGTATACTCCATCTCGAGATCGTCAATGGCACCTCTACACGGTTATGGATAACAAGCCACAACCAATTCAAAGAATGTTTCTTCGCACGCTTCTAAGACAGCCAACCACAAATGAAGGATTTTCTTCATATCAAAGGCTAGATGCAGAAACATCTCGAATCCAATTGGCTATGTCCTTTACTTCAAGGAGCATTTTTAGGTCCTTGATGGCTGCAATGGAGGAGTTGGAACTTAATGCACACAACGCCAATATAAAACCTGAGCATGCTCATATGTACCTCTATATCATACGGGAGCAACAAATAGACGATCTTGTTCCCTATCCCAAGAGAATTAATGTAGATGCTGGGAAAGAAGAAACAACAGTTGAGGCAGTCTTGGAAGAACTGGCACAGGAGATCCATTCATCCGTTGGTGTAAGAATGCATAGATTAGGGGTTGTTGTGTGGGAAGTCAAGCTCTGGATGGCAGCCGTTGGACAGGCAAATGGTGCTTGGAGGGTCATTGTGAACAATGTGACTGGTCATACATACACTGTACATATATACCGAGAAAAGGAGGATACCAGCACTCATAAAGTGGTATACAGTTCAGTCTCGGTTAAGGGTCCACTGCATGGTGTGCCAGTGAACGAAAATTATCAACCTTTGGGAGTTATTGACCGAAAACGTCTTTCAGCAAGAAAGAACAGCACCACATATTGCTATGATTTTCCACTTGCATTTGAAACAGCTTTGGAACAGTCATGGGCAATCCAACAGCCGGGATTTCAAAGAGCCAAAGATAAAAGTCTTCTAAAGGTAACGGAGCTTAAATTTGCAGAGAGAGAAGGTAGCTGGGGTACTCCTCTTGTTCCTGTGGAGCGTTACCCTGGACTCAATGATGTTGGTATGGTAGCCTGGTTTATGGATATGCGTACCCCTGAGTTCCCATCTGGAAGAACAATATTGGTTGTTTCAAATGATGTGACCTTCAAGGCTGGTTCTTTTGGACCGAGAGAGGATGCTTTCTTCCGTGCAGTAACTGATCTTGCATGTAAAAGAAAACTACCTTTAATTTATTTAGCAGCAAATTCTGGTGCCCGTTTAGGTGTAGCAGAGGAAGTCAAATCCTGTTTCAGAGTTGGTTGGTCTGAGGAATCTAGTCCTGAGCATGGCTTTCAGTATGTATATTTAACACCTGAGGACTATGCTCGGATTGGATCTTCAGTCATAGCACATGAATTAAAGCTTGAAAGTGGAGAAACCAGATGGGTTATAGATACCATTGTTGGCAAGGAGGATGGCCTTGGGGTTGAAAACTTGAGTGGCAGTGGGGCTATTGCTGGTGCCTATTCCAGGGCATACAAGGAAACTTTTACATTGACATATGTGACTGGCAGAACTGTTGGAATAGGTGCTTATCTTGCTAGGCTTGGAATGAGATGCATACAGAGGCTTGATCAGCCCATAATTCTTACTGGTTTTTCAGCACTAAACAAACTTCTTGGTCGGGAAGTATACAGTTCTCACATGCAACTTGGTGGACCCAAAATCATGGCTACAAATGGAGTTGTTCATCTTACAGTTTCCGATGATCTTGAAGGTGTTTATTCTATTTTGAAGTGGCTTAGCTACATTCCTTCTCATATAGGTGGTGCGCTTCCCATTGTTAAGCCTCTTGATCCTCCAGAAAGGCCAGTGGAGTATTTACCAGAAAACTCGTGTGATCCTCGTGCTGCCATTTCTGGAACCCTGGATGGTAATGGAAGATGGCTGGGAGGCATCTTTGACAAGGACAGCTTTGTGGAGACACTGGAGGGATGGGCTAGAACAGTTGTTACAGGAAGGGCAAAGCTTGGAGGAATCCCTGTGGGAATTGTTGCTGTAGAAACACAGACAGTGATGCAAATAATACCTGCTGATCCTGGTCAGCTTGATTCTCATGAGAGGGTCGTTCCTCAAGCTGGGCAAGTCTGGTTTCCTGATTCTGCAACCAAAACAGCCCAAGCAATATTGGATTTCAATAGAGAAGAGCTCCCGCTTTTCATTCTCGCAAACTGGAGAGGCTTTTCAGGAGGGCAAAGGGACCTTTTTGAAGGAATTCTTCAGGCTGGTTCAACTATTGTGGAGAACCTTAGAACATACAAGCAACCCATATTTGTGTACATCCCAATGATGGGGGAACTCCGAGGTGGTGCATGGGTGGTTGTTGACAGTCAAATCAATTCAGACCATATTGAAATGTATGCTGATCGAACAGCTAAAGGTAATGTCCTTGAGCCGGAAGGAATGATTGAGATCAAATTCAGAACAAGGGAATTGTTAGAGTGTATGGGTAGACTGGATCAACAGTTGATAACTCTGAAGGCAAAACTTCAGGAAGCCAAGAGTAACAGGGACCTTGCCGCCTTTGAATCCCTACAGCAGCAGATTAAATCCCGTGAAAAACAGCTTTTGCCTGTGTATACCCAGATAGCCACAAAGTTTGCTGAACTACATGATACTTCCTTAAGAATGGCTGCCAAGGGTGTAATTAGAGAAGTTCTGGACTGGCGTAACTCCCGAGCTGTCTTCTACCAGAGACTTCACCGAAGAATTGGAGAGCAGTCACTGATCAACAGTGTGAGAGATGCTGCT‑‑‑GGTGACCATTTGTCACATGCATCTGCAATGAACTTGCTCAAAGAATGGTATTTGAATTCTGATATTGCCAATAGTAAAGAAGATGCGTGGTTGGATGATGAAGCCTTCTTCAAATGGAAGAATAATCCTGCAAATTACGAGAATAAACTAAAGGAACTGCGTGTGCAGAAAGTGTTGCTTCAATTGACAAACATTGGTGACTCAGCCCTAGATTTGCAAGCTCTACCTCAAGGTCTTGCAGCCCTTTTAAGCAAGTTGGAGCCATCGGGTCGTGGGAAGTTGACTGATGAACTTCGAAAGGTACTTGGT‑‑‑

# Table S12. FASTA alignment of *accA* sequences used for phylogenetic inference

>Aar_accA_ZCDJ‑2005513

‑‑‑‑‑‑ATGGCTTCTGCATCACATGGTGCGTTTGCGTTTGCTGGG‑‑‑‑‑‑‑‑‑ACTTCTGCTTCGGATCTTCTCCGAAGTTCGACAAACGGTGTTTCTGGTGTCACTTTAAACACTTTAGGACGTCTGTCT‑‑‑CTTAAACTGAAGAGA‑‑‑AGGGATTTTACAGTTTCT‑‑‑‑‑‑GCGAAGATGAAGGGGAAGAAGAAGTACGAACGTCTCTGGCCCATTAACCCGGACCCCAATGTGAAGGGGGGA‑‑‑‑‑‑GTGCTAACTCATCTTTCTCATTTCAAGCCACTGCAAGAGAAGCCAAAG‑‑‑CCAGTCATACTGGATTTTGAAAAACCACTTGTTGAACTGGAAAAGAAGATCGACGATGTGCGGAAGATGGCACAAGATACTGGCCTAGACTTCAATGACCAGATTATCTCATTAGAGACTAAGTACCAAAAGGCTTTAAAAGATTTATATACTCATCTGAAACCTATTCAGCGGGTCAACATTGCCCGGCATCCTCACAGACCTACTTTCCTTGATCATATCTTTAACATAACTGAAAAGTTTGTTGAACTCCATGGTGATCGGGCAGGTTATGATGATCCTGCTATTGTTACTGGTATAGGGACCATAGATGGTAGAAGATACATGTTCATAGGTCATCAAAAGGGTAGAAACTCCAAAGAAAATATTCAGCGGAATTTTGGAATGCCGACTCCCCATGGTTACAGAAAGGCTCTTCGCATGATGTATTATGCAGAACACCATGGGTTTCCCATAGTCACTTTCATAGATACTCCTGGAGCTTTTGCTGACCTTAAATCAGAGGAACTAAACCAAGGTGAAGCCATTGCTCACAATTTGAGGACCATGTTTGGACTGAAGGTGCCGATTGTGTCTATAGTTCTTGGGGAAGGCGGTTCTGGTGGTGCCCTTGCCATTAGCTGTGCTAATAAATTACTTATGCTTGAAAATGCAGTTTTCTACGTGGCCAGTCCAGAGGCGGCTGCAGCAATCTTGTGGAAGAGTGCTAAAGAATCTCCAAAGGCTGCGGCGCGAATGAAAATAACAGCCAGAGCGTTGTGCAAATTGAAAGTTGCAGATGGTTTTATTCGTGAGCCGCTGGGTGGTGCACATAAGGATCCATCATGGGCATCTGAAGAGATAAAATCTGCAATCAATAAAGCCATGAATGAGCTCACTAAGATGGGAACAGAACAACTAGTAAAGCATCGTATGCGCAAGTTCCGAAAACTTGGTGGGTTCCAGGAAGGAGTTCCTATTGATCCTAAGAAGAAATTCAACATGAAGAAGAGGGAACTACCCGATACTGAGATAATTCCTGAT‑‑‑‑‑‑‑‑‑GCTGAATTAGAGGGTGAGGTTGAGAAACTGAAGGAGCAAATTTTGAAAGGCAAAACATCCACA‑‑‑‑‑‑ATCAAGCCTCCAAAGTCAGATGTGAAAACGATGATACAG‑‑‑‑‑‑‑‑‑‑‑‑AAATTAAAAGATGAGGTTGATCAAGAATACTCAGAGGCGGTGAAAGCTATGGGCTTGATGGACAAGTTGGTGGAGCTACGGGCGGAAGTTTCAAAAGCAAATTCAGAA‑‑‑‑‑‑‑‑‑GAC‑‑‑CAACTTATTGATCCGATTCTGATGGATAAGATTGAAAAACTAAAGGAGGAGTTCAATCAGGGATTATCAGCAGCTCCCAATTATGAGAGGCTTCAGAATAAGCTTGGCAAGTTGAAAGACTTGTCTGAAGAAAAATTTCAGGCAGATAAAGAAAAG‑‑‑‑‑‑AAGGCTGTCAAATGG‑‑‑AAGCGAGAATTCATGAATAAATTAAACAATATTATAAACAGCCCCAATATAAAGGAAAAATATGAAACGTTGAAGGCTGAAGTTGAAGGTTATGGTATATCCTCGCCAAGGGATTTGGATGAAAAGTTAAAAGAGAAAATCATTGAGCTTAAGAAAGAGATTGAGTCGCAGCTTGTTGATGCTTTGATGTCAAAGGGCTTAGAGGTTGATATTGTTCAAGCAAAGGCACAGGACAATATTGGGGAG‑‑‑‑‑‑TCATCA‑‑‑‑‑‑‑‑‑‑‑‑‑‑‑‑‑‑‑‑‑CAGCCATTGTTAAAAGAACTAAACACAGAC‑‑‑ATCTACAAGGGAATTGAAAATGCCCTAAGCTCACCAGGT‑‑‑CTTAAGAGCAACATAGAGCTACTGAACATGGAGGTTGCCAAAGCAGCAAACAATCCGGAT‑‑‑TCGGCCTTGAAAAATAGAATTGTTGCTTTGCAGCAACAAATAAAGCAAAGCCTT‑‑‑‑‑‑‑‑‑‑‑‑GCAGAGACCATTTCTACTTCCAGCTTAATAGAGAAGTTTGAGAATATAACATCTGAGACTTCCGGCATGAATGATTTT‑‑‑TCTGGAGAATCAGAT‑‑‑‑‑‑‑‑‑‑‑‑‑‑‑GAAGATCCTGCAGGAGACAGTCTTACCCATGATGAGTTGAGAGAGAGAGTTGGTGCAAACCGCACTTCTTCT‑‑‑‑‑‑‑‑‑‑‑‑‑‑‑‑‑‑

>Adu_accA1_Aradu.14CMN

‑‑‑‑‑‑ATGGCTTCTTCCTCTGCTGCT‑‑‑GCATCTCTTGCCGGC‑‑‑‑‑‑‑‑‑GGTTCGGCCTCGGATCTGCTGAGGGGTTCAACAAGTGGATTCAGCGGTGTCCCTTTGAGGACTTTAGGGAGGGCAAGG‑‑‑TTGCCCTTGAAGCAA‑‑‑AGGGACTTCTCAGTGTCT‑‑‑‑‑‑TGTAAGATGAGGAAGGTGAAGAAGCATGAGCACCCTTGGCCTGATAACCCTGATCCCAATGTGAAAGGCGGG‑‑‑‑‑‑GTGCTGAGCCACCTCTCACCTTTCAAACCACTCAAGGAGAAGCCGAAA‑‑‑CCGGTCACTTTGGATTTCGAGAAGCCCCTTATTGCTCTGCAAAAGAAGATCATAGATGTGCGGAAGATGGCAAACGAAACTGGTTTGGATTTTAGTGATCAGATTCTCTCATTGGAAACCAAGTACCAGCAGGCTTTGAAGGATTTATACACGCATCTGACTCCTATTCAGCGGGTCAACATTGCACGGCATCCTAACAGGCCGACTTTCCTTGATCATATCTATAACATTACTGACAAGTTTGTGGAACTCCATGGTGATCGAGCAGGTTACGATGACCCTGCTATTGTTACTGGTATAGGGACTATAGATGGTAGAAGGTACATGTTCATTGGTCAGCAAAAGGGTAGAAATACCAAAGAAAACATTCAGCGGAACTTTGGGATGCCAACTCCACATGGTTATAGGAAGGCTCTTCGCTTAATGGAATATGCAGATCACCATGGGTTTCCAATTGTTACTTTCATTGACACGCCTGGGGCATATGCTGACCTTAAATCAGAGGAACTGGGTCAAGGTGAAGCGATTGCTCATAATCTGAGATCCATGTTTGGCCTGAAAGTTCCAGTTATATCCATAGTTATTGGAGAAGGTGGTTCTGGTGGTGCCCTTGCTATTGGATGTGGCAACAAATTACTTATGCTTGAAAATGCAGTTTTCTATGTTGCCAGTCCTGAGGCATGTGCAGCAATCTTGTGGAAGAGTGCTAAAGCAGCACCAAAGGCTGCTGAGAAACTGAGGATCACAGCCAGTGAATTGTGCCGATTGGAAATTGCAGATGGGGTTATCCCTGAGCCACTTGGTGGTGCACATGCAGATCCATCTTGGACCTCGCAACAGATAAAAAAAGCAGTCAATGAAGCCATGGATGAGCTCACCAAGATGAATACCGAAGAGCTACTACGACATCGCATGCTTAAGTTCCGCAAGATTGGTGGGTTCCAGGAAGGAATTCCTGTAGAACCTAAGAAGAAAATCAACATGAAGAAGAAAGATATACCAATTGCCAATAAGATTTCGGAT‑‑‑‑‑‑‑‑‑GCCGAATTAGAGGTTGAGGTTGAGAAACTGAAGCAGCAAATCTTGGATTCTAAGGAATCCTCT‑‑‑‑‑‑‑‑‑ATTGAGCCGAGATTAGATCTGGATGATATGATAAAG‑‑‑‑‑‑‑‑‑‑‑‑CAACTGCAAATAGAGGTTGACCAAGAGTACTCTGAGGCAGTTAATGCCATTGGCTTGTCTGACAGGATGTCAAAACTAAAGGAGGAGGTTGTGAAAGCAAACACAGAT‑‑‑‑‑‑‑‑‑AAC‑‑‑CAATTTATTGATCCGTTGCTGAAGAGTAAGATAGAAAAGCTAAAGGAGGAGTTTGATCAGAAATTGTCCACGGCACCCAACTTTGGCAGGCTGGAGAATAAGGTTAACATGTTGAAAGAATTGTCTAAGGTAAAGCGTTTGCAAGATCAGAACAAG‑‑‑‑‑‑CGAACTTCTGCACTG‑‑‑GAGCAAGAATTGAAGACAAAATTCGATGGCATCATGAAGAATCCAAGAATAAAGGAAAAGTACGAGGCATTGAAGTCTGAAATTCAAGCTGCCGGGGCATCCTCCTCTAGGGATTTGGATGATGATCTGAAGCAGAAAATTGTGGAGTTTAATAAAGAATTTGACTCGCTGCTGGCTGAGTCTTTGAAGTCGGCCGGCATGGAAGTTAAGATTGCTCCGGCAAGGCCACGAGACAGCTCAGGGGAG‑‑‑‑‑‑TCTGCAGAGTTAGGA‑‑‑‑‑‑‑‑‑TATGAATCGAAGATAGAAGAATTAAGAGAGGGT‑‑‑ATAAGTAAGGAAATCGAGAAGTTGGCGAACTCATCCAAC‑‑‑ATTAAGAGCAAGATTGAACTATTGAAATTGGAGGTTGCTAAGGCTGGAGAGACACCTGAT‑‑‑ACAGAATCAAAAAATAGAATTGCTGCTTTGGTGCAACAAATTAAGCAGAGCCTT‑‑‑‑‑‑‑‑‑‑‑‑GAAGAGGCTGTTGACTCGTCTAGCTTGAAAGAGGAGTATGAAAATCTGGTTTCTAAGATTTCCAGCAGA‑‑‑‑‑‑‑‑‑‑‑‑‑‑‑‑‑‑‑‑‑‑‑‑GAT‑‑‑‑‑‑‑‑‑‑‑‑‑‑‑‑‑‑TCGGAAGTGGAAGATGGTCTCACCAATGATCAGTTGCGAGAGAAAGTTGGTGCAAACCGCAGCTTTTCT‑‑‑‑‑‑‑‑‑‑‑‑‑‑‑‑‑‑

>Aip_accA1_Araip.7H2NS

‑‑‑‑‑‑ATGGCTTCTTCCTCTGCTGCT‑‑‑GCATCTCTTGCCGGC‑‑‑‑‑‑‑‑‑GGTTCGGCCTCGGATCTGCTGAGGAGTTCAACAAGTGGATTCAGCGGTGTCCCTTTGAGGACTTTAGGGAGGGCAAGG‑‑‑TTGCCCTTGAAGCAA‑‑‑AGAGACTTCTCAGTGTCT‑‑‑‑‑‑TGTAAGATGAGGAAGGTGAAGAAGCATGAACACCCTTGGCCTGATAACCCTGATCCCAATGTGAAAGGCGGG‑‑‑‑‑‑GTGCTGAGCCACCTCTCACCTTTCAAACCACTCAAGGAGAAGCCGAAA‑‑‑CCGGTCACTTTGGATTTCGAGAAGCCCCTTATTGCGCTGCAAAAGAAGATCATAGATGTGCGGAAGATGGCAAACGAAACTGGTTTGGATTTTAGTGATCAGATTCTCTCATTGGAAACCAAGTACCAGCAGGCTTTGAAGGATCTATACACGCATCTGACTCCTATTCAGCGGGTCAACATTGCACGGCATCCTAACAGGCCGACTTTCCTTGATCATATCTATAACATTACTGACAAGTTTGTGGAACTCCATGGTGATCGAGCAGGTTACGATGACCCTGCTATTGTTACTGGTATAGGGACTATAGATGGTAGAAGGTACATGTTCATTGGTCAGCAAAAGGGTAGAAATACCAAAGAAAACATTCAGCGTAACTTTGGGATGCCAACTCCACATGGTTATAGGAAGGCTCTTCGCTTGATGGAATATGCAGATCACCATGGGTTTCCAATTGTTACTTTCATTGACACGCCTGGGGCATATGCTGACCTTAAATCAGAGGAACTGGGTCAAGGTGAAGCGATTGCTCATAATCTGAGATCCATGTTTGGCCTGAAAGTTCCAGTTATATCCATAGTTATTGGAGAAGGTGGTTCTGGTGGTGCCCTTGCTATTGGATGTGGCAACAAATTACTTATGCTTGAAAATGCAGTTTTCTATGTTGCCAGTCCTGAGGCATGTGCAGCAATCTTGTGGAAGAGTGCTAAAGCAGCACCAAAGGCTGCTGAGAAACTGAGGATCACAGCCAGTGAATTGTGCCGATTGGAAATTGCAGATGGGGTTATCCCTGAGCCACTTGGTGGTGCACATGCAGATCCATCTTGGACCTCGCAACAGATAAAAAAAGCAGTCAATGAAGCCATGGATGAGCTCACCAAGTTGAATACCGAAGAGCTACTACGACATCGCATGCTTAAGTTCCGCAAGATTGGTGGGTTCCAGGAAGGAATTCCTGTAGAACCCAAGAAGAAAATCAACATGAAGAAGAAAGATATACCAATTGCCAATAAGATTTCGGAT‑‑‑‑‑‑‑‑‑GCTGAATTAGAGGTTGAGGTTGAGAAGCTGAAGCAGCAAATCTTGGATTCTAAGGAATCCTCT‑‑‑‑‑‑‑‑‑ATTGAGCCGAGATTAGATCTGGATGATATGATAAAA‑‑‑‑‑‑‑‑‑‑‑‑CAACTGCAAATAGAGGTTGACCAAGAGTACTCTGAGGCAGTTAACGCCATTGGCTTGTCTGACAGGATGTCAAAACTAAAGGAGGAGGTTGTGAAAGCAAACACAGAT‑‑‑‑‑‑‑‑‑AAC‑‑‑CAATTTGTTGATCCGTTGCTGAAGAGTAAGATAGAAAAGCTAAAGGAGGAGTTTGATCAGAAATTGTCCACGGCACCCAACTTTGGCAGGCTGGAGAATAAGGTTAACATGTTGAAAGAATTGTCTAAGGTAAAGCGTTTGCAAGATCAGAACAAG‑‑‑‑‑‑CGAACTTCCGCATTG‑‑‑GAACAAGAATTGAAGACAAAATTCGATGGCATCATGAAGAATCCAAGAATAAAGGAAAAGTACGAGGCATTGAAGTCTGAAATTCAAGCTGCCGGGGCATCCTCCTCTAGGGATTTGGATGATGATCTGAAGCAGAAAATCGTCGAGTTTAATAAAGAATTTGACTCGCTGCTGGCTGAGTCTTTGAAGTCGGCCGGCATGGAAGTTAAGATTGCACCAGCAAGGCCACGAGACAGCTCAGAGGAG‑‑‑‑‑‑TCTGCAGTGTTAGGA‑‑‑‑‑‑‑‑‑TATGAATCGAAGATAGAAGAATTAAGAGAGGGT‑‑‑ATAAGTAAGGAAATCGAGAAGTTGGCAAACTCATCCAAC‑‑‑ATTAAGAGCAAGATTGAACTATTGAAATTGGAGGTTGCTAAGGCTGGAGAGACACCTGAT‑‑‑ACAGAATCAAAGAATAGAATTGCTGCTTTGGTGCAACAAATTAAGCAGAGCCTT‑‑‑‑‑‑‑‑‑‑‑‑GAAGAGGCTGTTGACTCGTCTAGCTTGAAAGAGGAGTATGAAAATCTGGTTTCTAAGATTTCCAGCAGA‑‑‑‑‑‑‑‑‑‑‑‑‑‑‑‑‑‑‑‑‑‑‑‑GAT‑‑‑‑‑‑‑‑‑‑‑‑‑‑‑‑‑‑TTGGAAACGGAAGATGGTCTCACCAATGATCAGTTGCGAGAGAAAGTTGGTGCAAACCGCAGCTTTTCT‑‑‑‑‑‑‑‑‑‑‑‑‑‑‑‑‑‑

>Gpo_accA_VLNB‑2004340

‑‑‑‑‑‑ATGGCTTCTACTTCT‑‑‑‑‑‑‑‑‑GCATCGCTTGCTGGT‑‑‑‑‑‑‑‑‑GTTTCGGCTTCGGATCTTCTCAAGAGTTCAACTAACGGTTTCAGCGGTGTTCCTTTGAGAACCTTGGGAAGGGCAAGG‑‑‑TTGGTTTTGAGAAAG‑‑‑AGGGATTTTACAGTTGCT‑‑‑‑‑‑GCTAAGCTGAGGAAGGTGAAGAAGAATGAATATCCCTGGCCTGATAACCCCGATCCCAATGTGAAAGGTGGG‑‑‑‑‑‑GTGCTGAGTCATCTCTCCCCTTTCAAGCCACTAAAAGAGAAGCCAAAG‑‑‑CCTGTCACTTTGGATTTCGAAAAGCCTCTTGTTGATCTGCAAAAGAAGATCATCGATGTGCGGAAGATGGCAAACGAAACGGGTCTGGACTTCACTGATCAGATTCTCTCATTGGAGTCTAAGTACCAGCAGGCTTTAAAGGATCTATATACGCATCTGACTCCTATACAGCGGGTCAGCATTGCACGGCATCCTAACAGGCCTACTTTCCTTGATCACATCTTTAACATAACTGAAAAGTTTGTGGAACTCCATGGTGATCGGGCAGGTTATGATGATCCTGCTATTGTTACGGGTATAGGGACTATAGATGGTAGAAGATACATGTTCATCGGTCACCAAAAGGGTAGAAATACTAAAGAAAACATTCAGCGTAACTTTGGGATGCCAACTCCACATGGTTACAGGAAGGCTCTTCGATTGATGGAGTATGCAGATCACCATGGGTTCCCAATAGTTACTTTCATTGACACGCCTGGGGCATATGCTGACCTCAAATCAGAGGAACTAGGCCAAGGTGAAGCGATTGCTCATAATTTGAGATCCATGTTTGGTCTGAAGGTGCCAATTGTTTCTATAGTTATTGGGGAAGGTGGTTCTGGTGGTGCCCTTGCCATTGGATGTGGTAATAAATTACTCATGCTTGAAAACGCAGTTTTCTATGTTGCAAGTCCAGAGGCATGTGCAGCAATCTTGTGGAAGAGTGCTAAAGCTTCTCCAAAGGCTGCTGAGAAACTGAAGATTACAGCCACTGAATTATGCAAATTGCAAATTGCAGACGGAATTATACCTGAGCCGCTCGGTGGTGCCCATGCAGATCAAAATTGGACCTCACAACAGATAAAAATTGCAATCAATGAAGCTATGGATGAGCTCACCAAGATGTCAACAGAAGACCTATTAAGACATCGCATGCTTAAATTCCGAAAGATTGGTGGGTTCCAGGAAGGAATTCCTATAGATCCTAAGAAGAAAGTCAACATGAAGAAGAAGGATATACCTGTTGCTAAGAAGATTCCTGAT‑‑‑‑‑‑‑‑‑GGTGAAATAGAGGTTGAGGTTGAGAAACTGAAGCAGAAAATTTTGGAAGCTAAGAAATCCTCT‑‑‑‑‑‑TCTGAGCCTCCAATGCTAGAGCTGGATGAGATGATAAAG‑‑‑‑‑‑‑‑‑‑‑‑AGACTGAAAAAGGAGGTTGATCAAGAATACTCCGAGGCAGTTAAAGCCATGGGCTTGACAGACAGGCTATTGAAACTAAAGGAGGAAGTTTCAAAAGCAATTTCAGAA‑‑‑‑‑‑‑‑‑AAC‑‑‑CAACTTATTGATCCATTCCTGAAGGATAAGATAGAAAGGCTAAAGGTGGAGTTTGACCAGGGTTTGTTAGCAGCTCCCAATTATGGTAGGCTGCAGAATAAGCTTGATTTTTTGAAAGAATTATCTAAAGTAAAACTTCTGTCAGATGCAAACAAG‑‑‑‑‑‑AGAGCTGACACATTT‑‑‑AAGCAAGAATTGAAGACAAAATTTGATGAAGTTGTGAATAATCCAAGAATAAAGGAAAACTATGAGGCATTAAAGGCTGAGATTCAAGCTGTTGGTGCATCCTCATCAAGTGATTTGGATGATGAGTTGAAGAAGAAAATAATTGAGTTTAAGAAAGAGGTAGACTTGCAGCTGGCTAATGCTCTGAAGTCGGCGGGCTTAGATGTTGAGCTTGTAAAAGCAAAGGGGCGAGAGGGCAAG‑‑‑GAG‑‑‑‑‑‑TCTTCAGTGTCAGAG‑‑‑‑‑‑‑‑‑TATGAGTCAAAGATAGAAGAAATAAACAAAGAT‑‑‑ATACAAAAGGAAATTGAAATTTCTGTGAACTCATCAGATATTATTAAGGGCAAGATAGAGATGTTGAAATTGGAGGTTGCCAAGGCTGGAGACACACCTGAT‑‑‑TTGGAATCAAAGAATAGAATTTCTGCTTTGGAGCAACAAATTAAGCAGAGCCTA‑‑‑‑‑‑‑‑‑‑‑‑GTAGAGGCCATTGACTCGCCTAGCTTAAAAGAGAAGTATGAAAATCTGGTGTCTAAGGTTTCCAGTGAATATGCATCT‑‑‑TCTGGGGAATTAGACGGAAATTTGAGAAACGAAAATGCAGCAGGAGACGATCCCACCAATGATGAGTTGAGAGTAAAAGTTGGTGCAAACCGCAACTGCCTGCTGCTCATATGGCGCTGG

>Lal_accA1_LAGI01_9432

‑‑‑ATGGCTGCTTCTTCTGCT‑‑‑‑‑‑‑‑‑GTGTATCTTACCGGG‑‑‑‑‑‑‑‑‑AATCCAGCTTCGGATCTTCTGAGGAATTCAACCAGTGGTTTCAGTGGTGTCCCTTTGAGAACCTTGGGAAGGGCAAGG‑‑‑TTGGTTTTGAAGACA‑‑‑AGGGATTTTACAGTTTCG‑‑‑‑‑‑TGTAAGTTGAGGAAGTTTAAGAAGCATGAGTATCCTTGGCCTGATAACCCTGATCCCAATGTGAAAGGTGGG‑‑‑‑‑‑GTGCTAAGTCATCTTTCTTCTTTCAAGCCACTCAATGAGAAGCCAAAG‑‑‑CCTGTGACTTTGGAGTTTGAAAAGCCTCTTCTTGATCTGCAAAGGAAGATCATTGATGTGCGGAAGATGGCGAACGAAACTGGTCTGGACTTTAGTGATCAGATTCTTTCATTGGAGTCTAAGTACCAGCAGGCTTTAAGGGATCTATATACACATCTGACTCCTATACAGCGGGTCAACATTGCACGGCATCCTAACAGGCCAACTTTCCTTGATCATATCTTTAACATAACGGAAAAGTTTGTTGAACTCCATGGTGATCGGGAAGGTTATGATGACCCTGCTATTGTTACTGGCATAGGGACTATAGATGGTAGAAGATACATGTTCATTGGTCAACAAAAAGGTAGAAATACTAAAGAAAATATTCAGCGTAACTTTGGGATGCCAACTCCTCATGGTTACAGGAAGGCTCTTCGCTTGATGGAGTATGCAGATCATCATGGATTCCCAATAGTTACTTTCATTGACACGCCTGGGGCATATGCTGACCTTAAATCGGAGGAACTGGGTCAGGGTGAAGCGATAGCCCATAATTTGAGATCCATGTTTGGTCTGAAGGTGCCAGTTCTGTCAATAGTTATTGGAGAAGGTGGTTCTGGTGGTGCACTTGCCATTGGGTGTGCTAATAAATTACTTATGCTTGAAAATGCCGTTTTCTATGTTGCTAGTCCGGAGGCATGTGCAGCAATCTTGTGGAAGAGTGCTAAAGCTGCTCCTAAGGCTGCTGAGAAACTGAAGATTACAGCCACCGAATTGTGCAAACTGGAAATTGCAGATGGTGTTATCCCTGAACCACTTGGCGGTGCACATGCAGATTCAGCTTGGACCTCACAACAGATAAAAATTGCAATCAATGAAACCATGGATGAACTTAGCAAACTTAGTACAGAAGAACTACTCAAACATCGCCATGATAAGTTTCGAAAGATTGGTGGATTCCAGGAAGGCATTCCTATTGATCCTAAGAGGAAGTTCAGCATGAAAAAGAAGGATATACCCATTCCCAAGAAGATTTCTGAT‑‑‑‑‑‑‑‑‑GCTGAAATAGAGGCTGAGATTCAGAAATTGAAGCAACAAATATTAGAAGCCAAGGGATCCTCT‑‑‑‑‑‑CCCGAGCCTCCAAAACATGATTTGGGTGATATGATAAAG‑‑‑‑‑‑‑‑‑‑‑‑AAACTGAAAAGGGAGGTTGATCACGAATACTTTGAGGCAGTTAAAGCCATGGGCTTGACAGACAGGTTCTCAAAACTAAGGGAGGAAGTTACAAAAGCAAATTCACGA‑‑‑‑‑‑‑‑‑AAC‑‑‑CAATCTATTGATCCATCGCTGAAGGATAAGATAGAAAAGTTAGAGGCGGAGTTTGATCAGGGTTTGCCAGCGGCTCCAAACTATGACAAGCTACAGAAGAAGCTTGGCATGTTGAAAGAGTTATCTGAAGTAAAGCATCTGTCAGAGACAAACGAG‑‑‑‑‑‑GAGGTTGTCAGATTG‑‑‑AAGCAAGAAATGCAGACAGTACTTGATGATGTCTTGAACAGTCCCAGAATAAAGGAAAAGTATGAGACATTAAAGGGTGAAATTGAAAGTATTGGTGCATCCTCACCAAGTGATTTGGATGATGAGTTGAAGAAGAAAATCATTGAGTTTAGAGAAGAGGTTGGCTTGCAGGTAGCTAATGATCTGAAGACAGCAGGCATAGATCTTACACCTTTAAGAGCAAAGCCACGAGAGAACAGCGACGAG‑‑‑‑‑‑TCTTCATTGTCAGAG‑‑‑‑‑‑‑‑‑TACGAGTCAAAGGTTGAAGAACTAAAAAAAGAT‑‑‑ATAGAAAAGGAAATTGAATATTCGGTGAACTCATCAGAT‑‑‑ATTAAGAGCAAGATAGAGGCATTGAATCTGGAAGTTGCCAAGGCTGCAGACACACCTGAT‑‑‑GTGGATTCAAAGAATAGAATTGCTGCTTTGGTGCAACAAATTAAGCAGAGCCTT‑‑‑‑‑‑‑‑‑‑‑‑GTTGAAACTCTTGACTCATCTAGCTTAAAAGACAAGTATGAAAATCTGGTGTCTAAAGTTTCCAGTGAA‑‑‑‑‑‑‑‑‑‑‑‑‑‑‑‑‑‑‑‑‑‑‑‑AATGGAAGTTTGAAAAAGGCAGATCCAACAGGCGAGAGTCCCAACAACGATGATTTGAAAGTGGAAGTTGCCACAAACCACACCTTTTCT‑‑‑‑‑‑‑‑‑‑‑‑‑‑‑‑‑‑

>Lal_accA5_LAGI01_11229

‑‑‑ATGGCTGCTTCTTCTGCT‑‑‑‑‑‑‑‑‑GTGTATCTTACCGGG‑‑‑‑‑‑‑‑‑AATCCAGCTTCGGATCTTCTGAGGAATTCAACCAGTGGTTTCAGTGGTGTCCCTTTGAGAACCTTGGGAAGGGCAAGG‑‑‑TTGGTTTTGAAGACA‑‑‑AGGGATTTTACAGTTTCG‑‑‑‑‑‑TGTAAGTTGAGGAAGTTTAAGAAGCATGAGTATCCTTGGCCTGATAACCCTGATCCCAATGTGAAAGGTGGG‑‑‑‑‑‑GTGCTAAGTCATCTATCATCTTTCAAGCCACTCAATGAGAAGCCAAAG‑‑‑CCTGTGACTTTGGAGTTTGAAAAGCCTCTTATTGATCTGCAAAGGAAGATCATCGATGTGCGGAAGATGGCGAACGAAACTGGTCTGGACTTTAGTGATCAGATTCTTTCATTGGAGTCTAAGTACCAGCAGGCTTTAAGGGATCTATATACACATCTGACTCCTATACAGCGGGTCAACATTGCACGGCATCCTAACAGGCCAACTTTCCTTGATCATATCTTTAACATAACGGAAAAGTTTGTTGAACTCCATGGTGATCGGGAAGGTTATGATGACCCTGCTATTGTTACTGGCATAGGGACTATAGATGGTAGAAGATACATGTTCATTGGTCAACAAAAAGGTAGAAATACTAAAGAAAATATTCAGCGTAACTTTGGGATGCCAACTCCTCATGGTTACAGGAAGGCTCTTCGCTTGATGGAGTATGCAGATCATCATGGATTCCCAATAGTTACTTTCATTGACACGCCTGGGGCATATGCTGACCTTAAATCGGAGGAACTGGGTCAGGGTGAAGCGATAGCCCATAATTTGAGATCCATGTTTGGTCTGAAGGTGCCAGTTCTGTCAATAGTTATTGGAGAAGGTGGCTCTGGTGGTGCACTTGCCATTGGGTGTGCGAATAAATTACTAATGCTTGAAAATGCAGTTTTCTATGTTGCCAGTCCAGAGGCATGTGCAGCAATCTTGTGGAAGAGTGCTAAAGCTGCTCCTAAGGCTGCTGAGAAACTGAAGATTACAGCCACCGAATTGTGCAAACTGGAAATTGCAGATGGTGTTATCCCTGAACCACTTGGCGGTGCACATGCAGATTCAGCTTGGACCTCACAACAGATAAAAATTGCAATCAATGAAACCATGGATGAACTTAGCAAACTTAGTACAGAAGAACTACTCAAACATCGCCATGATAAGTTTCGAAAGATTGGTGGATTCCAGGAAGGCATTCCTATTGATCCTAAGAGGAAGTTCAGCATGAAAAAGAAGGATATACCCATTCCCAAGAAGATTTCTGAT‑‑‑‑‑‑‑‑‑GCTGAAATAGAGGCTGAGATTCAGAAATTGAAGCAACAAATATTAGAAGCCAAGGGATCCTCT‑‑‑‑‑‑CCCGAGCCTCCAAAACATGATTTGGGTGATATGATAAAG‑‑‑‑‑‑‑‑‑‑‑‑AAACTGAAAAGGGAGGTTGATCACGAATACTTTGAGGCAGTTAAAGCCATGGGCTTGACAGACAGGTTCTCAAAACTAAGGGAGGAAGTTACAAAAGCAAATTCACGA‑‑‑‑‑‑‑‑‑AAC‑‑‑CAATCTATTGATCCATCGCTGAAGGATAAGATAGAAAAGTTAGAGGCGGAGTTTGATCAGGGTTTGCCAGCGGCTCCAAACTATGACAAGCTACAGAAGAAGCTTGGCATGTTGAAAGAGTTATCTGAAGTAAAGCATCTGTCAGAGACAAACGAG‑‑‑‑‑‑GAGGTTGTCAGATTG‑‑‑AAGCAAGAAATGCAGACAGTACTTGATGATGTCTTGAACAGTCCCAGAATAAAGGAAAAGTATGAGACATTAAAGGGTGAAATTGAAAGTATTGGTGCATCCTCACCAAGTGATTTGGATGATGAGTTGAAGAAGAAAATCATTGAGTTTAGAGAAGAGGTTGGCTTGCAGGTAGCTAATGATCTGAAGACAGCAGGCATAGATCTTACACCTTTAAGAGCAAAGCCACGAGAGAACAGCGACGAG‑‑‑‑‑‑TCTTCATTGTCAGAG‑‑‑‑‑‑‑‑‑TACGAGTCAAAGGTTGAAGAACTAAAAAAAGAT‑‑‑ATAGAAAAGGAAATTGAATATTCGGTGAACTCATCAGAT‑‑‑ATTAAGAGCAAGATAGAGGCATTGAATCTGGAAGTTGCCAAGGCTGCAGACACACCTGAT‑‑‑GTGGATTCAAAGAATAGAATTGCTGCTTTGGTGCAACAAATTAAGCAGAGCCTT‑‑‑‑‑‑‑‑‑‑‑‑GTTGAAACTCTTGACTCATCTAGCTTAAAAGACAAGTATGAAAATCTGGTGTCTAAAGTTTCCGGTGAA‑‑‑‑‑‑‑‑‑‑‑‑‑‑‑‑‑‑‑‑‑‑‑‑AATGGAAGTTCGAAAAAGGTAAATCCAACAGGAGACAGTCCCACCAATGACGAGTTGAAAGAGAAAGTTGGCACAAACCACACCTTTTCT‑‑‑‑‑‑‑‑‑‑‑‑‑‑‑‑‑‑

>Lal_accA4_LAGI01_9668

‑‑‑ATGGCTGCTTCTGCTGCT‑‑‑‑‑‑‑‑‑GCATCTCTTACTGGG‑‑‑‑‑‑‑‑‑GCTTCGGCTTCGGATCTTCTGAGGAGTTCAACCAGTGGTTTCAGTGGTGTCCCTTTGAGAACCTTAGGAAGGGCAAGG‑‑‑TTGGCTTTGAAGACT‑‑‑AGGGATTTTGCAGTTTCT‑‑‑‑‑‑TGTAAGTTGAGGAAGGTGAAGAAGCATGAGTATCCTTGGCCTGATAACCCTGATCCCAATGTGAAAGGTGGG‑‑‑‑‑‑GTCCTAAGTCATCTTTCTCCTTTCAAGCCACTCAAAGAGAAGCCAAAG‑‑‑CCTGTCACTTTGGAGTTTGAAAAGCCTCTTCTTGATCTGCAAAAGAAGATCATTGATGTACGAAAGATGGCGAACGAAACTGGTCTGGACTTTAGTGATCAGATTCTTTCATTGGAGTCTAAGTACCAGCAGGCTTTAAAGGATCTATATACACATCTGACTCCTATACAGCGAGTCAACATTGCACGACATCCTAACAGGCCAACTTTCCTTGATCATATCTTCAACATAACTGAAAAGTTTGTTGAACTCCATGGTGATCGGGAAGGTTATGATGATCCTGCCATTGTTACTGGCATAGGGACTATAGATGGTAGAAGATACATGTTCATTGGTCACCAAAAAGGTAGAAATACTAAAGAAAATATTCAGCGTAACTTTGGGATGCCAACTCCTCATGGTTACAGGAAGGCTCTTCGCTTGATGGAGTATGCAGATCATCATGGATTCCCAATAGTTACTTTCATTGACACGCCTGGGGCATATGCTGACCTTAAATCAGAGGAACTGGGTCAGGGTGAAGCGATTGCCCATAATTTGAGATCCATGTTTGGTCTAAAGGTGCCAGTTCTGTCAATAGTTATTGGAGAAGGTGGTTCTGGTGGTGCACTTGCCATTGCATGTGCTAATAAATTACTTATGCTTGAAAATGCCGTTTTCTATGTCGCTAGTCCGGAGGCATGTGCAGCAATCTTGTGGAAGAGTGCTAAAGCTGCTCCTAAGGCTGCTGAGAAACTGAAGATTACAGCCACCGAATTGTGCAAATTGGAAATTGCAGATGGTGTTATCCCTGAACCACTTGGCGGTGCACATGCAGATTCAGCTTGGACCTCACAACAGATAAAAAATGCAATCAATGAATCCATGGATGAGCTTAGCAAACTTAGTACAGAAGAACTACTCAAACATCGCCATGATAAGTTTCGAAAGATTGGTGGATTCCAGGAAGGCATTCCTATTGATCCTAAGAGGAAGTTCAGCATGAAAAAGAAGGATATACCCATTCCCAAGAAGATTTCTGAT‑‑‑‑‑‑‑‑‑GCTGAAATAGAGGCTGAGATTCAGAAATTGAAGCAACAAATATTAGAAGCCAAGGGATCCTCT‑‑‑‑‑‑CCCGAGCCTCCAAAACATGATTTGGCCGATATGATAAAG‑‑‑‑‑‑‑‑‑‑‑‑AAACTGAAAAGGGAGGTTGATCACGAATACTTTGAGGCAGTTAAAGCCATGGGCTTGGCAGACAGGCTCTCAAAACTAAGGGAGGAAGTTACAAAAGCAAATTCACGA‑‑‑‑‑‑‑‑‑AAC‑‑‑CAATCTATTGATCCATCGCTGAAGGATAAGATAGAAAAGTTAGAGGCGGAGTTTGATCAGGGTTTGCCAGCGGCTCCAAACTATGACAAGCTACAGAAGAAGCTTGGCATGTTGAAAGAGTTATCTGAAGTAAAGCATCTGTCAGAGACAAACGAG‑‑‑‑‑‑GAGGTTGTCAGATTG‑‑‑AAGCAAGAAATGCAGACAGTACTTGATGATGTCTTGAACAGTCCCAGAATAAAGGAAAAGTATGAGACATTAAAGGGTGAAATTGAAAGTATTGGTGCATCCTCACCAAGTGATTTGGATGATGAGTTGAAGAAGAAAATCATTGAGTTTAGAGAAGAGGTTGGCTTGCAGGTAGCTAATGATCTGAAGACAGCAGGCATAGATCTTACACCTTTAAGAGCAAAGCCACGAGAGAACAGCGACGAG‑‑‑‑‑‑TCTTCATTGTCAGAG‑‑‑‑‑‑‑‑‑TACGAGTCAAAGGTTGAAGAACTAAAAAAAGAT‑‑‑ATAGAAAAGGAAATTGAATATTCGGTGAACTCATCAGAT‑‑‑ATTAAGAGCAAGATAGAGGCATTGAATCTGGAAGTTGCCAAGGCTGCAGACACACCTGAT‑‑‑GTGGATTCAAAGAATAGAATTGCTGCTTTGGTGCAACAAATTAAGCAGAGCCTT‑‑‑‑‑‑‑‑‑‑‑‑GTTGAAACTCTTGACTCATCTAGCTTAAAAGACAAGTATGAAAATCTGGTGTCTAAAGTTTCCAGTGAA‑‑‑‑‑‑‑‑‑‑‑‑‑‑‑‑‑‑‑‑‑‑‑‑AATGGAAGTTTGAAAAAGGCAGATCCAACAGGCGAGAGTCCCAACAACGATGATTTGAAAGTGGAAGTTGCCACAAACCACACCTTTTCT‑‑‑‑‑‑‑‑‑‑‑‑‑‑‑‑‑‑

>Lal_accA3_LAGI01_9523

‑‑‑ATGGCTGCTTCTTCTGCT‑‑‑‑‑‑‑‑‑GTGTATCTTACCGGG‑‑‑‑‑‑‑‑‑AATCCAGCTTCGGATCTTCTGAGGAATTCAACCAGTGGTTTCAGTGGTGTCCCTTTGAGAACCTTGGGAAGGGCAAGG‑‑‑TTGGTTTTGAAGACA‑‑‑AGGGATTTTACAGTTTCG‑‑‑‑‑‑TGTAAGTTGAGGAAGTTTAAGAAGCATGAGTATCCTTGGCCTGATAACCCTGATCCCAATGTGAAAGGTGGG‑‑‑‑‑‑GTGCTAAGTCATCTATCATCTTTCAAGCCACTCAATGAGAAGCCAAAG‑‑‑CCTGTGACTTTGGAGTTTGAAAAGCCTCTTATTGATCTGCAAAGGAAGATCATCGATGTGCGGAAGATGGCGAACGAAACTGGTCTGGACTTTAGTGATCAGATTCTTTCATTGGAGTCTAAGTACCAGCAGGCTTTAAGGGATCTATATACACATCTGACTCCTATACAGCGGGTCAACATTGCACGGCATCCTAACAGGCCAACTTTCCTTGATCATATCTTTAACATAACGGAAAAGTTTGTTGAACTCCATGGTGATCGGGAAGGTTATGATGACCCTGCTATTGTTACTGGCATAGGGACTATAGATGGTAGAAGATACATGTTCATTGGTCAACAAAAAGGTAGAAATACTAAAGAAAATATTCAGCGTAACTTTGGGATGCCAACTCCTCATGGTTACAGGAAGGCTCTTCGCTTGATGGAGTATGCAGATCATCATGGATTCCCAATAGTTACTTTCATTGACACGCCTGGGGCATATGCTGACCTTAAATCGGAGGAACTGGGTCAGGGTGAAGCGATAGCCCATAATTTGAGATCCATGTTTGGTCTGAAGGTGCCAGTTCTGTCAATAGTTATTGGAGAAGGTGGCTCTGGTGGTGCACTTGCCATTGGGTGTGCGAATAAATTACTAATGCTTGAAAATGCAGTTTTCTATGTTGCCAGTCCAGAGGCATGTGCAGCAATCTTGTGGAAGAGTGCTAAAGCTGCTCCTAAGGCTGCTGAGAAACTGAAGATTACAGCCACCGAATTGTGCAAACTGGAAATTGCAGATGGTGTTATCCCTGAACCACTTGGCGGTGCACATGCAGATTCAGCTTGGACCTCACAACAGATAAAAATTGCAATCAATGAAACCATGGATGAACTTAGCAAACTTAGTACAGAAGAACTACTCAAACATCGCCATGATAAGTTTCGAAAGATTGGTGGATTCCAGGAAGGCATTCCTATTGATCCTAAGAGGAAGTTCAGCATGAAAAAGAAGGATATACCCATTCCCAAGAAGATTTCTGAT‑‑‑‑‑‑‑‑‑GCTGAAATAGAGGCTGAGATTCAGAAATTGAAGCAACAAATATTAGAAGCCAAGGGATCCTCT‑‑‑‑‑‑CCCGAGCCTCCAAAACATGATTTGGGTGATATGATAAAG‑‑‑‑‑‑‑‑‑‑‑‑AAACTGAAAAGGGAGGTTGATCACGAATACTTTGAGGCAGTTAAAGCCATGGGCTTGACAGACAGGTTCTCAAAACTAAGGGAGGAAGTTACAAAAGCAAATTCACGA‑‑‑‑‑‑‑‑‑AAC‑‑‑CAATCTATTGATCCATCGCTGAAGGATAAGATAGAAAAGTTAGAGGCGGAGTTTGATCAGGGTTTGCCAGCGGCTCCAAACTATGACAAGCTACAGAAGAAGCTTGGCATGTTGAAAGAGTTATCTGAAGTAAAGCATCTGTCAGAGACAAACGAG‑‑‑‑‑‑GAGGTTGTCAGATTG‑‑‑AAGCAAGAAATGCAGACAGTACTTGATGATGTCTTGAACAGTCCCAGAATAAAGGAAAAGTATGAGACATTAAAGGGTGAAATTGAAAGTATTGGTGCATCCTCACCAAGTGATTTGGATGATGAGCTGAAGCAGAAAATCGTCGAGTTTAGAAAAGAGGTTCACTTGCAGCTAGCTAATGCTCTGAAGTCAGCAGACTTAGATGTCAAGTTTGTAAGAGCTAAGCAACGGGATGACGGGGATGAG‑‑‑‑‑‑GGTTCATTGTCAGAG‑‑‑‑‑‑‑‑‑TATGAGTCAAAGTTTGAAGAACTGAACAAAGAT‑‑‑ATAGAAAAGGAAATCGAGTATTCGATA‑‑‑‑‑‑‑‑‑‑‑‑‑‑‑‑‑‑‑‑‑‑‑‑‑‑‑‑‑‑‑‑‑‑‑‑TTGAAATTGGAGGTTGCAAAGGCTGGAGACACACCTGAT‑‑‑GTGGATTCAAAGAATAGAATTGCTGCTTTGGTGCAACAAATTAAGCAGAGCCTT‑‑‑‑‑‑‑‑‑‑‑‑GTTGAAACTCTTGACTCATCTAGCTTAAAAGACAAGTATGAAAATCTGGTGTCTAAAGTTTCCAGTGAA‑‑‑‑‑‑‑‑‑‑‑‑‑‑‑‑‑‑‑‑‑‑‑‑AATGGAAGTTTGAAAAAGGCAGATCCAACAGGCGAGAGTCCCAACAACGATGATTTGAAAGTGGAAGTTGCCACAAACCACACCTTTTCT‑‑‑‑‑‑‑‑‑‑‑‑‑‑‑‑‑‑

>Lal_accA2_LAGI01_9477

‑‑‑ATGGCTGCTTCTTCTGCT‑‑‑‑‑‑‑‑‑GTGTATCTTACCGGG‑‑‑‑‑‑‑‑‑AATCCAGCTTCGGATCTTCTGAGGAATTCAACCAGTGGTTTCAGTGGTGTCCCTTTGAGAACCTTGGGAAGGGCAAGG‑‑‑TTGGTTTTGAAGACA‑‑‑AGGGATTTTACAGTTTCG‑‑‑‑‑‑TGTAAGTTGAGGAAGTTTAAGAAGCATGAGTATCCTTGGCCTGATAACCCTGATCCCAATGTGAAAGGTGGG‑‑‑‑‑‑GTGCTAAGTCATCTATCATCTTTCAAGCCACTCAATGAGAAGCCAAAG‑‑‑CCTGTGACTTTGGAGTTTGAAAAGCCTCTTATTGATCTGCAAAGGAAGATCATCGATGTGCGGAAGATGGCGAACGAAACTGGTCTGGACTTTAGTGATCAGATTCTTTCATTGGAGTCTAAGTACCAGCAGGCTTTAAGGGATCTATATACACATCTGACTCCTATACAGCGGGTCAACATTGCACGGCATCCTAACAGGCCAACTTTCCTTGATCATATCTTTAACATAACGGAAAAGTTTGTTGAACTCCATGGTGATCGGGAAGGTTATGATGACCCTGCTATTGTTACTGGCATAGGGACTATAGATGGTAGAAGATACATGTTCATTGGTCAACAAAAAGGTAGAAATACTAAAGAAAATATTCAGCGTAACTTTGGGATGCCAACTCCTCATGGTTACAGGAAGGCTCTTCGCTTGATGGAGTATGCAGATCATCATGGATTCCCAATAGTTACTTTCATTGACACGCCTGGGGCATATGCTGACCTTAAATCGGAGGAACTGGGTCAGGGTGAAGCGATAGCCCATAATTTGAGATCCATGTTTGGTCTGAAGGTGCCAGTTCTGTCAATAGTTATTGGAGAAGGTGGCTCTGGTGGTGCACTTGCCATTGGGTGTGCGAATAAATTACTAATGCTTGAAAATGCAGTTTTCTATGTTGCCAGTCCAGAGGCATGTGCAGCAATCTTGTGGAAGAGTGCTAAAGCTGCTCCTAAGGCTGCTGAGAAACTGAAGATTACAGCCACCGAATTGTGCAAACTGGAAATTGCAGATGGTGTTATCCCTGAACCACTTGGCGGTGCACATGCAGATTCAGCTTGGACCTCACAACAGATAAAAATTGCAATCAATGAAACCATGGATGAACTTAGCAAACTTAGTACAGAAGAACTACTCAAACATCGCCATGATAAGTTTCGAAAGATTGGTGGATTCCAGGAAGGCATTCCTATTGATCCTAAGAGGAAGTTCAGCATGAAAAAGAAGGATATACCCATTCCCAAGAAGATTTCTGAT‑‑‑‑‑‑‑‑‑GCTGAAATAGAGGCTGAGATTCAGAAATTGAAGCAACAAATATTAGAAGCCAAGGGATCCTCT‑‑‑‑‑‑CCCGAGCCTCCAAAACATGATTTGGGTGATATGATAAAG‑‑‑‑‑‑‑‑‑‑‑‑AAACTGAAAAGGGAGGTTGATCACGAATACTTTGAGGCAGTTAAAGCCATGGGCTTGACAGACAGGTTCTCAAAACTAAGGGAGGAAGTTACAAAAGCAAATTCACGA‑‑‑‑‑‑‑‑‑AAC‑‑‑CAATCTATTGATCCATCGCTGAAGGATAAGATAGAAAAGTTAGAGGCGGAGTTTGATCAGGGTTTGCCAGCGGCTCCAAACTATGACAAGCTACAGAAGAAGCTTGGCATGTTGAAAGAGTTATCTGAAGTAAAGCATCTGTCAGAGACAAACGAG‑‑‑‑‑‑GAGGTTGTCAGATTG‑‑‑AAGCAAGAAATGCAGACAGTACTTGATGATGTCTTGAACAGTCCCAGAATAAAGGAAAAGTATGAGACATTAAAGGGTGAAATTGAAAGTATTGGTGCATCCTCACCAAGTGATTTGGATGATGAGTTGAAGAAGAAAATCGCTGAGTTTAAGAAAGAGTTAGTTTTGCAGTTTGCTAATGCTATGAAGTCAGCTGGCTTAGATGTTAAGTATGTAAAAAGAAAGGCGCGGGATGGCAGGGACAAA‑‑‑‑‑‑TCTGCATTGTCAGAG‑‑‑‑‑‑‑‑‑TATAAGTCAAAGATTGATGAACTAAACAAAGAT‑‑‑TTAGAAAAGGAAATTGAAGATGCAGTGAACTCAGCAGAT‑‑‑ATTAAGAGCAAGATAGAGTTATTGAATTTGGAAGTTGCTAAGGCAGGAGACACACCTGGT‑‑‑GTGGATTCAAAGAGTAGAATTGATGCTTTGGTGCAACAAATTAAACAGAGCGTT‑‑‑‑‑‑‑‑‑‑‑‑GTTGAAACCCTTGACTCGTCCAGCTTAAAAGAGAAGTATGAAAATCTGTTGTCTGAATTTTCCATCGAA‑‑‑‑‑‑‑‑‑‑‑‑‑‑‑‑‑‑‑‑‑‑‑‑ACTATAAGTTCGAAAACGGCAGATCCAACAGTAGACAGTCCCACCAATGATGATCTGAAAGTGAAAGTTGGCACAAACCGCACTTCTTCT‑‑‑‑‑‑‑‑‑‑‑‑‑‑‑‑‑‑

>Lan_accA1_Lup023032

‑‑‑ATGGCTGCTTCTGCTGCT‑‑‑‑‑‑‑‑‑GCATCTCTTACTGGG‑‑‑‑‑‑‑‑‑GCTTCGGCTTCGGATCTTCTGAGGAGTTCAACCACTGGTTTCAGTGGTGTCCCTTTGAGAACCTTGGGAAGGGCAAGG‑‑‑TTGGCTTTGAAGACT‑‑‑AGGGATTTTGCAGTTTCT‑‑‑‑‑‑TGTAAGTTGAGGAAGGTGAAGAAGCATGAGTATCCTTGGCCTGATAACCCCGATCCCAATGTGAAAGGCGGG‑‑‑‑‑‑GTCCTAAGTCATCTCTCTCCTTTCAAGCCACTCAGAGAGAAGCCAAAG‑‑‑CCTGTTACTTTGGAGTTTGAAAAGCCTCTTCTTGATCTGCAAAAGAAGATCATTGATGTACGAAAGATGGCGAACGAAACTGGTCTGGACTTTAGCGATCAGATTCTTCTATTGGAATCTAAGTACCAGCAGGCTTTAAAGGATCTATATACACATCTGACTCCTATTCAGCGGGTCAACATTGCACGGCATCCTAACAGGCCAACTTTCCTTGATCACATCTTCAACATAACTGAAAAGTTTATGGAACTCCATGGTGATCGGGAAGGTTATGACGATCCTGCCATTGTTGCTGGCATAGGGACTATAGATGGTAGAAGATACATGTTCATTGGTCACCAAAAAGGTAGAAATACTAAAGAAAATATTCAGCGTAACTTTGGGATGCCAACTCCTCATGGTTATAGGAAGGCTCTTCGCTTGATGGAGTATGCTGATCATCACGGATTCCCGATAGTTACTTTCATTGACACGCCTGGGGCATATGCTGACCTTAAATCAGAGGAACTGGGCCAGGGTGAAGCGATTGCCCATAATTTGAGATCCATGTTTGGTCTAAAGGTGCCAGTTGTGTCAATAGTTATTGGAGAAGGTGGTTCTGGTGGTGCACTTGCCATTGGATGTGCCAATAAATTACTTATGCTTGAAAATGCCGTTTTCTATGTCGCTAGTCCGGAGGCATGTGCAGCAATCTTGTGGAAGAGTGCTAAAGCTGCTCCTAAGGCTGCTGAGAAACTGAAGATTACAGCCACCGAATTGTGCAAATTGGAAATTGCTGATGGTGTTATCCCTGAGCCACTCGGCGGTGCACATGCAGATTCAGCTTGGACCTCACAACAGATAAAAAATGCAATCAATGAATCCATGGATGAGCTTAGCAAACTGAGTATAGAAGAACTACTCAAACATCGGCATGATAAATTTCGTAAGATTGGTGGGTTCCAGGAAGGCATTCCTATTGATCCTAAGAGAAAGTTCAGCATGAAAAAGAAGGATATACCCATTCCTAAGAAGATTTCTGAC‑‑‑‑‑‑‑‑‑GCTGAAATAGAGGCTGAGATTCAGAACTTGAAGCAACAAATATCGGAAGCCAAGGGATCCTAT‑‑‑‑‑‑GCCGAGCTTCCGAAACTTGATTTGGGTGATATGATAAAG‑‑‑‑‑‑‑‑‑‑‑‑AAACTGAACAGGGAGATTGATCATGAATACTTCGAGGCAGCTAAAGCCATCGGCTTGGCAGATAGGTTCTCAAAACTAAGGGAGGAAGTTACAAAAGCAAATTCACAA‑‑‑‑‑‑‑‑‑AAC‑‑‑CAATCTATTGATCCATTGCTGAAGGATAAGATAGAAAAGTTAGAGGCGGAGTTTGATCAGGGTTTGCCGGGGGCTCCAAACTATGGCAAGCTACAGAAGAAGCTTGACATGTTGAAAGAGTTATCTAAAGTAAAACATCTAATAGAGACGAACAGG‑‑‑‑‑‑GAGGCTGTCAAATTG‑‑‑AAGCAAGAAGCGATGACAATATTTGATGATGTCTTGAACAATCCCAGAATAAAGGAAAACTATGAGACATTAAAGGGTGAAATTGAAAGTATTGGTGCATCCTCACCAAGTGATTTGGATGATGAGTTGAAGAAGAGAATCGTGGAGTTTAGAAAAGAGGTTGGTTTGCAGCTAGCTAATGCTCTGAAGTCAGCAGGCTTAGTTATATCT‑‑‑GTAAGAGAAAAGCCACGAGAGAACAGTGACGAG‑‑‑‑‑‑TCTTCATCGTCAGAG‑‑‑‑‑‑‑‑‑TATGAGTCAAAGATTGAAGAACTAAACAAAGAT‑‑‑ATAGAAAAGGAAATTGAATCTTCGGTGAACTCATCAGAT‑‑‑ATTAAGAGCAAGATAGAGGCATTAAATCTCGAAATTGCTAAGGCTGGAGACACACCTGAT‑‑‑GCGGATTCAAAGAATAGAATTGCTGCTTTGGTGCAACAAATTAAGCAGAGCCTT‑‑‑‑‑‑‑‑‑‑‑‑GTTGAAACTGTTGACTCATCTAGCTTAAAAGACAAGTATGAAAATCTGGTGTCTAAAGTTTCCACTGAA‑‑‑‑‑‑‑‑‑‑‑‑‑‑‑‑‑‑‑‑‑‑‑‑AATGGAAGTTTGAAAAAGGCAGAGCCAACAGGTGACAATCCCACCAATGATGAGTTGAAAGTGGAAGTTAGCACAAACAACAGCCTTTCT‑‑‑‑‑‑‑‑‑‑‑‑‑‑‑‑‑‑

>Lan_accA2_Lup010366

ATGGCTGCTGCTTCTTCTGCT‑‑‑‑‑‑‑‑‑GCATCTCTTACCGGG‑‑‑‑‑‑‑‑‑GCTTCAGCTTCGGATCTTCTAACGAGTTCAACCAGTGCTTTCAGTGGTATCCCTTTGAGAACCTTGGGAAAGGCAAGG‑‑‑TTGGTTTTGAAAAGG‑‑‑AGGGATTTTACAATTTCA‑‑‑‑‑‑TGTAAGTTGAGGAAGGTGAAGAAGCATGAATATCCATGGCCTGATAACCCTGATCCCAATGTGAAAGGTGGG‑‑‑‑‑‑GTACTAAGTCATCTCTCTCCTTTCAAGCCACTCAAAGAGAAGCCAAAG‑‑‑CCTGTCACTTTGGAGTTCGAAAAGCCTCTTGTTGATCTGCAAAAGAAGATTATCGATGTGCGGAAGATGGCGAATGAAACTGGTCTGGACTTTACTGATCAAATTCTTTCTTTGGAGTCTAAATACCAGCAGGCTTTAAAAGATCTATATACTCATCTGACTCCTATACAGCGGGTCAACATTGCACGACATCCTAACAGGCCAACTTTCCTTGATCATATCTTCAACATAACTGAAAAGTTTGTGGAACTCCATGGTGATCGGGAAGGTTACGATGATCCTGCTATTGTTACTGGCATAGGGACTATAGATGGTAGAAGATACATGTTCATTGGTCACCAAAAAGGTAGAAATACTAAAGAAAACATTCAGCGTAACTTTGGGATGCCAACTCCTCATGGTTACAGGAAGGCTCTTCGCCTGATGGAGTATGCAGATCACCATGGATTCCCGATAGTTACTTTCATTGACACGCCTGGGGCTTATGCTGACCTTAAATCAGAGGAACTAGGCCAGGGTGAAGCAATTGCCCATAATTTGAGATCCATGTTTGGCCTGAAGGTGCCAGTTCTGTCAATAGTTATTGGAGAAGGTGGTTCTGGTGGTGCACTTGCCATTGGATGCGCTAATAAATTACTTATGCTTGAAAATGCTGTTTTCTATGTTGCAAGTCCGGAGGCATGTGCAGCAATCTTGTGGAAGAGTGCTAAAGCTGCTCCTAAGGCCGCTGAGAAACTGAAGATTACAGCCTCTGAATTGTGCCGATTGGAAATTGCAGATGGCATTATCCCTGAACCACTTGGCGGTGCACATGCAGATTCAGCTTGGACCTCACAACAGATAAAAATTGCAATCAATGAATCCATGGATGAGCTTAGCAAAATGAATACAGAAGACCTACTCAAACATCGCCATGATAAGTTCCGAAAGATTGGCGGGTTCCAGGAAGGCATTCCTATTGATCCTAAGAGGAAATTCAGCATGAAGAAGAAGGATATACCCATTCCTAAGAAGATTTCTGAT‑‑‑‑‑‑‑‑‑GCTGAACTAGAGGTTGAGGTTCAGAAACTGAAGCAGCAAATATTAGAAGCTAAGGGATCCTCT‑‑‑‑‑‑GCCGAGCCTCCAACACTTGATTTGGATGATATGATAAAG‑‑‑‑‑‑‑‑‑‑‑‑AAACTGAAAAAGGAGGTTGATCATGAATATTCTGAGGCAGTTAAGGCCATGGGCTTGGCTGACAGGTTGTCGAAACTAAAGGAGGAAGTTACAAAAGCAAATTCACAA‑‑‑‑‑‑‑‑‑AAC‑‑‑CCATTTGCCGATCCATTGCTGAAGGATAAGATAGAAAAGGTAAAGGCGGAGTTTGACCAGGGTGTGTCAGCGGCTCCGAACTATGGCAAGCTGCAGAATAAACTTGGCATGTTGAAAGAATTATATAAATTAAAACATCTGTCCGAGACCAACAAG‑‑‑‑‑‑GAGGCTGCCACATTG‑‑‑AAGCAAGAATTGATGACAATATTTGATGATGTCTTGAACAATCCTAGAATAAAGGAAAACTATGAGAGTTTAAAGGATGAAATTCAAAGTATCGGTGCATCCTCACCAAGTGATTTGGATGATGAGCTGAAGCAGAAAATCATTGAGTTTAGAAAGGAGGTTGACTTGCAATTAACTAATGCTCTGAAGTCAGCAGGCTTAGATGTCAAGTTTGTAAGCGCTAAGCAACGGGGTGACAGGGATGAG‑‑‑‑‑‑GGTTCATTGTCAGAG‑‑‑‑‑‑‑‑‑TATGAGTCAAAGTTTGAAGAACTGAACAAAGAT‑‑‑ATAGAAAAGGAAATTGAGTATTCGGTGAACTCATCGGAT‑‑‑ATCAAGAGCAAGATAGAGCTATTGAAATTGGAGGTTGCAAAGGCTGGAGACACACCGGAT‑‑‑GTGGACTCAAAGAATAGAATTGCTGCTTTGGTGCAACAAGTTAAGCAGAGCCTT‑‑‑‑‑‑‑‑‑‑‑‑GTTGAAACCGTTGACTCGTCCAGCTTAAAAGAGAAGTATGAAAATCTGATGTCTAAAGTTTCCGGTGAA‑‑‑‑‑‑‑‑‑‑‑‑‑‑‑‑‑‑‑‑‑‑‑‑AATGGAAGTTCGAAAAAGGTAAATCCAACAGGAGACACTCCCACCAATGATGAGTTGAAAGTGAAAGTCGGCACAAACCACACCTTTTCT‑‑‑‑‑‑‑‑‑‑‑‑‑‑‑‑‑‑

>Lan_accA3_Lup014474

‑‑‑ATGGCTGCTTCTTCTGTT‑‑‑‑‑‑‑‑‑GCTTATCTTACCGGG‑‑‑‑‑‑‑‑‑AATTCAGCTTCGGATCTTCTGAGGAATTCAACCAGTGGTTTCAGTGGTGTCCCTTTGAGAACCTTGGGAAGGGCAAGG‑‑‑TTGGTTTTGAAGAAA‑‑‑CGGGATTTTACGGTTTCA‑‑‑‑‑‑TGTAAGTTGAGGAAGGTGAAGAAGCATGAGTATCCATGGCCTGATAACCCGGATCCCAATGTGAAAGGTGGG‑‑‑‑‑‑GTGCTAAGTCATCTATCTCCTTTCAAGCCACTCAAAGAGAAGCCGAAG‑‑‑CCTGTCACTTTGGAGTTTGAAAAGCCTCTTATTGATCTGCAAAGGAAGATCATCGATGTTCGGAAGATGGCGAACGAAACTGGTCTGGATTTTAGTGATCAGATTCTTTCATTGGAGTCGAAGTACCAGCAGGCTTTAAGGGATCTATATACACATCTGACTCCTATACAGCGGGTCAACATTGCACGGCATCCTAACAGGCCAACTTTCCTCGATCATATCTTCAACATAACGGAAAAGTTTGTGGAACTCCATGGTGATCGGGAAGGTTATGATGACCCTGCTATTGTTACTGGCATAGGGACTATAGATGGTAGAAGATACATGTTCATTGGTCACCAAAAAGGTAGAAATACGAAAGAAAACATTCAGCGTAACTTTGGGATGCCAACTCCTCATGGTTACCGGAAGGCTCTTCGCTTGATGGAGTATGCCGATCATCATGGATTTCCAATAGTTACTTTCATTGACACGCCTGGGGCATATGCTGACCTTAAATCAGAGGAACTGGGCCAGGGTGAAGCGATTGCCCATAATTTGAGATCGATGTTTGGTCTGAAGGTGCCGGTTCTGTCAATAGTTATTGGAGAAGGTGGTTCTGGTGGTGCACTTGCCATTGGATGTGCAAATAAATTACTAATGCTTGAAAATGCTGTTTTCTATGTTGCCAGTCCAGAGGCATGTGCAGCAATCTTGTGGAAGAGTGCTAAAGCTGCTCCTAAGGCTGCTGAGAAACTGAAGATTACGGCCTCCGAATTATGCAGACTGGAAATCGCAGATGGTGTTATCCCTGAACCACTTGGCGGTGCACATGCAGATTCAGCTTGGACCTCACAACAGATAAAAAATGCAATCAATGAAACCATGGATGAGCTTAGCAAACTGAGTACAGAAGATCTACTCAAACATCGCCATGATAAGTTTCGAAAGATTGGTGGGTTCCAGGAAGGCATTCCTATCGATCCTAAGAGGAAATTCAGCATGAAGAAGAAGGATATACCCGCTCCTAAGAAGATTTCTGAT‑‑‑‑‑‑‑‑‑GCCGAAATAGAGATTGAAGTTCAGAAACTGAAGCAACAAATATTGGAAGCTAAGGGATCCCCT‑‑‑‑‑‑ACCGAGCCTCCAAAACTTGATTTGGATGATATGATAAAG‑‑‑‑‑‑‑‑‑‑‑‑AAACTGACAAGGGAGGTTCATCATGAGTACTCCGAGGCCGTTAAAGCCATGGGGTTGGCAGACAGGATGTCGAAACTAAGGGAGGAAGTTACGAAAGCAAATTCACAA‑‑‑‑‑‑‑‑‑AAC‑‑‑CAGTTTATCGATCCATTGCTGAAGGATAAGATGGAAAGGCTAGAAGCAGAGTTTGAGCAGGGTTTGCCAGCCACTCCAAACTATGGCAAGCTGCAGAATAAGCTTAGCATGTTGAAAGAATTATCAAAAGTAAAGAAAATGTCGGAGAAGAACAAG‑‑‑‑‑‑GAGGCTGCTATATTG‑‑‑GATCAAAAATTGAAGACAGTATACGATGATGTCATAAACAATCCTAGTATAAAGGAAAACTTTGAAACATTAAAGGCTGAAATTGAAAGTACCGGTGCATCCTCACCAAGTGATTTGGATGATGAGTTGAAGAAGAAAATCGTTGAGTTTAACAAAGAGGTAGACTTGCAGTTAGCTAATGCTCTGAAGTCAATTGGCTTAGATCTTCTGTATGTAGGATCAAAGGCACGAGATGGAAGGTACGAG‑‑‑‑‑‑TCTTCATTGTCAGAG‑‑‑‑‑‑‑‑‑TATATTTCAAAGATCGATGAACTAAACAAAGAT‑‑‑TTAGTAAAGGAAATTGAAAATTCAGTGAACTCATCAGAT‑‑‑ATTAAGAGCAAGATAGAGTTATTGAAATTGGAAGTTGCTAAGGCACGAGACACACCTGGT‑‑‑GTGGATTCAAAGAGTAGAATTGATGCTTTGGTGCAACAAATTAAACAGAGCCTT‑‑‑‑‑‑‑‑‑‑‑‑GTTGAAACCGTTGACTCGTCCAGCTTAAAAGAGAAGTATGAAAATCTGGTGCCTAAAGTTTCCAGCGAA‑‑‑‑‑‑‑‑‑‑‑‑‑‑‑‑‑‑‑‑‑‑‑‑AATGGAAGTTTGAAAACGGCAGATCAAACAGTAGACAGTCCCACCAATGATGACTTGAAAGTGAAAGTTGGCACAAACCACACTTCT‑‑‑‑‑‑‑‑‑‑‑‑‑‑‑‑‑‑‑‑‑

>Car_accA_Ca_26217

‑‑‑‑‑‑ATGGCTTCCTCTTCT‑‑‑‑‑‑‑‑‑ACAACACTTGCTGGT‑‑‑‑‑‑‑‑‑TCTGCTGCTTCTGATCTTCTCAGGAGTACAACTACCGGTTTCACTGGTGTCCCTTTGAGAACCTTAGGAAGGGTAGGG‑‑‑TTGGTTTTGAAAAGA‑‑‑AGGGATTTAACTGTTTCT‑‑‑‑‑‑GCTAAGTTGAGGAAAGTGAAGAAGAAGGAATATCCATGGCCTGCTAACCCTGATCCCAATGTTAAAGGTGGG‑‑‑‑‑‑GTGTTGAGTCATCTCTCCCCTTTCAAGCCACTCAAAGAGAAGCCTAAG‑‑‑CCTGTTACTTTAGATTTCGAAAAGCCTCTTGTTGATCTGCAAAAGAAGATCATCGAAGTTCGGAAGATGGCGAACGAAACCGGTCTGGATTTCAGTGATCAGATTCTCTTATTGGAGACTAAATACGATCAGGCTTTAAAGGATCTATATACACATCTTACTCCTATACAGCGGGTCAACATTGCACGGCATCCTAACAGGCCAACTTTCCTTGATCACATCTTTAACATAACTGAAAAGTTTGTGGAACTCCATGGTGATCGGGAAGGTTATGATGATCCTGCTATTGTTACCGGTATAGGGACTATAGATGGTAGAAGCTACATGTTCATAGGCCACCAAAAGGGTAGAAACACTAAAGAAAATATTCAGCGTAACTTTGGGATGCCAACTCCTCATGGTTATAGGAAAGCTCTTCGCTTGATGCAATATGCAGATCACCACGGGTTCCCGATAGTTACTTTTATTGACACCCCTGGGGCATATGCTGACCTTAAATCAGAAGAACTAGGCCAAGGTGAAGCAATTGCTTATAATTTGAGAGCCATGTTTGGTCTGAAGGTGCCAATCGTGTCTATAGTTATCGGGGAAGGAGGATCTGGTGGTGCTCTTGCCATTGGATGTGGCAATAAATTACTCATGCTTGAAAATGCAGTGTTCTATGTTGCCAGTCCAGAGGCGTGTGCTGCAATCTTGTGGAAGAGTTCTAAAGCTGCTCCAAAGGCTGCTGAGAAACTGAAGATTACAGCGCCTGAATTGTGCAAATTGGAAGTTGCAGATGGCATTATACCTGAACCCCTCGGTGGTGCACATGCGGATCCAAGTTGGACCTCTCAGCAGATCAAAATTGCAATCAATGAAGCTATGGATGAACTCACCAAGATGAACACAGAAGACCTATTAAAAGATCGCATGCTTAAGTTTCGAAAGCTCGGTGGGTTCCAAGAAGGAATTCCTTTAGATCCCAAGAAGAAACGCAACATGAAGAAAAAGGATATACCTGTTGCTCCGAAGATTTCTGAC‑‑‑‑‑‑‑‑‑TCTGAATTAGGAGTTGAGATTGAGAAACTGAAGCAAAAAATTTTGGAAGGTAAAGAGTCCTCTTCT‑‑‑TCCGAGCCTCCAAAACTCGATCTGGACGAGATGATAAAG‑‑‑‑‑‑‑‑‑‑‑‑AAACTGGAAAAGGAGGTTAATCAAGAATTCTCTGAGGCTGTTAAAGCCCTGGGCTTAACAGAGAGGTTGTCGAAACTACAAGATGAAATTTCAAAAGCAAATGCAGAT‑‑‑‑‑‑‑‑‑AAC‑‑‑CAATCTATTGATCCATTGCTGACGGATAAGATAGAAAAACTAAAGGCAGATTTTAATCAGGGATTATCAGCAGCTCCTAATTCGGGTCGGCTGCAGAAGAAGCGTGACATGTTGAGAGAGTTGACTAAAGCAAAGCTTCTGACAGATAGAAGCAAG‑‑‑‑‑‑GAGGCTGCCACACTT‑‑‑AAGCAAGAATTGAAGAAAAAATTTGACGACGTCATGAATAATCCTAGAATAAAGGAAAACTACAAGGCGTTACAGTCAGAAATCCAGCGTGTCAGAGCATCCGCGTCAAGTGATTTGGATGATGAATTGAAGAAGAAAATCATCGAGTTCAACAAGGAGGTAGACTCACAGCTGGCTAATGCTCTGAAGGCATCAGGGTTAGATGTTCAGTTAGTGAAACCGGGACGAGACAGCAACAAG‑‑‑‑‑‑‑‑‑‑‑‑TCGTCGGTG‑‑‑‑‑‑‑‑‑‑‑‑‑‑‑‑‑‑‑‑‑CCAGAGATCGAGGAACTAAACAAAGATGTAGTACAAAAGGAAATTGATATTTTGGCAAACTCGTCGACAAGTATTAAGAGCATGATAGAGCAATTGAAACTGGAGGTTGCCAGGGCTGGAGGGAAACCAGAT‑‑‑TCTGAATCTAAGAACAGAATTGAGACTTTGACGCAACAGATTAAGCAGAGCCTTGCCGAGGCCGTAGCCGAGGCCGTTGCCTCGTCTAGCTTGAAAGAGAAGCCTGAAAATCTGGTGTCTGGA‑‑‑‑‑‑‑‑‑‑‑‑‑‑‑‑‑‑‑‑‑‑‑‑‑‑‑‑‑‑‑‑‑‑‑‑GACGGAAGTTTAAAAAAGGAAGGTCCAACAGGAGACAGTCTCACCGATGACGAGTTGAGAGAGAAAGTTGGTGCAAATCGCAACTTTTCT‑‑‑‑‑‑‑‑‑‑‑‑‑‑‑‑‑‑

>Lja_accA1_Lj0g3v0256879

‑‑‑‑‑‑‑‑‑ATGGCTTCTTCT‑‑‑‑‑‑‑‑‑GCAGCTCTTACCGGT‑‑‑‑‑‑‑‑‑GCTTCGGCTTCGGATCTTCTAAGGAGTTCAACTAGTGGTTTCAGTGGTGTCCCTTTGAGAACCTTGGGAAGGTCAAGG‑‑‑TTGGCCATAAAAACA‑‑‑AGGGATTTTTCAGTTGTT‑‑‑‑‑‑GCTAAGCTCAGGAAGGTTAAAAAGCATGAATATCCCTGGCCTGATAACCCTGATCCCAATGTGAAGGGTGGG‑‑‑‑‑‑GTGCTAAGTCATCTGTCCCCTTTTAAGCCATTAAAAGTGAAGCCAAAG‑‑‑CCTGTCACTTTGGATTTCGAAAAGCCTCTTGTTGATCTGCAAAAGAAGATCATCGATGTGCGAAAGATGGCAAATGAAACGGGTCTGGACTTCAGCGATCAGATTCTCTCATTGGAGGCCAAGTACCAGCAGGCTTTAAAGGATCTATATACACATCTGACTCCTATACAGCGGGTCAGCATTGCGCGGCATCCTAACAGGCCCACTTTTCTTGATCACATCTTTAACATAACTGAAAAGTTTGTGGAGCTCCATGGTGACCGGGCTGGTTATGATGATCCTGCTATTGTGACTGGTATTGGTACTATAGATGGTAGAAGATACATGTTCATTGGTCACCAAAAGGGTAGAAATACGAAAGAGAACATTCAGCGTAACTTTGGGATGCCAACCCCACATGGCTACAGGAAGGCTCTTCGCTTAATGGAATATGCAGATCATCATGGGTTCCCAATCGTTACTTTCATTGACACACCTGGGGCATATGCTGACCTTAAATCAGAGGAACTAGGCCAAATCCATGTTCGGTTTGAA‑‑‑‑‑‑‑‑‑‑‑‑‑‑‑‑‑‑‑‑‑‑‑‑‑‑‑‑‑‑‑‑‑‑‑‑‑‑‑‑‑‑‑‑‑‑‑‑‑‑‑‑‑‑‑‑‑‑‑‑‑‑‑‑‑‑‑‑‑‑‑‑GCCCTAGCCATTGGATGTGGTAATAAATTACTCATGCTTGAAAATGCAGTTTTCTATGTTGCCAGTCCAGAGGCATGTGCAGCAATCTTATGGAAGACTGCTAAAGCTTCTCCAAAGGCTGCTGAGAAACTGAAGATTACAGCGAATGAATTGTGCAGATTGCAAATTGCTGATGGTGTTATACCTGAACCTATTGGTGGAGCACATGCAGATCCAAATTGGACCTCTCAACAGATAAAAATTGCAATCAATGAAGCCATGAGCGAGCTCACCAAGTTGAACACAGAAGATCTATTAAAAGATCGCATGCTTAAGTTCCGAAAGATAGGTGGGTTCCAGGAAGGAATTCCTGTAGATCCTAAGAGGAAAGTCAACATGAAGAAGAAGGATGTACCTGTTGCTGTAAAGATTCCCGAT‑‑‑‑‑‑‑‑‑GCTGAGTTAGAGGTTGAAGTTGAGAAACTTAAGCAACAAATTTTGAATGCTAATGAATCTTCT‑‑‑‑‑‑TCCAAGCCTCCAAAACTAGATCTGGATGAGATGATAATG‑‑‑‑‑‑‑‑‑‑‑‑AAACTAGAAAGGGAGGTTGATCAAGAATACTCTGAGGCAGTTAAAGCCTTGGGCTTAACAGACAGGTTCTCGAAATTACAGGAGGAAGTTTCAGCAGCAAATTCAGAT‑‑‑‑‑‑‑‑‑AAT‑‑‑CAACTTTCTGACCCATTGCTGAAGGATAAGATAGAAAAACTAAAGGTGGAGTTTGAACAGGGTTTGTCAGCAGCTCCCAATCATGGCAGGCTGCAGAATAAGCTTGATATGTTGAAAGAATTGTCTAAAGTAAAGCATCTGTCAGAAACAAAAAAG‑‑‑‑‑‑CAG‑‑‑‑‑‑‑‑‑‑‑‑‑‑‑AAACAAGAAGTGAGAAAGAAATTTGTTGAGGTCATCAGTAATCCTAGAATAAAAGAAAACTATGAAGCATTGACTGCTGAAATTCAACGTGTAGGCGCATCCTCATCAAGCGATTTGGATGATGAGTTGAAGAACAAAATCATTGAGTTTAATAAAGAGGTAGACTCACAGCTGGTTAATGCTCTGAAGTCAGTAGGCTTAGATGTTCAGTTGGTAAAAGCAGAGGGACGAGACAGCAAGGAG‑‑‑‑‑‑‑‑‑TCTGAAGTGTCAGAG‑‑‑‑‑‑‑‑‑TATGTTCCAGAGATAGAAGAACTAAAGAAGGAT‑‑‑ATAGAAAAGGAGATTGAAATTTCGGCAAGCTCATCTGAT‑‑‑GTTAAGAGCAAAATAGAGCAATTGAAACTGGAGGTTGCCAAGGCTGGAGAGACACCTGAT‑‑‑CCAGAATCAAAGAAAAGAATTACTTCTTTGATGCAACAAATTAAGCAGGGCCTT‑‑‑‑‑‑‑‑‑‑‑‑GTAGAGGCCATTGACTCATCTAGCATAAAAGAGAAGTATGACAATCTGGGAAATTTCGGGTATGATCCGGAG‑‑‑‑‑‑‑‑‑‑‑‑‑‑‑‑‑‑‑‑‑GATGGAGCTCTGAGGACC‑‑‑TTGCCTAGTGGC‑‑‑‑‑‑‑‑‑‑‑‑‑‑‑‑‑‑‑‑‑‑‑‑CGGCTAGAGCTGACATGGACACGTGAGTCATTTTTAACTAAATTT‑‑‑‑‑‑

>Cca_accA2_C.cajan_33027

‑‑‑‑‑‑ATGGCTGCTGCTTCT‑‑‑‑‑‑‑‑‑GCATCTCTTGCTGGT‑‑‑‑‑‑‑‑‑GCTTCTGCTTCGGATCTTTTAAGGAGTTCAACCAGCGGTTTCAGTGGTGTTCCTTTGAGAACCTTGGGGAAGGGAAGG‑‑‑TTGGTTTTGAAGAGG‑‑‑AGGGATTTTACTGTTGGT‑‑‑‑‑‑GCTAAGATGAGGAAGGTGAAGAAGCATGAATATCCCTGGCCTGCTAACCCTGATCCCAATGTGAAAGGTGGG‑‑‑‑‑‑GTGCTGACCCACCTTTCATATTTCAAGCCACTCAATGAGAAGTCAAAG‑‑‑CCTGTCACCTTAGAGTTTGAAAAGCCTCTTCTTGATCTGCAGAAGAAGATCATTGATGTGCGGAAGATGGCGAACGAAACTGGGCTGGACTTCAGCGATCAGATTCTCTCGTTGGAGTCCAAGTATCAGCAGGCTTTAAAGGATTTATATACGCATCTGACTCCTATACAGCGGGTCAACATTGCACGGCATCCTAACAGGCCAACTTTCCTTGATCACATCTTTAACATAACTGAAAAGTTTGTTGAACTCCATGGTGATCGGGCAGGTTATGATGATCCTGCTATTGTTACTGGTTTGGGAACTATAGATGGTAGAAATTATATGTTCATTGGTCACCAAAAGGGTAGAAATACTAAAGAAAACATTCAGCGTAACTTTGGGATGCCAACTCCTCATGGTTACAGGAAGGCTCTTCGGTTGATGGAGTATGCAGATCATCATGGGTTCCCCATAGTTACTTTCATTGACACACCTGGGGCTTATGCTGACGTTAGATCAGAGGAACTAGGCCAAGGTGAAGCGATTGCTCAAAATTTGAGATCCATGTTTGGTCTGAAGGTGCCAATTATATCTATAGTTATTGGAGAAGGTGGTTCAGGTGGTGCCCTAGCCATTGGATGTGCTAATAAATTACTCATGCTTGAAAATGCTGTTTTCTATGTTGCCAGTCCAGAGGCATGTGCAGCAATCTTATGGAAGAGCGCTAAAGCTGCTCCGAAGGCTGCTGAGAAACTGAGGATTACAGCCCCTGAACTGTGCAAATTACAAATAGCAGATGGTATTATACCTGAACCACTTGGTGGTGCACATGCTGATCCAGAATGGACCTCTAAACAGATAAAAAAGACAATCAATGAAACCATGGATGAGCTCTCCAAGATGAACACGGAAGACCTATTAAAACATCGCATGCTTAAGTACAGAAAGATTGGTGGTTTCCAGGAAGGTATTCCTATAGATCCTAAGAGAAAGGTCAATATGAAGAAGAGGGATCTATCTATCGCT‑‑‑AAGATTCCCGAT‑‑‑‑‑‑‑‑‑GCTGAGCTAGAAGTTGAGATTGAGAAACTGAAACAACAGGTTTCGGAAGTTAAGGAATCTTCT‑‑‑‑‑‑‑‑‑GTTCCTCCAAAACTAGATCTGGATGAGATGATGCAG‑‑‑‑‑‑‑‑‑‑‑‑CAACTGGAAAGGGAGGTGGATCAAGAATACTCCGAGGCAGTTAAAGGTGCGGGCTTGACAGACAAATTGTTGAAGTTACGGGAGGAAGTTTCAAAAGCAAATGCAGAT‑‑‑‑‑‑‑‑‑AAT‑‑‑GAAACTATTGATCCATTACTGAAGGATGAGGTAGAAAAGCTAAGGGTGGAGTTTGAACAGCGATTGCAAGCAGCTCCCAATTATGGTAGGCTGCAGACTAAGCTTAACCGTTTGAAAGAATTATCTAAAGTGAAGCATTTGTCAGCTACAAAGAAG‑‑‑‑‑‑GAGGCTGCCACATTG‑‑‑AAGCAAGAGTTGAAGATGAAAGTTGATCCTGTCTTGAATGATCCTAAAATAAGGGAAAAGTTTGAAGCATTAAAGGCTGAAATTGAAGATGCTGGTGCATCCTCACCAAGTGATTTGGATGATGAGTTGAAGAAGAAAATCGTTGAGTTTAGGAAAGAGGTGGACTCACAGCTGGTTAGTGCTCTGGAGTCAGCAGGCTTTGAAGTCATTGGAGAACAAGACAGACAA‑‑‑‑‑‑‑‑‑‑‑‑‑‑‑‑‑‑‑‑‑‑‑‑‑‑‑‑‑‑‑‑‑‑‑‑‑‑‑‑‑‑‑‑‑‑‑‑‑‑‑GAGCCAGGCGCAGAAGAACTAAAAGAAGAA‑‑‑ATAGAAAAGCAAATTGAAAGTTTGGTAAACTCATCAGATGATATTAAGAGCATGATAGCTCAATTGAAATCGGAGGTGGCCAAGGCTGGAGAGACTCCTGAT‑‑‑TCAGATTCAAAGAATAGAATTGATGCTTTGGCGCAAAAAATTAAGCAGAGCCTT‑‑‑‑‑‑‑‑‑‑‑‑GCAGAGGCTATTGACTCGTCTAGCTTAAAAGAACAGTATGATAATCTGGTATCTAAA‑‑‑‑‑‑‑‑‑‑‑‑‑‑‑‑‑‑‑‑‑‑‑‑‑‑‑‑‑‑‑‑‑‑‑‑GATGTAAGTTTGAAACATGAAGATCCAGCCGAAGACAATCTCACTGCTGACGAGTTGAGAATGAAAGTTGGTGCAAACCGCAATATTTCT‑‑‑‑‑‑‑‑‑‑‑‑‑‑‑‑‑‑

>Gma_accA1_Glyma.18G195700

‑‑‑‑‑‑ATGGCTGCTTCTTCT‑‑‑‑‑‑‑‑‑GCATCTCTTTCTGGT‑‑‑‑‑‑‑‑‑GCTTCTGCTTCGGATCTTCTGAGGAGTTCAACCAGCGGTTTCAATGGTGTTCCTTTGAGAACCATGGGGAAGGGAAAG‑‑‑TTGGTTTTGAAGAGG‑‑‑AGGAACTTTACAGTTGCT‑‑‑‑‑‑GCCAGGCTGAGGAAGGTGAAGAAGCATGAATATCCTTGGCCTCCTAACCCTGATCCCAATGTGAAAGGTGGG‑‑‑‑‑‑GTGCTGAGCCACCTTTCCTTGTTCAAGCCACTCAAGGAGAAGCCAAAG‑‑‑CCTGTCACTTTGGATTTTGAAAGGCCTCTTGTTGATCTGCAAAAGAAGATCATTGATGTACAGAAGATGGCGAACGAAACTGGACTGGACTTCAGTGATCAGATTCTCTCATTGGAGACCAAGTACCACCAGGCTTTAAAGGATCTGTATACGCATCTGACTCCTATTCAGCGGGTCAACATCGCACGGCACCCTAACAGGCCAACTTTCCTTGATCATGTGTTTAACATAACTGAAAAGTTTGTTGAACTCCATGGTGATCGGGCAGGTTACGATGATCCTGCTATTGTTACTGGTCTAGGGACTATAGATGGTAGAAGCTACATGTTCATTGGTCACCAAAAGGGTAGAAATACTAAAGAAAATATTCAGCGTAACTTTGGGATGCCAACTCCTCATGGTTACAGGAAGGCCCTTCGCTTGATGGAATATGCAGATCATCATGGGTTCCCCATAGTTACTTTCATTGACACGCCTGGGGCATATGCTGACCTTAAATCAGAGGAACTAGGACAGGGTGAAGCGATTGCTCACAATTTGAGATCCATGTTTGGTCTGAAGGTGCCAGTTATATCTATAGTTATTGGAGAAGGTGGTTCAGGTGGCGCCCTAGCCATTGGATGTGCTAATAAATTACTCATGCTTGAAAATGCTGTTTTTTATGTTGCCAGTCCAGAGGCATGTGCAGCAATCTTGTGGAAGACAGCTAAAGCTTCTCCAAAGGCTGCTGAGAAATTGAAGATTACAGCCACTGAACTGTGCAAATTACAAATTGCAGATGGTGTTATACCTGAGCCACTTGGTGGTGCACATGCAGATCCAGAGTGGACCTCTCAACAGATAAAAAAGGCTATCAAAGAAACCATGGATGAGCTCATGAAGATGAACACAGAAGAACTGTTAAAACATCGCATGCTTAAGTTCAGAAAGATTGGTGGATTCCAGGAAGGTATTCCTATAGATCCTAAGAGAAAAGCCAACATGAAGAAGAGGGATCTATCTATTGCT‑‑‑AAGATTCCTGAT‑‑‑‑‑‑‑‑‑GCTGAACTAGAAGTTGAGGTTGAGAAACTGAAGCAACAGGTTTTGGAAGCTAAGGAATCTTCT‑‑‑‑‑‑CCTGTTCCTCCAAAACTAGATCTGGATGAGATGCTAAAG‑‑‑‑‑‑‑‑‑‑‑‑CAACTGGCAAGGGAGGTCGATCTAGAATACTCTGAGGCAGTTAAAGCCACGGGCTTGACAGACAGTTTGTTGAAACTAAGGGAGGAAGTTTCGAAAGCAAATGCAGAT‑‑‑‑‑‑‑‑‑AAT‑‑‑CAAATTGTTGATCCATTGCTGGAGGGTAAGATAGAAAAGCTAAGGGTGGAGTTTGAACAGCAATTGCGTGCAGCTCCCAATTATGGTAGGCTGCAGAATAAGCTTAACTATCTGAGCGAATTATGTAAAGTAAAGCTTCTGTCAGATGGAAAGAAGGACAATGAGGCTGTCACATTT‑‑‑AAGCAAGAGTTGAAGAAAAAAATTGATAATGCCTTGAGTGATCCAAAAATAAGGGAGACATTTGAAGCATTAAAGGCTGAAATTAAAGGTGTTGGTGCATCCTCAGCAAGTGATTTGGATGACGAGTTGAAGAAGAAAATCATTGAGTTTATCAAAGAAGTA‑‑‑‑‑‑‑‑‑‑‑‑‑‑‑‑‑‑‑‑‑‑‑‑‑‑‑‑‑‑‑‑‑‑‑‑‑‑‑‑‑‑‑‑‑‑‑‑‑‑‑‑‑‑‑‑‑‑‑‑‑‑‑‑‑‑‑‑‑‑‑‑‑‑‑‑‑‑‑‑‑‑‑‑‑‑‑‑‑‑‑‑‑‑‑‑‑‑‑‑‑‑‑‑‑‑‑‑‑‑‑‑‑‑‑‑‑‑‑‑AAAGAAGTAAAAGAAGTAAAAGAGGTA‑‑‑ATAGAAAATCAAATTGAAAGTTTGGTAAACTCGTCGGATGATATTAAGAGCAAGATACTGCAATTGAAATTGGAGGTTCCTAAGGCTGGAGAGACGCCTGAT‑‑‑TCAGAACCAAAGAATAGAATTGGTGCTCTGGTGCAACTAATTAAGCCGAGCCTA‑‑‑‑‑‑‑‑‑‑‑‑GTGGAGGCCGTTGACTCGTCTGGCTTAAAAGATCTGTTTGAAAATCTGGTGTCTAAT‑‑‑‑‑‑‑‑‑‑‑‑‑‑‑‑‑‑‑‑‑‑‑‑‑‑‑‑‑‑‑‑‑‑‑‑GATGGAAGTTTGACACATGAAGATCCAGCTAGAGACAGTCTCACCGATGACCAG‑‑‑‑‑‑‑‑‑‑‑‑‑‑‑‑‑‑‑‑‑‑‑‑‑‑‑‑‑‑‑‑‑‑‑‑‑‑‑‑‑‑‑‑‑‑‑‑‑‑‑‑‑‑

>Gma_accA2_Glyma.18G195900

‑‑‑‑‑‑ATGGCTGCTTCTTCT‑‑‑‑‑‑‑‑‑GCATCTCTTTCTGGT‑‑‑‑‑‑‑‑‑GCTTCTGCTTCGGATCTTCTGAGGAGTTCAACCAGCGGTTTCAATGGTGTTCCTTTGAGAACCTTGGGGAAGGGAAAG‑‑‑TTGGTTTTGAAGAGG‑‑‑AGGGACTTTACAGTTGCT‑‑‑‑‑‑GCTAAACTGAGGAAGGTGAAGAAGCATGAATATCCTTGGCCTCCTAACCCTGATCCCAATGTGAAAGGTGGA‑‑‑‑‑‑GTGCTGAGCCACCTTTCCATGTTCAAGCCACTCAAGGAGAAACCAAAG‑‑‑CCTGTCACTTTGGATTTTGAAAAGCCTCTTGTTGATCTGCAAAAGAAGATCATTGATGTACAAAAGATGGCGAACGAAACTGGACTGGACTTCAGTGATCAGATTCTCTCATTGGAGACCAAGTACCAGCAGGCTTTAAAGGATCTGTATACGCATCTGACTCCTATTCAGCGGGTCAACATCGCACGGCACCCTAACAGGCCAACTTTCCTTGATCATGTCTTTAACATAACTGAAAAGTTTGTTGAACTCCATGGTGATCGGGCAGGTTATGATGATCCTGCTATTGTTACTGGTCTAGGGACTATAGATGGTAGAAGCTACATGTTCATCGGTCACCAAAAGGGTAGAAATACTAAAGAAAACATTCAGCGTAACTTTGGGATGCCAACTCCTCATGGTTACAGGAAGGCCCTTCGCTTGATGGAATATGCAGATCATCATGGGTTCCCCATAGTTACTTTCATTGACACGCCTGGGGCATATGCTGACCTTAAATCAGAGGAACTAGGACAGGGTGAAGCGATTGCTCACAATTTGAGATCCATGTTTGGTCTGAAGGTGCCAGTTATATCTATAGTTATTGGAGAAGGTGGTTCAGGTGGCGCCCTAGCCATTGGATGTGCTAATAAATTACTCATGCTTGAAAATGCTGTTTTTTATGTTGCCAGTCCAGAGGCATGTGCAGCAATCTTGTGGAAGACAGCTAAAGCTTCTCCAAAGGCTGCTGAGAAATTGAAGATTACAGCCACTGAACTGTGCAAATTACAAATTGCAGATGGTGTTATACCTGAGCCACTTGGTGGTGCACATGCAGATCCAGAGTGGACCTCTCAACAGATAAAAAAGGCTATCAAAGAAACCATGGATGAGCTCATGAAGATGAACACAGAAGAACTGTTAAAACATCGCATGCTTAAGTTCAGAAAGATTGGTGGATTCCAGGAAGGTATTCCTATAGATCCTAAGAGAAAAGCCAACATGAAGAAGAGGGATCTATCTATTGCT‑‑‑AAGATTTCTGAT‑‑‑‑‑‑‑‑‑GCTGAACTAGAAGTTGAGGTTGAGAAACTGAAGCAACAGGTTTTGGAAGCTAAGGAATCTTCT‑‑‑‑‑‑CCTGTTCCTCCAAAACTAGATCTGGATGAGATGCTAAAG‑‑‑‑‑‑‑‑‑‑‑‑CAACTGACAAGGGAGGTTGATCTAGAATACTCTGAGGCAGTTAAAGCCACGGGCTTGACAGACAGTTTGTTGAAACTAAGGGAGGAAGTTTCAAAAGCAAATGCAGAT‑‑‑‑‑‑‑‑‑AAT‑‑‑CAAATTGTTGATCCATTGCTGAAGGATAAGATAGAAAAGCTAAGGGTGGAGTTTGAACAGCAACTGCGTGCAGCTCCCAATTATGGAAGGCTGCAGAATAAGTTTACCTATCTGAGCGAATTATGTAAAGTTAAGCTTCTGTCAGATGCAAACAAGGACAATGAGGCTGTCACATTT‑‑‑AAGCAAGAGTTGGAGAAAAAAGTTGATAATGCCTTGAGTAATCCAAAAATAAGGGAAACATTTGAAGCATTAAAGGCTGAAATTAAAGGTGCTGGTGCATCCTCAGCAAGTGATTTGGATGACGAGTTGAAGAAGAAAATCGTTGAGTTTATGATAGAACTA‑‑‑‑‑‑‑‑‑‑‑‑‑‑‑‑‑‑‑‑‑‑‑‑‑‑‑‑‑‑‑‑‑‑‑‑‑‑‑‑‑‑‑‑‑‑‑‑‑‑‑‑‑‑‑‑‑‑‑‑‑‑‑‑‑‑‑‑‑‑‑‑‑‑‑‑‑‑‑‑‑‑‑‑‑‑‑‑‑‑‑‑‑‑‑‑‑‑‑‑‑‑‑‑‑‑‑‑‑‑‑‑‑‑‑‑‑‑‑‑AAAGAAGTAAAAGAAGTAAAAGAGGTA‑‑‑ATAGAAAATCAAATTGAAAGTTTGGTTAACTCATCGGATGATATTAAGAACAAGGTACTGCAATTGAAATTGGAGGTTCCTAAGGCTGGAGAGACGCCTGAT‑‑‑TCAGAATCAAAGAGTAGAATTGGTGATTTTATATTTAGAACATCATCTAGAATT‑‑‑‑‑‑‑‑‑‑‑‑ATTATGGCCATA‑‑‑‑‑‑‑‑‑CTTCTTAGAGACATGTTTGGTAATTTT‑‑‑‑‑‑‑‑‑‑‑‑‑‑‑‑‑‑‑‑‑‑‑‑‑‑‑‑‑‑‑‑‑‑‑‑‑‑‑‑‑‑‑‑‑‑‑‑‑‑‑‑‑‑‑‑‑‑‑‑AAGGAGATACCGTGT‑‑‑‑‑‑‑‑‑‑‑‑‑‑‑‑‑‑‑‑‑‑‑‑‑‑‑‑‑‑‑‑‑‑‑‑‑‑‑‑‑‑‑‑‑‑‑‑‑‑‑‑‑‑‑‑‑‑‑‑‑‑‑‑‑‑‑‑‑‑‑‑‑‑‑‑‑‑

>Gma_accA3_Glyma.18G196000

‑‑‑‑‑‑ATGGCTGCTTCTTCT‑‑‑‑‑‑‑‑‑GCATCTCTTTCTGGT‑‑‑‑‑‑‑‑‑GCTTCTGCTTCGGATCTTCTGAGGAGTTCAACCAGCGGTTTCAATGGTGTTCCTTTGAGAACCTTGGGGAAGGGAAAG‑‑‑TTGGTTTTGAAGAGG‑‑‑AGGGACTTTACAGTTGCT‑‑‑‑‑‑GCTAAGCTGAGGAAGGTGAAGAAGCATGAATATCCTTGGCCTGCTAACCCTGATCCCAATGTGAAAGGTGGG‑‑‑‑‑‑GTGCTGAGCCACCTTTCCTTGTTCAAGCCACTCAAGGAGAAGCCAAAG‑‑‑CCTGTCACTTTGGATTTTGAAAAGCCTCTTGTTGATCTGCAAAAGAAGATCATTGATGTACAGAAGATGGCGAACGAAACTGGACTGGACTTCAGTGATCAGATTCTCTCATTGGAGAACAAGTACCAGCAGGCTTTAAAGGATCTGTATACGCATCTGACTCCTATTCAGCGGGTCAACATCGCACGGCACCCTAACAGGCCAACTTTCCTTGATCATGTCTTTAACATAACTGAAAAGTTTGTTGAACTCCATGGTGATCGGGCAGGTTATGATGATCCTGCTATTGTTACTGGTCTAGGGACTATAGATGGTAGAAGCTACATGTTCATCGGTCACCAAAAGGGTAGAAATACTAAAGAAAACATTCAGCGTAACTTTGGGATGCCAACTCCTCATGGTTACAGGAAGGCCCTTCGCTTGATGGAATATGCAGATCATCATGGGTTCCCCATAGTTACTTTCATTGACACGCCTGGGGCATATGCTGACCTTAAATCAGAGGAACTAGGACAGGGTGAAGCGATTGCTCACAATTTGAGATCCATGTTTGGTCTGAAGGTGCCAGTTATATCTATAGTTATTGGAGAAGGTGGTTCGGGTGGTGCCCTAGCCATTGGATGTGCTAATAAATTACTCATGCTTGAAAATGCTGTTTTTTATGTTGCCAGTCCAGAGGCATGTGCAGCAATCTTGTGGAAGACCGCCAAAGCTTCTCCAAAGGCTGCTGAGAAATTGAAGATTACTGCCACTGAATTGTGCAAATTACAAATTGCAGACGGTGTTATACCTGAGCCACTTGGTGGTGCACATGCAGATCCAGAGTGGACCTCTCAACAGATAAAAAAGGCTATCAAAGAAACCATGGATGAACTCACGAAGATGAACACAGAAGAACTGTTAAAACATCGCATGCTTAAGTTCAGAAAGATTGGTGGATTCCAGGAAGGTATTCCTATAGATCCCAAGAGAAAAGCCAACATGAAGAAGAGGGATCTATCTATTGCT‑‑‑AAGATTCCTGAT‑‑‑‑‑‑‑‑‑GCTGAACTAGAAGTTGAGGTTGAGAAACTGAAGCAACAGGTTTTGGAAGCTAAGGAATCTTCT‑‑‑‑‑‑CCTGTTCCTCCAAAACTAGATCTGGATGAGATGCTAAAG‑‑‑‑‑‑‑‑‑‑‑‑CAACTGGCAAGGGAGGTCGGTCTAGAATACTCTGAGGCAGTTAAAGCCACGGGCTTGACAGACAGTTTGCTGAAACTAAGGGAGGAAGTTTCAAAAGCAAATGCAGAT‑‑‑‑‑‑‑‑‑AGT‑‑‑CAAATTGTTGATCCATTGCTGAAGGATAAGATAGAAAAGCTAAGGGTGGAGTTTGAACAGCAATTGCGTGCAGCTCCCAATTATGGTAGGCTGCAGAATAAGTTTAAGTATCTGAGCGAATTATGTAAAGTAAAGCTTCTTTCAGATGCAAACAAGGACAATCAGGCTGTCACCTTT‑‑‑AAGCAAGAGTTGGAGAAAAAAGTTGATAATGCCTTGAGTGATCCAAAAATAAGGGAAACATTTGAAGCATTAAAGGCTGAAATTAAAGGTGCTGGTGCATCCTCGGCAAGTGATTTGGATGACGAGTTGAAGAAGAAAATCGTTGGGTTTATGATAGAACTA‑‑‑‑‑‑‑‑‑‑‑‑‑‑‑‑‑‑‑‑‑‑‑‑‑‑‑‑‑‑‑‑‑‑‑‑‑‑‑‑‑‑‑‑‑‑‑‑‑‑‑‑‑‑‑‑‑‑‑‑‑‑‑‑‑‑‑‑‑‑‑‑‑‑‑‑‑‑‑‑‑‑‑‑‑‑‑‑‑‑‑‑‑‑‑‑‑‑‑‑‑‑‑‑‑‑‑‑‑‑‑‑‑‑‑‑‑‑‑‑AAAGAAGTAAAAGAAGTAAAAGAAGTA‑‑‑ATAGAAAATCAAATTGAAAGTTTGGTAAACTCATCGGATGATATTAAGAGCAAGATACTGCAATTGAAATTGGAGCTTCCTAAGGCTGGAGAGACGCCTGAT‑‑‑TCAGAATCAAATAATAGAATTGGTGATTTTATATTTAAAAAATCATCTAAAATT‑‑‑‑‑‑‑‑‑‑‑‑ATTATGGCCATA‑‑‑‑‑‑‑‑‑CTTCTTAGAGACATGTTTTGG‑‑‑‑‑‑‑‑‑‑‑‑‑‑‑‑‑‑‑‑‑‑‑‑‑‑‑‑‑‑‑‑‑‑‑‑‑‑‑‑‑‑‑‑‑‑‑‑‑‑‑‑‑‑‑‑‑‑‑‑‑‑‑‑‑‑‑‑‑‑‑‑‑‑‑‑‑‑‑‑‑‑‑‑‑‑‑‑‑‑‑‑‑‑‑‑‑‑‑‑‑‑‑‑‑‑‑‑‑‑‑‑‑‑‑‑‑‑‑‑‑‑‑‑‑‑‑‑‑‑‑‑‑‑‑‑‑‑‑‑‑‑‑‑‑‑‑‑‑‑‑‑‑‑‑‑‑‑‑

>Pvu_accA_Phvul.008G108700

‑‑‑‑‑‑ATGGCTGCTTCTTCT‑‑‑‑‑‑‑‑‑GCGTCTCTCGCTGGT‑‑‑‑‑‑‑‑‑GCTTCTGCTTCGGACCTTTTGAGAAGTTCCACCAGCGGTTTCAATGGCGTTCCTTTGAGAAGTTTGGGGAAGGGAAGA‑‑‑TTGGTTTTGAAGAGT‑‑‑AGAGATTTTAGTGTTGCT‑‑‑‑‑‑GCCAGGCTGAGGAAGCCGAAGAAGTTTGACAATCCTTGGCCTGCTAATCCTGATCCTAATGTGAAAGGTGGG‑‑‑‑‑‑GTGTTGTCCTACCTTGCCCGTTTCAAGCCACTCGAGGAAAAGCCAAGG‑‑‑CGTGTCATTTTGGATTTTGAAAAGCCTCTTGCTGATATGCAAAAGAAGATCAATGATATACAAAACATGGCAAACGAAACTGGGCTGGACTTCACTGATCAGATTCAATTATTGGAGACTAAGCACAAGCAGGCTTTAAAGGATCTCTATAAGCATCTGACTCCTATACAGCGGGTCAAAATTGCACGACATCCTAACAGGCCAACTTTTCTTGATCACCTCTATAACATAACTGAAAAGTTTATGGAAATCCATGGTGATCGTTTAGGTTATGATGATCCAGCTATTACTACTGGTATAGGGATGATAGATGGTAGAAGATACATGTTCATTGGTCAGCAAAAGGGTAGAAATACTAAAGAAAACATTCAGTGTAACTTTGGGATGTCAACTCCTCATGGTTACAGGAAGGCTCTTCGTATGATGAGATATGCAGATCATCATGGGTTCCCCATAGTTACATTCATTGATACACCTGGTGCTTACGCTGACCTTGAATCAGAGCATAAAGGCCAAGGTGAAGCAATTGCTCAGAATTTGAGAACCATGTTTGGTCTGAAGGTTCCAGTATTATCTATAGTTATCGGAGAAGGTGGTTCAGGTGGTGCCCTAGGCATTGGATGTGCTAATAAACTACTGATGCTTGAAAATGCTGTTTTTTATGTTGCAAGTCCAGAAGCATGTGCAGCAATCTTGTGGAAGAATTCTAAAGCGGCTCCAGAGGCTGCCGAGAACCTGAGGATTACAGCTCCTGAATTGTGCAAATTAGATGTTGTAGATGGTATTATACCTGAGCCGATTGGTGGTGCACATTCTGATCCAGAGTGGACATCTCAACAGATAAAAAAAGCAATCAATGAAACCATGGACGAACTCACCAAGATGAGCGCTGAAGAGCTTCTAGAACATCGCAGGCTTAAGTTCAGAAAGATCGGTCAGTTCCAGGAAGGTATTCCTATAGATCCTATTCGAAAAGTCAAGATGAAGAAGAAGGATCTATCTATTGCT‑‑‑AAGATTCCTGAT‑‑‑‑‑‑‑‑‑GCTGAACTGGAAGTTGAGGTTGAGAAACTGAAGAAACAAATTTTGGAAGCTAAGAAATCTTCT‑‑‑‑‑‑CCCATTCCTCCAAAACTAGAGTTAGATGAAATGTTAAAG‑‑‑‑‑‑‑‑‑‑‑‑CAACTGGAGAGGGAGATCAATCAAGAATACTCTGAGGCAATTAAAGCTGCTGGCTTGACAGAGAGGTTGTCGCAACTACAGGAGGAAGCCTCAGAAGCAAATGCAGAT‑‑‑‑‑‑‑‑‑TAT‑‑‑GAAAGTCTTGATCCATCACTGAAGGATAAGGTAGAAAAGCTAAGGGTGGAGTTCGAAAAACAATTACGTGCATCTCCTAATTATGTTAAGCTGCAGAAAAATCTTAAGTATTTCAATGAATTATCTGAAGCAAAGCGTCTGTCAGATGTAAGCAAG‑‑‑‑‑‑GAGAACACTGTCCTATTTGAGCAAGAGTTGAAGATGAAAGTTGATCAAATCTTGAGTGATCCTAGATTAAGGGAAGAGTTTGAAGCATTAAAGGCTGAACTTACAGGTGTTGATGCATCCTCAGAAAGTGATTTGGATGAGGAGTTGAAGAAGAAAATCATTGACTTTAAGAAAGAGTTAGACTCACAGATGGTTAATGCTCTGCAGTCGATGGGCTTCAATATTGCTTTAGAAGAAGAAGATAACACTGACCAAGGTATAGGCTTCAATATTGCTTTAAAAGAAGAAGATAACACTGACCAAGGTATAATAGAACTAAATGAAGAA‑‑‑ATAGGAAAGCAAATTGATAGTTTGGTTAACTCATCGGATGATATTAAGAGCAAGATACAACAATTGAAATTGGAGGTTGCCAAGGCTAGAAATTCGCCAACT‑‑‑TCTGAATCAAAGAGTAGAATTGAGGCTTTGGTGCAACAAATTAAGCAGGATCTC‑‑‑‑‑‑‑‑‑‑‑‑GTAGAGGGCATTGAATCGTCTGGCTTAAAAGAACAGTATGAACATCTGCTGTCAGATGGAAGTTTT‑‑‑‑‑‑‑‑‑‑‑‑‑‑‑‑‑‑‑‑‑‑‑‑‑‑‑‑‑‑‑‑‑‑‑‑‑‑‑‑‑‑‑‑‑‑‑‑GATTCAGCTGCGAACAGGCTCACCGAAGAGGAG‑‑‑‑‑‑‑‑‑‑‑‑‑‑‑‑‑‑‑‑‑‑‑‑‑‑‑‑‑‑‑‑‑‑‑‑‑‑‑‑‑‑‑‑‑‑‑‑‑‑‑‑‑‑

>Vra_accA_Vradi06g10650

‑‑‑‑‑‑ATGGCTGCTTCTTCT‑‑‑‑‑‑‑‑‑GCGTCTCTCGCTGGT‑‑‑‑‑‑‑‑‑GCTTCTGCTTCGGACCTTTTGAGAAGTTCAACGAGCGGTTTCAGTGGTGTTCCTTTGAGAAGCTTGGGCAAGGGAAGA‑‑‑TTGGTTGTAAAGAGT‑‑‑AGGGATTTTAGTGTTGCT‑‑‑‑‑‑GCCAAGCTGAGGAAGGTGAAGAAGCATGACTATCCTTGGCCCGCTAATCCTGATCCCAATGTGAAAGGTGGG‑‑‑‑‑‑GTGCTGACCCACCTTTCCCGTTTCAAGCCACTCAAGGAAAAGCCAAAG‑‑‑CCTGCCACTTTGGATTTTGAAAAGCCTCTTGTTGATCTGCAAAAGAAGATCTTTGATGTACAAAAGATGGCAGACGAAACTGGTCTGGACTTCACTGATCAGATTCAATTATTGGAGACTAAGTACCAGCAGGCTTTAAAGGATCTCTATACGCATCTGACTCCTATACAGCGGGTCAACATCGCACGACATCCTAACAGGCCAACTTTTCTTGATCACATCTTTAACATAACTGAAAAGTTTATGGAAATCCATGGTGATCGGGCAGGTTATGATGATCCAGCTATTGTTACTGGTATAGGGTTGATAGATGGTAGAAGATACATGTTCATTGGTCACCAAAAGGGTAGAAATACTAAAGAAAATATTCAGCGTAACTTTGGGATGCCAACTCCTCATGGTTACAGGAAGGCTCTTCGTTTGATGGAATATGCAGATCATCATGGGTTCCCCATAGTTACATTCATTGACACACCTGGTGCTTACGCTGACCTTAAATCAGAGGAACTAGGACAAGGTGAAGCAATTGCTCACAATTTGAGATCCATGTTTGGTCTGAAGGTTCCAGTTATATCTATAGTTATTGGAGAAGGTGGTTCAGGTGGTGCCCTTGCGATTGGATGTGCTAATAAACTACTGATGCTTGAAAATGCTGTTTTTTATGTTGCAAGTCCAGAAGCATGTGCAGCAATCTTGTGGAAGAGTGCCAAAGCTGCTCCAAAGGCTGCTGAGAAGCTGAGGATTACAGCACCTGAATTGTGCAAATTACAAGTTGCAGATGGTATTATACCTGAGCCGCTTGGTGGTGCACATGCAAATCCAGAGTGGACATCTCAACAGATAAAGAAAGCAATCAATGACACCATGGACGAACTTACCAAGATGGACACTGACGAGCTTATAAAACATCGCATGCTTAAGTTTAGAAAGATCGGTGGGTTCCAGGAAGGTGTTCCTATAGATCCTAAGAGAAAAGTCAACATGAGGAAGAAGGATCTTTCAATTACT‑‑‑AAGATGCCCGAT‑‑‑‑‑‑‑‑‑GCTGAACTGCAAGCTGAGGTTCAGAAACTGAAGAAACAAATTTTGGAAGCTAAGGAATCTTCT‑‑‑‑‑‑CCCATTCCTCCAAAACTAGATTTAGATGAGATGTTAAAG‑‑‑‑‑‑‑‑‑‑‑‑CAACTGGAGAAGGAGATCAATCAAGAATACACTGAGGCAATTAAAGCTGCAGGCTTGACAGAGAGGTTGATGCAACTACAGGAGGAAGTCTCAGCAGCAAATGCAGAT‑‑‑‑‑‑‑‑‑TAT‑‑‑GAAAGTCTTGATCCATCGCTGAAGGATAAGGTATATAAGCTAAGGGTGGAGTTTGAAAAGAAATTGCGTGCATATCCTAATCATGGTAGGCTGCAGAAATATCTTAAGTATTTCAATGAATTATCTGAAGCAAAGCAGTTGTCAGGTGTAAGCAAG‑‑‑‑‑‑GACACTGACACATTT‑‑‑AAGCAAGAGTTGAAGACGACACTTGGTCAAGTCTTGAGTAATCCGAAATTAAAGAAAGAGTTTGAAGCATTAAAGGATGAAATTAGAAGTGTTGGTGCATCCTCAGGAAGTGATTTGGATGAGGAGTTGAAGAAGAAAATCACTGACTTTAAGAAAGAGGTAGACTCACAGATGGTTACTGCTCTGAAGTCGATGGGCTTAGATGTTGCTGTAAAACAAGATAAGGCCACCGAC‑‑‑‑‑‑‑‑‑‑‑‑‑‑‑‑‑‑‑‑‑‑‑‑‑‑‑‑‑‑‑‑‑‑‑‑‑‑‑‑‑‑‑‑‑‑‑‑CCAGGTACAGAAGAACTAAAAGAAGAA‑‑‑ATAGTGAAGCAAATTGATAGTTTGGTTAACTCATCGGAAGATATTAAGAGCAAGATGCAACTACTGAAATCGGAGGTTGCCAAGGCTGGAAAGTCACCAAGT‑‑‑TCTGAATCAGTGAATAGAATTGATGCTTTGGTGCAACAAATTAGGCGGAACCTT‑‑‑‑‑‑‑‑‑‑‑‑GCAGAGTTCATTGACTCGTCTGGCTTAAAAGAAGAGTTTGAAGCTCTGCTGTCTAAT‑‑‑‑‑‑‑‑‑‑‑‑‑‑‑‑‑‑‑‑‑‑‑‑‑‑‑‑‑‑‑‑‑‑‑‑‑‑‑GGAAGTCTT‑‑‑‑‑‑‑‑‑GATTCAGCTGCAGACAGGGTCACCGGTGAGGAGGCGAGAGAGAAAGTTGGTGCAAACTCTACCATTTCT‑‑‑‑‑‑‑‑‑‑‑‑‑‑‑‑‑‑

>Mtr_accA_Medtr7g066870

‑‑‑‑‑‑ATGGCTTCCTCTTCT‑‑‑‑‑‑‑‑‑GCAACTCTTGCTAGT‑‑‑‑‑‑‑‑‑TCTGCTGCTTCTGATCTTCTGAGGAGTTCGACTGGCGGTTTCACTGGTGTTCCTTTGAGAACCTTGGGAAGGACAGGG‑‑‑CTGGTTTTGAAAAGA‑‑‑AGGGACTTAACAGTTAGTGTTACTGCTAAGTTGAGGAAGGTGAAGAAGAAAGAGTATCCATGGTCTGGTAACCTTGATCCCAATGTTAAAGGTGGG‑‑‑‑‑‑GTGTTGACTCATCTCTCTCCTTTCAAGCCACTCAAAGAGAAGCCAAAG‑‑‑CCGGTCATTTTGGATTTCGAAAAGCCTCTTCTTGATCTGGAAAAGAAGATCAGCGATGTTCGGAAGATGGCAGATGAAACTGGTTTGGATTTCAGTGATCAGATTTCCTCATTGGAGAATAAGTACCAAAAGGCTTTAAAGAATCTATATACACATTTAACTCCTATACAGAGGGTCAACATTGCACGACATCCTCACAGGCCAACTTGCCTTGATCACATCTTTAACATAACTGATAAGTTTATGGAACTCCATGGTGATCGAAATGGTTATGATGATCCTGCTATTGTTACTGGTTTAGGGTCTATAGATGGTAGGACGTACATGTTTATAGGCCACCAAAAGGGTAGAAATACTAAAGAAAGTATTAACCGTAACTTTGGGATGCCAACTCCACATGGTTATAGGAAAGCTCTACGCATGATGGAATATGCAGATCATCATGGATTCCCAGTAGTTACTTTCATTGATACACCTGGGGCATATGCTGACCTCAAATCTGAGGAACTTAACCAAGGTGAAGCAATTGCTCATAATTTGAGATCCATGTTTGGTCTGAAGGTGCCGGTTATTTCTATAGTTATTGGGGAAGGTGGATCTGGTGGTGCCCTTGCCATTGGATGCGCTAATAAATTACTCATGCTTGAAAATGGAGTGTTCTATGTTGCCAGTCCAGAGGCGTGTGCTGCAATCTTGTGGAAGAGTTCTAAAGCTGCTCCCAAGGCTGCTAAAAAACTGAAGATTACAGCGACTGAATTGTGCAAATTGAAAGTTGCAGATGGAGTTATACCTGAGCCCCTCGGTGGTGCACATGTTGATCCAAGTTGGACCTCTCAACAGATAAAAATTGCAATCAATAAAGCTATGGATGAACTCACTAAGATGAACACAGAAGACCTGTTAAAACATCGCATGCTTAAGTTCCGAAAACTCGGTGGGTTCAAGGAAGGAATTCCT‑‑‑‑‑‑‑‑‑‑‑‑‑‑‑‑‑‑‑‑‑‑‑‑‑‑‑‑‑‑‑‑‑‑‑‑‑‑‑‑‑‑‑‑‑‑‑‑‑‑‑‑‑‑‑‑‑‑‑‑‑‑‑‑‑‑‑‑‑‑‑‑‑‑‑‑‑‑‑‑‑‑‑‑GAAGTTCAGATTGAGAAGCTGAAGCAACAAATTTTGAAG‑‑‑‑‑‑‑‑‑TCCTCT‑‑‑‑‑‑TCTCAGCCTCCGAAACTCAATTTAGATAAGACGATAAAGAAACTGGAAGAGAAACTGGAAAAGGAGGTTGATCAAGAATTGTCTGAGGCTGCTAAAGCCTTGGGCTTAACACAAAGTTTGTCGAAACTACGTGATGAATTTTCAAAAGCAAGTTCAGAT‑‑‑‑‑‑‑‑‑GAC‑‑‑CAACCTCTTGATCCATTGCTGAAGGGTAAGATAGAAAAACTACAGGCAGATTTTAATCGAAGGTTGTCAGCAGCTCCTAATGCTAACAAGCTGAAGAAGAAGCATGGCATGTTGAAAGAATTGACTAAAGTGAAGCTTCTTTCAAAAATAAACAAG‑‑‑‑‑‑GAGGCCGCAACACTT‑‑‑ACGCAAGAACTGAAGAAGAAATTTGATGATGTCATGAATAATCCTAGAATAAAGGAAAACTATGAGGCATTACAGTCTGAAATCCAGCGA‑‑‑‑‑‑GCATCCTCAGCAAGCGATTTGGATGATGAATTGAAGAAGAAAATCATTGAGTTCAATAATGAGGTAGACTTGCAGGTGGCTAATGCTGTGAAGTCGGTCGGGTTAGATGTTCAGTTTGTAAAACCAGGACAAGATGGCAACAAGTTT‑‑‑‑‑‑‑‑‑‑‑‑‑‑‑‑‑‑‑‑‑‑‑‑‑‑‑‑‑‑‑‑‑TTGGTGCGTGAGATAGAAGAACTAAACAAGGAT‑‑‑GTGAAAAAGGAAATTGATGTTTTGGCAAACTCGTCGACAGATATTAAGAGAATGATAGAGCAATTGAAAGTGGAGGTCGCCAAAGCTGGAGGGACACCAAAT‑‑‑TCGGAATCGAAGAATAGAATTGTTGCTTTGACGCAAAAAATAAAGCGGAGCCTT‑‑‑‑‑‑‑‑‑‑‑‑GCAGAGGCCGTAGGCTCTTCTAGCTTGAAAGAGAGATATGAAAACCTGATGTCTGGAACCTCTGGG‑‑‑‑‑‑‑‑‑‑‑‑‑‑‑‑‑‑‑‑‑‑‑‑‑‑‑GATGGAAGTTTGAAA‑‑‑‑‑‑‑‑‑‑‑‑GTAGGAAATGGTCTCACCGATGATGAGTTGAGAGAGAAAGTTGGTGCAAATTGCTCCTTTTCT‑‑‑‑‑‑‑‑‑‑‑‑‑‑‑‑‑‑

>Bto_accA1_JETM‑2027321

‑‑‑‑‑‑ATGGCTTCTGTATCTCATATCCCAGTTGGACTTGGTGGA‑‑‑‑‑‑‑‑‑ACTTCGGCTTCAGATTTTCTTCGGAGTTCAACTAATGGTGTCAGTGGTTTCGCTTTGAAAGCCTTAGGACGGGCTAGA‑‑‑TTAGGTTTGAAAAGA‑‑‑AGGGATTTTACAGTTGCT‑‑‑‑‑‑GCCAGGCTGAGGAAAGTGAAGAAGCGCGAGCATCCCTGGCCTGATAATCCTAAT‑‑‑‑‑‑‑‑‑‑‑‑‑‑‑‑‑‑‑‑‑‑‑‑‑‑‑CTTACTCATTTTTCATCTTTCCAGCCGCTGAAAGAGAAGCCAAAG‑‑‑CCAGTCATTTTGGATTTTGAAAGACCACTTGTTGAACTAGAAAAGAAGATCAGCGATGTGCGGAAGATGGCAAATGAAACTGGTCTGGACTTTAGTGATCAGATTACCTCACTAGAGAATAAGTACCATCAGGCTTTAAAGTATTTATATACGCATCTGACTCCAATACAGCGGGTCTATATTGCACGGCATCCTAACAGACCTACTTTTCTTGACCATATCTTCAACATAACTGAAAAGTTTGTGGAACTTCATGGTGATCGGGCGGGTTATGATGATCCTGCTATTGTTACTGGTATAGGGACCGTAGACGGTAGGAGATACATGTTCATGGGTCACCAAAAGGGCAGAAACACCAAGGAAAACATTAAGCGTAACTTTGGGATGCCTACTCCTCATGGTTACAGGAAGGCTCTTCGCATGATGCGTTATGCAGATCATCATGGATTCCCCATAGTTACTTTCATTGACACACCTGGGGCATATGCTGACCTTAAATCTGAGGAACTAGGCCAAGGTGAAGCCATTGCTCACAATTTGAGGGAAATGTTTGGTCTGAAAGTGCCAATTATTTCTATTGTCATTGGAGAAGGTGGTTCTGGTGGTGCCCTTGCCATTGGCTGTGCTAACAAGTTACTTATGCTTGAAAATGCAGTTTTCTATGTTGCCAGTCCAGAAGCATGTGCAGCAATCTTGTGGAAGAGTGCTAAAGCTGCTCCAAAGGCTGCTAAGAAACTGAAGATTACAGCATCAGCATTGTACAGATTGCAAGTTTCCGATGGTGTTATCCCTGAGCCACTTGGTGGTGCACATGCAAATCCTGCTTGGACATCACAACAGATAAAGTTTGCAATCAATAAAGCCATGGATGAGCTTGAGGCAATGGAAACAGAAGACCTCCTAAGACATCGCAAGATTAAGTTCCGCAAATTGGGTGGATTACAAGAAGGTGGTCCTATAGATCCTAAGAAGATGGTCAATATGAAGAAGAGAGATATACCTGTTGCT‑‑‑‑‑‑‑‑‑‑‑‑‑‑‑‑‑‑‑‑‑‑‑‑‑‑‑‑‑‑‑‑‑‑‑‑‑‑‑‑‑‑‑‑‑‑‑‑‑‑‑‑‑‑AAGCAGAAAATTTTGAACGCAAAGGAATCCTCC‑‑‑‑‑‑ATCAAAGCTCCAAAATCAGATCTTAATGTGAGGATACAG‑‑‑‑‑‑‑‑‑‑‑‑CAGTTAAAAAAAGTAGTTGACAAAGAGTACTTTGAGGCAGTTAAAGCCATTGGCTTGTCAGACAAGTTGGAGAAGATACGGGAGGAATTTTCAAAAGCAAATTCGGAG‑‑‑‑‑‑‑‑‑GAC‑‑‑CAACTTATTGATCCAGCTCTGAAGGATAGGATGGAAAAGCTATCTGAGGAGATCAACCAAGGTCTGTCAGCAGCTCCCAATTATGGAAGGCTGCAGGATAAGCTTCACAATTTGAAAGAATTATCAAAAGCCAAGTTTCTGTCAGAAAAGGAAAAG‑‑‑‑‑‑CGTGCTGCTACACTG‑‑‑AAGCAAGAGTTTGAGGCAAAGTTTGAAGACGTCATTAATAACCCAAGTATAAAGAAGAAATATGAGGCAATAAAAGCAGAAATTGCGGGTTCTGGTGTGTCCTCTATGAGTGATTTGGACGCTGAATTGATGAATAAAGTCATTGAATTTGAAAAAGAAGTAGAGCTTCATTTAGCTGATGCTGTGAAGTCCTCAGGCTTAGACGTAGACATTGTAAAAGCAAAGGTGCCGGGCAATATTAAGGAG‑‑‑‑‑‑‑‑‑‑‑‑‑‑‑‑‑‑‑‑‑‑‑‑‑‑‑‑‑‑‑‑‑‑‑‑‑‑‑TCATTGTCAAAGTTGAACAAAGAA‑‑‑ATAAATGAGGAAATTGAAACAGTGCTGAAGTCATCAGAT‑‑‑CTGAAGAGCAAGATAGAACTTTTGAGAATAGAGGTTGCTAAGGCAGGACAAACACCTGAC‑‑‑TTGAAATCAAAAAATAGAATTGCTGCTTTGGAGCAACAAATTAAGCAGAACCTT‑‑‑‑‑‑‑‑‑‑‑‑GAAGACGCTATTGATTCTGCTAGGTTAAAACAGAAGTATGAGAATGTAGCATCTAAAATTTCCAACAATGCTGTGTCT‑‑‑TCTGGAGACGCAGACGGAAGTCTAAACAAG‑‑‑‑‑‑‑‑‑‑‑‑‑‑‑‑‑‑‑‑‑‑‑‑‑‑‑AAAGATGAGCTGAGAGTGGAGGTTGGCGCAAATCGCATCTTTACT‑‑‑‑‑‑‑‑‑‑‑‑‑‑‑‑‑‑

>Ccan_accA_RKFX‑2008617

‑‑‑‑‑‑‑‑‑‑‑‑‑‑‑‑‑‑‑‑‑‑‑‑‑‑‑‑‑‑GTTGGACTTGGTGGA‑‑‑‑‑‑‑‑‑ACTTCGGCTTCAGATTTTCTTCGGAGTTCAACTAATGGTGTCAGTGGTTTCCCTTTGAAAGCCTTAGGAAGGGCAAGA‑‑‑TTAGGTTTGAAAAGA‑‑‑AGGGATTTTACAGTCGCT‑‑‑‑‑‑GCCAAGCTGAGGAAGGTGAAGAAGCACGAGTATCCCTGGCCTGATGATCCTGATCCCAATGTTAAAGGTGGA‑‑‑‑‑‑GTCCTAACTCATCTTTCGCCTTTCAAGCCACTGAAAGAGAAGCCAAAG‑‑‑CCAGTTACCTTGGAATTTGAAAGACCACTTCTTCAGCTAGAAAAGAAGATCATCGATGTGCGCAAGATGGCAAACGAAACTGGTCTGGACTTTAGTGATCAGATTATCTCATTAGAGAATAAGTACCAACAGGCTTTAAAGGATTTATATTTGCGTCTGACTCCAATACAGCGGGTCAATATTGCACGGCATCCTAACAGACCTACTTTCCTTGATCATGTCTTCAACATAACTGAAAAGTTTGTGGAACTTCATGGTGATCGGGCAGGTTATGATGATCCTGCTATTGTTACTGGTATAGGGACCATAGACGGTAGGAGATACATGTTCATGGGTCACCAAAAGGGCAGAAACACCAAGGAAAACATTCAGCGTAACTTTGGGATGCCCACTCCACATGGTTACAGGAAGGCTCTTCGCATGATGTATTATGCAGATCATCATGGATTCCCCATAATTACTTTCATTGACACTCCTGGGGCATATGCTGACCTTAAATCTGAGGAACTAGGCCAAGGTGAAGCCATTGCTCACAATTTGAGGACAATGTTTGGTCTGAAAGTGCCCATTGTTTCTATTGTCATTGGGGAC‑‑‑‑‑‑‑‑‑‑‑‑‑‑‑‑‑‑‑‑‑‑‑‑‑‑‑‑‑‑‑‑‑‑‑‑‑‑‑‑‑‑‑‑‑‑‑‑TTTCAGACCAAACATTTTTTCTATGTCGCCAGTCCTGAAGCATGTGCAGCAATCTTGTGGAAGACTGCTAAAGCTGCTCCACAGGCTGCTGAGAAACTGAAGATTACAGCAACAGAGTTGCGCAAATTGCAAATTGCAGATGGTGTTGTCCCTGAGCCACTTGGTGGTGCACATGCAGATCCAACTTGGACCTCGCAACAGATAAAATTTGCAATCAATGAAGCCATGGATGAGCTTGGGGCAATGGAAACAGAAGACCTACTAAGGCATCGCATGCTTAAGTTTCGCAAAATTGGTGGCTTCCAAGAAGGTATTCCTATAGATCCAAAGAGGAAAGTCAACATGAAGAAGAGGGAGCAACCCACTGCTAAGAAAATTCCAGAT‑‑‑‑‑‑‑‑‑GCTGAACTAGCGGGTGAGGTTGAGAAACTGAAGCAGCAAATTTTGAAAGCAAAGGAATCCTCC‑‑‑‑‑‑ATCAAACCTCCAGAATCAGATCTG‑‑‑‑‑‑ATGATACAG‑‑‑‑‑‑‑‑‑‑‑‑AAATTGAAAAGAGAAGTTGACAAAGAATACTTTGAGGCAGTTAAAGCCATCGGCTTGACAGACAGGTTGGTGAAGTTACGGGAGGAATTTTCAAAAGCAAATTCGGAG‑‑‑‑‑‑‑‑‑GAC‑‑‑CAACTTATTCATCCAGCACTGAAGGACAAGATTGAAAAGCTACATGAGGAGATCAACCAAGGTCTGTCAGCAGCTCCCAATTATGGAAGGCTGCAGGATAAGCTTGACATGTTGAAAGAATTATCAAAAGCCAAGTTTCTGTCAGAAAAGAATAAG‑‑‑‑‑‑CATGCTGCTACACTG‑‑‑AAGCAAGAATTTGAGGCAAAATTTGAAGACATCATTAATAACCCTAGTATAAAGAAGAAATATGAGGCATTAAAATCAGAAATTGCAGGTTCTGGTGTGTCCTCACTGAGTGATTTGGATGATGAATTGAGGAATAAAATCATCGAATTTAAGAAAGAAGTAGAGCTTCAGTTAGCTGATGCTGTGAAGTCT‑‑‑GGCTTAGATGTTGTCATTGTAAAAAAAAAGGTACCGGACAGCATTAAGGAG‑‑‑‑‑‑‑‑‑TTAGTATCAGAG‑‑‑‑‑‑‑‑‑TTTAAGTCAAATCTAGCAGAATTGAACAAAGAA‑‑‑ATAAATGAGGGAATTGAAACTGCACTGAACTCATCAGAT‑‑‑CTCAAGAGCAAGATAGAACTACTGAAAATAGAGGTTGCCAAGGCAGGAAAAACGCCTGAC‑‑‑TTGAAATCAAAAAATAGAATTGCTGCTTTGGAGCAACAAATTAAGCGGAGCCTT‑‑‑‑‑‑‑‑‑‑‑‑GAAGAGGCGATTGATGCTTCTAGCTTAAAAGAGAAGTATGAGAATCTAAAATCCAAAATTTCCAGCAATGATGAATCT‑‑‑TCTGGAGATTTAGATGGAAGTTTAAACAAG‑‑‑‑‑‑‑‑‑‑‑‑‑‑‑‑‑‑‑‑‑‑‑‑‑‑‑GAAGATGAATTGAGAGTGGAGGTTGGCGCAAACCGCACCTTTGCT‑‑‑‑‑‑‑‑‑‑‑‑‑‑‑‑‑‑

>Cof_accA1_RKLL‑2017992

‑‑‑‑‑‑ATGGCTTCTGTATCCCACTCCCCAGTTGCATTTGCTGGAAGA‑‑‑‑‑‑ACTTCAGCTTCGGATCTTCTCCAGAGTTCCGCTAATAGCATCAGTGGTGTTCCTTTAAGGACTTTAGGAAGAGTGCGA‑‑‑TTGGGTTTGAAAAGA‑‑‑AGGGATCACACGGTTGTC‑‑‑‑‑‑GCAAAGCTTAGAAAGGTGAAGAAGCATGAGTATCCCTGGCCTGATGATCCTGATCCTAATGTTAAGGGGGGA‑‑‑‑‑‑GTGCTAAGCCATCTTTCGCCTTTCAAGCCACTTAAAGAGAAGCAAAAG‑‑‑CCGGTCACTTTGGATTTTGAGAAGCCACTTGTTGAATTAGAAAAGAAGATCATTGATGTGCGGAAGATGGCAACTGAAACGGGCTTGGACTTCAGCGATCAGATTATCTCATTGGAGAATAAGTACCAACAGGCTTTAAAGGATTTATATACACGCCTGACTCCTATACAGCGGGTCAACATTGCTCGGCATCCTAACAGGCCTACTTTCCTTGATCATATATTCAACATAACTGAGAAGTTTGTGGAACTTCACGGTGATAGGGCAGGGTACGATGATCCTGCCATTGTTACTGGTATAGGGACCATAGATGATAGAAGATACATGTTCATGGGTCATCAGAAGGGTAGAAATACTAAAGAAAATATTCAGCGTAACTTTGGGATGCCAACTCCCCATGGTTACAGGAAGGCTCTCCGCATGATGTATTATGCTGATCATCATGGGTTCCCTATTATTACTTTCATTGACACGCCTGGGGCATATGCTGACCTAAAATCTGAGGAATTAGGCCAAGGTGAAGCCATTGCTCACAACTTGAGGACAATGTTTGGCCTTAAGGTGCCAATTATTTCTATAGTTATTGGTGAAGGTGGTTCTGGTGGTGCCCTTGCCATTGGCTGTGCTAATAAATTATTAATGCTTGAAAATGCAGTTTTTTATGTTGCCAGTCCAGAAGCATGTGCAGCGATCTTGTGGAAGAGTGCTAAAGCTTCTCCACAGGCTGCTGAGAAACTCAAGATTACAGCCTCTGAGTTGTGCAAACTGCAAATTGCAGATAGTATAATCCCTGAGCCACTTGGTGGTGCACATGCAGATCCATCTTGGACCTCACAGCAGATAAAACTGGCAATCAATGAATACATGGATGAGCTCACAAGCATGAGCACAGAAGACCTACTAAGGCATCGGTATCTCAAGTTCCGGAAAATTGGTGGGTTCCAGGAGGGAATTCCTATAGATCCTAAGAGGACAGTCAACATGAAGAAGAAGGAAGCACCTATAGCTAAGAGGATTCCAGAT‑‑‑‑‑‑‑‑‑GCTGAATTAGAGGGGGAGGTTGAGAAACTGAAACAGCAAATTTTGAAAGCCATGGAAACTTCA‑‑‑‑‑‑AGCAAGACTTCAGAGCTGGGGCTGAATGAGATGATACAG‑‑‑‑‑‑‑‑‑‑‑‑AAACTGAAAAGGGAGGTTGATCAAGAGTACTCTCAAGCAGTGAAAGCCATGGGCTTGCAAGACAGGTTGACAGAGCTACGAGAGGAATTTAAAAAAGTAGAGTCAGAG‑‑‑‑‑‑‑‑‑GGC‑‑‑CAACTTATCCATCCAGCACTGAAGGATAAGATGGAAAAGCTAGAGGAGAAGTTCAAGCAGGGTTTGCCTTCAGCTCCCAATTATGAAAGCCTTCAAAATAAGCTTGACATGTTGAAAGAATTATCCAAAGTCAAATTTATATTAGATGGGAATAAG‑‑‑‑‑‑AAGGCTGCCACTCTG‑‑‑AAGCAAACTTTGAAGGCAAAATTTGAAGATGTCATTAATAACCCTAGTTTCAAGGAGAAATATGAGGCATTGAAGGCCGAAATTGCAAGTTCTGGTGTGTCCTCACCAGGTGATTTGGACAATGAGTTTAAGAAGAAAATCTTTGAGTTTAAAAAAGAAGCAAGGTTGCAGCTGGTTGATGCTTTAAAGTCACAGGGACTAGAAGTTAAGACTGCCAAAGCAAAGGCACGAGGTAGCATTGAGGAG‑‑‑‑‑‑TCTTCGGTGTCAGAG‑‑‑‑‑‑‑‑‑TTCAAGTCAAATATAGAAGAACTGAACAAAGAG‑‑‑ATCAATGAGGAAATTAAATCTGCCCTAAACCCATCTGAT‑‑‑GTCAAGAGCATGATAGAGCTCTTGAAAATAGAGGTAGCCAAGGCAGGTGAAACGCCTGAC‑‑‑TTGGCATCAAAAAATAGAATTGCTGCTTTGGAACAACAAATTAAGCATCGCCTT‑‑‑‑‑‑‑‑‑‑‑‑GCAGATGTCCTTGATAATTCTAGCTTGAAGGATAAGTTTGAGAATCTAAAATCCAAGATTTCTATCAAGGTCGAGTCT‑‑‑GCTGGACAATTAGATGGAAGTTTAAAAAACAACAACTCTATAGGAGACAATCCTGCCAATGATGAGTCAAGAACGAAGGTTGAAACAAACCGCACCTTTGTT‑‑‑‑‑‑‑‑‑‑‑‑‑‑‑‑‑‑

>Ath_accA_AT2G38040

‑‑‑‑‑‑ATGGCTTCAATATCGCATTCATCACTAGCTTTAGGAGGAGCTTCTTCTGCTTCTGCTTCAGATTACTTGCGTAGTTCGAGCAATGGTGTTAATGGGGTACCATTGAAAACCTTAGGAAGAGCAGTGTTTACTACCATCAGGAGG‑‑‑AAGGACTTGGCGGTGACA‑‑‑‑‑‑TCTCGGCTCAAGAAAGGGAAGAAATTTGAGCATCCATGGCCTGCAAATCCTGACCCGAATGTGAAAGGAGGA‑‑‑‑‑‑GTCTTGTCTTACCTGGCCGAGTTCAAACCATTGGGGGATACTCAAAAG‑‑‑CCTGTCACTTTGGATTTCGAGAAGCCACTAGTCGAATTGGAGAAGAAGATTGTTGATGTGAGGAAGATGGCGAATGAAACGGGGTTGGATTTCACTGAGCAGATTATCACTTTGGAGAACAAGTATAGACAGGCACTGAAAGATCTTTACACGCATCTTACTCCGATACAACGTGTGAACATTGCGCGCCATCCCAACCGACCTACTTTCCTTGATCATATACATAACATAACTGACAAGTTTATGGAGCTTCATGGAGACCGAGCGGGGTATGATGACCCTGCAATTGTGACGGGTATTGGAACCATAGATGGAAAACGTTACATGTTCATAGGTCACCAGAAAGGTAGAAACACCAAAGAAAATATAATGCGGAACTTTGGTATGCCTACTCCTCACGGATATAGGAAAGCACTTCGGATGATGTATTATGCAGACCATCACGGTTTTCCAATCGTGACATTTATCGACACTCCTGGAGCCTATGCAGATCTTAAATCCGAGGAACTTGGACAGGGTGAAGCGATTGCCAACAATCTGAGGACGATGTTCGGCCTGAAAGTGCCAATTCTTTCTATTGTCATTGGGGAAGGTGGTTCTGGTGGTGCCCTAGCCATTGGCTGTGCGAATAAAATGCTGATGCTCGAAAACGCAGTTTTCTATGTTGCCAGTCCAGAGGCATGTGCAGCGATCTTGTGGAAGACTTCTAAGGCTGCTCCTGAGGCTGCTGAAAAGCTTAGAATTACCTCCAAGGAGCTGGTCAAGCTTAATGTAGCTGATGGAATCATTCCTGAACCGCTTGGAGGGGCCCATGCCGATCCTTCATGGACGTCGCAGCAAATAAAGATTGCTATCAATGAAAACATGAATGAATTCGGAAAAATGAGTGGGGAGGAGCTCCTGAAACACAGGATGGCTAAGTACCGAAAGATTGGAGTGTTCATAGAGGGCGAACCAATAGAGCCAAGTAGGAAAATCAACATGAAGAAAAGGGAAGCCGTGTTCTCA‑‑‑‑‑‑‑‑‑GATAGC‑‑‑‑‑‑‑‑‑CGGAAGCTGCAGGGTGAGGTTGACAAGCTGAAGGAGCAGATTCTGAAAGCCAAGGAGACGTCTACGGAAGCCGAGCCTTCGAGTGAAGTTCTTAATGAGATGATTGAG‑‑‑‑‑‑‑‑‑‑‑‑AAACTCAAATCCGAGATAGATGACGAGTACACTGAAGCTGCAATAGCAGTAGGTTTGGAGGAGAGACTAACGGCAATGCGCGAAGAGTTCTCGAAAGCGAGTTCAGAA‑‑‑‑‑‑‑‑‑GAG‑‑‑CACCTTATGCACCCGGTTCTGATCGAGAAAATTGAGAAGCTCAAGGAAGAATTCAATACCCGTTTGACTGACGCACCTAACTACGAGAGCCTAAAATCTAAGCTTAACATGCTTAGGGACTTTTCCAGAGCCAAAGCAGCATCA‑‑‑‑‑‑‑‑‑‑‑‑‑‑‑‑‑‑GAAGCTACTTCATTG‑‑‑AAAAAGGAGATCAATAAGCGGTTCCAGGAAGCTGTAGACCGCCCAGAAATTAGAGAAAAGGTCGAGGCAATCAAAGCTGAGGTCGCGAGCTCAGGAGCTTCTTCTTTTGACGAGTTACCTGATGCACTGAAAGAAAAAGTTCTGAAGACTAAAGGGGAGGTCGAAGCAGAGATGGCGGGTGTGTTAAAGTCAATGGGTCTGGAGCTTGACGCTGTTAAACAGAATCAGAAGGATACGGCTGAGCAGATCTATGCCGCAAACGAAAAC‑‑‑‑‑‑‑‑‑CTTCAAGAAAAACTTGAAAAGCTGAACCAAGAA‑‑‑ATCACCAGCAAGATTGAGGAGGTGGTGAGGACACCAGAG‑‑‑ATCAAGAGCATGGTGGAGTTGCTGAAAGTGGAAACCGCAAAGGCGAGCAAAACGCCTGGTGTCACCGAAGCATATCAGAAAATCGAGGCACTTGAGCAGCAGATCAAGCAGAAGATT‑‑‑‑‑‑‑‑‑‑‑‑GCAGAGGCTCTGAACACGTCCGGACTGCAGGAAAAGCAAGACGAGCTCGAGAAGGAGCTTGCAGCTGCACGTGAACTAGCTGCAGAGGAATCAGACGGGAGTGTGAAGGAAGATGATGAC‑‑‑‑‑‑‑‑‑‑‑‑‑‑‑‑‑‑GATGACGAAGATAGTTCAGAATCCGGGAAATCGGAGATGGTTAACCCCAGCTTCGCC‑‑‑‑‑‑

>Adu_accA2_Aradu.Z0G82

‑‑‑‑‑‑‑‑‑‑‑‑‑‑‑‑‑‑‑‑‑‑‑‑‑‑‑‑‑‑ATGGCTATT‑‑‑‑‑‑‑‑‑‑‑‑‑‑‑‑‑‑‑‑‑‑‑‑‑‑‑‑‑‑‑‑‑‑‑‑‑‑‑‑‑‑‑‑‑‑‑‑‑‑‑‑‑‑‑‑‑‑‑‑‑‑‑‑‑‑‑‑‑‑‑‑‑‑‑TCACTGCTCACGGCGGCC‑‑‑GCCACTGCACATACC‑‑‑AAAAGA‑‑‑CAGGAAGCT‑‑‑‑‑‑GCAAAGATTAGAAAGGGAAAGAAGCATGATTATCCATGGCCTGATAAGATGGATCCAAATATCAGTAGTGGA‑‑‑‑‑‑TACTTGACTTACCTTTCTCACTTTAAGCCTCTGGCTGAGAAACCTAAA‑‑‑CCTGTCACACTTGATTTTGAGAAACCACTTGTTGATCTAGAGAAAAAGATTATAGAGGTACGTAATATGGCAGATGACACCGGCTTAGACTTCAGCAATCAAATTGAGGCTCTGGAAAGCAAATATCAACAGGCTTTGAAAGATCTATACACACACTTAACACCATTCCAACGGTTGATGATTGCGCGACATCCAAATAGGCCAACAGTTCTTGATCATATACTTAACATCACAGAGAAGTGGGTAGAGCTTCATGGCGATCGAGCAGGCTACGATGATCCAGCTATTGTGACTGGTTTAGGGACTATGGATGGTAAAAGTTACATGTTCATAGGCCATCAGAAAGGTAGGAACACAAAGGAAAATATTACTCGAAACTTTGCAATGCCAACTCCTCATGGTTACCGGAAAGCTTTGCGCATGATGAAATATGCAGATCACCATAAATTCCCTATAATTACATTTGTGGACACTCCTGGTGCCTATGCTGATCTAAAGTCTGAGGAACTTGGTCAAGGTGAAGCAATAGCCCATAATTTGAGGACAATGTTTGGTCTCAAGGTACCTATACTAACAGTTGTAACCGGCGAAGGTGGTTCAGGTGGGGCACTTGCCATTGCTTGTGCCAATAAATTGTTCATGCTTGAGAATTCTGCCTTTTATGTAGCAAGCCCTGAAGCTTGTGCTGCAATATTGTGGAAATCTTCCAAAGCTGCTCCTAAGGCTGCTGAAAAGCTAAGAATTACAGCTCAAGAACATTATAGACTTGGAATTGCTGATGGTGTTATTCCTGAACCATTAGGTGGTGCACATGTTGATCCTACTTGGACTTCTCAGCAAATTCAGCTTACACTCACTCAGGCCATGGAGGAGCTGACCAAAATGAATGAAGAAGAGTTGCTTCGCCATAGACAGCTCAAGTTCCGGTCGATTGGAGGATTCCAAGAAGGCATACCTGTGGAGCCAGAGAGGAAGAGAAACATGAAGCCGTCCGATGTAAACTCGTCC‑‑‑ACTCTTACTGAT‑‑‑‑‑‑‑‑‑‑‑‑‑‑‑ATCGAGTCAGAGCTCCAAACTCTGAGAAAGCAAATTTTAGAATCAAAGGGACCAACT‑‑‑‑‑‑‑‑‑‑‑‑‑‑‑‑‑‑GATCCTATCACGAACGAATCGATTCAG‑‑‑‑‑‑‑‑‑‑‑‑AAGCTTGTAAAAGAAGTAGATGAGGAGATCACCAAAGCAATCATTTCAATGGGGCTAGCAGAGAAAGTTCAATCAGTAAGAATGGAGTTATCTAAGAACTCA‑‑‑‑‑‑‑‑‑‑‑‑‑‑‑AAC‑‑‑CAACCTCTTAGTACTAACATGGAGGAAAAAGTGGACAGAATCATGGAAGAGATAAATATGAAGATGGCGCAGCCGGGAGCATATCTAGGACTAAAACAGAAGCTT‑‑‑‑‑‑‑‑‑AAGAAGCTAGACACTATAAATAGTTTCATAGAGATGAAAGTA‑‑‑‑‑‑AAACAAGAGAAACTA‑‑‑AGAAAAGAGCTCAACGAGAAACTCTCGGCC‑‑‑‑‑‑‑‑‑‑‑‑‑‑‑GATACCAAGGCGAAGATTGCAAGCCTGATGGATGCTCAGGAAAGG‑‑‑‑‑‑‑‑‑‑‑‑‑‑‑‑‑‑‑‑‑ATACCGGATCATGAATTGGTAGAGAAAGCAATGGAAGTTCAGAGAGAG‑‑‑‑‑‑‑‑‑‑‑‑TTGGAAGAGGTTTTGAAATCAGCTAACTTGGAAATTGTTGGAGTGATGAAGAAGAATGTGGAAACT‑‑‑‑‑‑‑‑‑‑‑‑‑‑‑‑‑‑CCACCAGCAGAT‑‑‑‑‑‑‑‑‑ATAAAACAGAAGATTGTAGAGTTGAACAATGAG‑‑‑ATCATTGGTGAAATTGATAGGGTGGTGAATGCAGAAGAAGGCCTTAAGGATCAAATTAAGGAACTGGAGAATATGATAGCACAAGGCTTGGATTCTAAGGAT‑‑‑‑‑‑‑‑‑‑‑‑‑‑‑‑‑‑‑‑‑GCTGAGAAGATGGAAGCCGGCATAAAAGAGCGAATC‑‑‑‑‑‑‑‑‑‑‑‑CTCGCTGCGTTGGATGCTGCTGGAGTGAAGGAAAAGATTGAGAGAATGAAGGAGGAGGTAGAATCTTTGTCCATGGCAGGTTTTGAGGACAAGATTGGTGTG‑‑‑‑‑‑‑‑‑‑‑‑‑‑‑‑‑‑‑‑‑GAAAATGGTAGATGC‑‑‑‑‑‑‑‑‑‑‑‑‑‑‑‑‑‑‑‑‑‑‑‑‑‑‑‑‑‑‑‑‑‑‑‑‑‑‑‑‑‑‑‑‑‑‑‑‑‑‑‑‑‑‑‑‑‑‑‑‑‑‑

>Aip_accA2_Araip.77JRH

‑‑‑‑‑‑‑‑‑‑‑‑‑‑‑‑‑‑‑‑‑‑‑‑‑‑‑‑‑‑ATGTCCGTT‑‑‑‑‑‑‑‑‑‑‑‑‑‑‑‑‑‑‑‑‑‑‑‑‑‑‑‑‑‑‑‑‑‑‑‑‑‑‑‑‑‑‑‑‑‑‑‑‑‑‑‑‑‑‑‑‑‑‑‑‑‑‑‑‑‑‑‑‑‑‑‑‑‑‑‑‑‑‑‑‑‑‑‑‑‑‑‑‑‑‑‑‑‑‑‑‑‑‑‑‑‑‑‑‑‑‑‑TAC‑‑‑GAAAGA‑‑‑AGAGAAGCT‑‑‑‑‑‑GCAAAGATTAGAAAGGGAAAGAAGCATGATTATCCATGGCCTGATAAGATGGATCCAAATATCAGTAGTGGA‑‑‑‑‑‑TACTTGACTTACCTTTCTCACTTTAAGCCTCTGGCTGAGAAACCTAAA‑‑‑CCTGTCACACTTGATTTTGAGAAACCACTTGTTGATCTAGAGAAAAAGATTATAGAGGTACGTAGTATGGCAGATGACACCGGCTTAGACTTCAGCAATCAAATTGAGGCTCTGGAGAGCAAATATCAACAGGCTTTGAAAGATCTATACACACACTTAACACCATTCCAACGGTTGATGATTGCGCGACATCCAAATAGGCCAACAGTTCTTGATCATATACTTAACATCACAGAGAAGTGGGTAGAGCTTCATGGCGATCGAGCAGGCTATGATGATCCAGCTATTGTGACTGGTTTAGGGACTATGGATGGTAAAAGTTACATGTTCATAGGCCATCAGAAAGGTAGGAACACAAAGGAAAATATTACTCGAAACTTTGCAATGCCAACTCCTCATGGTTACCGGAAAGCTTTGCGCATGATGAAATATGCAGATCACCATAAATTCCCTATAATTACATTTGTGGACACTCCTGGTGCCTATGCTGATCTAAAGTCTGAGGAACTTGGTCAAGGTGAAGCAATAGCCCATAATTTGAGGACAATGTTTGGTCTCAAGGTACCTATACTAACAGTTGTAACCGGCGAAGGTGGTTCAGGTGGGGCACTTGCCATTGCTTGTGCCAATAAATTGTTCATGCTTGAGAATTCTGCCTTTTATGTAGCAAGCCCTGAAGCTTGTGCTGCAATATTGTGGAAGTCTTCCAAAGCTGCTCCTAAGGCTGCTGAAAAGCTAAGAATTACAGCTCAAGAACATTATAGACTTGGAATTGCTGATGGTGTTATTCCTGAACCATTAGGTGGTGCACATGTTGATCCTACTTGGACTTCTCAGCAAATTAAGCTTACACTCACTCAGGCCATGGAGGAGCTGACCAAAATGAATGAAGAAGAGTTGTTTCGCCATAGACATCTCAAGTTCCGGTCGATTGGAGGATTCCAAGAAGGCATACCTGTGGAGCCAGAGAGGAAGAGAAACATGAAGCCGTCCGATGTAAACTCGTCC‑‑‑ACTCTTACTGAT‑‑‑‑‑‑‑‑‑‑‑‑‑‑‑ATCGAGTCAGAGCTCCAAACTCTGAGAAAGCAAATTTTAGAATCAAAGGGACCAACT‑‑‑‑‑‑‑‑‑‑‑‑‑‑‑‑‑‑GATCCTATCACGAATGAATCGATTCAG‑‑‑‑‑‑‑‑‑‑‑‑AAGCTTGTAAAAGAAGTAGATGAGGAGATCACCAAAGCAATCATTTCAATGGGGCTAGCAGAGAAAGTTCAATCAGTAAGAATGGAGTTATCTAAGAACTCA‑‑‑‑‑‑‑‑‑‑‑‑‑‑‑AAC‑‑‑CAACCTCTTAGTACTAACATGGAGGAAAAAGTGGACAGAATCATGGAAGAGATAAATATGAAGATGGCGCAGCCGGGAGCATATCTAGGACTAAAACAGAAGCTT‑‑‑‑‑‑‑‑‑AAGAAGCTAGACACTATAAATAGTTTCATAGAGATGAAAGTA‑‑‑‑‑‑AAACAAGAGAAACTA‑‑‑AGAAACGAGCTCAACGAGAAACTCTCGGCC‑‑‑‑‑‑‑‑‑‑‑‑‑‑‑GATACCAAGGCGAAGATTGCAAGCCTGATGGATGCTCAGGAAAGG‑‑‑‑‑‑‑‑‑‑‑‑‑‑‑‑‑‑‑‑‑ATGCCGGATCATGAATTGGTAGAGAAAGCAATGGAAGTTCAGAGAGAG‑‑‑‑‑‑‑‑‑‑‑‑TTGGAAGAGGTTTTGAAGTCAGCTAACTTGGAAATTGTTGGAGTGATGAAGAAGAATGTTGAAACT‑‑‑‑‑‑‑‑‑‑‑‑‑‑‑‑‑‑CCACCAGCAGAT‑‑‑‑‑‑‑‑‑ATAAAACAGAAGATTGTAGAGTTGAACAATGAG‑‑‑ATCATTGGTGAAATTGATAGGGTGGTGAATGCAGAAGAAGGCCTTAAGGATCAAATTAAGGAACTGGAGAATATGATAGCACAAGGCTTGGATTCTAAGGAT‑‑‑‑‑‑‑‑‑‑‑‑‑‑‑‑‑‑‑‑‑GTTGAGAAGATGGAAGCCGGCATAAAAGAGCGAATC‑‑‑‑‑‑‑‑‑‑‑‑CTCGCTGCTTTGGATGCTGCTGGAGTGAAGGAAAAGATTGAGAGAATGAAGGAGGAGGTAGAATCTTTGTCCATGGCAGGTTTTGAGGACAAGATTGGTGAG‑‑‑‑‑‑‑‑‑‑‑‑‑‑‑‑‑‑‑‑‑GAAAATGGTAGATGC‑‑‑‑‑‑‑‑‑‑‑‑‑‑‑‑‑‑‑‑‑‑‑‑‑‑‑‑‑‑‑‑‑‑‑‑‑‑‑‑‑‑‑‑‑‑‑‑‑‑‑‑‑‑‑‑‑‑‑‑‑‑‑

>Cca_accA1_C.cajan_18479

‑‑‑‑‑‑‑‑‑‑‑‑‑‑‑‑‑‑‑‑‑‑‑‑‑‑‑‑‑‑ATGTTG‑‑‑‑‑‑‑‑‑‑‑‑‑‑‑‑‑‑‑‑‑‑‑‑‑‑‑‑‑‑‑‑‑‑‑‑‑‑‑‑‑‑‑‑‑‑‑‑‑‑‑‑‑‑‑‑‑‑‑‑‑‑‑‑‑‑‑‑‑‑‑‑‑‑‑‑‑‑‑‑‑‑‑‑‑‑‑‑‑‑‑‑‑‑‑‑‑‑‑‑‑‑‑‑‑‑‑‑‑‑‑‑‑‑‑‑‑CCACGT‑‑‑CAGAGTGCT‑‑‑‑‑‑CCAATAATTATAAAGGGAAAGAAGCATGATTATCCATGGCCTGATAAAATGGATCCAAATATCAGTAGTGGA‑‑‑‑‑‑TATTTAACTTACCTGTCTCACTTTAAGCCTCTGGCAGAGAAACCAAAA‑‑‑CCTGTGACACTAGATTTTGAGAAGCCACTGGTTGATCTAGAGAAAAAGATTATAGAGGTACGTAGAATGGCAGATGACACAGGTTTAGACTTTAGTAATCAAATTGGAGCTCTGGAGAGCAAGTATCAACAGGCTCTGAAAGATCTATACAAGCATTTAACACCTATTCAACGGTTGATGATTGCTCGACATCCCAATAGGCCAACAGTTCTTGATCATATACTTAATATCACAGACAAGTGGCTTGAGCTTCATGGAGACCGTGCTGGCTATGATGATCCAGCAATTGTGACTGGTATAGGGAGTATAGATGGAAAGAGTTACATTTTTATTGGCCATCAAAAAGGTAGGAACACAAAGGAAAACATTGCTCGCAACTTTGCAATGCCAACTCCTCATGGCTACCGCAAAGCATTGCGCATGATGAAATATGCTGATCACCATCAATTCCCCATCATTACATTTGTTGACACTCCTGGGGCCTTTGCTGATCTAAAGTCTGAGGAACTTGGTCAAGGTGAGGCGATAGCCAAAAATTTAAGGACTATGTTTGGTCTCAAGGTACCAATAATAACAGTGGTAACTGGGGAAGGTGGTTCAGGTGGTGCACTTGCCATTGCCTGTGCAAATAAATTGTTTATGCTCGAGAACTCTGCCTTTTATGTTGCAAGTCCTGAAGCTTGTGCTGCAATATTGTGGAAATCATCCAAATCCTCTCCTAAGGCAGCTGAAAAGCTAAAAATAACAGCACAAGAACATTACAGACTCGGAATAGCTGATGGTGTCATTCCTGAGCCACTAGGTGGGGCACATGCTGATCCTACATGGACTTCTCAACAAATCAAGCTTGCAATCACTCAGGCCATGGAGGAGTTGACAAAAATGGATGCAGAGGAGTTGCTACACCATAGACATCTCAAGTTTCGGTCAACTGGAGGCTACCAAGAAGGCATACCTGTGGAACCAGAGAGGAAACGCAACATGAAGCCATCTGAGGTCAACTCAACA‑‑‑AAGATCAGTGAT‑‑‑‑‑‑‑‑‑‑‑‑‑‑‑ATTGAGTCAGAGCTCCAAAGTCTCGAGAAGAAAATTTTAGAAGCAAAAGGACCGGTT‑‑‑‑‑‑‑‑‑‑‑‑GAACTTGATTCACTCACAAAGGAAGCACTTCAA‑‑‑‑‑‑‑‑‑‑‑‑AAGCTGGTGAAAGAAGCTGACCAAGAGATAACTGAAGCACTCATTTCAATGGATTTGGCCGAAAAAGTTCAATCTGCGAAGATGGAACTATCTAAAGTCTCCTCA‑‑‑‑‑‑‑‑‑‑‑‑AAC‑‑‑CAACCCCTCAATAGCAACTTAAAGGAGAAAGTGGACAAAATAGTGAAAGAAATGAAGACCAAGATGTCTCAGCCAGCGGCCTATCTTGGATTGAAACAGAAGCTT‑‑‑‑‑‑‑‑‑CAGAAGCTGGACAATGCCAACAGGCTCATTGAGATGAAGTTG‑‑‑‑‑‑AAACAAGAAGAATTG‑‑‑AAACAAGAGCTGAATCAGAAACTTGGTGAT‑‑‑‑‑‑‑‑‑‑‑‑‑‑‑GACACCAAAGCAAAGATGGAAAACTTGAAGGTTGCATTGGCAAAG‑‑‑‑‑‑GTACCAAAAGGGGAGTCACCAAACAAGGAATTGGTGGAGAAAGCAATGGAAGTCATGAAGGAG‑‑‑‑‑‑‑‑‑‑‑‑TTGGAAGAGGTGTTGAAATCAGCCAACTTGGAGATTGTTGGGGTAGTCGAGAGAAGTGTGGTGAGT‑‑‑‑‑‑‑‑‑‑‑‑‑‑‑‑‑‑CCGCCACCAGAT‑‑‑‑‑‑‑‑‑GTTAAAGAGAAGATAACAGAGTTGAGGAATGAG‑‑‑ATCATGAGTGAAATTGATAGAGTGGTAAAT‑‑‑GAAGAAGGTCTTAAGGGCCAAATCAAGGAGTTGGAGAATGAGATAGCTCGAGTTTCAAATTCTGAGGAT‑‑‑‑‑‑‑‑‑‑‑‑‑‑‑‑‑‑‑‑‑GTACAAAAGATGGAGGAAGGTATTAAGGAGAGGATT‑‑‑‑‑‑‑‑‑‑‑‑CTTGCTGCATTGGATGTCAAAGCACTGAAGGAAAAGATTGAGAGACTGAGAGAAGAGTTGGGATCCTCATCCAAGGATGTTTATGAAAACAAGATTGGTGTTGGAATCCTCATCCAAGAGTTGGAGTCCTTGAGAAAG‑‑‑‑‑‑‑‑‑‑‑‑‑‑‑‑‑‑‑‑‑‑‑‑‑‑‑‑‑‑‑‑‑‑‑‑‑‑‑‑‑‑‑‑‑‑‑‑‑‑‑‑‑‑‑‑‑‑‑‑‑‑‑

>Cof_accA2_RKLL‑2019542

‑‑‑‑‑‑‑‑‑‑‑‑‑‑‑‑‑‑‑‑‑‑‑‑‑‑‑‑‑‑ATGATGCTGAGTCCT‑‑‑‑‑‑‑‑‑TCTGTGGGAATAGACTGCCTTACCAATTACTTTCTTGGAAGTTCATCCAAAAGAGCACTGAATTTGGGTGGTCCGAGG‑‑‑AATTTTCCAGAAGTG‑‑‑AAGAGGTTTCATGTGATT‑‑‑‑‑‑GCAAAGATTAGGAAAGGAAAGAAGCATGATTATCCGTGGCCTGAAAATATAGATCCAAATATCAGCAGTGGG‑‑‑‑‑‑TTTTTGACTTATTTATCTCACTTCAAGCCTCTGCCTGAGAAACCAAAG‑‑‑CCTGTGACACTTGATTTTGAGAAGCCACTGGTTGACTTAGAGAAAAAGATCATTGAGGTACGCCAGATGGCAGACGACACTGGCTTGGATTTCAGTGATCAAATTGGTGCTCTGGAGGGAAAATATCAACAAGCTTTGAAAGATTTATACACCCATTTAACACCAATTCAACGGCTTACCATTGCTCGGCATCCCAATAGGCCAACAGTTCTTGATCATATTCTGAACATAACAGACAAGTGGGTGGAACTTCATGGAGATCGGGCAGGTTATGATGATCCGGCGATTGTGACCGGTATAGGGAGTATGGATGGAAAAAGCTACATGTTCATAGGCCATCAGAAAGGTAGGAACACAAAGGAAAACATCGCCCGCAACTTTGCAATGCCAACTCCTCATGGCTATCGGAAAGCTTTGCGTATGATGAAATATGCTGATCATCATAAATTCCCCATCATTACATTCGTTGACACCCCTGGGGCCTTTGCTGATCTTAAGTCCGAGGAACTGGGTCAAGGCGAAGCAATAGCCCATAATTTGAGGACTATGTTTGGCCTCAAGGTGCCCATAGTGACAGTGGTAACTGGTGAAGGCGGCTCAGGTGGAGCTCTTGCCATTGCATGTGCAAATAAGCTGCTCATGCTGGAGAATTCTGCCTTCTATGTTGCAAGCCCTGAAGCCTGTGCTGCAATATTGTGGAAGTCTTCCCAAGCTGCTCCAAAGGCAGCCGAGAAGCTAAGGATAACAGCTCAAGAACATTACAGACTAGGAATTGCTGATAGTATTATTCCTGAGCCTCTTGGGGGTGCACATGCTGATCCCAGATTGACTTCACAGCAAATTAAGCTTGCAATCACTCAGGCCATGGAGGAGTTGATAAAAATGGATGCAGAAGAGCTACTACACCATCGGAGACTCAAATTCCGGTCAATTGGTCGTTTTCAAGAAGGAAAACCTGTGGAAGACGACAGAAAGCGCAACATGAAGCCAGCTGATGTCAATACATCT‑‑‑AAGATTACTGAT‑‑‑‑‑‑‑‑‑‑‑‑‑‑‑ATCGAGTCCGAGCTTCAAAGTCTCAAGAAGAAAGTCTTGGAAGCAAAAGGACCATCC‑‑‑‑‑‑‑‑‑‑‑‑‑‑‑‑‑‑GATCCCATTACAACGGAAACAATTCAA‑‑‑‑‑‑‑‑‑‑‑‑AAGATCAGGCAAGAA‑‑‑‑‑‑‑‑‑‑‑‑‑‑‑‑‑‑‑‑‑‑‑‑‑‑‑‑‑‑‑‑‑‑‑‑‑‑‑‑‑‑‑‑‑‑‑‑‑‑‑‑‑‑‑‑‑TCTGTGAAGCTGGAACTATCTAAATCATCCTCAAATCGCACGCCAAAC‑‑‑CAGCCCCTTAGCCGGAACTTAAAGGAAAAAGTGGACAAAATAATGCAAGAATTCAAGAGCAGAATGTCACTGCCTGGCGCCTTTCCTGGATTGAAGCAGAAGCTT‑‑‑‑‑‑‑‑‑CACAAGCTAGACACTGTCAACAGGTTCATTGAGATGAAAATG‑‑‑‑‑‑AGACAAGAAAAATTA‑‑‑AAAGCAGATATCAATCAGAAGCTTTCAGAG‑‑‑‑‑‑‑‑‑‑‑‑‑‑‑GACATTAAAGCAAAGATAGCATATCTGAAGGATGCTCAAGAAAAA‑‑‑‑‑‑TTATCAAATGGGAAGTTACCAGATAAGGAATTGATAGAGGAAGCAAAGAAAGCCAAGAAAGAG‑‑‑‑‑‑‑‑‑‑‑‑CTGGAAGAGGTCTTGAGATCAGCCAAATTGGAGGTTGTTGGAGTAATGAAGAGAAAAATGATCAAT‑‑‑‑‑‑‑‑‑‑‑‑‑‑‑‑‑‑CCGCCACAAGAT‑‑‑‑‑‑‑‑‑ATAAAGCAGAAAATATCAGAGTTGAACAATGAA‑‑‑ATCATAGAAGAGATTGATAGAGTGGTGAAA‑‑‑GCAGAAGGGCTACAAGAAAAAATTAAAGAGTTGGAAGATCAGATGGCTAAAGGCTTAAGTTCTGAGGAT‑‑‑‑‑‑‑‑‑‑‑‑‑‑‑‑‑‑‑‑‑GCAGAAAAAAGGAAAGCGGAAATCAAGGATCGCATT‑‑‑‑‑‑‑‑‑‑‑‑CTTGCTGCTTTAGACGCTGCGGCCTTGAAGGAGAAGGTTGATAGTTTGAGATTAGAGTTAGAATCCTTGTATGGAGCAGATGCTGAAAGCAAGGTTGGTGCT‑‑‑‑‑‑‑‑‑‑‑‑‑‑‑‑‑‑‑‑‑GAAAATGGCAGGTGG‑‑‑‑‑‑‑‑‑‑‑‑‑‑‑‑‑‑‑‑‑‑‑‑‑‑‑‑‑‑‑‑‑‑‑‑‑‑‑‑‑‑‑‑‑‑‑‑‑‑‑‑‑‑‑‑‑‑‑‑‑‑‑

>Lja_accA2_Lj1g3v2628750

‑‑‑‑‑‑‑‑‑‑‑‑‑‑‑‑‑‑‑‑‑‑‑‑‑‑‑‑‑‑ATGTTTGTTTGGCCA‑‑‑‑‑‑‑‑‑‑‑‑‑‑‑‑‑‑‑‑‑‑‑‑‑‑‑‑‑‑‑‑‑‑‑‑‑‑‑‑‑‑‑‑‑‑‑‑‑‑‑‑‑‑‑‑‑‑‑‑‑‑‑‑‑‑AAGTATTTAAGAAGTTCAAGA‑‑‑‑‑‑AATACAAAAGAG‑‑‑CAAAGGTTTCATGTCGCT‑‑‑‑‑‑GCAAAGATTAGA‑‑‑‑‑‑AAGAAGCACGATTATCCATGGCCTGATAACATTGATCCAAATATCACTAGCGGA‑‑‑‑‑‑TATCTGAGTTACTTGTCTCACTTTAAGCCTCTGGCTGAGAAACCAAAA‑‑‑GTTGTAACACTTGACTTTGAGAAGCCACTAGTTGATCTGGAAAAAAAGATTATAGAGATACGCAGAATGGCAGATGACACAGGCTTAGATTTCAGTAATCAAATTGGTTCTCTGGAGAGCAAATATCAACAGGCTCTGAAAGATCTATACTCACACTTAACCCCAATTCAACGTCTGATGATTGCGCGACATCCAAACAGGCCAACAGTTCTTGATCATATACTTAATATCACAGACAAGTGGATAGAGCTACATGGAGATCGAGCTGGTTATGATGATCCAGCAATTGTGACAGGTATAGGGAGTATGGATGGAAAAAGTTACATGTTTATTGGTCATCAGAAAGGTAGAAACACAAAGGAAAACATTGCTCGCAACTTTGCAATGCCAACTCCTCATGGTTGGATACTTGGATTATTA‑‑‑‑‑‑‑‑‑‑‑‑‑‑‑‑‑‑‑‑‑‑‑‑‑‑‑TATTGGTTAATCAATCTGATT‑‑‑‑‑‑‑‑‑‑‑‑‑‑‑‑‑‑‑‑‑‑‑‑‑‑‑‑‑‑‑‑‑‑‑‑‑‑‑‑‑‑CTGCAGGGTGAAGCAATAGCCCATAATTTGAGGACCATGTTTGGTCTCAAGGTACCTATTGTAACAGTTGTAACTGGGGAAGGTGGTTCAGGTGGTGCACTTGCCATTGCATGTCCTAATAAGATGTTTATGCTGGAGAATTCTGCCTTTTATGTTGCAAGCCCTGATGCTTGTGCTGCAATATTGTGGAAATCTTCCAAAGCTGCTCCAAAGGCAGCTGAGAAGCTAAGAATAACAGCACAAGAACATTACAGACTTGGAATTGCTGATGGAATTATTCCTGAGCCAGTAGGTGGTGCACATGCTGATCCTACATGGACTTCTCAGCAAATTAAGCTTGCAATCACGCAGGCCATGGAGGAGCTGACAAAAATGGATGAAGAAGAGTTGCTACATCACAGACACCTCAAGTTCAGGTCAATTGGAGGCTTCCAAGAAGGCAAACCAGTGGAACCGGAAAGGAAACGTAGAATGAAGCCATCTGAAGTCAACTCATCA‑‑‑AACATCACTGAT‑‑‑‑‑‑‑‑‑‑‑‑‑‑‑GTTGAGTCAGAGCTCCAAGGCCTGAAGAAGATAATTTTAGAAGCAAAGGGACCATCT‑‑‑‑‑‑‑‑‑‑‑‑‑‑‑‑‑‑GATCCCATCTCAAAGGAACCAATTCAA‑‑‑‑‑‑‑‑‑‑‑‑AAGCTTGTAAATGAAGTAGATCAAGAGATCACCAAAGCACTCATTTCAATGGGGTTGACAGAGAAAGTTCAGTCTGTGATGAAGGAATTATCATCTAAAGCCTCAAATGACTCCCCAAATCAACAACCCCTTGATGGAAACTTAAAGGAGAAGGTGGACAAAATAATGCAGGAAATAAAGAGCAAGATGTCACAGCCAGGTGCCTATCTAGGATTGAAACAGAAGCTT‑‑‑‑‑‑‑‑‑CAGAAGCTTGACACTGTCAACAGGCTCATGGAGGTGAAATCC‑‑‑‑‑‑CAACAAGAAAAACTA‑‑‑AGACAAGAGCTCAATCAGAAACTTTCAGAT‑‑‑‑‑‑‑‑‑‑‑‑‑‑‑GATACAAAAGCAAAGATAGCAACCCTAATGCAAGCTCAGGAACAG‑‑‑‑‑‑‑‑‑‑‑‑‑‑‑‑‑‑‑‑‑GGAAGGACCCAGGAATTGGAAGAGAAAGCAGTGGAAGTCTACAAAGAG‑‑‑‑‑‑‑‑‑‑‑‑CTAGAAGGGGTTTTCCAATCAGCTAACTTGGAGGTTGTTGGTGTCAGGAATAGAAGTGTGGATGGT‑‑‑‑‑‑‑‑‑‑‑‑‑‑‑CCACTTCCACCAGAG‑‑‑‑‑‑‑‑‑ATTGAACAGAAAATAGCAGACTTGAACAATGAG‑‑‑ATCATTAGTGAAATTGATAGAGTGGTGAAT‑‑‑GCAGAAGGTCTTAAGGGCCAAATCAAGAAGTTGGAGAGTGAGATAGCTCAAGGA‑‑‑‑‑‑TCAAAGGAT‑‑‑‑‑‑‑‑‑‑‑‑‑‑‑‑‑‑‑‑‑GTGGAAAAGATGGAAGCTGGCATTAAAGAGAGGATT‑‑‑‑‑‑‑‑‑‑‑‑CTTGCTGCATTGGATGTTACAGCACTGAATGAGAAAATTGAGAGACTGAGGGAGGAGGTGAAGTCCTCATCCAAGGGAGTTTCTGGAAACAAGATTGGCATG‑‑‑‑‑‑‑‑‑‑‑‑‑‑‑‑‑‑‑‑‑GAAAATGGTAGATGG‑‑‑‑‑‑‑‑‑‑‑‑‑‑‑‑‑‑‑‑‑‑‑‑‑‑‑‑‑‑‑‑‑‑‑‑‑‑‑‑‑‑‑‑‑‑‑‑‑‑‑‑‑‑‑‑‑‑‑‑‑‑‑

>Bto_accA2_JETM‑2010170

‑‑‑‑‑‑‑‑‑‑‑‑‑‑‑‑‑‑‑‑‑‑‑‑‑‑‑‑‑‑ATGTATGTGGAAGAG‑‑‑‑‑‑‑‑‑‑‑‑‑‑‑‑‑‑GACGCGAAGATCGTTCAAACGAAACGATACATCAACTTGTTGGAAAAGTTAACCTTGCTAATTGTTTTC‑‑‑TTGGAAGTTCATTCA‑‑‑AAAGGATTTCAAGTAGTT‑‑‑‑‑‑GCAAAGATCAAGAAGGTAAAGAAGCATGATCGTCCGTGGCTTGATAAAATAGACCCAAATAGCAGCACTGAA‑‑‑‑‑‑ATTTTGACTTACCTATCTCACTTCAAGCCACTGGCTGAGAAACCAAAA‑‑‑CCTGTGACACTTGATTTTGAGAAGCCACTGGTTGATCTAGAGAAAAAGATTATTGAGATACGTAGAATGGCAGATGATACTGGCTTAGATTTCACCGATCAAATTAATGCTCTGGAGAGAAAATATCAACAGATTTTGAAATATTTGTACAAACATTTAACACCGATTCAAAGGCTGACTGTTGCACGACATCCCAATAGGCCAACAACTCTTGATCACATACTCAATATTACAGACAAGTGGGTGGAGCTTCATGGAGATCGAGCAGGCTACAATGATCCAGCCATTGTGACTGGTATAGGCAGTATGGATGGGAAAACTTATATGTTCATAGGCCATCAGAAAGGAAGAAACACAAAGGAAAACATTGCTCGCAACTTTGCAATGCCAACTCCTCATGGATACCGTAAAGCCTTGCGCATGATGAAATATGCTGATCATCATAACTTCCCCATCATTACTTTTGTTGACACTCCTGGGGCATTTGCTGATCTTAAATCTGAGGAACTTGGTCAAGGTGAAGCCATAGCCCATAATTTGAGGACTATGTTTGGCCTCAAGGTGCCCATAGTAACAGTTGTAACTGGTGAAGGTGGTTCAGGTGGAGCACTTGCCATTGCCTGTGCCAATAAGCTGTTTATGCTGGAGAATTCTGCCTTCTACGTTGCAAGTCCAGAAGCTTGTGCTGCAATATTGTGGAAGTCTTCTAAAGCCGCTCCCAAGGCAGCTAAAAAGTTAAGAATAACAGCTCAAGAGCATTACAGACTCGGAATTGCTGATGGTATTATTCCTGAGCCGCTTGGTGGGGCACATGCTAATCCTGCATGGACTTCACAGCAAATTAAGCTCGTGATCACTCAGGCCATGGAGGAGTTGAGCAAAATGAATTCAGAAGAGCTTCTACACCATCGAAAGCTCAAGTTCCGGTCCATTGGAGGTTTCCAAGAAGGCATGCCAATAGATCCGAAGAAAATGCGCAACATGAAGCCTTCTGATGTTAATGCATCT‑‑‑AAAATAAATGAC‑‑‑‑‑‑‑‑‑‑‑‑‑‑‑CTTGAAGCAGAGCTGGAAAGTCTCAAGAAGAAAATTTTTGAAGCAAAAGCACCATCT‑‑‑‑‑‑‑‑‑‑‑‑‑‑‑‑‑‑GATCCTATTACTGAGGAAACAATTAAA‑‑‑‑‑‑‑‑‑‑‑‑AGGGTCAAGCAAGAAGTAGACAAAGAGATTACCAAAGCCATCATTTCAATGGGTTTAAAAGAGAAGGTTCAGTCTGTGAAGCTGGAGTTATCTAAAGCA‑‑‑CGAAATCGCTCTAAAAAC‑‑‑CAACCTCTTGATCATGCCTTGAAGGACAAGGTAGACAGCATAATGCAAGAATTCAAGAGTAAACTGTCACGACCAGGCGCCTATCTTGGATTGAAACACAAGCTT‑‑‑‑‑‑‑‑‑CAAAAGCTGGAGATGGTCAACAAGCTCATTGATATGAAAGTC‑‑‑‑‑‑AAAGGAGAAAATTTA‑‑‑AAGGCAGAGATAAATCAGAAAATCTCAGAT‑‑‑‑‑‑‑‑‑‑‑‑‑‑‑GATATTAAAGCAAAGATAACAAATCTGAAGAAATCCCAGAAATTA‑‑‑‑‑‑TTTTCTAACGGGACCTCGCCAAATAAAGAACTAACAGAAGAAGCAGAGAAAGCAATAAAGGAG‑‑‑‑‑‑‑‑‑‑‑‑CTTGTAGAGGCCTTGAGATCAGCCAACTTGGAGGTTGTTGGAGCAGTGAATAAAAAGGTGGTAAAT‑‑‑‑‑‑‑‑‑‑‑‑‑‑‑‑‑‑CTATCACCAGAT‑‑‑‑‑‑‑‑‑ATGGAACAGAGACTAGGACAAGTGAACAATGTG‑‑‑ATCATGAATGAAATTGATAGAGTGGTCAAT‑‑‑GTACAAGGTATTGGGAGCAAAATTAAGGAGCTGGAGGATGAGATAGCAAAAGGGTCAAATTCAAAGGAT‑‑‑‑‑‑‑‑‑‑‑‑‑‑‑‑‑‑‑‑‑GTGGAAAAAATAGAAGCAACTATAAGGGAACGTATT‑‑‑‑‑‑‑‑‑‑‑‑CAAGCTGCTTTGAATGGCACAGCACTGAAGGAAAAGCTTGATGGTCTAAGAGAGAAGGTGGAATCCTCGTCCAAAGTGGTTAGTGAAAGCAAGCAAAGATATGTAAAT‑‑‑‑‑‑‑‑‑‑‑‑‑‑‑‑‑‑‑‑‑‑‑‑‑‑‑‑‑‑‑‑‑‑‑‑‑‑‑‑‑‑‑‑‑‑‑‑‑‑‑‑‑‑‑‑‑‑‑‑‑‑‑‑‑‑‑‑‑‑‑‑‑‑‑‑‑‑‑‑‑‑‑‑‑‑‑‑‑‑‑‑‑

>Cre_accA_XM_001696893

‑‑‑‑‑‑‑‑‑‑‑‑‑‑‑‑‑‑‑‑‑‑‑‑‑‑‑‑‑‑‑‑‑‑‑‑‑‑‑‑‑‑‑‑‑‑‑‑‑‑‑‑‑‑‑‑‑‑‑‑‑‑‑‑‑‑‑‑‑‑‑‑‑‑‑‑‑‑‑‑‑‑‑‑‑‑‑‑‑‑‑‑‑ATGCAGGTCCTCAAGAGCAAGACTCTGGTTAGCGATGCTGCTGCGGCACCGCGCGCTGCTCAGCGTGCCACTGTGGCC‑‑‑‑‑‑CGACCGAGCGTTAAGGTGCAAGCGGCACCCCGACTGCCCCCGGACACGCCTAAGGAGCGCGAGGGAGGCCGCGAGTGGCTGGGCACTATCCTGTCTCGCTTTGGGCCGGTGAAGGACAAGGCGCAGAACACCACGACGCTTGAGTTTGAGAAGCCTCTGCTGGAGCTGGATAAGCGCATCAAGGAGGTGCGCAAGGTCGCCGAGGAGAACGGCGTGGATGTGAGCGCCTCCATTGCTGAGCTGGAGGGCCGCGCCAAGCAGCTGCGGAAGGAGACTTACAGCCGGCTGACGCCCGTCCAGCGTCTGCAAGTCGCTCGCCACCCCAATCGCCCTACCTGCCTGGACATCATCCTGAACATCACCGATAAGTTCGTGGAGCTGCACGGTGACCGCGCGGGTCTCGACGACCCCGCCATCGTGTGCGGTATCGGCAGCATCAATGGCACGCCGTTCATGATGATCGGCCACCAGAAGGGCCGGAACACAAAGGAGAACATTCGCCGCAACTTCGGCATGCCCCAGCCCAACGGCTACCGCAAGGCGCTGCGCTTCATGCGCCACGCCGACAAGTTTGGTCTGCCCATCATCACCTTCGTGGACACGCCCGGAGCCTATGCCGGCAAGACCGCGGAGGAGCTGGGCCAGGGCGAGGCCATTGCCGTGAACCTGCGTGAGATGTTCGGCCTGCGTGTGCCCATCATCTCGGTGGTCATTGGCGAGGGCGGCTCGGGCGGCGCGCTGGCCATTGGCTGCGCCAACCGCAACCTGATCATGGAGAACGCGGTCTACTACGTGGCCTCGCCCGAGGCCTGCGCCGCCATCCTGTGGAAGAGCCGCTCTGCCGCCGGCGAGGCCACTGAGGCCCTGCGCATCACCTCGGCCGAGCTGGTGAAGTTCGGCGTCATGGACCACATCGTGCCGGAGCCGCTGGGCGGCGCGCACAGCGACCCGCTGGCGGCCTTCCCCATGATCAAGGAGAGCATCCTCAACGTCTACTCCGAGTACGCCGTGATGAGCGAGGAGGAGATCAAGCTGGACCGCTACGCCAAGTTCCGCAAGCTGGGCCAGTTCCAAGAGTTTGTGGTCAAGGGCGGTGACTGGCGCACCGCCCTGGCGGAGCGCGCGGCAACCTCCGGCACCACCACCAAGACCGGCGCCTGGGCCGCCACTGAGGCGGAGGCGCGCTACATCGAGCAGCTGGTG‑‑‑‑‑‑‑‑‑‑‑‑‑‑‑‑‑‑‑‑‑‑‑‑‑‑‑‑‑‑‑‑‑‑‑‑‑‑‑‑‑‑‑‑‑GACGCGGACGAGAAGTGGGAG‑‑‑‑‑‑‑‑‑‑‑‑AAGCTCATGGCGGAGGGCGCCGAGTGGCTCAACAAGCCCGTGCAGCCGCCGGGCCTGGGCCGGTCA‑‑‑‑‑‑‑‑‑‑‑‑‑‑‑‑‑‑‑‑‑‑‑‑‑‑‑‑‑‑‑‑‑‑‑‑‑‑‑‑‑‑‑‑‑‑‑‑‑‑‑‑‑‑‑‑‑‑‑‑‑‑‑‑‑‑‑‑‑‑‑‑‑‑‑‑‑‑‑‑‑‑‑‑‑‑‑‑‑‑‑‑‑‑‑‑‑‑‑‑‑‑‑‑‑‑‑‑‑‑‑‑‑‑‑‑‑‑‑‑‑‑‑‑‑‑‑‑‑‑‑‑‑‑‑‑‑‑‑‑‑‑‑‑‑‑‑‑‑‑‑‑‑‑‑‑‑‑‑‑‑‑‑‑‑‑‑‑‑‑‑GGCATCATGGACGTGGCGGTGTCTATGGTGGAGGCGCGGCGG‑‑‑‑‑‑CGGAAGCAGCAGCAG‑‑‑‑‑‑‑‑‑‑‑‑‑‑‑‑‑‑‑‑‑‑‑‑‑‑‑‑‑‑‑‑‑‑‑‑‑‑‑‑‑‑‑‑‑‑‑‑‑‑‑‑‑‑‑‑‑‑‑‑‑‑‑‑‑‑‑‑‑‑‑‑‑‑‑‑‑‑‑‑‑‑‑‑‑‑‑‑‑‑‑‑‑‑‑‑‑‑‑‑‑‑‑‑‑‑‑‑‑‑‑‑‑‑‑‑‑‑‑‑‑‑‑‑‑‑‑‑‑‑‑‑‑‑‑‑‑‑‑‑‑‑‑‑‑‑‑‑‑‑‑‑‑‑‑‑‑‑‑‑‑‑‑‑‑‑‑‑‑‑‑‑‑‑‑‑‑‑‑‑‑‑‑‑‑‑‑‑‑‑‑‑‑‑‑‑‑‑‑‑‑‑‑‑‑‑‑‑‑‑‑‑‑‑‑‑‑‑‑‑‑‑‑‑‑‑‑‑‑‑‑‑‑‑‑‑‑‑‑‑‑‑‑‑‑‑‑‑‑‑‑‑‑‑‑‑‑‑‑‑‑‑‑‑‑‑‑‑‑‑‑‑‑‑‑‑‑‑‑‑‑‑‑‑‑‑‑‑‑‑‑‑‑‑‑‑‑‑‑‑‑‑‑‑‑‑‑‑‑‑‑‑‑‑‑‑‑‑‑‑‑‑‑‑‑‑‑‑‑‑‑‑‑‑‑‑‑‑‑‑‑‑‑‑‑‑‑‑‑‑‑‑‑‑‑‑‑‑‑‑‑‑‑‑‑‑‑‑‑‑‑‑‑‑‑‑‑‑‑‑‑GCGGGCCAGGTGCACAAGTCGGCACCCGCCCCCGCC‑‑‑AGCAGCAACGGCGCCGTCGTCAACGCCGCCGCT‑‑‑‑‑‑‑‑‑‑‑‑‑‑‑‑‑‑‑‑‑‑‑‑‑‑‑‑‑‑‑‑‑‑‑‑‑‑‑‑‑‑‑‑‑‑‑‑‑‑‑‑‑‑‑‑‑‑‑‑‑‑‑‑‑‑‑‑‑‑‑‑‑‑‑‑‑‑‑‑‑‑‑‑‑‑‑‑‑‑‑‑‑‑‑‑‑‑‑‑‑‑‑‑‑‑‑‑‑‑‑‑‑‑‑‑‑‑‑‑‑‑‑‑‑‑‑‑‑‑‑‑‑‑‑‑‑‑‑‑‑‑‑‑‑‑‑‑‑‑‑‑‑‑‑‑‑‑‑‑‑‑‑‑‑‑‑‑‑‑‑‑‑‑‑‑‑‑‑‑‑‑‑‑‑‑‑‑‑‑‑‑‑‑‑‑‑‑‑‑‑‑‑‑‑‑‑‑‑‑‑‑‑‑‑‑‑‑‑‑‑‑‑‑‑‑‑‑‑‑‑‑‑‑

# Table S13. FASTA alignment of *accB1* sequences used for phylogenetic inference

>Adu_accB1_Aradu.JDP66

‑‑‑ATGGCA‑‑‑‑‑‑TCTTCTTTCGCTTCAACTGCTTCTTCCGCATCTTCTTCGCTT‑‑‑CCCACTCCCTCAAAACCCAAACCC‑‑‑AAAATCAATCAT‑‑‑‑‑‑TTCCGCTTCTCTCACTCCAACCTCTCTTTC‑‑‑CGTCTCTCTCCTAAACCCAAC‑‑‑‑‑‑CTTCCTTTCCTCACCAAGGGATCCCCACCA‑‑‑TGTCAAATTGTATGCCCAAGGGTGAAGGCACAATTG‑‑‑GATGAGGTTTCTCTTGATGGTTCCTCAAATGCTGTC‑‑‑GCT‑‑‑‑‑‑‑‑‑CCTACAACAGCCAATTCAGAGGCC‑‑‑‑‑‑‑‑‑‑‑‑‑‑‑‑‑‑GAAGCGACTGCCAAACCTTCGAGTGGAACATCGTCTGGAGTCTTGGCTTCTCAAGAATCTATCTCTCAGTTTATTACTCAAGTTGCAAGCCTTGTCAAACTTGTTGATTCAAGAGACATTGTAGAGTTGCAGTTGAAGCAGCATGACTGTGAAGTAATGATCAGGAAGAGGGAGGCTATGCCTCAACCACAGCCTCCTGCTCAGCCTGCCATGTACTATCCACCC‑‑‑‑‑‑CCATCACTTGCA‑‑‑‑‑‑‑‑‑GCGCCACCTGCTGCTCCAGCATCTTCTCCAGCACCTGCCCCAACTCCTGCTACCCGT‑‑‑GCAGCA‑‑‑TCAGCTTCTCCACCTGCTGCAAAGTCAACTAAATCATCACTTCCG‑‑‑‑‑‑‑‑‑CCTCTTAAATGTCCTATGGCAGGGACATTTTACCGCAGTCCTGGTCCTGGTGAACCTCCATTTGTGAAGGTTGGAGACAAAGTAAAGAAGGGCCAAGTGTTGTGCATCATCGAGGCGATGAAATTGATGAATGAAATAGAAGCTGATCAATCAGGAACTATAGTCGAAATCCTTGCAGAAGATGGCAAGCCTGTTAGTGTTGACATGCCCCTTTTTGTGATTGAACCT

>Aip_accB1_Araip.95C8Z

‑‑‑ATGGCA‑‑‑‑‑‑TCTTCTTTCGCTTCAACTGCTTCTTCCGCATCTTCTTCGCTT‑‑‑CCCACTCCCTCAAAACCCAAACCC‑‑‑AAAATCAATCAT‑‑‑‑‑‑TTCCGCTTCTCTCACTCCAACCTCTCTTTC‑‑‑CGTCTCTCTCCTAAACCCAAT‑‑‑‑‑‑CTTCCTTTCCTCACCAAGGGATCCCCACCA‑‑‑TGTCAAATTGTATGCCCAAGGGTGAAGGCACAATTG‑‑‑AATGAGGTTTCTCTTGATGGTTCCTCAAATGCTGTC‑‑‑GCT‑‑‑‑‑‑‑‑‑CCTACAACAGCCAATTCAGAGGCC‑‑‑‑‑‑‑‑‑‑‑‑‑‑‑‑‑‑GAAGCAACAGCCAAACCTTCAAGTGGAACATCGTCTGGAGTCTTGGCTTCTCAAGAATCTATCTCTCAGTTTATTACTCAAGTTGCAAGCCTTGTCAAACTTGTTGATTCAAGAGACATTGTAGAGTTGCAGTTGAAGCAGCATGACTGTGAAGTAATGATCAGGAAGAGGGAGGCTATGCCTCAACCACAGCCTCCTGCTCAGCCTGCCATGTACTATCCACCC‑‑‑‑‑‑CCGTCACTAGCA‑‑‑‑‑‑‑‑‑GCGCCACCTGTTGCTCCTGCATCTTCTCCAGCACCTGCCCCAACTCCTGCTACCCGT‑‑‑GCAGCA‑‑‑TCAGCTTCTCCACCTGCTGCAAAGTCAACTAAATCATCACTTCCG‑‑‑‑‑‑‑‑‑CCTCTTAAATGTCCTATGGCAGGGACATTTTACCGCAGTCCTGGTCCTGGTGAACCTCCATTTGTGAAGGTTGGAGACAAAGTAAAGAAGGGCCAAGTATTGTGCATCATCGAGGCGATGAAATTGATGAATGAAATAGAAGCTGATCAATCAGGAACTATAGTCGAAATCCTTGCAGAAGATGGCAAGCCTGTTAGTGTTGACATGCCCCTTTTTGTGATTGAACCT

>Ath_accB1_AT5G16390

ATGGCGTCT‑‑‑‑‑‑TCGTCGTTCTCAGTCACATCTCCAGCTGCTGCTGCTTCCGTC‑‑‑TATGCAGTCACTCAAACCTCCTCG‑‑‑CACTTCCCAATC‑‑‑‑‑‑‑‑‑CAAAACCGCTCTCGCAGAGTTTCTTTC‑‑‑CGTCTCTCTGCTAAGCCCAAG‑‑‑‑‑‑CTTCGCTTTCTCTCCAAG‑‑‑‑‑‑‑‑‑CCT‑‑‑AGTCGCAGTAGCTACCCTGTGGTGAAAGCACAATCT‑‑‑AACAAGGTTAGTACTGGTGCATCATCAAATGCTGCC‑‑‑AAAGTTGATGGGCCATCATCAGCTGAAGGAAAGGAG‑‑‑‑‑‑‑‑‑‑‑‑‑‑‑‑‑‑AAAAACTCATTGAAGGAGTCGTCT‑‑‑GCTTCTTCTCCTGAATTAGCTACAGAAGAGTCTATTTCTGAGTTCCTTACCCAAGTAACAACTCTTGTCAAGCTTGTGGATTCAAGAGACATTGTTGAGTTGCAGTTGAAACAACTCGACTGTGAACTAGTCATTCGAAAAAAAGAAGCCTTACCTCAACCTCAAGCTCCTGCATCTTATGTTATGATGCAGCAACCAAATCAACCATCTTATGCCCAGCAAATGGCTCCTCCTGCTGCACCTGCTGCTGCC‑‑‑‑‑‑‑‑‑GCACCAGCCCCTTCTACG‑‑‑‑‑‑CCAGCCTCTCTGCCTCCACCATCCCCACCTACTCCAGCCAAATCGTCACTTCCT‑‑‑‑‑‑‑‑‑ACTGTTAAAAGCCCCATGGCTGGCACATTCTACCGTAGTCCTGCACCTGGTGAACCACCCTTTATTAAGGTTGGAGACAAAGTGCAGAAGGGGCAAGTTCTATGCATTGTTGAAGCCATGAAGTTAATGAATGAAATAGAGTCTGACCATACCGGAACCGTAGTCGATATTGTCGCAGAAGACGGCAAGCCTGTCAGCCTCGACACTCCTCTGTTTGTGGTTCAACCG

>Car_accB1_Ca_06111

‑‑‑‑‑‑‑‑‑‑‑‑‑‑‑‑‑‑‑‑‑‑‑‑‑‑‑‑‑‑‑‑‑‑‑‑‑‑‑‑‑‑‑‑‑‑‑‑‑‑‑‑‑‑‑‑‑‑‑‑‑‑‑‑‑‑‑‑‑‑‑‑‑‑‑‑‑‑‑‑‑‑‑‑‑‑‑‑‑‑‑‑‑‑‑‑‑‑‑‑‑‑‑‑‑‑‑‑‑‑‑‑‑‑‑‑‑‑‑‑‑‑‑‑‑‑‑‑‑‑‑‑‑‑‑‑‑‑‑‑‑‑‑‑‑‑‑‑‑‑‑‑‑‑‑‑‑‑‑‑‑‑‑‑‑‑‑‑‑‑‑‑‑‑‑‑‑‑‑‑AGGGGT‑‑‑‑‑‑‑‑‑‑‑‑‑‑‑‑‑‑‑‑‑‑‑‑‑‑‑‑‑‑CGTGGTAACTCGGAGTTTGGCAAGGAGGTTGCCCTTGATGGTCCCTCCAATGATGTG‑‑‑CCT‑‑‑‑‑‑‑‑‑CCTGTTAAAGCCAAATCAGGCGTG‑‑‑‑‑‑‑‑‑‑‑‑‑‑‑‑‑‑GAATCACCAACCACACCTTCAATTGAACCATCTTCTTCAGTCTCGGTAAACCAAGAATCAATCTCTCAGTTTTTTACTCAAGTTGCAAGTCTCGTCAAGCTTGTTGATTCAAGAGACATTGTAGAGTTGAAGCTGAAGCAGCTAAACTGTGAGGTAACAATCAGGAAAAAAGAGGCTTTGCCTCAACCACAGTATGCTCCTCAACCTGCAGTGATGTATTCACCA‑‑‑‑‑‑CCTCCGCAACAT‑‑‑‑‑‑‑‑‑‑‑‑‑‑‑‑‑‑TTTGCGCCATCATCT‑‑‑‑‑‑‑‑‑‑‑‑ACTCAGGCTCCTCTTGCT‑‑‑‑‑‑CCTTCACCGTCAACTTCTCCCCCTGCTGTCAAGTTGCTTAAATCATCACGTCCA‑‑‑‑‑‑‑‑‑CCTCTTAAAAGCCCCATGGCAGGGACATTCTACCGAAGTCCTGCACCGGGTGAACCTCCATTTGTGAAGGTTGGTGACAAAGTGAAGAAAGGTCAAGTCTTATGCATCGTTGAGGCAATGAAATTGATGAATGAAATAGAAGCTGATCAGTCAGGAACTATAGTTGAAATCGTCGCGGAAGATGCTAAGCCCGTAAGCGTTGACACTCCGCTATTTGTGATCGAACCG

>Cca_accB1_C.cajan_18238

‑‑‑ATGGCT‑‑‑‑‑‑TCCTCCTTCGCA‑‑‑‑‑‑‑‑‑‑‑‑‑‑‑‑‑‑‑‑‑‑‑‑‑‑‑‑‑‑‑‑‑CCTGGTACCACCAAAGCCGCCACT‑‑‑AATCTCTGTCTC‑‑‑‑‑‑ACACAC‑‑‑‑‑‑‑‑‑‑‑‑‑‑‑‑‑‑TCTTCC‑‑‑CGTCTCTCTCCTAAACCCAACAAC‑‑‑CTACGCTTTCACACCCCC‑‑‑AACAAGCCTGGTCATGTGCTGTTATGCCCAAGGGTTAAGGCCCAATTA‑‑‑AAAGAGGTTTCCCTTGATGGTTCCTCCAATGCGCCT‑‑‑CCT‑‑‑‑‑‑‑‑‑CCTAACAAAGAAAAATCAGACAAG‑‑‑‑‑‑‑‑‑‑‑‑‑‑‑‑‑‑GAACCACCCATGAAACCTTCAGCTGAATCATCTTCTTCGGTCCTGGCAACACAAGAGTCTGTCTCTCAGTTTATTAATCAAGTTGCAAGTCTTGTCAAGCTTGTTGATTCAAGAGACATTATGGAGTTGAAGATGCAGCAGCATGATGTAGAAATAACAATCAGGAAAAAGGAGGCTATGCCTCAACTG‑‑‑‑‑‑‑‑‑‑‑‑CAACCTGCAGTGGTGTATTCAACCCCTCCACCAGCGATGCCC‑‑‑‑‑‑‑‑‑CTTCCACCTGTTGAATCGGCTTCTCCTCCAACATCTGCTCTAGCTCGCCCTTCC‑‑‑‑‑‑CCTACA‑‑‑CCAGCTTCTCCCCCTGCTGTCAAGTCAACTAAGTCATCACATCCG‑‑‑‑‑‑‑‑‑CCTCTTAAAAGCCCCATGGCAGGGACATTCTACCGAAGTCCTGCACCTGGTGAACCTCCATTTGTGAAGGTTGGAGACAAAGTAAAGAAAGGGCAAGTGGTATGCATCATTGAGGCAATGAAATTGATGAATGAAATAGAAGCTGATCTGTCAGGAACTATAGTTGAAGTCATTGCGGAAGATGGCAAGTCTGTTAGCGTTGACGCTCCCCTATTTGTGATTGAACCA

>Ccan_accB1_RKFX‑2045617‑Cercis_canadensis

‑‑‑ATGGCT‑‑‑‑‑‑TCCTCTTTTGCTACAACAGCATCTTCGGCTTCCGCCTCACCTTCTGCTATTACCACCAAAGCCACCACCATCAATTTGCCTCTTTCTTATAGCAGATACTCTTTATCTAATGTATCTTTC‑‑‑GGCCTCTCTTCCAAATCCAAG‑‑‑‑‑‑CTCCGATTTTCCACCAAGGGATCACCACCC‑‑‑AGTCAAATGTTATTCCCAGTGGTCAAGGCCCAGTTG‑‑‑AATGAGGTTGCGGTTGATGGATCCTCAAATGCTGCG‑‑‑GCG‑‑‑‑‑‑‑‑‑GCTAGCAAACCCAAATCAGAGGTA‑‑‑‑‑‑GCAGCAGTGGGTGCAAAGGATGGCAAGCCATCAGGTGAACTATCTTCTGGAGGTTTGGCCACTGAAGAATCAATTGCTGAGTTTATTACTCAAGTTGCAAGTCTCGTCAAACTCGTTGATTCAAGAGACATTGTAGAGTTGCAGTTGAAGCAGCTTGATTGCGAAGTAACCATCAGGAAAAAGGAGGCCATACCTCAACCTGCATCTCCTGCTGAATTTGCCATGATTCAGTCTCCC‑‑‑‑‑‑CCTTCACTG‑‑‑‑‑‑‑‑‑‑‑‑‑‑‑CCGCCAGCC‑‑‑‑‑‑‑‑‑‑‑‑‑‑‑‑‑‑‑‑‑‑‑‑ACACCAGCTCCTCCTACTCCTACTCCTACTCCATCTACTTCTCCCCCTGCTGTTAAGTCAGCCAAATCATCGCATCCA‑‑‑‑‑‑‑‑‑CCTCTTAAGTGCCCCATGGCAGGGACATTCTATCGAAGTCCAGCACCTGGTGAACCTCCATTTGTGAAGGTTGGAGACAAAGTAAAAAAGGGACAAGTCTTATGCATCATTGAAGCCATGAAATTGATGAACGAAATAGAGGCTGATCAATCGGGAACTGTAGTTGATATCCTTGCAGAAGATGGCAAACCTGTAAGCGTTGATATGCCCCTTTTTGTGATTGAACCC

>Cre_accB_XM_001700390

‑‑‑‑‑‑‑‑‑‑‑‑‑‑‑‑‑‑ATGTTTGCC‑‑‑‑‑‑‑‑‑‑‑‑‑‑‑‑‑‑‑‑‑‑‑‑‑‑‑‑‑‑‑‑‑‑‑‑‑‑‑‑‑‑‑‑‑‑‑‑‑‑‑‑‑‑‑‑‑‑‑‑‑‑‑‑‑‑‑‑‑‑‑‑‑‑‑‑‑‑‑‑‑‑‑‑‑‑‑‑‑‑‑‑‑‑‑‑‑‑‑‑‑‑‑‑‑‑‑‑‑‑‑‑‑‑‑‑‑‑‑‑‑‑‑‑‑‑‑‑‑‑‑‑‑‑‑‑‑‑CTTCGC‑‑‑‑‑‑GCTCCTGTGGCCCGGCCC‑‑‑‑‑‑‑‑‑‑‑‑‑‑‑‑‑‑‑‑‑‑‑‑TCCGCCGCTCGGAGT‑‑‑GTGCAGTCTGGTGTAGCCAGCCGTGTGCAGCGCGTC‑‑‑GTG‑‑‑‑‑‑‑‑‑CCCCGCGCGGCTAAGACCGAGGAG‑‑‑‑‑‑‑‑‑‑‑‑‑‑‑‑‑‑GTTGAGGCTGCTACCAACGGTGCCGCTGCCTCGAACGGC‑‑‑‑‑‑CATGCTTCGGCCAACATGATGCAGTTC‑‑‑GACGAGCTGTCTGATATCATTCGGCTGGTGCATGATACCGACATTGTCGAGTTCGAGCTGAAGAGCAAGCGTTTCAGCCTGAGCGTGCGCAAGAAGGAGGCTCTGCAGGCTGAGCAGGCGGCTGCCTACCAGGCCATGCTG‑‑‑‑‑‑CCC‑‑‑‑‑‑CAGATGCTGGCG‑‑‑‑‑‑‑‑‑GCTGCCCCCGTGGCCCCCATCGCTGCCCCCGTGGCTGCCGCCCCTGCCCCTGCCCCGGCGCCAGCTGCCGCCCCCGCGGCACCGGCCGCCGCGCCAGCGCCCGCCGCAGCGCCCAAGGGCATTGAGGTTGCCTCTCCCATGGGTGGCACCTTCTACCGCAAGCCTGCCCCCGGTGAGCCGGAGTTCGCCAAGGTTGGCGACAAGGTGAAGAAGGGCCAGACCGTGTGCATCATTGAGGCCATGAAGCTGATGAACGAGATCGAGGCCGAGGTTGGCGGTGAGGTGATCAAGTTCCTGGTGGAGAACGGCCAGCCCGTGACTGTGGGCCAGCCCATCATGATCATCAAGCCA

>Gma_accB1a_Glyma.09G248900

‑‑‑ATGGCA‑‑‑‑‑‑TCCTCGTTG‑‑‑‑‑‑‑‑‑‑‑‑‑‑‑‑‑‑‑‑‑‑‑‑‑‑‑‑‑‑‑‑‑‑‑‑GCACCTGCTACCAAAGCCGCCACT‑‑‑AATTTGCGCCTC‑‑‑‑‑‑ACACAC‑‑‑‑‑‑‑‑‑‑‑‑‑‑‑‑‑‑TCTCTC‑‑‑CGCTTCTCTCCTAAACCCAACAAC‑‑‑CTACGCTTTGCCACCAAG‑‑‑‑‑‑‑‑‑CCTGGTAATACGCTGTTATGCGCAAGGGTTAAGGCCCAATTA‑‑‑GATGAGGTTGCCCTTGATAGTTCCTCAAATGCTACT‑‑‑CCT‑‑‑‑‑‑‑‑‑CCTATCAAAGCCAAATCAAAGGAG‑‑‑‑‑‑‑‑‑‑‑‑‑‑‑‑‑‑GAACCACCCGCAAAACCTTTAGCTGAACCATCTTCTTCAGTCTTGGCAACTCAAGAATCGGTCTCTCAGTTTATTACTCAAGTTGCAAGTCTTGTCAAGCTTGTTGATTCGAGAGACATTGTGGAGTTGAAGCTGAAGCAGCATGATGTTGAAGTAACAATTAGGAAAAAGGAGGCTATGCCTCAACCACCACCTCCTCCTCAACCTGCGGTGGTGTATTCATCCCCTCCACCAGTGTTG‑‑‑‑‑‑‑‑‑‑‑‑CCACCGCCATCCGTACCAGCATCTACTCCAGCACCTACTCTAGCTCGTGCAACC‑‑‑‑‑‑CCTTCA‑‑‑CCAACTTCAGCCCCTGCTGTGAAGTCAGCTAAATCATCACTTCCG‑‑‑‑‑‑‑‑‑CCTCTTAAAAGCCCCATGGCAGGGACATTCTACCGAAGTCCTGCACCCGGTGAACCTCCCTTTGTGAAGGTTGGAGACAAAGTAAAGAAGGGGCAAGTTGTATGCATCATTGAGGCAATGAAATTGATGAATGAAATAGAAGCTGATCAGTCAGGAACTATAGTTGAAATTGTTGCGGAAGATGCCAAATCTGTAAGCGTTGACACTCCCCTATTTGTGATTCAACCA

>Gma_accB1b_Glyma.18G243500

‑‑‑ATGGCA‑‑‑‑‑‑TCCTCGTTG‑‑‑‑‑‑‑‑‑‑‑‑‑‑‑‑‑‑‑‑‑‑‑‑‑‑‑‑‑‑‑‑‑‑‑‑GCACCAGCTACCAAAGCCGCCACT‑‑‑AATTTGCGCCTC‑‑‑‑‑‑ACACAC‑‑‑‑‑‑‑‑‑‑‑‑‑‑‑‑‑‑TCTCTC‑‑‑CGCTTCTCTCCTAAACCCAACAAC‑‑‑CTACGCTTTGCCACCAAG‑‑‑‑‑‑‑‑‑CCTGGTAATACGCTGTTATGCACGAGGGTTAAGGCCCAATTA‑‑‑AATGAGGTTGCCCTTGATAGTTCCTCCAATGCTACTTCTCCT‑‑‑‑‑‑‑‑‑CCTATGAAAGCCAAATCAAAGGAG‑‑‑‑‑‑‑‑‑‑‑‑‑‑‑‑‑‑GAACCACCCGCAAAGCCTTTAGCAGAACCATCTTCTTCAGTGTTGGCAACTCAAGAATCGGTCTCTCAGTTTATTACTCAAGTTGCAAGTCTTGTCAAGCTTGTTGATTCAAGAGACATTGTGGAGTTGAAGCTGAAGCAGCATGACGTTGAAGTAACAATCAGGAAAAAGGAGGCTATGCCTCAGCCACCACCTGCTCCTCAACCTTCTGTGGTGTATTCACCCCCTCCACCAGCGTTG‑‑‑‑‑‑‑‑‑‑‑‑CCACCACCACCTGTACCAGCATCTACTCCAGCACCTACTCTAGCTCGTGCAACC‑‑‑‑‑‑CCTACA‑‑‑CCAACTTCAGCCCCTGCTGTGAAGTCAGCTAAATCATCACTTCCG‑‑‑‑‑‑‑‑‑CCTCTTAAAAGCCCCATGGCAGGGACATTCTACCGAAGTCCTGCACCTGGTGAACCTTCCTTTGTGAAGGTTGGAGACAAAGTAAAGAAGGGGCAAGTTGTATGCATCATTGAGGCAATGAAATTGATGAATGAAATTGAAGCTGATCAGTCAGGAACTATAGTTGAAATCGTTGCGGAAGATGCCAAGTCTGTAAGCGTTGACACTCCCCTATTTGTGATTCAACCA

>Gpo_accB1_VLNB‑2074938‑Gompholobium_polymorphum

‑‑‑ATGGCATCGTCATCATCTTTGGCTTCAACATCACCTTCACCTGCTGCT‑‑‑‑‑‑‑‑‑GCTGCTGCCACCAAAGCCGCCACT‑‑‑AATTTGCGTCTC‑‑‑‑‑‑TCTCATTCCTCAAACTCAAACCTCTCTTTC‑‑‑CCTCTCTCTCCCAAACCCAAC‑‑‑‑‑‑TTCCGTTTATTCACCAAGGTATGCCCTCCTAGTCATATGGTGTTATGCCCAAGGGTTAAGGCCCAGTTA‑‑‑AATGAGGTTGCCTTTGATGGTTCCTCCAATGCTGCG‑‑‑CCT‑‑‑‑‑‑‑‑‑CCTATCAAAGCCAAATCAGACATG‑‑‑‑‑‑‑‑‑‑‑‑‑‑‑‑‑‑GAGTCATCGAACAAGCCTTCAACTGAACCATCTTCTTCAGTCTTGGCATCTCAAGAATCAATCTCTGAGTTTATTACTCAAGTTGCAAGACTTGTTAAGCTTGTTGATTCAAGAGACATTGTAGAGTTGAAGATGAAGCAGCTTGACTGTGAAGTAACAATCAGAAAAAAGGAGGCTATGCCTCAACTACAATCTGCTGCTCAACCTGCCATGGTATATTCACCC‑‑‑‑‑‑CCTTCA‑‑‑‑‑‑‑‑‑‑‑‑‑‑‑CCACCGCCATCTACTACAACACCT‑‑‑‑‑‑‑‑‑‑‑‑ACTCCGTCTCCTCCTACC‑‑‑‑‑‑CCTCCATCATCAACTTCTCCCCCTGCTGTCAAGTCAGCTAAATCATCACATCCA‑‑‑‑‑‑‑‑‑CCTCTCAAATGCCCGATGGCAGGGACATTCTACCGAAGTCCAGCACCCGGTGAACCTGCATTTGTGAAGGTTGGAGACAAAGTAAAGAAGGGGCAAGTCTTATGCATCATTGAGGCAATGAAATTGATGAATGAAATAGAAGCTGATCAGTCAGGAACTATAGTTGAAGTCCTTGCGGAAGATGGGAAGCCTGTAAGCGTTGACACTCCCCTATTTGTGATTGAACCG

>Lal_accB1a_LAGI01_36216

‑‑‑ATGGCA‑‑‑‑‑‑TCAACATCTTCT‑‑‑‑‑‑TTGGCTTCAACACCTTCC‑‑‑‑‑‑‑‑‑CCTATCCCAACCAAACCCACCACT‑‑‑ATTTTCCCTCTC‑‑‑‑‑‑TTTCGTTCCTTTAACTCCAACCTTTCTTTT‑‑‑CGCCTCTCTCCTAATCCCCAGCTC‑‑‑CTGCGTTTTTTGCCTTATGGGTCGGTGTCT‑‑‑CATCTTGTATTTTACCCAATGGTTAAGGCTCAGCTA‑‑‑‑‑‑AAGGCTGGGCTTGACGGTTCCTCAAACGCTGCG‑‑‑GCT‑‑‑‑‑‑‑‑‑CCTAGCATAACCAAATCAGAGTCAAAGGTAGAAACACTGGGTGAAAAGGACCCCAAACCATCAACTGAACTGTCTTCCGGAGTCTTGGCAACTGAAGAATCAATCTCTCAGTTTATTAGTCAAGTTGCAAGTCTTGTAAAGCTTGTTGATTCAAGAGATATTGTAGAGTTGCACTTGAAGCAGCTTGACATTGAAGTAACAATCAGGAAAAAAGAGGCTATACCTCATCTACAGTCTCCACCTCAACCTGCCTTGGTATATTCACCC‑‑‑‑‑‑CCTTCAGTTGTG‑‑‑‑‑‑‑‑‑GCACCACCAGTCGCACCATCTTCTACTTCAATACTTACCCCTGCTCCTCCTGCCCGTGCACCATCG‑‑‑TCAAGTTCTCCTCCTGCCGTCAAGTCAGCAAAATCATCACACCCG‑‑‑‑‑‑‑‑‑CCCCTTAAAAGCCCCATGGCAGGGACATTCTACAAAAGTCCAGCACCTGGTGAACCTGCATTTGTGAAGGTTGGAGACAAAGTAAAGAAGGGGCAAGTCTTATGCATCATCGAGGCAATGAAATTGATGAACGAAATAGAAGCTGATCAGTCAGGAACTATAGTCGATATTCTTGCCGAAGAGGGCAAGCCTGTAAGCGTTGACACTCCCTTATTTGTGATTGAACCG

>Lal_accB1b_LAGI01_41055

‑‑‑ATGGCA‑‑‑‑‑‑TCAACATCTTCT‑‑‑‑‑‑TTGGCTTCAACACCTTCC‑‑‑‑‑‑‑‑‑CCTATCCCAACCAAACCCACCACT‑‑‑ATTTTCCCTCTC‑‑‑‑‑‑TTTCGTTCCTTTAACTCCAACCTTTCTTTT‑‑‑CGCCTCTCTCCTAATCCCAACCTC‑‑‑CCACGATTTTTCACTCAGGGGTCGGTGCCT‑‑‑AATCAAGTATTATACCCAAGGGTTAAGGCTCAGATA‑‑‑AATGAGGCTGGGCTTGACGGTTCCTCAAACGCTGCG‑‑‑GCT‑‑‑‑‑‑‑‑‑CCTAGCATAACCAAATCAGAGTCAAAGGTAGAAACACTGGGTGAAAAGAACCCCAAACCATCAACTGAACTGTCTTCCGGAGTCTTGGCAACTGAAGAATCAATCTCTCAGTTTATTAGTCAAGTTGCAAGTCTTGTAAAGCTTGTTGATTCAAGAGATATTGTAGAGTTGCACTTGAAGCAGCTTGACATTGAAGTAACAATCAGGAAAAAAGAGGCTATACCTCATCTACAGTCTCCACCTCAACCTGCCTTGGTATATTCACCC‑‑‑‑‑‑CCTTCAGTTGTG‑‑‑‑‑‑‑‑‑GCACCACCAGTCGCACCATCTTCTACTTCAATACTTACCCCTGCTCCTCCTGCCCGTGCACCATCG‑‑‑TCAAGTTCTCCTCCTGCCGTCAAGTCAGCAAAATCATCACACCCG‑‑‑‑‑‑‑‑‑CCCCTTAAAAGCCCCATGGCAGGGACATTCTACAAAAGTCCAGCACCTGGTGAACCTGCATTTGTGAAGGTTGGAGACAAAGTAAAGAAGGGGCAAGTCTTATGCATCATCGAGGCAATGAAATTGATGAACGAAATAGAAGCTGATCAGTCAGGAACTATAGTCGATATTCTTGCCGAAGAGGGCAAGCCTGTAAGCGTTGACACTCCCTTATTTGTGATTGAACCG

>Lal_accB1c_LAGI01_41651

‑‑‑ATGGCA‑‑‑‑‑‑TCAACATCTTCT‑‑‑‑‑‑TTGGCTTCAACACCTTCC‑‑‑‑‑‑‑‑‑CCTATCCCAACCAAACCCACCACT‑‑‑ATTTTCCCTCTC‑‑‑‑‑‑TTTCGTTCCTTTAACTCCAACCTTTCTTTT‑‑‑CGCCTCTCTCCTAATCCCAACCTC‑‑‑CCACGATTTTTCACTCAGGGGTCGGTGCCT‑‑‑AATCAAGTATTATACCCAAGGGTTAAGGCTCAGATA‑‑‑AATGAGGCTGGGCTTGACGGTTCCTCAAACGCTGCG‑‑‑GCT‑‑‑‑‑‑‑‑‑CCTAGCATAACCAAATCAGAGTCAAAGGTAGAAACACTGGGTGAAAAGGACCCCAAACCATCAACTGAACTGTCTTCCGGAGTCTTGGCAACTGAAGAATCAATCTCTCAGTTTATTAGTCAAGTTGCAAGTCTTGTAAAGCTTGTTGATTCAAGAGATATTGTAGAGTTGCACTTGAAGCAGCTTGACATTGAAGTAACAATCAGGAAAAAAGAGGCTATACCTCATCTACAGTCTCCACCTCAACCTGCCTTGGTATATTCACCC‑‑‑‑‑‑CCTTCAGTTGTG‑‑‑‑‑‑‑‑‑GCACCACCAGTCGCACCATCTTCTACTTCAATACTTACCCCTGCTCCTCCTGCCCGTGCACCATCG‑‑‑TCAAGTTCTCCTCCTGCCGTCAAGTCAGCAAAATCATCACACCCG‑‑‑‑‑‑‑‑‑CCCCTTAAAAGCCCCATGGCAGGGACATTCTACAAAAGTCCAGCACCTGGTGAACCTGCATTTGTGAAGGTTGGAGACAAAGTAAAGAAGGGGCAAGTCTTATGCATCATCGAGGCAATGAAATTGATGAACGAAATAGAAGCTGATCAGTCAGGAACTATAGTCGATATTCTTGCCGAAGAGGGCAAGCCTGTAAGCGTTGACACTCCCTTATTTGTGATTGAACCG

>Lan_accB1_Lup015815

‑‑‑ATGGCA‑‑‑‑‑‑TCAACATCTTCT‑‑‑‑‑‑TTGGCTTCAACACCTTCC‑‑‑‑‑‑‑‑‑CCTATCCCAACCAAAGCCACCACT‑‑‑AATTTGCGCATC‑‑‑‑‑‑TCTCGTTACTCCAACTCCAACCTTTCTTTT‑‑‑CGCCTCTCTCCTAAACCCAACCTC‑‑‑ATCCGATTTTTCACACAGGGGTCGGTGCCT‑‑‑AATCAAGTATTTTGCCCCAGGGTTAAGGCTCAGTTA‑‑‑AATGAGGCTGGGCTTGATGGTTCCTCGAATGCTGCG‑‑‑GCT‑‑‑‑‑‑‑‑‑CCTAGCATAACCAAATCAGAGTCAAAGGTAGAAACACTGGGTACAAAGGACCCCAAACCATCAACTGAACTATCTTCTGGAGTCTTGGCAACTGAAGAATCAATCTCTCAGTTTATTAGTCAAGTCGCAAGTCTTGTAAAGCTTGTTGATTCAAGAGATATTGTAGAGTTGCACTTGAAGCAGCTTGACATTGAAGTAACAATCAGGAAAAAGGAGGCTATACCTCATCTACAGTCTCCCCCTCAACCTGCCATAGTATATTCGCCC‑‑‑‑‑‑CCTTCAGTTGTG‑‑‑‑‑‑‑‑‑TCACCACCAGTCGCACCATCTTCTACTTCAACACATACCCCTGCTCCTCCTGCCCGTGCACCATCA‑‑‑TCAAGTTCTCCTCCTGCCGTCAAGTCATCAAAATCATCACACCCA‑‑‑‑‑‑‑‑‑CCCCTTAAAAGCCCTATGGCAGGGACATTCTACAAATGTCCAGCACCTGGTGAACCTGCATTTGTGAAGGTTGGAGACAAAGTAAAGAAGGGGCAGGTCTTATGCATCATCGAGGCAATGAAATTGATGAATGAAATAGAAGCTGATCAATCTGGAACTATAGTCGATATTCTTGCCGAAGATGGCAAGCCTGTAAGCGTCGACACTCCCTTATTTGTGATCGAACCG

>Lja_accB1_Lj1g3v2838150

‑‑‑‑‑‑‑‑‑‑‑‑‑‑‑‑‑‑‑‑‑‑‑‑‑‑‑‑‑‑‑‑‑‑‑‑‑‑‑‑‑‑‑‑‑‑‑‑‑‑‑‑‑‑‑‑‑‑‑‑‑‑‑‑‑‑‑‑‑‑‑‑‑‑‑‑‑‑‑‑‑‑‑‑‑‑‑‑‑‑‑‑‑‑‑‑‑‑‑‑‑‑‑‑‑‑‑‑‑‑‑‑‑‑‑‑‑‑‑‑‑‑‑‑‑‑‑‑‑‑‑‑‑‑‑‑‑‑‑‑‑‑‑‑‑‑‑‑‑‑‑‑‑‑‑‑‑‑‑‑‑‑‑‑‑‑‑‑‑‑‑‑‑‑‑‑‑ATGAAAGGATGCCCACCTAGTCATGTGGTATTGTGCCCCAGGGTTAAGGCCCAGTTA‑‑‑AATGAGGTTGCCCATGATGGTTCCTCCAATGCTGCA‑‑‑CCT‑‑‑‑‑‑‑‑‑ACTAACAAATCCGACTCGGACGTG‑‑‑‑‑‑‑‑‑‑‑‑‑‑‑‑‑‑GAACCACCAAACAAACCTTCACCCGAACCATCTTCTTCGGTCTTTGCAACTCAAGAATCAATTTCTGATTTTCTTACTCAAGTTGCAAGTCTTGTCAAGCTTGTTGATTCAAGAGACATTGTAGAGTTGACATTGAAGCAACTAGATTGTGAAGTAACAATCAGGAAAAGGGAGGCTATGCCGCAACTGCAGTCTGCTCTTCAGCCTGCAGTGTTCTATTCACCC‑‑‑‑‑‑CCTTCACCAACG‑‑‑‑‑‑‑‑‑ATGCCACCTGTCGCGACAGCATCTAATCCAGCACCTGCTGTAGCCGCTTCTACC‑‑‑‑‑‑CCTTCG‑‑‑CCATCGACTTCTCTTGCAATCAAGTCAGCTAAATCATCACATCCA‑‑‑‑‑‑‑‑‑CCTCTTAAATCCCCCATGGCAGGAACATTCTACCGAAGTCCTGCACCGGGTGAACCTGCATTTGTGAAGGTTGGAGACAAAGTAAAGAAAGGGCAAGTCTTGTGTATCATCGAGGCAATGAAATTGATGAATGAAATAGAGGCTGACCAGTCAGGAACTGTAGTCGACGTCATTGCAGAGGACGGCAAGTCTGTAAGCGTTGACACTCCCCTATTTGTGATTGAACCG

>Mtr_accB1_Medtr7g080290

‑‑‑ATGGCT‑‑‑‑‑‑TCCTCGTTGTCTTCCACTTCACCTTCTGCTGCTCCT‑‑‑‑‑‑‑‑‑ACTGTCACTGTTGCAGCTGCAGCT‑‑‑TCCACCAAATCT‑‑‑‑‑‑GCACGCTTTTCTCATTCCTCCATCTCTTTCCCTCGTCTCTCTCCTAATTCCAACAACTCTCTCCGCTTTTTCACCAAGGGATGCCCACCTAATCATATGCTATTATGCCCCAGGGTTAAGGCTCAACTA‑‑‑AATGAGGTTGCCCTTGATGGTTCTTCTAATGATGTG‑‑‑CCT‑‑‑‑‑‑‑‑‑CCT‑‑‑AAAGCCAAATCAGATGTT‑‑‑‑‑‑‑‑‑‑‑‑‑‑‑‑‑‑GAACCACCGACCGCACCTTCAGTTGAACCATCTTCTTCAGTCTTGGCAGATCAAGAATCAATCTCTGAACTTTTTGATCAAATTGCAAGTCTTGTCAAGCTTGTTGATTCAAGAGACATTGTAGAATTGAAGCTGAAGCAGCTAAACTGTGAAGTATTAATCAGGAAGAAAGAGGCTATGCCTCAACCAGAATATGCTCCTCAACCTGCAGTGTTGTATTCACAA‑‑‑‑‑‑CCTTCGCAAACG‑‑‑‑‑‑‑‑‑GCGCCACCTGTTGCGCCATCACAT‑‑‑‑‑‑‑‑‑‑‑‑ACTCTGGCTCTTCCTGCT‑‑‑‑‑‑CCTTCAACACCAATTTCTCCCCCTCCTGTCAATCTAACTACATCATCACGTCCG‑‑‑‑‑‑‑‑‑CCTCTTAAAAGCCCCATGGCAGGGACATTCTACCGAAGTCCTGCACCGGGTGAACCTCCGTTTGTAAAGGTGGGAGACGAAGTAAAGAAGGGGCAAGTCGTGTGCATCATTGAAGCAATGAAATTGATGAATGAAATTGAAGCTGATAAGTCAGGGACTATAGTTGAAATAATCGCGGAAGATGCTAAGCCTGTAAGCGTTGATACTCCCCTATTTGTGATTGAACCA

>Pvu_accB1_Phvul.008G053000

ATGGCATCC‑‑‑‑‑‑TCTTCCTTG‑‑‑‑‑‑‑‑‑‑‑‑‑‑‑‑‑‑‑‑‑‑‑‑‑‑‑‑‑‑‑‑‑‑‑‑CCACCTGCGACCAAAGCCACAACC‑‑‑AATTTCCCTCTC‑‑‑‑‑‑ACACAC‑‑‑‑‑‑‑‑‑‑‑‑‑‑‑‑‑‑TCTTTT‑‑‑CGCCTCTCTCCTAAGCCCAACAAC‑‑‑CTACGTTTT‑‑‑‑‑‑CCCCCCACCAAGCCTGGTAATTCGTTGTCCTTCACAAGGTTCAAGGCGCAACTG‑‑‑AATGAGGTTGCCCTTGACAGTTCCTCCAATGCTACT‑‑‑‑‑‑‑‑‑‑‑‑‑‑‑CCTATCAAAGCCAAATCGGATGAG‑‑‑‑‑‑‑‑‑‑‑‑‑‑‑‑‑‑GAACAACCCTCAAAACCTTCAGCTGAACCA‑‑‑TCTTCAGTGTTGGCAACTCAAGAATCGGTCTCTCAGTTTATTACTCAAGTTGCAAGTCTTGTCAAGCTTGTTGACTCAAGGGACATTGTGGAGTTGAAGATGAAGCAGTATGACATTGAACTAACTATCAGGAAAAAGGAGGCCATGCCTGAGCTGCAACCTGCTCCTCAACCTACGGTGGTGTACTCACCCCTTCCGCCAATGATGTCA‑‑‑‑‑‑‑‑‑CCGCCACCTGTCGCACCAACATCTAGTCCAACAACTTCTGTAGCTCATCCAACT‑‑‑‑‑‑GCCACA‑‑‑CCAACTTCTCCCCCTGCTCTCAAGTCAACCAAATCATCAGTTCCA‑‑‑‑‑‑‑‑‑CCTCTTAAATCTCCTATGGCAGGGACATTCTACCGAAGTCCTGGACCTGGTGAACCTGCCTTTGTGGAGGTGGGAGACAAAGTCAAAAAGGGGCAAGTTGTATGCATCATCGAGGCAATGAAATTAATGAATGAAATAGAAGCCGAGGAGTCAGGAACCATAGTTGAAATCCTTGCAGAAGATGCGAAGCCTGTAAGCCTGGACACTCCCCTATTTGTGATTGAACCA

>Vra_accB1_Vradi0363s00170

‑‑‑ATGGCA‑‑‑‑‑‑TCCTCGTTG‑‑‑‑‑‑‑‑‑‑‑‑‑‑‑‑‑‑‑‑‑‑‑‑‑‑‑‑‑‑‑‑‑‑‑‑CCACCTGCGACCAAAGCCGCCACC‑‑‑AATTTCCCTCTC‑‑‑‑‑‑ACACAC‑‑‑‑‑‑‑‑‑‑‑‑‑‑‑‑‑‑TCTTTT‑‑‑CGCCTCTCTCCTAAACCTAACAAC‑‑‑CTTCGTTTT‑‑‑‑‑‑CCTCCCACCAAGCCT‑‑‑CATTCGGTGTCCTTCACAAGGTTTAAGGCGCAATTA‑‑‑AATGAGGTTGCACTTGACAGTTCCTCCAATGCTACT‑‑‑‑‑‑‑‑‑‑‑‑‑‑‑CCTATCAAAGCCAAATCAAATGAG‑‑‑‑‑‑‑‑‑‑‑‑‑‑‑‑‑‑GAACCACCTGCAAAACCTTCGACTGAA‑‑‑TCACCTTCAGTCTTGGCAACTCAAGAATCGGTTTCTCAGTTTATTACTCAAGTTGCAAGTCTTGTCAAGCTTGTTGATTCAAGGGACATTGTGGAGTTGAAGATGAAGCAGTATGACATTGAACTAACAATCAGGAAAAAGGAGGCTATGCCTGAACTACAACCTGCTCCTCAACCTGCGGTGGTGTACTCGTCCCTTCCACCAGCAGCAACA‑‑‑‑‑‑‑‑‑CCGCCACCTGCCGCACCGACATCTACTCCAACAACTAATCTAGCTCGTCCAACT‑‑‑‑‑‑GCTACA‑‑‑CCAACTTCTTCTCCTGCTCCTAAGTCAACCAAATCATCGCTTCCA‑‑‑‑‑‑‑‑‑CCTCTTAAATCACCCATGGCAGGGACATTCTACCGAAGTCCTGCACCTGCTGAACCTCCCTTTGTGAAGGTTGGAGACAAAGTCAAGAAGGGGCAAGTTATATGCATCATCGAGGCAATGAAATTAATGAATGAAATAGAGGCTGATCAGTCAGGAACCATAGCTGAAATCATTGCAGAAGATGCGAAGCCTGTAAGCGTTGACACTCCCCTATTTGTGATTGAACCA

# Table S14. FASTA alignment of *accB2* sequences used for phylogenetic inference

>Aar_accB2a_ZCDJ‑2015355‑Acacia_argyrophylla

‑‑‑ATGGCTTCCTTC‑‑‑ACTGTTCCTTGCCCCAAGTGCCCTTCTCCT‑‑‑‑‑‑‑‑‑‑‑‑‑‑‑‑‑‑‑‑‑GCA‑‑‑‑‑‑‑‑‑CCCCTGAGTTTCCACTCA‑‑‑CCGAATCTTCAACGGTACTCGCAGAAGCTGCCTTTCCACAATGGC‑‑‑TTGAGCTTAACACCTACT‑‑‑CAATCATCCGGATCTTCGCGAGCTGCTCTGGCGTCG‑‑‑TCTGCGTTTCAGTGCCTA‑‑‑AACACAAAGCAACCTTCAGGATGGAAGTTACTTGCAAAACTTAAAGAGGCTACTATTGCTGAAACTCCT‑‑‑TCAAATTCTGCACCTGTGCGTGCTTCTGGATCTGCAGTTGGT‑‑‑ACATCAAAAGAGGAAGATGAGCCTAATGATAAACCCTCGGATCCAAGCGCC‑‑‑CTTGCAGATGCATCTTCAGTCACTGCATTCATGACTCAAGTGTCAGATCTTGTGAAACTTGTGGATTCCAGAGATATTATGGAACTGCAACTCAAGCAGTTAGACTGTGAACTATTGATAAGGAAAAAGGAAGCTTTGCAGCCC‑‑‑‑‑‑CCACAAGCCCCAATTCTATACTCTGCATCA‑‑‑CCA‑‑‑‑‑‑CAAGCGGCCTACCCAACGGGC‑‑‑CCT‑‑‑‑‑‑‑‑‑TCTCTG‑‑‑TCACCACCA‑‑‑‑‑‑‑‑‑‑‑‑GCTCCAGCTGCTACTGTT‑‑‑‑‑‑‑‑‑TCTCCTGCA‑‑‑AGCCCACCATCTTCACAACCAGCGCCTTCTTCACCT‑‑‑‑‑‑GCCCCTGGAAAGGCAAGCCCATCATCATCTCTCCCAGCATTCAAATGTCCAATGGCTGGAACTTTTTATCGGAGTCCTGCG‑‑‑‑‑‑‑‑‑‑‑‑‑‑‑‑‑‑‑‑‑‑‑‑‑‑‑‑‑‑‑‑‑‑‑‑‑‑‑‑‑‑AAAGGCCAAGTCGTTTGCATTATCGAGGCAATGAAACTGATGAATGAAATTGAAGCTACTCAATCCGGAACAATAACTGAGATACTTGTGGAGGATGGTAAACCAGTTAGTGTAGACACGCCA‑‑‑‑‑‑‑‑‑CTTTTTGTAATTGCACCC

>Aar_accB2b_ZCDJ‑2015788‑Acacia_argyrophylla

‑‑‑ATGGCTTCGTTT‑‑‑ACTGTTCCCTGCCCCAAATGTGTTTCTCCT‑‑‑‑‑‑‑‑‑‑‑‑‑‑‑‑‑‑‑‑‑CTC‑‑‑‑‑‑‑‑‑CATTTGGGATTGGGCTCG‑‑‑CAGAATCTGCAGCGACGGAGGCAGATTATGTCTCTTCAAAATGGG‑‑‑TTGAGCTTGAAGCCGTGT‑‑‑CAATCCTCGCCTGCTTCGTTCGCTGATCCCGCGTTG‑‑‑TTTGGGAGTCGGAGCCTT‑‑‑AACCGGAAGCAATTTTCTTCCTTGAGGCTTCAAGCACAGCCAAATGAGGTTACTGCTATCGAAGGTTCT‑‑‑TTTAACTCTGCACCCATACTTAATGCTGGATCCGAAGCTGCA‑‑‑TCACCACAAGATAAAGAT‑‑‑‑‑‑GACGGGAAACTCTCTGGTCCAGGCACT‑‑‑TCTGCGGATGCAGCTTCAGTTTCTGAATTCATGAATCAAGTATCTAGTCTTGTCAAACTTGTGGATTCCAGAGATATTGTGGAGCTGCAACTTAAGCAATCAGACTATGAGCTCTTGATAAGGAAAAAGGAAGCCTTGCAGCCT‑‑‑‑‑‑CCACCAACCACAGCAGCAGCTACAGCACCA‑‑‑CCA‑‑‑‑‑‑CCTTTTCCCTATCCCACATTT‑‑‑CCT‑‑‑‑‑‑‑‑‑TATCCA‑‑‑‑‑‑‑‑‑‑‑‑‑‑‑‑‑‑‑‑‑‑‑‑CCACCAGTTCCAGCTCCT‑‑‑‑‑‑‑‑‑GCTCCTCCA‑‑‑AGTCCTCCAGCTTCAAAAGCCGTGCCTGCCCTACCT‑‑‑‑‑‑TCCCCAGGGAAATCAAGC‑‑‑GCTTCATCCCATCCGCCATTGAAGAGTCCAATGGCTGGCACCTTTTATCGAAGTCCTGGG‑‑‑‑‑‑‑‑‑‑‑‑‑‑‑‑‑‑‑‑‑‑‑‑‑‑‑‑‑‑‑‑‑‑‑‑‑‑‑‑‑‑‑‑‑‑‑‑‑‑‑‑‑‑‑‑‑‑‑‑‑‑‑‑‑‑‑‑‑‑‑‑ATGAAATTGAAG‑‑‑‑‑‑‑‑‑‑‑‑‑‑‑‑‑‑‑‑‑‑‑‑‑‑‑‑‑‑‑‑‑‑‑‑‑‑‑‑‑‑‑‑‑‑‑‑‑‑‑‑‑‑‑‑‑‑‑‑‑‑‑‑‑‑CTGATCGATCAGGAA‑‑‑‑‑‑‑‑‑CTA‑‑‑‑‑‑‑‑‑‑‑‑‑‑‑

>Adu_accB2a_Aradu.0JT6M

‑‑‑ATGGCGTCTTGC‑‑‑ACTATCCCTTGTCCCAAGTGTCTCTCTTTT‑‑‑‑‑‑‑‑‑‑‑‑‑‑‑‑‑‑‑‑‑TCA‑‑‑‑‑‑‑‑‑CATTTGGGATTGAACTCT‑‑‑CAGACCACACAACGGAACATG‑‑‑‑‑‑‑‑‑‑‑‑CACGTGGCTGGG‑‑‑TTGGGATTGAAGAAATCC‑‑‑CAGTCATTTGGATCTTTGGTTTGTGATTCAAACTCA‑‑‑ATTGGGGTTCAGTGCCTT‑‑‑AATACGAAGAAATTTTCTGCCTTGAAGTGTCAAGCACAGCCTAAAGAGGTTGTTACTCTTGAAAATTCT‑‑‑TCAAACTCTGCACCTGCACTTGTCAATGGACCTATACCTGCTTCTTCATCAAAAGAGAAAGACGACGAAAACAGGAAACCTGCTGGGCCAAGCACT‑‑‑TTTGCAGATCCAGCTTCCATGTCTGCATTCATGAATCAAGTTTCAGACCTTGTCAAACTTGTGGATTCTAGAGATATCGTTGAGCTACAACTTAAGCAGGCCGACTGCGAGCTCATGATAAGGAAAAAGGAAGCTTTGGAGCCT‑‑‑‑‑‑CCACCGGCC‑‑‑ATGGTAGCTCCGGTGTCT‑‑‑‑‑‑‑‑‑‑‑‑‑‑‑TTTCCTTATCCTACATAT‑‑‑CCTTCTATGCCTTCACCA‑‑‑CTGCCA‑‑‑‑‑‑‑‑‑‑‑‑‑‑‑CCAGCTGCTGCTGCTGCTCCTGCTCCTGCTCCTGCA‑‑‑AGCGCTGCACCTTCAAAAGCAGCACCTGCCTTACCA‑‑‑‑‑‑CCACCTGCAAAAGCAAGC‑‑‑AGGTCATCTCATCCACCCCTGAAATGTCCAATGGCTGGGACCTTTTATCGTAGTCCAGCACCTGGTGAACCGCCATTTGTCAAGGTGGGAGATAAGGTGCAGAAAGGGCAGGTTATTTGCATTGTTGAGGCTATGAAACTGATGAATGAAATTGAGGCTGATCAATCTGGAACAATAACTGAGATAATTGCCGAGGATGGCAAACCAGTCAGCGTCGACACGCCT‑‑‑‑‑‑‑‑‑CTTTTGGTAATAGTACCA

>Adu_accB2b_Aradu.CG4KR

‑‑‑ATGGCTTCTTTC‑‑‑ACGGTCCCATGTCCCAAGTGCCCTTCTCCT‑‑‑‑‑‑‑‑‑‑‑‑‑‑‑‑‑‑‑‑‑‑‑‑‑‑‑‑‑‑‑‑‑TCTTTGGGATTGAACTCC‑‑‑CAGAAG‑‑‑‑‑‑‑‑‑‑‑‑‑‑‑‑‑‑‑‑‑CTC‑‑‑‑‑‑‑‑‑‑‑‑‑‑‑‑‑‑‑‑‑‑‑‑TTCAAGCCATCT‑‑‑CTCTCGTTTGGATCTTTGGCTGCTGAGTCAGCTTCA‑‑‑TCTGGGATTCGGTGCCTT‑‑‑AATGGGAAGCAATTTTCTGTCCAGAAGCTTCAAGCACAACGCCGTGAGGCTGTTACTACTATT‑‑‑‑‑‑‑‑‑GAAAACTCTGCACCTGTATTGGTCAGTGGACCTAAAGTCGCC‑‑‑GCACCAAATGAGAAAGAAGACCAGAATGGGAAACCCGGT‑‑‑‑‑‑GGCACT‑‑‑ACCACAGATCCATCTTTGGTTTCTGCATTCATGGCTCAAGTGGCAGACCTTGTAAAGCTTGTAGATTCCAGAGATATTGTGGAATTGCAACTTAAACAGTCAGATTGTGAGCTCATGATAAGGAAAAAGGAAGCTTTGGAGCCT‑‑‑‑‑‑CCTTCCCAG‑‑‑GTTATAGCCCCGGCATCA‑‑‑GCA‑‑‑‑‑‑CCAATGCATTATGCTGCATAT‑‑‑CCT‑‑‑‑‑‑‑‑‑TCTCTGCCACCTCCACCACCA‑‑‑‑‑‑‑‑‑CCAGCGGCAGCATCTTCC‑‑‑‑‑‑‑‑‑ACTCCTGCA‑‑‑AGCTCTCCACCTGCAAAAGCAGCACCTGCCTTACCT‑‑‑‑‑‑TCCCCTGGAAAAGCAAGC‑‑‑ACATCAGGTCACCCACCGCTGAAATGTCCAATGGCGGGAACCTTTTATAGGAGTCCAGCTCCTGGTGAACCTCCATTTGTCAAGGTGGGAGATAAAGTACAAAAAGGACAGGTTATATGCATTATTGAGGCCATGAAACTGATGAATGAAATCGAGGCTGATCAAACAGGAACAATAACTGAGATACTAGTTGAAGATGGGAAACCAGTCAGTGTAGACACGGAT‑‑‑‑‑‑‑‑‑GTACCTGAACTCTCTCCA

>Aip_accB2a_Araip.4I8QR

‑‑‑ATGGCTTCTTTC‑‑‑ACGGTCCCATGTCCCAAGTGCCCTTCTCCT‑‑‑‑‑‑‑‑‑‑‑‑‑‑‑‑‑‑‑‑‑‑‑‑‑‑‑‑‑‑‑‑‑TCTTTGGGATTGAACTCC‑‑‑CAGAAG‑‑‑‑‑‑‑‑‑‑‑‑‑‑‑‑‑‑‑‑‑CTC‑‑‑‑‑‑‑‑‑‑‑‑‑‑‑‑‑‑‑‑‑‑‑‑TTGAAGCCATCT‑‑‑CTCTCGTTTGGATCTTTGGCTGCTGAGTCAGCTTCA‑‑‑TCTGGGATTCGGTGCCTT‑‑‑AATGGGAAGCAATTTTCTGTCCAGAAGCTTCAAGCACAACGCCGTGAGGCTGTTACTACTATT‑‑‑‑‑‑‑‑‑GAAAACTCTGCACCTGTATTGGTCAGTGGACCTAAAGTCGCC‑‑‑GCACCAAATGAGAAAGAAGACCAGAATGGGAAACCCGGT‑‑‑‑‑‑GGCACT‑‑‑ACCACAGATCCATCTTTGGTTTCTGCATTCATGGCTCAAGTGGCAGACCTTGTAAAGCTTGTAGATTCCAGAGATATTGTGGAATTGCAACTTAAACAGTCAGATTGTGAGCTCATGATAAGGAAAAAGGAAGCTTTGGAGCCT‑‑‑‑‑‑CCTCCCCAG‑‑‑GTTATAGCCCCGGCATCA‑‑‑GCA‑‑‑‑‑‑CCAATGCATTATGCTGCATAT‑‑‑CCT‑‑‑‑‑‑‑‑‑TCTCCGCCACCTCCACCACCA‑‑‑‑‑‑‑‑‑CCAGTGGCAGCATCTTCC‑‑‑‑‑‑‑‑‑ACTCCTGCA‑‑‑AGCTCTCCACCTGCAAAAGCAGCACCTGCCTTACCT‑‑‑‑‑‑TCCCCTGGAAAAGCAAGC‑‑‑ACATCAGGTCACCCACCGCTGAAATGTCCAATGGCGGGAACCTTTTATAGGAGTCCAGCTCCTGGTGAACCTCCATTTGTCAAGGTGGGAGATAAAGTACAAAAAGGACAGGTTATATGCATTATTGAGGCCATGAAACTGATGAATGAAATCGAGGCTGATCAAACAGGAACAATAACTGAGATACTAGCTGAAGATGGGAAACCAGTCAGTGTAGACACGCCT‑‑‑‑‑‑‑‑‑CTCCTTGTAATAGCTCCG

>Aip_accB2b_Araip.LLR5T

‑‑‑ATGGCGTCTTTC‑‑‑ACTATCCCTTGTCCCAAGTGTCTCTCTTTT‑‑‑‑‑‑‑‑‑‑‑‑‑‑‑‑‑‑‑‑‑TCA‑‑‑‑‑‑‑‑‑CATTTGGGATTGAACTCT‑‑‑CAGACCACCCAACGGAACATG‑‑‑‑‑‑‑‑‑‑‑‑CACGTGGCTGGG‑‑‑TTGGAATTGAAGAAATCC‑‑‑CAGTCATTTGGATCTTTGGTTTGTGATTCAAACTCA‑‑‑ATTGGGGTTCAGTGCCTT‑‑‑AATACGAAGAAATTTTCTGCCTTGAAGTGTCAAGCACAGCCTAAAGAGGTTGTTACTCTTGAAAATTCT‑‑‑TCAAACTCTGCACCTGCACTTGTCAATGGACCTATACCTGCT‑‑‑TCATCAAAAGAGAAAGACGACGAAAACAGGAAACCTTCTGGGCCAAGCACT‑‑‑TTTGCAGATCCAGCTTCCATGTCTGCATTCATGAATCAAGTTTCAGACCTTGTAAAACTTGTGGATTCTAGAGATATCGTTGAGCTACAACTTAAGCAGGCCGACTGCGAGCTCATGATAAGGAAAAAGGAAGCTTTGGAGCCT‑‑‑‑‑‑CCACCGGCC‑‑‑ATGGTAGCTCCGGTGTCT‑‑‑‑‑‑‑‑‑‑‑‑‑‑‑TTTCCTTATCCTACATAT‑‑‑CCTTCTATGCCTTCACCA‑‑‑CCGCCA‑‑‑‑‑‑‑‑‑‑‑‑‑‑‑CCAGCTGCTGCTGCTGTT‑‑‑‑‑‑‑‑‑GCTCCTGCA‑‑‑AGCGCTGCGCCTTCCAAAGCAGCACCTGCCTTACCA‑‑‑‑‑‑CCACCTGCAAAAGCAAGC‑‑‑AGGTCATCTCATCCACCCCTGAAATGTCCAATGGCTGGGACCTTTTATCGTAGTCCAGCACCTGGTGAACCGCCATTTGTCAAGGTGGGAGATAAGGTGCAGAAAGGGCAGGTTGTTTGCATTGTTGAGGCTATGAAACTGATGAATGAAATTGAGGCTGATCAATCTGGAACAATAACTGAGATAATTGCCGAGGATGGCAAACCAGTCAGCGTCGACACGCCT‑‑‑‑‑‑‑‑‑CTTTTGGTAATAGTACCA

>Ath_accB2_AT5G15530

‑‑‑ATGGCGTCATTG‑‑‑TCGGTACCTTGCGTCAAGATCTGTGCTCTG‑‑‑‑‑‑AACAGGCGGGTCGGATCT‑‑‑‑‑‑‑‑‑CTCCCTGGAATCTCTACT‑‑‑CAAAGATGGCAGCCGCAACCCAATGGGATCTCCTTTCCCTCCGAT‑‑‑‑‑‑‑‑‑‑‑‑‑‑‑‑‑‑‑‑‑‑‑‑‑‑‑‑‑‑‑‑‑‑‑‑‑‑‑‑‑‑‑‑‑‑‑‑‑‑‑‑‑‑‑‑‑‑‑‑‑‑‑‑‑‑‑‑‑‑‑‑‑‑‑‑‑‑GTT‑‑‑TCTCAGAATCATTCTGCATTCTGGAGGTTGCGTGCAACAACTAATGAGGTTGTT‑‑‑‑‑‑‑‑‑‑‑‑‑‑‑‑‑‑TCTAACTCCACTCCAATGACTAATGGTGGGTATATG‑‑‑‑‑‑‑‑‑‑‑‑‑‑‑‑‑‑‑‑‑‑‑‑‑‑‑‑‑‑‑‑‑AACGGAAAAGCGAAA‑‑‑‑‑‑ACCAAT‑‑‑GTTCCTGAACCCGCCGAGCTCTCTGAATTTATGGCTAAAGTCTCTGGTCTTCTTAAGCTTGTGGATTCAAAAGACATAGTGGAACTTGAACTAAAGCAGCTCGACTGTGAGATCGTTATTCGAAAGAAGGAAGCTTTACAGCAAGCTGTACCACCAGCTCCAGTTTATCACTCAATGCCT‑‑‑CCT‑‑‑‑‑‑GTAATG‑‑‑‑‑‑GCAGACTTT‑‑‑TCA‑‑‑‑‑‑‑‑‑ATGCCT‑‑‑‑‑‑‑‑‑‑‑‑‑‑‑‑‑‑‑‑‑‑‑‑CCAGCTCAACCAGTGGCT‑‑‑‑‑‑‑‑‑CTTCCTCCT‑‑‑TCTCCTACTCCTACC‑‑‑TCAACGCCTGCAACAGCA‑‑‑‑‑‑AAACCAACATCCGCCCCA‑‑‑TCCTCGTCTCATCCTCCACTCAAGAGTCCCATGGCTGGTACTTTCTATAGATCTCCTGGACCCGGTGAACCCCCTTTTGTAAAGGTTGGAGATAAGGTACAGAAGGGTCAAATTGTTTGTATTATCGAAGCTATGAAACTGATGAACGAGATTGAGGCTGAGAAGTCAGGAACCATCATGGAACTACTGGCTGAAGATGGAAAACCAGTCAGCGTTGACACGCCT‑‑‑‑‑‑‑‑‑CTTTTTGTCATCGCACCT

>Bto_accB2_JETM‑2006359‑Bauhinia_tomentosa

‑‑‑ATGGCTTCGTTC‑‑‑ACTGTCCCATGCCCCAAGTGCCCTTCACCT‑‑‑‑‑‑‑‑‑‑‑‑‑‑‑‑‑‑‑‑‑GCA‑‑‑‑‑‑‑‑‑AACCCACAACGACAAACC‑‑‑CAGAAG‑‑‑‑‑‑‑‑‑‑‑‑‑‑‑‑‑‑‑‑‑GTGTCGTTTCAAGGAGGG‑‑‑TTGAGCTTGAAA‑‑‑‑‑‑‑‑‑‑‑‑TCCCTCGGATCG‑‑‑‑‑‑‑‑‑‑‑‑‑‑‑‑‑‑‑‑‑‑‑‑‑‑‑‑‑‑GTTCAGTGGCCT‑‑‑AATAGGAAGCGATTTTCTGTCTTGAAGTGTCAAGCAAAACTTAACGAGGCTACT‑‑‑‑‑‑‑‑‑‑‑‑TCT‑‑‑TCGAATTCTGCACCTCTACATGTTGCTGGATCTGAAGTTGCT‑‑‑TCGTCTCAGGAGAAGGATGACATTAATGGGAAAATCTCTGGTCCAAGCACT‑‑‑ATTCCAGATGCGTCTTCACTTTCTGCATTCATGACTCAAGTTTCAGACCTTGTAAAGCTTGTGGATTCCAGAGATATTGTGGAGCTGGAACTTAAGCAAGCTGGCATTGAGGTCATGATAAGAAAGAAGGAAGCAGTGCAGCCT‑‑‑‑‑‑CCATCA‑‑‑‑‑‑‑‑‑‑‑‑GCTCCTGTTCCA‑‑‑CCA‑‑‑‑‑‑CCAGCATACTATCAGACACTT‑‑‑CCT‑‑‑‑‑‑‑‑‑GCACCA‑‑‑CCACCACCTCCA‑‑‑‑‑‑‑‑‑GCCCCGGCACCTGTTTCT‑‑‑‑‑‑‑‑‑ACTTCTGCA‑‑‑AGCCCTCCACCTTCAAAAGGAGTACCCGCCTTACCT‑‑‑‑‑‑GCTCCACCAAAAGCAAGC‑‑‑ACATCATCACATCCACCTCTGAAATGTCCAATGGCTGGAACCTTTTACCGGAGTCCTGCACCGGGTGAACCAACATTTGTCAAGGTTGGAGATAAAGTGCAGAAAGGACAGGTTGTTTGCATTATTGAGGCTATGAAACTGATGAATGAAATTGAGGCTGATCGATCAGGAACGGTAGTTGAGGTACTAGCAGAAGATGGGAAGCCAGTCAGCGTAGGCACGCCT‑‑‑‑‑‑‑‑‑CTCCTTGTAATTGCACCA

>Car_accB2a_Ca_22093

‑‑‑‑‑‑‑‑‑‑‑‑‑‑‑‑‑‑‑‑‑‑‑‑‑‑‑‑‑‑‑‑‑‑‑‑‑‑‑‑‑‑‑‑‑‑‑‑‑‑‑‑‑‑‑‑‑‑‑‑‑‑‑‑‑‑‑‑‑‑‑‑‑‑‑‑‑‑‑‑‑‑‑‑‑‑‑‑‑‑‑‑‑‑‑‑‑‑‑‑‑‑‑‑‑‑‑‑‑‑‑‑‑‑‑‑‑‑‑‑‑‑‑‑‑‑‑‑‑‑‑‑‑‑‑‑‑‑‑‑‑‑‑‑‑‑‑‑‑‑‑‑‑‑‑‑‑‑‑‑‑‑‑‑‑‑‑‑‑‑‑‑‑‑‑‑‑‑‑‑‑‑‑‑‑‑‑‑‑‑‑‑‑‑‑‑‑‑‑‑‑‑‑‑‑‑‑‑‑‑‑‑‑‑‑‑‑‑‑‑‑‑TGCCTT‑‑‑GAAAGGAAGCAATCTTCTGTCTGGAAGGTTCAAGCACATCCTAATGAGGCTGCTACTATTGAAAATTCT‑‑‑TCAAATTCTGCACCTGTATTGGTCAATGAACCTAAAGTTGCT‑‑‑TCACCAAAAGAGGAAGATAACCATAATGGGAAACCCTCTGGTCCAAGCACT‑‑‑TCTACTGATGCATCTTCAGTTTCCACATTTATGAATCAAGTTTCAGAACTTGTTAAACTTGTGGATTCTAGAGATATTGTGGAGCTGCAACTGAAGCAAGCAGACTATGAGCTTACGATAAGGAAAAAGGAAGCTTTGGAGCCT‑‑‑‑‑‑CCTCCACAGGTG‑‑‑‑‑‑‑‑‑‑‑‑‑‑‑TCA‑‑‑CAA‑‑‑‑‑‑CCATATCCCTATCCGGCATAT‑‑‑GCTTCGCCACAAGCACCA‑‑‑CCACCACCA‑‑‑‑‑‑‑‑‑‑‑‑CCTCCAGTTGCTGTTTCT‑‑‑‑‑‑‑‑‑GCTCCTGCA‑‑‑AGCGCTCCACCTTCAAAAGTAGTTCCTGCCTTGCCT‑‑‑‑‑‑GCGCCTGGAAAAACCAGT‑‑‑GCATCATCTCATCCTCGGCTTAAATGTCCAATGGCAGGAACTTTCTATCGATGTCCCGGACCTGGTGAACCACCATTTGTCAAGGTGGGAGATAAAGTTCAGAAAGGGCAGGTTGTTTGCATTATTGAAGCTATGAAACTGATGAATGAAATTGAAGCTGATCAATCAGGAACAGTAACTGAGATACTAGTAGAAGATGGGAAACCAGTCAGTATAGACATGCCT‑‑‑‑‑‑‑‑‑CTTTTTGCAATTGCACCA

>Car_accB2b_Ca_10464

‑‑‑ATGGCTTCTTTA‑‑‑TCGGTTCCATGCCCTAAATGTCCTTCTCCT‑‑‑‑‑‑‑‑‑‑‑‑‑‑‑‑‑‑‑‑‑TCC‑‑‑‑‑‑‑‑‑ATATTGGGGTTGAAATCT‑‑‑AATAAG‑‑‑‑‑‑‑‑‑‑‑‑‑‑‑‑‑‑‑‑‑ATCTTGTTTGGAAGTGGG‑‑‑TTGAGCTTGAAGCATTCT‑‑‑CAATCATTTGGATCTTTGTCTGCTGAGTCAGCATCC‑‑‑TTTGGGATTAAGTGTCTT‑‑‑AACAGGAAGCAATTTCCTCTC‑‑‑AAGACTCAAGCACAACGTAACGAGGCTGCTGCCGTCGAAAAT‑‑‑‑‑‑TCAAAGTCTGTACCTGTACCGGTCAATGGACCTACTGTTGCT‑‑‑GCAATAAAAGAGAAAGAAAACCAAAATGGAAATGTCTCTGGT‑‑‑GACTCT‑‑‑GTTGAAGATGCATCTTCAGTTTCTGCATTCATGACTCAAGTTGCTGACCTTGTAAAACTTGTGGATTCTAGAGATATTGTGGAACTGCAACTGAAGCAATCAGACTGTGAGCTCTTGATAAGAAAAACAGAAGCTTTGCAGCCT‑‑‑‑‑‑CCACCGGCCGCAGCCATGCCTCAA‑‑‑‑‑‑‑‑‑CAA‑‑‑‑‑‑CCGTTGTACTATCAAACACTT‑‑‑CCT‑‑‑‑‑‑‑‑‑TCGCCA‑‑‑CCACCCCCA‑‑‑‑‑‑‑‑‑‑‑‑GCACCGGCAGCTGCTTCT‑‑‑‑‑‑‑‑‑GCTCCTGCAGGCTCTCCTCCGCCTTCGAAAGCAACACCTGCGTTACCT‑‑‑‑‑‑CCCCCC‑‑‑AAAAGAAGT‑‑‑GCATCCTCTCATCCACAGCTGAAATGTCCAATGGCAGGAACCTTTTACAGGTGTCCGGGACCTGGTGAACCTCCGTTTGTCAAGGTGGGAGATAAAGTGCAAAAAGGCCAGGTTGTTTGCATTATTGAGGCTATGAAACTGATGAATGAAATTGAGGCTGATCAATCAGGAACAATAACTGAGATACTAGTTGAAGATGGAAAACCGGTCAGCATAGACCTGCCT‑‑‑‑‑‑‑‑‑CTTTTCGTAATAGCTCCA

>Car_accB2c_Ca_21112

‑‑‑ATGGCTTCTTTC‑‑‑ACTGTCCCTTGTCCCAAATGTCTCTCTTTT‑‑‑‑‑‑‑‑‑‑‑‑‑‑‑‑‑‑‑‑‑CAT‑‑‑‑‑‑‑‑‑CATTTTGGTTTGAAATCT‑‑‑CAGACCCCAACAAAGAATATTCAA‑‑‑‑‑‑‑‑‑‑‑‑‑‑‑‑‑‑‑‑‑‑‑‑TTGGGGTTCAGCAAATCT‑‑‑CAGTCATTTGGATCTGTGAGTCGTGATTCAGTTTCA‑‑‑ACTGGGATTCAGTGCCTT‑‑‑GAAAGGAAGCAATCTTCTGTCTGGAAGGTTCAAGCACATCCTAATGAGGCTGCTACTATTGAAAATTCT‑‑‑TCAAATTCTGCACCTGTATTGGTCAATGAACCTAAAGTTGCT‑‑‑TCACCAAAAGAGGAAGATAACCATAATGGGAAACCCTCTGGTCCAAGCACT‑‑‑TCTACTGATGCATCTTCAGTTTCCACATTTATGAATCAAGTTTCAGAACTTGTTAAACTTGTGGATTCTAGAGATATTGTGGAGCTGCAACTGAAGCAAGCAGACTATGAGCTTACGATAAGGAAAAAGGAAGCTTTGGAGCCT‑‑‑‑‑‑CCTCCACAGGTG‑‑‑‑‑‑‑‑‑‑‑‑‑‑‑TCA‑‑‑CAA‑‑‑‑‑‑CCATATCCCTATCCGGCATAT‑‑‑GCTTCGCCACAAGCACCA‑‑‑CCACCACCA‑‑‑‑‑‑‑‑‑‑‑‑CCTCCAGTTGCTGTTTCT‑‑‑‑‑‑‑‑‑GCTCCTGCA‑‑‑AGCGCTCCACCTTCAAAAGTAGTTCCTGCCTTGCCT‑‑‑‑‑‑GCGCCTGGAAAAACCAGT‑‑‑GCATCATCTCATCCTCGGCTTAAATGTCCAATGGCAGGAACTTTCTATCGATGTCCCGGACCTGGTGAACCACCATTTGTCAAGGTGGGAGATAAAGTTCAGAAAGGGCAGGTTGTTTGCATTATTGAAGCTATGAAACTGATGAATGAAATTGAAGCTGATCAGTCAGGAACAATAACTGAGATACTAGTAGAAGATGGGAAACCAGTCAGTATAGACATGACTTGTAATTTATTATTTGTTTTTTGGTTT

>Cca_accB2a_C.cajan_18788

‑‑‑ATGGCGTCGTTC‑‑‑ACGGTCCCTTGCCCCAAGTGCGTTCCTTTT‑‑‑‑‑‑‑‑‑‑‑‑‑‑‑‑‑‑‑‑‑GCT‑‑‑‑‑‑‑‑‑CGTTTGGGACTGAACTCT‑‑‑CAGACCCAGCAACGGAACGCGCCAATGGTCTCGTATGGGAGCGGG‑‑‑TTGGCCTTGAAGAAATCA‑‑‑CTGTCATTTGGATCT‑‑‑‑‑‑‑‑‑TCGAATGCACCA‑‑‑AATGGGATTCAGTGCCTT‑‑‑AACAGGAAGCAATTTTCTGTCTTGAAATCTCAAGCAAAAAAAAATGAGGCCGTTACTGTCGAA‑‑‑‑‑‑‑‑‑‑‑‑AATTCAGCACCTGTACAGGCAAATGGACCTAAAGTTGCT‑‑‑TCACCAGAAGAGAAAGTTGACCATAATGGAAAACCCTCTGGTCCAAGCACT‑‑‑TCCATTGATGCATCTTCAATTTCTGCATTTATTAATCAAGTTTCAGATCTTGTTAAACTTGTGGACTCTAGAGATATTATGGAGCTGCAACTTAAGCAAGCAGACTGTGAGCTCGTGATAAGAAAAAAGGAAGCATTACAGCCT‑‑‑‑‑‑CCACCAGCTACTGTTATGGCTCCAGTGTCA‑‑‑CAACCATATCCATATCCATATCCCACACAT‑‑‑CCT‑‑‑‑‑‑‑‑‑ACGCCA‑‑‑GCACCACCA‑‑‑‑‑‑‑‑‑‑‑‑CCACCCGTTGCTGCTTCC‑‑‑‑‑‑‑‑‑ACTCCTGCA‑‑‑AGCCCTCCACCTTCAAAA‑‑‑‑‑‑‑‑‑GCCTTACCT‑‑‑‑‑‑GCTCCCGGAAAAGCAAGC‑‑‑ACATCGTCTCATCCAGCGCTGAAAAGTCCAATGGCAGGAACCTTTTATCGAAGTCCAGCGCCTGGCGAGCCTCCATTTGTCAAGGTTGGAGATAAAGTGCAGAAAGGGCAGGTCGTTTGCATCATCGAGGCTATGAAATTAATGAATGAAATTGAAGCTGATCAAGCAGGAACAATAACTGAGATAATGGCTGAGGATGGTAAAGCAGTCAGTTTAGACACGCCT‑‑‑‑‑‑‑‑‑CTTTTTGTAATTGTACCA

>Cca_accB2b_C.cajan_22132

‑‑‑ATGGCCTCTTTC‑‑‑TCCGTCCCATGCCCCAAGTGCCCTTCTTCT‑‑‑‑‑‑‑‑‑‑‑‑‑‑‑‑‑‑‑‑‑TTC‑‑‑‑‑‑‑‑‑CCTTTGGGGTTGAAATCC‑‑‑CAAAAG‑‑‑‑‑‑‑‑‑‑‑‑‑‑‑‑‑‑‑‑‑GCCTCATTTCAAAGTGGG‑‑‑TTGCGCTTGAAGCCTTCT‑‑‑ATATCATTTGGATCTTTGTCCGTTGAATCTGTTGCA‑‑‑TCTAGGATTCAA‑‑‑‑‑‑‑‑‑‑‑‑‑‑‑‑‑‑‑‑‑‑‑‑‑‑‑‑‑‑‑‑‑‑‑‑‑‑‑‑‑‑‑‑‑‑‑‑‑‑‑‑‑‑‑‑‑GCTGCTAATGTTGAAAAT‑‑‑‑‑‑TCTAATTCTGCCTCAATAGTGGTCAATGGCCCTAAGGTTGCT‑‑‑TCATCAAAAGAAAAACAAGACCAGAATGGAAAAGTATCTGGT‑‑‑GGCACC‑‑‑AGCGCAAATGAATCTTCAATTACTACATTCATGTCTCAAGTTTCAGACCTTGTAAAACTTGTGGATTCAAGAGATATTGTGGAACTTCAACTTAAGCAATCAAACTGTGAGCTCATGATAAGAAAAAAAGAAGCATTGTTGCCT‑‑‑‑‑‑CCACCG‑‑‑‑‑‑GTTGTAGCCCCAGCATCA‑‑‑TCA‑‑‑‑‑‑CCTATGCAATATGCCACATTT‑‑‑CCT‑‑‑‑‑‑‑‑‑TCTCCA‑‑‑CCAATGCCT‑‑‑‑‑‑‑‑‑‑‑‑CCACTAGCAACTGCTTCT‑‑‑‑‑‑‑‑‑GCCCCTACT‑‑‑AGCTCTCTGACTCCAAAAGCAGCACCGAGCTTACCT‑‑‑‑‑‑ACCCCCAAAAAAGCAAGC‑‑‑ACATTTTCTCACCCACCGATAAAAAGTCCAATGGCAGGAACGTTCTACAAAAGTCCAGCACCTGGTGAACCTCCATTTGTCAAGGTGGGAGATAAAGTGAAGAAAGGACAGGTTGTTTGCATTATTGAGGCTATGAAACTGATGAATGAAATTGAGGCTGATCAGTCAGGAACAGTAGCTGAGATACTAGCTGAGGATGGGAAACCAGTTAGTGTAGACACGCCT‑‑‑‑‑‑‑‑‑CTTTTTGTAATAGCTCCA

>Cca_accB2c_Scaffold132424

‑‑‑ATGGCGTCGTTC‑‑‑ACGGTCCCTTGCCCCAAGTGCGTTCCTTTT‑‑‑‑‑‑‑‑‑‑‑‑‑‑‑‑‑‑‑‑‑GCT‑‑‑‑‑‑‑‑‑CGTTTGGGACTGAACTCT‑‑‑CAGACCCAGCAACGGAACGCGCCAATGGTCTCGTATGGGAGCGGG‑‑‑TTGGCCTTGAAGACATCA‑‑‑CTGTCATTTGGATCT‑‑‑‑‑‑‑‑‑TCGAATGCACCA‑‑‑AATGGGATTCAGTGCCTT‑‑‑AACAGGAAGCAATTTTCTGTCTTGAAGTATCAAGCAAAAACAAATGAGGCCGTTACTGTTGAA‑‑‑‑‑‑‑‑‑‑‑‑AATTCTGCACCTGTACAGGTCAATAGACCTAAAGTTGCT‑‑‑TCACCAGAAGAGAAAGTTGACCATAATGGAAAACCCTCTGGTCCAAGCACT‑‑‑TCCGTTGATGCATCTTCAATTTCTGCATTTATGAATCAAGTTGCAGATCTTGTTAAACTTGTGGACTCTAGAGATATTATGGAGCTTGAACTTAAACAAGCAGACTGTGAGCTCGTGATAAGAAAAAAGGAAGCATTACAGCCT‑‑‑‑‑‑CCACCAGCTACTGTTATGGCTCCAGTGTCA‑‑‑CAA‑‑‑‑‑‑CCATATCCATATCCCACACAT‑‑‑CCT‑‑‑‑‑‑‑‑‑ATGCCG‑‑‑GTACCACCA‑‑‑‑‑‑‑‑‑‑‑‑CCACCCGTTGCTGCTTCC‑‑‑‑‑‑‑‑‑GCTCCTGCA‑‑‑AGCCCTCCACCTTCAAAA‑‑‑‑‑‑‑‑‑GCCTTACCT‑‑‑‑‑‑GCTCCTGGAAAAGAAAGC‑‑‑ACATCATCTCATCCACCGCTGAAAAGTCCAATGGCTGGAACCTTTTATCGAAGTCCAGCGCCTGGCGAACCTCCATTTGTCAAGGTTGGAGATAAAGTGCAGAAAGGGCAGGTCGTTTGCATTATCGAGGCTATGAAATTAATGAATGAAATTGAAGCTGATCACGCAGGAACAATAACCGAGGTAATAGCTCAGGATGGTAAAGCAGTCAGTTTAGACACGCCT‑‑‑‑‑‑‑‑‑CTTTTTGTAATTGTACCA

>Ccan_accB2_RKFX‑2046821‑Cercis_canadensis

‑‑‑ATGGCTTCGTTC‑‑‑ACGGTCCCATGCCCCAAGTGCCTTTCGCCT‑‑‑‑‑‑‑‑‑‑‑‑‑‑‑‑‑‑‑‑‑GCA‑‑‑‑‑‑‑‑‑CATTTGGGATTGAGCTCA‑‑‑CAGAACCTGCAACGACAGACCCAGAAGGTGTCGTTTCAAAGAGGG‑‑‑TTGAGCTTGAAA‑‑‑‑‑‑‑‑‑‑‑‑TCGTTCGAATCTTCGGTTGTTGATCCAGCGTCA‑‑‑TCTGAGGTTCAGTGCCTT‑‑‑AACAGGAAGCAATTCTCTGTCTTGAAGTGTCAAGCAAAACTTAATGAGGCTACTGTTGCCAAAAGTTCT‑‑‑TCAAATTCTGCACCTGCACTTGTTGCTGGATCTGAAGTTGCT‑‑‑TCATCAAAGGAGAAAGATGACCTTAATGGGAAAGTCTCTAGTCCAAGCACT‑‑‑ATTCCAGATGCATCTTCAATTTCTGCATTCATGACTCAAGTTTCAGACCTTGTAAAACTTGTGGATTCCAGAGATATTGTGGAGCTGCAACTTAAGCAATCTGATTGTGAGGTCATGATAAGAAAGAAGGAAGCATTGCAGCCT‑‑‑‑‑‑CCACCA‑‑‑‑‑‑‑‑‑‑‑‑GCCCCAGTGTCA‑‑‑CCA‑‑‑‑‑‑CCAGTGCACTATCACGCACTT‑‑‑CCT‑‑‑‑‑‑‑‑‑TCACCA‑‑‑CCACCACCTCCA‑‑‑‑‑‑‑‑‑GCCCCAGCATCTGTTTCT‑‑‑‑‑‑‑‑‑ACCCCTGCA‑‑‑AGCCCTCCACCTTCAAAAGCAGCACCCGCCTTACCT‑‑‑‑‑‑TCTCCTGCAAAAACAAGC‑‑‑ACATCATCTCATCCTCCTCTGAAATGTCCAATGGCTGGAACCTTTTATCGGAGTCCTGCACCTGGTGAACCAGTATTTGTCAAGGTTGGAGATAAAGTGCAGAAAGGCCAGGTTGTTTGCATTATTGAGGCTATGAAACTGATGAATGAAATTGAGGCTGATCAATCAGGAACAGTAGTTGAGATACTTGTAGATGATGGGAAACCAGTCAGTGTAGACATGCCT‑‑‑‑‑‑‑‑‑CTTTTTGTAATTGCACCA

>Cof_accB2_RKLL‑2007824‑Copaifera_officianalis

‑‑‑ATGGCTTCGTTC‑‑‑ACTGTTCCATGTCCTAAGTGCATTTCTCCT‑‑‑‑‑‑‑‑‑‑‑‑‑‑‑‑‑‑‑‑‑TCC‑‑‑‑‑‑‑‑‑CGCTTGGGATTGAAATCG‑‑‑AAGATTCTTCAGGGACAGGCGCAGAAGTTGTCGTTTCAAAGTGGG‑‑‑ACGAGCTTAAAGGCATGC‑‑‑CCGTTATTCGGATCT‑‑‑‑‑‑‑‑‑‑‑‑‑‑‑‑‑‑TCA‑‑‑TCTGGGTTGCAGTGCGTC‑‑‑AACAAGGAACAAATTCCAGTTGGGAAGATTCAAGCAAATTTTAATGAGGCCACCACTGCTAAAAGCTCT‑‑‑TCGAATTCTGCATCTGTT‑‑‑GCCCCTGAATCTGGAGTTGGT‑‑‑TCTTCAAAT‑‑‑‑‑‑‑‑‑‑‑‑GAGAATGGCAAAATCTCTGGTCCAAGTACT‑‑‑TCTTTAGATCAATCTGCAGTCTCTGCATTCATGAATCAAGTAGCTGACCTGGTAAAACTTGTGGATTCCCGTGATATTGTTGAACTGGAACTTAAGCAATTGGATTGTGAGGTTATGATAAGAAAGAAGGAAGCTTTGCAGCCT‑‑‑‑‑‑CCACCAGCTGCTGCAGTAGCCCCAATGCCA‑‑‑CCA‑‑‑‑‑‑TCAATGCCTTAT‑‑‑‑‑‑TAT‑‑‑CCT‑‑‑‑‑‑‑‑‑CCTCCA‑‑‑CCACCACCAGCA‑‑‑‑‑‑‑‑‑ACGGCAGCCCCTCCTTCT‑‑‑‑‑‑‑‑‑ACTCTTGCA‑‑‑AGCCCTCCACCTTCAAAACCAGCACCTGCTTTACCG‑‑‑‑‑‑TCCCCTCCAAAAGCAAGC‑‑‑ACTTCTTCTCATCCACCATTGAAATGCCCAATGGCTGGAACCTTTTACAGGAGTCCTGGACCTGGTGAACCACCATTTGTGAAGGTGGGAGATAAAGTGCAGAAAGGCCAGGTTGTTTGCATTATTGAGGCTATGAAACTGATGAATGAAATTGAAGCTGATCAATCAGGAACAATAACTGAGATTCTAGTTGATGATGGTAAAACAGTCAGCATAGACACGCCT‑‑‑‑‑‑‑‑‑CTGCTTGTTATAGTGCCA

>Cre_accB_XM_001700390

‑‑‑ATGTTTGCCCTT‑‑‑CGCGCTCCT‑‑‑‑‑‑‑‑‑‑‑‑‑‑‑‑‑‑‑‑‑‑‑‑‑‑‑‑‑‑‑‑‑‑‑‑‑‑‑‑‑‑‑‑‑‑‑‑‑‑‑‑‑‑‑‑‑‑‑‑‑‑‑‑‑‑‑‑‑‑‑‑‑‑‑‑‑‑‑‑‑‑‑‑‑‑‑‑‑‑‑‑‑‑‑‑‑‑‑‑‑‑‑‑‑‑‑‑‑‑‑‑‑‑‑‑‑‑‑‑‑‑‑‑‑‑‑‑‑‑‑‑‑‑‑‑‑‑‑‑‑‑‑‑‑‑‑‑‑‑‑‑‑‑‑‑‑‑‑‑‑‑‑‑‑‑‑‑‑‑‑‑‑‑‑‑‑‑‑‑‑‑‑‑‑‑‑‑‑‑‑‑‑‑‑‑‑‑‑‑‑‑‑‑‑‑‑‑‑‑‑‑‑‑‑‑‑‑‑‑‑‑‑‑‑‑‑‑‑‑‑‑‑‑GTGGCCCGGCCCTCCGCCGCTCGGAGTGTGCAGTCT‑‑‑‑‑‑GGTGTAGCCAGCCGTGTGCAGCGCGTCGTGCCCCGCGCGGCT‑‑‑AAGACCGAGGAGGTTGAGGCTGCTACCAACGGTGCCGCT‑‑‑‑‑‑GCCTCG‑‑‑AACGGCCATGCTTCGGCCAACATGATGCAGTTCGACGAGCTGTCTGATATCATTCGGCTGGTGCATGATACCGACATTGTCGAGTTCGAGCTGAAGAGCAAGCGTTTCAGCCTGAGCGTGCGCAAGAAGGAGGCTCTGCAGGCT‑‑‑‑‑‑GAGCAGGCGGCTGCCTACCAGGCCATGCTG‑‑‑‑‑‑‑‑‑‑‑‑CCCCAGATGCTGGCGGCTGCC‑‑‑CCC‑‑‑‑‑‑GTGGCCCCCATC‑‑‑‑‑‑‑‑‑‑‑‑‑‑‑‑‑‑‑‑‑GCTGCCCCCGTGGCTGCC‑‑‑‑‑‑‑‑‑GCCCCTGCC‑‑‑CCTGCCCCGGCGCCAGCTGCCGCCCCCGCGGCACCGGCCGCCGCGCCAGCGCCCGCCGCA‑‑‑GCGCCCAAGGGCATTGAGGTTGCCTCTCCCATGGGTGGCACCTTCTACCGCAAGCCTGCCCCCGGTGAGCCGGAGTTCGCCAAGGTTGGCGACAAGGTGAAGAAGGGCCAGACCGTGTGCATCATTGAGGCCATGAAGCTGATGAACGAGATCGAGGCCGAGGTTGGCGGTGAGGTGATCAAGTTCCTGGTGGAGAACGGCCAGCCCGTGACTGTGGGCCAGCCC‑‑‑‑‑‑‑‑‑ATCATGATCATCAAGCCA

>Gma_accB2a_Glyma.13g057400

‑‑‑ATGGCCTCTTTC‑‑‑TCGGTCCCATGCCCCAAGTGTCCTACAACT‑‑‑‑‑‑‑‑‑‑‑‑‑‑‑TCTTCTTCTCCC‑‑‑‑‑‑CCTTTGGGGTTGAAATCT‑‑‑CTAAAT‑‑‑‑‑‑‑‑‑‑‑‑‑‑‑‑‑‑‑‑‑GTTTCATTTCAAAGGGTG‑‑‑TTGAGCTTGAAGCCTTCT‑‑‑CTTTCATTCGGATCTTTGTCTGCTGAATCTGCTGCA‑‑‑TCAAGGATTCAGTGCCTT‑‑‑AACAGGAAGCAATTTTCTGTTCTGAAG‑‑‑‑‑‑‑‑‑‑‑‑‑‑‑‑‑‑‑‑‑GCTACTAAAGTTGAAAAT‑‑‑‑‑‑TCCAATTCTGCCCCTGTAATGGTCAATGGACCTAATGTTGCT‑‑‑TCATCGAAAGAAAAAGAAGTGCATAATGGAAAACTCTCTGGT‑‑‑GGTACT‑‑‑ATCCCAGATGATGCTTCAATTATTGCATTCATGTCTCAAGTTTCAGACCTTGTAAAACTCGTGGATTCGAGAGATATTGTGGAACTTCAACTTAAGCAATCAGACTGTGAGCTCATGATAAGAAAAAAAGAAGCATTGCAGCCT‑‑‑‑‑‑CCACCA‑‑‑‑‑‑GTTATAGCCCCAACATCC‑‑‑CCA‑‑‑‑‑‑CCAATGCACTATGCCACAGTT‑‑‑CCT‑‑‑‑‑‑‑‑‑TCTCCG‑‑‑‑‑‑‑‑‑‑‑‑‑‑‑‑‑‑‑‑‑‑‑‑CCACCACCACCAGCAGCT‑‑‑‑‑‑‑‑‑GCTCCTGCT‑‑‑AGCTCTGCACCTCCAAAAGCAGTTCCTGCCTTGCCT‑‑‑‑‑‑TCCCCTGCAAAAGCAGGC‑‑‑ACATCTTCTCACCCAACGCTGAAATGTCCGATGGCAGGAACCTTCTACAGGAGTCCAGCACCTGGTGAACCTGCATTTGTCAAGGTGGGAGATAAAGTGCAGAAAGGCCAGGTTATTTGCATTATCGAGGCTATGAAACTGATGAATGAAATTGAGGCTGATCAGTCAGGAACAATAGCTGAGGTATTAGCTGAGGATGGGAAACCAGTCAGTGTGGACACGCCT‑‑‑‑‑‑‑‑‑CTTTTTGTAATAGTTCCA

>Gma_accB2b_Glyma.18g265300

‑‑‑ATGGCGTCATTC‑‑‑ACGATCCCTTGCCCCAAGTGCGTTGTTGTT‑‑‑‑‑‑‑‑‑‑‑‑‑‑‑CCTTTTGCT‑‑‑‑‑‑‑‑‑CATTTGGGACTGAACTCT‑‑‑CAGACCCAGCAACGGAACGCG‑‑‑‑‑‑‑‑‑‑‑‑‑‑‑‑‑‑‑‑‑‑‑‑‑‑‑CTGGGGTTGAAGAAATCG‑‑‑CTCTCATTTGGATCTTTGAGTAGTGATTCGGCGCCA‑‑‑AATGGGATTCAGTGCCTT‑‑‑AACAAGAAGCAATCTTCTGTCTGGAAGCTTCAAGCACAACCAAAAGAGGCTGTTACTGTTGAA‑‑‑‑‑‑‑‑‑‑‑‑AATTCTGCACCTGTACAGGTCAATGGACCTAAAATTGCT‑‑‑CCACCAGAAGAGAAGGATGACCATAATGGAAAACCCTCTGGTCCAAGCACT‑‑‑TCTGCTGATGCATCTTCAATTTCTGCATTTATGAACCAAGTTTCAGATCTTGTTAAACTTGTGGACTCTAAAGATATTATGGAGCTGCAACTCAAGCAAGCAAACTGTGAACTCGTGATAAGAAAGAAGGAAGCTTTGCTGCCT‑‑‑‑‑‑CCACCAGCTACATTTGTAGCTCCAGTGTCA‑‑‑CAA‑‑‑‑‑‑CCATTTCCATATCCCACAAAT‑‑‑TCT‑‑‑‑‑‑‑‑‑CTTCCA‑‑‑GCAGCACCA‑‑‑‑‑‑‑‑‑‑‑‑CCTCCAGTTGCTACTTCC‑‑‑‑‑‑‑‑‑ACTCCTGCA‑‑‑AGTTCTCCATCTTCAAAAGCAGCTCCTGCCTTACCT‑‑‑‑‑‑‑‑‑CCTGCAAAAGCAAGC‑‑‑AAATCATCTCATCCGGCACTGAAATGTCCAATGGCAGGAACCTTTTATCGAAGTCCAGCACCTGGCGAACCTCCATTTGTCAAGGTGGGAGATAAAGTGCAGAAGGGGCAGGTCATTTGCATTATTGAGGCTATGAAACTAATGAATGAAATTGAAGCTGATCAATCTGGAACAGTAGCTGAGGTTGTAGCAGAGGATGGGAAACCAGTCAGTGTAGACACGCCT‑‑‑‑‑‑‑‑‑CTTTTTGTAATTGTACCA

>Gma_accB2c_Glyma.19g028800

‑‑‑ATGGCCTCCTTC‑‑‑TCGGTCCCATGCCCCAAGTGTCCTACAACT‑‑‑‑‑‑‑‑‑‑‑‑‑‑‑TCTTCTTCGTCTTCTCTCCCTTTGGGGTTGAATTCT‑‑‑CAAAAG‑‑‑‑‑‑‑‑‑‑‑‑‑‑‑‑‑‑‑‑‑GTCTCATTTCAAAGTGGG‑‑‑TTGATCTTGAAGCCTTCT‑‑‑CTTTCATTCGGATCTTTGTCTGCTGAATCTGCTGCA‑‑‑TCAAGGATTCAGTGCCTT‑‑‑AACAGGAAGCAATTTTCTGTTCTGAAG‑‑‑‑‑‑‑‑‑‑‑‑‑‑‑‑‑‑‑‑‑GCTACTAAAGTTGAAAAT‑‑‑‑‑‑TCCAACTCTGCCCCTGTAACGGTCAATGGACCTACTGTTGCT‑‑‑TCATCAAAAGAAAACCAAGTGCATAATGGAAAACTCTCTGAT‑‑‑ACTACT‑‑‑ATCCCAGATGAAGCTTCAATTATTGCATTCATGTCTCAAGTTTCAGACCTTGTAAAACTTGTGGATTCGAGAGATATTGTGGAACTTCAACTTAAGCAATCAGACTGTGAGCTCATGATAAGAAAAAAAGAAGCATTGCAGCCT‑‑‑‑‑‑CCACCA‑‑‑‑‑‑ATTATAGCCCCAGCACCA‑‑‑CCA‑‑‑‑‑‑CCAATGCACTATGCCACTTTT‑‑‑CCT‑‑‑‑‑‑‑‑‑TCTCCG‑‑‑TCTTCG‑‑‑‑‑‑‑‑‑‑‑‑‑‑‑CCGCTACCAGCAGAAGCT‑‑‑‑‑‑‑‑‑GCTCCTGCT‑‑‑AGCTCTGCACCTCCAAAAGCAGCTCCTGCCTTGCCT‑‑‑‑‑‑TCCCCCGGAAAAGCAAGC‑‑‑ACATCTTCTCACCCACCACTGAAATGTCCAATGGCAGGAACCTTCTATAGGAGTCCAGCACCTGGTGAACCTGCATTTGTCAAGGTGGGAGATAAAGTGAAGAAAGGCCAGGTTATTTGCATTATCGAGGCTATGAAACTGATGAATGAAATTGAGGCTGATCAGTCAGGAACAATAGCTGAGGTATTAGCTGAGGATGGGAAACCAGTCAGTGTAGACATGCCT‑‑‑‑‑‑‑‑‑CTTTTTGTAATAGTTCCA

>Gpo_accB2a_VLNB‑2013644

‑‑‑ATGGCTTCTTTC‑‑‑TCGGTCCCATGCCCCAAGTGCCCTTCTCCT‑‑‑‑‑‑‑‑‑‑‑‑‑‑‑‑‑‑‑‑‑GCC‑‑‑‑‑‑‑‑‑GCTTTGGGATTGAAATCC‑‑‑CACAAG‑‑‑‑‑‑‑‑‑‑‑‑‑‑‑‑‑‑‑‑‑CTCTCGTTTCGAAGCGGG‑‑‑TTGAGCTTGAAGCATTTT‑‑‑CAATCATTCGGATCTTTGTCTTCTGAGTCAGCTTCA‑‑‑TCTGGGATTCGGTGCCTT‑‑‑AACAGGAAGCAATTTTCTGTCCGGAAGTTTCAAGCACAACTTAATGAGGCTGCTAGTGTTGAAAATTCT‑‑‑TCTAATTCTGCACCTGTACTGGTCAATGGACCTAATGTTGCT‑‑‑TCATCAAAAGATAAA‑‑‑GATCAGAATGGGAAACTCTCTGGT‑‑‑GACACT‑‑‑GCTGCAGATGCATCTTCAGTTTCTGCATTCATGGCTCAAGTTTCAGACCTTGTAAAACTAGTGGATTCCAGAGATATTGTGGAACTGCAACTTAAGCAATCAGACAGTGAGCTGATGATAAGAAAAAAGGAAGCTTTGCAGCCT‑‑‑‑‑‑CCACCGGCCACAGTTATAGCTCCAGCATCA‑‑‑CCA‑‑‑‑‑‑CCGTTGCATTATCCCACACTT‑‑‑CCT‑‑‑‑‑‑‑‑‑TCTCCG‑‑‑‑‑‑‑‑‑‑‑‑‑‑‑‑‑‑‑‑‑‑‑‑CCACTGCCAGCTTCTCCT‑‑‑‑‑‑‑‑‑GCTGCTGCA‑‑‑AGCTCTCCACCTTCAAAAGCAGCACCTGCCTTACCT‑‑‑‑‑‑TCCCCTGGAAAAACAAGC‑‑‑GCATCATCTCACCCACCGCTGAAATGTCCAATGGCAGGAACCTTTTATCGGAGTCCAGCACCTGGTGAAGCTCCATTTGTCAAGGTGGGAGACAAAGTGCAGAAAGGCCAGATAATA‑‑‑‑‑‑‑‑‑‑‑‑‑‑‑‑‑‑‑‑‑‑‑‑‑‑‑‑‑‑‑‑‑‑‑‑‑‑‑‑‑‑‑‑‑‑‑‑‑‑‑‑‑‑‑‑‑‑‑‑‑‑‑‑‑‑‑‑‑‑‑‑GCTGAGGATGGGAAACCAGTCAGTATAGATACGCCT‑‑‑‑‑‑‑‑‑CTTTTTGTGATAGTTCCA

>Gpo_accB2b_VLNB‑2013645

‑‑‑ATGGCTTCTTTC‑‑‑TCGGTCCCATGCCCCAAGTGCCCTTCTCCT‑‑‑‑‑‑‑‑‑‑‑‑‑‑‑‑‑‑‑‑‑GCC‑‑‑‑‑‑‑‑‑GCTTTGGGATTGAAATCC‑‑‑CACAAG‑‑‑‑‑‑‑‑‑‑‑‑‑‑‑‑‑‑‑‑‑CTCTCGTTTCGAAGCGGG‑‑‑TTGAGCTTGAAGCATTTT‑‑‑CAATCATTCGGATCTTTGTCTTCTGAGTCAGCTTCA‑‑‑TCTGGGATTCGGTGCCTT‑‑‑AACAGGAAGCAATTTTCTGTCCGGAAGTTTCAAGCACAACTTAATGAGGCTGCTAGTGTTGAAAATTCT‑‑‑TCTAATTCTGCACCTGTACTGGTCAATGGACCTAATGTTGCT‑‑‑TCATCAAAAGATAAA‑‑‑GATCAGAATGGGAAACTCTCTGGT‑‑‑GACACT‑‑‑GCTGCAGATGCATCTTCAGTTTCTGCATTCATGGCTCAAGTTTCAGACCTTGTAAAACTAGTGGATTCCAGAGATATTGTGGAACTGCAACTTAAGCAATCAGACAGTGAGCTGATGATAAGAAAAAAGGAAGCTTTGCAGCCT‑‑‑‑‑‑CCACCGGCCACAGTTATAGCTCCAGCATCA‑‑‑CCA‑‑‑‑‑‑CCGTTGCATTATCCCACACTT‑‑‑CCT‑‑‑‑‑‑‑‑‑TCTCCG‑‑‑‑‑‑‑‑‑‑‑‑‑‑‑‑‑‑‑‑‑‑‑‑CCACTGCCAGCTTCTCCT‑‑‑‑‑‑‑‑‑GCTGCTGCA‑‑‑AGCTCTCCACCTTCAAAAGCAGCACCTGCCTTACCT‑‑‑‑‑‑TCCCCTGGAAAAACAAGC‑‑‑GCATCATCTCACCCACCGCTGAAATGTCCAATGGCAGGAACCTTTTATCGGAGTCCAGCACCTGGTGAAGCTCCATTTGTCAAGGTGGGAGACAAAGTGCAGAAAGGCCAGGTTATTTGCATTATCGAGGCTATGAAACTGATGAATGAAATTGCAGCTGATCAATCAGGAACAATAACTGAGATACTAGCGGAGGATGGGAAACCAGTCAGTGTAGACACGCCA‑‑‑‑‑‑‑‑‑CTTTTGGTAATTGTACCA

>Lal_accB2a_LAGI01_35736

‑‑‑ATGTCTTCTTTT‑‑‑TCTGTCCCATGCCCAAAATGCCCT‑‑‑‑‑‑‑‑‑‑‑‑‑‑‑CCTCATTCTTCTTCA‑‑‑‑‑‑‑‑‑CTTTTGGGGTTGAAAGCT‑‑‑ACCAAG‑‑‑‑‑‑‑‑‑‑‑‑‑‑‑‑‑‑‑‑‑TTATCTTTTCAAAGCGGGTTCATGTGTTTGAAGCAGTTT‑‑‑CAATCATTTGGATCTTTGTCTGCTGAGTCAGCAACA‑‑‑TTTGGGATTCGGTGCCTT‑‑‑AACAGAAAGCAAATTTCTGGTGGGAAGCTTCAAGCAAAACTCAATGAGGCTGCTACCGTTGAAAAT‑‑‑‑‑‑TCAAATTCTGCACCTGTACTC‑‑‑AACGGACCTAGTGTCGTCACTTCATCAAAAGAGGAAGAAGATAATAACGGGGCACTATCTAAT‑‑‑GGCACTGCTGCTGCAGATGAATCTTCAATTTCTGCTTTCATGGCTCAAGTTGCAGACCTTGTAAAACTTGTAGATTCCAGAGATATCGTGGAACTGGAACTAAAGCAATCAAACTGCGAGGTCTTGATAAGAAAAAAGGAAGCTTTGCAGCCT‑‑‑‑‑‑CCACCA‑‑‑‑‑‑‑‑‑‑‑‑GCCCCAGCAGCA‑‑‑CCA‑‑‑‑‑‑CAATATCCGTATCCAACTTAT‑‑‑CAT‑‑‑‑‑‑‑‑‑GCTCCG‑‑‑CCACCAGCACCA‑‑‑‑‑‑‑‑‑CCAGCACCACCTGCTGCA‑‑‑‑‑‑‑‑‑GCTCCTGCC‑‑‑AGCTCTCCACCTTCTAAGGCAGCACCTGCCCTACCT‑‑‑‑‑‑TCCCCAGGAAAGGCAATT‑‑‑GCGTCATCTCACCCACCACTGAAATGTCCAATGGCAGGGACCTTTTATCGGAGCCCAGCACCTGGTGAACCGGCATTTGTCAAAGTGGGAGACAAAGTGAAGAAAGGCCAGGTTATTTGCATTATCGAGGCTATGAAACTGATGAATGAAATTGAGGCTGATCAATCAGGAACAATAGCTGAGGTAATCGCTGAGGATGGGAAACCAGTCAGTGTAGACACGCCA‑‑‑‑‑‑‑‑‑CTTTTTGTAATAGTTCCA

>Lal_accB2b_LAGI01_37973

‑‑‑ATGGCTTCTTTT‑‑‑TCTGTTCCATGCCCAAAATGCCCTTCTCCTCCTATTTCTTCTTCTTCTTCTTCC‑‑‑‑‑‑‑‑‑CTTTTGGGGTTGAAATCT‑‑‑AACAAG‑‑‑‑‑‑‑‑‑‑‑‑‑‑‑‑‑‑‑‑‑CTTCCTTTCCAGAAAGGATTGTTGTGTTTGAAGCCATCT‑‑‑CAATCA‑‑‑‑‑‑‑‑‑‑‑‑‑‑‑‑‑‑‑‑‑‑‑‑‑‑‑‑‑‑‑‑‑TTTGGGATTCGGTGCCTT‑‑‑AACAGAAACCAATTTTCTGTCAGAAAGCTTCAAGCAAAACTCAATGAGGCTGATACCGTTGAAAAT‑‑‑‑‑‑TCAAATTCTGCACCTGTACTC‑‑‑AACGGACCTAGTGTCGTCACTTCATCAAAAGAGGAAGAAGATAATAACGGGGCACTATCTAAT‑‑‑GGCACTGCTGCTGCAGATGAATCTTCAATTTCTGCTTTCATGGCTCAAGTTGCAGACCTTGTAAAACTTGTAGATTCCAGAGATATCGTGGAACTGGAACTAAAGCAATCAAACTGCGAGGTCTTGATAAGAAAAAAGGAAGCTTTGCAGCCT‑‑‑‑‑‑CCACCA‑‑‑‑‑‑‑‑‑‑‑‑GCCCCAGCAGCA‑‑‑CCA‑‑‑‑‑‑CAATATCCGTATCCAACTTAT‑‑‑CAT‑‑‑‑‑‑‑‑‑GCTCCG‑‑‑CCACCAGCACCA‑‑‑‑‑‑‑‑‑CCAGCACCACCTGCTGCA‑‑‑‑‑‑‑‑‑GCTCCTGCC‑‑‑AGCTCTCCACCTTCTAAGGCAGCACCTGCCCTACCT‑‑‑‑‑‑TCCCCAGGAAAGGCAATT‑‑‑GCGTCATCTCACCCACCACTGAAATGTCCAATGGCAGGGACCTTTTATCGGAGCCCAGCACCTGGTGAACCGGCATTTGTCAAAGTGGGAGACAAAGTGAAGAAAGGCCAGGTTATTTGCATTATCGAGGCTATGAAACTGATGAATGAAATCGAGGCTGATCAATCAGGAACAATAGCTGAGGTAATCGCTGAGGATGGGAAACCAGTCAGTGTAGACACGCCA‑‑‑‑‑‑‑‑‑CTTTTTGTAATAGTTCCA

>Lal_accB2c_LAGI01_40148

‑‑‑ATGGCTTCTTTT‑‑‑TCTGTTCCATGCCCAAAATGCCCTTCTCCTCCTATTTCTTCTTCTTCTTCTTCC‑‑‑‑‑‑‑‑‑CTTTTGGGGTTGAAATCT‑‑‑AACAAG‑‑‑‑‑‑‑‑‑‑‑‑‑‑‑‑‑‑‑‑‑CTTCCTTTCCAGAAAGGATTGTTGTGTTTGAAGCCATCT‑‑‑CAATCA‑‑‑‑‑‑‑‑‑‑‑‑‑‑‑‑‑‑‑‑‑‑‑‑‑‑‑‑‑‑‑‑‑TTTGGGATTCGGTGCCTT‑‑‑AACAGAAACCAATTTTCTGTCAGAAAGCTTCAAGCAAAACTCAATGAGGCTGATACCGTTGAAAAT‑‑‑‑‑‑TCAAATTCTGCACCTGTACTC‑‑‑AATGGACCTACTGTTGCT‑‑‑TCATCAAAAGAT‑‑‑GACGAACATAATGGGAAACTCTCCGAT‑‑‑GGTACT‑‑‑GCTGCAAATGAATCGTCAATTTCGGCCTTCATGGCTCAAGTTGCAGACCTTGTCAAACTCGTAGATTCCAGAGATATTGTGGAATTGCAACTTAAGCAATCAGGCTGCGAGCTCGTAATAAGAAAAAAAGAAGCTTTGCAGCCT‑‑‑‑‑‑CCACCAGCCTCAATTATAGCACCGGCATCA‑‑‑CCA‑‑‑‑‑‑CCATATCCTTATCCAACATAT‑‑‑GAT‑‑‑‑‑‑‑‑‑GCTCCA‑‑‑CCAGCACCAGTA‑‑‑‑‑‑‑‑‑CCAGCACCAGCTGCTTCA‑‑‑‑‑‑‑‑‑GCTCCTGCT‑‑‑AGCCCCCCACCTTCAAAAGCAGCACCCGCCTTACCT‑‑‑‑‑‑TTCCCGGGGAAAGCAAGC‑‑‑ACATCATCTCACCCACCACTGAAATGTCCGATGGCAGGAACCTTTTATCGGAGTCCAGCACCCGGTGAACCTCCATTTGTCAAAGTGGGCGACAAAGTTCAGAAAGGCCAGGTTATTTGCATTATCGAGGCTATGAAACTGATGAATGAAATCGAGGCTGATCAATCAGGAACAATAGCCGAGATAATAGCCGAAGACGGGAAACCAGTCAGCGTAGACACGCCT‑‑‑‑‑‑‑‑‑CTTTTTGTAATAGTTCCA

>Lal_accB2d_LAGI01_40382

‑‑‑ATGTCTTCTTTT‑‑‑TCTGTCCCATGCCCAAAATGCCCT‑‑‑‑‑‑‑‑‑‑‑‑‑‑‑CCTCATTCTTCTTCA‑‑‑‑‑‑‑‑‑CTTTTGGGGTTGAAAGCT‑‑‑ACCAAG‑‑‑‑‑‑‑‑‑‑‑‑‑‑‑‑‑‑‑‑‑TTATCTTTTCAAAGCGGGTTCATGTGTTTGAAGCAGTTT‑‑‑CAATCATTTGGATCTTTGTCTGCTGAGTCAGCAACA‑‑‑TTTGGGATTCGGTGCCTT‑‑‑AACAGAAAGCAAATTTCTGGTGGGAAGCTTCAAGCAAAACTCAATGAGGCTGCTACCGTTGAAAAT‑‑‑‑‑‑TCAAATTCTGCACCTGTACTC‑‑‑AATGGACCTACTGTTGCT‑‑‑TCATCAAAAGAT‑‑‑GACGAACATAATGGGAAACTCTCCGAT‑‑‑GGTACT‑‑‑GCTGCAAATGAATCGTCAATTTCGGCCTTCATGGCTCAAGTTGCAGACCTTGTCAAACTCGTAGATTCCAGAGATATTGTGGAATTGCAACTTAAGCAATCAGGCTGCGAGCTCGTAATAAGAAAAAAAGAAGCTTTGCAGCCT‑‑‑‑‑‑CCACCAGCCTCAATTATAGCACCGGCATCA‑‑‑CCA‑‑‑‑‑‑CCATATCCTTATCCAACATAT‑‑‑GAT‑‑‑‑‑‑‑‑‑GCTCCA‑‑‑CCAGCACCAGTA‑‑‑‑‑‑‑‑‑CCAGCACCAGCTGCTTCA‑‑‑‑‑‑‑‑‑GCTCCTGCT‑‑‑AGCCCCCCACCTTCAAAAGCAGCACCCGCCTTACCT‑‑‑‑‑‑TTCCCGGGGAAAGCAAGC‑‑‑ACATCATCTCACCCACCACTGAAATGTCCGATGGCAGGAACCTTTTATCGGAGTCCAGCACCCGGTGAACCTCCATTTGTCAAAGTGGGCGACAAAGTTCAGAAAGGCCAGGTTATTTGCATTATCGAGGCTATGAAACTGATGAATGAAATCGAGGCTGATCAATCAGGAACAATAGCTGAGGTAATCGCTGAGGATGGGAAACCAGTCAGTGTAGACACGCCA‑‑‑‑‑‑‑‑‑CTTTTTGTAATAGTTCCA

>Lal_accB2e_LAGI01_44867

‑‑‑ATGTCTTCTTTT‑‑‑TCTGTCCCATGCCCAAAATGCCCT‑‑‑‑‑‑‑‑‑‑‑‑‑‑‑CCTCATTCTTCTTCA‑‑‑‑‑‑‑‑‑CTTTTGGGGTTGAAAGCT‑‑‑ACCAAG‑‑‑‑‑‑‑‑‑‑‑‑‑‑‑‑‑‑‑‑‑TTATCTTTTCAAAGCGGGTTCATGTGTTTGAAGCAGTTT‑‑‑CAATCATTTGGATCTTTGTCTGCTGAGTCAGCAACA‑‑‑TTTGGGATTCGGTGCCTT‑‑‑AACAGAAAGCAAATTTCTGGTGGGAAGCTTCAAGCAAAACTCAATGAGGCTGCTACCGTTGAAAAT‑‑‑‑‑‑TCAAATTCTGCACCTGTACTC‑‑‑AACGGACCTAGTGTCGTCACTTCATCAAAAGAGGAAGAAGATAATAACGGGGCACTATCTAAT‑‑‑GGCACTGCTGCTGCAGATGAATCTTCAATTTCTGCTTTCATGGCTCAAGTTGCAGACCTTGTAAAACTTGTAGATTCCAGAGATATCGTGGAACTGGAACTAAAGCAATCAAACTGCGAGGTCTTGATAAGAAAAAAGGAAGCTTTGCAGCCT‑‑‑‑‑‑CCACCA‑‑‑‑‑‑‑‑‑‑‑‑GCCCCAGCAGCA‑‑‑CCA‑‑‑‑‑‑CAATATCCGTATCCAACTTAT‑‑‑CAT‑‑‑‑‑‑‑‑‑GCTCCG‑‑‑CCACCAGCACCA‑‑‑‑‑‑‑‑‑CCAGCACCACCTGCTGCA‑‑‑‑‑‑‑‑‑GCTCCTGCC‑‑‑AGCTCTCCACCTTCTAAGGCAGCACCTGCCCTACCT‑‑‑‑‑‑TCCCCAGGAAAGGCAATT‑‑‑GCGTCATCTCACCCACCACTGAAATGTCCAATGGCAGGGACCTTTTATCGGAGCCCAGCACCTGGTGAACCGGCATTTGTCAAAGTGGGAGACAAAGTGAAGAAAGGCCAGGTTATTTGCATTATCGAGGCTATGAAACTGATGAATGAAATCGAGGCTGATCAATCAGGAACAATAGCCGAGATAATAGCCGAAGACGGGAAACCAGTCAGCGTAGACACGCCT‑‑‑‑‑‑‑‑‑CTTTTTGTAATAGTTCCA

>Lan_accB2a_Lup032465

‑‑‑ATGGCTTCTTTC‑‑‑TCTGTTCCATGTCCAAAATGCCCTTGTCCT‑‑‑‑‑‑‑‑‑‑‑‑‑‑‑TCTTCTTCC‑‑‑‑‑‑‑‑‑CTTTTGGGGTTGAAATCT‑‑‑AACAAG‑‑‑‑‑‑‑‑‑‑‑‑‑‑‑‑‑‑‑‑‑GTACCTTTTCAGAATGGGTTGTTGTGTTTGAAGACATCT‑‑‑CAATCATTTGGATCTTTGTCTTCTGAGTCAGCAACA‑‑‑TTTGGGATTCGGTGCCTT‑‑‑AACAGAAAGCAATTTTCCGTCGGAAAGCTTCAAGCAAAACTCAATGAGGCTGATACTGTTGAAAAT‑‑‑‑‑‑TCAAAATCTGCACCTGTACTC‑‑‑AATGGACCTACTGTCCCT‑‑‑TCATCAAACGAG‑‑‑GAAGAACATAATGGGAAACTCGCTGAT‑‑‑GGTACT‑‑‑GCTGCAAATGAATCTTCGATTTCTGCCTTCTTGACTCAAGTTGCAGACCTTGTCAAACTTGTAGATTCCCGAGATATTGTGGAATTGCAACTTAAGCAATCAGGTTGCGAGCTCGTGATAAGAAAACAAGAAGCTTTGCAGCCT‑‑‑‑‑‑CCACCAGCCTCGACTATAGCACCAGCATCA‑‑‑CCG‑‑‑‑‑‑CCGTATCCTTATCCAACATAT‑‑‑CAA‑‑‑‑‑‑‑‑‑GCTCCA‑‑‑CCAGCA‑‑‑‑‑‑‑‑‑‑‑‑‑‑‑CCAGCACCACCTGCTCCA‑‑‑‑‑‑‑‑‑GCTCCTGCC‑‑‑AGCCCACCACCTTCAAAAGCAGTACCCGCCTTACCT‑‑‑‑‑‑TCTCCGGGGAAAGCAAGC‑‑‑ACATCATCTCACCCACCACTGAAATGTCCAATGGCAGGAACCTTTTATCGGAGTCCAGCACCCAGTGAACCTCCATTTGTCAAAGTGGGCGACAAAGTTCAGAAAGGCCAGGTTATTTGCATTATCGAGGCTATGAAGCTGATGAATGAAATCGAGGCTGATCAATCGGGAACAATAGCCGAGATAATAGCGGAGGATGGGAAACCAGTCAGCGTAGACACGCCT‑‑‑‑‑‑‑‑‑CTTTTTGTAATAGTTCCA

>Lan_accB2b_NLL05_2

‑‑‑‑‑‑‑‑‑‑‑‑‑‑‑‑‑‑‑‑‑‑‑‑‑‑‑‑‑‑‑‑‑‑‑‑‑‑‑‑‑‑‑‑‑‑‑‑‑‑‑‑‑‑‑‑‑‑‑‑‑‑‑‑‑‑‑‑‑‑‑‑‑‑‑‑‑‑‑‑‑‑‑‑‑‑‑‑‑‑‑‑‑‑‑‑‑‑‑‑‑‑‑‑‑‑‑‑‑‑‑‑‑‑‑‑‑‑‑‑‑‑‑‑‑‑‑‑‑‑‑‑‑‑‑‑‑‑‑‑‑‑‑‑‑‑‑‑‑‑‑‑‑‑‑‑‑‑‑‑‑‑‑‑‑‑‑‑‑‑‑‑‑‑‑‑‑‑‑‑‑‑‑‑‑‑‑‑‑‑‑‑‑‑‑‑‑‑‑‑‑‑‑‑‑‑‑‑‑‑‑‑‑‑‑‑‑‑‑‑‑‑TGCCTT‑‑‑AACAGAAAGCAATTTTCCGTCGGAAAGCTTCAAGCAAAACTCAATGAGGCTGATACTGTTGAAAAT‑‑‑‑‑‑TCAAAATCTGCACCTGTACTC‑‑‑AATGGACCTACTGTCCCT‑‑‑TCATCAAACGAG‑‑‑GAAGAACATAATGGGAAACTCGCTGAT‑‑‑GGTACT‑‑‑GCTGCAAATGAATCTTCGATTTCTGCCTTCTTGACTCAAGTTGCAGACCTTGTCAAACTTGTAGATTCCCGAGATATTGTGGAATTGCAACTTAAGCAATCAGGTTGCGAGCTCGTGATAAGAAAACAAGAAGCTTTGCAGCCT‑‑‑‑‑‑CCACCAGCCTCGACTATAGCACCAGCATCA‑‑‑CCG‑‑‑‑‑‑CCGTATCCTTATCCAACATAT‑‑‑CAA‑‑‑‑‑‑‑‑‑GCTCCA‑‑‑CCAGCA‑‑‑‑‑‑‑‑‑‑‑‑‑‑‑CCAGCACCACCTGCTCCA‑‑‑‑‑‑‑‑‑GCTCCTGCC‑‑‑AGCCCACCACCTTCAAAAGCAGTACCCGCCTTACCT‑‑‑‑‑‑TCTCCGGGGAAAGCAAGC‑‑‑ACATCATCTCACCCACCACTGAAATGTCCAATGGCAGGAACCTTTTATCGGAGTCCAGCACCCAGTGAACCTCCATTTGTCAAAGTGGGCGACAAAGTTCAGAAAGGCCAGGTTATTTGCATTATCGAGGCTATGAAGCTGATGAATGAAATCGAGGCTGATCAATCGGGAACAATAGCCGAGATAATAGCGGAGGATGGGAAACCAGTCAGCGTAGACACGCCT‑‑‑‑‑‑‑‑‑CTTTTTGTAATAGTTCCA

>Lan_accB2c_Lup029955

‑‑‑ATGGCTTCTTTT‑‑‑TCTGTTCCATGCCCAAAATGCCCTCCTCCTCCT‑‑‑‑‑‑CCTCCTCCTTCTTCC‑‑‑‑‑‑‑‑‑CTTTTGGGATTGAAAGCT‑‑‑AGCAAG‑‑‑‑‑‑‑‑‑‑‑‑‑‑‑‑‑‑‑‑‑CTCTCTTTTCAGAGTGGGTTGTTGTGTTTGAAGCAGTTT‑‑‑CAATCATTTGGATCTATGTCTTCTGAGTCAGCAACA‑‑‑TTTGGGATTCGGTGCCTT‑‑‑AACCGAAAACAAATTTCTGGTGGGAAGCTTCAAGCAAAACTCAATGAGGCTGCTACTGTTGAAAAT‑‑‑‑‑‑TCAAATTCTGCACCTGTACTC‑‑‑AATGGACCTAGCGTCGTCACTTCATCAAAAGAGGAAGAAAATCATAACGGGGCACTATCTAAT‑‑‑GGCACT‑‑‑GCTGCAGATGAATCTTCAATTTCTGCTTTCTTGTCTCAAGTTGCAGACCTTGTAAAACTTGTAGATTCCAGAGATATTGTGGAACTGCAACTAAAGCAATCAGGCTGCGAGGTCATGATAAGAAAAACGGAAGCTTTGCAGCCT‑‑‑‑‑‑CCACCA‑‑‑‑‑‑‑‑‑‑‑‑GCCCCAGCATCA‑‑‑CCA‑‑‑‑‑‑CAATATCCGTATCCAACTTAT‑‑‑CAG‑‑‑‑‑‑‑‑‑GCTCCA‑‑‑CCACCACCACCACCAGCAGCACCAGCACCACCTGCTGCA‑‑‑‑‑‑‑‑‑GCTCCTGCC‑‑‑AACTCTCCACCTTCAAAAGCAGCACTTGCCTTACCT‑‑‑‑‑‑TCCCCAGGAAAGGCAATC‑‑‑GCATCATCTCATCCACCGCTGAAAAGTCCAATGGCAGGGACCTTGTATCGGAGCCCAGCACCTGGTGAACCTGCATTTGTGAAAGTGGGAGACAAAGTGCAGAAAGGCCAGGTTATTTGCATTATTGAGGCTATGAAACTGATGAATGAAATCGAGGCTGATCAATCAGGAACAATAGCTGAGGTATTAGTTGAGGATGGGAAACCAGTCAGCGTAGACACGCCA‑‑‑‑‑‑‑‑‑CTTTTTGTAATAGTTCCA

>Lja_accB2a_Lj1g3v3008790

ATGGCGTCGTCTTTC‑‑‑ACTGTCCCTTGCCCCAAATGCCTCTCTTTT‑‑‑‑‑‑‑‑‑‑‑‑‑‑‑‑‑‑‑‑‑ACC‑‑‑‑‑‑‑‑‑GGTTCTGGGTTGAACTCT‑‑‑CGGACCCCTCCGCCGCAGAAC‑‑‑‑‑‑ATGCAGATTCGGAGCGGG‑‑‑TTGGGGTTGAAGGAATCC‑‑‑GGGTCGTTTGGATCTTTGAGCCATGATTCAGCAGCACCAAATGGGATTCAGGCCCTT‑‑‑AACAGAAAACAATATTCTGTCTGGAAGTTTCAAGCATTACCTAGTGAGGCTGCTACAGTTGGAAATTCT‑‑‑TCAAATTCTGCTCCTGTACTGGTCAAAGAACCTAAAGTCGCT‑‑‑TCACTAGAAGAGAAAGAT‑‑‑‑‑‑AATGGGAAACCCTCTGGTCCAAGCACT‑‑‑TCTACAGATGCATCTTCAATATCTGCTTTTATGAATCAAGTTTCAGACCTTGTTAAACTTGTGGATTCTAAAGATATCGTGGAGCTGCAACTTAAGCAAGCGGACTATGAGCTGATGATAAGAAAAAAGGAAGCTTTGCAGCCT‑‑‑‑‑‑CCACCAGCTACTTTTGTAGCTCCAGCATCA‑‑‑CAG‑‑‑‑‑‑CCATATCCCTATCCCGCACAT‑‑‑GCTTCTCCGCAAGCACCA‑‑‑CCACCACCA‑‑‑‑‑‑‑‑‑‑‑‑CCACCTGCTGTTGCTTCT‑‑‑‑‑‑‑‑‑‑‑‑‑‑‑‑‑‑‑‑‑‑‑‑‑‑‑GCTCCTACAAAAGCAGCTCCTGCCCTTCCA‑‑‑‑‑‑‑‑‑CCTGGAAAATCAAGC‑‑‑TCATCATCTCTTCCACAGTTGAAATGTCCAATGGCGGGAACCTTTTATCGGAGCCCAGCACCTGGTGAACCACCATTTGTCAAGGTGGGAGATAAAGTGCAGAAAGGGCAGGTTATTTGCATTATTGAGGCCATGAAACTGATGAACGAAATTGAAGCTGATAAATCAGGAACAATAGCTGAGGTACTGGCGGAGGATGGGAAACCAGTCAGTGTCGACTCGCCT‑‑‑‑‑‑‑‑‑CTTTTTGCAATTGCACCA

>Lja_accB2b_Lj2g3v1079560

‑‑‑ATGGCCTCTTTC‑‑‑TCGGTTCCATGCTCAAAATCTCCTGCCCCT‑‑‑‑‑‑‑‑‑‑‑‑‑‑‑‑‑‑‑‑‑GCA‑‑‑‑‑‑‑‑‑CCTTTGGGATTGAGATCATCCCACAAG‑‑‑‑‑‑‑‑‑‑‑‑‑‑‑‑‑‑‑‑‑GTTGAGTTTCAGAATGTG‑‑‑TTGTGCTTGAAGCCTTCCCAACAATCATTTGGATCTTTGTGTGCTCGGCCAGCTTCA‑‑‑TCTGAGATTGGGTGTCTT‑‑‑AACAGGAAGCAACTATCTGTCCAGAAGTTGCAAGCACAACGTAATGAGGCAGCTACTGTTGGAAAT‑‑‑‑‑‑TCTAATTCTGCACCGTTACCTGTCAAT‑‑‑‑‑‑‑‑‑GTTGCT‑‑‑TCTTCAAAAGAGAAAGAAGACTATAATGGGAGTGTCTCTTGT‑‑‑GGCACT‑‑‑GTCGCAGACGCATCTTCAATTTCTACATTCATGGCCCAGGTTTCAGACCTTGTAAAACTTGTGGATTCTAGAGATATTGTAGAACTGCAACTTAAGCAATCAGACTGTGAGCTCATGATAAGAAAAAGAGAAGCTTTGCACCCT‑‑‑‑‑‑CCACCAGCCCCAGCAGTGGCTCCAGCATCA‑‑‑CCA‑‑‑‑‑‑TCATTTTACTATCCCCCACTT‑‑‑CCT‑‑‑‑‑‑‑‑‑TCGTCG‑‑‑CCATCCCCG‑‑‑‑‑‑‑‑‑‑‑‑CCACCAGTGGCTGCTTCT‑‑‑‑‑‑‑‑‑GCTCCTGTG‑‑‑AGCTCTCCACCTTCAAAAGCAGCACCTGCCTTACCT‑‑‑‑‑‑TCCCCTGGAAAAGAAAGC‑‑‑AAATCATCTCACCCTCCACTGAAATGTCCAATGGCAGGAACCTTTTATAGGAGTCCAGCACCTGGTGAACCTCCCTTTGTCAAGGTTGGAGATAAAGTGCAGAAAGGACAGGTTATTTGCATTATTGAGGCCATGAAACTGATGAATGAAATTGAGGCTGATCAATCAGGAACAATAACTGAGATAGTGGCTGAGGATGGAAAACCAGTCAGTGTAGACGCGCCT‑‑‑‑‑‑‑‑‑CTTTTTGTAATAGCTCCA

>Mtr_accB2a_Medtr6g015020

‑‑‑ATGGCATCATTC‑‑‑TCAGTTCCATGCCCCAAATGTCCTGTTCCT‑‑‑‑‑‑‑‑‑‑‑‑‑‑‑‑‑‑TCTTCC‑‑‑‑‑‑‑‑‑CTTTTGGGTTTGAAATCT‑‑‑AACAAG‑‑‑‑‑‑‑‑‑‑‑‑‑‑‑‑‑‑‑‑‑ATCTTGTTTCAAAGTGGG‑‑‑TTGAGTTTGAAGAATTCT‑‑‑CAATCATTTGGATCTTTGTCTGCTGAGTCAGCTTCA‑‑‑TTTGGAATTCAGTGTCTT‑‑‑AACAAGAAACAATTTCCTGTC‑‑‑AAGATTCAAGCACAACTTAACGAGGCTGCTGTTGTCGAAAAT‑‑‑‑‑‑TTGAACTCTGCA‑‑‑‑‑‑‑‑‑‑‑‑‑‑‑‑‑‑CCTACTGTTGCT‑‑‑TCATCAGAAGAGAAAGAAAACCAAAATGGGAGTGTCCCTGCT‑‑‑AGCACT‑‑‑GTGTCAGATGAATCTGCAATTTCTGCATTCATGTCTCAAGTTGCTGACCTTGTAATACTTGTGGATTCAAGAGATATTGTGGAGCTGCAACTAAAGCAATCTGATTATGAACTCGTGATAAGAAAAAAAGAAGCTTTGCAGCCT‑‑‑‑‑‑CCACCAGCCACGGCTATGCCGCAATCAGCA‑‑‑CCA‑‑‑‑‑‑CCATTGTACTATCCGACACTT‑‑‑CCT‑‑‑‑‑‑‑‑‑TTGCCG‑‑‑CCACCCCCG‑‑‑‑‑‑‑‑‑‑‑‑CCACCGACAGCTTATTCT‑‑‑‑‑‑‑‑‑GCTACTGCG‑‑‑AGCTCTCCACCTTCGAAAGCAACACCTGCCTTACCT‑‑‑‑‑‑CCCCCC‑‑‑AAAACAAGT‑‑‑GCATCATCTCACCCACCGCTGAAATGTCCGATGGCAGGAACCTTTTACAGGTGCCCTGGACCTGGCGAACCTCCATTTGTCCAGGTGGGAGATACAGTGCAAAAAGGCCAAGTTATTTGCATTATTGAGGCTATGAAACTGATGAATGAAATTGAGGCTGATCAATCAGGAACAATAGCTGAGATACTAGTTGAGGATGGAAAACCAGTTAGTGTAGACTTGCCT‑‑‑‑‑‑‑‑‑CTTTTCGTAATAGTTCCA

>Mtr_accB2b_Medtr7g013100

‑‑‑ATGGCTTCTTTCACCACTGTTCCTTGTCCTAAATGTTTAACTTTT‑‑‑‑‑‑‑‑‑‑‑‑‑‑‑‑‑‑‑‑‑CAT‑‑‑‑‑‑‑‑‑CATTTGGGTTTGAAGTCT‑‑‑CAGACTTCTTCAAGGAATGTGCAG‑‑‑‑‑‑‑‑‑‑‑‑‑‑‑‑‑‑‑‑‑‑‑‑TTGGGGTTTATGAAATCT‑‑‑CAATCTTTTGGATCTTTGAGTTGTGATTTGGTGTCA‑‑‑AATGGGATTCAGTGTCTC‑‑‑GAAAGGAAGCAATTTTCGGTCTGGAGGTCTCAAGCACTTCCTAACGAGGTTGCAACTATTGAAAATTCA‑‑‑TCAAATTCTGTACCTGTATTGATCAATGAACCTAATGGTGCT‑‑‑TTACCAAAAGAGAAAGATAACCATAACGGGAAACCCCCTGGTCCAAGCGCT‑‑‑TCTACAGATGCATCTTCAGTTTCCACATTTATGGATCAAGTTTCAGAACTTGTTAAACTTGTGGATTCTAGAGATATCATGGAGCTGGAACTTAAGCAAGCAGGCTATGAGCTCATGATAAGGAAAAAGGAAGCTTTGCAGCCC‑‑‑‑‑‑CCACCAGTATCA‑‑‑‑‑‑‑‑‑‑‑‑‑‑‑CAA‑‑‑CAA‑‑‑‑‑‑CCATTTCCTTATCCTGCATATCCTCCTTCTTATCAAGCACCGCTGCCACCACCA‑‑‑‑‑‑‑‑‑‑‑‑CCACCCGTTGTTGCTTCT‑‑‑‑‑‑‑‑‑ACTCCCCCA‑‑‑AGTGCTCCTCCTTCAAATGTAGTTCCAGCCTTGCCT‑‑‑‑‑‑‑‑‑CCTGCAAAAACAAAT‑‑‑GCATCATCTCATCCACAGCTTAAATGTCCGATGGCAGGAACTTTCTATCGATGTCCTGGACCTGGCGAACCACCATTTGTCAAGGTGGGAGATCAAGTGCAGAAAGGGCAGGTTGTTTGCATTATTGAGGCTATGAAACTGATGAATGAAATTGAAGCTGATCGATCAGGAACAATAGTTGAGGTACTGGTAGAAGATGGGAAACCAGTCAGTGTTGGCATGCCT‑‑‑‑‑‑‑‑‑CTTTTTGCAATTGCACCA

>Pvu_accB2a_Phvul.004G034300

‑‑‑ATGGCCTCATTC‑‑‑TCGGTCCCCTGCCCCAAGTGCCCTTCTACT‑‑‑‑‑‑‑‑‑‑‑‑‑‑‑TCTTCACCC‑‑‑‑‑‑‑‑‑TCTTTTGGGTTCAAATCC‑‑‑CAACAG‑‑‑‑‑‑‑‑‑‑‑‑‑‑‑‑‑‑‑‑‑CTTCCATTTCAAACTGGG‑‑‑TTCAGCTTCAAGCCTTTT‑‑‑CCATCATTCAGATCTTTGTTTGCTGAATCTGCATCA‑‑‑TCTAGGATTCAGTGTCCT‑‑‑GACAGGAAGCAATTTCCTGTTCTGAAG‑‑‑‑‑‑‑‑‑‑‑‑‑‑‑‑‑‑‑‑‑GCTGCTAAAGTTGAAAATTCTAATTCTAATTCTGCCCCTGCAACGGTCAAAGGACCTAATGTTGCT‑‑‑TCATCAAAAGAAAAAGAAGTGCTTAATGGAAAACACTCTGGG‑‑‑AGCACT‑‑‑ACTGCAGATCAAGCTTCAGTTACAGCATTCTTATCTCAAGTTTCAAATCTTATAAAACTTGTTGATTCAAAAGATATTGTGGAACTTCAACTTGAGCAATCAGATTGTGAGCTCTTGATAAGAAAGAAAGAAGCACTGCAGCCT‑‑‑‑‑‑CCACCA‑‑‑‑‑‑‑‑‑‑‑‑GCCCCAGCATCACCACCT‑‑‑‑‑‑CCAATGCACTATGCCACATTC‑‑‑CCA‑‑‑‑‑‑‑‑‑CCAGCA‑‑‑‑‑‑‑‑‑‑‑‑‑‑‑‑‑‑‑‑‑‑‑‑GCAGCAGCACCAGCAGCT‑‑‑‑‑‑‑‑‑GCTCCTGCT‑‑‑AGCTCCTCGCCTCCAAAAGCACTGCCTGCCTTGCCT‑‑‑‑‑‑TCCCCTGGAAAAGCAAGC‑‑‑TCATCTTCTCACCCACCACAGAAAAGTCCGATTGCAGGAACTTTCTACAGGAGTCCAGGACCTGGTGAACCTCCATTTGTCAAGGTGGGCGATAAAGTGAAGAAAGGTCAGGTTATTTGCATTATTGAGGCTATGAAAATGATGAATGAAATTGAGGCTGATCAGTCAGGAACAATAACTGAGATATTGATTGAGGATGCCAAACCAATCAGTTTAGACACGCCT‑‑‑‑‑‑‑‑‑CTTTTTGTAATAGTTCCA

>Pvu_accB2b_Phvul.004G035000

‑‑‑ATGGCCTCATTC‑‑‑TCCGTCCCATGCCCCAAATGCCCTTCTATT‑‑‑‑‑‑‑‑‑‑‑‑‑‑‑TCTTCTCCC‑‑‑‑‑‑‑‑‑TCTCTGGGGTTGAAATCC‑‑‑CCACAG‑‑‑‑‑‑‑‑‑‑‑‑‑‑‑‑‑‑‑‑‑GCTCCCTTTCAAAGTGGG‑‑‑TTGAGCTTCAAGCCTTTT‑‑‑CTATCATTCAGTTCTCTGTCTGCTGAATCTGCATCA‑‑‑TCCAGAATTCAGTGTCCTAACAACAGAAAGCAATTTTCTGTTTTGAAG‑‑‑‑‑‑‑‑‑‑‑‑‑‑‑‑‑‑‑‑‑GCTGCTAAAGTTGAAAATTCGAATTCTAATTCTGCCCCCGTAACGGTCAAGGAGCCTAATGTTGCT‑‑‑TCATCAAAAGAAAAAGAAGTGCTTGATGGAAAACAATCTGGT‑‑‑GGCACT‑‑‑ATTGCAGATGAAGCTTCAGTTACTGCATTCATTTCTCAAGTTGTAGACCTTGTAAAAGTTGTTGATTCAAGAGATGTTGTGGAACTTCAACTTAAGCAATTAGACTGTGAGATTGTGATAAGAAAAAAGGAAGCACTGCAGCCT‑‑‑‑‑‑CCACCA‑‑‑‑‑‑GTTATGGCCCCAGCATCACCACCA‑‑‑‑‑‑CCTATGCACTATGCAGCATTT‑‑‑GCA‑‑‑‑‑‑‑‑‑CCGCCG‑‑‑‑‑‑‑‑‑‑‑‑‑‑‑‑‑‑‑‑‑‑‑‑CCGGCAGCTCCAGCTGCT‑‑‑‑‑‑‑‑‑GCTCCTGCT‑‑‑AGCTCTTCCTCCCCAAAAGCACCGCCTGCCTTGCCT‑‑‑‑‑‑TCCCCTGCAAAAGCAAGC‑‑‑TCACCTTCTCACCCACCAATGAAGAGTCCAATTGCAGGAACTTTCTACAGGAGTCCAGGACCTGGTCAACCTGAATTTGTCAAGGTGGGTGATAAAGTGAAGAAAGGCCAGGTTCTTTGCATTCTTGAGGCTATGAAAATGATGAATGAAATTGAGGCTGAAGAGTCAGGAACAGTAACTGAGATATTGATTGAGAATGGCAAACAAGTCAGTTTAGACACGCCT‑‑‑‑‑‑‑‑‑CTTTTTGTGATAGTTCCA

>Vra_accB2a_Vradi01g07390

‑‑‑ATGGCCTCATTC‑‑‑TCGGTCCCATGCCCCAAGTGCCCTTCTACT‑‑‑‑‑‑‑‑‑‑‑‑‑‑‑TCTTTTTCC‑‑‑‑‑‑‑‑‑TCTCTGGGGTTGAAATCC‑‑‑CAACAG‑‑‑‑‑‑‑‑‑‑‑‑‑‑‑‑‑‑‑‑‑GTTCCCTTTCAAAGTGGG‑‑‑TTCAGCTTCAAGCCTTTT‑‑‑CTATCATTCAGTCCTTTGTCTGCTGAATCTGCATCA‑‑‑TCTAGAATTCAGTGTTCT‑‑‑AACAGGAAGCAATATTCTGTTCTGAAG‑‑‑‑‑‑‑‑‑‑‑‑‑‑‑‑‑‑‑‑‑GCTGCTAAAGTCGAAAATTCTAATTCTAATTCTGCCCCGGTAGCGGTCAAAGGACCTGATGTTGCT‑‑‑TCATCAAAAGAAAAAGAAGTGCCTAAGGGAAAACAGTCTGGT‑‑‑GGCACT‑‑‑ATTGCAGATGAAGCTTCAGTTACTGCATTCATGTCTCAAGTTGCAGACCTTGTAAAACTCGTTGATTCAAGAGATATTGTGGAACTTCAACTTAAGCAATCAGACTATGAGCTCATGATAAGAAAAAAGGAAGCAGTGCAGCCT‑‑‑‑‑‑CCACCA‑‑‑‑‑‑GTTGCAGCGCCAGTATCACCACCT‑‑‑‑‑‑CCTATA‑‑‑TATGCCGCATTT‑‑‑CCA‑‑‑‑‑‑‑‑‑CCACCA‑‑‑‑‑‑‑‑‑‑‑‑‑‑‑‑‑‑‑‑‑‑‑‑CCGGTAGCACCAGCTGCT‑‑‑‑‑‑‑‑‑GCTCCTACT‑‑‑ACCTCTCCCCCCCAGAAAGCACTGCCTGCATTGCCT‑‑‑‑‑‑TCCCCTGCAAAAGCAAGC‑‑‑TCATCTTCTCACCCACCACTGAAAAGTCCGATGGCAGGAACTTTCTACAGGAGTCCAGCACCTGGTGAACCTCCATTTGTCAAGGTGGGTGATAAAGTGAAGAAAGGGCAGGTTATTTGCATTATTGAGGCTATGAAACTGATGAATGAAATTGAGGCTGATCAGTCAGGAACAATAGCTGAAATATTGATTGAGGATAGCAAACCAGTCAGTTTAGAAACGCCC‑‑‑‑‑‑‑‑‑CTTTTTGTAATAGTTCCA

>Vra_accB2b_Vradi01g07460

‑‑‑ATGGCCTCATTC‑‑‑TCGGTCCCATGCCCCAAGTGCCCTTCTACT‑‑‑‑‑‑‑‑‑‑‑‑‑‑‑TCATCTCCC‑‑‑‑‑‑‑‑‑TCTCCTGGGTTCAAATCC‑‑‑CAGCTT‑‑‑‑‑‑‑‑‑‑‑‑‑‑‑‑‑‑‑‑‑GTTCCATTTCAAAGTGGG‑‑‑TTCAGCTTCAAGCCTTTT‑‑‑CTATCATTCAGATCTTTGTCTGCTGAATCTGCATCA‑‑‑TCTAGGATTCAGTGTGCT‑‑‑AACAGGAAGCAATTTTCTGTTCTGAAG‑‑‑‑‑‑‑‑‑‑‑‑‑‑‑‑‑‑‑‑‑GCTGCTAAAGTTGAAAGTTCTAATTCTAATTCTGCCCCTGTAATTGTCAAAGAACCTAATGTTGCT‑‑‑TCATCAAAAGAAAAAGATGTGCCTAATGGAAAACACTCTGGG‑‑‑GGCACT‑‑‑GTTGCAGATGAAGCTTCAGTTACTGCATTCATCTCTCAAGTTTCAGACCTTATAAAACTTGTTGATTCAAGAGATATTGTGGAACTTCAACTTAAGCAATCAGATTGTGAGCTCATGATTAGAAAGAAAGAAGCACTGCAGCCT‑‑‑‑‑‑CCACCG‑‑‑‑‑‑‑‑‑‑‑‑GCCCCAGCATCACCACCT‑‑‑‑‑‑CCAATGCACTATGCTGCATTCCCACCA‑‑‑‑‑‑‑‑‑CCGCCT‑‑‑‑‑‑‑‑‑‑‑‑‑‑‑‑‑‑‑‑‑‑‑‑CCAGCAGCACCTGCAGCT‑‑‑‑‑‑‑‑‑GCTCCTACT‑‑‑AGCTCCTCGCCTCCAAAAGCAGTGCCTGCCTTGCCT‑‑‑‑‑‑GCCCCTGGAAAAGCAAGC‑‑‑TCATCTTCTCACCCACCACTGAAAAGTCCAATGGCAGGAACTTTCTACAGGAGTCCAGGACCTGGTGAACCGCCATTTGTCAAGGTGGGCGATAAAGTGAAGAAAGGTCAGGTTATTTGCATTATTGAGGCTATGAAACTGATGAATGAAATTGAGGCTGATCAGTCAGGAACAATAGCTGAGATATTGATTGAGGATAGCAAGCCAGTCAGTTTAGAAACGCCT‑‑‑‑‑‑‑‑‑CTTTTTGTAATAGTTCCA

# Table S15. FASTA alignment of *accC* sequences used for phylogenetic inference

>Aar_accC1_ZCDJ‑2014388

ATGGAGGCTACA‑‑‑‑‑‑ATGCCA‑‑‑ATTTGCAAGTCTGTTAGCTCACCTCGTGTT‑‑‑‑‑‑‑‑‑‑‑‑‑‑‑‑‑‑‑‑‑GGTATGTTTGTGCCAAAATCTAGAGAAATC‑‑‑AAGAGT‑‑‑‑‑‑TCC‑‑‑CAATTC‑‑‑AGCTTTTTAGTG‑‑‑GGA‑‑‑AATAAAGTGAATTTTTCAAAGCAAAGGGCT‑‑‑CAAAAT‑‑‑GCTCAAGTT‑‑‑AGTCATAAACATAGGAGG‑‑‑‑‑‑CATTGTGGGGCTCTGAATGTGACATGC‑‑‑‑‑‑‑‑‑‑‑‑CAA‑‑‑GCTGATAAGATCTTGGTCGCAAACAGAGGTGAAATTGCTGTTCGTGTAATTAGAACTGCTCATGAATTGGGCATACCTTGTGTGGCTGTGTACTCAACTATAGATAAAGATGCTCTTCATGTGAAATTGGCTGATGAATCAGTTTGCATAGGTGAAGCACCAAGTAGTAAATCATACTTATTAATTCCAAATGTTCTATCTGCTGCTGTCAGCCTTAAATGCACAATGTTGCATCCTGGATATGGTTTTCTTGCTGAGAATGCATTATTTGTTCAAATGTGCATAGAACATGGGATCAACTTCATTGGGCCCAATCCTGACAGCATACGGGTTATGGGTGACAAATCAACTGCTAGAGACACAATGAAGAAAGCAGGCGTGCCAACTGTTCCAGGAAGTGATGGGCTGTTGCAGAGCACAGAGGAAGCCATTAAGCTGGCAAATGAGATTGGTTTCCCAGTGATGATCAAGGCAACAGCTGGTGGGGGAGGGCGTGGCATGCGTCTTGCTAAAGAACCTGATGAGTTTGTGAAGTTGTTACAGCAAGCTAAGAGTGAGGCAGCAGCTGCATTTGGCAATGATGGTGTTTATTTGGAAAAATACATTCAAAATCCTAGGCACATTGAGTTTCAGGTTCTGGCAGATAAACATGGTAATGTTGTTCACTTTGGAGAGCGTGATTGCAGCATCCAGAGGCGGAATCAGAAACTGTTGGAAGAAGCACCTTCTCCTGCATTAACCCCAGAGTTGCGGAAAGCAATGGGTGATGCTGCAGTTGCGGCCGCTGCATCTATAGGTTACATTGGTGTGGGAACAGTTGAATTCCTCTTGGATGAAAGAGGTTCCTTTTACTTCATGGAGATGAACACCCGTATCCAGGTGGAGCATCCTGTGACAGAAATGATTTCCTCTGTTGATTTAATAGAAGAGCAAATTCTTGTTGCTATGGGAGAAAAACTTAGA‑‑‑‑‑‑‑‑‑‑‑‑‑‑‑TACAAACAGGAAGATATTGTGCTCAGAGGTCATTCCATTGAATGCCGTATAAATGCTGAAGATGCTTTCAAGGGATTCAGACCAGGGCCAGGGAGAATAACAGCATACTTGCCATCTGGAGGTCCTTTTGTGAGAATGGACAGTCATGTTTATCCTGATTATGTG‑‑‑GTTCCTCCTAGCTATGACTCCCTGCTTGGAAAGCTAATTGTATGGGCTCCAACAAGGGAAAAAGCAATTGAGCGAATGAAGAGGGCACTAGATGACACTATCATCACAGGGGTTCCAACAACAATTGATTATCATAAGCTTATCCTTGACATTGAGGATTTTAGAAATGGCAAAGTTGATACTGCTTTTATTCCTAAGCATGAGCAGGAGTTGGCTATG‑‑‑CCGCCCCAGAAG‑‑‑AATGTACCAGCT‑‑‑CACACG‑‑‑GCC‑‑‑‑‑‑‑‑‑‑‑‑‑‑‑TTCATTGTG

>Aar_accC2_ZCDJ‑2014389

ATGGAGGCTACA‑‑‑‑‑‑ATGCCA‑‑‑ATTTGCAAGTCTGTTAGCTCACCTCGTGTT‑‑‑‑‑‑‑‑‑‑‑‑‑‑‑‑‑‑‑‑‑GGTATGTTTGTGCCAAAATCTAGAGAAATC‑‑‑AAGAGT‑‑‑‑‑‑TCC‑‑‑CAATTC‑‑‑AGCTTTTTAGTG‑‑‑GGA‑‑‑AATAAAGTGAATTTTTCAAAGCAAAGGGCT‑‑‑CAAAAT‑‑‑GCTCAAGTT‑‑‑AGTCATAAACATAGGAGG‑‑‑‑‑‑CATTGTGGGGCTCTGAATGTGACATGC‑‑‑‑‑‑‑‑‑‑‑‑CAA‑‑‑GCTGATAAGATCTTGGTCGCAAACAGAGGTGAAATTGCTGTTCGTGTAATTAGAACTGCTCATGAATTGGGCATACCTTGTGTGGCTGTGTACTCAACTATAGATAAAGATGCTCTTCATGTGAAATTGGCTGATGAATCAGTTTGCATAGGTGAAGCACCAAGTAGTAAATCATACTTATTAATTCCAAATGTTCTATCTGCTGCTGTCAGCCTTAAATGCACAATGTTGCATCCTGGATATGGTTTTCTTGCTGAGAATGCATTATTTGTTCAAATGTGCATAGAACATGGGATCAACTTCATTGGGCCCAATCCTGACAGCATACGGGTTATGGGTGACAAATCAACTGCTAGAGACACAATGAAGAAAGCAGGCGTGCCAACTGTTCCAGGAAGTGATGGGCTGTTGCAGAGCACAGAGGAAGCCATTAAGCTGGCAAATGAGATTGGTTTCCCAGTGATGATCAAGGCAACAGCTGGTGGGGGAGGGCGTGGCATGCGTCTTGCTAAAGAACCTGATGAGTTTGTGAAGTTGTTACAGCAAGCTAAGAGTGAGGCAGCAGCTGCATTTGGCAATGATGGTGTTTATTTGGAAAAATACATTCAAAATCCTAGGCACATTGAGTTTCAGGTTCTGGCAGATAAACATGGTAATGTTGTTCACTTTGGAGAGCGTGATTGCAGCATCCAGAGGCGGAATCAGAAACTGTTGGAAGAAGCACCTTCTCCTGCATTAACCCCAGAGTTGCGGAAAGCAATGGGTGATGCTGCAGTTGCGGCCGCTGCATCTATAGGTTACATTGGTGTGGGAACAGTTGAATTCCTCTTGGATGAAAGAGGTTCCTTTTACTTCATGGAGATGAACACCCGTATCCAGGTGGAGCATCCTGTGACAGAAATGATTTCCTCTGTTGATTTAATAGAAGAGCAAATTCTTGTTGCTATGGGAGAAAAACTTAGA‑‑‑‑‑‑‑‑‑‑‑‑‑‑‑TACAAACAGGAAGATATTGTGCTCAGAGGTCATTCCATTGAATGCCGTATAAATGCTGAAGATGCTTTCAAGGGATTCAGACCAGGGCCAGGGAGAATAACAGCATACTTGCCATCTGGAGGTCCTTTTGTGAGAATGGACAGTCATGTTTATCCTGATTATGTG‑‑‑GTTCCTCCTAGCTATGACTCCCTGCTTGGAAAGCTAATTGTATGGGCTCCAACAAGGGAAAAAGCAATTGAGCGAATGAAGAGGGCACTAGATGACACTATCATCACAGGGGTTCCAACAACAATTGATTATCATAAGCTTATCCTTGACATTGAGGATTTTAGAAATGGCAAAGTTGATACTGCTTTTATTCCTAAGCATGAGCAGGAGTTGGCTATG‑‑‑CTGCTTTTATTC‑‑‑CTAAGCATGAGC‑‑‑AGGAGT‑‑‑‑‑‑‑‑‑‑‑‑‑‑‑‑‑‑‑‑‑TGGCTATGG

>Adu_accC1_Aradu.ET2TE

ATGGAGAGTAGAATA‑‑‑ATGGCT‑‑‑GCGCTCAACTCCGTTACCTCTCCTCATCTG‑‑‑CCTTCTCATTCC‑‑‑CCGGGTCTGTACGCGGTGGAGAAC‑‑‑AGCATC‑‑‑AAGAGC‑‑‑‑‑‑TCC‑‑‑CAATGC‑‑‑AGCTTCTCAGCG‑‑‑GGGAGTAAGAAGGTGAGTTTTCCGAGGCAAAGGTGC‑‑‑AGTCAC‑‑‑GTGACGAAG‑‑‑ACCAGGGCGGCACGTGATGGTGGTGCTGGTGGTGCTCTCGGTGCCACGTGT‑‑‑‑‑‑‑‑‑‑‑‑CAG‑‑‑GCGGAGAAGATCCTGGTGGCGAACAGAGGCGAGATCGCGGTGCGAGTCATCAGAACCGCTCATGAGATGGGAATTCCGTGCGTGGCTGTGTACTCGACCATTGATAAGGACGCGCTTCATGTCAAGCTCGCTGATGATGCTGTTTGCATCGGTGAAGCGCCTAGCAGTCAATCGTACTTATTGATTCCAAATGTTCTATCTGCTGCTATTAGCCGAAGATGCACAATGTTGCATCCTGGGTATGGTTTCCTTGCTGAAAATGCAGTGTTTGTTGAGATGTGCAGAGAGCATGGAATCAATTTTATTGGGCCAAATCCCGACAGTATTCGGGTTATGGGTGACAAATCAACTGCCAGAGATACAATGAAGAAAGCAGGTGTTCCTACGGTTCCGGGAAGTGATGGATTGTTACAGACCACAGAGGAAGCTATCAGGCTGGCAAATGAGATTGGTTTCCCTGTAATGATCAAGGCAACAGCTGGAGGTGGAGGGCGTGGCATGCGCCTTGCTAAAGAACCTGATGAATTTGTAAAGTTATTACAGCAAGCTAAGAGTGAAGCTGCTGCTGCATTTGGTAATGATGGAGTTTATTTGGAAAAGTATGTCCAAAACCCAAGGCACATTGAGTTCCAGGTTCTTGCTGATAAATATGGTAATGTAGTTCACTTTGGAGAACGTGATTGCAGCATCCAGAGGCGTAATCAGAAACTGTTGGAAGAAGCACCTTCTCCTGCATTGACCCCAGAGTTGCGTAAGGCAATGGGTGATGCAGCAGTTGCTGCAGCTGCATCTATAGGTTACATAGGTGTTGGAACAGTTGAGTTCCTCTTGGATGAAAGAGGTTCTTTTTACTTCATGGAGATGAACACTCGTATCCAGGTTGAGCATCCGGTGACAGAAATGATTTCTTCTGTTGATTTGATAGAAGAGCAAATTCGTGTAGCTATGGGGGAAAAACTTCGA‑‑‑‑‑‑‑‑‑‑‑‑‑‑‑TACAAACAGGAGGATATTGTGCTCAGAGGACATTCTATTGAATGCCGTATCAATGCAGAGGATGCTTTCAAGGGTTTTAGACCAGGGCCAGGTAGAATAACAGCATACTTGCCATCTGGTGGTCCATTTGTTAGAATGGACAGCCATGTTTATCCTGATTATGTG‑‑‑GTTCCTCCAAGCTATGACTCCCTTCTTGGAAAGCTTATTGTTTGGGCTCCAACAAGAGAAAAGGCAATTGAACGTATGAAAAGGGCACTTGATGACACAATTATCACAGGGGTTCCTACTACAATTGATTACCATAAACTTATCCTTGACATAGAGGATTTCAAAAATGGCAAAGTTGATACTGCTTTTATTCCAAAGCATGAAGAGGAGTTGGCAATG‑‑‑CCTCCTGTGAAG‑‑‑ATGGTACTGGCC‑‑‑‑‑‑‑‑‑‑‑‑‑‑‑AAAGAATTCGCTGGTGTAAATGCA

>Adu_accC2_Aradu.ZF3WE

ATGGAGGCCACA‑‑‑‑‑‑ATGGCA‑‑‑GCCTGCAACTCTCTTAGCTCGCCTTCTGTT‑‑‑CCC‑‑‑‑‑‑ATC‑‑‑CCAGGCTTGTATGCGGGAACAAGTAGAGGAATC‑‑‑AAGAAT‑‑‑‑‑‑TCA‑‑‑CAATGC‑‑‑AGTTTCTTA‑‑‑‑‑‑GGTGCAACTAAGGTGAATTTTCCCAGCCAAACAATGTCCAGAACT‑‑‑TGTCAACTC‑‑‑AATCACAAACATAAAACA‑‑‑‑‑‑CGTTCTGGGGCACTCCATGCTACCTGC‑‑‑‑‑‑‑‑‑‑‑‑CAG‑‑‑GGTGACAAGATCCTGGTAGCTAACAGAGGCGAAATCGCAGTTCGTGTTATTCGCACTGCTCATGAATTGGGAATACCCTGTGTGGCTGTGTACTCAACCATAGACAAGGATGCACTTCATGTCAAATTGGCTGATGAATCCGTTTGCATTGGCGAAGCGCCAAGCAGCCAATCGTACTTATTGATCCCAAATGTACTGTCTGCTGCTATTAGCCGTAGATGCACAATGTTGCATCCCGGATATGGTTTTCTTGCTGAGAATGCAGTGTTTGTTGAAATGTGTAGAGAACATGGAATCAATTTTATTGGACCTAATCCTGACAGTATTCGGGTTATGGGTGACAAATCAACTGCCAGAGACACAATGAAGAATGCAGGGGTTCCTACTGTTCCAGGAAGTGATGGGCTTTTACAGAGCACTGAAGAAGCTATCAGGCTAGCAAATGAGATTGGTTTCCCTGTTATGATCAAGGCAACAGCAGGTGGCGGTGGACGTGGTATGCGACTTGCTAAAGAACCTGGAGAGTTTGTGAAGTTGTTACAGCAAGCTAAGAGTGAGGCTGCTGCCGCCTTTGGTAATGATGGAGTTTATTTGGAGAAGTACATTCAAAATCCTAGGCACATTGAGTTCCAGGTTCTTGCAGATAAATATGGTAATGTTGTTCACTTTGGAGAACGTGATTGCAGCATCCAGAGGCGTAATCAGAAACTGTTGGAAGAAGCACCATCTCCTGCTTTGACCCCAGAGCTGCGGAAGGCAATGGGAGATGCAGCAGTTGCAGCAGCTGCATCTATCGGTTACATAGGTGTTGGAACAGTTGAATTCCTCTTGGATGAAAGGGGCTCCTTTTACTTCATGGAGATGAATACTCGTATCCAGGTTGAACATCCGGTGACAGAAATGATATCTTCTGTTGATTTGATTGAAGAGCAAATTCGTGTGGCTATGGGAGCGAAGCTTCGT‑‑‑‑‑‑‑‑‑‑‑‑‑‑‑TACAAACAGGAGGATATTATTCTGAGAGGACATTCTATTGAATGCCGTATCAATGCAGAAGATGCTTTTAAGGGATTTAGACCAGGGCCAGGTAGAATTACAGCATACTTACCATCAGGAGGCCCATTTGTCAGAATGGATAGTCATGTTTATCCTGATTATGTA‑‑‑GTTCCTCCAAGCTATGACTCCCTTCTTGGAAAGTTGATTGTGTGGGCTCCAACAAGAGAAAAAGCAATTGAACGCATGAAAAGGGCACTTGATGACACCATCATCACAGGTGTGCCTACAACAATTGAATATCATAAACTCATCCTTGACATAGAGGATTTCAGAAATGGCAAGGTTGACACTGCTTTCATTCCAAAGCATGAAGAGGAGTTGACCATG‑‑‑CCACCTCAGAAG‑‑‑ATGGTACCTGCTATTAACAAG‑‑‑GCAAAAGAATTTGTTGGAGCAACTGTA

>Aip_accC1_Araip.KVI16

ATGGAGAGTAGAATA‑‑‑ATGGCT‑‑‑GCGCTCAACTCCGTTACCTCTCCTCATCTG‑‑‑CCTTCTCATTCC‑‑‑CCGGGTCTGTTCGCGGTGGAGAAC‑‑‑AGCATC‑‑‑AAGAGC‑‑‑‑‑‑TCC‑‑‑CAATGC‑‑‑AGCTTCTCAGCG‑‑‑GGCAGCAAGAAGGTGAGTTTTCCGAGGCAAAGGTGC‑‑‑AGTCAC‑‑‑GTGACGAAG‑‑‑ACTAGGGCGGCACGTGACGGTGGTGCTGGTGGTGCTCTCGGTGCCACGTGT‑‑‑‑‑‑‑‑‑‑‑‑CAG‑‑‑GCGGAGAAGATCCTGGTGGCGAACAGAGGCGAGATCGCGGTGCGAGTCATCAGAACCGCTCATGAGATGGGGATTCCGTGCGTGGCTGTGTACTCAACCATTGATAAGGACGCGCTTCATGTCAAGCTCGCTGATGATGCTGTTTGCATCGGTGAAGCGCCTAGCAGTCAATCGTACTTATTGATTCCAAATGTTCTATCTGCTGCTATTAGCCGAAGATGCACAATGTTGCATCCTGGGTATGGTTTCCTTGCTGAAAATGCAGTGTTTGTTGAGATGTGCAGAGAGCATGGAATCAATTTTATTGGGCCAAATCCTGACAGTATTCGGGTTATGGGTGATAAATCTACTGCCAGAGATACAATGAAGAAAGCAGGTGTTCCTACGGTTCCGGGAAGTGATGGATTGTTACAGACCACAGAGGAAGCTATCAGGCTGGCAAATGAGATTGGTTTCCCTGTAATGATCAAGGCAACAGCTGGAGGTGGAGGGCGTGGCATGCGCCTTGCTAAAGAACCTGATGAATTTGTAAAGTTATTACAGCAAGCTAAGAGTGAAGCTGCTGCTGCATTTGGTAATGATGGAGTTTATTTGGAAAAGTATGTCCAAAACCCAAGGCACATTGAGTTCCAGGTTCTTGCTGATAAATATGGTAATGTAGTTCACTTTGGAGAACGTGATTGCAGCATCCAGAGGCGTAATCAGAAACTGTTGGAAGAAGCACCTTCTCCTGCATTGACCCCAGAGTTGCGTAAGGCAATGGGTGATGCAGCAGTTGCTGCAGCTGCATCTATAGGTTACATAGGTGTTGGAACGGTTGAGTTCCTCTTGGATGAAAGAGGTTCTTTTTACTTCATGGAGATGAACACTCGTATCCAGGTTGAGCATCCGGTGACAGAAATGATTTCTTCTGTTGATTTGATAGAAGAGCAAATTCGTGTAGCTATGGGGGAAAAACTTCGA‑‑‑‑‑‑‑‑‑‑‑‑‑‑‑TACAAACAGGAGGATATTGTGCTCAGAGGACATTCTATTGAATGCCGTATCAATGCAGAGGATGCTTTCAAGGGTTTTAGACCAGGGCCAGGTAGAATAACAGCATACTTGCCATCTGGTGGTCCATTTGTTAGAATGGACAGCCATGTTTATCCTGATTATGTG‑‑‑GTTCCTCCAAGCTATGACTCCCTTCTTGGAAAGCTTATTGTTTGGGCTCCAACAAGAGAAAAGGCAATTGAACGTATGAAAAGGGCACTTGATGACACAATTATCACAGGGGTTCCTACTACAATTGATTACCATAAACTTATCCTTGACATAGAGGATTTCAAAAATGGCAAAGTTGATACTGCTTTTATTCCAAAGCATGAAGAGGAGTTGGCAATG‑‑‑‑‑‑‑‑‑‑‑‑‑‑‑‑‑‑‑‑‑‑‑‑‑‑‑‑‑‑‑‑‑‑‑‑‑‑‑‑‑‑‑‑‑‑‑‑‑‑‑‑‑‑‑‑‑‑‑‑GTGACATTT

>Aip_accC2_Araip.Z9LG3

ATG‑‑‑‑‑‑‑‑‑ATC‑‑‑CTGTCA‑‑‑TTTTGGCCCAAGTGTGATGGGCCCAGTTGT‑‑‑GCA‑‑‑‑‑‑AGT‑‑‑GCAGGTGCAGGTGCAGGA‑‑‑‑‑‑‑‑‑GGAATC‑‑‑AAGAAT‑‑‑‑‑‑TCA‑‑‑CAATGC‑‑‑AGTTTCTTA‑‑‑‑‑‑GGTGCAACTAAGGTGAATTTTCCCAGCCAAACAATGCCCGGAACT‑‑‑TGTCAACTC‑‑‑AATCACAAACATAAAACA‑‑‑‑‑‑CGTTCTGGAGCACTCCATGCTACCTGC‑‑‑‑‑‑‑‑‑‑‑‑CAG‑‑‑GGTGACAAGATCCTGGTAGCTAACAGAGGCGAAATCGCAGTTCGTGTTATTCGCACTGCTCATGAATTGGGAATACCCTGTGTGGCTGTGTACTCAACCATAGACAAGGATGCACTTCATGTCAAATTGGCTGATGAATCCGTTTGCATTGGTGAAGCGCCAAGCAGCCAATCGTACTTATTGATCCCAAATGTACTGTCTGCTGCTATTAGCCGTAGATGCACAATGTTGCATCCTGGATATGGTTTTCTTGCTGAGAATGCAGTGTTTGTTGAAATGTGTAGAGAACATGGAATCAATTTTATTGGACCTAATCCTGACAGTATTCGGGTTATGGGTGACAAATCAACTGCCAGAGACACAATGAAGAATGCAGGGGTTCCTACTGTTCCAGGAAGTGATGGGCTTTTACAGAGCACTGAAGAAGCTATCAGGCTAGCAAATGAGATTGGTTTCCCTGTTATGATCAAGGCAACAGCAGGTGGCGGTGGACGTGGTATGCGACTTGCTAAAGAACCTGGAGAGTTTGTGAAGTTGTTACAGCAAGCTAAGAGTGAGGCTGCTGCCGCCTTTGGTAATGATGGAGTTTATTTGGAGAAGTACATTCAAAATCCTAGGCACATTGAGTTCCAGGTTCTTGCAGATAAATATGGTAATGTTGTTCACTTTGGAGAACGTGATTGCAGCATCCAGAGGCGTAATCAGAAACTGTTGGAAGAAGCACCATCTCCTGCTTTGACCCCAGAGCTGCGGAAGGCAATGGGAGATGCAGCAGTTGCAGCAGCTGCATCTATCGGTTACATAGGTGTTGGAACAGTTGAATTCCTCTTGGATGAAAGGGGCTCCTTTTACTTCATGGAGATGAATACTCGTATCCAGGTTGAACATCCGGTGACAGAAATGATATCTTCTGTTGATTTGATTGAAGAGCAAATTCGTGTGGCTATGGGAGCGAAGCTTCGT‑‑‑‑‑‑‑‑‑‑‑‑‑‑‑TACAAACAGGAGGATATTATTCTGAGAGGACATTCTATTGAATGCCGTATCAATGCAGAAGATGCTTTTAAGGGATTTAGACCAGGGCCA‑‑‑‑‑‑‑‑‑‑‑‑‑‑‑‑‑‑‑‑‑‑‑‑‑‑‑‑‑‑‑‑‑‑‑‑‑‑‑‑‑‑‑‑‑‑‑‑‑‑‑‑‑‑‑‑‑‑‑‑‑‑‑‑‑‑‑‑‑‑‑‑‑‑‑‑‑‑GTTCCTCCAAGCTATGACTCCCTTCTTGGAAAGTTGATTGTGTGGGCTCCAACAAGAGAAAAAGCAATTGAACGCATGAAAAGGGCACTTGATGACACCATCATCACAGGTGTGCCTACAACAATTGAATATCATAAACTCATCCTTGACATAGAGGATTTCAGAAATGGCAAGGTTGACACTGCTTTCATTCCAAAGCATGAAGAGGAGTTGACCATG‑‑‑CCACCTCAGAAG‑‑‑ATGGTACCTGCTATTAACAAG‑‑‑GCAAAAGAACTTGTTGGAGCAACTGTA

>Ath_accC_AT5G35360

ATGGACGCTTCTATG‑‑‑ATTACC‑‑‑AATTCCAAATCCATTACTTCTCCACCCTCT‑‑‑‑‑‑‑‑‑‑‑‑TTAGCCTTAGGGAAGTCAGGAGGAGGAGGA‑‑‑GTTATC‑‑‑AGAAGT‑‑‑‑‑‑TCA‑‑‑CTATGT‑‑‑AACTTAATGATG‑‑‑‑‑‑CCAAGCAAAGTTAACTTCCCTAGACAAAGAACT‑‑‑CAAACT‑‑‑CTAAAGGTT‑‑‑AGCCAGAAGAAACTCAAGAGAGCTACTAGTGGTGGTCTTGGTGTTACATGC‑‑‑‑‑‑‑‑‑‑‑‑AGTGGTGGTGATAAGATTCTCGTAGCGAATCGAGGTGAAATTGCAGTTCGTGTTATCAGAACTGCTCATGAAATGGGGATTCCTTGTGTTGCTGTGTATTCTACTATAGATAAAGATGCTCTTCATGTTAAATTAGCTGATGAAGCTGTTTGTATTGGTGAAGCTCCTAGTAACCAGTCGTATTTGGTGATTCCGAATGTTCTGTCTGCGGCTATTAGCCGTGGATGTACAATGCTTCATCCTGGATATGGTTTTCTTTCGGAGAACGCTCTCTTTGTTGAAATGTGTAGAGACCATGGGATCAATTTTATTGGACCTAATCCTGATAGCATCCGTGTTATGGGAGACAAAGCGACTGCAAGAGAGACAATGAAAAATGCAGGGGTTCCCACTGTACCAGGGAGTGATGGGCTATTGCAGAGTACAGAAGAAGCTGTCAGGGTCGCCAATGAGATTGGTTTCCCTGTAATGATCAAGGCAACAGCTGGTGGTGGTGGACGTGGAATGCGTCTTGCTAAGGAACCGGGAGAGTTTGTAAAACTGTTGCAGCAAGCTAAGAGCGAGGCTGCTGCTGCTTTTGGGAATGATGGATGTTATCTGGAGAAGTTCGTACAAAACCCTAGACATATTGAGTTCCAGGTGCTGGCAGATAAATTTGGAAATGTTGTTCACTTTGGTGAGCGTGACTGCAGCATCCAGAGGCGTAACCAAAAGCTTCTGGAAGAAGCACCTTCTCCAGCATTGACCGCTGAATTGCGAAAAGCCATGGGTGATGCAGCAGTCGCAGCAGCAGCATCCATTGGGTACATTGGTGTTGGTACTGTGGAGTTTCTTTTAGATGAGAGAGGTTCCTTCTACTTCATGGAAATGAACACTAGGATCCAGGTGGAGCATCCTGTGACAGAGATGATTTACTCTGTTGATTTGATAGAGGAGCAGATTCGTGTTGCAATGGGAGAGAAACTTCGT‑‑‑‑‑‑‑‑‑‑‑‑‑‑‑TACAAACAGGAAGATATTGTGCTCAGAGGGCACTCAATTGAATGTCGTATCAATGCAGAAGATCCATTTAAAGGATTCAGACCTGGACCTGGCAGAATAACATCATACCTGCCATCTGGAGGTCCTTTCGTTAGAATGGACAGCCATGTCTATTCCGACTATGTT‑‑‑GTGCCTCCAAGCTATGATTCTCTTCTTGGGAAGCTTATTGTGTGGGCTCCAACGAGGGAAAAAGCAATTGAACGGATGAAGCGGGCGCTTAATGACACTATCATTACAGGGGTTCCAACGACTATCAATTACCACAAACTTATCCTTGATGTTGAGGATTTCAAAAATGGAAAAGTTGATACAGCTTTCATCGTCAAGCATGAAGAGGAGCTAGCTGAG‑‑‑CCTCAAGAA‑‑‑‑‑‑ATTGTGGCAGTG‑‑‑‑‑‑‑‑‑‑‑‑‑‑‑AAAGATCTGACAAACGCAACGGTT

>Bto_accC1_JETM‑2008872

ATGGAGGCTACT‑‑‑‑‑‑ATGCCT‑‑‑GTCTGCAAGTCTGTTAGCTCGCCTGCTGTA‑‑‑‑‑‑‑‑‑‑‑‑‑‑‑‑‑‑‑‑‑GGTTTATTTCTGGGGAGAACTAGAGGTATT‑‑‑GAGAGT‑‑‑‑‑‑TCT‑‑‑CAGTGC‑‑‑AGCTTTTTAGTG‑‑‑GGA‑‑‑AATAAAGTGAACTTTCCTAGGCTAAGAGCT‑‑‑CAAGAT‑‑‑GCTCAAGTA‑‑‑AGTCCTAAATATCGAAGG‑‑‑‑‑‑CATTGTGGGGCTCTTCGTGCAACATGT‑‑‑‑‑‑‑‑‑‑‑‑CAA‑‑‑GCTGATAAAATCCTGGTGGCAAATAGAGGGGAAATTGCTGTTCGTGTTATTAGAACTGCTCATGAATTGGGGATACCTTGTGTGGCTGTTTACTCAACCATAGACAAGGATGCACTTCATGTGAAATTGGCTGATGAAGCAGTATGTATAGGTGAAGCACCCAGTAATCAATCGTATTTATTAATTCCAAATGTTCTATCAGCTGCTATCAGCCGTAGATGTACAATGTTGCATCCTGGATATGGTTTTCTTGCTGAAAATGCAGTATTTGTTGATATGTGCAGATCACATAGAATCAATTTTATTGGGCCCAATCCTGATAGTATTCGGATAATGGGTGACAAATCAACTGCCAGAGACACGATGAAGAAAGCAGGTGTTCCGACTGTTCCAGGAAGTGATGGACTATTGCAGACCACGGATGAAGCAATCAAGCTGGCAAATGAGATTGGTTTCCCTGTGATGATCAAGGCAACAGCAGGTGGTGGAGGACGTGGCATGCGTCTCGCCAAAGAACCTGATGAGTTTGTGAAATTGTTACAGCAAGCTAAGAGTGAGGCAGCTGCTGCTTTCGGCAATGATGGGGTTTATTTGGAAAAGTACATTCAAAATCCCAGGCATATTGAGTTCCAGGTTCTTGCAGACAAATACGGTAATGTAGTTCACTTTGGCGAGCGTGATTGCAGCATCCAGAGGCGAAACCAAAAACTGTTGGAAGAAGCACCTTCTCCTGCGTTGACCCCAGAGTTGAGAAAGGCAATGGGTGATGCAGCAGTTGCAGCAGCTGCGTCTATAGGTTACATAGGTGTTGGAACAGTTGAATTCCTCTTGGATGAGAGAGGTTCCTTTTACTTCATGGAGATGAACACTCGCATTCAGGTGGAGCATCCTGTGACAGAAATGATTTCATCTGTTGATTTGATTGAAGAGCAAATTCGTGTAGCTTTGGGAGAAAAGCTTCGA‑‑‑‑‑‑‑‑‑‑‑‑‑‑‑TACAAACAGGAAGATATTGTTCTCAAGGGACATTCTATCGAATGCCGCATCAATGCAGAAGATGCTTTTAAGGGATTTAGACCAGGACCAGGAAGAATAACAGCATACCTGCCATCTGGAGGTCCATTTGTCAGAATGGACAGCCATGTTTATCCAGGTTATGTT‑‑‑GTACCTCCAAGCTACGACTCCCTCCTTGGAAAGCTAATTGTGTGGGCTCCAACAAGGGAAAAAGCAATTGAACGCATGAAAAGGGCACTTGACGACACCATTATCACAGGGGTACCTACAACAATTGATTATCATAAACTGATCCTTGACATAGAGGATTTCAGAAATGGAAAGGTTGACACTGCTTTTATTCCAAAGCATGAGGAGGAGTTGGCTACG‑‑‑CCTGCTCCGAAG‑‑‑CATTGGCCGATGGTAACTACG‑‑‑AACAAGAAC‑‑‑‑‑‑‑‑‑‑‑‑‑‑‑‑‑‑

>Bto_accC2_JETM‑2008874

ATGGAGGTTACA‑‑‑‑‑‑ATGCCT‑‑‑GTCTGCAAGTCTGTTAGCTCGCCTAGTGTA‑‑‑‑‑‑‑‑‑‑‑‑‑‑‑‑‑‑‑‑‑GGTTTATTTGTGGGGACAACTAGAGGAATT‑‑‑AAGAGT‑‑‑‑‑‑TCT‑‑‑CAATGC‑‑‑AGCTTTTTAGTG‑‑‑GGA‑‑‑AACAAAGTGAATTTTCCTAGGCAAAGAGCT‑‑‑CAAAGT‑‑‑GCTAAGGTA‑‑‑AGTCGTAAACATAGAACA‑‑‑‑‑‑CATTGTGGGGCTCTCCAAGCAACATGT‑‑‑‑‑‑‑‑‑‑‑‑CAA‑‑‑GCTGATAAAATCCTGGTGGCAAATAGAGGGGAAATTGCTGTTCGTGTTATTAGAACTGCTCATGAATTGGGGATACCTTGTGTGGCTGTTTACTCAACCATAGACAAGGATGCACTTCATGTGAAATTGGCTGATGAAGCAGTATGTATAGGTGAAGCACCCAGTAATCAATCGTATTTATTAATTCCAAATGTTCTATCAGCTGCTATCAGCCGTAGATGTACAATGTTGCATCCTGGATATGGTTTTCTTGCTGAAAATGCAGTATTTGTTGATATGTGCAGATCACATAGAATCAATTTTATTGGGCCCAATCCTGATAGTATTCGGATAATGGGTGACAAATCAACTGCCAGAGACACGATGAAGAAAGCAGGTGTTCCGACTGTTCCAGGAAGTGATGGACTATTGCAGACCACGGATGAAGCAATCAAGCTGGCAAATGAGATTGGTTTCCCTGTGATGATCAAGGCAACAGCAGGTGGTGGAGGACGTGGCATGCGTCTCGCCAAAGAACCTGATGAGTTTGTGAAATTGTTACAGCAAGCTAAGAGTGAGGCAGCTGCTGCTTTCGGCAATGATGGGGTTTATTTGGAAAAGTACATTCAAAATCCCAGGCATATTGAGTTCCAGGTTCTTGCAGACAAATACGGTAATGTAGTTCACTTTGGCGAGCGTGATTGCAGCATCCAGAGGCGAAACCAAAAACTGTTGGAAGAAGCACCTTCTCCTGCGTTGACCCCAGAGTTGAGAAAGGCAATGGGTGATGCAGCAGTTGCAGCAGCTGCGTCTATAGGTTACATAGGTGTTGGAACAGTTGAATTCCTCTTGGATGAGAGAGGTTCCTTTTACTTCATGGAGATGAACACTCGCATTCAGGTGGAGCATCCTGTGACAGAAATGATTTCATCTGTTGATTTGATTGAAGAGCAAATTCGTGTAGCTTTGGGAGAAAAGCTTCGA‑‑‑‑‑‑‑‑‑‑‑‑‑‑‑TACAAACAGGAAGATATTGTTCTCAAGGGACATTCTATCGAATGCCGCATCAATGCAGAAGATGCTTTTAAGGGATTTAGACCAGGACCAGGAAGAATAACAGCATACCTGCCATCTGGAGGTCCATTTGTCAGAATGGACAGCCATGTTTATCCAGGTTATGTT‑‑‑GTACCTCCAAGCTACGACTCCCTCCTTGGAAAGCTAATTGTGTGGGCTCCAACAAGGGAAAAAGCAATTGAACGCATGAAAAGGGCACTTGACGACACCATTATCACAGGGGTACCTACAACAATTGATTATCATAAACTGATCCTTGACATAGAGGATTTCAGAAATGGAAAGGTTGACACTGCTTTTATTCCAAAGCATGAGGAGGAGTTGGCTACG‑‑‑CCTGCTCCGAAG‑‑‑CATTGGCCGATGGTAACTACG‑‑‑AACAAGAAC‑‑‑‑‑‑‑‑‑‑‑‑‑‑‑‑‑‑

>Car_accC_Ca_05874

ATGGAAGCAACA‑‑‑‑‑‑ATGTCG‑‑‑GCGTGCAAGTCTCTTACCTCACCTTCCACCGTTCCC‑‑‑‑‑‑GTC‑‑‑ACAGGTTTATTTGCGGTGAGAGGAGGAGGAATC‑‑‑AAGAGTTCGCATTCA‑‑‑CAGTGC‑‑‑AGTTTCTTAACG‑‑‑GGAACTAACAAGGTGAAGTTTCCGAGGCAAACTACT‑‑‑CAACCT‑‑‑TGT‑‑‑‑‑‑‑‑‑AAACACCGTCAAACGCGT‑‑‑‑‑‑CATTGTGGTGCTCTTCGTGCGACTTGT‑‑‑‑‑‑‑‑‑‑‑‑CGT‑‑‑GCAGATAAGATTCTGATCGCGAATAGAGGTGAAATTGCGGTTCGTATTATTCGTACTGCTCATGAATTGGGGATTCCGTGTGTCGCTGTTTATTCCACCGTTGATAAGGATGCTCTTCATGTTAAATTGGCTGATGAATCTGTTTGCATTGGTGAAGCTCCTAGTAGTCAATCGTATTTACTGATTCCAAATGTTCTGGCTGCTGCAATTAGCCGACAATGCACAATGTTGCATCCTGGATATGGTTTTCTTGCTGAGAATGCAGTGTTTGTTGACATGTGTAGAGAACATGGAATCAATTTTATTGGGCCTAATCCTGATAGTATTCGGGTTATGGGTGATAAAGCAACTGCCAGAGAGACCATGAAGAAAGCAAACGTTCCTACTGTTCCAGGAAGTGATGGGCTTTTGCAGACCACTGAAGAAGCTATCAGGGTGGCACACGAAATTGGTTTCCCTGTGATGATCAAGGCAACCGCAGGTGGTGGAGGTCGTGGTATGCGTCTTGCTAAAGAACCTGAAGAGTTTGTGAAGTTATTACAGCAAGCTAAGAGCGAGGCAGCTGCTGCATTTGGAAATGATGGGGTTTATTTGGAGAAGTACATTCAAAACCCAAGGCACATTGAGTTCCAGGTTCTTGCAGATAAATATGGTAATGTTGTTCACTTTGGAGAACGTGATTGCAGTATCCAGAGGCGTAACCAGAAACTGCTTGAAGAAGCACCATCTCCTGCATTGACCCCAGAGTTACGGAAGGCAATGGGAGATGCAGCAGTTGCTGCAGCCGCATCTATTGGTTACATAGGTGTTGGAACGGTTGAGTTCCTTTTGGATGAAAGGGGTTCTTTTTACTTCATGGAGATGAACACTCGTATCCAGGTTGAACATCCTGTGACAGAAATGATTTCCTCTGTTGATTTGATAGAAGAGCAAATTCGTGTAGCTATGGGAGAAAAGCTTCGA‑‑‑‑‑‑‑‑‑‑‑‑‑‑‑TACACTCAGGAGGATATTGTGCTCAGAGGACATTCTATTGAATGCCGTATCAATGCAGAAGATGCTTTTAAGGGATTTAGACCAGGGCCAGGTAGAATTACAGCATACTTGCCCTCTGGAGGTCCATTTGTCAGAATGGATAGTCACATTTACCCGGATTATGTT‑‑‑GTTCCTCCAAGCTATGATTCTCTTCTTGGAAAGCTGATTGTATGGGCTCCGACTAGGGAAAAAGCAATTGAACGCATGAAAAGGGCACTCGATGACACTATTATCACAGGAGTTCCAACAACAATTGATTATCATAAACTTATCCTTGACATAGAGGATTTCAAAAATGGCAAGGTTGATACTGCTTTCATTCCAAAGCATGAGGAAGAGTTGGCTGTG‑‑‑CCCCCGCAGCAGAAGATGGTATTAGTT‑‑‑AACAAG‑‑‑GTGACAAAACTAACTGGCTCGACAGCC

>Cca_accC_C.cajan_23212

‑‑‑‑‑‑‑‑‑‑‑‑‑‑‑‑‑‑‑‑‑‑‑‑‑‑‑‑‑‑‑‑‑‑‑‑‑‑‑‑‑‑‑‑‑‑‑‑‑‑‑‑‑‑‑‑‑‑‑‑‑‑‑‑‑‑‑‑‑‑‑‑‑‑‑‑‑‑GGTTTATTTGCGGGGAAAGGA‑‑‑GGGATT‑‑‑AAGAGC‑‑‑‑‑‑TCT‑‑‑CAATGC‑‑‑AGTTTCCTAGCG‑‑‑GGAACGAACCGAGTGAGGTTCCCCAGGCAAGTAGGT‑‑‑CAGGTT‑‑‑AGTCGATTTCACAAACAACGCCAAACGCGT‑‑‑‑‑‑CACTGCGGCGCGCTCCACGCCACTTGT‑‑‑‑‑‑‑‑‑‑‑‑AAC‑‑‑GGTAACAAGATCCTCATCGCGAATAGAGGCGAGATTGCGGTTCGCGTCATTCGAACCGCTCATGAGTTGGGGATTCCCTGTGTGGCTGTGTACTCCACCATTGATAAGGACGCGCTTCATGTCAAATTAGCTGATGAAGCCGTTTGCATCGGCGAAGCGCCGAGTAGCCAGTCATACTTGCTGATCCCGAATGTTCTTTCGGCTGCAATTAGCCGCAGATGCACGATGTTGCATCCTGGCTATGGTTTTCTGGCGGAGAATGCAGTGTTTGTTGAAATGTGCACGGAACATGGAATCAACTTTATTGGGCCTAACCCTGACAGTATTCGGGTTATGGGTGACAAATCAACTGCAAGGGAGACAATGAAGAAAGCAGGTGTTCCCACTGTTCCGGGAAGTGAAGGCCTTTTGCAGACCACTGAAGAAGCTATCAGGCTGGCAAATGAGATTGGTTTCCCTGTGATGATCAAGGCAACAGCTGGTGGTGGGGGACGTGGCATGCGTCTTGCTAAAGAACCTGATGAGTTTGTGAAGTTGTTACAGCAAGCTAAGAGTGAAGCAGCTGCTGCATTTGGAAATGATGGGGTATATTTGGAGAAGTACATTCAAAACCCAAGGCACATTGAGTTCCAGGTTCTTGCAGATAAATATGGTAATGTTGTTCACTTTGGAGAACGTGATTGCAGCATTCAGAGGCGTAATCAAAAACTGCTTGAAGAAGCACCATCTCCTGCACTGACCCCTGAGTTGCGAAAGGCAATGGGAGATGCAGCAGTGGCAGCTGCTGCATCTATTGGTTACATAGGTGTTGGAACGGTTGAATTCCTTTTGGATGAAAGGGGTTCCTTTTACTTCATGGAGATGAACACTCGTATCCAGGTTGAGCATCCTGTGACAGAAATGATTTCCTCAACTGATTTGATAGAAGAGCAAATTCGTGTTGCTATGGGAGAAAAACTTCGA‑‑‑‑‑‑‑‑‑‑‑‑‑‑‑TACAAACAGGAGGATATTGTGCTCAGAGGACATTCTATTGAATGCCGTATCAATGCAGAAGATGCTTTTAAGGGATTTAGACCAGGGCCAGGTAGAATAACAGCATACTTGCCATCTGGAGGTCCATTTGTCAGAATGGATAGTCATGTTTATCCTGATTATGTG‑‑‑GTTCCTCCAAGCTACGACTCTCTTCTTGGAAAGCTGATTGTATGGGCTCCAACAAGAGAAAAAGCAATTGAACGCATGAAAAGGGCACTTGATGACACGATTATCACAGGAGTTCCTACTACAATTGATTATCATAAACTTATCCTCGACATAGAGGATTTCAGAAATGGCAATGTTGATACTGCTTTTATTCCTAAACATGAGGAAGAGTTGGCAATG‑‑‑CCGCCACAGAAG‑‑‑ATGGTACTCTCT‑‑‑AACACG‑‑‑GTGAAAGAACTGGCTGGCTCAATTGCC

>Ccan_accC_RKFX‑2047844‑Cercis_canadensis

ATGGAGGCTACA‑‑‑‑‑‑ATGCCT‑‑‑GTCTGCAAGTCTGTTAGCTCGCCTACTGTA‑‑‑‑‑‑‑‑‑‑‑‑‑‑‑‑‑‑‑‑‑GGTTTATTTGTGGGGAGAACTAGAGGAATT‑‑‑AAGAGT‑‑‑‑‑‑TCT‑‑‑CAATGC‑‑‑AGCTTTTTAGTG‑‑‑GGA‑‑‑AATAAAGTGAACTTTCCTAGGCAAAGAGTT‑‑‑CAAAGT‑‑‑GCTCAAGTA‑‑‑AGTTGGAAACATAGAAGG‑‑‑‑‑‑CATTGTGGGGCTCTCCATGCAACATGT‑‑‑‑‑‑‑‑‑‑‑‑CAA‑‑‑GCTGATAAAATCCTGGTGGCAAATAGAGGGGAAATTGCTGTTCGTGTTATTAGAACTGCTCATGAATTGGGGATACCTTGTGTGGCTGTGTACTCAACCATAGACAAGGATGCACTTCATGTGAAATTGGCTGATGAAGCAGTTTGCATCGGTGAAGCACCCAGTAGTCAATCGTACTTATTAATTCCAAATGTTCTATCAGCTGCTATCAGCCGTAAATGCACAATGTTGCATCCTGGATATGGTTTTCTTGCTGAAAATGCAGTATTTGTTGAAATGTGCAGAGAACATGGAATCAATTTTATTGGGCCCAATCCTGATAGTATTCGGGTTATGGGTGACAAATCAACTGCCAGAGACACAATGAAGAAAGCAGGTGTTCCCACTGTTCCAGGAAGTGATGGACTCTTGCAGACCACGGATGAAGCAATCAGGCTGGCGAATGAGATTGGTTTCCCTGTGATGATCAAGGCAACAGCAGGTGGTGGAGGGCGTGGCATGCGTCTTGCCAAAGAACCTGATGAGTTTGTGAAGTTGTTACAGCAAGCTAAGAGTGAGGCGGCTGCTGCTTTTGGCAATGATGGGGTTTATTTGGAAAAGTACATTCAAAATCCCAGGCACATTGAGTTCCAGGTTCTTGCAGATAAATACGGTAATGTTGTTCACTTCGGAGAGCGTGATTGCAGCATCCAGAGGCGAAACCAAAAACTGTTGGAAGAAGCACCTTCTCCTGCATTGACCCCAGAGTTGCGAAAGGCAATGGGTGATGCAGCGGTTGCAGCAGCTGCATCTATAGGTTACATAGGTGTTGGAACAGTTGAATTCCTCTTGGACGAGAGAGGTTCCTTTTACTTCATGGAGATGAACACTCGTATTCAGGTGGAGCATCCTGTGACGGAAATGATTTCCTCTGTTGATTTGATCGAAGAGCAAATTTGTGTAGCTTTGGGAGAAAAACTTCGA‑‑‑‑‑‑‑‑‑‑‑‑‑‑‑TACAAACAGGAAGAGATTGTGCTCAGAGGACATTCTATTGAATGCCGCATCAATGCAGAAGATGCTTTCAAGGGATTTAGACCAGGACCAGGAAGAATAACAGCATACTTGCCATCTGGAGGTCCATTTGTCAGAATGGACAGCCATGTTTATCCAGATTATGTG‑‑‑GTCCCTCCAAGCTATGACTCCCTTCTTGGAAAGCTGATTGTGTGGGCTCCAACAAGGGAAAAAGCAATTGAGCGCATGAAAAGGGCGCTTGACGACACCATTATCACAGGGGTCCCTACAACAATTGATTATCATAAACTGATCCTTGACGTAGAGGATTTCAGAAATGGAAAGGTTGACACTGCTTTTATTCCAAAGCATGAGCAGGAGTTGGCTATG‑‑‑CCGGCCCCGAAG‑‑‑ATAGTATTATCC‑‑‑GACAGG‑‑‑GCCAAAGAACTAGCTGGTGCAACTGCA

>Cof_accC_RKLL‑2067350‑Copaifera_officianalis

ATGGAGGCTACA‑‑‑‑‑‑TTGCCT‑‑‑GTGTGCCATTCCGTCTGCTCCCCTTCTGTC‑‑‑‑‑‑‑‑‑‑‑‑‑‑‑‑‑‑‑‑‑GGTTTGTTTTTGGGAAGAACTAGAGGAATA‑‑‑AGGAGT‑‑‑‑‑‑TCC‑‑‑CAATGC‑‑‑AGCTTTTTAGCG‑‑‑‑‑‑GCAAATAAACTCAACTTTCCCTTGCAAAGAGCT‑‑‑CAAACT‑‑‑ATTCGTAAA‑‑‑CAACAGCATACGAGGCGG‑‑‑‑‑‑CATAGCGGGGCTCTCAAGGCAACTTGC‑‑‑‑‑‑‑‑‑‑‑‑AAC‑‑‑GCTGATAAAATATTGGTGGCAAATAGAGGAGAGATTGCTGTTCGTGTCATTAGAACTGCTCATGAAATGGGGATACCTTGTGTGGCTGTCTACTCAACCATAGATAAGGATGCACTTCATGTCAAACTGGCCGATGACTCGGTTTGCATAGGCGAAGCCCCCAGTAGTCAGTCGTACTTGTTGATTCCAAATGTTCTATCCGCGGCTATCAGCCGTAAATGTACAATGCTGCATCCTGGATATGGTTTTCTGGCTGAAAATGCAGTTTTTGTTGAAATGTGCAGAGAGCATGGGATTAATTTTATTGGGCCTAACCCTGACAGTATACGGGTTATGGGCGACAAATCAACTGCCAGAGACACAATGAAGAAAGCAGGTGTTCCCACTGTTCCAGGAAGTGATGGACTGTTACAGAGCACAGAGGAAGCAATCAGGTTGGCGAATGAGATTGGTTTTCCTGTGATGATCAAGGCTACAGCAGGTGGCGGAGGCCGTGGAATGCGTCTTGCTAAAGAATCTGATGAGTTTGTGAAATTGTTACAGCAAGCTAAGAGTGAGGCAGCCGCTGCATTTGGCAATGATGGAGTTTATCTGGAAAAGTACATTCAAAATCCAAGGCACATAGAGTTCCAAGTACTAGCAGATAAATTTGGCAATGTTGTTCACTTTGGAGAGCGCGATTGCAGCATCCAGAGGAGAAATCAGAAGCTGCTGGAAGAAGCTCCTTCTCCTGCATTGAGCCCAGAATTGCGGAAGGCGATGGGTGATGCAGCAGTTGCAGCAGCTGCATCTATAGGTTACATAGGTGTTGGCACGATTGAGTTCCTCTTGGATGAAAGAGGTTCCTTTTACTTCATGGAGATGAACACTCGTATTCAGGTTGAGCATCCTGTGACAGAAATGATTTCCTCTGTTGATTTGATTGAAGAGCAAATTCGTGTAGCTATGGGAGAAAAACTTAGA‑‑‑‑‑‑‑‑‑‑‑‑‑‑‑TACAAACAGGAAGATATTGTGCTCAGGGGACATTCTATTGAATGCCGTATCAATGCAGAAGATGCTTTTAAGGGATTTAGGCCAGGGCCAGGAAGAATAACAGCATACCTGCCATCTGGGGGTCCATTTGTTAGAATGGACAGCCACGTTTATCCTGATTATGTG‑‑‑GTTCCACCAAGCTATGACTCCCTTCTTGGGAAGTTAATTGTATGGGCTCCAACAAGAGAAAAAGCAATTGAGCGCATGAAGAGGGCACTCGATGACACCGTTATCACAGGGGTTCCAACAACAATTGATTATCATAAACTTATCCTTGACATAGAGGATTTCAGAAATGGCGAGGTTGATACTGCTTTTATTCCAAAGCATGAGCAGGAATTGGCAATG‑‑‑CCGCCCCAG‑‑‑‑‑‑ATGGCAAGAGCC‑‑‑GACACT‑‑‑ACCAAACGACTAGCTGGTGCAACCGCA

>Cre_accC_XM_001702267

ATGCCCGCT‑‑‑‑‑‑‑‑‑‑‑‑‑‑‑‑‑‑‑‑‑‑‑‑‑‑‑‑‑‑‑‑‑‑‑‑‑‑‑‑‑‑‑‑‑‑‑‑‑‑‑‑‑‑‑‑‑‑‑‑‑‑‑‑‑‑‑‑‑‑‑‑‑‑‑‑‑‑‑‑‑‑‑‑‑‑‑‑‑‑‑‑‑‑‑‑‑‑‑‑‑‑AAGGGC‑‑‑‑‑‑‑‑‑‑‑‑‑‑‑‑‑‑‑‑‑‑‑‑‑‑‑‑‑‑‑‑‑‑‑‑‑‑‑‑‑‑‑‑‑‑‑‑GTCTCGGGCCGCCGCAGCCGCACC‑‑‑CAG‑‑‑‑‑‑‑‑‑‑‑‑‑‑‑‑‑‑‑‑‑‑‑‑‑‑‑‑‑‑‑‑‑‑‑‑‑‑‑‑‑‑‑‑‑‑‑‑‑‑‑‑‑‑GTCGCCGCGCAGGCT‑‑‑‑‑‑‑‑‑‑‑‑GCC‑‑‑TTCAACAAGGTGCTCATCGCCAACCGCGGCGAGATTGCTGTGCGCGTCATTCGCGCCTGCAAGGAGCTGGGCCTGCAGACCGTGGCCGTCTACTCCACCGCTGACAAGAACTCGCTGCACACGCTGCTGGCCGATGAGGCGGTGTGCATCGGCGACGCGCCCTCCTCCGAGTCCTACCTCAACATCCCCAACCTGCTGGCGGCCGCCGTCAGCCGCGGCGCCCAGGCCGTGCACCCCGGCTACGGCTTCCTGTCTGAGCGCGCCGAGTTTGTGGAGATCTGCACCGACCACGGCCTGGAGTTCATCGGGCCCAAGCCGCAGCAGATCCGGCTCATGGGCGACAAGTCCACCGCCCGCGACACCATGAAGAAAGCCGGTGTGCCCACCGTGCCCGGCTCCGAGGGCCTGATCCAGGACGACGCCGACGCCATTGCCAACGCCCAGCGCATCGGCTTCCCCGTCATGATCAAGGCCACCGCGGGTGGCGGCGGCCGCGGTATGCGCCTGTGCACCAAGGAGGAGGACCTCATGCCCCTGTTCAAGCAGGCCCAGCAGGAGGCCGAGGCCGCGTTCGGCAACGGCGCCGTGTACATGGAGCGCTTCGTGCAGGACCCCCGCCACATCGAGTTCCAGGTCCTGGCGGACAAGTACGGCAACGTGGTGCACCTGGGTGAGCGCGACTGCTCGGTGCAGCGCCGCAACCAGAAGCTGGTGGAGGAGGCGCCCTCGCCCGCCCTCACGCCCGAGGTCCGCAAGGCCATGGGCGACGCCGCGGTGGCCGCCGCCAAGGCCATTGGCTACGTGGGCGTGGGCACCATTGAGTTCCTGTGGGAGAAGAAGGGC‑‑‑TTCTACTTCATGGAGATGAACACCCGCATCCAGGTGAGGCCGCGTGTGCGTGCCTTTATATCAAACGGGAACACTACTTCGGAGGGTCTACGCGCTGGAGCTGGAGCTGGAGCTCGGGCTGGAGCCAGGAGCTGGTCGACATCGTCGGTAACGCTTCATGGCCACGCCATCGAGTGCCGCATCAACGCCGAGGACCCCTTCCAGAACTTCCGCCCCGGCCCCGGCCGCGTGTCCACCTACCTGGCGCCCGGCGGCCCCCACGTGCGCATGGACAGCCACCTCTCCCCACGTGCTGTCCCCGTCCCCCCGAACTACGACTCCCTGCTGGGCAAGCTGATTGTGTGGGGCGCAGACCGCCAGCAGGCCGTACGCGTACGCATCCGCGCGCTGGACGAGCTGGTGATCACGGGCGTGCCCACCACCGCGCCCTTCCACAAGCTCATCCTGGCCACGGAGGCCTTCAAGGCCGGCGACGTTGACACGGGCTTCATCATCAAGCACGCTGAGGAGCTGAAGGAG‑‑‑TCGCCGGGCGTGCCCAAGGTGTGTGTC‑‑‑‑‑‑‑‑‑‑‑‑‑‑‑‑‑‑‑‑‑‑‑‑‑‑‑‑‑‑‑‑‑‑‑‑‑‑‑

>Gma_accC1_Glyma.05G221100

ATGGAAGTCACA‑‑‑‑‑‑CTTTCA‑‑‑GCGTGCAAGTCCATTAGCTCGCCTTCTGTT‑‑‑CCC‑‑‑‑‑‑GTC‑‑‑GCAGCTTTATTTGCGGGGAAAGCA‑‑‑GGAACT‑‑‑AAGAGT‑‑‑‑‑‑TCG‑‑‑CAATGC‑‑‑AGTTTTTTAGCT‑‑‑GGAGCAAACAAAGTGAGGTTTCCTAGGCAAGTCGGT‑‑‑CAAGTT‑‑‑AGCCACGTTCGCAAACAGCGCCAAACGCGT‑‑‑‑‑‑CACTGTGGTGCTCTCCACGCGACTTGC‑‑‑‑‑‑‑‑‑‑‑‑AGC‑‑‑GGTGACAAGATCCTGATCGCGAATAGAGGCGAGATTGCGGTTCGCGTTATTCGAACCGCTCATGAATTGGGGATTCCGTGTGTGGCTGTGTACTCCACCATTGATAAGGACGCCCTTCATGTCAAACTGGCTGATGAAGCTGTATGCATCGGCGAAGCGCCGAGTAGCCAGTCGTACTTGCTGATCCCGAATGTTCTGTCGGCTGCTATTAGCCGCAGATGCACGATGTTGCATCCTGGCTATGGTTTTCTTGCGGAGAATGCTGTGTTTGTTGAAATGTGCAGGGAACATGGAATCAACTTTATTGGGCCCAACCCTGACAGTATTCGGGTTATGGGTGACAAAGCAACTGCCAGAGAGACAATGAAGAAAGCAGGTGTTCCCACCGTTCCAGGAAGTGATGGCCTTTTACAGAGCACAGAAGAAGCTATTAGGCTGGCAAATGAGATTGGTTTCCCTGTGATGATCAAGGCAACAGCTGGTGGTGGAGGACGTGGCATGCGTCTTGCTAAAGAACCTGATGAGTTTGTGAAGTTGTTACAGCAAGCTAAGAGTGAGGCAGCTGCTGCATTTGGCAATGACGGGGTTTATTTGGAGAAGTACATTCAAAACCCAAGGCACATTGAGTTCCAGGTTCTTGCAGATAAATATGGTAATGTTGTTCACTTTGGAGAACGTGATTGCAGCATTCAGAGGCGTAATCAGAAACTGCTTGAAGAAGCACCATCTCCTGCCCTGACCCCTGAGTTGCGAAAGGCAATGGGAGATGCAGCAGTTGCTGCTGCTGCATCTATTGGTTACATAGGTGTTGGAACGATTGAGTTTCTTTTGGATGAAAGAGGTTCCTTTTACTTTATGGAGATGAACACACGTATCCAGGTTGAGCATCCTGTGACAGAAATGATTTCCTCAACAGATTTGATAGAAGAGCAAATTCGTGTAGCTATGGGAGAAAAACTTCGA‑‑‑‑‑‑‑‑‑‑‑‑‑‑‑TACAAACAGGAGGATATTGTCCTCAGAGGACATTCTATTGAATGCCGTATCAATGCAGAAGATGCTTTTAAGGGATTTAGACCAGGGCCAGGTAGAATTACAGCATACTTGCCATCTGGAGGTCCATTTGTTAGAATGGATAGCCATGTTTATCCTGATTATGTG‑‑‑GTTCCTCCAAGCTATGACTCCCTTCTTGGAAAGCTGATTGTATGGGCTCCAACAAGAGAAAAAGCAATTGAACGCATGAAAAGGGCACTTGATGACACTATTATCACAGGAGTTCCTACTACAATTGATTATCATAAACTTATCCTTGACATAGAGGATTTCAGAAATGGAAAGGTTGATACTGCTTTTATTCCAAAACACGAGGAAGAGTTGGCAATG‑‑‑CCTCCGCAGAAG‑‑‑ATGGTACTCGCT‑‑‑AACAGG‑‑‑GTGAACGAACTAGCTGGTTCAACTGCC

>Gma_accC2_Glyma.08G027600

ATGGAAGTCACA‑‑‑‑‑‑CTTTCA‑‑‑GCCTGCAAGTCCGTTAGCTCGCCTTCTGTT‑‑‑CCC‑‑‑‑‑‑GTC‑‑‑GCAGGTTTGTTTGCGGGGAATGGA‑‑‑GGAATT‑‑‑AAGAGC‑‑‑‑‑‑TCG‑‑‑CAATGC‑‑‑AGTTTCTTAGCG‑‑‑GGAGCGAGCAAAGTGAGGTTTCCTAGGCAAGTAGGT‑‑‑CAAGTT‑‑‑AGCCACCTTCGCAAACAGCGCCAAACGCGT‑‑‑‑‑‑CACTGCGGTGCTCTCCACGCGACTTGC‑‑‑‑‑‑‑‑‑‑‑‑AGC‑‑‑GGTGACAAGATCCTGATCGCGAACAGAGGCGAGATTGCCGTCCGCGTTATTCGAACCGCTCATGAATTGGGGATTCCTTGTGTGGCTGTGTACTCCACCATCGACAAGGACGCGCTTCATGTCAAATTGGCTGATGAAGCCGTTTGCATCGGGGAAGCGCCGAGTAGCCAGTCCTACTTGCTGATCCCGAATGTTCTGTCAGCTGCTATTAGCCGCAGATGCACCATGTTGCATCCTGGCTATGGTTTTCTTGCGGAGAATGCAGTGTTTGTTGAAATGTGCAGAGAACATGGAATCAACTTTATTGGGCCTAACCCTGACAGTATTCGGGTTATGGGTGACAAAGCAACTGCCAGAGAGACAATGAAGAAAGCAGGTGTTCCCACCGTTCCGGGAAGTGATGGCCTTTTACAGAGCACAGAAGAAGCTATCAGACTGGCAAATGAGATTGGTTTCCCTGTGATGATCAAGGCAACAGCTGGTGGTGGAGGACGTGGCATGCGTCTTGCTAAAGAACCTGCTGAGTTTGTGAAGTTTTTACAGCAAGCTAAGAGTGAGGCAGCTGCTGCATTTGGCAATGATGGGGTTTATTTGGAGAAGTACATTCAAAACCCAAGGCACATTGAGTTCCAGGTTCTTGCAGATAAATATGGTAATGTTGTTCACTTTGGAGAACGTGATTGCAGCATTCAGAGGCGCAACCAGAAACTGCTTGAAGAAGCACCATCTCCTGCCCTGACCCCTGAGTTGCGAAAGGCAATGGGAGATGCAGCAGTTGCAGCTGCTGCATCTATTGGTTACATAGGTGTTGGAACGGTTGAGTTCCTTTTGGATGAAAGAGGTTCCTTTTACTTTATGGAGATGAACACTCGTATCCAGGTTGAGCATCCGGTGACAGAAATGATTTCCTCAACGGATTTGATAGAAGAGCAAATTCGTGTAGCTATGGGAGAAAAACTTCGA‑‑‑‑‑‑‑‑‑‑‑‑‑‑‑TACAAACAGGAGGATATTGTCCTCAGAGGACATTCTATTGAATGCCGTATCAATGCAGAAGATGCTTTTAAGGGATTTAGACCAGGGCCAGGTAGAATTACTGCATACTTGCCATCCGGAGGTCCATTTGTTAGAATGGATAGCCATGTTTATCCTGATTATGTG‑‑‑GTTCCTCCAAGCTATGATTCCCTTCTTGGAAAGCTGATTGTATGGGCTCCAACTAGAGAAAAAGCAATTGAACGCATGAAAAGGGCACTTGATGATACTATTATCACAGGAGTTCCTACTACAATTGATTATCATAAACTTATCCTTGACATAGAGGATTTCAGAAATGGCAAGGTTGATACTGCTTTTATTCCAAAACACGAGGAAGAGTTGGCAATG‑‑‑CCACCGCAGAAG‑‑‑ATGGTACTCGCT‑‑‑AACAGG‑‑‑GTGAATGAACTAGCTGGTTCAACTGCC

>Gpo_accC_VLNB‑2076119‑Gompholobium_polymorphum

ATGGAGGCCACA‑‑‑‑‑‑ATGTCA‑‑‑GCGTGCAAGTCTGTTGGCTCGCCTTCTGTC‑‑‑CCC‑‑‑‑‑‑GTT‑‑‑GCAGGTTTATTTGTTGGGAGAGGA‑‑‑GGAATG‑‑‑AAGAGT‑‑‑‑‑‑TCC‑‑‑CAGTGC‑‑‑ATCTTCCTAGCG‑‑‑GGAACGAATAAGGTGAGGTTTCCTAGGCAAACAGCT‑‑‑CAACAT‑‑‑AGTCACACTCGCAAACAACACCAAACGCGT‑‑‑‑‑‑CGTTGTGGGGCTCTCCATGCAACTTGT‑‑‑‑‑‑‑‑‑‑‑‑CAA‑‑‑GGTGATAAGATCCTGATCGCAAATAGAGGCGAGATTGCCGTTCGTGTTATTCGTACCGCTCATGAACTGGGGATACCGTGTGTGGCCGTGTACTCAACCATTGACAAGGATGCGCTTCATGTCAAATTGGCTGATGAAGCCGTTTGCATAGGTGAAGCGCCAAGTAGTCAATCGTACTTACTGATCCCAAATGTTCTGTCTGCTGCTATTAGCCGCAAATGCACAATGTTGCATCCCGGATACGGTTTTCTTGCTGAGAATGCAGTGTTTGTTGAAATGTGCAGAGAACATGGAATCAATTTTATTGGGCCTAATCCTGACAGTATTCGAGTTATGGGTGACAAATCAACTGCCAGAGACACAATGAAGAAAGCAGGTGTTCCTACTGTTCCAGGAAGTGATGGGCTTTTACAGAGCACGGAAGAAGCTATCAGGCTGGCAAGTGAAATTGGTTTTCCTGTGATGATCAAGGCGACAGCAGGTGGAGGAGGACGTGGCATGCGTCTTGCTAAAGAACCTGATGAGTTTGTGAAGTTGTTGCAGCAAGCTAAGAGTGAGGCAGCTGCTGCATTTGGCAATGACGGGGTTTATTTGGAGAAGTACGTTCAAAATCCAAGGCACATTGAGTTCCAGGTTCTTGCAGATAAATACGGTAATGTTGTTCACTTTGGAGAACGTGATTGCAGCATCCAGAGGCGTAATCAAAAACTGCTGGAAGAAGCACCATCTCCTGCATTGACCCCAGAGTTGCGCAAGGCAATGGGAGATGCAGCAGTTGCGGCAGCTGCATCTATTGGCTACATAGGCGTTGGAACGGTTGAGTTTCTCTTGGATGAAAGGGGTTCCTTTTACTTCATGGAGATGAACACTCGTATCCAGGTTGAACATCCTGTGACAGAAATGATTTCCTCTGTTGATTTGATAGAAGAACAAATTCGTGTAGCTATGGGAGAAAAGCTTCGA‑‑‑‑‑‑‑‑‑‑‑‑‑‑‑TACAAACAGGAGGAAATTGTGCTCAGAGGACATTCTATTGAATGTCGTATCAATGCAGAAGATGCTTTTAAGGGATTTAGACCAGGGCCAGGTAGAATTACAGCATACTTGCCATCTGGAGGTCCATTTGTCAGAATGGATAGCCATGTTTATCCTGACTATGTG‑‑‑GTTCCTCCAAGCTATGACTCTCTTCTTGGAAAGCTGATTGTGTGGGCTCCAACAAGAGAAAAAGCAATCGAACGCATGAAAAGGGCACTTGATGACACTATTATCACAGGAATTCCTACAACAATTGATTATCATAAACTTATCCTTGATATAGAGGATTTCAGAAATGGCAAGGTTGATACCGCTTTTATTCCAAAGCATGAGGAGGAATTGGCAATG‑‑‑CCGCCACAGAAG‑‑‑ATGGTACTAGCT‑‑‑AACAGG‑‑‑GTGAAAGATCTAGCTGGCTCAACTGCC

>Lal_accC1_LAGI01_20167

ATGGATTCCACTCTCTCAATCTCA‑‑‑CTCTCCACATCTCTTACCTCACCTTCTGTT‑‑‑CCT‑‑‑‑‑‑CTT‑‑‑TCAGGTTTATTTGCTGTGAGAAGTAGAGGAATG‑‑‑AAGAGT‑‑‑‑‑‑TCA‑‑‑CAATGC‑‑‑AGTTTCTTACCT‑‑‑GGAACTAATAATGTGAGTTTTCCTAAACAAACTACTGTTCAACAA‑‑‑GTTAGTTAT‑‑‑AAAAGAGAACGAAGAGCG‑‑‑‑‑‑TGTTGTGGGGCTCTTGGTGCAACTTGT‑‑‑‑‑‑‑‑‑‑‑‑GGA‑‑‑GGGGATAAGATCTTGATTGCGAATAGAGGCGAAATCGCGGTTCGTGTTATTCGAACTGCTCATGAATTGGGGATACCATGTGTGGCTGTGTACTCTACAGTTGACAAGGATGCACTTCATGTTAAATTGGCTGATGAGGCTGTTTGTATTGGCGAAGCGCCGAGTAGCCAATCGTACTTGCTTATCCCGAATGTCCTTTCTGCTGCTATTAGTCACAAATGCACGATGTTGCATCCTGGATATGGTTTTCTTTCTGAGAATGCGGTGTTTGTTGATATGTGTAGAGAACATGGAATCAATTTTATTGGGCCTAATCCTGACAGTATTCGGGTTATGGGTGACAAAGCAACTGCCCGAGAGACAATGAAGAAAGCAGGTGTTCCTACTGTTCCAGGAAGTGATGGGCTTTTACAGAGTACGGAAGAAGCTATCAGGGTCGCAAATGAGATTGGTTTCCCCGTGATGATCAAGGCAACAGCAGGTGGCGGAGGACGTGGTATGCGTCTTGCTAAAGAACCTGATGAGTTTGTTAAGTTGTTACAGCAAGCTAAGAGCGAAGCAGCTGCTGCCTTTGGCAATGATGGTGTTTATCTGGAGAAGTATGTTCAAAACCCAAGGCACATTGAGTTCCAGGTTCTTGCAGATAAATATGGTAATGTTGTTCACTTTGGAGAACGTGATTGCAGCATCCAGAGGCGTAATCAGAAACTGCTGGAAGAAGCACCTTCTCCTGCATTGACCCCAGAGTTGCGGAAGGCAATGGGAGATGCAGCAGTTGCAGCGGCTGCCTCTATTGGTTACGTTGGTGTTGGAACAGTTGAGTTTCTATTGGATGAAAGGGGTTCCTTTTACTTCATGGAGATGAACACTCGTATTCAGGTTGAACATCCTGTTACAGAAATGATTTCCTCTGTTGATTTGATAGAAGAGCAAATTCTTGTAGCTATGGGAGAAAAACTTCGA‑‑‑‑‑‑‑‑‑‑‑‑‑‑‑TACAAACAGGAGGATATTGTGCTCCAAGGACATTCTATCGAATGCCGTATCAACGCAGAAGACGCGTTTAAGAATTTTAGACCTGGGCCAGGTAGAATTACAGCATACTTGCCCTCTGGAGGTCCATTTGTCAGAATGGATAGCCATGTTTATCCCGATTATGTG‑‑‑GTTCCTCCAAGCTATGACTCCCTTCTTGGAAAGCTGATTGTATGGGCTCCAACAAGAGAAAAAGCAATTGAGCGCATGAAAAGGGCACTTGATGACACTACTATCACAGGAATTCCTACAACAATCGAATATCATAAACTTATCCTTGACATTGAGGATTTCAGAAATGGCAAGGTTGACACTGCTTTCATTCCAAAGCATGAGGAGGAATTGGCAATG‑‑‑CCACCCCAGAAG‑‑‑ATTGTACCATCT‑‑‑AACAGG‑‑‑‑‑‑‑‑‑‑‑‑‑‑‑‑‑‑‑‑‑‑‑‑‑‑‑GCT

>Lal_accC2_LAGI01_21333

ATGGAATCCACAATGTCAATGTCT‑‑‑GTGTGCAAATCTCTTCACTCCCCTTCTTCT‑‑‑CCT‑‑‑‑‑‑ATC‑‑‑ACAGGTTTATTTGATGGGAGAAATAGAGGAATC‑‑‑AAGAGT‑‑‑‑‑‑TCT‑‑‑CAATGCAGAAGCTTGTTAGTTAGAAGAACCAATAAGGTGAATTTTCCTAAGCAAAATGTTAACAACAAAGGACTTAGTTAC‑‑‑AAACAAGAGCAACACAAAACGCAGCGTTGTGGGGCTCTTGCTGCTACTTGTGTTGTTGTTGGTGGT‑‑‑GGTGAGAAGATTTTGATTGCGAATCGAGGCGAGATCGCGGTTCGTGTGATTCGTACTGCTCATGAATTGGGGATACCTTGTGTGGCAGTGTACTCAACTATTGATAAGGATGCACTTCATGTTAAATTGGCTGATGAGGCTGTTTGTATTGGCGAAGCGCCAAGTAACCAATCGTACTTGCTTATCCCAAATGTCCTTTCTGCTGCTACTAGTCACAAATGCACGATGTTGCATCCCGGATATGGTTTTCTTTCTGAGAATGCAGTGTTTGTTGATATGTGTAGAGAACATGGAATCAATTTTATTGGGCCTAATCCTGACAGTATTCGGGTTATGGGTGACAAAGCAACTGCCCGAGAGACAATGAAGAAAGCAGGTGTTCCTACTGTTCCAGGAAGTGATGGGCTTTTACAGAGTACGGAAGAAGCTATCAGGGTCGCAAATGAGATTGGTTTCCCCGTGATGATCAAGGCAACAGCAGGTGGCGGAGGACGTGGCATGCGTCTTGCTAAAGAACCTGAAGAGTTTGTGAAGTTGTTACAGCAAGCTAAGAGCGAAGCAGCTGCTGCCTTTGGCAATGATGGTGTTTATCTGGAGAAGTATGTTCAAAACCCAAGGCACATTGAGTTCCAGGTTCTTGCAGATAAATATGGTAATGTTGTTCACTTTGGAGAACGTGATTGCAGCATCCAGAGGCGTAATCAGAAACTGATGGAAGAAGCACCTTCTCCTGCATTGACCCCAGAGTTGCGGAAGGCAATGGGAGATGCAGCAGTTGCAGCGGCTGCATCTATTGGTTACGTTGGTGTTGGAACAGTTGAGTTTCTATTGGATGAAAGGGGTTCCTTTTACTTCATGGAGATGAACACTCGTATTCAGGTTGAACATCCTGTTACAGAAATGATTTCCTCTGTTGATTTGATAGAAGAGCAAATTCTTGTAGCTATGGGAGAAAAACTTCGA‑‑‑‑‑‑‑‑‑‑‑‑‑‑‑TACAAACAGGAGGATATTGTGCTCCGTGGACATTCTATCGAATGCCGTATCAACGCAGAAGACGCGTTTAAGAATTTTAGACCTGGGCCAGGTAGAATTACAGCATACTTGCCCTCTGGAGGTCCATTTGTCAGAATGGATAGCCATGTTTATCCCGATTATGTG‑‑‑GTTCCTCCAAGCTATGACTCCCTTCTTGGAAAGCTGATTGTATGGGCTCCAACAAGAGAAAAAGCAATTCAACGCATGAAAAGGGCACTTGATGACACTATTATCACAGGAGTTCCTACAACAATTGATTATCATAAACTTATCCTTGACATTGAGGATTTCAGAAATGGCAAGGTTGACACTGCTTTTATTCCAAAGCATGAGGAGGAGTTGGCAATG‑‑‑CCGCCACAGAAG‑‑‑ATTGTACCATTC‑‑‑AACAGG‑‑‑‑‑‑‑‑‑‑‑‑‑‑‑‑‑‑‑‑‑‑‑‑‑‑‑GCG

>Lal_accC3_LAGI01_21523

ATGGAATCCACAATGTCAATGTCT‑‑‑GTGTGCAAATCTCTTCACTCCCCTTCTTCT‑‑‑CCT‑‑‑‑‑‑ATC‑‑‑ACAGGTTTATTTGATGGGAGAAATAGAGGAATC‑‑‑AAGAGT‑‑‑‑‑‑TCT‑‑‑CAATGCAGAAGCTTGTTAGTTAGAAGAACCAATAAGGTGAATTTTCCTAAGCAAAATGTTAACAACAAAGGACTTAGTTAC‑‑‑AAACAAGAGCAACACAAAACGCAGCGTTGTGGGGCTCTTGCTGCTACTTGTGTTGTTGTTGGTGGT‑‑‑GGTGAGAAGATTTTGATTGCGAATCGAGGCGAGATCGCGGTTCGTGTGATTCGTACTGCTCATGAATTGGGGATACCTTGTGTGGCAGTGTACTCAACTATTGATAAGGATGCACTTCATGTTAAATTGGCTGATGAGGCTGTTTGTATCGGCGAAGCGCCGAGTAGCCAATCGTACTTGCTGATCCCGAATGTCCTTTCTGCTGCTATTAGCCACAAATGCACAATGTTGCATCCTGGATATGGTTTTCTTGCTGAGAATGCGGTGTTTGTTGAAATGTGCAGAGAACATGGAATCAATTTTATTGGGCCTAATCCTGACAGTATTCGGGTTATGGGTGACAAAGCAACTGCCCGAGAGACAATGAAGAAAGCAGGTGTTCCTACTGTTCCAGGAAGTGATGGGCTTTTACAGAGCACGGAAGAAGCTATCAGGCTCGCAAATGAGATTGGTTTCCCCGTGATGATCAAGGCAACAGCAGGTGGCGGAGGACGTGGCATGCGTCTTGCTAAAGAACCTGAAGAGTTTGTGAAGTTGTTACAGCAAGCTAAGAGCGAAGCAGCTGCTGCCTTTGGCAATGACGGTGTTTATCTGGAGAAGTATGTTCAAAACCCAAGGCACATTGAGTTCCAGGTTCTTGCAGATAAATACGGTAATGTTGTTCACTTCGGAGAACGTGATTGCAGCATCCAGAGGCGTAATCAGAAACTGATGGAAGAAGCACCTTCTCCTGCATTGACCCCAGAGTTGCGGAAGGCAATGGGAGATGCAGCAGTTGCAGCGGCTGCATCTATTGGTTACGTTGGTGTTGGAACAGTTGAGTTTCTATTGGATGAAAGGGGTTCCTTTTACTTCATGGAGATGAACACTCGTATTCAGGTTGAACATCCTGTTACAGAAATGATTTCCTCTGTTGATTTGATAGAAGAGCAAATTCTTGTAGCTATGGGAGAAAAACTTCGA‑‑‑‑‑‑‑‑‑‑‑‑‑‑‑TACAAACAGGAGGATATTGTGCTCCAAGGACATTCTATCGAATGCCGTATCAACGCAGAAGACGCGTTTAAGAATTTTAGACCTGGGCCAGGTAGAATTACAGCATACTTGCCCTCTGGAGGTCCATTTGTCAGAATGGATAGCCATGTTTATCCCGATTATGTG‑‑‑GTTCCTCCAAGCTATGACTCCCTTCTTGGAAAGCTGATTGTATGGGCTCCAACAAGAGAAAAAGCAATTCAACGCATGAAAAGGGCACTTGATGACACTACTATCACAGGAATTCCTACAACAATCGAATATCATAAACTTATCCTTGACATTGAGGATTTCAGAAATGGCAAGGTTGACACTGCTTTCATTCCAAAGCATGAGGAGGAATTGGCAATG‑‑‑CCACCCCAGAAG‑‑‑ATTGTACCATCT‑‑‑AACAGG‑‑‑‑‑‑‑‑‑‑‑‑‑‑‑‑‑‑‑‑‑‑‑‑‑‑‑GCT

>Lan_accC1_Lup032034

ATGGATTCCACTATTTCAATGTCA‑‑‑CTCTGCAAATCTCTTTCTTCACCTTCTGTT‑‑‑CCT‑‑‑‑‑‑CTTGTAGTAAGTCCAATTACTCAATCATCTAGAGGAATG‑‑‑AAGAGT‑‑‑‑‑‑TCA‑‑‑CAATGC‑‑‑AGTTTCTTACCT‑‑‑GGAACTAATAACATGAGTTTTCCTAAACAAACTGCTGTTCAACAA‑‑‑GTTAGTTAT‑‑‑AAAAAAGAACAAAGAGCG‑‑‑‑‑‑CGTTGTGGGGCTCTTGGTGCAACTTGT‑‑‑‑‑‑‑‑‑‑‑‑CGA‑‑‑GCGGATAAGATTTTGATTGCGAATAGAGGTGAAATCGCGGTTCGTGTTATTCGAACTGCTCATGAATTGGGGATACCATGTGTGGCAGTGTACTCTACAATTGACAAGGATGCACTTCATGTTAAATTGGCTGATGAGGCTGTTTGTATTGGCGAAGCGCCAAGTAACCAATCGTACTTGCTTATCCCAAATGTCCTTTCTGCTGCTACTAGTCACAAATGCACGATGTTGCATCCTGGATATGGTTTTCTTTCTGAGAATGCAGTGTTTGTTGATATGTGTAGAGAACATGGAATCAATTTTATTGGGCCTAATCCTGACAGTATTCGGGTTATGGGTGACAAAGCAACTGCCCGAGAGACAATGAAGAAAGCAGGTGTTCCCACTGTTCCAGGAAGTGATGGGCTTTTACAGAGCACAGAAGAAGCTGTCAAGGTTGCAAATGAGATTGGTTTCCCTGTGATGATCAAGGCAACAGCAGGTGGCGGAGGACGTGGCATGCGTCTTGCTAAAGAACCTGAAGAGTTTGTGAAGTTGCTACAGCAAGCTAAGAGTGAAGCAGCTGCTGCCTTTGGCAATGATGGTGTTTATCTGGAGAAGTATGTCCAAAACCCAAGACACATTGAGTTCCAGGTTCTTGCAGACAAATATGGTAATGTTGTTCACTTTGGAGAACGTGATTGCAGCATCCAGAGGCGTAATCAGAAACTGATGGAAGAAGCACCTTCTCCTGCATTGACCCCAGAGTTGCGGAAGGCAATGGGAGATGCAGCAGTTGCAGCGGCTGCATCTATTGGTTATGTTGGTGTTGGAACAGTTGAGTTTCTATTGGATGAAAGGGGTTCCTTTTACTTCATGGAGATGAACACTCGTATTCAGGTTGAACATCCTGTTACAGAAATGATTTCCTCTGTGGATTTGATAGAAGAGCAAATTCTTGTAGCTATGGGAGAAAAACTTCGA‑‑‑‑‑‑‑‑‑‑‑‑‑‑‑TACAAACAGGAGGATATTGTGCTCCGTGGACATTCTATTGAATGCCGTATCAATGCAGAAGATGCGTTTAAGAATTTTAGACCTGGGCCAGGTAGAATTACAGCATACTTGCCATCTGGAGGTCCATTTGTCAGAATGGATAGCCATGTTTATCCCGATTATGTG‑‑‑GTTCCTCCAAGCTATGACTCCCTTCTTGGGAAGCTGATTGTATGGGCTCCAACAAGAGAAAAAGCAATCGAGCGCATGAAAAGGGCACTTGATGACACTACTATCACAGGAATTCCTACAACTATCGAATATCATAAACTTATCCTTGACATTGAGGATTTCAGAAATGGCAAGGTTGACACTGCTTTTATTCCAAAGCATGAGGAGGAATTGGCAATG‑‑‑CCACCCCAGAAG‑‑‑ATTGTACCATCT‑‑‑AACAGG‑‑‑‑‑‑‑‑‑‑‑‑‑‑‑‑‑‑‑‑‑‑‑‑‑‑‑GCA

>Lan_accC2_Lup005680

ATGGAGGCCACAATGTCAATGTCT‑‑‑GTATGCAAATCTCTTCACTCCCCTTCTTCT‑‑‑CCT‑‑‑‑‑‑ATC‑‑‑ACAGGTTTATTTGGA‑‑‑‑‑‑‑‑‑‑‑‑GGAATC‑‑‑AAGGGT‑‑‑‑‑‑TCT‑‑‑GAATGCAGAAGCTTCTTAGTT‑‑‑AGAACCAATAAGGTGAATTTTCCTAAGCAAAATGTTAACAAGAAAGGAGTTAGTTAC‑‑‑AAACAAGGGCAACATAAAACGCAGCGTTTTGGGGCTCTTGCTGCTACTTGT‑‑‑CGTGGTGGTGGT‑‑‑GGGGATAAGATTCTGATTGCGAATCGAGGCGAGATCGCGGTTCGTGTGATTCGTACTGCTCATGAGTTGGGGATACCTTGTGTGGCAGTGTACTCAACTATTGATAAGGATGCACTTCATGTTAAATTGGCTGATGAGGCTGTTTGCATTGGCGAAGCGCCGAGTAGTCAATCGTACTTGCTGATCCCGAATGTCCTTTCTGCTGCTATTAGCCACAAATGCACGATGTTGCATCCTGGATATGGTTTTCTTGCTGAGAATGCGGTGTTTGTTGAGATGTGCAGAGAACATGGAATCAATTTTATTGGGCCTAATCCTGACAGTATTCGGGTTATGGGTGACAAAGCAACTGCCCGGGAGACAATGAAGAAAGCAGGTGTTCCCACTGTTCCAGGAAGTGATGGGCTTTTACAGAGCACAGAAGAAGCTATCAGGCTCGCACATGAGATTGGGTTCCCCGTGATGATCAAGGCAACAGCAGGTGGCGGAGGACGTGGCATGCGTCTTGCTAAAGAACCTGATGAGTTTGTGAAGTTGTTACAGCAAGCTAAGAGCGAAGCAGCTGCTGCCTTTGGCAATGACGGTGTTTATCTTGAGAAGTATGTTCAAAACCCAAGGCACATTGAGTTCCAGGTTCTTGCAGATAAATATGGTAATGTCGTTCACTTTGGAGAACGTGATTGCAGCATCCAGAGGCGTAATCAGAAACTGCTGGAAGAAGCACCATCTCCTGCATTGACCCCAGAGTTGCGGAAGGCAATGGGAGATGCAGCAGTTGCAGCGGCTGCATCTATTGGTTACATTGGTGTTGGAACAGTTGAGTTCCTATTGGATGAAAGGGGTTCCTTTTACTTCATGGAGATGAACACTCGTATTCAGGTTGAACATCCTGTCACAGAAATGATTTCCTCTGTTGATTTGATAGAAGAGCAAATTCTTGTAGCCATGGGAGAAAAACTTCGA‑‑‑‑‑‑‑‑‑‑‑‑‑‑‑TACAAACAGGAGGATATTGTGCTCCGAGGACATTCTATCGAATGCCGTATCAATGCAGAAGATGCATTTAAGAATTTTAGACCTGGGCCAGGTAGAATTACAGCATACTTGCCATCTGGAGGTCCATTTGTCAGAATGGATAGCCATGTTTATCCCGATTATGTG‑‑‑GTTCCTCCAAGCTACGACTCCCTTCTCGGAAAGCTGATAGTATGGGCTCCAACAAGAGAAAAAGCAATTGAACGCATGAAAAGGGCACTTGATGACACTATTATCACAGGAGTTCCTACAACAATTGATTATCATAAACTTATCCTTGACATTGAGGATTTCAGAAGTGGCAAGGTTGACACTGCTTTTATTCCAAAGCATGAGGAGGAGTTGTCAATG‑‑‑CCACCACAGAAG‑‑‑ATTGTACCATCC‑‑‑AAAAGG‑‑‑‑‑‑‑‑‑‑‑‑‑‑‑‑‑‑‑‑‑‑‑‑‑‑‑GCA

>Lja_accC_Lj4g3v3015020

ATGGAGGCCACC‑‑‑‑‑‑ATGTCC‑‑‑GCCTGCAAATCAATCACCTCCTCTTCCGTT‑‑‑CCC‑‑‑‑‑‑GTC‑‑‑ACAGGTTTATTCGCGGGAAGA‑‑‑CGAGGAGTG‑‑‑AAGAGT‑‑‑‑‑‑TCCACCCAATGC‑‑‑AGCTTCTTAGCT‑‑‑GAAACCAACAAGGTCAAATTCCCGAGGCAAGCTGCT‑‑‑CAGTTC‑‑‑AACAAGCAG‑‑‑CACCACCAGACTCGCCGC‑‑‑‑‑‑CACTGCGGCCCTCTCCGCGTGACTTGC‑‑‑GGTGGCGGCAGC‑‑‑GGCGAGAAGATCCTCATTGCTAACAGAGGCGAGATCGCCGTCCGTGTCATTCGTACCGCCCATGAATTGGGGATTCCGTGTGTGGCTGTGTACTCCACCATTGACAAGGACGCGCTTCATGTCAAGCTGGCTGATGAAGCCGTTTGCATTGGTGAAGCACCCAGTAGCCAATCGTATTTGCTGATCCCGAATGTTCTAGCTGCTGCTATTAGCCGCAAATGCACAATGTTGCATCCCGGTTATGGTTTCCTTGCTGAGAATGCAGTGTTTGTTGAGATGTGTAGGGAACATGGAATCAATTTTATTGGGCCTAATGCTGACAGTATTCGGGTTATGGGTGACAAAGCAACTGCCAGAGAAACAATGAAGAAAGCAAATGTTCCTACTGTTCCAGGAAGTGAGGGGCTTTTACAGGGTACAGAAGAAGCTATCAGACTTGCACATGAGATTGGTTTCCCTGTGATGATCAAGGCAACAGCTGGTGGTGGAGGACGTGGCATGCGTCTTGCTAAAGAACCTGAGGAGTTTGTGAAGTTGTTACAGGAAGCTAAGAGTGAGGCAGCTGCTGCATTTGGCAACGATGGAGTTTATTTGGAGAAGTACATTCAGAACCCAAGGCACATTGAGTTCCAGGTTCTTGCCGATAAATTTGGTAATGTTGTTCACTTTGGTGAGCGTGATTGCAGTATCCAGGGGCGTAATCAGAAACTTCTTGAAGAAGCACCATCTCCTGCATTGACCCCCGAGTTGCGAAAGGCAATGGGAGACGCAGCAGTTCAAGCTGCTGCATCAATTGGTTACATAGGAGTTGGAACGGTTGAGTTCCTTTTGGATGAAAGGGGTTCATTTTACTTCATGGAGATGAACACACGTATCCAGGTTGAACATCCTGTGACAGAAATGATTTCCTCTACTGATTTGATAGAAGAGCAAATTCGTGTAGCTATGGGTGAAAAACTACGA‑‑‑‑‑‑‑‑‑‑‑‑‑‑‑TACAAACAGGAGGACATTGTTCTCAGAGGACATTCTATTGAATGCCGTATCAATGCAGAAGATGCTTTTAAGGGGTTTAGACCAGGGCCAGGTAGAATAACAGCATACTTGCCATCTGGAGGTCCATTTGTCAGAATGGATAGCCATGTTTATCCTGATTATGTA‑‑‑GTTCCTCCAAGCTATGACTCCCTTCTTGGAAAGGTGATTGTATGGGCACCAACAAGAGAAAAAGCAATTGAGCGCATGAAACGGGCACTTGATGACACTATCATCACAGGAGTTCCTACAACAATTGATTATCATAAACTTATCCTTGACATTGAGGATTTCAAAAATGGCAAGGTTGATACTGCTTTTATTCCAAAACACGAGGAGGAGTTGGCGGTGGCGCCGCCGCAAAAG‑‑‑AGGGTACTTTCT‑‑‑AACACG‑‑‑ACGAAAGAGCTAAATGGCGCAACTGCC

>Mtr_accC_Medtr8g101330

ATGGAGGCAACA‑‑‑‑‑‑ATGTCCGCAGCCTGCAATTCTCTCACCTCACCATCCACC‑‑‑‑‑‑‑‑‑‑‑‑GTC‑‑‑ACAGGTTTATTTGCGACGAGAGGAGGAGGATTGAACAAGAATTCACATTCT‑‑‑CAGTGC‑‑‑ACTTTCTTGACC‑‑‑GGAACAAACAAGCTGAAGTTTCCGAGGCAAACTACT‑‑‑CAACCT‑‑‑TGT‑‑‑‑‑‑‑‑‑AAACAACGCCAAACGCGT‑‑‑‑‑‑CATTGCGGTGCTCTTCGTGCAACTTGT‑‑‑‑‑‑‑‑‑‑‑‑CGT‑‑‑CTTGAGAAGATATTGGTTGCCAATCGAGGCGAGATTGCTGTTCGTGTTATTCGTACTGCTCATGAATTGGGGATTCCTTGTGTTGCTGTTTATTCCACCATTGATAAAGATGCTCTTCATGTTAAATTGGCTGATGAATCTGTTTGTATTGGTGAAGCTCCCAGTAGCCAATCGTATTTAGTGATTCCAAATGTTCTGGCTGCTGCTACCAGCCGCCAATGTACAATGTTGCATCCTGGATATGGTTTTCTTGCAGAGAATGCTGTTTTTGTTGAAATGTGTAGAGATCATGGAGTCAATTTTATTGGACCTAACCCTGACAGTATTCGGGTTATGGGTGACAAAGCAACTGCAAGAGAAACAATGAAGAAAGCAAATGTTCCTACTGTTCCAGGAAGTGAGGGGCTTTTGCAGACCACTGAAGAAGCTATCAGACTGGCACACGAGATTGGTTTCCCTGTGATGATCAAGGCAACCGCAGGTGGTGGAGGACGTGGCATGCGTCTTGCTAAAGAACCTAAAGAGTTTGTGAAGTTATTACAGCAAGCTAAGAGTGAGGCAGCTGCTGCATTTGGAAATGATGGGGTTTATTTGGAGAAGTACATTCAAAACCCAAGGCACATTGAATTCCAGGTTCTTGCAGATAAATATGGTAATGTTGTTCACTTTGGAGAACGTGATTGCAGTATCCAGAGGCGTAATCAGAAACTGCTCGAAGAAGCACCGTCTCCTGCATTGACCCCAGAGTTACGGAAGGCAATGGGAGATGCAGCAGTTCAGGCTGCCGCATCTATCGGTTACATAGGTGTTGGAACGGTTGAGTTCCTTTTGGATGAAAGGGGTTCTTTTTACTTCATGGAGATGAACACTCGTATCCAGGTTGAACATCCCGTGACAGAAATGATTTCCTCAGTTGATTTGATAGAAGAGCAAATTCGTGTAGCTATGGGAGAAAAGCTTCGA‑‑‑‑‑‑‑‑‑‑‑‑‑‑‑TACAAACAGGAGGATATTGTACTCAGAGGACATTCCATTGAATGCCGTATCAATGCAGAAGATGCTTTCAAGGGATTTAGACCAGGGCCAGGTAGAATTACAGCATACTTGCCGTCTGGTGGTCCATTTGTCAGGATGGATAGCCATATTTATCCTGATTATGTT‑‑‑GTTCCTCCAAGCTATGACTCTCTTCTTGGAAAGCTGATTGTATGGGCTCCGACTAGAGAAAAAGCAATTGAACGCATGAAAAGGGCACTCGATGACACTATTATCACAGGAGTTCCTACAACAATTGATTATCATAAACTTATCCTTGACATAGAGGATTTCAAAAATGGCAAGGTTGATACTGCTTTTATTCCAAAGCACGAGGAGGAGTTGGCTGTG‑‑‑CCCCCACAACAGAAGATGGTATTAGTT‑‑‑AACAAGGTGGTGACAGAACCAGCTGGCTCGACTGCC

>Pvu_accC_Phvul.002G302900

ATGGAAGTCACA‑‑‑‑‑‑CTCTCC‑‑‑GCCTGCAAGTCCGTTACCTCGCCTTCTCTT‑‑‑CCC‑‑‑‑‑‑GTC‑‑‑GCAGGCTTATCTGCGAGGAAGGGA‑‑‑GGGATT‑‑‑AAGAGT‑‑‑‑‑‑TCG‑‑‑CAATGC‑‑‑AGTTTCTTAGCA‑‑‑GGAACGAACAGAGTGAAGTTTCCTAGGCAAGTAGGT‑‑‑CAAGTT‑‑‑AGTCACGTCCGCAAACAGCGCCAAACGCGT‑‑‑‑‑‑CACTGTGGTGCTCTCCACGCGACTTGT‑‑‑‑‑‑‑‑‑‑‑‑AGC‑‑‑GGCGATAAGATCCTTATCGCGAACAGAGGCGAGATTGCGGTTCGAGTGATTCGAACCGCCCGTGAATTGGGGATTCCTTGCGTGGCTGTGTACTCCACCATTGATAAGGACTCGCTTCATGTCAAATTGGCTGATGAAGCTGTTTGCATTGGCGAAGCTCCGAGTAACCAATCGTACTTGCTGATTCCGAATGTTCTGTCTGCTGCTCTTAGCCGCAATTGCACGATGTTGCATCCTGGCTATGGTTTTCTTGCGGAGAATGCTCGGTTTGTTGAAATGTGCTGCGGTCATAGAATCAACTTTATCGGGCCTAAACCTGATAGTATTCGGGTTATGGGTGACAAAGCAACTGCCAGAGAAACAATGAAGAAAGCAGGTGTTCCCACTGTTCCAGGAAGCGAGGGTCTTTTACAGAGCACAGAGGAAGCTATCAGGCTGGCAAAAGAGATTGGTTTCCCTGTGATGATCAAGGCAACAGCTGGTGGTGGAGGACGTGGCATGCGTCTTGCTAAAGAACCTGATGAATTTGTGAAGTTGTTGCAGCAAGCTAAGAGTGAAGCCGCCGCTGCATTTGGCAATGATGGTGTTTATCTGGAGAAGTACATTCAAAACCCAAGGCACATTGAGTTCCAGGTTCTTGCCGATAAACATGGTAACATCGTTCACTTTGGAGAACGTGATTGCAGCATCCAGAGGCGTAATCAGAAGCTGCTAGAAGAAGCACCATCTCCTGCCCTGACCCCTGAGTTGCGAAAGGCAATGGGAGATGCAGCAGTTGCAGCTGCTGCATCTATTGGTTACATAGGTGTTGGAACAGTGGAGTTCCTTTTGGATGAAAGGGGTTCCTTTTACTTCATGGAGATGAACACTCGTATCCAGGTTGAGCATCCTGTGACAGAAATGATTTCCTCAACAGATTTGATAGAAGAGCAAATTCGTGTTGCTATGGGAGAAAAACTTAGA‑‑‑‑‑‑‑‑‑‑‑‑‑‑‑TTTAAACAGGAGGATATTGTGCTCAGAGGACATTCTATTGAATGTCGTATCAATGCTGAAGATGCTTTTAAGGGATTTAGACCAGGGCCAGGTAGAATTACAGCATACTTGCCATCTGGAGGTCCATTTGTCAGAATGGATAGCCATGTTTATCCTGATTATGTT‑‑‑GTTCCTCCAAGCTATGACTCCCTTCTTGGAAAGCTGATTGTTTGGGCTCCAAATAGAGAAAAAGCCATTGAACGCATGAAAAGGGCTCTTGATGACACAGTTATCACAGGAGTTCCTACAACAATTGATTATCATAAACTTATCCTTGACATAGAGGATTTCAGAAATGGCAAAGTTGACACCGCTTTTATTCCAAAACACGAGGAAGAGTTGTCAATG‑‑‑CCGCCGCCACCG‑‑‑AAGATAGTACCG‑‑‑AGC‑‑‑‑‑‑‑‑‑‑‑‑‑‑‑‑‑‑‑‑‑‑‑‑‑‑‑‑‑‑‑‑‑

>Vra_accC_Vradi07g29170

ATGGAAGTCACA‑‑‑‑‑‑CTCTCC‑‑‑GCATGCAAGTCCGTTAGCTCGCCTTCTCTT‑‑‑CCC‑‑‑‑‑‑ATC‑‑‑TCAGGTTTATCTGGGAGGAAGGGA‑‑‑GGGATT‑‑‑AAGAGT‑‑‑‑‑‑TCG‑‑‑CAATGC‑‑‑AGTTTCATATCA‑‑‑GGAACGAACGGAGTGAGGTTTCCTAGACAAGTAAGT‑‑‑CAGGTT‑‑‑ACTCACCTTCGCAAACAGCGCCAAACGCGT‑‑‑‑‑‑CACTGTGGTGCTCTCCACGCGACTTGT‑‑‑‑‑‑‑‑‑‑‑‑AGC‑‑‑GGTGAGAAGATCCTGATCGCGAACAGAGGCGAGATTGCGGTTCGAGTGATTCGAACCGCTCGTGAAATGGGGATTCCTTGCGTAGCTGTGTACTCCATCATTGATAAGGACGCGCTTCATGTCAAATTGGCTGATGAAGCTGTTTGCATTGGCGAAGCGCCGAGTAACCAATCGTACTTGCTGATTCCGAATGTTCTGTCTGCTGCTATTAGCCGCAAATGCACGATGTTGCATCCCGGCTATGGTTTTCTTGCGGAGAATGCCGTGTTTGTTGAAATGTGCTGGGAACATGGAATCAACTTTATTGGGCCTAAACCTGATAGTATTCGGGTTATGGGTGACAAATCAACTGCCAGAGAAACAATGAAGAAAGCAGGTGTTCCCACTGTTCCAGGAAGCGAGGGTCTTTTACAGAGCACAGAAGAAGCTATCAGGCTGGCAAATGAGATTGGTTACCCTGTGATGATCAAGGCAACAGCTGGTGGTGGAGGACGTGGCATGCGTCTTGCTAAAGAACCTGATGAATTTGTGAAGTTGTTGCAGCAAGCTAAGAGTGAAGCCGCTGCTGCATTTGGCAATGATGGGGTTTATTTGGAGAAGTACATTCAAAACCCAAGGCACATTGAGTTCCAGGTTCTTGCAGATAAATATGGTAATGTTGTTCACTTTGGAGAACGTGATTGCAGCATCCAGAGGCGTAATCAGAAACTGCTGGAAGAAGCACCGTCTCCTGCCCTGACCCCTGAGTTGCGAAAGGCAATGGGAGATGCAGCGGTTGCAGCTGCTGCATCTATTGGTTACATAGGTGTTGGAACAGTGGAGTTCCTTTTGGATGAAAGGGGTTCCTTTTACTTCATGGAGATGAACACTCGTATCCAGGTTGAGCATCCCGTGACAGAGATGATTTCCTCAACAGATTTGATAGAAGAGCAAATTCGTGTTGCAATGGGAGAAAAACTTAGA‑‑‑‑‑‑‑‑‑‑‑‑‑‑‑GTCAAACAGGAGGATATTGTGCTCAGAGGACATTCTATTGAATGTCGTATCAATGCAGAAGATGCTTTTAAGGGATTTAGACCAGGGCCAGGTAGAATTACAGCATACTTGCCATCTGGAGGCCCATTTGTCAGAATGGATAGCCATGTTTATCCTGATTATGTT‑‑‑GTTCCTCCAAGCTATGACTCCCTTCTTGGAAAGCTGATTGTATGGGCTCCAAATAGAGAAAAAGCCATTGAACGCATGAAAAGGGCTCTTGATGACACAGTTATCACAGGAGTCCCTACCACAATTGATTATCATAAACTTATCCTCGACATAGAGGATTTCAGAAACGGAAAAGTTGATACTGCATTTATTCCAAAACATGAGGAAGAGTTGGCAATG‑‑‑CCTCCGCAGAAG‑‑‑TTGGTACTGAGC‑‑‑‑‑‑‑‑‑‑‑‑‑‑‑‑‑‑‑‑‑‑‑‑‑‑‑‑‑‑‑‑‑‑‑‑‑‑‑

# Table S16. Substitutions in ACCase paralogous comparisons.

Ka, nonsynonymous substitution rate; Ks, synonymous substitution rate.

| **Sequence pair** | **Ks** | **Ka** | **Ka/Ks** |
| --- | --- | --- | --- |
| Aar_ACC1, Aar_ACC2 | 0.0132 | 0.0005 | 0.04 |
| Gma_ACC1, Gma_ACC2 | 0.0935 | 0.0130 | 0.14 |
| Lal_ACC1, Lal_ACC2 | 0.0251 | 0.0060 | 0.24 |
| Lal_ACC1, Lal_ACC3 | 0.0264 | 0.0049 | 0.19 |
| Lal_ACC1, Lal_ACC4 | 0.1601 | 0.0256 | 0.16 |
| Lan_ACC1, Lan_ACC2 | 0.1958 | 0.0236 | 0.12 |
| Mtr_ACC1, Mtr_ACC2 | 0.2188 | 0.0529 | 0.24 |
| Adu_accA1, Adu_accA2 | 5.2901 | 0.3559 | 0.07 |
| Bto_accA1, Bto_accA2 | 2.3763 | 0.3887 | 0.16 |
| Cof_accA1, Cof_accA2 | 3.2340 | 0.3936 | 0.12 |
| Gma_accA1, Gma_accA2 | 0.0718 | 0.0371 | 0.52 |
| Gma_accA1, Gma_accA3 | 0.0726 | 0.0355 | 0.49 |
| Lal_accA1, Lal_accA2 | 0.0636 | 0.0347 | 0.55 |
| Lal_accA1, Lal_accA3 | 0.0495 | 0.0162 | 0.33 |
| Lal_accA1, Lal_accA4 | 0.0498 | 0.0123 | 0.25 |
| Lal_accA1, Lal_accA5 | 0.0239 | 0.0062 | 0.26 |
| Lan_accA1, Lan_accA2 | 0.2581 | 0.0656 | 0.25 |
| Lan_accA1, Lan_accA3 | 0.2474 | 0.0763 | 0.31 |
| Gma_accB1a, Gma_accB1b | 0.0901 | 0.0174 | 0.19 |
| Lal_accB1a, Lal_accB1b | 0.0117 | 0.0250 | 2.14 |
| Lal_accB1a, Lal_accB1c | 0.0117 | 0.0233 | 1.99 |
| Aar_accB2a, Aar_accB2b | 0.6723 | 0.3237 | 0.48 |
| Adu_accB2a, Adu_accB2b | 0.5815 | 0.1895 | 0.33 |
| Aip_accB2a, Aip_accB2b | 0.5769 | 0.1717 | 0.30 |
| Car_accB2a, Car_accB2b | 0.5182 | 0.1821 | 0.35 |
| Car_accB2a, Car_accB2c | 0.0284 | 0.0175 | 0.62 |
| Cca_accB2a, Cca_accB2b | 0.6080 | 0.2179 | 0.36 |
| Cca_accB2a, Cca_accB2c | 0.0646 | 0.0284 | 0.44 |
| Gma_accB2a, Gma_accB2b | 0.5693 | 0.2045 | 0.36 |
| Gma_accB2a, Gma_accB2c | 0.0771 | 0.0463 | 0.60 |
| Gpo_accB2a, Gpo_accB2b | 0.0417 | 0.0052 | 0.12 |
| Lal_accB2a, Lal_accB2b | 0.0499 | 0.0312 | 0.63 |
| Lal_accB2a, Lal_accB2c | 0.3584 | 0.0915 | 0.26 |
| Lal_accB2a, Lal_accB2d | 0.2309 | 0.0536 | 0.23 |
| Lal_accB2a, Lal_accB2e | 0.0383 | 0.0016 | 0.04 |
| Lal_accB2c, Lal_accB2d | 0.0817 | 0.0327 | 0.40 |
| Lan_accB2a, Lan_accB2c | 0.3020 | 0.0807 | 0.27 |
| Lja_accB2a, Lja_accB2b | 0.6046 | 0.2181 | 0.36 |
| Mtr_accB2a, Mtr_accB2b | 0.6396 | 0.2566 | 0.40 |
| Pvu_accB2a, Pvu_accB2b | 0.2268 | 0.0925 | 0.41 |
| Vra_accB2a, Vra_accB2b | 0.2031 | 0.0565 | 0.28 |
| Aar_accC1, Aar_accC2 | 0.0185 | 0.0185 | 1.00 |
| Adu_accC1, Adu_accC2 | 0.6113 | 0.0609 | 0.10 |
| Aip_accC1, Aip_accC2 | 0.6314 | 0.0737 | 0.12 |
| Bto_accC1, Bto_accC2 | 0.0258 | 0.0120 | 0.47 |
| Gma_accC1, Gma_accC2 | 0.1242 | 0.0082 | 0.07 |
| Lal_accC1, Lal_accC2 | 0.1286 | 0.0476 | 0.37 |
| Lal_accC1, Lal_accC3 | 0.1207 | 0.0437 | 0.36 |
| Lan_accC1, Lan_accC2 | 0.2194 | 0.0466 | 0.21 |

# Table S17. Results of branch-site model analysis of positive selection

| **Branch** | **lnL1 (alternative hypothesis)** | **lnL0 (null hypothesis)** | **LRT value** | **P value** | **FDR qvalue** | **Naive Empirical Bayesa** | **Bayes Empirical Bayes** |
| --- | --- | --- | --- | --- | --- | --- | --- |
| Arachis ACC | -41394.39327 | -41394.39327 | 0 | 1 | 0.82 |  |  |
| Glycine ACC | -41394.64286 | -41394.64286 | -2E-06 | 0.999 | 0.82 |  |  |
| Lupinus ACC | -41394.58255 | -41394.58255 | 0 | 1 | 0.82 |  |  |
| Arachis accA1 | -30055.41383 | -30055.41383 | 0 | 1 | 0.82 |  |  |
| Arachis accA2 | -30047.12745 | -30047.12745 | 0 | 1 | 0.82 |  |  |
| Glycine accA | -30044.30559 | -30046.94107 | 5.27 | 0.022 | 0.07 |  |  |
| Lupinus accA | -30055.41383 | -30055.41383 | 2E-06 | 0.999 | 0.82 |  |  |
| Bto cluster accA | -30037.81539 | -30051.24658 | 26.86 | 2.2E-07 | 0.00 | 075P|0.971* 182F|0.950* 411K|0.974* 723P|0.990* | 075P|0.930 182F|0.899 411K|0.947 723P|0.977* |
| Arachis accB1 | -6375.439546 | -6375.439546 | 0 | 1 | 0.82 |  |  |
| Glycine accB1 | -6372.582353 | -6373.580962 | 2.00 | 0.157 | 0.41 |  |  |
| Lupinus accB1 | -6374.569338 | -6375.439546 | 1.74 | 0.187 | 0.42 |  |  |
| Adu_accB2a, Aip_accB2b | -12277.87025 | -12282.19432 | 8.65 | 0.003 | 0.02 | 046F|0.961* 068A|0.990* | 046F|0.879 068A|0.975* |
| Adu_accB2b, Aip_accB2a | -12278.70168 | -12283.12677 | 8.85 | 0.003 | 0.02 | 257S|0.969* | 257S|0.961* |
| Glycine accB2 | -12283.66857 | -12283.66857 | 0 | 1 | 0.82 |  |  |
| Lupinus accB2 | -12283.66857 | -12283.66857 | 0 | 1 | 0.82 |  |  |
| Arachis accC1 | -12896.86484 | -12896.86483 | -1.8E-05 | 0.997 | 0.82 |  |  |
| Arachis accC2 | -12896.86483 | -12896.86484 | 2.2E-05 | 0.996 | 0.82 |  |  |
| Glycine accC | -12896.29789 | -12896.29789 | 0 | 1 | 0.82 |  |  |
| Lupinus accC | -12891.70028 | -12894.24801 | 5.10 | 0.024 | 0.07 | 445G0.998* | 445G0.990* |

a positions refer to sequence alignment provided in the Table S...

* statistically significant values (P≤0.05)

# Table S18. Gene expression values of legume cytosolic ACCase genes and subunits of plastid ACCase genes (normalized)

| Gene | Value | Nodule | Root |  | Mainstem leaf | Perianth | Fruit pattee 3 | Pericarp pattee 5 | Pericarp pattee 6 | Seed pattee 5 | Seed pattee 6 | Seed pattee 7 | Seed pattee 8 | Seed pattee 10 |
| --- | --- | --- | --- | --- | --- | --- | --- | --- | --- | --- | --- | --- | --- | --- |
| Aradu.GHK20 | Adu_ACC | 1201.3 | 825.3 |  | 1176.7 | 538.7 | 811.7 | 959.3 | 813.7 | 496.5 | 1293.5 | 788.0 | 534.0 | 236.0 |
| Araip.ZAK9V | Aip_ACC | 1448.0 | 1048.0 |  | 1320.0 | 475.7 | 867.3 | 1076.0 | 922.3 | 680.5 | 1564.0 | 893.5 | 666.5 | 309.5 |
| Gene | Value SD | Nodule | Root |  | Mainstem leaf | Perianth | Fruit pattee 3 | Pericarp pattee 5 | Pericarp pattee 6 | Seed pattee 5 | Seed pattee 6 | Seed pattee 7 | Seed pattee 8 | Seed pattee 10 |
| Aradu.GHK20 | Adu_ACC | 348.2 | 517.5 |  | 315.4 | 55.6 | 453.7 | 385.0 | 272.7 | 64.5 | 329.5 | 56.0 | 139.0 | 164.0 |
| Araip.ZAK9V | Aip_ACC | 376.9 | 681.6 |  | 475.2 | 70.0 | 481.7 | 434.5 | 328.0 | 64.5 | 290.0 | 114.5 | 237.5 | 216.5 |
| Gene | Value | Nodule reproductive | Root reproductive | Stem reproductive | Leaf reproductive | Flower |  | Immature pod | Mature pod |  | Immature Seed |  |  | Mature Seed |
| C.cajan_25715 | Cca_ACC | 15.8 | 39.4 | 18.9 | 41.4 | 12.7 |  | 102.3 | 14.3 |  | 80.1 |  |  | 10.3 |
| Gene | Value |  | Root 10d | Stem mature plant | Young leaves mature plant | Flower mature plant |  | Young pod mature plant |  |  |  |  |  |  |
| Ca_16623 | Car_ACC |  | 61.0 | 25.3 | 42.6 | 25.6 |  | 24.4 |  |  |  |  |  |  |
| Gene | Value | Nodule | Root |  | Young leaf | Flower | Pod 1 cm | Pod shell 10d | Pod shell 14d | Seed 10d | Seed 14d | Seed 21d | Seed 28d | Seed 35d |
| Glyma.04g104900 | Gma_ACC1 | 1.0 | 19.0 |  | 8.0 | 9.0 | 5.0 | 5.0 | 4.0 | 1.0 | 2.0 | 5.0 | 1.0 | 3.0 |
| Glyma.06g105900 | Gma_ACC2 | 2.0 | 14.0 |  | 6.0 | 9.0 | 5.0 | 5.0 | 4.0 | 1.0 | 2.0 | 3.0 | 2.0 | 5.0 |
| Gene | Value | Nodule | Root | Stem | Leaf | Flower |  | Pod 20d |  | Seed 10d | Seed 12d | Seed 14d | Seed 16d | Seed 20d |
| Lj0g3v0268829 | Lja_ACC | 18.0 | 58.2 | 22.8 | 56.0 | 19.1 |  | 19.4 |  | 20.7 | 20.2 | 18.1 | 20.5 | 14.6 |
| Gene | Value SD | Nodule | Root | Stem | Leaf | Flower |  | Pod 20d |  | Seed 10d | Seed 12d | Seed 14d | Seed 16d | Seed 20d |
| Lj0g3v0268829 | Lja_ACC | 2.5 | 7.5 | 2.2 | 10.0 | 0.7 |  | 2.1 |  | 3.0 | 2.8 | 1.4 | 0.5 | 0.1 |
| Gene | Value | Nod 28d | Root | Stem | Leaf | Flower |  | Pod |  | Seed 10d | Seed 16d | Seed 20d | Seed 24d | Seed 36d |
| Medtr3g073860.1 | Mtr_ACC2 | 1516.7 | 2202.1 | 2130.1 | 2431.2 | 1759.6 |  | 1400.2 |  | 2450.7 | 1739.2 | 2022.3 | 2015.0 | 863.8 |
| Gene | Value SD | Nod 28d | Root | Stem | Leaf | Flower |  | Pod |  | Seed 10d | Seed 16d | Seed 20d | Seed 24d | Seed 36d |
| Medtr3g073860.1 | Mtr_ACC2 | 29.1 | 143.6 | 159.8 | 49.8 | 304.4 |  | 119.4 |  | 237.2 | 172.4 | 265.0 | 80.0 | 213.3 |
| Gene | Value | Nodule | Root | Stem | Leaf | Young flower | Young pod | Pod 9cm | Pod 11 cm | Seed 4 mm |  | Seed 7 mm |  | Seed 10 mm |
| Phvul.009G028700.1 | Pvu_ACC | 11.0 | 71.0 | 23.0 | 26.0 | 22.0 | 6.0 | 21.0 | 15.0 | 52.0 |  | 15.0 |  | 9.0 |
| Gene | Value |  | Root | Stem | Leaf | Flower | Pod 6d | Pod 10d | Pod 16d | Seed 8d | Seed 10d | Seed 14d | Seed 18d |  |
| Vun_T00150.1 | Vun_ACC |  | 25.3 | 16.4 | 9.1 | 10.3 | 19.4 | 19.3 | 23.8 | 10.9 | 3.5 | 3.7 | 0.9 |  |
| Gene | Value | Nodule | Root |  | Mainstem leaf | Perianth | Fruit pattee 3 | Pericarp pattee 5 | Pericarp pattee 6 | Seed pattee 5 | Seed pattee 6 | Seed pattee 7 | Seed pattee 8 | Seed pattee 10 |
| Aradu.14CMN | Adu_accA1 | 505.3 | 1420.0 | 0.0 | 1521.7 | 56.7 | 519.3 | 435.0 | 341.0 | 1787.5 | 2291.0 | 1846.5 | 1281.5 | 103.5 |
| Araip.7H2NS | Aip_accA1 | 451.0 | 1224.0 | 0.0 | 1319.3 | 50.0 | 431.0 | 406.7 | 334.3 | 1683.0 | 2037.0 | 1687.5 | 1227.0 | 112.5 |
| Aradu.Z0G82 | Adu_accA2 | 1.7 | 2.7 | 0.0 | 244.7 | 15.3 | 2.3 | 0.7 | 0.0 | 1.5 | 1.5 | 0.0 | 0.0 | 0.0 |
| Araip.77JRH | Aip_accA2 | 0.7 | 7.3 | 0.0 | 477.0 | 22.7 | 1.3 | 0.7 | 0.3 | 2.0 | 0.0 | 0.5 | 0.0 | 0.0 |
| Gene | Value SD | Nodule | Root |  | Mainstem leaf | Perianth | Fruit pattee 3 | Pericarp pattee 5 | Pericarp pattee 6 | Seed pattee 5 | Seed pattee 6 | Seed pattee 7 | Seed pattee 8 | Seed pattee 10 |
| Aradu.14CMN | Adu_accA1 | 187.8 | 908.1 | 0.0 | 615.6 | 19.0 | 90.5 | 84.9 | 205.8 | 161.5 | 322.0 | 107.5 | 718.5 | 69.5 |
| Araip.7H2NS | Aip_accA1 | 180.1 | 770.4 | 0.0 | 502.5 | 14.4 | 70.7 | 111.2 | 192.1 | 139.0 | 331.0 | 174.5 | 568.0 | 72.5 |
| Aradu.Z0G82 | Adu_accA2 | 0.5 | 2.1 | 0.0 | 52.7 | 6.9 | 0.5 | 0.9 | 0.0 | 1.5 | 1.5 | 0.0 | 0.0 | 0.0 |
| Araip.77JRH | Aip_accA2 | 0.9 | 5.4 | 0.0 | 159.7 | 9.8 | 1.3 | 0.5 | 0.5 | 0.0 | 0.0 | 0.5 | 0.0 | 0.0 |
| Gene | Value | Nodule reproductive | Root reproductive | Stem reproductive | Leaf reproductive | Flower |  | Immature pod | Mature pod |  | Immature Seed |  |  | Mature Seed |
| C.cajan_18479 | Cca_accA1 | 3.7 | 1.8 | 12.3 | 11.0 | 1.0 |  | 10.2 | 8.9 |  | 4.7 |  |  | 4.5 |
| C.cajan_33027 | Cca_accA2 | 33.1 | 41.3 | 43.2 | 68.3 | 35.4 |  | 63.0 | 22.4 |  | 39.0 |  |  | 13.2 |
| Gene | Value |  | Root 10d | Stem mature plant | Young leaves mature plant | Flower mature plant |  | Young pod mature plant |  |  |  |  |  |  |
| Ca_26217 | Car_accA |  | 26.2 | 57.3 | 92.3 | 34.8 |  | 47.2 |  |  |  |  |  |  |
| Gene | Value | Nodule | Root |  | Young leaf | Flower | Pod 1 cm | Pod shell 10d | Pod shell 14d | Seed 10d | Seed 14d | Seed 21d | Seed 28d | Seed 35d |
| Glyma.18g195700 | Gma_accA1 | 1.0 | 4.0 |  | 6.0 | 3.0 | 3.0 | 4.0 | 2.0 | 2.0 | 3.0 | 2.0 | 2.0 | 4.0 |
| Glyma.18g195900 | Gma_accA2 | 1.0 | 3.0 |  | 10.0 | 3.0 | 3.0 | 3.0 | 3.0 | 2.0 | 2.0 | 4.0 | 1.0 | 2.0 |
| Glyma.18g196000 | Gma_accA3 | 1.0 | 2.0 |  | 4.0 | 2.0 | 2.0 | 2.0 | 2.0 | 1.0 | 1.0 | 1.0 | 1.0 | 2.0 |
| Gene | Value | Nodule | Root | Stem | Leaf | Flower |  | Pod 20d |  | Seed 10d | Seed 12d | Seed 14d | Seed 16d | Seed 20d |
| Lj0g3v0256879 | Lja_accA1 | 2676.3 | 1505.6 | 1380.8 | 1625.4 | 816.9 |  | 1697.6 |  | 1382.7 | 1268.3 | 1262.1 | 1426.4 | 2144.0 |
| Lj1g3v2628750 | Lja_accA2 | 23.7 | 43.5 | 171.2 | 284.5 | 133.5 |  | 42.4 |  | 56.6 | 60.7 | 50.0 | 52.7 | 21.0 |
| Gene | Value SD | Nodule | Root | Stem | Leaf | Flower |  | Pod 20d |  | Seed 10d | Seed 12d | Seed 14d | Seed 16d | Seed 20d |
| Lj0g3v0256879 | Lja_accA1 | 215.7 | 30.7 | 34.1 | 84.0 | 31.6 |  | 114.1 |  | 9.2 | 76.6 | 57.0 | 77.0 | 122.9 |
| Lj1g3v2628750 | Lja_accA2 | 3.1 | 11.2 | 17.7 | 22.8 | 2.4 |  | 1.8 |  | 3.7 | 1.8 | 1.2 | 7.6 | 1.2 |
| Gene | Value | Nod 28d | Root | Stem | Leaf | Flower |  | Pod |  | Seed 10d | Seed 16d | Seed 20d | Seed 24d | Seed 36d |
| Medtr7g066870 | Mtr_accA | 1148.3 | 1300.2 | 1351.0 | 1544.1 | 840.4 |  | 1297.6 |  | 1558.6 | 2173.6 | 1694.2 | 1145.9 | 670.3 |
| Gene | Value SD | Nod 28d | Root | Stem | Leaf | Flower |  | Pod |  | Seed 10d | Seed 16d | Seed 20d | Seed 24d | Seed 36d |
| Medtr7g066870 | Mtr_accA | 122.2 | 72.5 | 91.5 | 55.4 | 132.1 |  | 15.9 |  | 218.4 | 158.0 | 345.3 | 139.7 | 183.6 |
| Gene | Value | Nodule | Root | Stem | Leaf | Young flower | Young pod | Pod 9cm | Pod 11 cm | Seed 4 mm |  | Seed 7 mm |  | Seed 10 mm |
| Phvul.008G108700.1 | Pvu_accA | 24.0 | 30.0 | 33.0 | 38.0 | 39.0 | 17.0 | 48.0 | 15.0 | 38.0 |  | 30.0 |  | 19.0 |
[truncated: 28,284 more chars]
